# Two Faces of UV Mutagenesis: Independent and Collateral Mutagenesis in Skin Cancers

**DOI:** 10.3390/life16081300

**Published:** 2026-08-07

**Authors:** Konstantin Gunbin, Zamart Ramazanova, Bakhyt Matkarimov, Murat Saparbaev, Sergey Nikolaev, Andrey Yurchenko

**Affiliations:** 1 Center for Mitochondrial Functional Genomics, Immanuel Kant Baltic Federal University, 236016 Kaliningrad, Russia; genkvg@gmail.com; 2 Department of Electrical and Computer Engineering, School of Engineering and Digital Sciences, Nazarbayev University, Astana 010000, Kazakhstan; zamart.ramazanova@nu.edu.kz; 3 National Laboratory Astana, Nazarbayev University, Astana 010000, Kazakhstan; 4 L.N. Gumilyov Eurasian National University, Astana 010008, Kazakhstan; 5 Department of Molecular Biology and Genetics, Faculty of Biology and Biotechnology, Al-Farabi Kazakh National University, Almaty 050040, Kazakhstan; murat.saparbaev@gustaveroussy.fr; 6 Groupe «Mechanisms of DNA Repair and Carcinogenesis», CNRS UMR9019, Université Paris-Saclay, Gustave Roussy Cancer Campus, F-94805 Villejuif, France; 7 INSERM U981, Gustave Roussy Institute, Université Paris-Saclay, F-94805 Villejuif, France; sergey.nikolaev@gustaveroussy.fr

**Keywords:** basal cell carcinoma, melanoma, collateral mutagenesis, UV damage, non-UV mutational signatures

## Abstract

Cutaneous melanoma and basal cell carcinoma (BCC) represent the two primary malignancies driven by solar ultraviolet (UV) radiation. To map the spatial topology and distance dependence of their mutational signatures, we deployed new analytical bioinformatics workflow utilizing two convergent strategies: a data-driven read-level phasing framework to discriminate between collateral mutational events versus independent ones and a hypothesis-driven spatial permutational simulation model to capture density-dependent local deviations. A whole-genome analysis employing this framework unexpectedly reveals two fundamentally distinct lesion-processing landscapes, present in both BCC and melanoma. While independent mutations consistently reproduce canonical UV signatures (SBS7a–c) in both tumor types, collateral mutations tell a distinct and more varied narrative between BCC and melanoma. These collateral mutations are unusually abundant for non-UV characteristics, such as age-related SBS1 and SBS5, and display a notable 3′ to 5′ asymmetry near UV-induced lesions in pyrimidine dimers. We also demonstrated that BCC displays a pronounced enrichment of dinucleotide base substitutions flanking UV-signature sites on the 3′ side, particularly CA>TG and CG>TA changes. Ultimately, these topological patterns in both tumors indicate that a primary photoproduct seeds secondary mutagenesis within its local chromatin environment, dramatically altering our understanding of UV-induced lesion processing.

## 1. Introduction

Cell exposure to ultraviolet (UV) light is the primary driver of skin carcinogenesis, inducing DNA damage through distinct wavelength-dependent mechanisms. UVB radiation is directly absorbed by DNA, primarily generating two major types of dimeric lesions: cyclobutane pyrimidine dimers (CPDs) and pyrimidine (6–4) pyrimidone photoproducts (6-4PPs). Both pyrimidine dimers are cytotoxic—blocking DNA transcription and replication—and highly mutagenic, though CPDs occur at significantly higher frequencies than 6-4PPs [1,2]. Conversely, UVA radiation predominantly causes indirect DNA damage via the generation of reactive oxygen species, leading to oxidative DNA lesions. To maintain genomic integrity, cells rely on the nucleotide excision repair (NER) pathway, which encompasses global genomic NER for overall genome surveillance and transcription-coupled repair (TC-NER) for the rapid removal of bulky transcription-blocking lesions like CPDs and 6-4PPs. When these lesions evade repair, they form the biological basis of canonical UV mutational signatures (e.g., the SBS7 signature family). A specific hallmark of UV mutagenesis is the high frequency of C>T transitions at dipyrimidine sites, which is driven by the extremely high deamination rate of cytosine residues within unrepaired CPD sites in DNA [3]. Furthermore, these unrepaired CPD and 6-4PP lesions can trigger extended error-prone DNA transactions. Recent studies have described this phenomenon as “collateral mutagenesis”—the generation of tightly clustered multi-nucleotide substitutions arising from a single localized DNA damage event [4,5,6]. Building upon this concept, our research indicates that basal cell carcinoma (BCC) exhibits a ‘repair overrun’ pattern, where primary UV photoproducts accumulate faster than they can be removed, and the local sequence context directs the repair load towards the accumulation of collateral (secondary) substitutions.

Cellular genomes are continually challenged by endogenous and exogenous DNA-damaging agents, which are handled by a network of distinct repair pathways. When nucleotide excision repair (NER) or other excision-based systems are deficient or overwhelmed, unrepaired lesions accumulate and interfere with replication and transcription. To tolerate unrepaired DNA damage during cell replication, cells deploy translesion synthesis (TLS), a replication-dependent damage-tolerance pathway in which specialized DNA polymerases bypass lesions, unlocking stalled replication forks at the cost of increased mutagenesis [7]. Noteworthy, TLS DNA polymerases are distributive, lack a proofreading function, and possess spacious active sites, these make them highly error-prone when copying undamaged templates [8].

Beyond replication-coupled lesion bypass, mutations can arise from error-prone DNA synthesis during excision repair itself. The major mutagenic impact comes not from the excision itself but ssDNA gap filling after excision. If the repair patch is synthesized by an error-prone DNA polymerase, or if repair intermediates are processed incorrectly, the original damage can be converted into a permanent base substitution or small indel. This is particularly important for oxidative base modifications and bulky DNA adducts, where the wrong DNA polymerase choice can create mutations despite the fact that the lesion was successfully recognized and removed [9]. Indeed, the time-dependent accumulation of mutations in post-mitotic tissues may suggest the existence of replication-independent error-prone repair synthesis [10,11].

Recent studies have formally documented another phenomenon known as “collateral mutagenesis”—the generation of tightly clustered multi-nucleotide substitutions arising from one localized DNA damage event [4,5,6]. It has been proposed that collateral tracks either during DNA replication or during the aberrant repair outside of S phase could funnel diverse damage sources into shared mutational outcomes, severely confounding classical signature deconvolution. However, the precise extent to which collateral events shape the genomic landscapes of skin cancers—a classical example of UV-induced DNA damage-driven neoplasms—remains poorly understood. Decoding the full complexity of these mutational landscapes is essential, as the accumulation of such genomic alterations ultimately shapes the tumor immune microenvironment and dictates the efficacy of emerging therapeutic interventions, including advanced targeted delivery systems [12,13].

For a thorough analysis of collateral mutagenesis, we present a robust analytical workflow to dissect the spatial topology and distance dependence of mutational signatures in whole-genome sequences of basal cell carcinoma (BCC) and cutaneous melanoma. We deployed two independent convergent approaches: a data-driven read-level phasing framework that identifies clustered mutational events and a hypothesis-driven [14] spatial permutation model designed to capture density-dependent local deviations of mutation distribution. Both methods yielded identical highly congruent results, confirming that short-range collateral mutations represent a biologically distinct class enriched for specific nucleotide substitutions. Our analysis revealed BCC-specific patterns: the strong 3′ bias of non-UV mutations near YY dinucleotides indicates that lesion processing is asymmetric; the enrichment of double base substitutions (DBS) flanking UV-signature 3′ sites (CA>TG, CG>TA) suggests TLS-mediated tandem substitutions. Both tumor types exhibit a modest significant attraction to AA/TT and repulsion from CC/GG neighbor dinucleotides, reflecting AT-rich flexibility and GC-rich stability.

## 2. Results

### 2.1. The Selection of Samples for the Studied Cohort

For the analysis, we used the public cohorts of skin cancers, BCC and melanoma (see Section 4), as clear examples of DNA damage induced mutagenesis. To select tumor samples of non-ultraviolet (UV) origin, we first applied a two-step filtering process to our overall sample sets.

- The initial signature-based filtering based on COSIC signature decomposition. We selected samples whose mutational spectra closely matched known UV-associated signatures. Specifically, we required a cosine similarity of ≥0.9 to the COSMIC v3.4 SBS7a signature and ≥0.75 to the SBS7b signature.
- The outlier rejection via clustering. To ensure a homogeneous cohort, we next identified and removed atypical samples (outliers) from the filtered BCC and melanoma sets. This was achieved by (1) decomposing the mutational profile of each tumor into its constituent signatures, (2) clustering these signatures using an affinity propagation approach (Figure S1), and (3) iteratively removing samples identified as outliers until the dendrogram of the main cluster no longer exhibited multifurcations (i.e., a single coherent cluster was achieved) (Figure S1). Finally, we selected 99 samples in the BCC cohort and 107 samples in the melanoma cohort (Figure S2).

**Figure 1:**
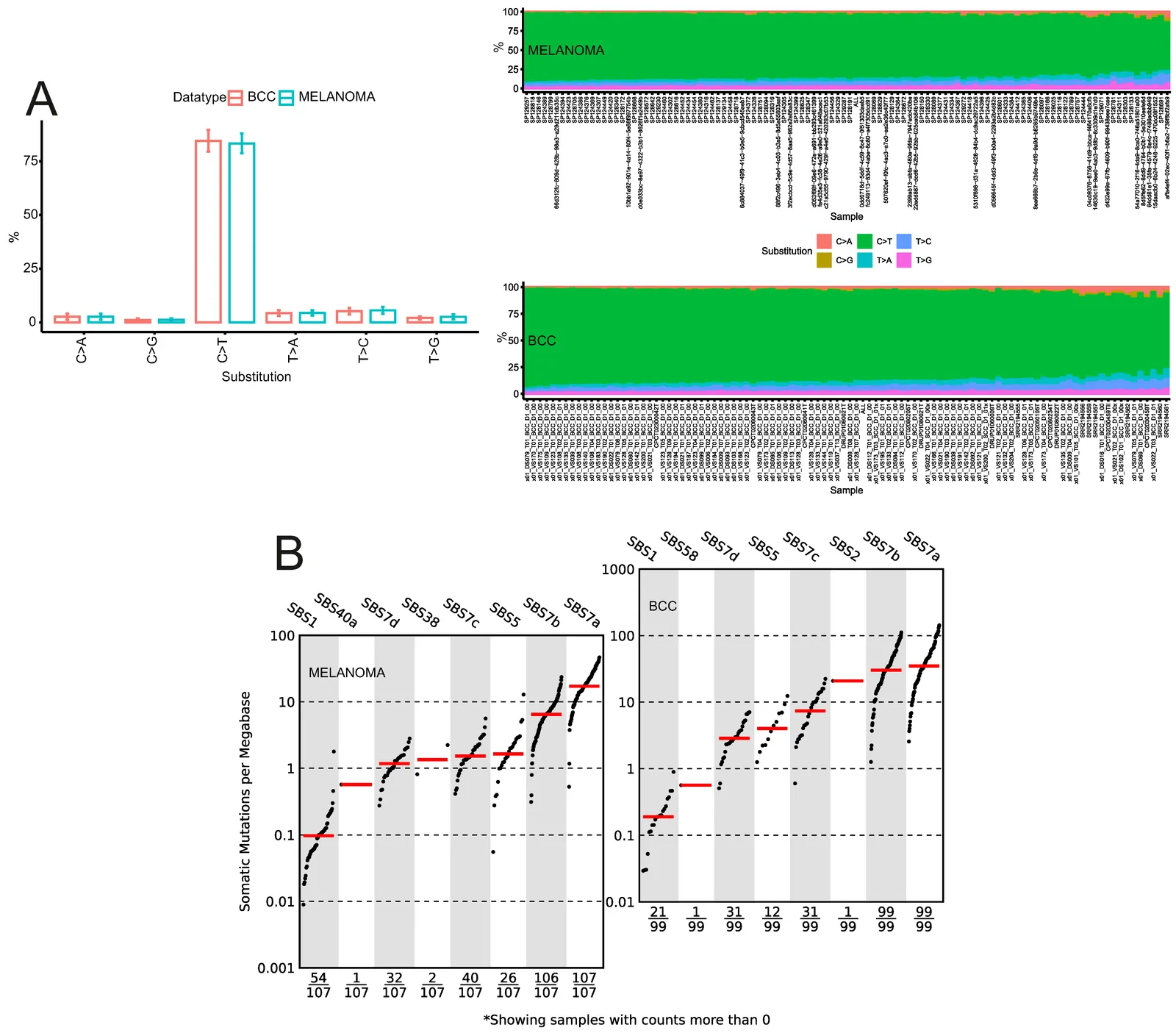
Mutational profile and signature analysis of basal cell carcinoma (BCC) and melanoma. (See Figure S1 for details about BCC and melanoma sample sets selection and Figure S2 for details about each selected tumor sample). (**A**). Proportion (%) of six types of nucleotide substitutions in 99 BCC samples and 107 melanoma samples. The bar chart on the left shows aggregate data for the melanoma and BCC sample sets—means values with confidence intervals. The plots on the right show the proportions of the six types of nucleotide substitutions for each individual sample in the melanoma and BCC sets. The “ALL” category in these plots represents the aggregated data for all samples. (**B**). Tumor mutation burden plots (number of mutational signatures per sample per 1 Mb of the genome) for mutational signatures observed in 99 BCC samples and 107 melanoma samples. The Y axes are aligned between the two plots.

At the nucleotide level, the selected samples of both tumor types exhibited a similar distribution across the six standard single-base substitution (SBS) classes. This spectrum was overwhelmingly dominated by a C>T transition pattern in the pyrimidine dimers (Figure 1A), a classic molecular hallmark indicative of ultraviolet (UV) light-induced DNA damage [15]. The mutational landscape of the studied cancers was dominated by the classical UV COSMIC signatures SBS7a and SBS7b (Figure S2). Despite sharing a predominant UV-driven etiology, the relative contributions of non-UV individual mutational signatures differed significantly between the two cohorts (Figure 1B):

- The baseline contributions of the non-UV signatures, such as SBS1 and SBS5, primarily characterized the profiles of melanoma SBS profiles.
- These clock-like signatures SBS1 and SBS5 exhibited a markedly higher activity level in the BCC cohort (up to three times in absolute numbers) than in the melanoma cohort.

### 2.2. Independent Mutational Events and Collateral Mutational Events Have Different Mutation Spectrums

In this part of the study, we examine whether closely spaced mutations that arose from a presumably single polymerase replication pass have different patterns compared to closely spaced mutations that arose from multiple replication passes. As a primary validation strategy, all analyzed variants were strictly required to pass all standard Mutect2 quality control filters. In order to robustly distinguish between collateral (serial or clustered) mutational events—such as those driven by low-fidelity translesion DNA synthesis (TLS) [4,6] and independent stochastic alterations, we implemented a data-driven method leveraging read-level phasing information from reference-aligned next-generation sequencing data. This approach establishes a rigorous experimental framework to classify proximal mutation pairs based on their underlying reads:

- Collateral mutagenesis: a pair of variants was classified as collateral (clustered) if two or more mutations co-occurred on the same sequencing read within a genomic distance cutoff of ≤100 base pairs, and this specific co-inheritance was confirmed across multiple independent reads supporting both mutant alleles. To minimize potential sources of false-positive classification arising from terminal sequencing errors or alignment artifacts, mutations falling on the extreme ends of reads were excluded from the analysis. This structural pattern presumably identifies a single localized mutagenic event that altered multiple nearby bases during a single synthesis track. While adjacent mutations captured on an isolated single read can theoretically occur through the coincidental overlap of two independent events (e.g., one mutation and other sequencing error), the probability of such stochastic co-occurrence decreases exponentially as the number of independent reads supporting the identical paired mutation pattern increases.
- Independent mutagenesis: conversely, mutations were classified as independent if each variant in the pair was supported by a distinct mutually exclusive set of sequencing reads. This distribution indicates that the variants arose from separate unlinked mutational processes on different chromosomes/alleles.

**Figure 2:**
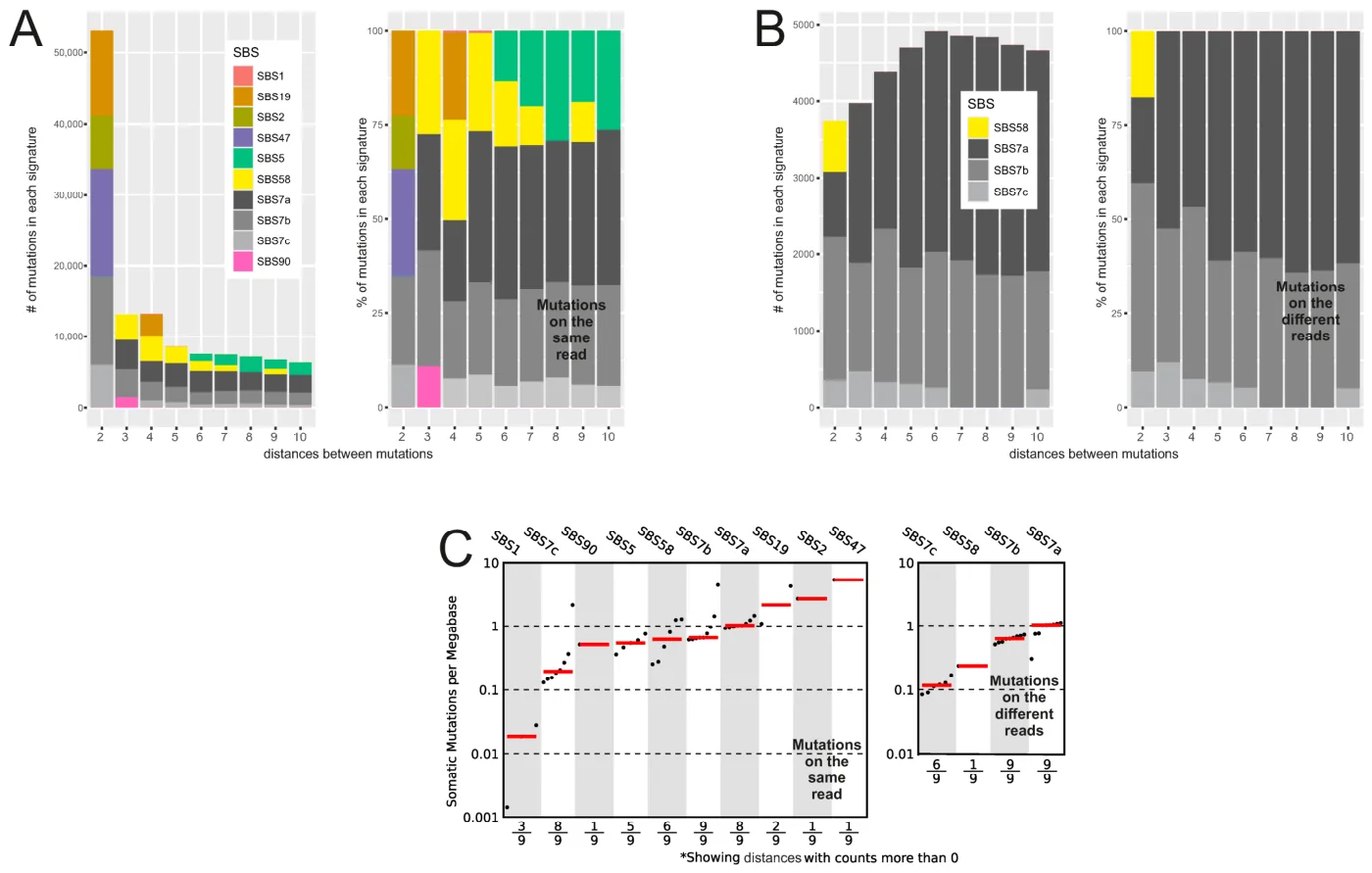
Relationship between inter-mutation distance and the landscape of COSMIC single-base-substitution (SBS) mutational signatures in basal-cell carcinoma (BCC). (**A**,**B**). Dependence of the number and type of mutational signatures in BCC tumors for paired mutations on the distance between them. (**A**)—paired mutations located on the same read; (**B**)—paired mutations located on two different reads. Left plot—number of mutational signatures in paired mutations; right plot—percentage of mutational signatures in paired mutations. (**C**). Modified tumor mutation burden plots (number of mutational signatures per sample per 1 Mb of the genome) for mutational signatures observed in paired mutations located on the same read or on two different reads. The Y axes are aligned between the two plots. The X axes represent the number of intervals between paired mutations where mutation signatures were observed.

To identify concurrent collateral mutations arising from the same replication event, we leveraged sequencing read information to determine whether closely located mutations occurred on the same read or on separate reads. This examination was conducted using whole-genome sequencing (WGS) data from 99 BCC tumor samples. We tracked the absolute and relative contributions of COSMIC Single-Base Substitution (SBS) signatures as a function of the genomic distance separating paired proximal mutations (Figure 2). To prevent confounding from multi-base substitutions, double- and triple-base substitutions (DBSs and TBSs) were explicitly excluded from this analysis. Additionally, to avoid overlapping trinucleotides in paired SBS, we analyzed inter-mutational intervals of at least 2. Panels 2A and 2B in Figure 2 display the distinctive COSMIC signature patterns across the paired SBS as the inter-mutational intervals become progressively larger. Panel 2C in Figure 2 details the specific tumor mutation burden (TMB) attributable to each identified COSMIC signature across the evaluated distance cohorts.

Our analysis resulted in the following three findings:

(1) Distance-dependent signature variation: both the absolute frequencies and relative proportions of somatic mutational signatures undergo systematic continuous shifts as the distance between paired nucleotide substitutions expands, indicating that the proximity is strongly coupled to distinct biochemical mechanisms.
(2) Event-type signature specificity: the mutational spectra diverge sharply when stratified by underlying read-level architectures:
  ○ Independent mutagenesis (mutations in close proximity to each other but supported by the non-overlapping set of reads): this category is overwhelmingly dominated (>90\%) by canonical ultraviolet (UV) light-associated signatures (SBS7a, SBS7b, SBS7c). Remarkably, their relative compositional fractions remain stable across all analyzed physical genomic distances.
  ○ Collateral mutagenesis (mutations in close proximity and located at the same reads): in stark contrast, co-localizing mutational events capture a highly diverse spectrum of etiologies. This includes classic UV signatures alongside age-related and rare processes (SBS1, SBS2, SBS5, SBS19, SBS47, SBS90, and SBS58). Furthermore, signatures linked to strand-coordinated mutagenesis and localized hypermutation (SBS2, SBS19, and SBS58) are highly sensitive to distance constraints within the collateral category, contributing stable proportions only at close-range intervals of 2–7 bp. Conversely, the ubiquitous clock-like signature SBS5 becomes progressively more dominant within collateral tracks as the inter-mutational distance widens to 5–10 bp.
(3) Distinct impact on mutational patterns of the collateral and independent mutagenesis: these disparate signature profiles directly shape divergent mutational patterns between collateral and independent events across the genome. This variance underscores the necessity of read-level phasing to accurately deconvolute the overlapping multi-layered mutagenic processes active in BCC (Figure 2C).

The high activity of non-UV and clock-like signatures (such as SBS5) within short-range collateral events may suggest that, while primary UV lesions initiate DNA damage, the resulting adjacent alterations are frequently generated by the error-prone footprints of, probably, translesion DNA synthesis (TLS) polymerases copying undamaged neighboring templates [4,5].

### 2.3. Separating Collateral Mutational Events from Independent Ones Through Permutation-Based Approach Leads to Identical Results: A Close Association with Translesion DNA Synthesis

To examine the spatial distribution of somatic variants, we established a hypothesis-driven approach evaluating the intervals between adjacent mutations. The underlying null hypothesis is the following: if all somatic mutations occur as strictly independent and stochastic events, the physical distance separating inter-mutational intervals will be determined solely by the sample-specific TMB; that is, a higher absolute mutation volume inherently compresses the expected spatial intervals [14]. To model this baseline random expectation, we performed in silico random permutations of all naturally occurring somatic mutations into the two evaluated cohorts (separately for BCC and melanoma cohorts). This approach effectively shuffled sample designations within the fixed genomic positions of all observed mutations, creating a robust mutation-density-controlled baseline.

Additionally, our hypothesis-driven analytical workflow was benchmarked against existing spatial mutagenesis frameworks, including SigFit [16], Helmsman [17], MutationalPatterns [18], and SigProfilerClusters [19]. While tools such as SigFit [16] and Helmsman [16] do not retain the chromosome coordinate-level resolution (to analyze distance between mutations) required for this specific analysis, and MutationalPatterns focuses primarily on region-specific density bins, SigProfilerClusters v. 1.2.3 (SigProfilerSimulator v. 1.2.2 [20], context: [‘6144’]) provides a comparable baseline for cluster detection (Supplementary File S1). However, our proposed approach demonstrated significant advantages for our specific application: by relying on natural inter-sample mutation permutations rather than synthetic intra-sample simulation, it achieved a >20-fold reduction in computational time (45 min vs. 16 h for the melanoma cohort; 1 h 20 min vs. 37 h for the BCC cohort; all computations were made on the AMD EPYC 7742 processor). Furthermore, our framework uniquely allows for the precise evaluation of mutational signature changes at exact nucleotide distances between paired mutations.

Our prior observations indicated that proximal mutations phased to the same sequencing read (collateral events) present a complex mixture of UV and non-UV etiologies, whereas variants split across different reads (independent events) are predominantly UV-induced. To maximize the resolution and specificity of this etiological differentiation, we refined our spatial mapping by analyzing mutations specifically within isolated dipyrimidine (YY) dimers, explicitly excluding mutations located within extended poly-pyrimidine (poly-Y) tracts. This filtering step establishes an absolute mathematical separation between direct UV photoproduct footprints and non-UV sequence contexts (characterized by a regular, rhythmic alternation of purines and pyrimidines). Furthermore, excluding poly-Y tracts eliminates spatial ambiguity. In extended pyrimidine runs, it is mathematically impossible to determine exactly which adjacent YY base pair sustained the initial photoproduct, which confounds precise inter-mutational distance calculations.

To integrate these structural criteria, we stratified all somatic variants into three mutually exclusive categories, allowing for a high-resolution unbiased comparison between observed spatial distributions and our permuted null model:

- Strict UV mutagenesis: Highly concordant mutation pairs displaying canonical UV-light signatures—specifically Y[C>T]R or R[G>A]Y transitions (where Y represents a pyrimidine and R a purine). Discordant pairs (e.g., a Y[C>T] R paired with an R[G>A]Y) are systematically excluded, as they manifest on opposite DNA strands and cannot physically represent a co-directional collateral event on a single read track. To maintain spatial precision, mutations failing the isolation criteria (i.e., relaxed UV patterns occurring within poly-Y tracts) are omitted from this category.
- Strict non-UV mutagenesis: Substitutions targeting a pyrimidine (Y) situated within a rigid alternating RYR trinucleotide context or a purine (R) positioned within a YRY trinucleotide context. This represents the cleanest structural signature of non-UV-associated background processes.
- ‘Other’ mutagenesis: Any remaining somatic variant not captured by the strict criteria of the two preceding classifications. This residual group serves as an essential repository for relaxed versions of both UV and non-UV mutation patterns, including those mapping within complex poly-Y or poly-R tracts.

**Figure 3:**
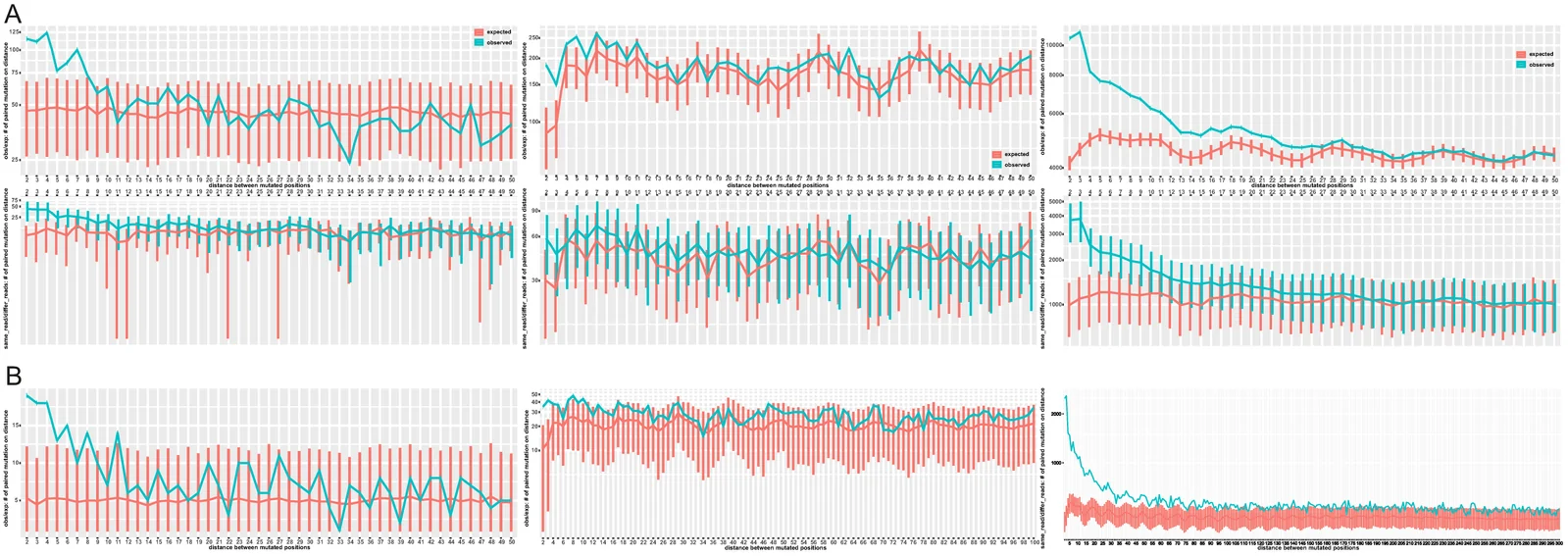
Spatial clustering of collateral mutation pairs in BCCs and melanomas. (See Figure S3 for details about those mutation pairs’ trinucleotide composition). (**A**). Dependence of the number of closely spaced mutation pairs in 99 BCC samples on the distance between them (distance is given as the nucleotide position from the nucleotide with the first mutation, which is assigned position 0). The upper plots compare the number of randomized mutation pairs (expected, not collateral) with the number of observed mutation pairs (observed, probably collateral); the lower plots compare the number of paired mutations on two different reads (expected, non-collateral) with the number of paired mutations on a single read (observed, collateral). (**B**). Dependence of the number of closely spaced collateral mutation pairs in 107 melanoma samples on the distance between them (distance is given as the nucleotide position from the nucleotide with the first mutation, which is assigned position 0). The plots compare the number of randomized mutation pairs (expected, not collateral) with the number of observed mutation pairs (observed, probably collateral). Columns of plots: (1) Left column—5′nonUV-UV3′ and 5′UV-nonUV3′ mutation pairs. (2) Middle column—UV–UV mutation pairs. (3) Right column—other mutation pairs not described by the two patterns above.

We evaluated the spatial distribution of distinct mutation pair classes in BCC cohort (Figure 3A). Each mutation class is displayed vertically as a column of plots, presenting two separate levels of resolution:

- Permutation-based comparison (upper row): Compares the absolute number of observed mutation pairs (blue line) against the null expectation generated by the permuted random model (red line, with a ±3 standard deviation spread).
- Read phasing-based comparison (lower row): Segregates the observed mutations into those localized to a single sequencing read (blue trace, ±3 standard deviation) versus those distributed across separate reads (red trace, ±3 standard deviation), with the latter serving as an empirical control for independent, unlinked events.

Our analysis of the BCC cohort identified three distinct spatial enrichment profiles (Figure 3A): (1) heteromorphic (5′nonUV-UV3′/5′UV-nonUV3′) pairs exhibited a clear short-range enrichment, peaking at an approximate 2.5-fold excess over the random baseline at a distance of 1 bp, before decaying to background levels by 8–10 bp; (2) canonical UV-UV pairs displayed a subtle modest enrichment (~1.5-fold), strictly confined to the immediate 1–3 bp window, indicating an absence of extended middle-range clustering.; (3) other mutational pairs demonstrated a pronounced long-range enrichment, where the observed mutation counts significantly exceeded random expectations up to an interval of 20–40 bp. Importantly, these spatial trends were fully congruent between our permutation-based and read phasing-based analysis approaches.

To test the pan-cancer generalizability of these spatial dynamics, we analyzed cutaneous melanoma datasets using the permutation-based framework (Figure 3B). The spatial enrichment profiles largely mirrored those found in BCC, albeit with distinct cohort-specific variations: (1) heteromorphic pairs demonstrated a sharp four-fold excess over the random baseline at 1 bp, followed by an exponential decay back to background levels by approximately 10 bp; (2) UV–UV pairs replicated the modest enrichment (~1.8-fold), restricted to the narrow 1–3 bp physical window; (3) other pairs similarly demonstrated a highly elevated and persistent excess across the entire analyzed distance spectrum.

The consistent accumulation of tightly spaced (≤8 bp) pairs—specifically within the heteromorphic and “other” mutation categories—indicates that these clusters reflect true collateral mutagenesis tracks originating from a single primary lesion. This observation aligns with the processive behavior of error-prone translesion DNA synthesis (TLS) polymerases resolving a single lesion into multiple base substitutions.

To determine whether these collateral tracks display orientation bias relative to the primary damage site, we quantified the flanking nucleotide frequencies of mutated trinucleotides across key proximal distance groups (Figure S3):

1. Asymmetry in heteromorphic pairs: Within the 5′nonUV-UV3′/5′UV-nonUV3′ class, C>T transitions in the pyrimidine dimer situated at an inter-mutational distance of 2–3 bp were statistically overrepresented in the 3′ trinucleotide position. This positional bias implies that primary UV photoproducts, such as cyclobutane pyrimidine dimers (CPDs), introduce a localized helical distortion that selectively hypersensitizes the immediate 5′ flanking template to subsequent misincorporation events during bypass.
2. Opposing constraints in “other” pairs: At intervals of 2–4 bp, C>T transitions were preferentially skewed toward the 3′ position, whereas G>A mutations showed a complementary enrichment in the 5′ position. This difference might imply that multiple opposing enzymatic processes or stereochemical limitations control more intricate non-standard collateral effects, though further research is required for a definitive conclusion.
3. Universal flanking stereotype: Both heteromorphic and “other” mutation pairs shared a uniform sequence preference, exhibiting a strong bias for a pyrimidine (C/T) at the first (−1) position and a purine (A/G) at the third (+1) position of the mutated trinucleotide. This flanking stereotype was notably more potent in heteromorphic pairs, suggesting a highly specific sequence environment that facilitates or encourages error-prone TLS bypass, although more research is needed for a conclusive determination.

Thus, the identified asymmetric 3′ flanking bias strongly mirrors the established biochemical properties of translesion synthesis polymerases (such as Pol η or Pol κ), which frequently display altered fidelity downstream of a bulky adduct before switching back to high-fidelity replicative enzymes. Furthermore, the downstream stereochemical distortion induced by rigid UV photoproducts can explain why subsequent collateral errors are directionally funneled along the nascent strand.

### 2.4. A Qualitative Distinction Between Translesion DNA Synthesis in BCC and Melanoma Is Shown by the Directional Asymmetry of Non-UV Mutations Flanking UV-Significant YY-Dinucleotides

First of all, to investigate the mechanistic basis of the observed mutational asymmetry (for any mutations and for paired mutations with a genomic distance limit of ≤100 base pairs), we evaluated the impact of various genomic and epigenomic features using SigProfilerTopography v. 1.0.102 [21] with the default options. For both tumors, we identified a usual significant enrichment of mutations in untranscribed regions and on the lagging DNA strand, highlighting the prominent role of transcription-coupled nucleotide excision repair (TC-NER) and replication asymmetry. Notably, both the TC-NER-associated signatures and lagging-strand bias were more pronounced in BCC than in melanoma, suggesting a relatively higher UV-damage repair efficiency in the melanoma samples. Other structural factors, including nucleosome occupancy and replication timing, showed no statistically significant impact on the mutational distribution (Supplementary File S2; statistically significant results are shown).

To build upon our previous identification of a significant 3′/5′ directional asymmetry between non-UV and UV mutation pairs, we performed a high-resolution analysis of the spatial distribution of non-UV alterations flanking UV-induced lesions in pyrimidine dimers. We made a detailed analysis of the spatial bias of non-UV mutations located near UV mutations, specifically, near the YY-dinucleotide that is located in the first and second position of the UV-mutated trinucleotide. Because mutational data are conventionally reported relative to a single reference strand, observing a pair of mutations textually on the “left” or “right” does not inherently reflect the biological orientation. To establish true strand-resolved directional mapping, we corrected for the orientation of the underlying UV motif (YCR on the forward strand, where Y is a pyrimidine, and R is a purine, with its reverse complement YGR on the reverse strand). The counting rules were uniformly applied across both orientations as shown in Table 1. To determine whether the observed directional frequencies deviated from baseline stochastic expectations, the statistical significance was calculated using an empirical null model. This baseline (null model) was generated via random permutations of the observed mutational data, shuffling sample identifiers across the fixed genomic coordinates of all verified somatic mutations within the cohorts (see Section 3 of Results).

**Figure 4:**
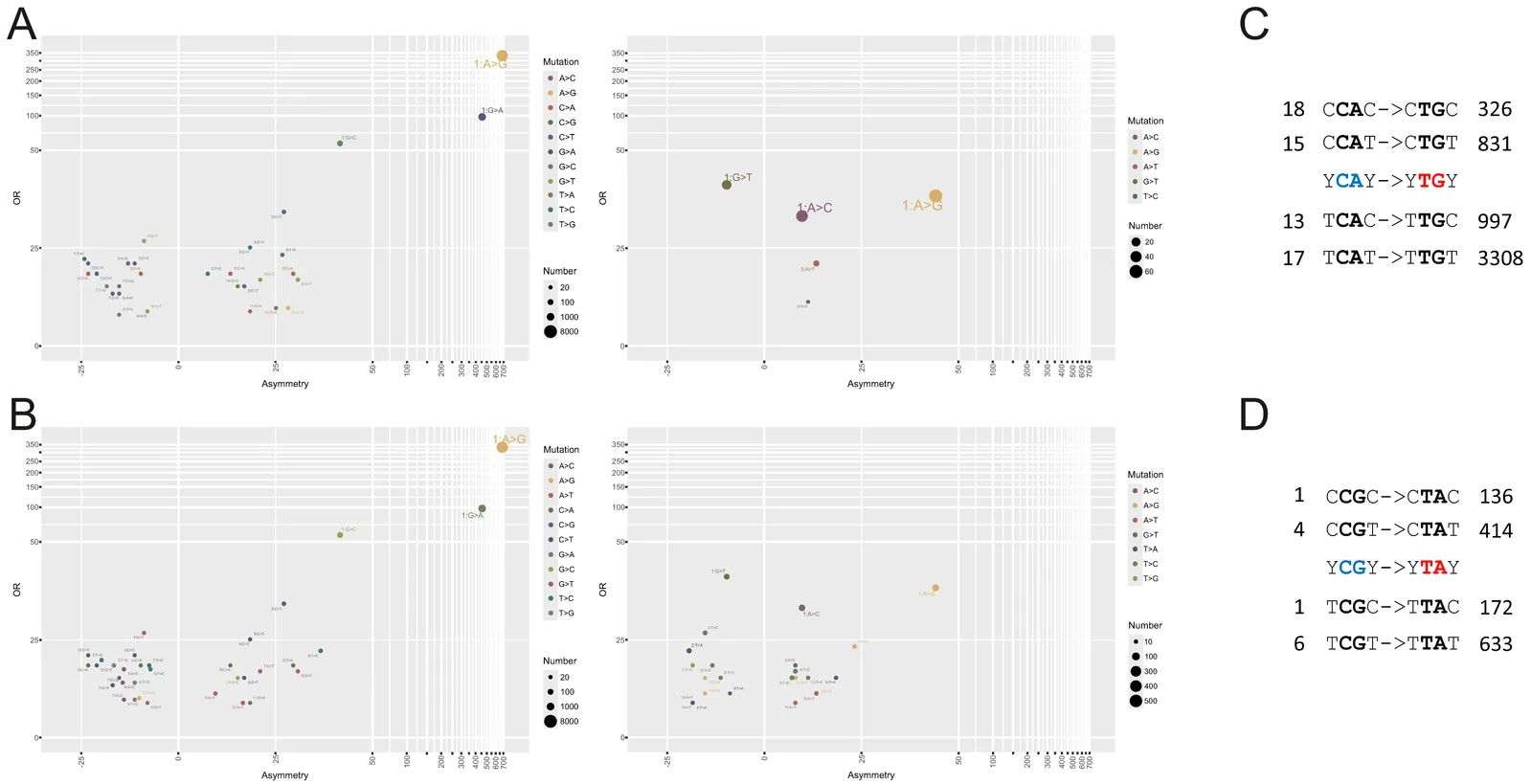
Directional asymmetry of non-UV mutations flanking UV-signature dinucleotides in BCC and melanoma. (See Figure S4 for directional bias of “other” types of base-substitution pairs flanking UV-induced YY-dimers in skin cancers). Asymmetry in the number of different types of non-UV mutations in paired mutation combinations: 5′nonUV-UV3′ and 5′UV-nonUV3′, where UV are mutations YCR>YTR (and their reverse complements YGR>YAR), and non-UV are mutations: (**A**) YRY>YNY (and their reverse complements RYR>RNR) or (**B**) YDY>YNY (and their reverse complements RHR>RNR). (**A**,**B**). The distance between paired mutations is given from the mutated YY-dimer (and its reverse complement RR-dimer). Left plots—BCC, right plots—melanoma. The Y axis shows the statistical significance (*p* < 0.05) of excess of observed mutations over expected; the X axis shows the directional bias value, the excess of the observed number of non-UV mutations in the 5′ vicinity of the YY-dimer relative to the 3′ vicinity (negative values) or the excess of the observed number of non-UV mutations in the 3′ vicinity of the YY-dimer relative to the 5′ vicinity (positive values). (**C**,**D**). DBS contexts that are significantly over-represented in the BCC cohort (observed counts of contexts are shown to the right of the mutating contexts) compared to the melanoma cohort (observed counts of contexts are shown to the left of the mutating contexts). (**C**), 5′UV-nonUV3′ contexts for CA>TG DBSs; (**D**), 5′UV-nonUV3′ contexts for CG>TA DBSs.

Figure 4 illustrates the genome-wide relationship between the spatial positioning of non-UV point mutations and their statistical enrichment relative to adjacent pyrimidine–pyrimidine (YY) dinucleotide cores. Panels A and B in Figure 4 present a symmetrically balanced comparative layout: the left-hand column tracks the basal cell carcinoma (BCC) dynamics, while the right-hand column tracks cutaneous melanoma. The X axes quantitatively map the directional bias value: in a specific SBS type (e.g., A>G) for a particular distance to the pyrimidine–pyrimidine (YY) dinucleotide center (e.g., 1 unit or the neighboring nucleotide closest to YY), we divide the higher count of non-UV substitutions by the lower; if the higher count of substitutions is at the 3′ proximity of YY-dimer, the directional bias value is considered positive; otherwise, if the larger number of non-UV substitutions is observed in 5′ proximity of YY-dimer, the directional bias value is considered negative. The *Y* axis indicates the statistical enrichment of observed over expected non-UV substitution counts that are adjacent to the targeted YY-dimer. Panels C and D in Figure 4 illustrate specific DBS contexts, which include both non-UV and UV SBS types, showing significant context-specific overrepresentation.

Our spatial analysis reveals a striking strand-directed co-localization pattern in BCC, where non-UV point mutations preferentially accumulate on the 3′ side (upstream) of UV-induced photoproducts. This directional asymmetry is highly pronounced for A>G and G>A transitions, as well as G>C transversions. These classes demonstrate extreme directional skewing, yielding 3′/5′ positional ratios between 300–600 and odds ratios (ORs) spanning 30–320. Conversely, melanoma samples exhibit less spatial asymmetry. The vast majority of mutational classes in melanoma cluster tightly near the origin of the distribution (X axis within ±25, OR < 25), with the isolated exception of a moderately 3′-biased A>G transition occurring within CA>TG double-nucleotide contexts.

Di-nucleotide alterations (or DBSs) show extensive structural divergence between the two skin cancers. In BCC, specific DBSs undergo dramatic enrichment when localized directly to the 3′ side of a canonical UV-signature dinucleotide: (1) CA>TG transitions exhibit a more than 20-fold enrichment relative to the baseline genomic expectation; (2) CG>TA transitions demonstrate up to a ~300-fold over-representation. Given that the impacts of different variant calling methodologies (for samples of melanoma and BCC) are usually minor (a significant overlap exists between GATK and qSNP (see Section 4)), the frequent occurrence of CG>TA DBS in BCC may suggest a potentially cooperative molecular mechanism.

Expanding the spatial mapping framework to the “Other” mutation pair category unmasked a striking directional polarity flanking the primary lesion sites (Figure S4). Both BCC and melanoma cohorts demonstrated highly congruent strand-specific asymmetric distributions across several major substitution classes: C>T transitions show the strongest 3′ bias in both cancers (BCC asymmetry ≈ 22, OR ≈ 2000; melanoma asymmetry ≈ 15, OR ≈ 800); T>A and T>C classes show a pronounced 3′ bias; G>A transitions show a pronounced 5′ (downstream) bias. While these fundamental directional polarities are broadly conserved between the two malignancies, their fine-grained molecular configurations diverge significantly: (1) the specific localized mutational contexts that undergo hyper-enrichment (defined by an odds ratio (OR) > 150) differ markedly between the BCC and melanoma cohorts; (2) BCC samples exhibit an approximate three-fold expansion in the absolute number of available 5′-biased mutation types compared to melanoma, matching the three-fold higher overall TMB observed in BCC relative to melanoma.

Thus, the divergent mutational signature spectrum identified between BCC and melanoma implies that distinct tissue-specific secondary mutagenic mechanisms intersect with raw UV-damage processing, leaving unique topographical scars on their respective cancer genomes. The clear reversal of directional polarity between C>T transitions (3′-biased) and G>A transitions (5′-biased) indicates that collateral mutations are deposited with strong strand-directed coordination, likely reflecting differences in how leading and lagging strands process bulky photoproducts or the strand-specific kinetics of transcription-coupled nucleotide excision repair (TC-NER) [22].

### 2.5. The Spatial Coupling Between UV Trinucleotides and the Nearest YY/RR Dinucleotides Is Different from That of Non-UV Trinucleotides

Building on our observation of directional asymmetry in collateral mutagenesis near UV-mutated pyrimidine–pyrimidine (YY) cores—a phenomenon likely orchestrated by error-prone translesion DNA synthesis—we sought to determine whether this spatial constraint generalizes to the global distribution of all single-base substitutions relative to their nearest YY or purine–purine (RR) dinucleotide. To evaluate this hypothesis, we developed a context-aware genome randomization model that controls for local nucleotide context, sequence mappability, and cohort-specific mutational burdens (see Materials and Methods). Next, we measured the physical distances between SBS (in trinucleotide context) and the closest YY or RR in the observed data and our context-aware genome randomization model. In the final step, we determined the absolute difference between the observed and the expected distance between SBS and its nearest YY or RR, with each SBS-YY/RR pair weighted according to this.

**Figure 5:**
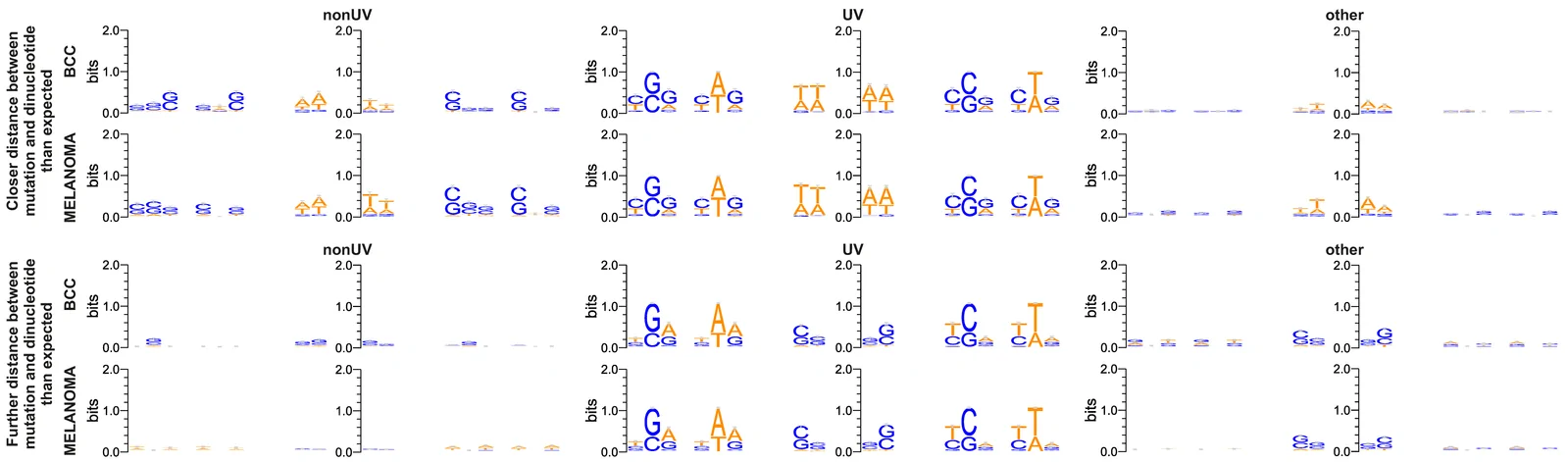
Spatial coupling between mutated trinucleotides and the nearest YY/RR dinucleotide. In each plot, the starting (original) trinucleotide pattern is shown on the left, with the final (mutated) trinucleotide pattern on the right; the position of the YY (or RR) dinucleotide relative to the mutated trinucleotide can be 5′ (when the dinucleotide is to the *left* of the trinucleotide) or 3′ (when the dinucleotide is to the *right* of the trinucleotide). Columns of plot pairs: Left plot pairs—combinations of non-UV-mutated trinucleotide and a YY (or RR) dinucleotide (non-UV—any Y mutations in the RYR pattern, any R mutations in the YRY pattern). Central plot pairs—combinations of UV-mutated trinucleotide and a YY (or RR) dinucleotide (UV—YCR>YTR mutations or RGY>RAY mutations). Right plot pairs—combinations of other mutations in trinucleotide patterns, not described by the two patterns above, with YY (or RR) dinucleotides.

Figure 5 maps the statistical deviations of these somatic variations from the randomized baseline: “closer distance” denotes mutations situated statistically nearer to the target YY-dinucleotide than expected by chance, indicating spatial attraction; “further distance” identifies mutations that are significantly displaced away from the target YY-dinucleotide, indicating spatial repulsion.

The following findings were demonstrated:

1. Attraction to AA/TT dinucleotides: Both BCC and melanoma displayed a modest but statistically significant spatial attraction of SBSs toward adjacent AA/TT dinucleotides. This localized enrichment was consistently observed across both canonical UV-signature and non-UV-signature substitutions, appearing slightly more pronounced in the BCC cohort. Notably, UV-signature mutations exhibited the highest absolute affinity for these TT/AA templates.

2. Repulsion from CC/GG configurations: While non-UV mutations lacked a uniform directional trend, both UV-signature and “other” mutation types exhibited consistent statistically robust spatial repulsion from CC/GG dinucleotides.

3. Process-specific spatial selectivity: Substitutions unclassified by either UV or non-UV paradigms (“other” mutations) completely lacked systematic distance biases. This indicates that the observed spatial relationships are a specific hallmark of the two dominant mutational processes (UV-induced and non-UV-associated) and not a generic feature of all substitutions.

4. Opposing strand-polarized footprints: We identified a highly coordinated direction-specific coupling between the somatic variant and its proximal dinucleotide core. UV-driven mutations displayed a strong attraction to TT dinucleotides situated strictly on their 3′ side. Conversely, non-UV mutations exhibited a completely inverted polarity, showing a preferential attraction to TT dinucleotides located on their 5′ side.

The pronounced attraction of UV-signature mutations to 3′ TT dinucleotides aligns closely with the established stereochemical kinetics of cyclobutane pyrimidine dimer (CPD) formation, which occurs preferentially at thymine–thymine tracks [4]. Conversely, the mirrored 5′-polarized attraction observed for non-UV mutations indicates that these flanking alterations are governed by separate biochemical mechanisms. This opposite polarity likely reflects either the strand-biased error profiles of translesion polymerases during post-replication gap filling or localized template-strand preferences of endogenous deaminases acting on transiently single-stranded DNA [4,5].

## 3. Discussion

*Distinct mutational coupling in BCC and melanoma*

We gathered precomputed VCF data for melanoma and basal cell carcinoma from various resources. If the effects of different variant calling pipelines for melanoma and BCC are minimal (a significant overlap exists between GATK and qSNP (see Section 4)), our data support a cancer-type-specific model of mutational coupling. In BCC, at least a fivefold higher count of collateral mutations was observed in comparison to melanoma. Therefore, in BCC, primary UV-induced photoproducts can serve as spatial “anchors” that actively stimulate downstream single-base and double-base collateral alterations. This rigid directional bias is absent in melanoma, indicating fundamental differences in how raw DNA lesions are processed between these cell types. This divergence is likely driven by tissue-specific variations in DNA repair machinery and polymerase engagement—such as the strict directionality of transcription-coupled or global-genome nucleotide excision repair (NER) working in concert with low-fidelity translesion synthesis (TLS) polymerases. Because the excision of UV photoproducts by the NER machinery features asymmetric dual incisions around the lesion, tissue-specific variations in the efficiency or polarity of gap-filling synthesis could explain the absolute lack of this spatial footprint in melanoma [23,24].

*Collateral versus independent mutagenesis. The biological significance of the observed enrichment of SBS1 and SBS5 among collateral mutations.*

The analysis of mutation clustering in BCC is a strong support for a model, where a portion of closely spaced mutations are inherited from a single mutational episode rather than independent events. The enrichment of SBS1, SBS2, SBS5, SBS19, SBS47, and SBS90 in collateral events indicates that a lesion caused by UV can lead to additional non-UV mutations within its local genomic neighborhood. Conversely, SBS7a–c and SBS58 dominate the independent mutational events class, because they are closely related to canonical UV lesion signatures. The distance dependence is biochemically plausible: immediately adjacent lesions arise from a shared local repair environment, whereas at larger separations, the collateral signal gradually reveals slower clock-like processes such as SBS5 [5,25,26].

SBS1 and SBS5 mutational signatures are clock-like signatures associated with age-related endogenous processes, rather than UV exposure itself. The signatures typically enriched in UV-exposed cells are SBS7a/SBS7b and DBS1. SBS1 is linked to the spontaneous deamination of 5-methylcytosine (5mC), whereas the precise origin and mechanisms of SBS5 remain unknown. SBS5 is widespread across tissues and cancers and also occurs in non-dividing cells.

The appearance of the SBS1 signature in UV-induced collateral mutations may suggest the presence of 5mC residues in cellular DNA. Indeed, UV light can induce pyrimidine dimers at CpG sites containing 5mC; furthermore, several studies show that these sites can be preferred targets for CPD formation under natural sunlight or UVB exposure. The effect is sequence- and wavelength-dependent, with some evidence that methylated CpG contexts are especially prone to UV-induced dimerization [27]. We suggest that unrepaired CPDs may accumulate at methylated CpG sites in silenced areas of the genome. Deamination of 5mC to thymine and subsequent aberrant DNA repair (see Discussion below) result in the observed double-nucleotide substitution.

**Figure 6:**
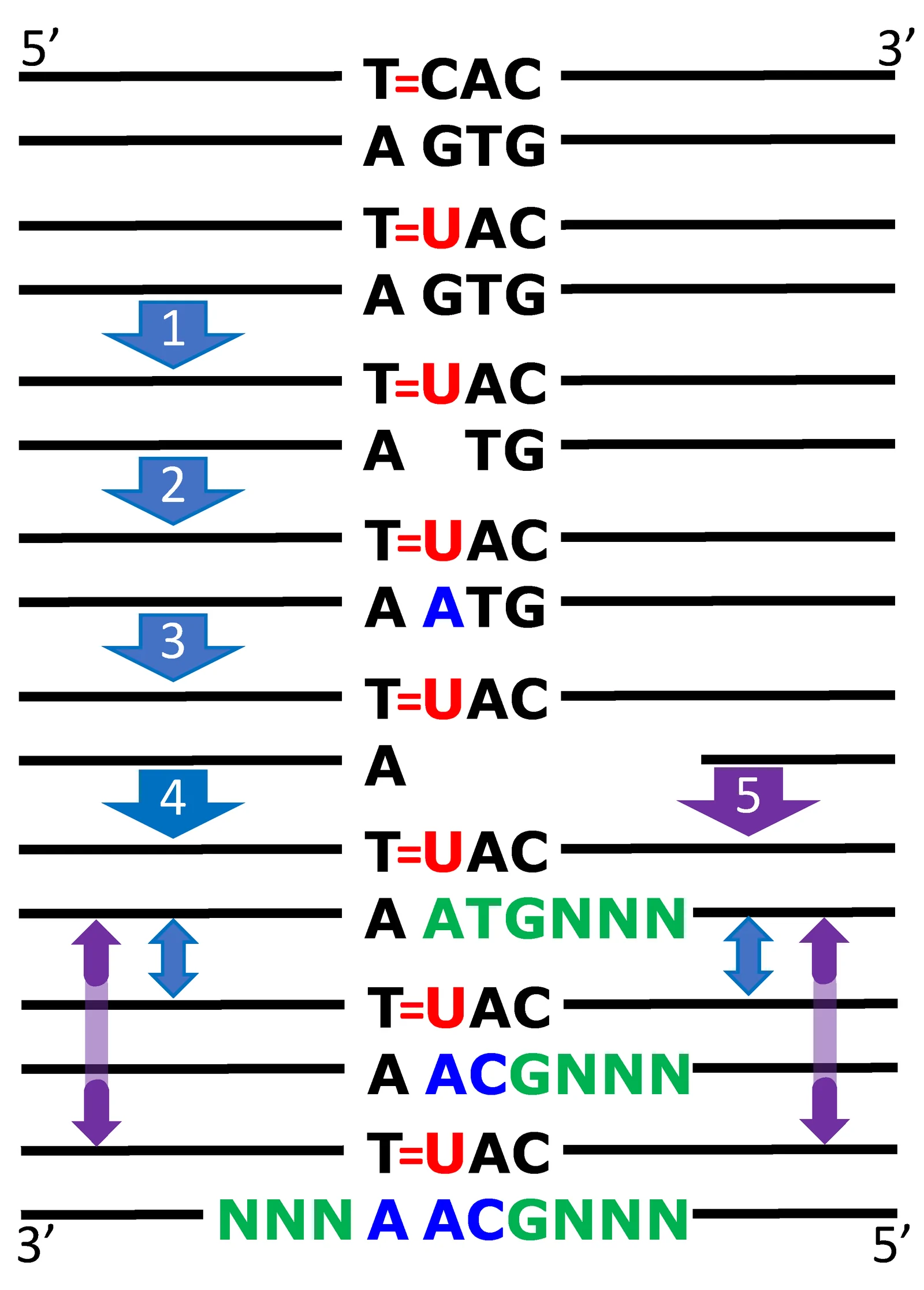
Proposed mechanistic model for double-nucleotide substitution (DNS) induction via aberrant UV-dimer repair in human cells. (1) Initiation of damage: The pathway is initiated by the aberrant excision of a regular base (G) opposite to the lesion by a DNA glycosylase or through the stochastic generation of a single-strand break (SSB) by the DNA trapped Topoisomerase I (TOP1). (2) Misincorporation: Pol η inserts an A opposite a deaminated C, stabilizing an intermediate containing SSB. (3) Exonucleolytic extension: The XRCC1/DNA ligase III complex or ligase I attempts to ligate the nick without success due to the steric hindrance imposed by the neighboring T=C (T=U) dimer. Subsequently, a 3′>5′ exonuclease—potentially APE1/APE2 or Pol δ in complex with PCNA—excises the mismatched A, expanding the initial break into a defined single-strand gap. (4) Canonical DNA polymerase switching and ligation: Replication protein-A (RPA) binds to ssDNA gap; then, RPA-coated ssDNA recruits Rad18/Rad6 ubiquitin ligase complex to monoubiquitinate PCNA (Ub-PCNA). Ub-PCNA still provides a processivity platform for Pol δ but enables switching to TLS polymerases upon stalling. Pol δ fills the single-stranded region up to the upstream boundary of the UV lesion. Upon encountering the rigid dimer, Pol δ stalls, causing a polymerase switch that recruits Pol η. In the primary scenario, Pol η inserts a CA dinucleotide opposite the damaged AU=T template strand; the resulting flanking mismatches create a substrate that could be successfully sealed by XRCC1/DNA ligase IIIα complex or ligase I. (5) Alternative strand-displacement synthesis: Otherwise, following PCNA ubiquitination and Pol η engagement, the polymerase inserts a longer CAA tract opposite the lesion. If the resulting junction configuration prevents immediate ligation by XRCC1/ligase III, Pol δ takes over downstream elongation from Pol η, continuing synthesis while displacing the downstream parental strand to resolve the blocked architecture. After that FEN1/PCNA cleaves the displaced 5′-strand generated by Pol δ, and only then, SSB is ligated by XRCC1/DNA ligase IIIα complex or ligase I.

While the SBS5 signature in UV-induced collateral mutations downstream of the lesion site might suggest age-dependent mutagenesis driven by unrepaired pyrimidine dimers in non-proliferating keratinocytes, we hypothesized that the accumulation of unrepaired UV lesions in these cells could trigger aberrant repair of the undamaged complementary strand (see Figure 6 and Section 3). This type of futile aberrant repair was described previously [28,29]. Because the lesion is not removed and persists, this process can lead to repeated futile cycles of excision and resynthesis, generating persistent DNA breaks. The genotoxic stress caused by these persistent breaks induces oxidative stress, which in turn promotes further mutations, thus forming the SBS5 signature associated with UV damage.

*Mechanistic underpinnings of collateral mutagenesis*

Exogenous lesions, such as cisplatin adducts and UV damage, have been reported to result in collateral mutations, and TLS polymerases are the primary cause of the observed downstream mutagenic events [4]. Collateral mutagenesis has been proposed to be a ‘funnel’ that gets input from multiple damage processing routes. Furthermore, it has been suggested that mutations such as SBS5 could come from TLS during whole-genome replication and repair errors in other stages of the cell cycle [5]. Beyond TLS, non-canonical mismatch repair (MMR) has the potential to cause mutations on lesion-containing DNA and become mutagenic if strand discrimination is lost. In the event of alkylation damage, repeated MMR cycles can result in futile excision resynthesis that can cause cytotoxicity and double-strand breaks. In the case of UV damage, older experimental research suggests that compound lesions that arise after bypass can be recognized by MMR and cause repeated cycles of breakage and repair [30,31,32,33]. Thus, in theory, UV lesions can generate mutations not only through replicative bypass but also through error-prone repair processing, and MMR may amplify damage rather than resolve it depending on the lesion type and cellular context.

*Spatial and strand bias in collateral mutagenesis*

The non-UV mutations near dipyrimidine (YY) dinucleotides in BCC are not symmetric around the damaged site, which is reflected by a striking 3′ bias. Mechanistically, the 3′ side of a dipyrimidine can be a good substrate for error-prone bypass, local deamination, or repair tract termination, particularly if a UV lesion alters polymerase behaviors or stalls excision intermediates. Melanoma’s asymmetry is much weaker, which may indicate a more efficient lesion processing or a different balance of repair and bypass, which would decrease the strand coordinated fixation of nearby non-UV changes [15,27,34].

*Collateral mutagenesis dinucleotide context*

The very large overrepresentation of CA>TG and CG>TA DNPs in BCC argues for a complex closely interconnected step-by-step mechanism, not merely an accumulation of independent point mutations [27]. When comparing these phenomena quantitatively between melanoma and BCC, it is important to note that discrepancies could occur due to differences in variant calling pipelines applied to melanoma and BCC samples (see Methods).

However, here, we attempt to provide the schematic framework for the mechanistic explanation of the hyper-enrichment of 3′-biased DBSs (CA>TG and CG>TA) observed within BCC. In Figure 6, we depict a putative molecular mechanism of the generation of unusual DBS observed in BCC. Upon high doses of UV irradiation, pyrimidine dimers accumulate in cellular DNA due to saturation of the capacity of NER machinery. It has been shown that unrepaired UV dimers could undergo futile aberrant cycles of excision and resynthesis [35,36]. For example, Topoisomerase 1 can be irreversibly trapped nearby the UV dimer but on the complementary non-damaged DNA strand, resulting in the generation of a single-strand break (SSB) 3′ downstream of the lesion [37]. Otherwise, guanine residue opposite to a UV dimer containing either cytosine or uracil could be excised in an aberrant manner. In both scenarios, an SSB or single-nucleotide gap opposite UV dimer cannot be ligated or used by Pol β to fill the gap because of the lesion in the template strand. This persistent unligatable SSB would recruit multiple repair factors, first PARP1 and then XRCC1/DNA ligase III, APE1, PCNA, Pol β and APE2. Noteworthy, APE1- and APE2-catalyzed 3′>5′ exonuclease can extend the unligatable SSB into a single-stranded gap up to 18–26 nucleotides in length [38]. This gap structure leads to ATR activation and PCNA mono-ubiquitination (ub-PCNA). An attempt to fill the gap would lead to DNA polymerase switching from pol δ to pol η to bypass the lesion. Although, DNA pol η could insert one A opposite to U in T=U the dimer, the resulting SSB would not be ligated because of the strong helix destabilizing effect of the unrepaired pyrimidine dimer. We hypothesize that this persistent SSB would undergo futile cycles of repair and de novo synthesis, until pol η together with other TLS polymerases catalyze erroneous dinucleotide extension, which would enable DNA Pol δ to extend and catalyze strand displacement synthesis, resulting in the completion of the bypass of the pyrimidine dimer. This model provides a working hypothesis for the observed patterns of DBS in BCC, which might serve as a basis for directing investigations to determine the precise mechanism for BCC-specific DBS in UV lesions.

*The potential significance of the enrichment of CA>TG and CG>TA dinucleotide substitutions in BCC.*

What is the physiological relevance of the enrichment of CA>TG and CG>TA DBS in basal cell carcinoma? Here, based on our model (Figure 6), we propose that the appearance of DBS may suggest the accumulation of unrepaired persistent DNA damage in slow-dividing or non-dividing cells. We hypothesize that (i) differentiated keratinocytes accumulating a high load of DNA damage upon extended exposure to UV light can still maintain essential cellular activities and normal skin functioning, and (ii) the unrepaired UV adducts can be targets for DNA replication-independent error-prone aberrant DNA repair. This aberrant repair generates persistent DNA strand breaks, which in turn induce a strong cellular response. Depending on the damage threshold, this response would either lead to cell death or manage to resynthesize and ligate the DNA strand breaks via recruitment of TLS machinery and other repair factors, resulting in the introduction of collateral mutations. Thus, our findings may shed light on the origin of the clock-like SBS5 signature in aging cells.

Here, we propose that the appearance of DBS suggests the existence of a specific cellular response to DNA damage that depends on the differentiation status of the cell, tissue architecture that protects the organism from the appearance of metastatic cancer cells, and a specific DNA damage detection system that may induce aberrant repair to either eliminate damaged cells or introduce complex mutations in non-transcribed silenced regions of the genome.

*Local sequence preferences*

Both UV signature and non-UV mutations show a modest but significant attraction to AA/TT dinucleotides and repulsion from CC/GG dinucleotides. AA/TT tracts are flexible and can be prone to bending, base flipping, or altered polymerase geometry, potentially making them permissive sites for damage fixation or secondary misincorporation. In contrast, CC/GG rich contexts may support more stable stacking and less accessible lesion conformations. Noteworthy, GC-rich isochores are gene-dense, transcriptionally active, and less condensed, all of which correlate with repair and recombinational activity [39,40]. It is tempting to speculate that error-free recombinational repair via template switching mechanisms is facilitated by CC/GG-rich contexts. The stronger affinity of UV signature mutations for TT/AA on the 3′ side further underscores that UV lesions are sequence-specific at the stages of repair and bypass, because the flanking sequence influences cyclobutane pyrimidine dimer formation and the mutational outcome of the lesion [34].

*The physiological basis of the observed differences in mutation spectra between melanoma and BCC.*

UV light exposure produces different primary responses in basal epidermal cells versus melanocytes because these cells are epigenetically very different and have different roles, signaling environments, and damage-response programs. Basal keratinocytes or basal epidermal cells function in barrier maintenance, proliferation control, and damage-induced cell-cycle arrest [41], while melanocytes are specialized for pigment production and photoprotection [42].

Basal epidermal cell/keratinocyte responses to UV are more directly associated with p53-dependent DNA damage responses, cell-cycle arrest, and apoptosis upon DNA damage overload. UVB is especially mutagenic in these cells, because it directly creates CPDs and 6-4PP adducts that, if not repaired by the NER machinery, induce mutations. Melanocytes respond differently to UV by increasing melanogenesis and activating DNA repair, antioxidant, and survival pathways, largely through paracrine signals from neighboring keratinocytes. Melanocytes are not just passive pigment cells: they can have altered or sometimes impaired repair of UV photoproducts and oxidative lesions, and their response depends strongly on whether they are in monolayer culture or in the epidermal niche with keratinocytes. Basal cells, by contrast, are the major proliferative compartment; so, UV damage there is more directly converted into mutations if checkpoint or repair responses fail [43].

Nevertheless, UV irradiation induces the same DNA lesions in both cell types, and these lesions should be removed by the NER pathway or bypassed during DNA replication by the same TLS DNA polymerases. Therefore, we expected that the UV-induced mutational patterns would be very similar between melanoma and BCC. However, our results revealed a striking difference in independent and collateral mutations between these tumors. The higher mutation rate in BCC can be explained by the higher UV doses that keratinocytes receive compared to melanocytes. Upon the accumulation of UV damage, keratinocytes stop proliferating; depending on the DNA damage level, some undergo apoptosis, while others survive and continue functioning with unrepaired persistent UV adducts in their genomes. Here, we propose that this persistent DNA damage in keratinocytes serves as a target for aberrant DNA repair that may induce collateral mutations, such as DBS (Figure 6).

In contrast, melanocytes activate the NER pathway in response to UV damage and continue to proliferate to produce protective melanin. This may explain the reduced mutation rate in melanoma compared to BCC. We suggest that most unrepaired UV adducts are bypassed during DNA replication with the help of specialized translesion synthesis, which is more error-free and avoids the appearance of collateral mutations.

*The translational implications of the identified mutational topology.*

In the present work, we revealed a striking difference in UV-induced mutational topology between melanoma and BCC, which was somewhat unexpected. Melanoma exhibits a lower mutation load and classical patterns of UV mutations compared to BCC. To explain these observations, we hypothesize that the appearance of collateral mutations and clock-related mutational signatures may suggest that these are features of less aggressive and non-metastatic cancers. Therefore, the appearance of collateral mutations could be used as a prognostic biomarker to predict a positive therapeutic response to anti-cancer therapy. Our study suggests that the mutation rate and spectra generated by UV light exposure of skin cells can be used in personalized dermatological oncology. For example, the appearance of collateral mutations in keratinocytes might be used as a prognostic parameter for BCC. In contrast, bypassing threshold mutation levels in melanocytes might indicate a high risk of melanoma.

## 4. Materials and Methods

### 4.1. Datasets Sources

Our study involved analyzing two tumor sample cohorts (represented as VCF files with mutation detection on the GRCh37 genome) that belong to two skin cancers: basal cell carcinoma (BCC) cohort and melanoma cohort. The previously published melanoma samples from sporadic patients [44] referenced in the study (consensus VCF files with SNVs and INDELs) are available in a public repository from the [45] website. Previously published WGS FASTQ datasets of sporadic cutaneous SCC and BCC used in this study and provided by Hartwig Medical Foundation [46] under data request DR-108 are available under restricted access after approval of the data sharing committee (https://www.hartwigmedicalfoundation.nl/en/data/data-acces-request/ (accessed on 6 July 2026)). Previously published WGS FASTQ datasets of sporadic cutaneous BCC [47] used in this study are available under reasonable request to the corresponding author and after approval of the data sharing committee of Gustave Roussy Institute.

Making bam files for BCC samples: Sequencing reads were mapped using BWA-MEM (v0.7.12) software [48] to the GRCh37 human reference genome.

Making vcf files for BCC samples: The standard GATK best practice pipeline to process the samples and call somatic variants was used. PCR duplicates were removed, and the base quality score was recalibrated using GATK [49] (v4.0.10.1), MarkDuplicates, and modules. Somatic variants were called and filtered using GATK tools Mutect2, FilterMutectCalls, and FilterByOrientationBias. Filtration of somatic variants in tumor samples PASS-flagged variants with reads on both strands were used for the analysis. VCF files were filtered using the alignability map of the human genome from the UCSC browser (https://genome.ucsc.edu/cgi-bin/hgFileUi?db=hg19&g=wgEncodeMapability (accessed on 6 July 2026)) with the length of K-mer equal to 75 bp (wgEncodeCrgMapabilityAlign75mer; mutations overlapped regions with a score < 1 were filtered out) and UCSC Browser blacklisted regions (Duke and DAC).

It is important to note that an accurate quantitative comparison of variant calling results (VCF files) between melanoma and basal cell carcinoma (BCC) is impossible due to several differences in the variant calling pipelines used on melanoma [44] and BCC samples: somatic mutations in melanoma are called using qSNP [50], whereas BCC somatic mutations adhere to GATK standard procedures as outlined above. Nonetheless, the overlap between qSNP and GATK somatic mutation detections is considerable [50], and qSNP exhibits a much higher false positive rate in somatic variant identification compared to GATK [50].

### 4.2. Datasets Filtration

We filtered the dataset in three consecutive steps.

First, using SigProfilerMatrixGenerator (Version 1.2.31) [51], we examined the trinucleotide context of mutations in all samples and retained only those with a cosine similarity of ≥0.9 to the COSMIC v3.4 SBS7a signature and ≥0.75 to the SBS7b signature, thereby selecting tumor samples of ultraviolet (UV) origin.

Second, we applied SigProfilerAssignment (Version 0.1.9) [52] to the selected samples to obtain mutational signature counts from the resulting Assignment_Solution_Activities.txt files.

Third, to remove outlier tumor samples that deviated from the cohort, we clustered the mutational signature count matrices using the affinity propagation method as implemented in the apcluster R package (Version 1.4.11) [53].

### 4.3. BCC Bam Files Analysis for Data-Driven Separation of Collateral Mutational Events from Independent Ones

To distinguish collateral mutational events from independent ones in a data-driven manner, we processed the BCC bam files through four consecutive procedures:

(1) Read filtering. Using a custom pipeline based on bedops [54], sambamba [55], and the jvarkit tool [56] biostar322664, we stringently retained only those reads whose alleles were present in the corresponding VCF file (this procedure is shown as PC 1 in the PseudoCodes (I) section).

(2) Extraction of paired mutations. We implemented a procedure that employs samtools [57] mpileup with the --output-QNAME option to extract all mutation (all analyzed mutations were strictly required to pass all standard Mutect2 quality control filters) pairs located within an intra-mutational distance ≤ 100 bp (shown as PC 2 in the PseudoCodes (I) section). Additionally, mutations falling on the extreme ends of reads were excluded from the analysis. This step analyzes the BAM outputs from the previous procedure and generates a final table containing the tumor ID, chromosome, 5′ and 3′ positions of the SNPs, reference and alternate alleles, distance between mutations, total number of reads, number of reads containing both mutations (bi-mutated reads), and number of reads mutated at the 5′ or 3′ position only.

Generation of pseudo-VCF files. The resulting table was parsed to obtain separate lists of neighboring mutations, stratified by whether they occurred on a single read or on two different reads. These lists were then printed as pseudo-VCF files.

(3) Mutational signature assignment. The pseudo-VCF files were analyzed with SigProfilerAssignment (Version 0.1.9) to quantify the mutational signatures associated with each class of mutation pairs.

### 4.4. BCC Sample Analysis to Identify Intra-Mutational Distances Overrepresented Among Neighboring Mutations

To identify intra-mutational distances that occur more frequently than expected by chance among neighboring mutations, we first applied the two consecutive procedures (read filtering and extraction of paired mutations) described in steps (1) and (2) of Section 4.3 (Materials and Methods). These steps produced a table of mutation pairs with their distances. Subsequently, we performed three additional procedures:

(3) Separation of reads by mutation count, and addition of genomic context. Using a custom procedure (shown as PC 3 in the PseudoCodes (I) section), we separated reads containing multiple mutations from reads containing only a single mutation. We then enriched the data with the mutational context from the GRCh37 reference genome using the Tabix module [58].

(4) Jackknife resampling. We performed a 50% jackknife of the sample set (shown as PC 4 in the PseudoCodes (I) section) to evaluate the distribution of observed mutation numbers.

(5) Summarization and visualization of randomized mutation data. Finally, we summarized and visualized the randomized mutation data (shown as PC 3 and PC 5 in the PseudoCodes (II) section, with modifications) to compare the observed overrepresentations against null expectations.

### 4.5. Permutation of Mutations Across BCC and Melanoma Samples to Identify Overrepresented Intra-Mutational Distances Among Neighboring Mutations

To determine which intra-mutational distances occur more frequently than expected by chance, we performed a permutation-based analysis organized into four consecutive procedures:

(1) Generation of randomized mutation data. Using the Mersenne Twister pseudo-random number generator (PRNG) [59] and parallel computing, we randomized the mutation data across tumor samples to obtain an expected (null) distribution of neighboring mutations (shown as PC 1 in the PseudoCodes (II) section).

(2) Summarization of observed mutation data. We summarized the observed mutation data across all tumor samples to extract the actual neighboring mutations (shown as PC 2 in the PseudoCodes (II) section).

(3) Extraction of specified pairwise patterns. From both the randomized and observed data, we extracted and summarized the mutation pairs conforming to specified pairwise IUPAC-patterns [60] (shown as PC 3 and PC 4 in the PseudoCodes (II) section).

(4) Generation of visualization tables. Finally, we produced formatted tables for subsequent data visualization (shown as PC 5 in the PseudoCodes (II) section).

Additionally, our permutation-based workflow was benchmarked against existing frameworks SigFit [16], Helmsman [17], MutationalPatterns [18], and SigProfilerClusters [19]. We demonstrated the significant benefits of our workflow in comparison to these methods, especially to SigProfilerClusters.

### 4.6. Quantifying Nucleotide Frequencies in Mutated Trinucleotides

To quantify the nucleotide frequencies within mutated trinucleotides, we analyzed three categories of mutation pairs: 5′nonUV-UV3′, 5′UV-nonUV3′, and “other” pairs. For each category, we simply counted the number of occurrences of each nucleotide at every position of the mutated trinucleotides (5′ and 3′).

### 4.7. Quantifying Asymmetry of Non-UV Mutations Adjacent to YY-Dinucleotides Within UV Mutation Patterns

First, to explore the mechanistic foundation of the noted mutational asymmetry, we analyzed the influence of diverse genomic and epigenomic elements through SigProfilerTopography v. 1.0.102 [21] with the standard settings. We accomplished this for all mutations and paired mutations (with a genomic distance of ≤100 base pairs).

After that, to assess whether non-UV mutations occur asymmetrically around YY-dinucleotides (i.e., pyrimidine–pyrimidine dimers) in UV mutation patterns, we performed the following three consecutive procedures:

(1) Polarized counting for observed and randomized mutations. For both observed mutations and mutations randomized by shuffling samples within the set of genomic positions, we counted mutations in non-UV patterns that neighbor a YY-dimer in UV patterns, separately for the 5′ side and the 3′ side (shown as PC 1 and PC 2 in the PseudoCodes (III) section).

(2) Non-parametric comparison of real versus randomized polarized counts. We compared the observed polarized counts (5′ vs. 3′) to those obtained from 500 randomizations (shown as PC 3 in the PseudoCodes (III) section). Statistical significance was defined as *p* = n/N, where N = 500 (total number of randomizations), and n is the number of randomized 5′nonUV-UV3′ or 5′UV-nonUV3′ pairs in which the count of non-UV mutations was equal to or greater than the observed count. The effect size (modulus of the ratio, OR) was calculated as *OR* = (*n_observed_* − *median*(*n_randomized_*))/*IQR*(*n_randomized_*), where IQR is the interquartile range of the randomized counts, and the median is the median of the randomized counts.

(3) Visualization of statistically significant comparisons. We generated signed values to represent the direction of asymmetry (shown as PC 4 in the PseudoCodes (III) section): Negative values indicate a bias toward the 5′ side (i.e., n5′ > 0 and n3′ > 0, we plot the ratio n5′/n3′; if n5′ > 0 and n3′ = 0, we plot n5′); positive values indicate a bias toward the 3′ side (i.e., n5′ > 0 and n3′ > 0, we plot the ratio n3′/n5′; if n5′ = 0 and n3′ > 0, we plot n3′).

### 4.8. Analyzing Spatial Coupling Between Mutated Trinucleotides and the Nearest YY/RR Dinucleotides

To evaluate whether mutated trinucleotides are spatially coupled to nearby YY (pyrimidine–pyrimidine) or RR (purine–purine) dinucleotides, we performed four consecutive procedures:

(1) Generation of randomized mutation positions that preserve the genomic context. Using the Tabix module [58], bedtools [57], and the alignability map of the human genome (described in Section 4.1 of Materials and Methods), we developed a novel procedure that generates randomly placed mutation positions matched to the real (observed) mutation set, thereby implicitly preserving the local nucleotide context of the genome (shown as PC 1 in the PseudoCodes (IV) section).

(2) Calculation of minimal distances to YY/RR dinucleotides. For both the observed and the randomly generated mutation positions, we computed the minimal distance from each mutated position to the nearest YY or RR dinucleotide using bedtools [57] (shown as PC 2 in the PseudoCodes (IV) section).

(3) Computation of a generalized minimal distance. To obtain a robust summary measure of spatial proximity, we calculated a composite distance for each observed and expected dataset (composed of all combinations of SBSs in trinucleotide context and nearest YY or RR dinucleotides) as: (mean_arithmetic_ + mean_geometric_ + mean_harmonic_ + median + 10^−6^)/4 (shown as PC 3 in the PseudoCodes (IV) section). The small constant (10^−6^) prevents division by zero in downstream steps.

(4) Weighted generation of sequence logos. We generated WebLogo [61] plots to visualize the spatial coupling (shown as PC 4 and PC 5 in the PseudoCodes (IV) section). For each mutated-trinucleotide YY (or RR) dinucleotide pair, the weight assigned during logo generation was proportional to the absolute difference between the observed and expected relative distances: a larger absolute difference resulted in a larger weight for that pair; a difference approaching zero resulted in a weight approaching zero. This weighting scheme ensures that the WebLogo [61] highlights only those dinucleotide–trinucleotide pairs whose spatial coupling deviates meaningfully from random expectation.

## 5. Conclusions

We establish a rigorous dual-approach bioinformatics open-source framework to resolve collateral mutagenesis in whole-genome sequencing data. First, a hypothesis-driven distance analysis compares observed intra-mutational spacing against the expected random distribution, revealing a significant excess of tightly clustered events. Second, a data-driven single-read phasing strategy validates that these clusters derive from single mutational episodes rather than independent hits.

Applying this framework to basal cell carcinoma (BCC) and melanoma whole-genome datasets uncovers fundamentally distinct lesion-processing landscapes. Independent mutations faithfully reproduce canonical UV signatures (SBS7a–c, SBS58) in both tumor types. In contrast, collateral clusters are highly enriched for non-UV signatures, including age-associated SBS1 and SBS5, cytidine deaminase-linked SBS2, and polymerase-associated SBS19/47/90, indicating that a primary photoproduct seeds secondary mutagenesis within its local chromatin environment. Critically, collateral mutations exhibit a pronounced 3′ asymmetry around dipyrimidine sites, with double-nucleotide substitutions hyper-enriched immediately 3′ of the initiating lesion. According to this, we showed that BCC exhibits a notable increase in dinucleotide base substitutions surrounding UV-signature sites on the 3′ side, especially CA>TG and CG>TA changes.

Because the mutation classification relies solely on whole-genome mutational coordinates and phased read evidence, it can be applied generically across various cancer types to disentangle primary damage signatures from the repair-process mutational burden. This framework thus offers a powerful tool for interpreting large-scale cancer genome datasets, enabling the detection of repair-overrun phenotypes, the inference of operative repair pathways, and the identification of collateral mutagenesis as a source of clinically relevant mutational complexity.

## Figures and Tables

**Table 1:** Counting rules for non-UV.

Sequence Context	Strand Position of Non-UV Variant	Assigned Orientation
YCR—non-UV	Downstream (Right side of motif)	3′
non-UV—YCR	Upstream (Left side of motif)	5′
YGR—non-UV	Downstream (Right side of complement)	5′
non-UV—YGR	Upstream (Left side of complement)	3′

## Data Availability

Melanoma samples are available from the [https://platform.icgc-argo.org/] website. WGS FASTQ datasets of cutaneous SCC and BCC are provided by Hartwig Medical Foundation under data request DR-108 after approval of the data sharing committee (https://www.hartwigmedicalfoundation.nl/en/data/data-acces-request/ (accessed on 6 July 2026)). Additional WGS FASTQ datasets of cutaneous BCC are available under restricted access after approval of the data sharing committee of Gustave Roussy Institute. The codes and all Supplementary Materials to reproduce the results of the study are available from Figshare dataset https://doi.org/10.6084/m9.figshare.32906894 version 2 (main repository, accessed on 6 July 2026), as well as at GitHub https://github.com/genkvg/clusmut (accessed on 6 July 2026).

## Supplementary Materials

The following supporting information can be downloaded at https://www.mdpi.com/article/10.3390/life16081300/s1, Supplement_Pseudocodes contains pseudocode describing the logic of the custom Perl scripts used in the main analyses (codes were deposited on Figshare and on GitHub); Supplementary_file S1 provides results of additional computations using SigProfilerClusters (v1.2.3) for the BCC and melanoma cohorts; Supplementary_file S2 provides results of additional analyses using SigProfilerTopography (v1.0.102).

## Abbreviations

The following abbreviations are used in this manuscript:

BCC	Basal cell carcinoma
UV	Ultraviolet
NER	Nucleotide excision repair
TLS	Translesion synthesis
ICLs	Interstrand crosslinks
UNG	Uracil-DNA glycosylase
BER	Base excision repair
CPD	Cyclobutane pyrimidine dimers
TOP1	Trapping of Topoisomerase I
SBS	Single-base substitution
TMB	Tumor mutation burden
Mb	Megabase
DBSs	Double-base substitutions
TBSs	Triple-base substitutions
MMR	Mismatch repair
