# Supplementary material for: Two Faces of UV Mutagenesis: Independent and Collateral Mutagenesis in Skin Cancers
Source: Life (Basel). 2026 Aug 7;16(8):1300. doi: 10.3390/life16081300 (PMC13514280; doi:10.3390/life16081300)
Supplement: Supplementary file 1 [file life-16-01300-s001.zip › Supplementary_Materials.pdf]

# Supplementary Materials

# Two Faces of UV Mutagenesis: Independent and Collateral Mutagenesis in Skin Cancers

**Konstantin Gunbin [1,3], Zamart Ramazanova [2,3], Bakhyt Matkarimov [3,4,*], Murat Saparbaev [5,6], Sergey Nikolaev [7] and Andrey Yurchenko [7,*]**

[1] Immanuel Kant Baltic Federal University, Kaliningrad 236016, Russia
[2] Department of Electrical and Computer Engineering, School of Engineering and Digital Sciences, Nazarbayev University, Astana, 010000, Kazakhstan
[3] National Laboratory Astana, Nazarbayev University, Astana 010000, Kazakhstan
[4] L.N. Gumilyov Eurasian National University, Astana 010008, Kazakhstan
[5] Department of Molecular Biology and Genetics, Faculty of Biology and Biotechnology, Al-Farabi Kazakh National University, Almaty 050040, Kazakhstan
[6] Groupe «Mechanisms of DNA Repair and Carcinogenesis», CNRS UMR9019, Université Paris-Saclay, Gustave Roussy Cancer Campus, F-94805 Villejuif Cedex, France
[7] INSERM U981, Gustave Roussy Institute, Université Paris-Saclay, Villejuif, France

* Correspondence: bmatkarimov@nu.edu.kz, andrey.yurchenko@gustaveroussy.fr

## Supplementary Figures:

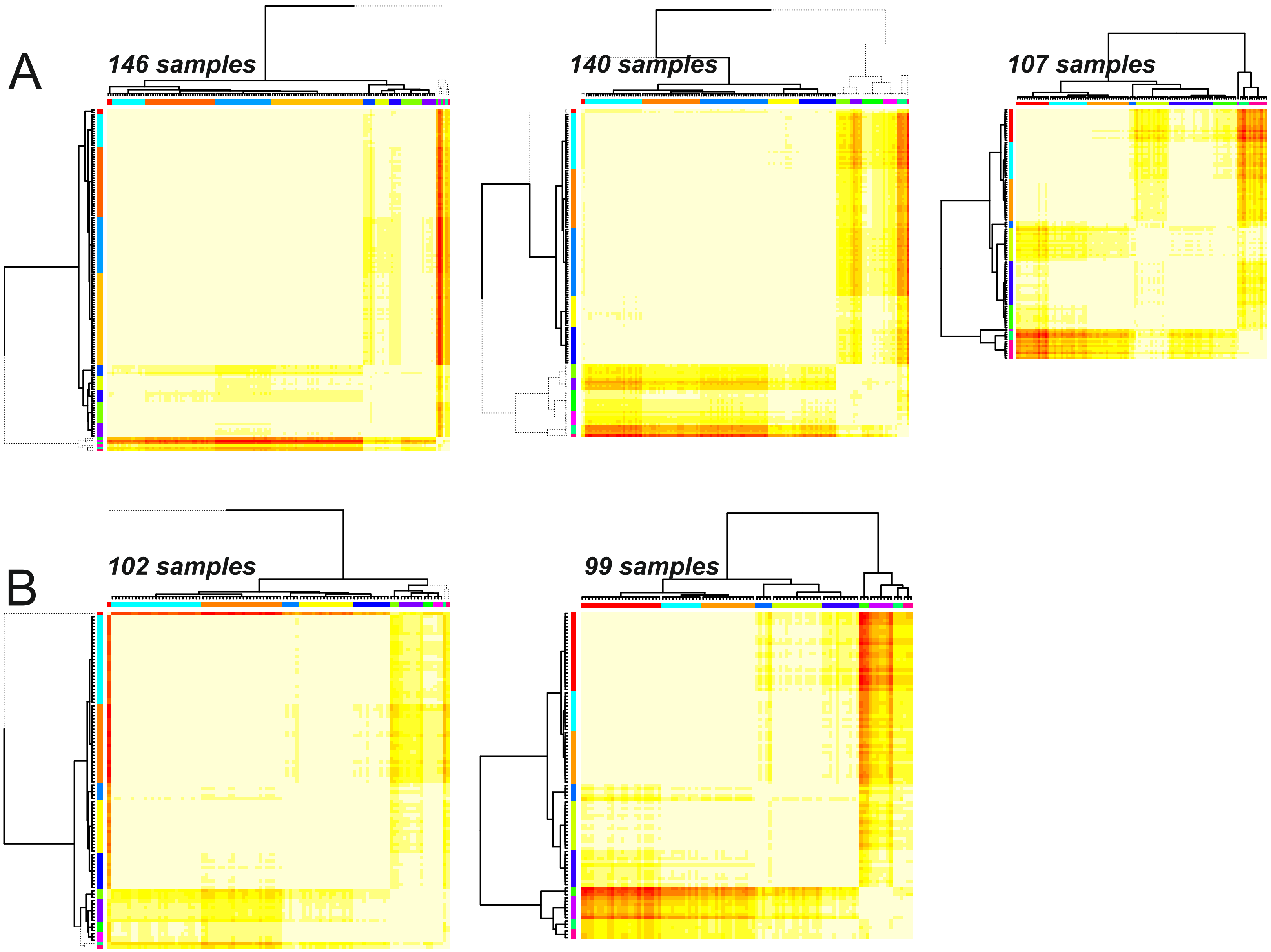
A
146 samples
140 samples
107 samples
B
102 samples
99 samples

**Figure S1.** Stepwise refining selection of melanoma and BCC samples.

Stepwise selection of melanoma and BCC samples using affinity propagation clustering of mutational signature counts. The selection was performed to remove atypical samples that deviate significantly from the cohort. The removed samples are indicated by dashed lines on the dendrograms. Removal of atypical samples was stopped when multifurcations of internal branches disappeared from the dendrogram of the main group.

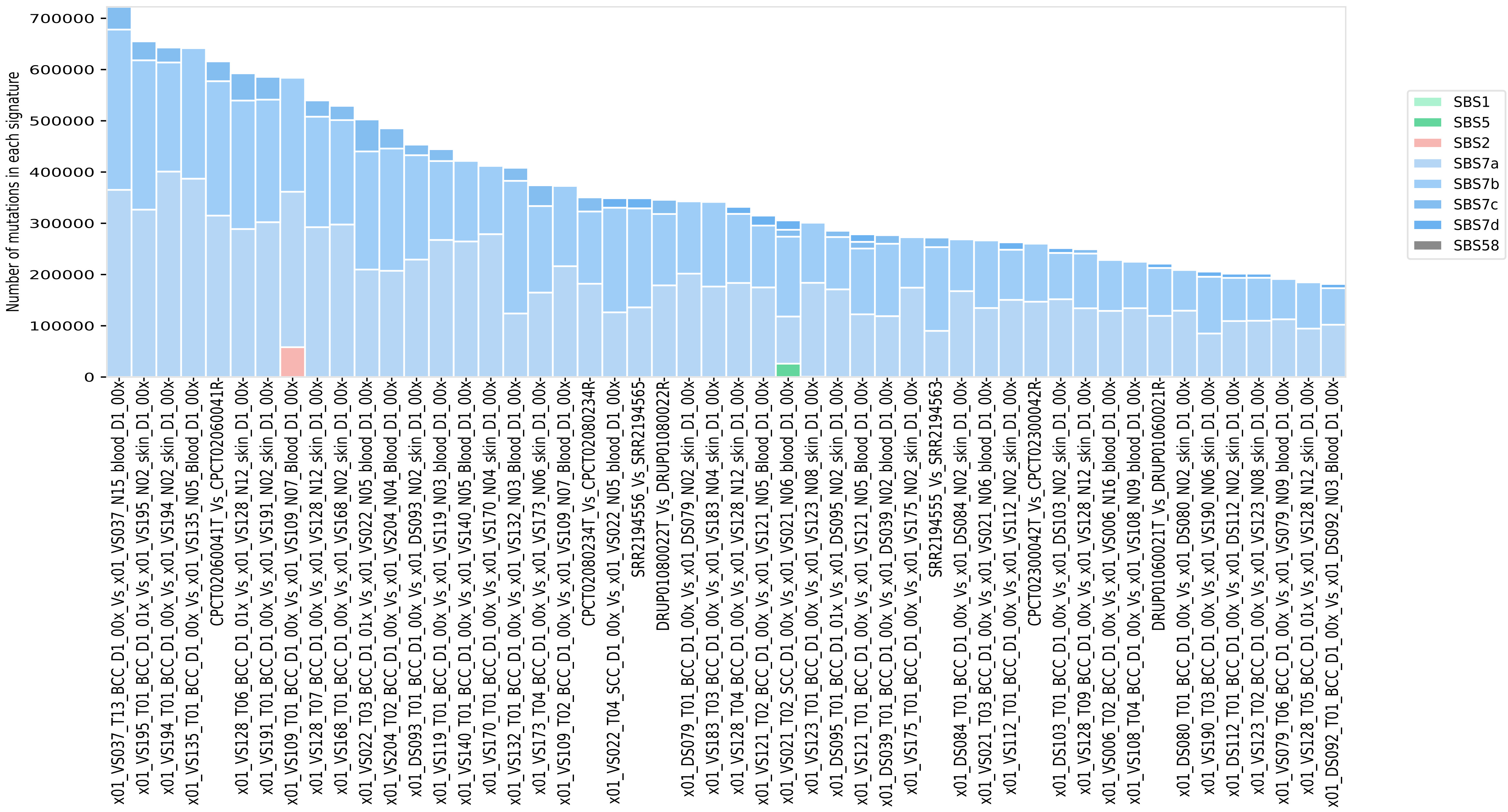
Number of mutations in each signature
0
100000
200000
300000
400000
500000
600000
700000
SBS1
SBS5
SBS2
SBS7a
SBS7b
SBS7c
SBS7d
SBS58
x01_VS037_T13_BCC_D1_00x_Vs_x01_VS037_N15_blood_D1_00x
x01_VS195_T01_BCC_D1_01x_Vs_x01_VS195_N02_skin_D1_00x
x01_VS194_T01_BCC_D1_00x_Vs_x01_VS194_N02_skin_D1_00x
x01_VS135_T01_BCC_D1_00x_Vs_x01_VS135_N05_Blood_D1_00x
CPCT02060041T_Vs_CPCT02060041R
x01_VS128_T06_BCC_D1_01x_Vs_x01_VS128_N12_skin_D1_00x
x01_VS191_T01_BCC_D1_00x_Vs_x01_VS191_N02_skin_D1_00x
x01_VS109_T01_BCC_D1_00x_Vs_x01_VS109_N07_Blood_D1_00x
x01_VS128_T07_BCC_D1_00x_Vs_x01_VS128_N12_skin_D1_00x
x01_VS168_T01_BCC_D1_00x_Vs_x01_VS168_N02_skin_D1_00x
x01_VS022_T03_BCC_D1_01x_Vs_x01_VS022_N05_blood_D1_00x
x01_VS204_T02_BCC_D1_00x_Vs_x01_VS204_N04_Blood_D1_00x
x01_DS093_T01_BCC_D1_00x_Vs_x01_DS093_N02_skin_D1_00x
x01_VS119_T01_BCC_D1_00x_Vs_x01_VS119_N03_blood_D1_00x
x01_VS140_T01_BCC_D1_00x_Vs_x01_VS140_N05_Blood_D1_00x
x01_VS170_T01_BCC_D1_00x_Vs_x01_VS170_N04_skin_D1_00x
x01_VS132_T01_BCC_D1_00x_Vs_x01_VS132_N03_Blood_D1_00x
x01_VS173_T04_BCC_D1_00x_Vs_x01_VS173_N06_skin_D1_00x
x01_VS109_T02_BCC_D1_00x_Vs_x01_VS109_N07_Blood_D1_00x
CPCT02080234T_Vs_CPCT02080234R
x01_VS022_T04_SCC_D1_00x_Vs_x01_VS022_N05_blood_D1_00x
SRR2194556_Vs_SRR2194565
DRUP01080022T_Vs_DRUP01080022R
x01_DS079_T01_BCC_D1_00x_Vs_x01_DS079_N02_skin_D1_00x
x01_VS183_T03_BCC_D1_00x_Vs_x01_VS183_N04_skin_D1_00x
x01_VS128_T04_BCC_D1_00x_Vs_x01_VS128_N12_skin_D1_00x
x01_VS121_T02_BCC_D1_00x_Vs_x01_VS121_N05_Blood_D1_00x
x01_VS021_T02_SCC_D1_00x_Vs_x01_VS021_N06_blood_D1_00x
x01_VS123_T01_BCC_D1_00x_Vs_x01_VS123_N08_skin_D1_00x
x01_DS095_T01_BCC_D1_01x_Vs_x01_DS095_N02_skin_D1_00x
x01_VS121_T01_BCC_D1_00x_Vs_x01_VS121_N05_Blood_D1_00x
x01_DS039_T01_BCC_D1_00x_Vs_x01_DS039_N02_blood_D1_00x
x01_VS175_T01_BCC_D1_00x_Vs_x01_VS175_N02_skin_D1_00x
SRR2194555_Vs_SRR2194563
x01_DS084_T01_BCC_D1_00x_Vs_x01_DS084_N02_skin_D1_00x
x01_VS021_T03_BCC_D1_00x_Vs_x01_VS021_N06_blood_D1_00x
x01_VS112_T01_BCC_D1_00x_Vs_x01_VS112_N02_skin_D1_00x
CPCT02300042T_Vs_CPCT02300042R
x01_DS103_T01_BCC_D1_00x_Vs_x01_DS103_N02_skin_D1_00x
x01_VS128_T09_BCC_D1_00x_Vs_x01_VS128_N12_skin_D1_00x
x01_VS006_T02_BCC_D1_00x_Vs_x01_VS006_N16_blood_D1_00x
x01_VS108_T04_BCC_D1_00x_Vs_x01_VS108_N09_blood_D1_00x
DRUP01060021T_Vs_DRUP01060021R
x01_DS080_T01_BCC_D1_00x_Vs_x01_DS080_N02_skin_D1_00x
x01_VS190_T03_BCC_D1_00x_Vs_x01_VS190_N06_skin_D1_00x
x01_DS112_T01_BCC_D1_00x_Vs_x01_DS112_N02_skin_D1_00x
x01_VS123_T02_BCC_D1_00x_Vs_x01_VS123_N08_skin_D1_00x
x01_VS079_T06_BCC_D1_00x_Vs_x01_VS079_N09_blood_D1_00x
x01_VS128_T05_BCC_D1_01x_Vs_x01_VS128_N12_skin_D1_00x
x01_DS092_T01_BCC_D1_00x_Vs_x01_DS092_N03_Blood_D1_00x

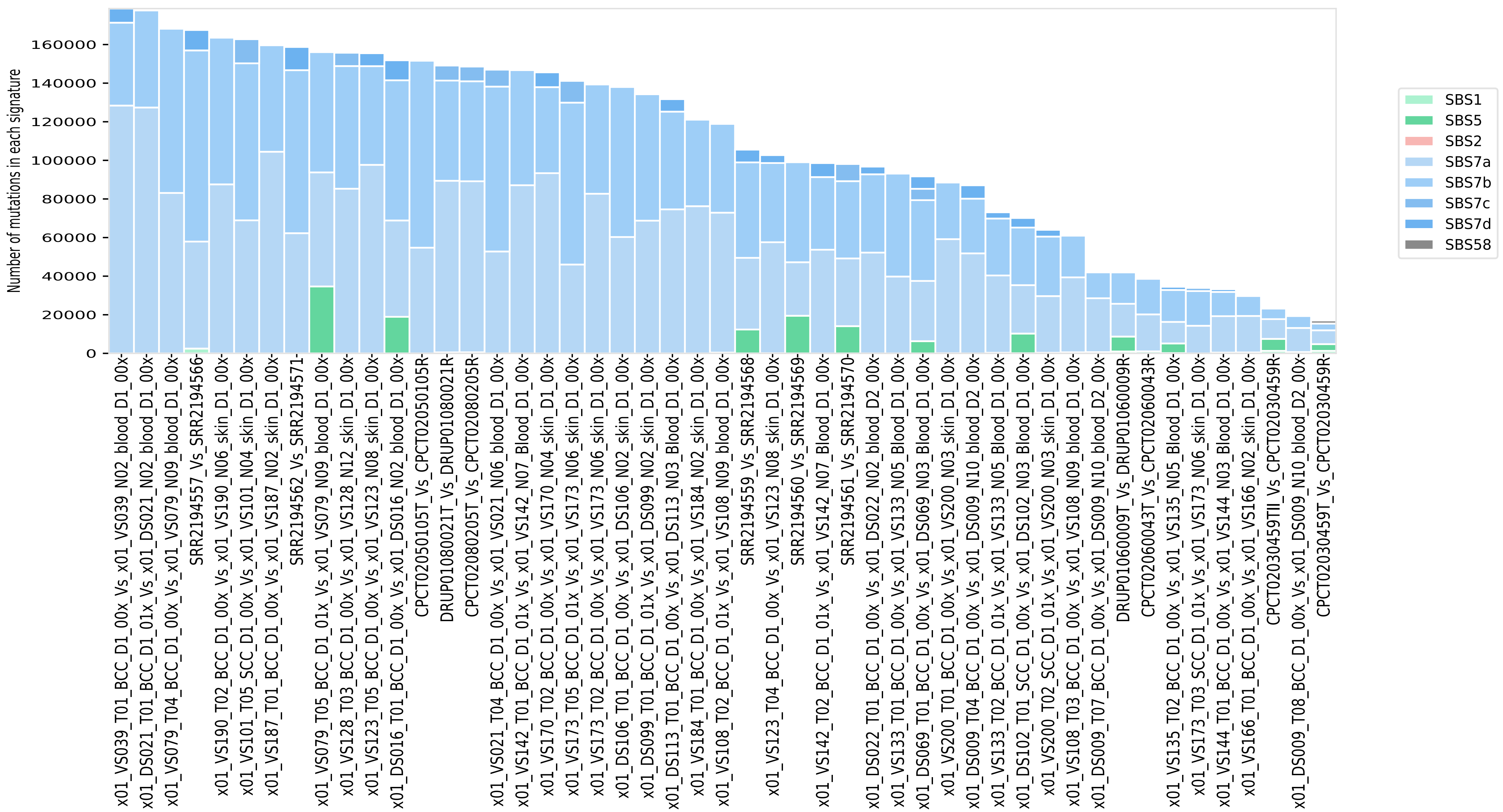
Number of mutations in each signature
160000
140000
120000
100000
80000
60000
40000
20000
0
x01_VS039_T01_BCC_D1_00x_Vs_x01_VS039_N02_blood_D1_00x
x01_DS021_T01_BCC_D1_01x_Vs_x01_DS021_N02_blood_D1_00x
x01_VS079_T04_BCC_D1_00x_Vs_x01_VS079_N09_blood_D1_00x
SRR2194557_Vs_SRR2194566
x01_VS190_T02_BCC_D1_00x_Vs_x01_VS190_N06_skin_D1_00x
x01_VS101_T05_SCC_D1_00x_Vs_x01_VS101_N04_skin_D1_00x
x01_VS187_T01_BCC_D1_00x_Vs_x01_VS187_N02_skin_D1_00x
SRR2194562_Vs_SRR2194571
x01_VS079_T05_BCC_D1_01x_Vs_x01_VS079_N09_blood_D1_00x
x01_VS128_T03_BCC_D1_00x_Vs_x01_VS128_N12_skin_D1_00x
x01_VS123_T05_BCC_D1_00x_Vs_x01_VS123_N08_skin_D1_00x
x01_DS016_T01_BCC_D1_00x_Vs_x01_DS016_N02_blood_D1_00x
CPCT02050105T_Vs_CPCT02050105R
DRUP01080021T_Vs_DRUP01080021R
CPCT02080205T_Vs_CPCT02080205R
x01_VS021_T04_BCC_D1_00x_Vs_x01_VS021_N06_blood_D1_00x
x01_VS142_T01_BCC_D1_01x_Vs_x01_VS142_N07_Blood_D1_00x
x01_VS170_T02_BCC_D1_00x_Vs_x01_VS170_N04_skin_D1_00x
x01_VS173_T05_BCC_D1_01x_Vs_x01_VS173_N06_skin_D1_00x
x01_VS173_T02_BCC_D1_00x_Vs_x01_VS173_N06_skin_D1_00x
x01_DS106_T01_BCC_D1_00x_Vs_x01_DS106_N02_skin_D1_00x
x01_DS099_T01_BCC_D1_01x_Vs_x01_DS099_N02_skin_D1_00x
x01_DS113_T01_BCC_D1_00x_Vs_x01_DS113_N03_Blood_D1_00x
x01_VS184_T01_BCC_D1_00x_Vs_x01_VS184_N02_skin_D1_00x
x01_VS108_T02_BCC_D1_01x_Vs_x01_VS108_N09_blood_D1_00x
SRR2194559_Vs_SRR2194568
x01_VS123_T04_BCC_D1_00x_Vs_x01_VS123_N08_skin_D1_00x
SRR2194560_Vs_SRR2194569
x01_VS142_T02_BCC_D1_01x_Vs_x01_VS142_N07_Blood_D1_00x
SRR2194561_Vs_SRR2194570
x01_DS022_T01_BCC_D1_00x_Vs_x01_DS022_N02_blood_D2_00x
x01_VS133_T01_BCC_D1_00x_Vs_x01_VS133_N05_Blood_D1_00x
x01_DS069_T01_BCC_D1_00x_Vs_x01_DS069_N03_Blood_D1_00x
x01_VS200_T01_BCC_D1_00x_Vs_x01_VS200_N03_skin_D1_00x
x01_DS009_T04_BCC_D1_00x_Vs_x01_DS009_N10_blood_D2_00x
x01_VS133_T02_BCC_D1_01x_Vs_x01_VS133_N05_Blood_D1_00x
x01_DS102_T01_SCC_D1_00x_Vs_x01_DS102_N03_Blood_D1_00x
x01_VS200_T02_SCC_D1_01x_Vs_x01_VS200_N03_skin_D1_00x
x01_VS108_T03_BCC_D1_00x_Vs_x01_VS108_N09_blood_D1_00x
x01_DS009_T07_BCC_D1_00x_Vs_x01_DS009_N10_blood_D2_00x
DRUP01060009T_Vs_DRUP01060009R
CPCT02060043T_Vs_CPCT02060043R
x01_VS135_T02_BCC_D1_00x_Vs_x01_VS135_N05_Blood_D1_00x
x01_VS173_T03_SCC_D1_01x_Vs_x01_VS173_N06_skin_D1_00x
x01_VS144_T01_BCC_D1_00x_Vs_x01_VS144_N03_Blood_D1_00x
x01_VS166_T01_BCC_D1_00x_Vs_x01_VS166_N02_skin_D1_00x
CPCT02030459TII_Vs_CPCT02030459R
x01_DS009_T08_BCC_D1_00x_Vs_x01_DS009_N10_blood_D2_00x
CPCT02030459T_Vs_CPCT02030459R
SBS1
SBS5
SBS2
SBS7a
SBS7b
SBS7c
SBS7d
SBS58

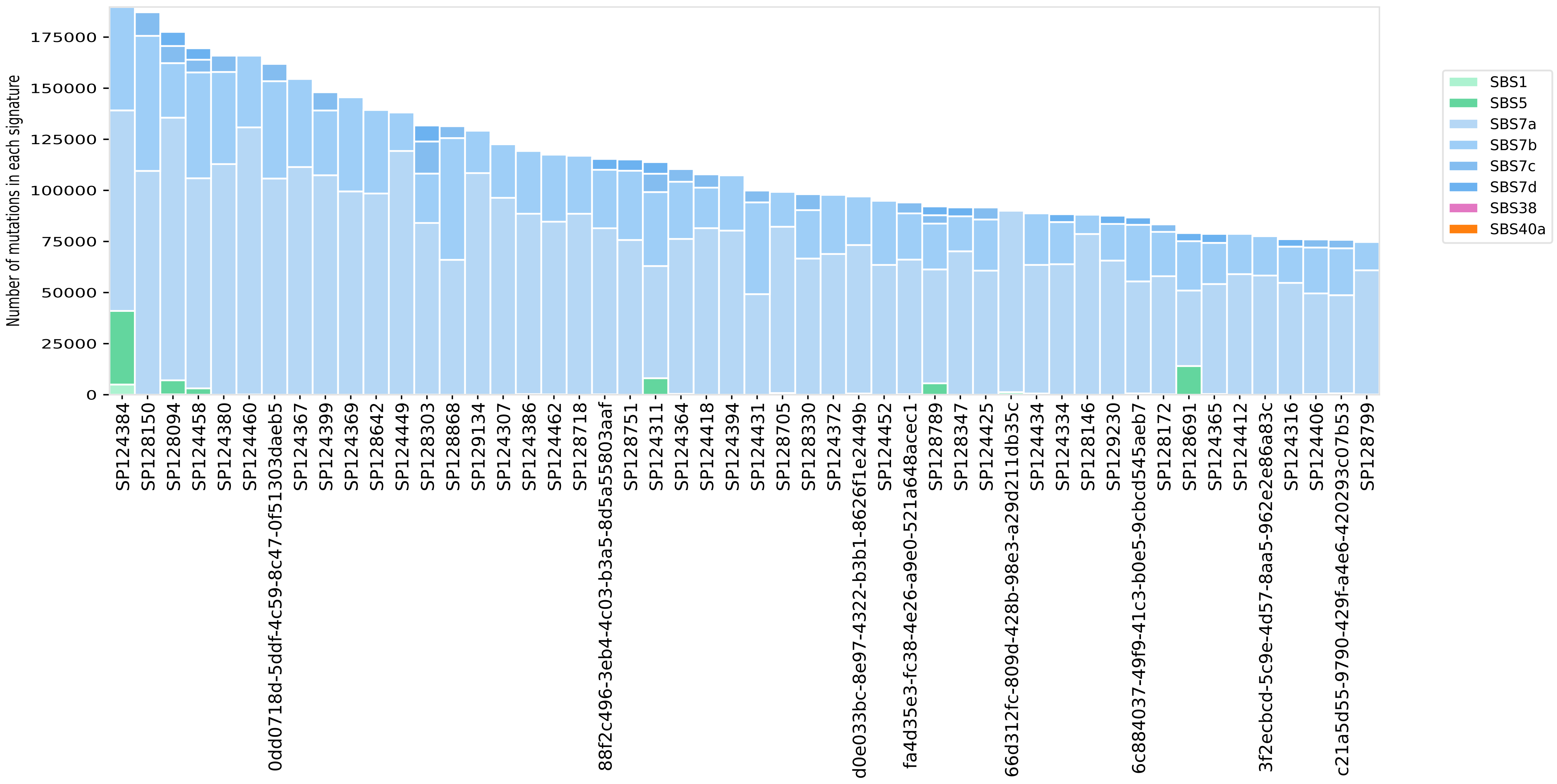

Number of mutations in each signature
0
25000
50000
75000
100000
125000
150000
175000
SBS1
SBS5
SBS7a
SBS7b
SBS7c
SBS7d
SBS38
SBS40a
SP124384
SP128150
SP128094
SP124458
SP124380
SP124460
0dd0718d-5ddf-4c59-8c47-0f51303daeb5
SP124367
SP124399
SP124369
SP128642
SP124449
SP128303
SP128868
SP129134
SP124307
SP124386
SP124462
SP128718
88f2c496-3eb4-4c03-b3a5-8d5a55803aaf
SP128751
SP124311
SP124364
SP124418
SP124394
SP124431
SP128705
SP128330
SP124372
d0e033bc-8e97-4322-b3b1-8626f1e2449b
SP124452
fa4d35e3-fc38-4e26-a9e0-521a648acec1
SP128789
SP128347
SP124425
66d312fc-809d-428b-98e3-a29d211db35c
SP124434
SP124334
SP128146
SP129230
6c884037-49f9-41c3-b0e5-9cbcd545aeb7
SP128172
SP128691
SP124365
SP124412
3f2ecbcd-5c9e-4d57-8aa5-962e2e86a83c
SP124316
SP124406
c21a5d55-9790-429f-a4e6-420293c07b53
SP128799

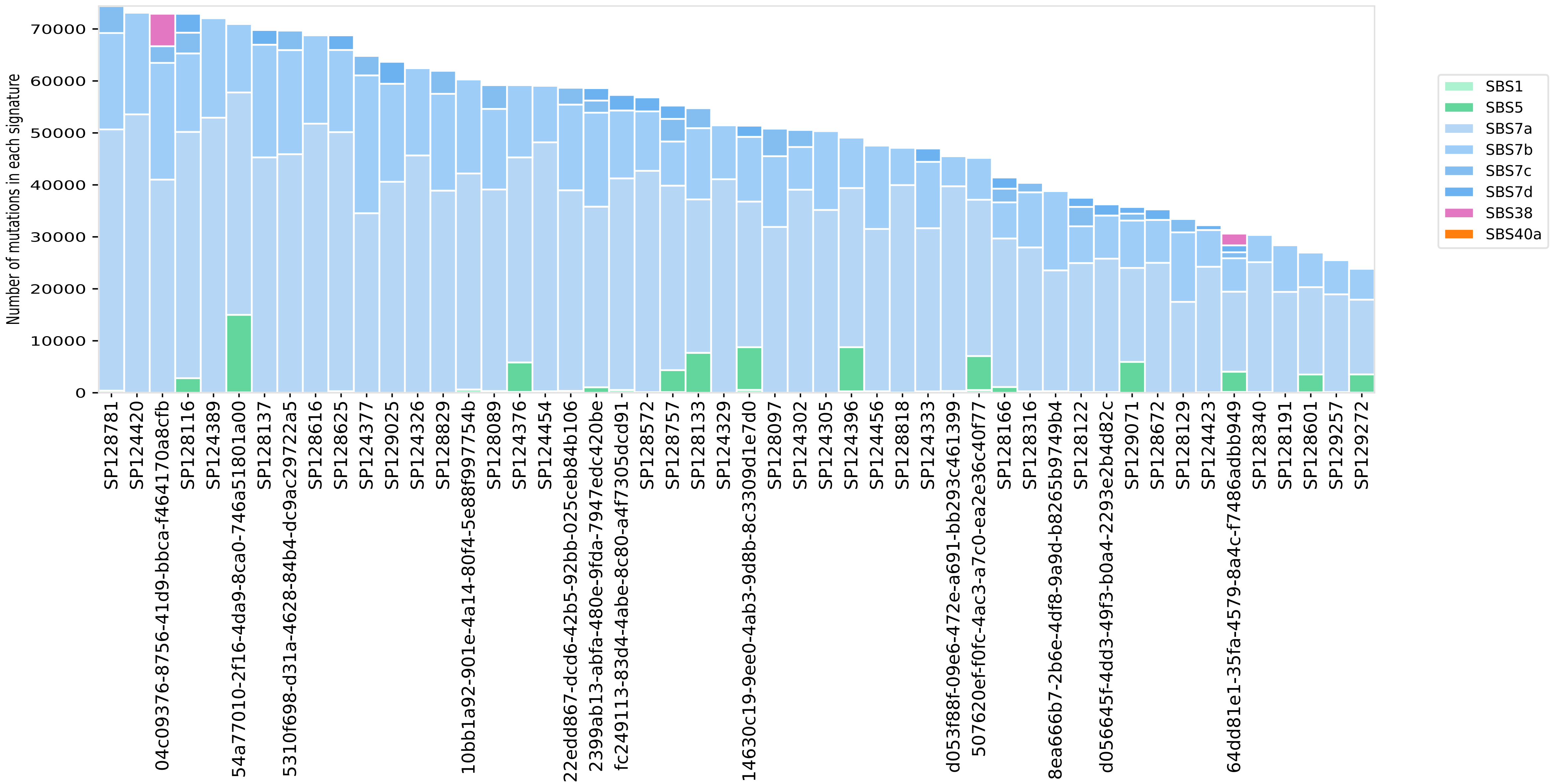
Number of mutations in each signature
0
10000
20000
30000
40000
50000
60000
70000
SBS1
SBS5
SBS7a
SBS7b
SBS7c
SBS7d
SBS38
SBS40a
SP128781
SP124420
04c09376-8756-41d9-bbca-f464170a8cfb
SP128116
SP124389
54a77010-2f16-4da9-8ca0-746a51801a00
SP128137
5310f698-d31a-4628-84b4-dc9ac29722a5
SP128616
SP128625
SP124377
SP129025
SP124326
SP128829
10bb1a92-901e-4a14-80f4-5e88f997754b
SP128089
SP124376
SP124454
22edd867-dcd6-42b5-92bb-025ceb84b106
2399ab13-abfa-480e-9fda-7947edc4420be
fc249113-83d4-4abe-8c80-a4f7305dcd91
SP128572
SP128757
SP128133
SP124329
14630c19-9ee0-4ab3-9d8b-8c3309d1e7d0
SP128097
SP124302
SP124305
SP124396
SP124456
SP128818
SP124333
d053f88f-09e6-472e-a691-bb293c461399
507620ef-f0fc-4ac3-a7c0-ea2e36c40f77
SP128166
SP128316
8ea666b7-2b6e-4df8-9a9d-b8265b9749b4
SP128122
d056645f-4dd3-49f3-b0a4-2293e2b4d82c
SP129071
SP128672
SP128129
SP124423
64dd81e1-35fa-4579-8a4c-f7486adbb949
SP128340
SP128191
SP128601
SP129257
SP129272

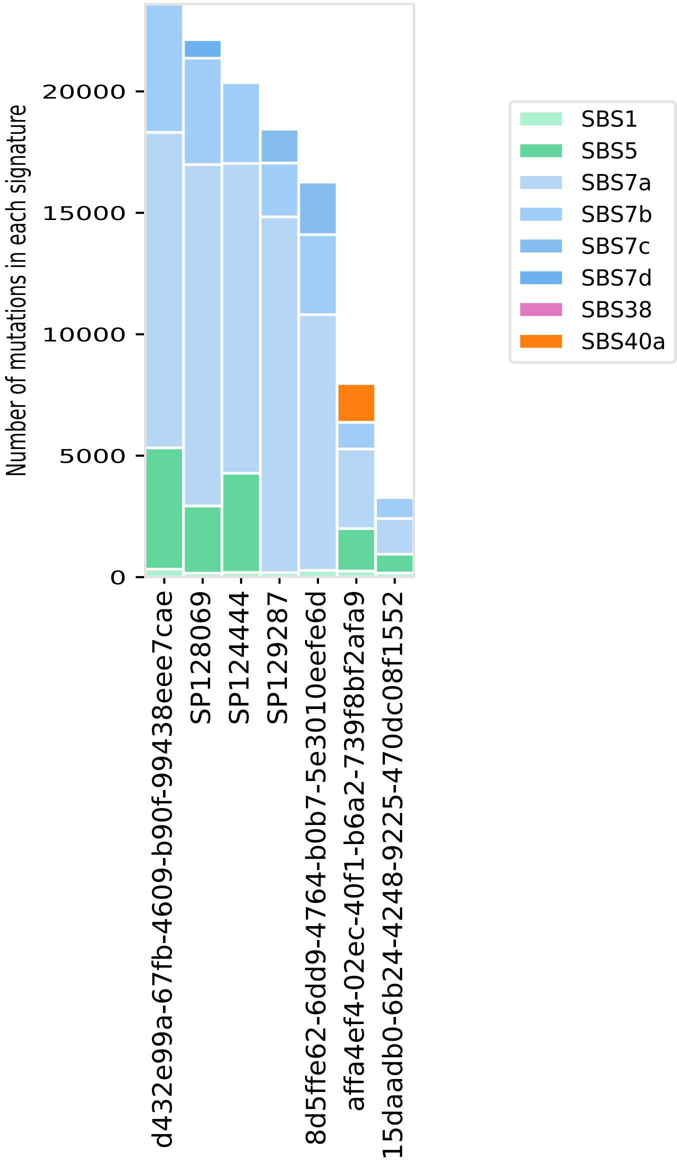

Number of mutations in each signature
0
5000
10000
15000
20000
d432e99a-67fb-4609-b90f-99438eee7cae
SP128069
SP124444
SP129287
8d5ffe62-6dd9-4764-b0b7-5e3010eefe6d
affa4ef4-02ec-40f1-b6a2-739f8bf2afa9
15daadb0-6b24-4248-9225-470dc08f1552
SBS1
SBS5
SBS7a
SBS7b
SBS7c
SBS7d
SBS38
SBS40a

**Figure S2.** The number of mutations of each COSMIC mutational signature in 99 BCC and 107 melanoma samples

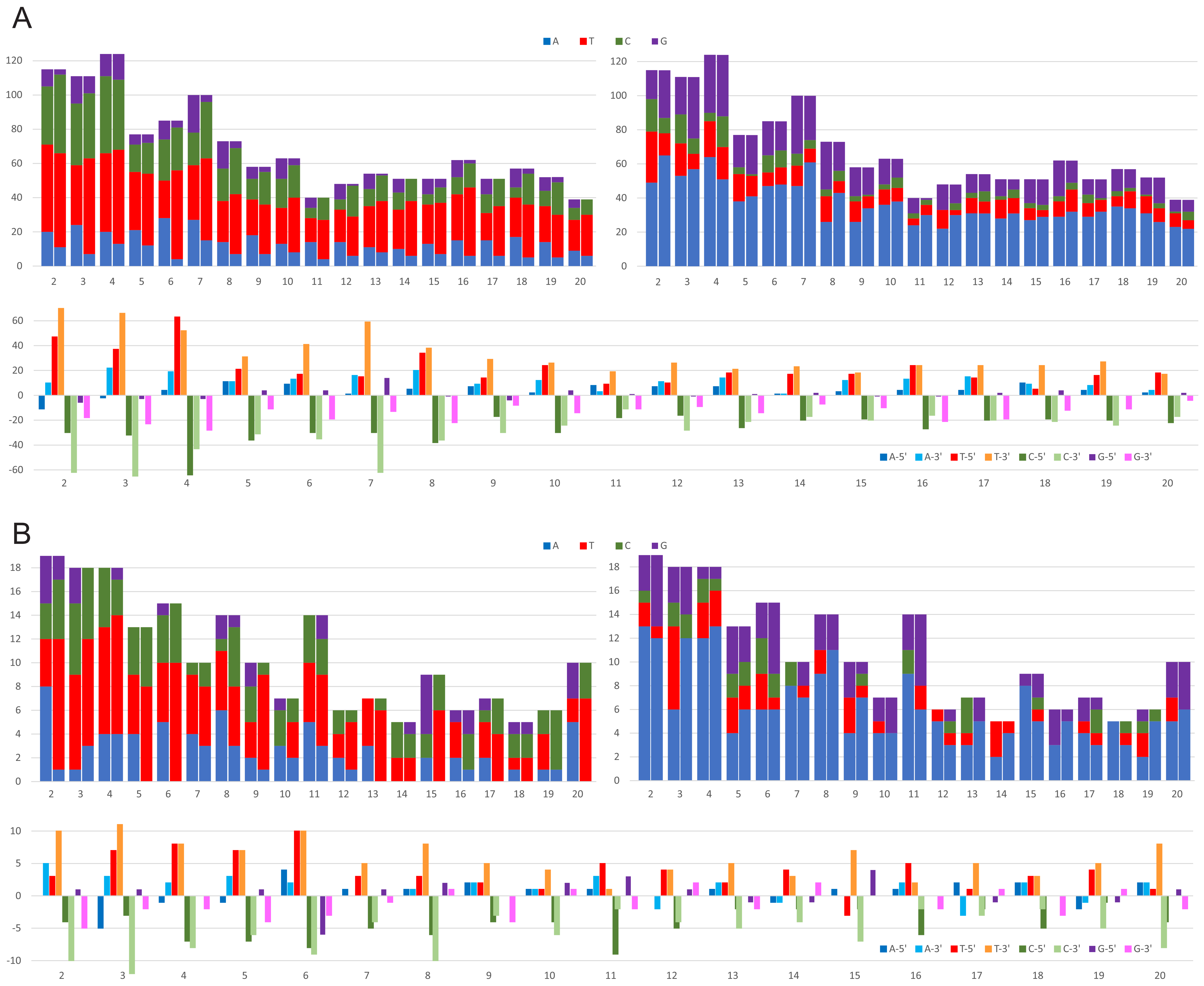

A
A
T
C
G
A-5'
A-3'
T-5'
T-3'
C-5'
C-3'
G-5'
G-3'
B

C

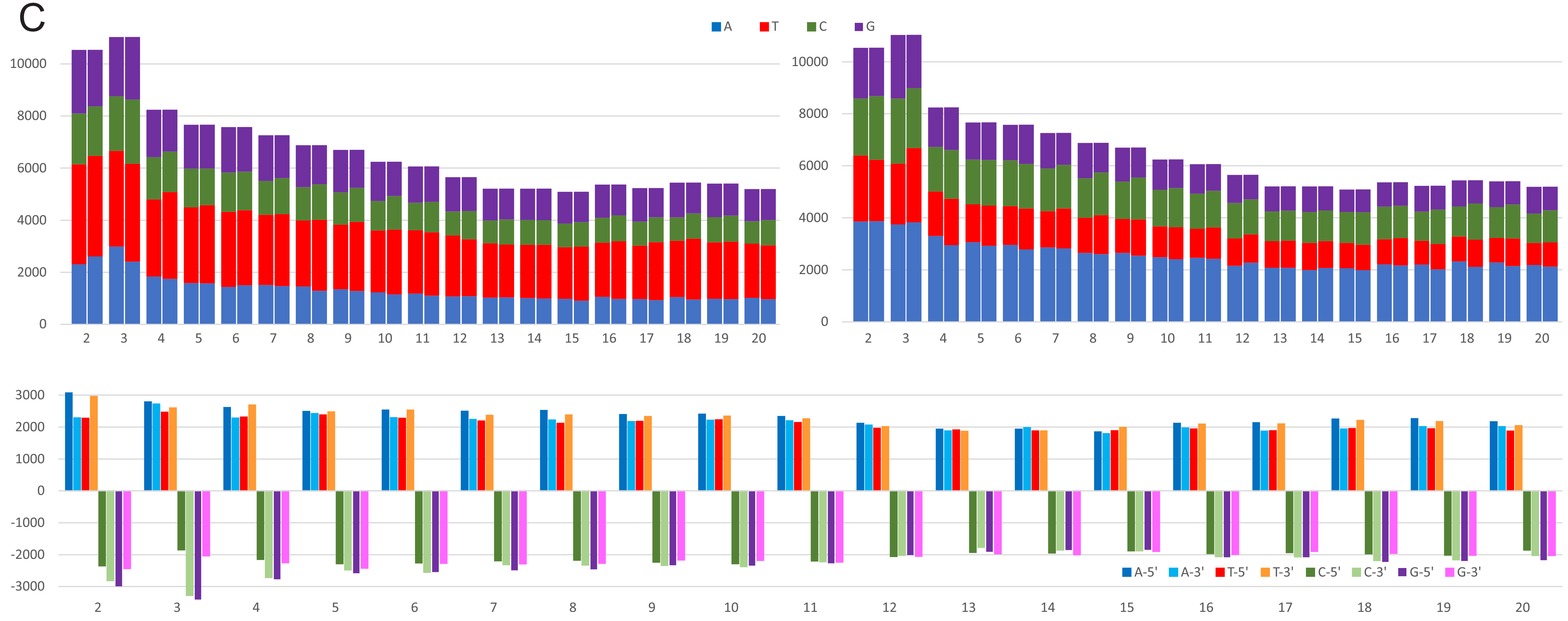
A
T
C
G
10000
8000
6000
4000
2000
0
2 3 4 5 6 7 8 9 10 11 12 13 14 15 16 17 18 19 20
3000
2000
1000
0
-1000
-2000
-3000
A-5' A-3' T-5' T-3' C-5' C-3' G-5' G-3'

D

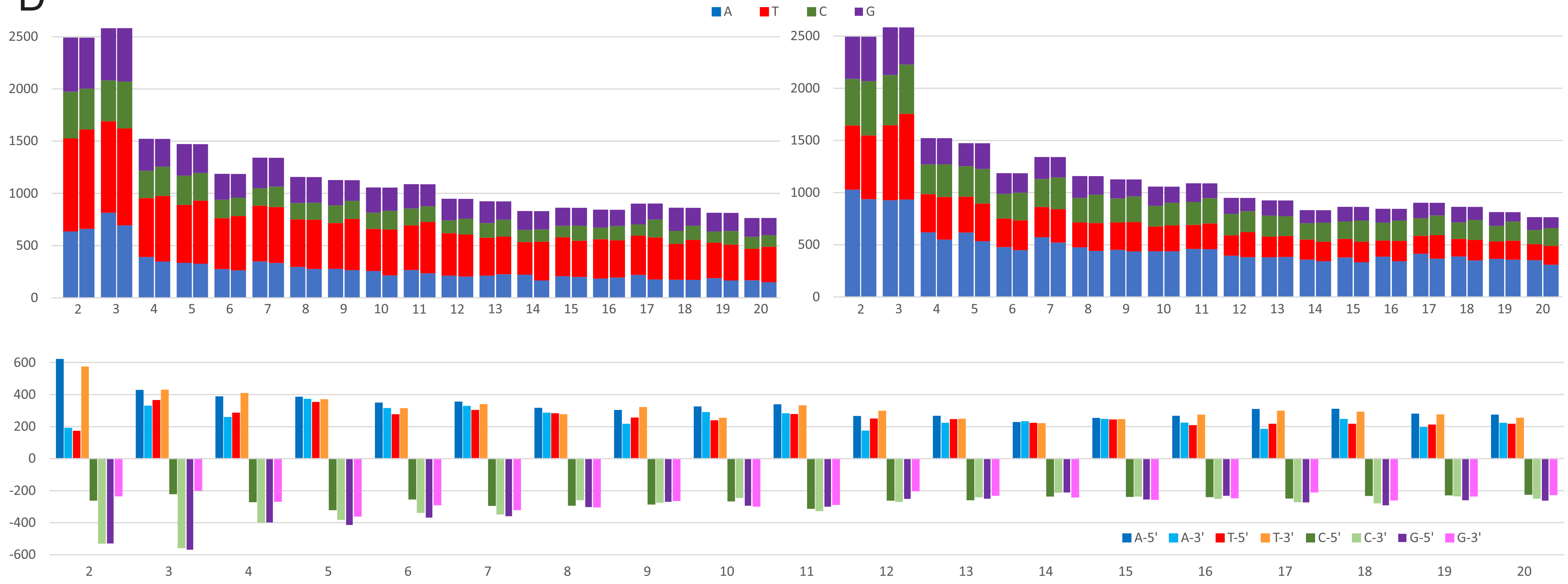
A
T
C
G
2500
2000
1500
1000
500
0
2 3 4 5 6 7 8 9 10 11 12 13 14 15 16 17 18 19 20
600
400
200
0
-200
-400
-600
A-5' A-3' T-5' T-3' C-5' C-3' G-5' G-3'

**Figure S3.** Spatial coupling between observed mutated trinucleotide pairs and their nucleotide composition.

Nucleotide composition of observed clustered mutated trinucleotides as a function of the distance between the mutated nucleotides in these trinucleotides in nonUV--UV and UV--nonUV pairs (see Figure 3, left column of plots) for **(A)** BCC and **(B)** melanoma, and in observed mutated trinucleotide pairs that do not belong to nonUV and UV patterns (see Figure 3, right column of plots) for **(C)** BCC and **(D)** melanoma. The upper pair of stacked plots shows the nucleotide composition for the first (left plot) and last (right plot) nucleotide in the paired mutated trinucleotides; for each distance, the right column shows the nucleotide composition of the 5′ trinucleotide, and the left column that of the 3′ rinucleotide. The lower bar plot shows the direction of change of the central nucleotide in the paired trinucleotide patterns as the number of observed mutated nucleotides minus the number of observed nucleotides in the original, unmutated sequence for each distance.

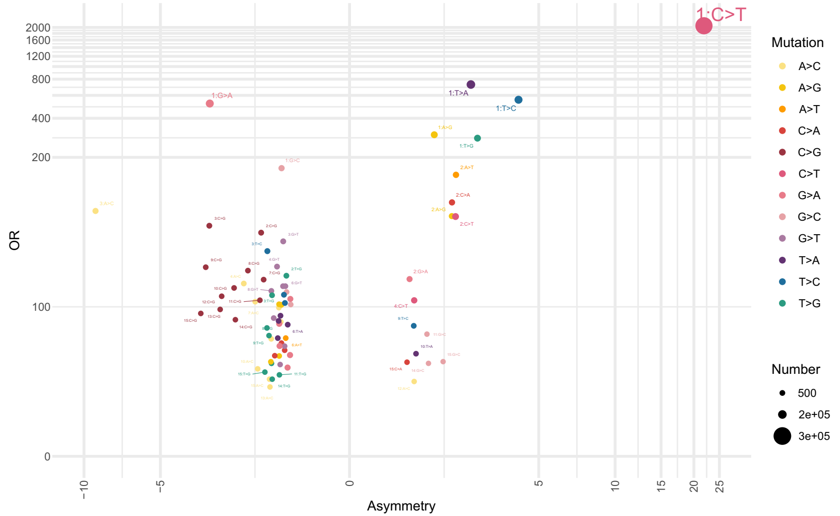
1:C>T
1:G>A
1:T>A
1:T>C
OR
Asymmetry
2000
1600
1200
800
400
200
100
0
−10
−5
0
5
10
15
20
25
Mutation
A>C
A>G
A>T
C>A
C>G
C>T
G>A
G>C
G>T
T>A
T>C
T>G
Number
500
2e+05
3e+05

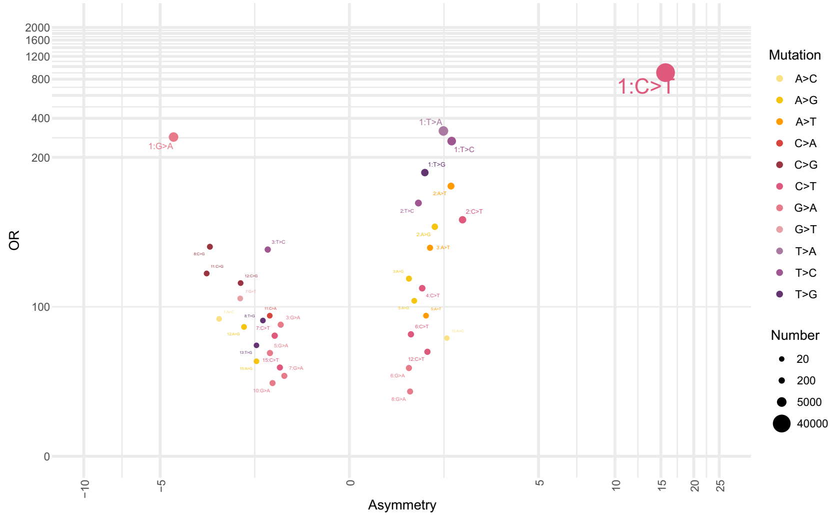
1:C>T
1:G>A
1:T>A
1:T>C
OR
Asymmetry
2000
1600
1200
800
400
200
100
0
−10
−5
0
5
10
15
20
25
Mutation
A>C
A>G
A>T
C>A
C>G
C>T
G>A
G>T
T>A
T>C
T>G
Number
20
200
5000
40000

**Figure S4.** Directional bias of ''other'' types of base-substitution pairs flanking UV-induced YY-dimers in skin cancers Asymmetry in the number of different types of mutations in paired mutations that are neither UV mutations YCR>YTR (and their reverse complements YGR>YAR)[1] nor nonUV mutations YRY>YNY (and their reverse complements RYR>RNR). Paired mutations are shown for distances up to 15 nucleotides; the distance is given from the mutated YY-dimer (and its reverse complement RR-dimer) if present in the mutated trinucleotides[2], or between the mutated nucleotides if the mutated trinucleotides do not contain a YY-dimer (and its reverse complement RR-dimer). Left plot – BCC, right plot – melanoma. The X and Y axes are in pseudo-logarithmic scale; the Y-axis shows the statistically significant ($p < 0.005$) excess of observed mutations over expected; the X-axis shows the excess (asymmetry) of the observed number of nonUV mutations in the 5′ vicinity of the YY-dimer relative to the 3′ vicinity (negative values) or in the 3′ vicinity of the YY-dimer relative to the 5′ vicinity (positive values).

[1] The analysis also excluded the more general UV patterns YCN>YTN (and their reverse complements NGR>NAR), as well as TTN>TVN and NTT>NVT (and their reverse complements NAA>NBA and AAN>ABN).

[2] In the paired combination YY--MUT, mutations were counted for the 3′ side (the right mutation in the pair); in the combination MUT--YY, mutations were counted for the 5′ side (the left mutation in the pair); in the combination YY_reverse_complement--MUT, mutations were counted for the 5′ side; in the combination MUT--YY_reverse_complement, mutations were counted for the 3′ side; in the combination MUT--MUT, mutations were counted for both the 5′ and 3′ sides

# Pseudo-Codes Section I

PC 1. Generating supplementary bam file containing only reads supporting variants under analysis

```
INPUT DATA:
$ARGV[0] — vcf file with variants under analysis
$ARGV[1] — original bam file

THE PROGRAM CODE:
If the BAM file doesn't exist:
   Check if the BED file exists
      If BED file doesn't exist, convert the VCF file to BED format using convert2bed command
   Extract reads from BAM file that match the BED regions using sambamba view command, use
"[NM]>=1” to include only those reads containing at least one mismatch, as recorded in the NM tag
   Sort the extracted BAM file using sambamba sort command
   Run a jvarkit Biostar322664 tool to extract PE Reads (--no-mate option, without their mates)
supporting variants in vcf file and generate a supplementary BAM file
   Clean up temporary files
```

## PC 2. Generating table containing all paired mutations

```
INPUT DATA:
$ARGV[0] — bed file with variants under analysis
$ARGV[1] — original bam file
$ARGV[2] — supplementary bam file (see PC 1)

THE PROGRAM CODE:
#1. Read BED Files

Initialize an empty hash %bed
For each file matching "*.bed":
    Extract the prefix as key
    Store the full filename as value in %bed

#2. Read Original BAM Files

Initialize an empty hash %bamsOri
For each file matching "*ori.bam":
    Extract the prefix as key
    Store the full filename as value in %bamsOri

#3. Read Supplementary BAM Files

Initialize an empty hash %bamsAlt
For each file matching "*supp.bam":
    Extract the prefix as key
    Store the full filename as value in %bamsAlt

#4. Main Loop Over BED Keys

For each sorted key in %bed:
    Let TID = current key
    If both original and supplementary BAMs exist for this TID:
        Let oribam = corresponding original BAM file
        For each altbam in %bamsAlt[TID]:
            Generate mpileup filenames for both alt and original BAMs using BED file, using --
output-QNAME option for making output containing the read names (QNAMEs) for every read covering
a specific genomic position:
                altmpileup = altbam
                orimpileup = oribam
            Call subroutine readsCount

# 5. Write Final Summary Table

Open file "table" for writing
Print header line to table
For each TID in sorted order:
    For each chromosome in sorted order:
        For each position pair in sorted order:
            Extract position values
            Print data to table including: TID, Chromosome, 5' and 3' positions, Reference and
alternate alleles, Distance, Total reads, Bimutated reads, 5' and 3' mutated reads

# 6. Subroutine: readsCount

Open mpileup file for reading
While reading each line from pileup:
    Parse line into fields:
        chr, pos, ref, depth, alleles, reads
    If this is the first line:
        Store line, chr, pos, ref, alleles, reads in pre-variables
    Else:
        Compare current line with previous line:
            If same chromosome and distance < 101:
                Process alleles and reads:
                    Identify 5' and 3' mutations
                    Count how many reads support:
                        - Both ends mutated
                        - Only 5' end mutated
                        - Only 3' end mutated
                        - Neither end mutated
                If filetype is "alt":
                    Add counts to %dists for the distance
                    Store mutation info in %table
                If filetype is "ori":
                    Update total read count in %table
        Update pre-variables with current line data
```

PC 3. Separating reads containing two clustered mutations from reads having only one mutation, adding mutation context to the data

```
INPUT DATA:
$ARGV[0] — table file containing all paired mutations for all samples (see PC 2)

THE PROGRAM CODE:
#1. Initialization

Two hash tables are initialized:
%bakhyTableMono — stores counts for mono-mutation cases.
%bakhyTableBi — stores counts for bi-mutation cases.

#2. Input file processing

Open the input file $ARGV[0]
Read the file line by line
   Split the line into fields (@F) using tab
   Check if the fields contain valid data
   Compute a frequency ratio from 3 fields to differentiate mono-mutation from bi-mutation cases
   Build Query Strings
      $queryA
      $queryB
   Query hg19/GRCh37 genomic data using Tabix for each query ($queryA and $queryB):
   Collect all results (@R) from the query:
      If the reference nucleotide matches the nucleotide in original table:
         Store the full pattern ($pattA, $pattB) as a 3-character string
         Store a modified pattern ($pattAm, $pattBm) where central nucleotides are replaced
   Update Hash Tables

3. Output

For %bakhyTableMono and %bakhyTableBi hashes:
   For each unique combination of patterns:
      For each of the 101 possible distances between mutations (0 to 100):
         Print the count or 0 if not present.
```

PC 4. Jackknifing samples and separating reads containing two clustered mutations from reads having only one mutation, with adding mutation context to the data (see PC 3)

```
INPUT DATA:
$ARGV[0] — table file containing list of samples to analyze
$ARGV[1] — table file containing all paired mutations for all samples (see PC 2)
$ARGV[2] — threads number
$ARGV[3] — number of iterations

THE PROGRAM CODE:
# 1. Data Loading Phase

Read sample names $ARGV[0] into array @tums
Read mutation data $ARGV[1] into hash %muts

#2. Parallel Processing Setup
Create a parallel processing manager with number of processes specified by command-line argument
$ARGV[2]
Loop for specified iterations: Run the processing loop $ARGV[3] times (second command-line
argument)
Start child process: For each iteration, fork a new child process
   Child process execution:
   Set random seed for this child process
   Call the Run() subroutine
   Exit child process

#3. Run

Initialize shuffled table list: Copy the original table names array @tums to @stums
Perform 10 random shuffles of the table names array
Take first 50 shuffled sample names from @stums
For each of these 50 tables, retrieve all associated mutation lines from hash %muts
Write all these mutation lines to output table

Run external analysis: Execute PC 3
```

**Pseudo-Codes Section II**

## PC 1. Randomizing mutation data across tumor samples to obtain random expectation about neighbouring mutations

```
INPUT DATA:

$ARGV[0] — file listed mutated real trinucleotide positions (for central nucleotide) in samples
of the sample set
$ARGV[1] – number of iterations
patterns — all possible mutated trinucleotide patterns of paired mutations.

THE PROGRAM CODE:
#1. Load reference mutation patterns in 36864 trinucleotide contexts

Initialize empty hash %patterns
Open file "patterns" for reading
   For each line in the file:
      Split line by tab delimiter into array @I
      Create key from first 4 elements (Ref0, Alt0, Ref1, Alt1) of @I joined by tabs
      Store 5th element of @I as serial number for this key in %patterns
Close the file "patterns"

#2. Load mutation data

Initialize empty arrays @mutations and @samples
Open file $ARGV[0] for reading:
   For each line in the file:
      Split line by tab delimiter into array @I
      Create mutation string "chromosome\tposition\tRef---Alt" and add to @mutations
      Add samp to @samples array
Close the input file

#3. Parallel processing setup

Create Parallel with 40 child processes
   Loop for $ARGV[1] iterations:
      Fork child process
      If in child process:
         Seed random number generator
         Call subroutine run
         Exit child process
      Wait for all child processes to complete

#4. Main processing subroutine (run)

Initialize variables:
   Copy @samples to @ssamples
   Shuffle @ssamples 10 times
   Initialize empty hash %mutationsSamples
   Initialize empty hash %patternMatrix

For each mutation in @mutations:
   Split mutation string by tab delimiter into @mut
   Assign mutation to %mutationsSamples using shuffled sample as first key, chromosome as second
key, position as third key (mutationsSamples[sampleRnd][chromosome][position]=”Ref---Alt”)

For each sample in %mutationsSamples:
   For each chromosome (sorted alphabetically):
      Initialize empty array @samplechr
      For each position (sorted numerically):
         - Add position and mutation to @samplechr as "position\tmutation"
      For each consecutive mutations in @samplechr:
         - Split first mutation into @A
         - Split second mutation into @B
         - Calculate distance: $B[0]-$A[0]
         - If distance > 100, skip to next pair
         - Else:
            - Replace "---" with tab in both mutation strings
            - Increment counter in %patternMatrix for this pattern pair (Ref0, Alt0, Ref1, Alt1)
at this distance

Open output file "rnd.$it" for writing
   For each pattern key in %patterns (sorted by pattern values):
      Print pattern key and its value
      For distances 0 to 100:
      - If pattern exists for this distance in %patternMatrix, print count
      - Else print "0"
      Print newline
Close output file
```

## PC 2. Summarize observed mutation data across tumor samples to extract neighbouring mutations

```
INPUT DATA:

$ARGV[0] —  — file listed mutated real trinucleotide positions (for central nucleotide) in
samples of the sample set
patterns — all possible mutated trinucleotide patterns of paired mutations.

THE PROGRAM CODE:
#1. Load reference mutation patterns in 36864 trinucleotide contexts

Initialize empty hash %patterns
Open file "patterns" for reading
   For each line in the file:
      Split line by tab delimiter into array @I
      Create key from first 4 elements (Ref0, Alt0, Ref1, Alt1) of @I joined by tabs
      Store 5th element of @I as serial number for this key in %patterns
Close the file "patterns"

#2. Load mutation data

Initialize empty arrays @mutations and @samples
Open file $ARGV[0] for reading:
   For each line in the file:
      Split line by tab delimiter into array @I
      Create mutation string "chromosome\tposition\tRef---Alt" and add to @mutations
      Add samp to @samples array
Close the input file

Call subroutine run

#3. Main processing subroutine (run)

Initialize variables:
   Initialize empty hash %mutationsSamples
   Initialize empty hash %patternMatrix

For each mutation in @mutations:
   Split mutation string by tab delimiter into @mut
   Assign mutation to %mutationsSamples using sample as first key, chromosome as second key,
position as third key (mutationsSamples[sample][chromosome][position]=”Ref---Alt”)

For each sample in %mutationsSamples:
   For each chromosome (sorted alphabetically):
      Initialize empty array @samplechr
      For each position (sorted numerically):
         - Add position and mutation to @samplechr as "position\tmutation"
      For each consecutive mutations in @samplechr:
         - Split first mutation into @A
         - Split second mutation into @B
         - Calculate distance: $B[0]-$A[0]
         - If distance > 100, skip to next pair
         - Else:
            - Replace "---" with tab in both mutation strings
            - Increment counter in %patternMatrix for this pattern pair (Ref0, Alt0, Ref1, Alt1)
at this distance

Open output file for writing
   For each pattern key in %patterns (sorted by pattern values):
      Print pattern key and its value
      For distances 0 to 100:
      - If pattern exists for this distance in %patternMatrix, print count
      - Else print "0"
      Print newline
Close output file
```

## PC 3. Summarize randomized mutation data to analyzed inter-mutational distances

```
INPUT DATA:

$ARGV[0] — output file prefix
$ARGV[1] — file containing mutated pattern in ambiguous nucleotide codes
patterns — all possible mutated trinucleotide patterns of paired mutations.
rnd.* - files generated by PC 1.

THE PROGRAM CODE:
#1. Initialize Hash Tables and Variables

%IU: Maps ambiguous nucleotide codes (e.g., R, Y) to their possible bases (e.g., R → A|G).
%comp: Maps each nucleotide to its complement (e.g., A → T, Y → R).
%PATTERNS — empty hash to store patterns

#2. Processing Pattern File

Open the file $ARGV[1]
   For each line in the file:
      Split the line into fields using whitespace
      Extract four fields ($P[1], $P[2], $P[3], $P[4]) to form two mutated patterns:
         Pattern 1: $P[1]-$P[3]
         Pattern 2: $P[2]-$P[4]
      Split each pattern into two parts
      If the first field ($P[0]) is "revcom":
         Reverse-complement each pattern
         Store both the original and reverse-complemented patterns in %PATTERNS
      Else if $P[0] is "dir":
         Store the original patterns directly in %PATTERNS

#3. Read Known Patterns File

Open the file named patterns.
   For each line in the file:
      Split the line by tab (\t) into fields (@X)
         Split each of the four fields ($X[0], $X[1], $X[2], $X[3]) into individual characters
   For each pattern in %PATTERNS:
      Split the pattern into reference and alternative parts
      For each character in the reference and alternative patterns:
         Check if the ambiguous base in the pattern matches the known base using the %IU hash
         Count how many characters match across all three positions for reference and alternative
            If all counts are 3 (i.e., all characters match):
               Store the known pattern in %patternsKnow

#4. Processing Data Files rnd.*

Set up a list of directories to search (@dirs).
For each directory:
   Use find to list all files with names starting with rnd.*
   For each file:
      Split the filename by . to get the file postfix
      Open the file.
         For each line in the file:
            Split by tab (\t) into fields (@F).
            Extract a pattern from the first four fields ($F[0] to $F[3])
         If the pattern exists in %patternsKnow:
            Add the values in @F to a hash %DATA indexed by position and rnd file postfix

#5. Compute Mean and Standard Deviation

Open two output files:
$ARGV[0].mean.tsv — to store means
$ARGV[0].sd.tsv — to store standard deviations
For each position in %DATA:
   Collect all values for that position across rnd file postfixes
   Compute:
      Mean of the values
      Standard deviation of the values
Write the final mean and standard deviation rows to the respective output files
```

## PC 4. Summarize observed mutation data to analyzed inter-mutational distances

```
INPUT DATA:

$ARGV[0] — output file prefix
$ARGV[1] — file containing mutated pattern in ambiguous nucleotide codes
patterns — all possible mutated trinucleotide patterns of paired mutations.
OBSERVED - file generated by PC 2.

THE PROGRAM CODE:
#1. Initialize Hash Tables and Variables

%IU: Maps ambiguous nucleotide codes (e.g., R, Y) to their possible bases (e.g., R → A|G).
%comp: Maps each nucleotide to its complement (e.g., A → T, Y → R).
%PATTERNS — empty hash to store patterns

#2. Processing Pattern File

Open the file $ARGV[1]
   For each line in the file:
      Split the line into fields using whitespace
      Extract four fields ($P[1], $P[2], $P[3], $P[4]) to form two mutated patterns:
         Pattern 1: $P[1]-$P[3]
         Pattern 2: $P[2]-$P[4]
      Split each pattern into two parts
      If the first field ($P[0]) is "revcom":
         Reverse-complement each pattern
         Store both the original and reverse-complemented patterns in %PATTERNS
      Else if $P[0] is "dir":
         Store the original patterns directly in %PATTERNS

#3. Read Known Patterns File

Open the file named patterns.
   For each line in the file:
      Split the line by tab (\t) into fields (@X)
         Split each of the four fields ($X[0], $X[1], $X[2], $X[3]) into individual characters
   For each pattern in %PATTERNS:
      Split the pattern into reference and alternative parts
      For each character in the reference and alternative patterns:
         Check if the ambiguous base in the pattern matches the known base using the %IU hash
         Count how many characters match across all three positions for reference and alternative
            If all four counts are 3 (i.e., all characters match):
               Store the known pattern in %patternsKnow

#4. Processing Data File

Open OBSERVED file:
   For each line in the file:
      Split by tab (\t) into fields (@F).
      Extract a pattern from the first four fields ($F[0] to $F[3])
      Shift the first 5 fields out of @F.
   If the pattern exists in %patternsKnow:
      Add the values in @F to a hash %DATA indexed by position

Open one output file $ARGV[0].summ.tsv
For each position in %DATA:
   Write the value
```

## PC 5. Generate table to data visualization

```
INPUT DATA:

$ARGV[0]: File with expected means
$ARGV[1]: File with expected standard deviations
$ARGV[2]: File with observed means
$ARGV[3]: Minimum distance to include
$ARGV[4]: Maximum distance to include

THE PROGRAM CODE:
#1. Read Means for Expected Values

Open $ARGV[0] file
   For each line in the file:
      Split the line by tab (\t) into an array @V.
      Extract the first element as $label
      Remove the first two elements of @V
      Loop through the remaining elements:
         Store the mean value in %exp{$label}{$i}{mean} where $i is the index
Close the file

#2. Read Standard Deviation Data for Expected Values

Open $ARGV[1] file
   For each line in the file:
      Split the line by tab into array @V.
      Extract the first element as $label.
      Remove the first two elements of @V.
      Loop through the remaining elements:
         Store the standard deviation value in %exp{$label}{$i}{sd} where $i is the index
Close the file

#3. Read Observed Data

Open $ARGV[2] file
For each line in the file:
   Split the line by tab into array @V.
   Extract the first element as $label.
   Remove the first two elements of @V.
   Loop through the remaining elements:
      Store the mean value in %obs{$label}{$i}{mean} where $i is the index
      Set the standard deviation %obs{$label}{$i}{sd} to 0 for observed data
Close the file.

#4. Process and Print Expected and Observed Data

Loop through all labels ($lb) in alphabetical order:
   For each label, loop through all positions ($ps) in ascending numerical order:
   If the position $ps is within the range specified by $ARGV[3] (min) and $ARGV[4] (max):
      Calculate:
         Maximum expected value: mean + 3 * standard_deviation
         Minimum expected value: mean - 3 * standard_deviation
         If the minimum is less than or equal to 1, set it to 1.
      Print the expected data in the format:
         $ps   expected      $mean   $maxexp $minexp
      Calculate:
         Maximum observed value: mean + 3 * 0 (since SD = 0)
         Minimum observed value: mean - 3 * 0 (since SD = 0)
         If the minimum is less than or equal to 1, set it to 1.
      Print the observed data in the format:
         $ps   observed      $mean   $maxobs $minobs
```

# Pseudo-Codes Section III

PC 1. Polarized Counting (3’ or 5’) of mutations in non-UV patterns neighbouring to YY-dimer in UV patterns, observed mutations

```
INPUT DATA:

$ARGV[0] — output file prefix
$ARGV[1] — file containing mutated pattern in ambiguous nucleotide codes
patterns — all possible mutated trinucleotide patterns of paired mutations.
OBSERVED - file generated by PC 2 of PseudoCodes II.

THE PROGRAM CODE:
#1. Initialize Hash Tables and Variables

%IU: Maps ambiguous nucleotide codes (e.g., R, Y) to their possible bases (e.g., R → A|G).
%IUanti: Maps ambiguous codes to their reverse complement codes.
%comp: Maps each nucleotide to its complement (e.g., A → T, Y → R).

%PATTERNS: Stores reference-alternative pattern pairs.
%PATTERNSout: Stores additional labels (like lY, rR) based on pattern match to YY-dimer or its
reverse comptlement.
%patternsKnow: Stores known patterns from a file (patterns).
%patternsKnowSym: Stores reverse-complement versions of known patterns.
%patternsKnowOut: Stores output labels for known patterns.
%ASYMMETRY: Stores asymmetry counts for each position and SNP.
%ASYMMETRYsum: Aggregates counts across all files for final output.

#2. Processing Pattern File

Open the file $ARGV[1]
   For each line in the file:
      Split the line into fields using whitespace
      Extract four fields ($P[1], $P[2], $P[3], $P[4]) to form two mutated patterns:
         Pattern 1: $P[1]-$P[3]
         Pattern 2: $P[2]-$P[4]
      Split each pattern into two parts
      If the first field ($P[0]) is "revcom":
         Reverse-complement each pattern
         Store both the original and reverse-complemented patterns in %PATTERNS
         Add labels (lY, lR, rY, rR) to %PATTERNSout if certain conditions (orientation to YY-
dimer in UV patterns) match.
      Else if $P[0] is "dir":
         Store the original patterns directly in %PATTERNS
         Add labels (lY, lR, rY, rR) to %PATTERNSout if certain conditions (orientation to YY-
dimer in UV patterns) match.

#3. Read Known Patterns File

Open the file named patterns.
   For each line in the file:
      Split the line by tab (\t) into fields (@X)
         Split each of the four fields ($X[0], $X[1], $X[2], $X[3]) into individual characters
   For each pattern in %PATTERNS:
      Split the pattern into reference and alternative parts
      For each character in the reference and alternative patterns:
         Check if the ambiguous base in the pattern matches the known base using the %IU hash
         Count how many characters match across all three positions for reference and alternative
            If all counts are 3 (i.e., all characters match):
               Store the pattern and its reverse-complement in %patternsKnow and
%patternsKnowSym.
               Store associated labels in %patternsKnowOut.

#4. Processing Data File

Open OBSERVED file:
   For each line in the file:
      Split by tab (\t) into fields (@F).
      Extract a pattern from the first four fields ($F[0] to $F[3])
   If the pattern exists in %patternsKnow:
     Determine which direction (l or r) the asymmetry is based on the labels in %patternsKnowOut.
```

```
      Update %ASYMMETRY with counts based on:
         The distance
         The SNP transition
         Direction (l or r)

#5. Aggregate Asymmetry Counts

For each position and SNP in %ASYMMETRY:
   Sum up the counts for each direction (l and r).
   Store the totals in %ASYMMETRYsum.

#6. Perform Fisher's Exact Test and Output Results

Open output file $ARGV[0].summ.tsv.
For each SNP in %ASYMMETRYsum:
   For each distance in the file:
      Get left (L) and right (R) counts
      Compute expected (E) value as average of L and R
      Run Fisher's exact test in R with a 2x2 matrix:
      Get p-value
         If p-value > 0.1, label as "ns" (not significant)
      Calculate a log-odds ratio (lo) for the asymmetry with pseudo-counts as (L+0.5)/(R+0.5)
      Output the result in tab-separated format:
         change    lo   p-value (L/R)

The final output is written to $ARGV[0].summ.tsv.
```

## PC 2. Polarized Counting (3’ or 5’) of mutations in non-UV patterns neighbouring to YY-dimer in UV patterns, randomized data

```
INPUT DATA:

$ARGV[0] — output file prefix
$ARGV[1] — file containing mutated pattern in ambiguous nucleotide codes
patterns — all possible mutated trinucleotide patterns of paired mutations.
rnd.* - files generated by PC 1 of PseudoCodes II.

THE PROGRAM CODE:
#1. Initialize Hash Tables and Variables

%IU: Maps ambiguous nucleotide codes (e.g., R, Y) to their possible bases (e.g., R → A|G).
%IUanti: Maps ambiguous codes to their reverse complement codes.
%comp: Maps each nucleotide to its complement (e.g., A → T, Y → R).

%PATTERNS: Stores reference-alternative pattern pairs.
%PATTERNSout: Stores additional labels (like lY, rR) based on pattern match to YY-dimer or its
reverse comptlement.
%patternsKnow: Stores known patterns from a file (patterns).
%patternsKnowSym: Stores reverse-complement versions of known patterns.
%patternsKnowOut: Stores output labels for known patterns.
%ASYMMETRY: Stores asymmetry counts for each position and SNP.

#2. Processing Pattern File

Open the file $ARGV[1]
   For each line in the file:
      Split the line into fields using whitespace
      Extract four fields ($P[1], $P[2], $P[3], $P[4]) to form two mutated patterns:
         Pattern 1: $P[1]-$P[3]
         Pattern 2: $P[2]-$P[4]
      Split each pattern into two parts
      If the first field ($P[0]) is "revcom":
         Reverse-complement each pattern
         Store both the original and reverse-complemented patterns in %PATTERNS
         Add labels (lY, lR, rY, rR) to %PATTERNSout if certain conditions (orientation to YY-
dimer in UV patterns) match.
      Else if $P[0] is "dir":
         Store the original patterns directly in %PATTERNS
         Add labels (lY, lR, rY, rR) to %PATTERNSout if certain conditions (orientation to YY-
dimer in UV patterns) match.

#3. Read Known Patterns File

Open the file named patterns.
   For each line in the file:
      Split the line by tab (\t) into fields (@X)
         Split each of the four fields ($X[0], $X[1], $X[2], $X[3]) into individual characters
   For each pattern in %PATTERNS:
      Split the pattern into reference and alternative parts
      For each character in the reference and alternative patterns:
         Check if the ambiguous base in the pattern matches the known base using the %IU hash
         Count how many characters match across all three positions for reference and alternative
            If all counts are 3 (i.e., all characters match):
               Store the pattern and its reverse-complement in %patternsKnow and
%patternsKnowSym.
               Store associated labels in %patternsKnowOut.

#4. Processing Data Files rnd.*

Set up a list of directories to search (@dirs).
For each directory:
   Use find to list all files with names starting with rnd.*
   For each file:
      Split the filename by . to get the file postfix
      Open the file.
         For each line in the file:
            Split by tab (\t) into fields (@F).
            Extract a pattern from the first four fields ($F[0] to $F[3])
         If the pattern exists in %patternsKnow:
            Determine which direction (l or r) the asymmetry is based on the labels in
%patternsKnowOut.
```

```
            Update %ASYMMETRY with counts based on:
               The distance
               The file name
               The SNP transition
               Direction (l or r)

#5. Computing data distribution (and Mean and Standard Deviation)
Initialize %ASYMMETRYsum to store summed values per SNP, distance, and direction.
For each position and SNP in %ASYMMETRY:
   Aggregate values for left (l) and right (r) directions.
   Compute mean and standard deviation for each.
Output results to three files:
   $ARGV[0].nms.tsv: Raw values per SNP
   $ARGV[0].mean.tsv: Means per SNP
   $ARGV[0].sd.tsv: Standard deviations per SNP
```

PC 3. Non-parametric comparison of polarized counting (3' or 5') of mutations in non-UV patterns neighbouring to YY-dimer in UV patterns between real and randomized cases (see PC 1 and PC 2)

```
INPUT DATA:

$ARGV[0] → Real data file
$ARGV[1] → Simulated data file (.nms.tsv: Raw values per SNP, see PC 2)

THE PROGRAM CODE:
#1. Loop Through Lines of Both Files

Open files $ARGV[0] and $ARGV[1]
   Read one line from each file ($X from real data, $Y from simulated data).
   Process Each Line
      If both lines are defined:
         Split each line by tab (\t) into arrays @X and @Y.
         Remove the first element from both arrays ($x0, $y0) which are SNP.
         Check if both arrays have the same number of elements and if their first elements ($x0, $y0) match:
         If yes:
            Print the identifier ($x0) to the output file.
            Loop through each column in the arrays:
            Split the observed L R into numerator (xl) and denominator (xr).
            Split the corresponding value from simulated data (Y) into numerator (yl) and denominator (yr).
            Split yl and yr by spaces to extract the actual values.

            Store the values from yl in array @vals
            Set obs = xl.
            Call stats function to compute q1, med, q3, OR, and p.

            Repeat for yr

            Compute observed rate ($obsrate) and expected rate ($exprate) based on comparisons between xl and xr:
               If xl > xr and xr > 0:
                  $obsrate = xl / xr
                  $exprate = q3l / q1r (if q1r > 0, otherwise "NA")
               Else if xl > xr and xr == 0:
                  $obsrate = xl
                  $exprate = q3l
               Else if xr >= xl and xl > 0:
                  $obsrate = -xr / xl
                  $exprate = -q3r / q1l (if q1l > 0, otherwise "NA")
               Else if xr > xl and xl == 0:
                  $obsrate = -xr
                  $exprate = -q3r
               Else:
                  $exprate = "NI" (Not Implemented)
   Output the computed values to the output file in tab-separated format: $obsrate/$exprate, $xl/$xr, $q1l/$q1r, $q3l/$q3r, $ORl/$Orr, $pl/$pr
   Continue looping until both input files are completely read.

#2. Subroutine: stats

Compute:
   First quartile (q1)
   Median (med)
   Third quartile (q3)
   Compute odds ratio (OR):
      If q3 - q1 > 0:
         OR = (obs - med) / (q3 - q1)
      Else: OR = "NA"
   Compute p-value (p):
      Count how many values in @vals are greater than or equal to obs.
         If count > 0:
            p = count / total_count
         Else:
            p = 1 / (2 * total_count)
   If p > 0.1:
      Set p = "NS" (Not Significant)
```

## PC 4. Visualize statistically significant non-parametric comparison of polarized counting (3' or 5')

```
INPUT DATA:

$ARGV[0] → tsv file from PC 3
$ARGV[1] – p-value threshold (0.01)

THE PROGRAM CODE:

Open the input file specified by $ARGV[0] for reading
Process Each Line of Input File
   Split the line by tab characters into array @I
   Extract the first element as $ch (mutation)
   For each column, split the value by spaces into array @vals
      Split the first element by "L/R" into @asym (asymmetry values)
      Split the second element by "L/R" into @realvs (number of counts)
      Split the fifth element by "L/R" into @ors (odds ratio values)
      Split the sixth element by "L/R" into @pvs (p-value values)

      Check if either both asymmetry values are valid (not "NA" or "NI") OR if the first is valid
and second is "NA" or "NI"
      Process Valid Asymmetry Values
         If absolute value of L asymmetry is greater than absolute value of R:
            Set $asym = -1 * asym[0] (negative of first asymmetry value)
         Check p-values and odds ratios against threshold $ARGV[1]:
            If both p-values are below threshold and both odds ratios are valid:
               If asymmetry is positive: store real value in %listLR using R odds ratio
               If asymmetry is negative: store real value in %listLR using L odds ratio
            If L p-value is below threshold, R p-value is above threshold, and R odds ratio is
valid:
               If asymmetry is positive: store real value in %listR using R odds ratio
            If L p-value is above threshold, R p-value is below threshold, and L odds ratio is
valid:
               If asymmetry is negative: store real value in %listL using L odds ratio
            If L p-value is "NS" (not significant) and R p-value is valid and R odds ratio is
valid:
               If R p-value is below threshold:
                  If asymmetry is positive: store real value in %listR using R odds ratio
            If L p-value is valid and R p-value is "NS" and L odds ratio is valid:
               If L p-value is below threshold:
                  If asymmetry is negative: store real value in %listL using L odds ratio

Output Results to File in the Format:
Mutation       Distance       Label   Asymmetry      OR     Number
```

# Pseudo-Codes Section IV

PC 1. The pseudocode for program generating randomly mutated genome positions neighbour to real (observed) to implicitly consider nucleotide genome context.

```
INPUT DATA:
$ARGV[0] — file listed mappable hg19/GRCh37 genome positions
$ARGV[1] — file listed mutated real trinucleotide positions (for central nucleotide)
$ARGV[2] — distance threshold to mutated real trinucleotide positions (10kb)
NNN.lst.gz — files listed all trinucleotide positions (for central nucleotide) in hg19/GRCh37
genome.

THE PROGRAM CODE:
#1. Reading mappable hg19/GRCh37 genome positions

OPEN file from $ARGV[0]
FOR each line in file:
    PARSE line by tab
    FOR position from start to end:
            STORE position in hash:
            noblacklist[chromosome][position] = 1
    END FOR
END FOR
CLOSE file

#2. Reading of mutated trinucleotide positions (for central nucleotide)

OPEN file from $ARGV[1]
FOR each line in file:
    PARSE line by tab
    CALCULATE 500kBlock = int(position / 500000)
    STORE position in hash:
        posrealtrinuc[trinucleotide][chromosome][500kBlock][position] = 1
END FOR
CLOSE file

#3. Processing of all trinucleotide positions in hg19/GRCh37 genome (for central nucleotide), 64
files, NNN.lst.gz format

FOR each file "*.lst.gz":
    RESET @mutations array
    EXTRACT trinucleotide from filename

    OPEN compressed file (.lst.gz)
    WHILE reading lines:
        PARSE line by tab
        IF position exists in noblacklist AND trinuc exists in posrealtrinuc:
            FIND minimum_distance to any real position in same 500kBlock
            IF minimum_distance <= threshold from $ARGV[2]:
                ADD line to @mutations array
        END IF
    END WHILE
    CLOSE file

    SHUFFLE @mutations array 10 times

    CREATE VCF file with name $vcf
    WRITE VCF header
    FOR first 300000 values in @mutations:
        EXTRACT chromosome and position
        WRITE VCF record:
            REF = original nucleotide (from 2nd nucleotide of trinucleotide from filename)
            ALT = nucleotide from {A,T,G,C} excluding REF
    END FOR
    CLOSE VCF file
END FOR
```

PC 2. The pseudocode for program counting minimal distance from mutated genome positions (from randomly generated or expected positions, see PC 1, and from observed) to YY/RR-dinucleotide.

```
INPUT DATA:
$ARGV[0] — distance threshold to YY/RR-dinucleotide (100)

THE PROGRAM CODE:
#1. Initialize an array of dinucleotides and output filename:

dinuc = ["CC", "TT", "AA", "GG", "CT", "TC", "GA", "AG"]
O = open "outname.$ARGV[0].FCON" for writing
For each .vcf.gz file (tumor sample or random data for reference genome):
    Parse filename to get trinucleotide

#2. Extract original trinucleotides coordinates and sequence

    Open temporary BED file
    OPEN compressed file (.vcf.gz)
    For each line in VCF:
        Skip header lines
        Split line by tab
        Extract chromosome (chr), position (pos)
        If ref and alt are single nucleotides:
            Skip if chromosome is "MT" (mitochondrial)
            Calculate pre and post positions & Write to BED file (chr, pos-2, pos+1)
    Close VCF and BED files
    Run bedtools getfasta to extract genomic sequences in hg19/GRCh37 genome for BED coordinates
    Save sequences in hash:
    patts{"chr:pos"} = sequence

#3. Extract neighbour to trinucleotides YY/RR-dinucleotide context with distance(s) to it

    OPEN compressed VCF file again
    For each line:
        Skip header lines
        Split line by tab
        Skip if chromosome is "MT"
        If ref and alt are single nucleotides:
            If sequence exists for this position in patts:
                Compare reference nucleotide with sequence
                If match:
                    Generate change string (e.g., "A(T>G)C")
                    Generate end positions: pos-$ARGV[0], pos+$ARGV[0]
                    Write to BED file (smp.1.bed) for forward context
                    Write to BED file (smp.2.bed) for reverse context

    Run bedtools getfasta to extract genomic sequences in hg19/GRCh37 genome on smp.1.bed and
smp.2.bed to get: smp.1.fa (forward context) & smp.2.fa (reverse context)

    For each forward sequence in smp.1.fa:
        Extract change string from header
        For each dinucleotide in dinuc[]:
            Find distance to forward dinucleotide in sequence
            Update count in distTOpirdim[dinuc][distance][change]
    For each reverse sequence in smp.2.fa:
        Extract change string from header
        Reverse the sequence
        For each reverse dinucleotide in dinuc[]:
            Find distance to reverse dinucleotide in sequence
            Update count in distTOpirdim[dinuc][distance][change]

    Remove temporary files
End loop (.vcf.gz files)

#4. Output Results

For each dinucleotide in dinuc:
    For each change in sorted changes:
        Print dinucleotide and change
        For each position from -ARGV[0]+1 to ARGV[0]-1:
            If count exists:
                Print count
            Else:
                Print "0.000"
        End line
End For loops
```

PC 3. The pseudocode for program computing general statistics for minimal distance from mutated genome positions to YY/RR-dinucleotides, for observed and expected data (see PC 1, PC 2).

```
INPUT DATA:
$ARGV[0] — file with observed data
$ARGV[1] — file with randomized/expected data
$ARGV[2] — lower threshold of distance
$ARGV[3] — upper threshold of distance

THE PROGRAM CODE:
#1. Processing observed data

open file $ARGV[0]
while reading line:
    for the first line save @I into @heads
    remove newline character from line
    split line by tab to make @I
        Extract Dinucleotide and Change
        Generate hash key str = chg + ":" + dn
        for i = 0 to length(@I):
            value = @I[i]
            head = @distances[i]
            if head >= $ARGV[2] and head <= $ARGV[3] and value > 0:
                for j = 1 to value:
                    append head to REAL[str][3]
            if head <= -$ARGV[2] and head >= -$ARGV[3] and value > 0:
                for j = 1 to value:
                    append -head to REAL[str][5]

#2. Processing expected data

open file $ARGV[1]
while reading line:
    for the first line save @I into @heads
    remove newline character from line
    split line by tab to make @I
        Extract Dinucleotide and Change
        Generate hash key str = chg + ":" + dn
        for i = 0 to length(@I):
            value = @I[i]
            head = @distances[i]
            if head >= $ARGV[2] and head <= $ARGV[3] and value > 0:
                for j = 1 to value:
                    append head to RANDOM[str][3]
            if head <= -$ARGV[2] and head >= -$ARGV[3] and value > 0:
                for j = 1 to value:
                    append -head to RANDOM[str][5]

#3. Statistics computation

for each str in sorted keys of RANDOM:
    split str into chg and dn using ":"
    vals = REAL[str][5], REAL[str][3], RANDOM[str][5], RANDOM[str][3]
    compute statistics for vals:
        mean
        geometric_mean
        harmonic_mean
        median
        q25
        q75
        n
```

PC 4. The pseudocode for program comparing observed minimal distances from mutated genome positions to YY/RR-dinucleotides with expected one (see PC 3).

```
INPUT DATA:
$ARGV[0] — input file with distance statistics

THE PROGRAM CODE:
open file $ARGV[0]
   read line-by-line:
   split each line by tab into an array @I:
      first two elements of @I:
         $chg is change.
         $dn is dinucleotide.
         $str = "$chg:$dn".
      from the remaining elements of @I, values are calculated:
         s5m: observed – expected for means, 5’ end
         s5gm: observed – expected for geometric_means, 5’ end
         s5hm: observed – expected for harmonic_means, 5’ end
         s5md: observed – expected for medians, 5’ end
         s3m: observed – expected for means, 3’ end
         s3gm: observed – expected for geometric_means, 3’ end
         s3hm: observed – expected for harmonic_means, 3’ end
         s3md: observed – expected for medians, 3’ end
         mms5=($s5me+$s5gm+$s5hm+$s5md+0.000001)/4
         mms3=($s3me+$s3gm+$s3hm+$s3md+0.000001)/4
         factor5: ratio of observed n over expected, 5’ end
         factor3: ratio of observed n over expected, 3’ end
```

## PC 5. Generate WebLogo representation (see PC 4).

```
INPUT DATA:
$ARGV[0] — input file with mms5 and mms3 scores
$ARGV[1] — threshold (1)

THE PROGRAM CODE:
open file $ARGV[0]
   read Input Line-by-Line:
   split each line by tab into an array @I
   extract original and mutated trinucleotide and neighbour dinucleotide and generate pseudo-
sequence (e.g. Ref Alt ~~ DN)
   use mms5 and mms3 scores to determine which group the pseudo-sequence belongs to:
      if mms5 >= $ARGV[1]:
         add pseudo-sequence to %seqs{"5pos"}, value is incremented by 50
      if mms5 <= -$ARGV[1]:
         add pseudo-sequence to %seqs{"5neg"}, value is incremented by 50
      if mms3 >= $ARGV[1]:
         add pseudo-sequence to %seqs{"3pos"}, value is incremented by 50
      if mms3 <= -$ARGV[1]:
         add pseudo-sequence to %seqs{"3neg"}, value is incremented by 50

for each key (category) in %seqs:
   open a new FASTA file for writing ($ARGV[0].p.fa), where p is the category
      for each sequence in the current category:
         write the sequence to the FASTA file multiple times:
         the number of times is determined by int($seqs{p}{pseudo-sequence}) + 1
         generate WebLogo:
            run the weblogo command to generate a PDF visualization
```
