# Supplementary material for: Two Faces of UV Mutagenesis: Independent and Collateral Mutagenesis in Skin Cancers
Source: Life (Basel). 2026 Aug 7;16(8):1300. doi: 10.3390/life16081300 (PMC13514280; doi:10.3390/life16081300)
Supplement: Supplementary file 1 [file life-16-01300-s001.zip › Supplementary_file_S1.zip!/BCC_SigProfilerClusters_intradistance_plots_6144_corrected.pdf]

VS187_T01_BCC_D1_00x_Vs_x01_VS187_N02_skin_D1_00x_twicefiltered

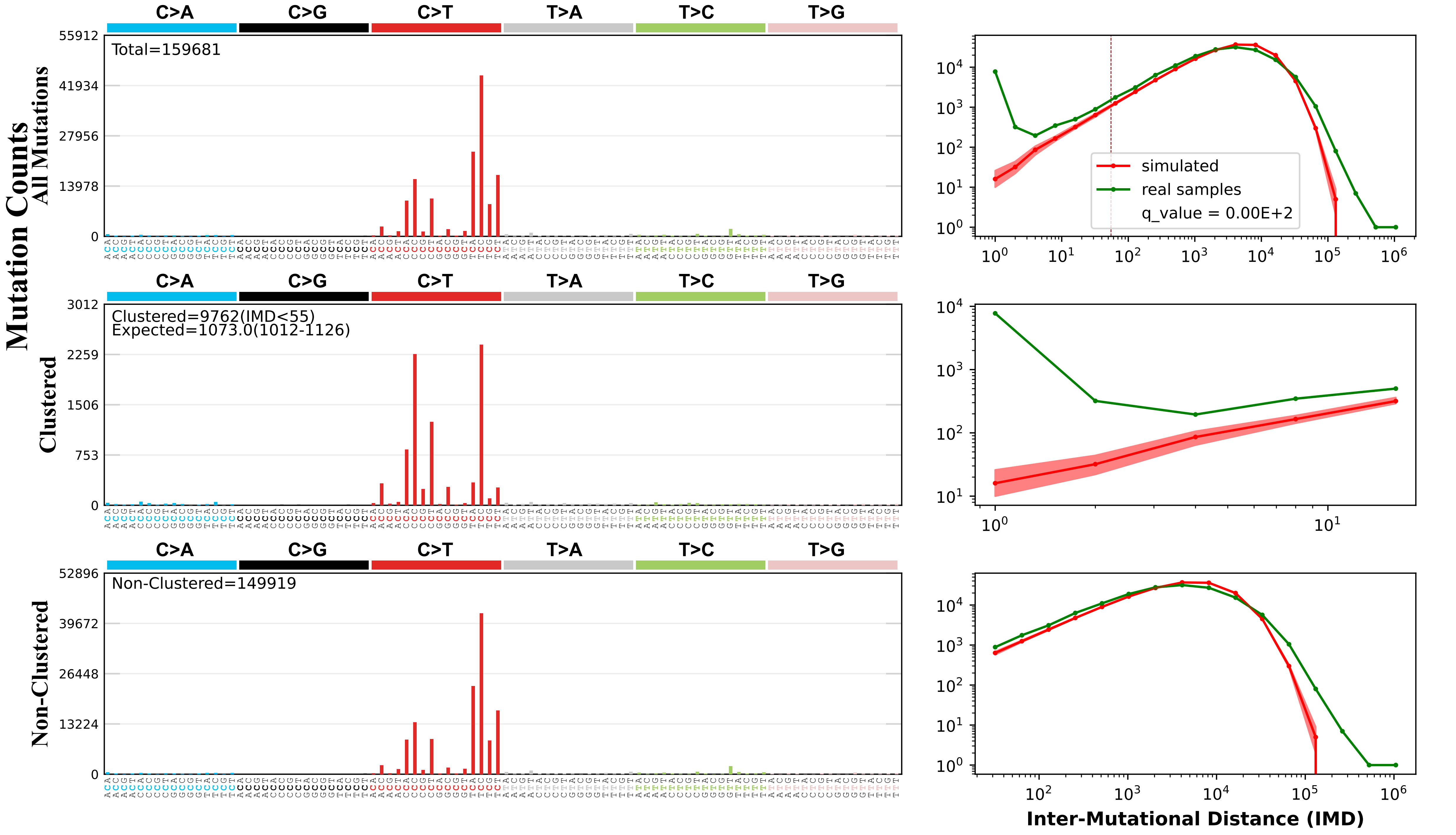

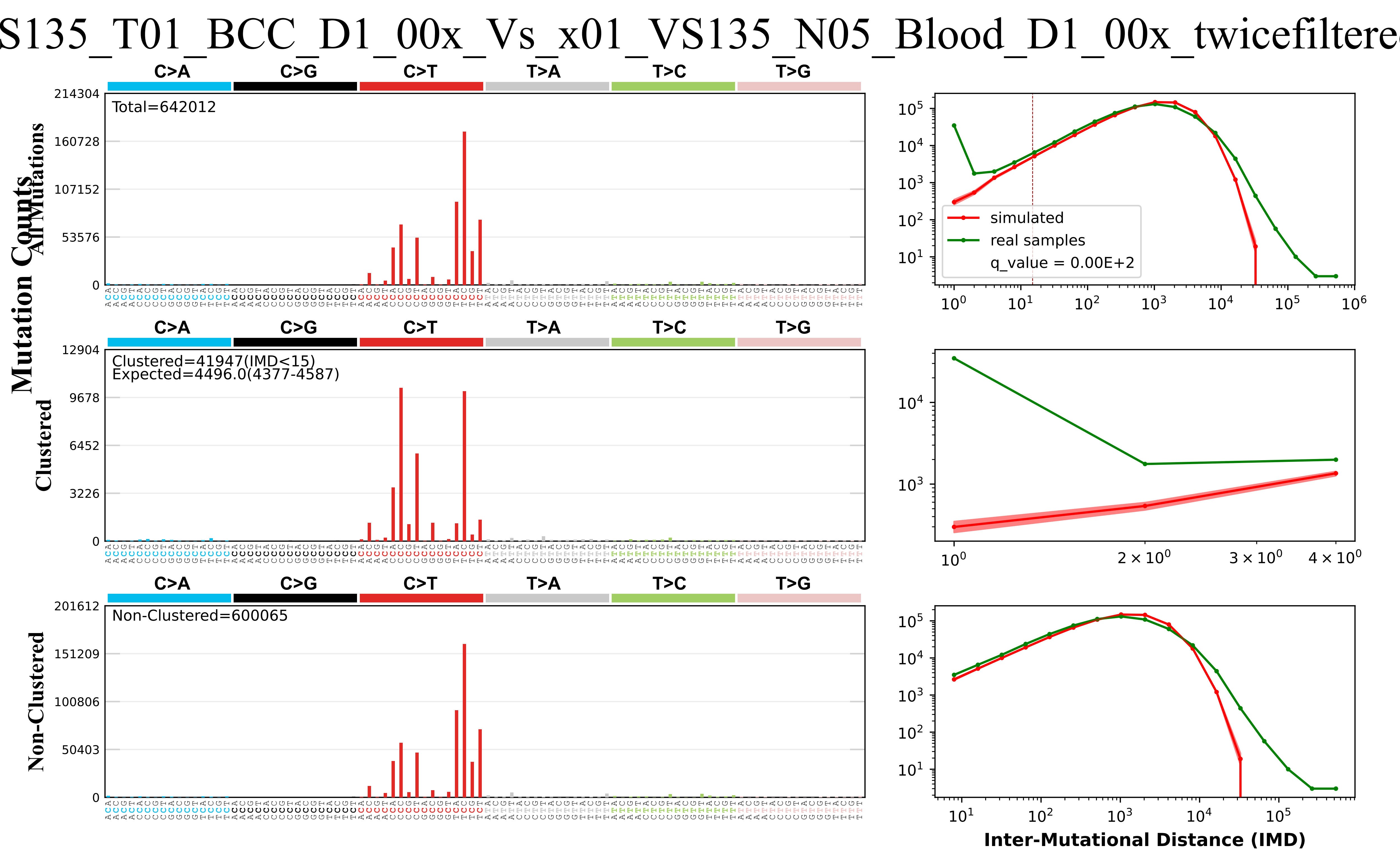

S135_T01_BCC_D1_00x_Vs_x01_VS135_N05_Blood_D1_00x_twicefiltere
Mutation Counts
All Mutations
Total=642012
C>A
C>G
C>T
T>A
T>C
T>G
214304
160728
107152
53576
0
simulated
real samples
q_value = 0.00E+2
Clustered
Clustered=41947(IMD<15)
Expected=4496.0(4377-4587)
12904
9678
6452
3226
Non-Clustered
Non-Clustered=600065
201612
151209
100806
50403
Inter-Mutational Distance (IMD)

/S123_T04_BCC_D1_00x_Vs_x01_VS123_N08_skin_D1_00x_twicefiltered

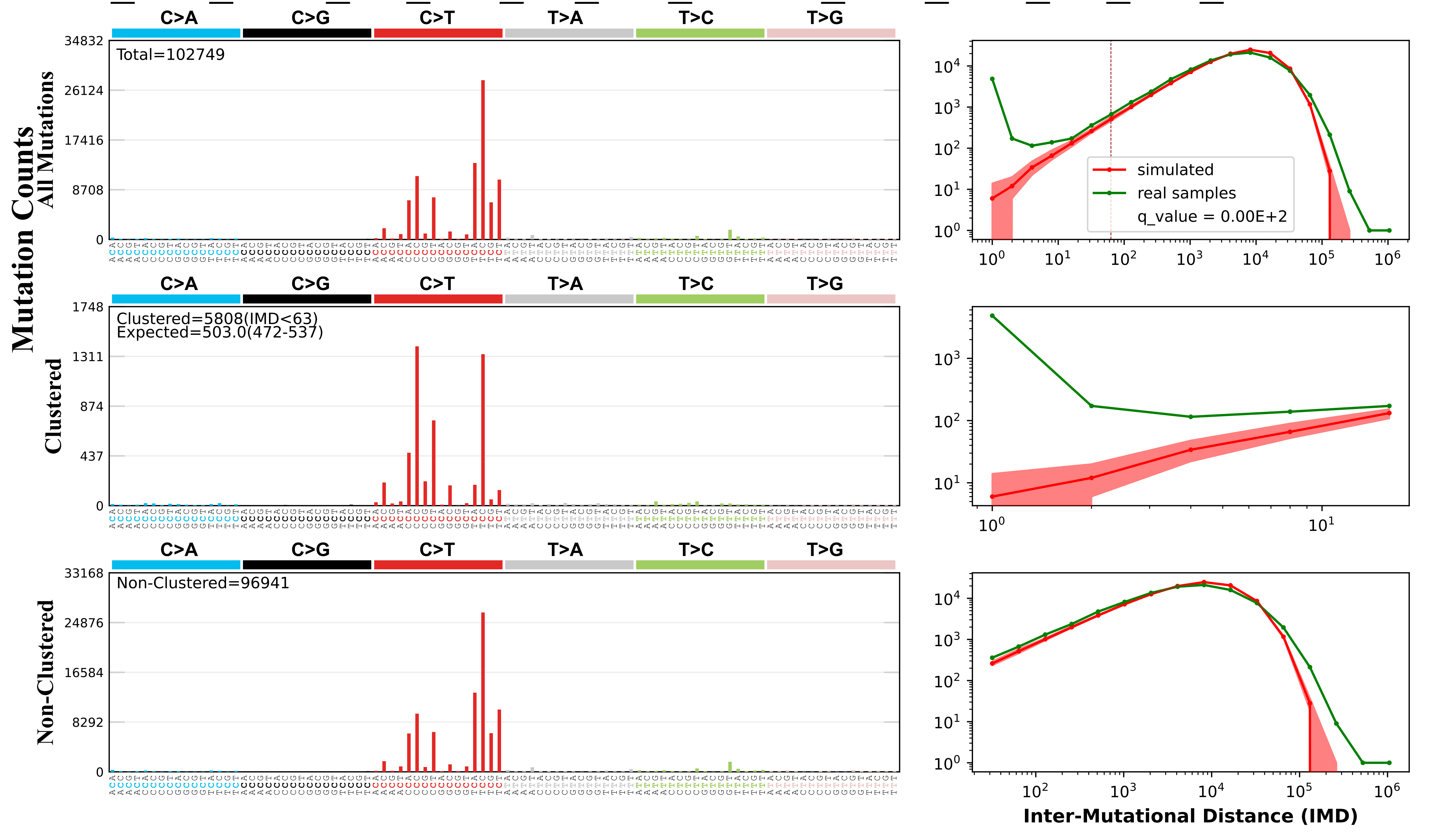

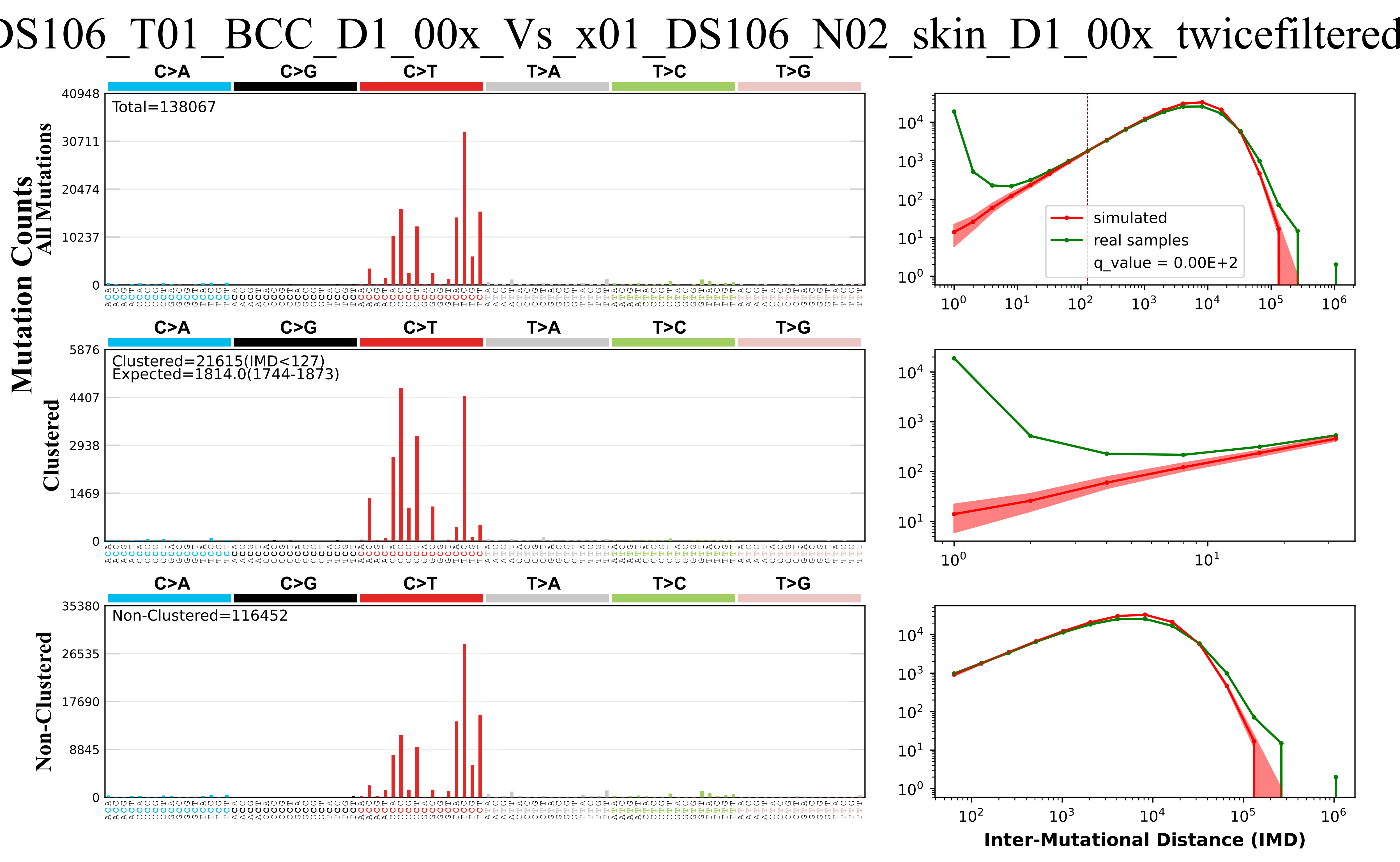

DS106_T01_BCC_D1_00x_Vs_x01_DS106_N02_skin_D1_00x_twicefiltered
Mutation Counts
All Mutations
Total=138067
Clustered
Clustered=21615(IMD<127)
Expected=1814.0(1744-1873)
Non-Clustered
Non-Clustered=116452
C>A
C>G
C>T
T>A
T>C
T>G
simulated
real samples
q_value = 0.00E+2
Inter-Mutational Distance (IMD)

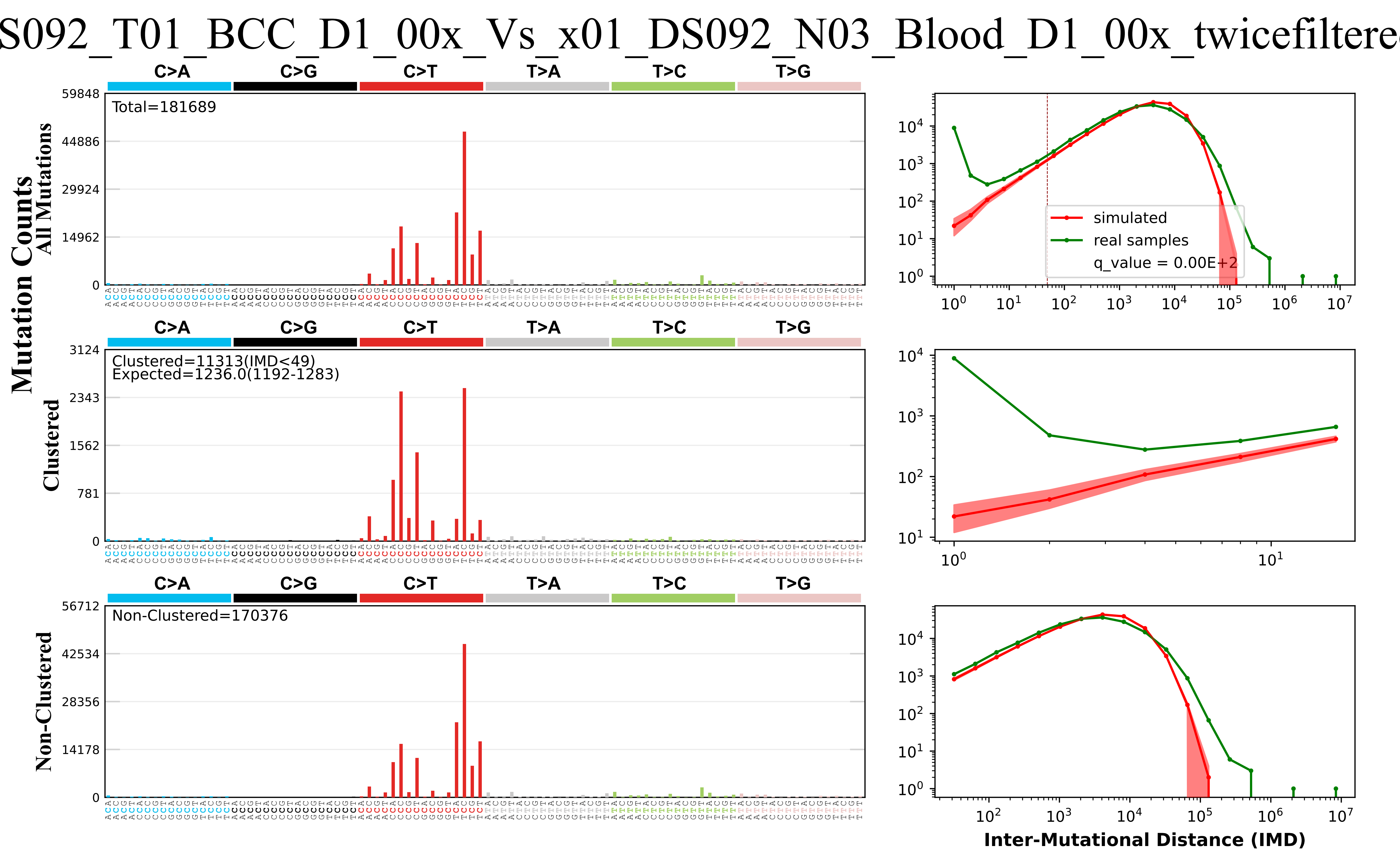
S092_T01_BCC_D1_00x_Vs_x01_DS092_N03_Blood_D1_00x_twicefiltere
Mutation Counts
All Mutations
Total=181689
Clustered
Clustered=11313(IMD<49)
Expected=1236.0(1192-1283)
Non-Clustered
Non-Clustered=170376
C>A
C>G
C>T
T>A
T>C
T>G
simulated
real samples
q_value = 0.00E+2
Inter-Mutational Distance (IMD)

DS099_T01_BCC_D1_01x_Vs_x01_DS099_N02_skin_D1_00x_twicefiltered

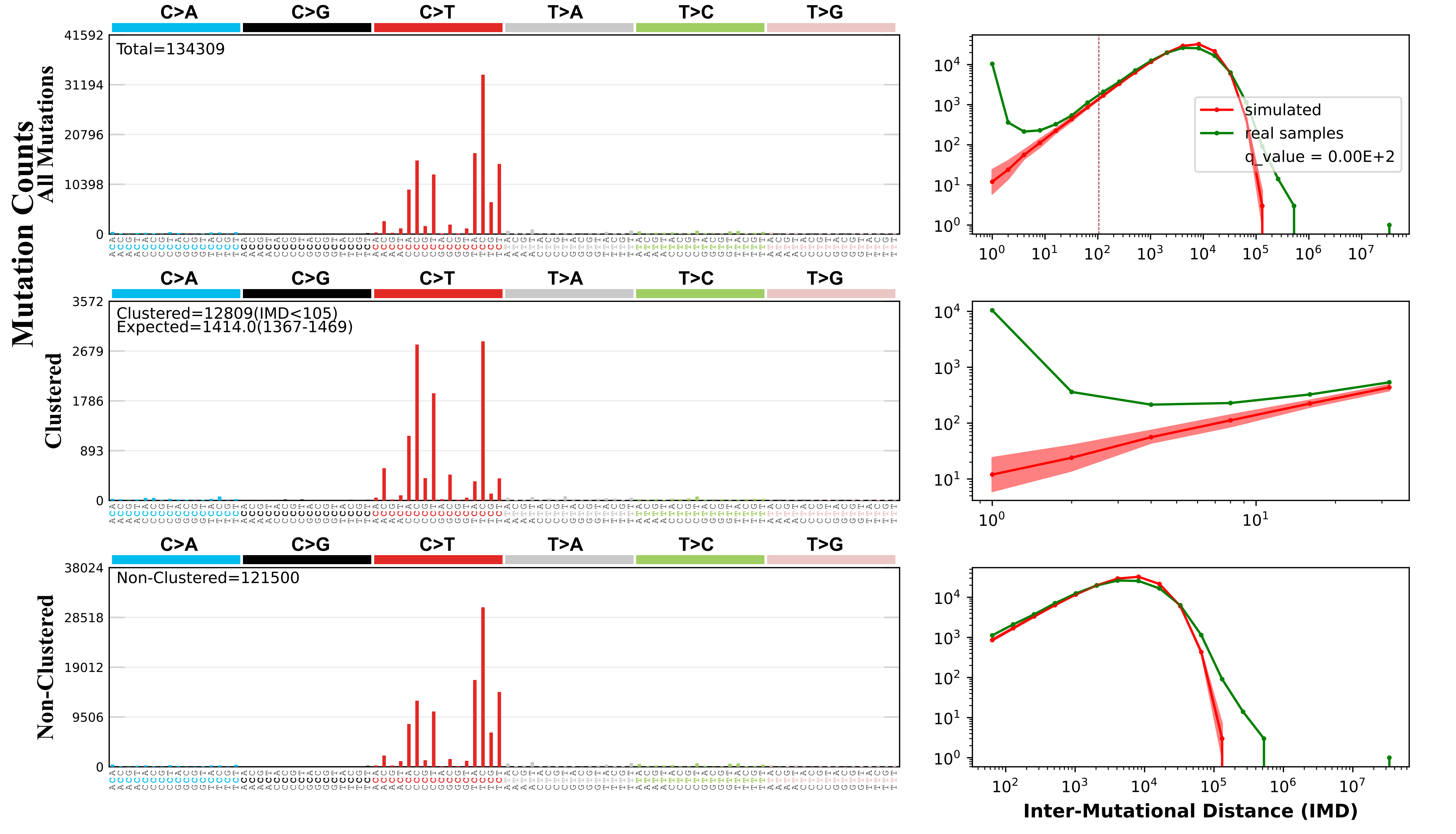

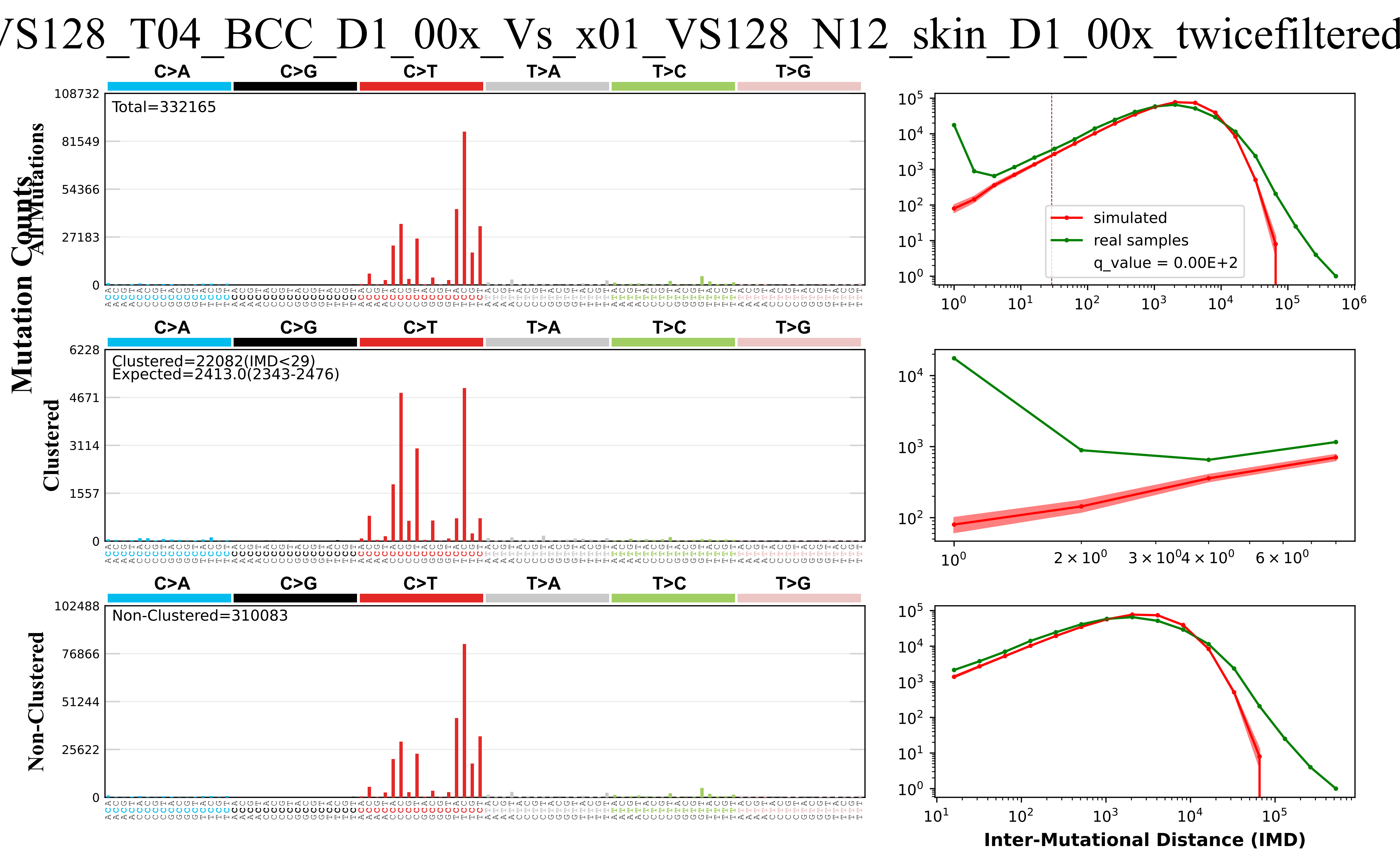
/S128_T04_BCC_D1_00x_Vs_x01_VS128_N12_skin_D1_00x_twicefiltered
Mutation Counts
All Mutations
C>A
C>G
C>T
T>A
T>C
T>G
Total=332165
108732
81549
54366
27183
0
simulated
real samples
q_value = 0.00E+2
Clustered
Clustered=22082(IMD<29)
Expected=2413.0(2343-2476)
6228
4671
3114
1557
0
Non-Clustered
Non-Clustered=310083
102488
76866
51244
25622
0
Inter-Mutational Distance (IMD)

SRR2194555_Vs_SRR2194563_twicefiltered_TvN

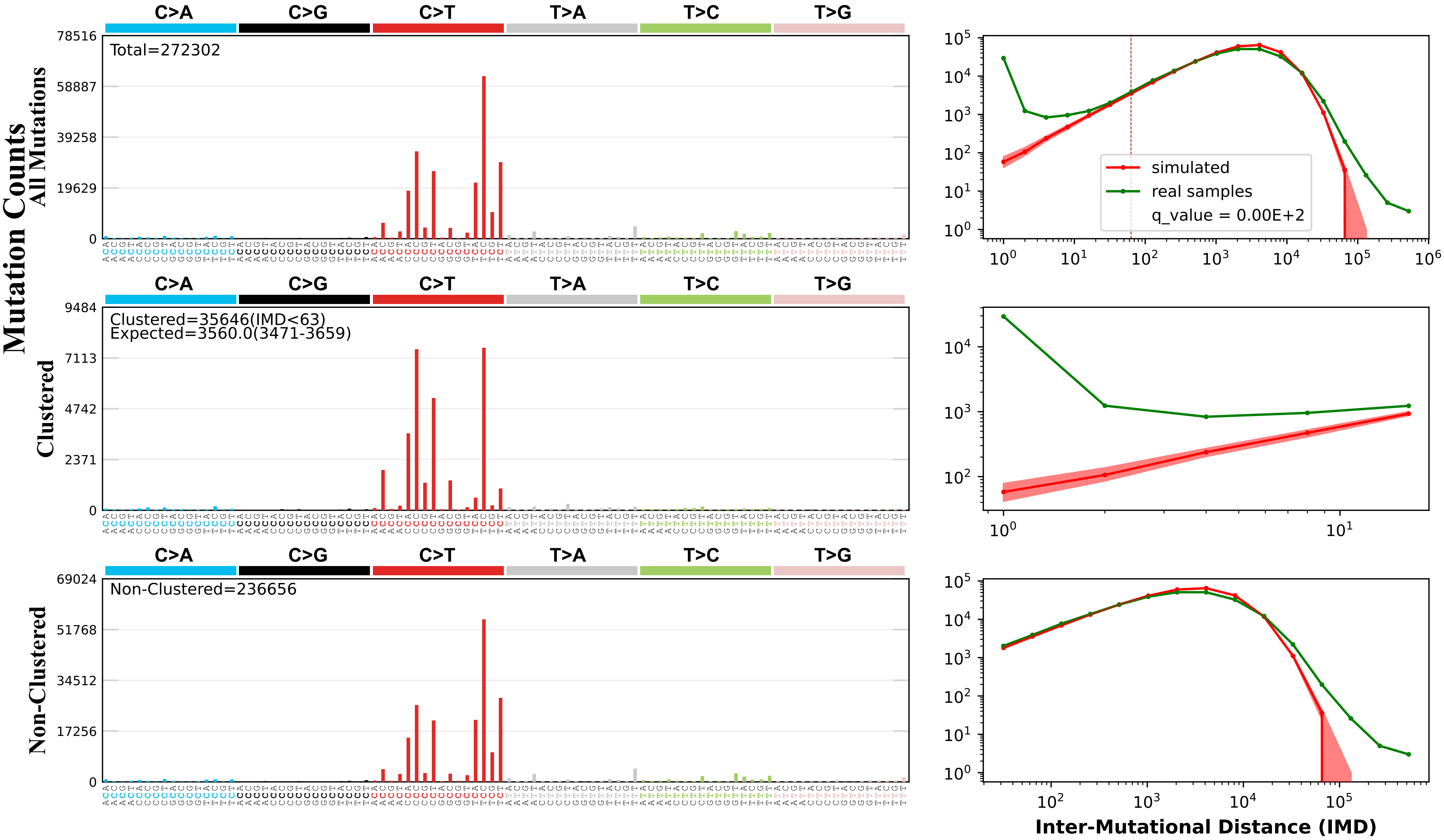

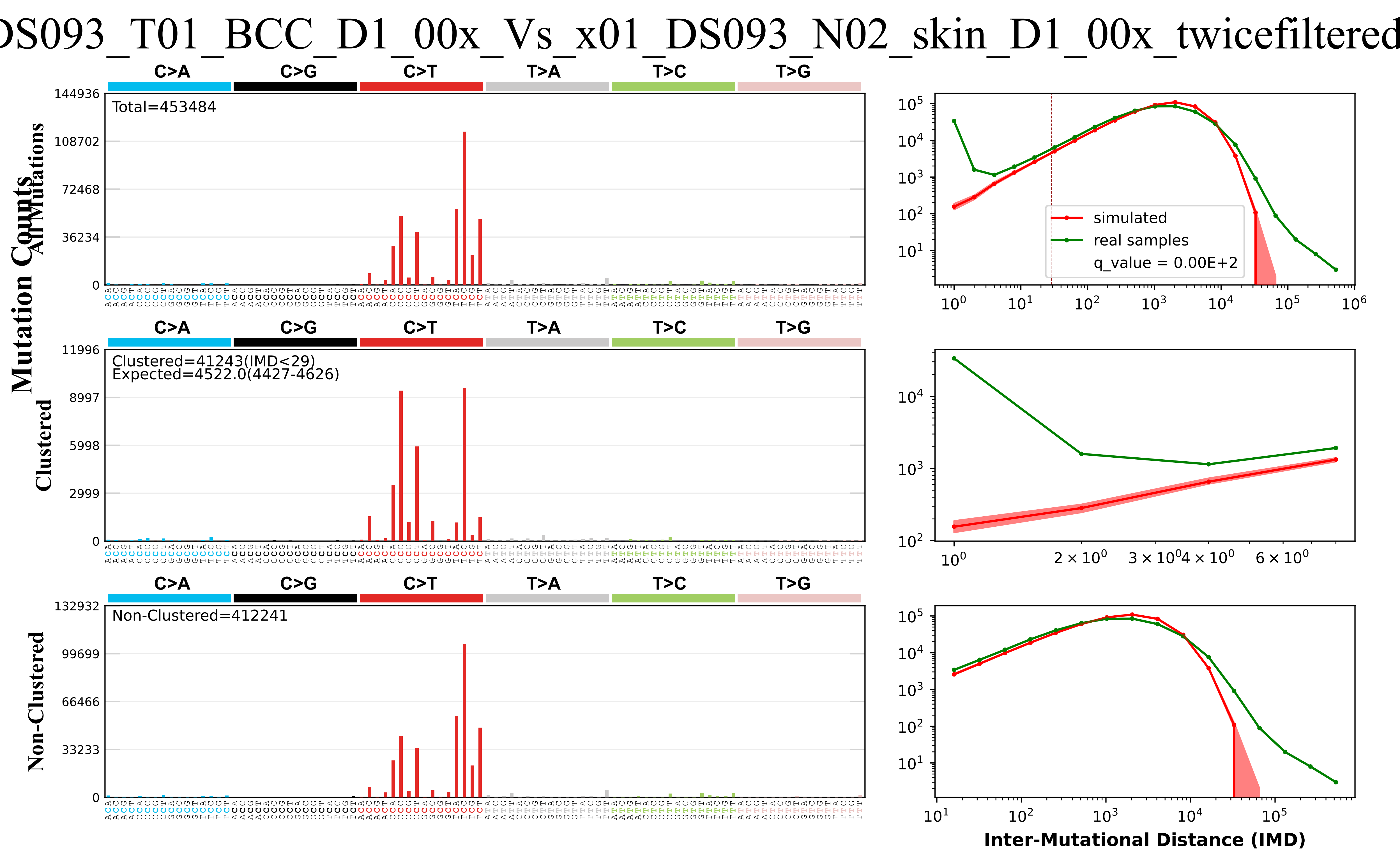

DS093_T01_BCC_D1_00x_Vs_x01_DS093_N02_skin_D1_00x_twicefiltered
Mutation Counts
All Mutations
Clustered
Non-Clustered
C>A
C>G
C>T
T>A
T>C
T>G
Total=453484
144936
108702
72468
36234
0
Clustered=41243(IMD<29)
Expected=4522.0(4427-4626)
11996
8997
5998
2999
Non-Clustered=412241
132932
99699
66466
33233
simulated
real samples
q_value = 0.00E+2
Inter-Mutational Distance (IMD)

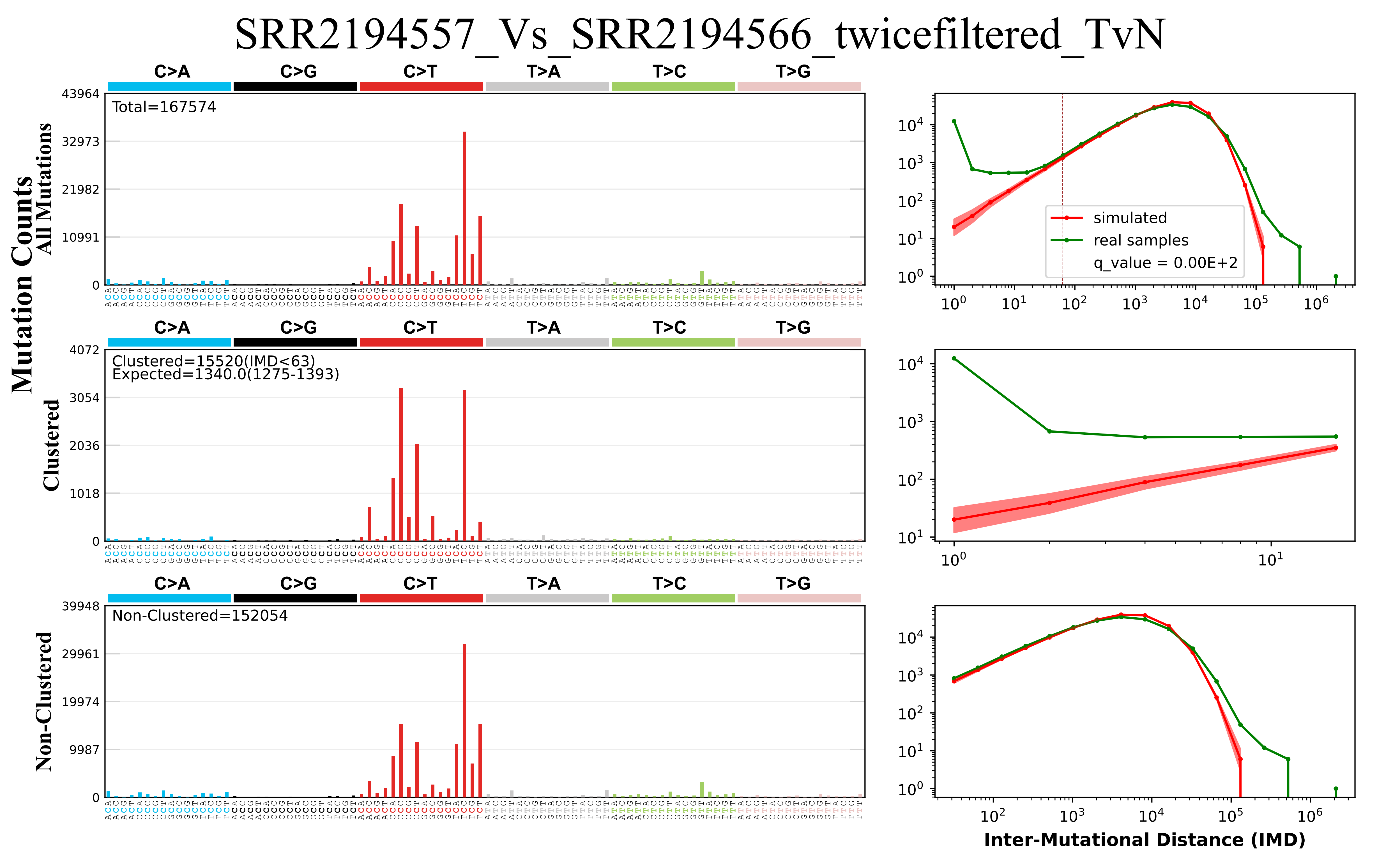
SRR2194557_Vs_SRR2194566_twicefiltered_TvN
Mutation Counts
All Mutations
Clustered
Non-Clustered
C>A
C>G
C>T
T>A
T>C
T>G
Total=167574
Clustered=15520(IMD<63)
Expected=1340.0(1275-1393)
Non-Clustered=152054
43964
32973
21982
10991
4072
3054
2036
1018
39948
29961
19974
9987
0
simulated
real samples
q_value = 0.00E+2
Inter-Mutational Distance (IMD)

VS191_T01_BCC_D1_00x_Vs_x01_VS191_N02_skin_D1_00x_twicefiltered

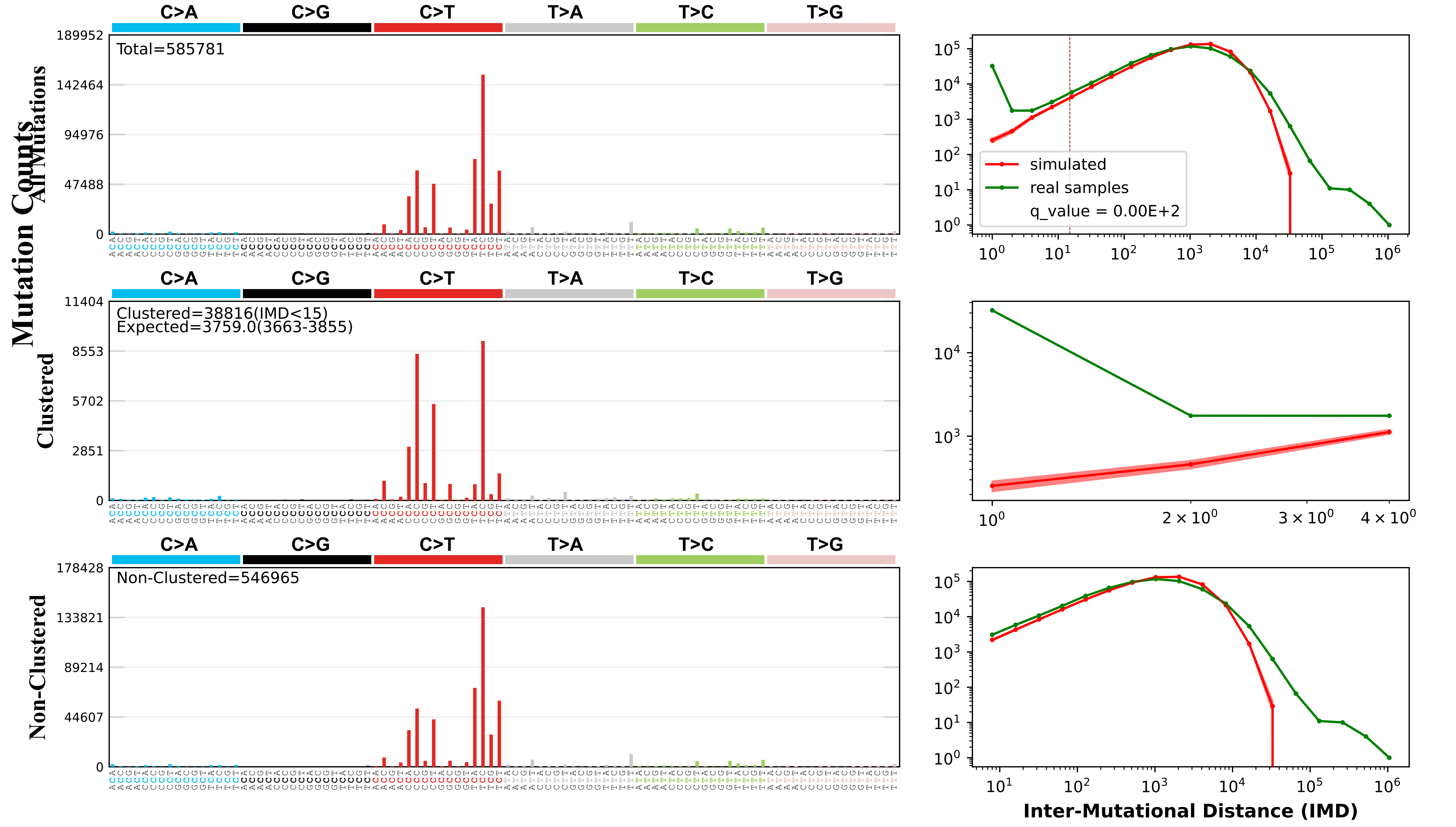

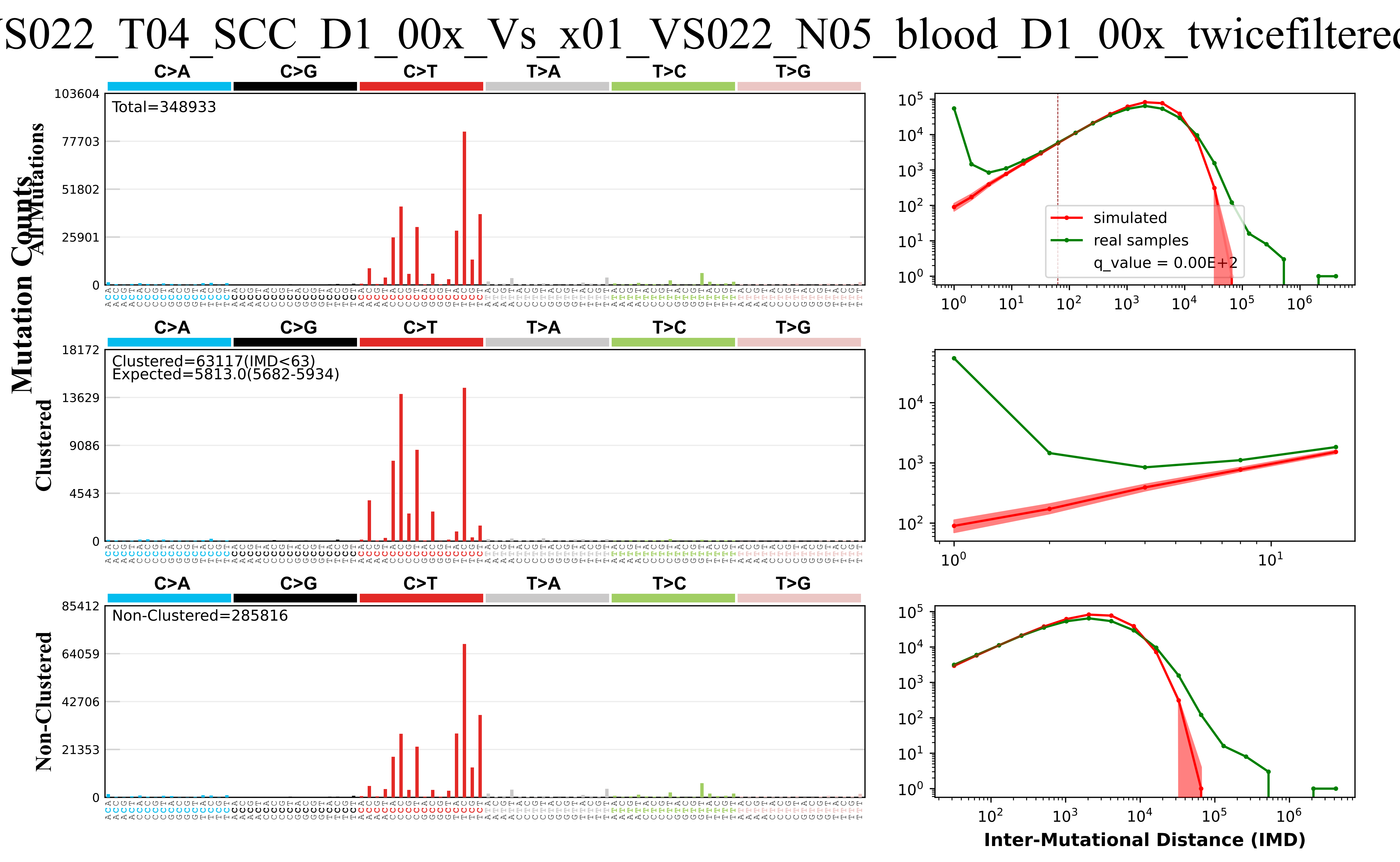

S022_T04_SCC_D1_00x_Vs_x01_VS022_N05_blood_D1_00x_twicefiltere
Mutation Counts
All Mutations
Clustered
Non-Clustered
C>A
C>G
C>T
T>A
T>C
T>G
Total=348933
Clustered=63117(IMD<63)
Expected=5813.0(5682-5934)
Non-Clustered=285816
103604
77703
51802
25901
18172
13629
9086
4543
85412
64059
42706
21353
0
simulated
real samples
q_value = 0.00E+2
Inter-Mutational Distance (IMD)

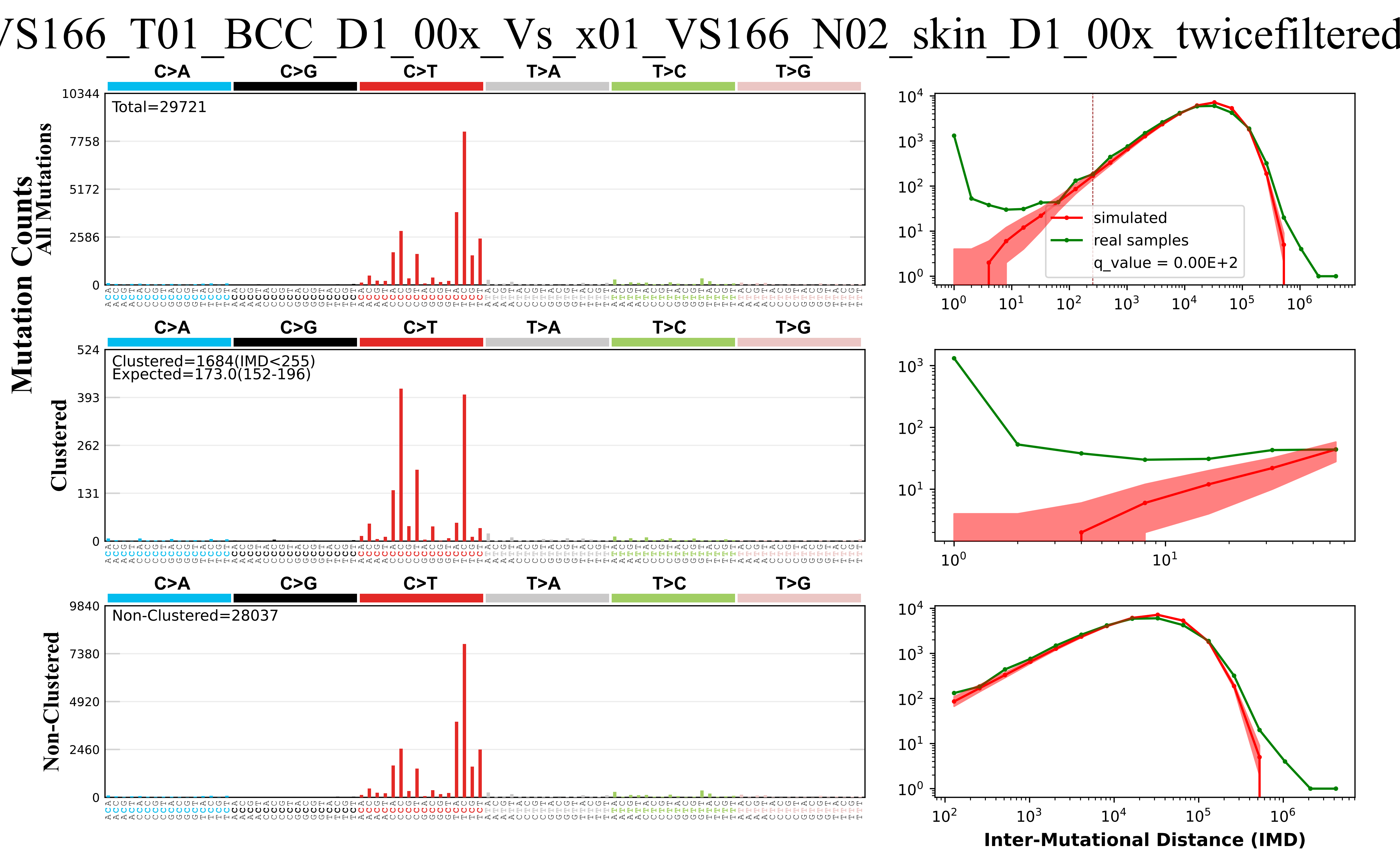

VS166_T01_BCC_D1_00x_Vs_x01_VS166_N02_skin_D1_00x_twicefiltered
Mutation Counts
All Mutations
Total=29721
Clustered
Clustered=1684(IMD<255)
Expected=173.0(152-196)
Non-Clustered
Non-Clustered=28037
C>A
C>G
C>T
T>A
T>C
T>G
simulated
real samples
q_value = 0.00E+2
Inter-Mutational Distance (IMD)

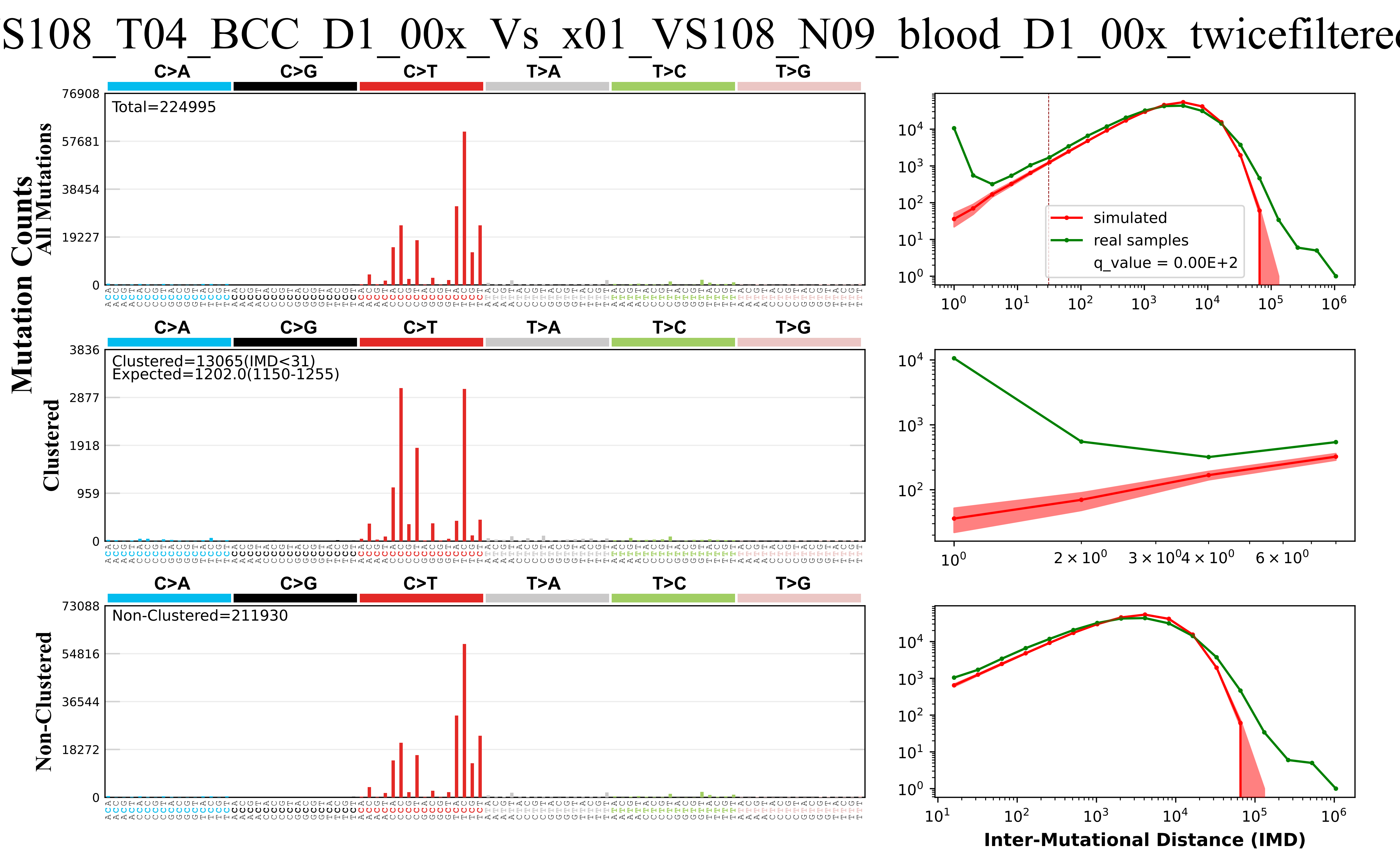

S108_T04_BCC_D1_00x_Vs_x01_VS108_N09_blood_D1_00x_twicefiltere
C>A
C>G
C>T
T>A
T>C
T>G
Mutation Counts
All Mutations
Total=224995
76908
57681
38454
19227
0
simulated
real samples
q_value = 0.00E+2
Clustered
Clustered=13065(IMD<31)
Expected=1202.0(1150-1255)
3836
2877
1918
959
0
Non-Clustered
Non-Clustered=211930
73088
54816
36544
18272
0
Inter-Mutational Distance (IMD)

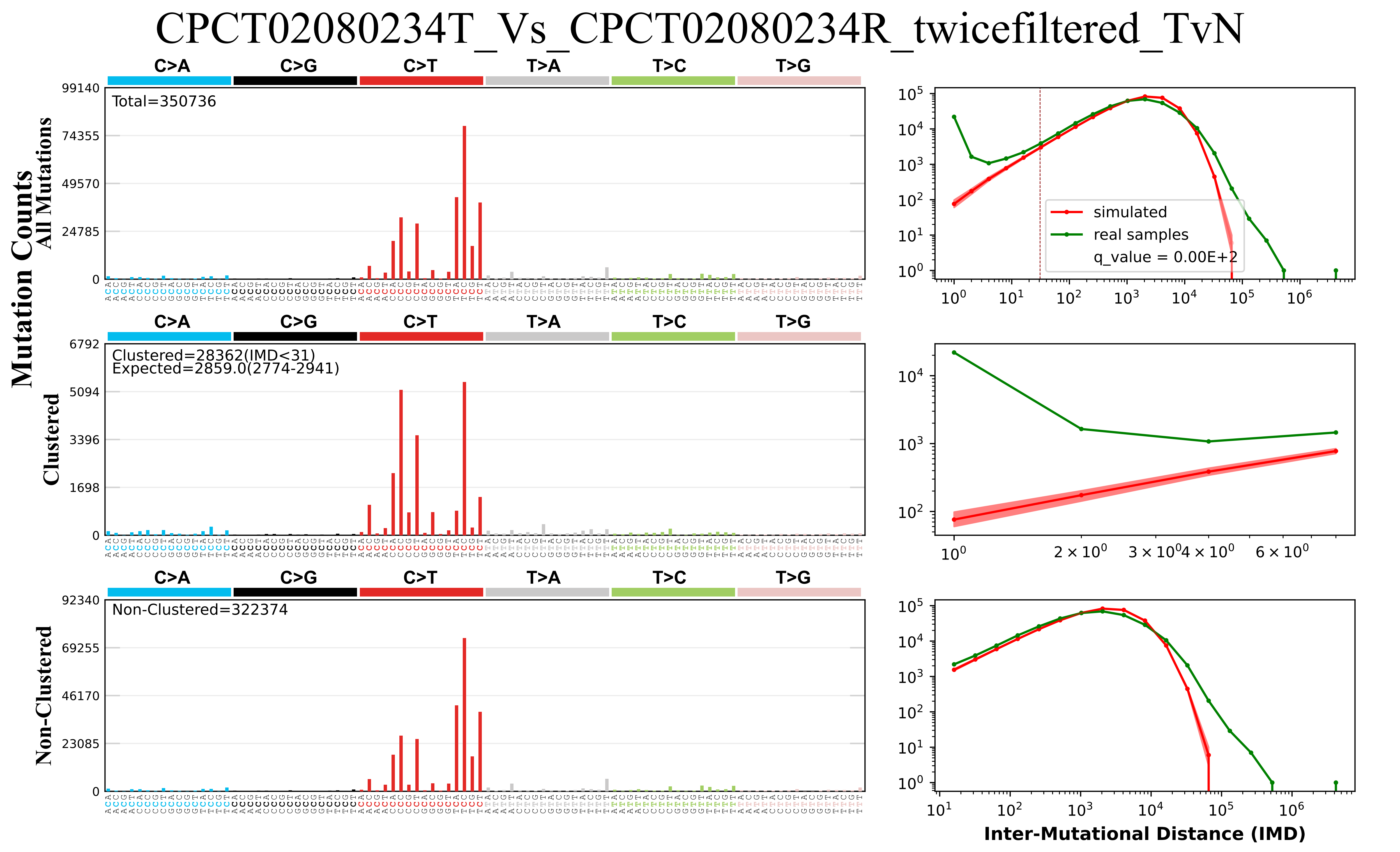

CPCT02080234T_Vs_CPCT02080234R_twicefiltered_TvN
C>A
C>G
C>T
T>A
T>C
T>G
Mutation Counts
All Mutations
Total=350736
99140
74355
49570
24785
0
Clustered
Clustered=28362(IMD<31)
Expected=2859.0(2774-2941)
6792
5094
3396
1698
0
Non-Clustered
Non-Clustered=322374
92340
69255
46170
23085
0
simulated
real samples
q_value = 0.00E+2
Inter-Mutational Distance (IMD)

DRUP01060009T_Vs_DRUP01060009R_twicefiltered_TvN

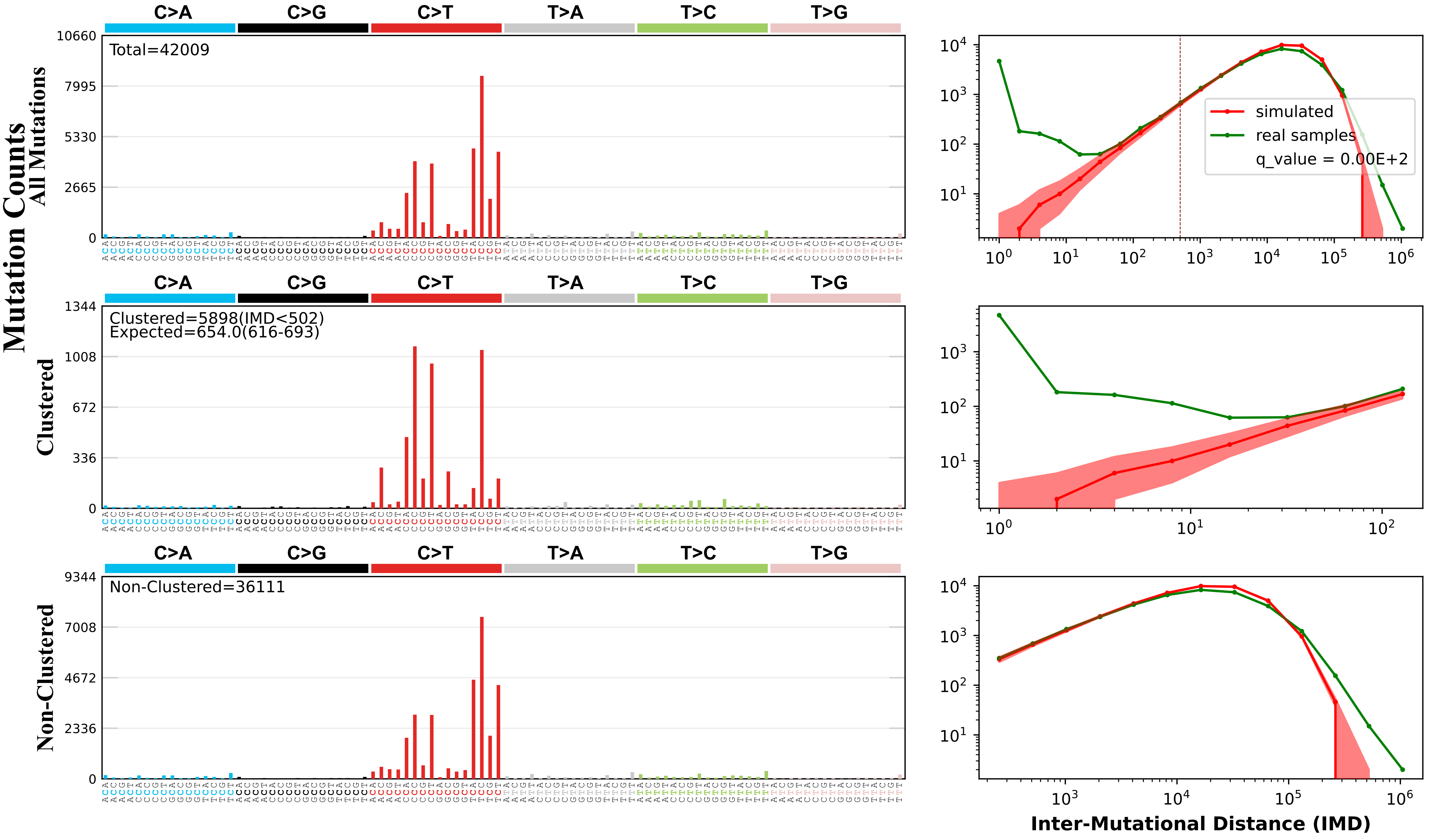

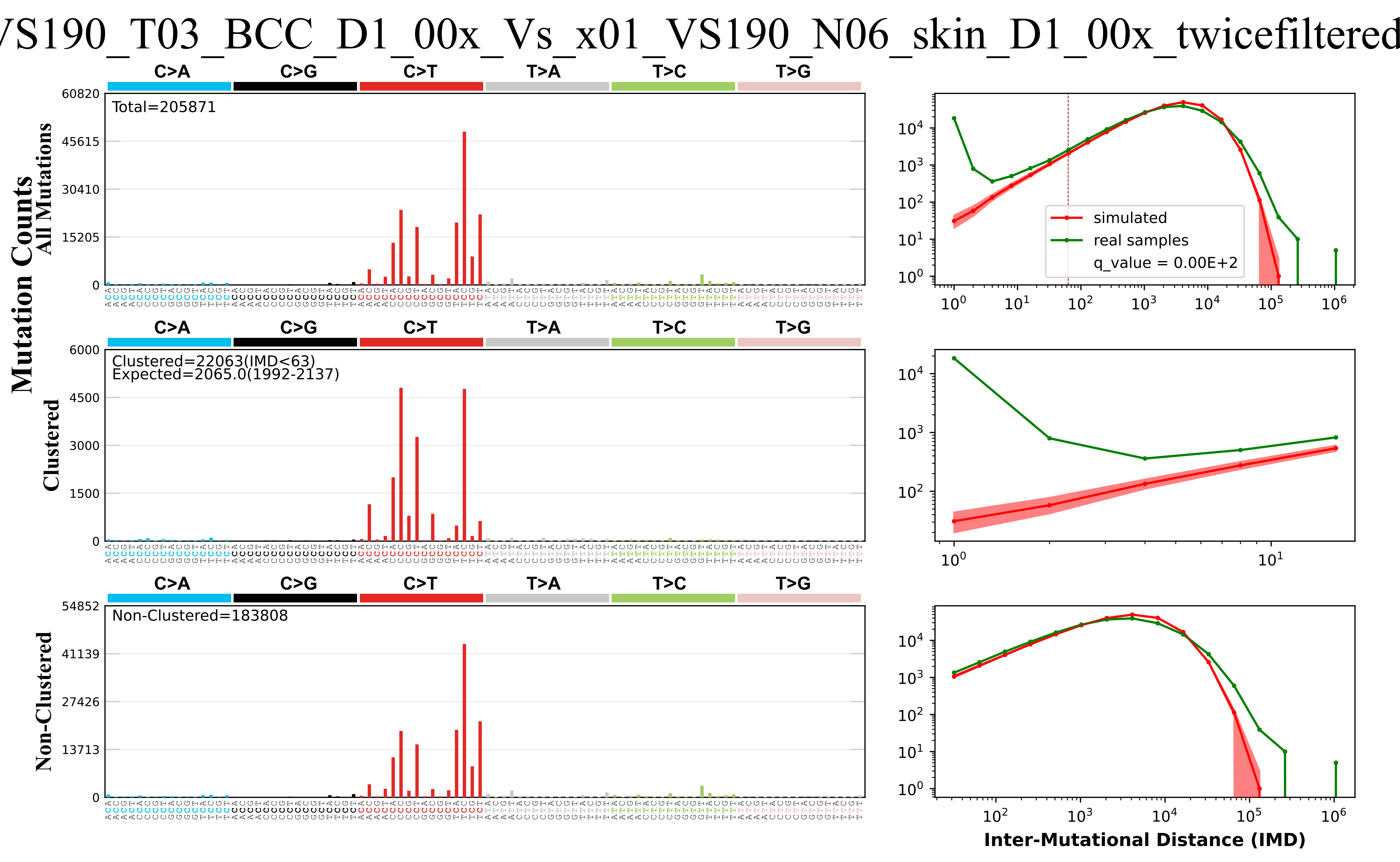

VS190_T03_BCC_D1_00x_Vs_x01_VS190_N06_skin_D1_00x_twicefiltered
Mutation Counts
All Mutations
Clustered
Non-Clustered
C>A
C>G
C>T
T>A
T>C
T>G
Total=205871
Clustered=22063(IMD<63)
Expected=2065.0(1992-2137)
Non-Clustered=183808
simulated
real samples
q_value = 0.00E+2
Inter-Mutational Distance (IMD)

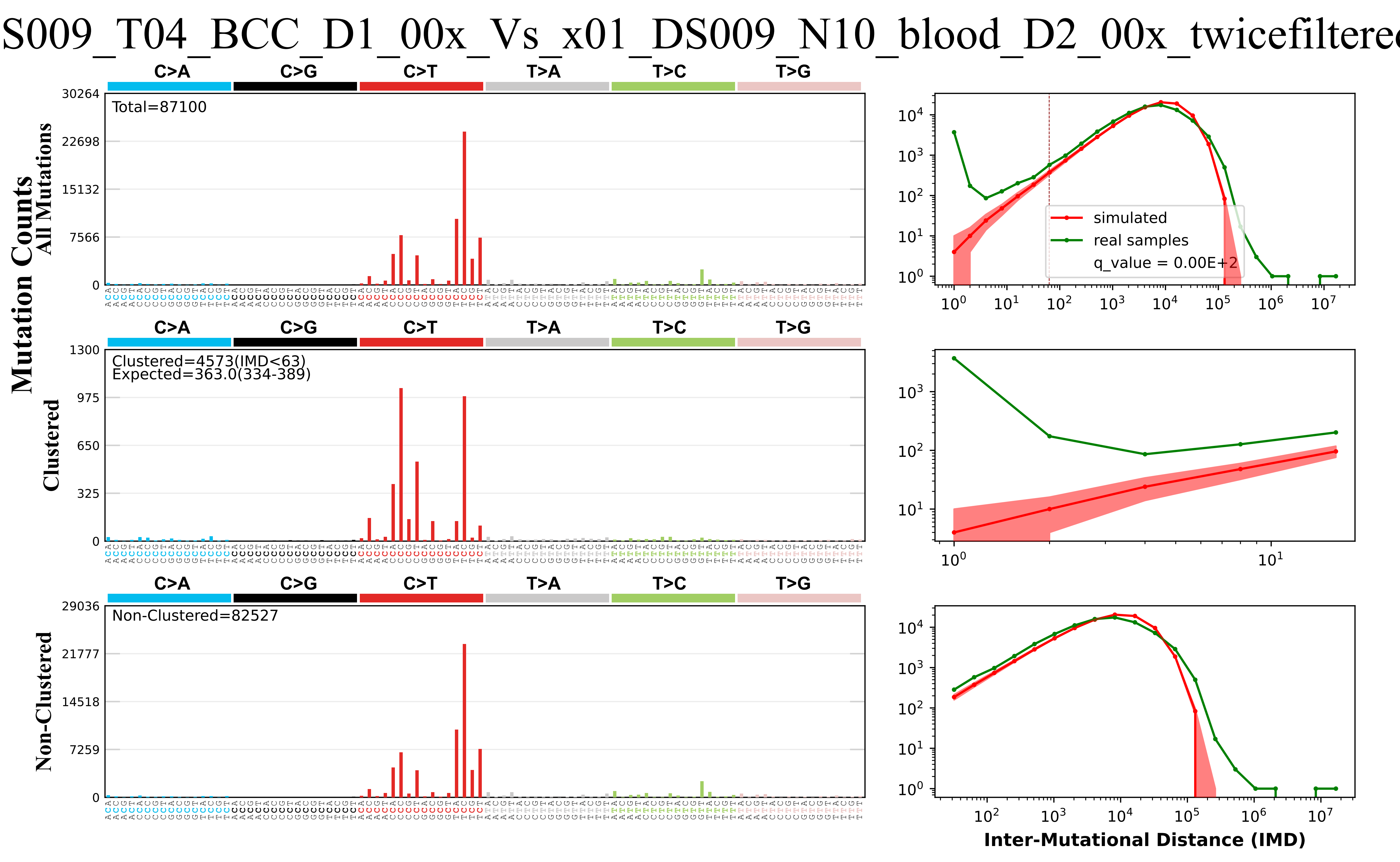

S009_T04_BCC_D1_00x_Vs_x01_DS009_N10_blood_D2_00x_twicefiltere
Mutation Counts
All Mutations
Clustered
Non-Clustered
C>A
C>G
C>T
T>A
T>C
T>G
Total=87100
Clustered=4573(IMD<63)
Expected=363.0(334-389)
Non-Clustered=82527
simulated
real samples
q_value = 0.00E+2
Inter-Mutational Distance (IMD)

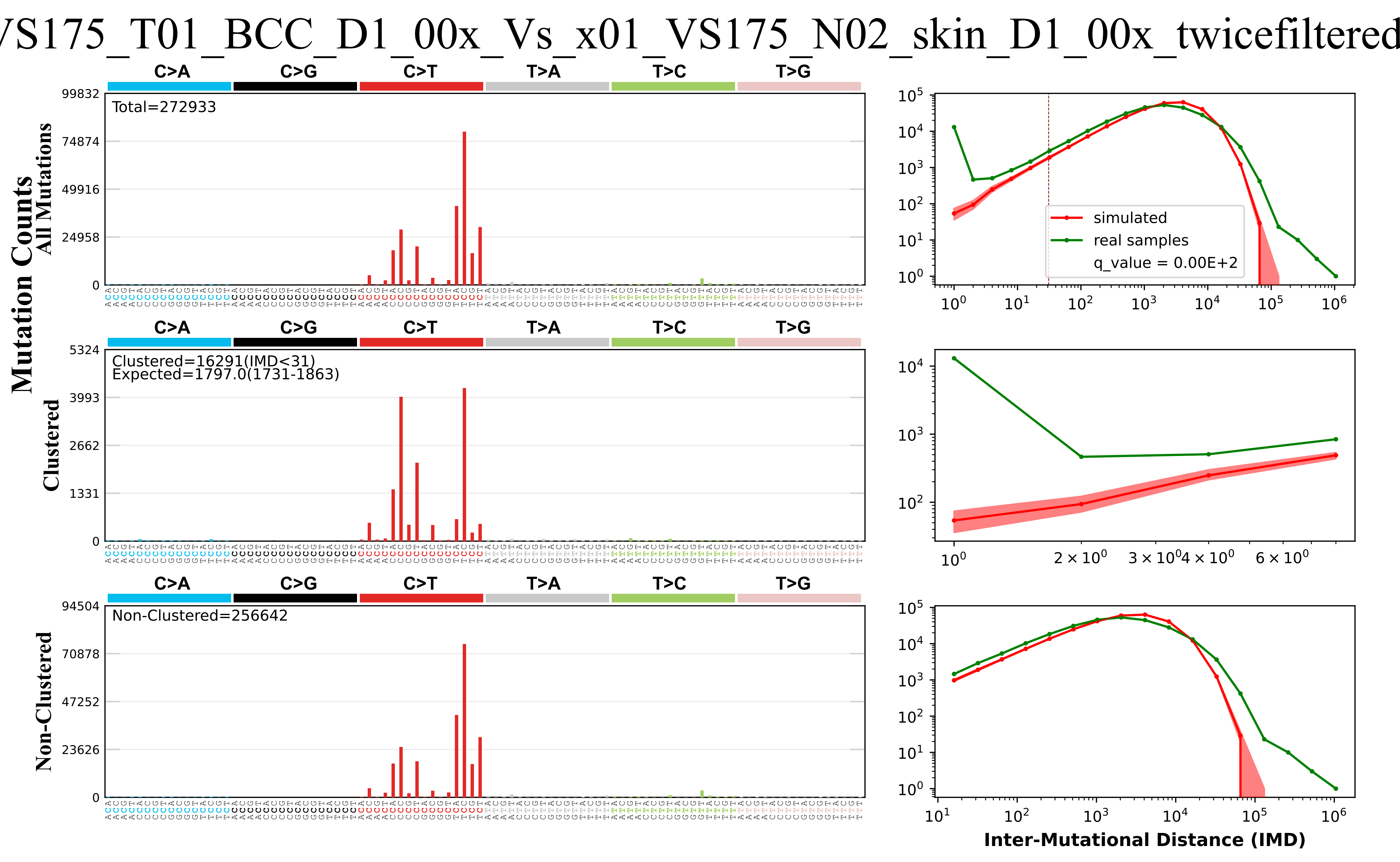

VS175_T01_BCC_D1_00x_Vs_x01_VS175_N02_skin_D1_00x_twicefiltered
Mutation Counts
All Mutations
Total=272933
C>A
C>G
C>T
T>A
T>C
T>G
Clustered
Clustered=16291(IMD<31)
Expected=1797.0(1731-1863)
Non-Clustered
Non-Clustered=256642
simulated
real samples
q_value = 0.00E+2
Inter-Mutational Distance (IMD)

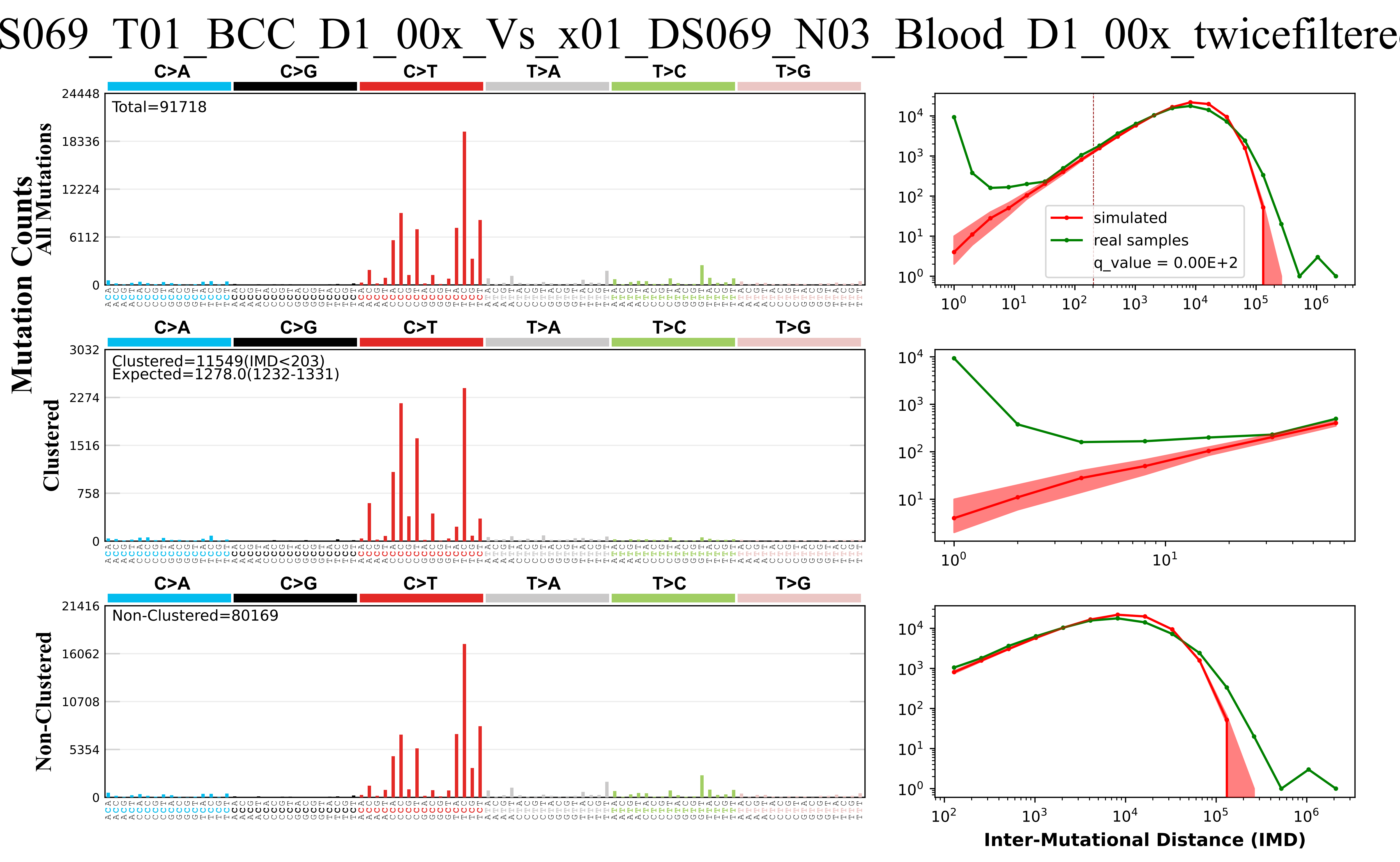

S069_T01_BCC_D1_00x_Vs_x01_DS069_N03_Blood_D1_00x_twicefiltere
Mutation Counts
All Mutations
Total=91718
C>A
C>G
C>T
T>A
T>C
T>G
Clustered
Clustered=11549(IMD<203)
Expected=1278.0(1232-1331)
Non-Clustered
Non-Clustered=80169
simulated
real samples
q_value = 0.00E+2
Inter-Mutational Distance (IMD)

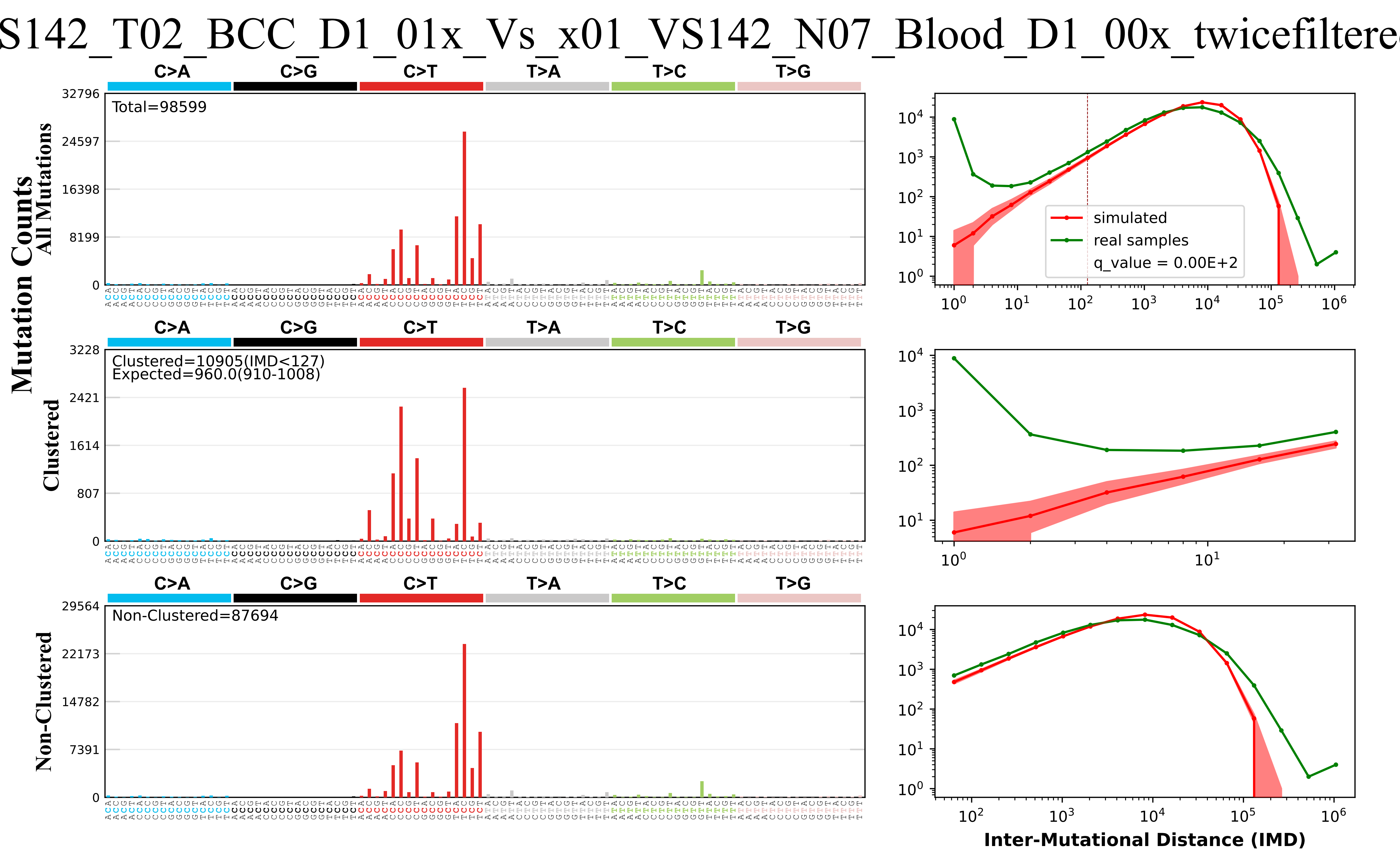
S142_T02_BCC_D1_01x_Vs_x01_VS142_N07_Blood_D1_00x_twicefiltere
Mutation Counts
All Mutations
Clustered
Non-Clustered
C>A
C>G
C>T
T>A
T>C
T>G
Total=98599
Clustered=10905(IMD<127)
Expected=960.0(910-1008)
Non-Clustered=87694
simulated
real samples
q_value = 0.00E+2
Inter-Mutational Distance (IMD)

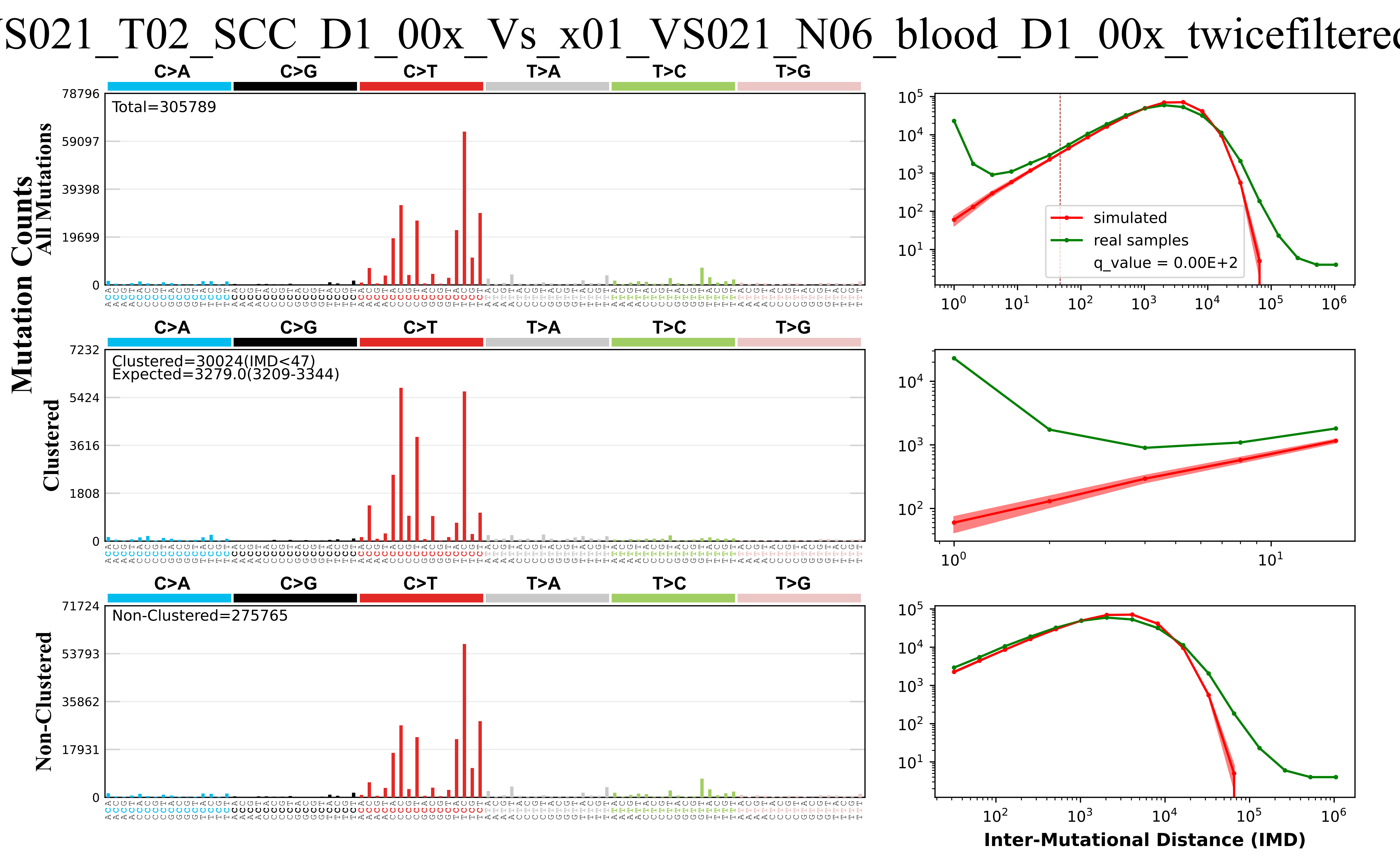

VS021_T02_SCC_D1_00x_Vs_x01_VS021_N06_blood_D1_00x_twicefiltered
C>A
C>G
C>T
T>A
T>C
T>G
Mutation Counts
All Mutations
Total=305789
Clustered
Clustered=30024(IMD<47)
Expected=3279.0(3209-3344)
Non-Clustered
Non-Clustered=275765
simulated
real samples
q_value = 0.00E+2
Inter-Mutational Distance (IMD)

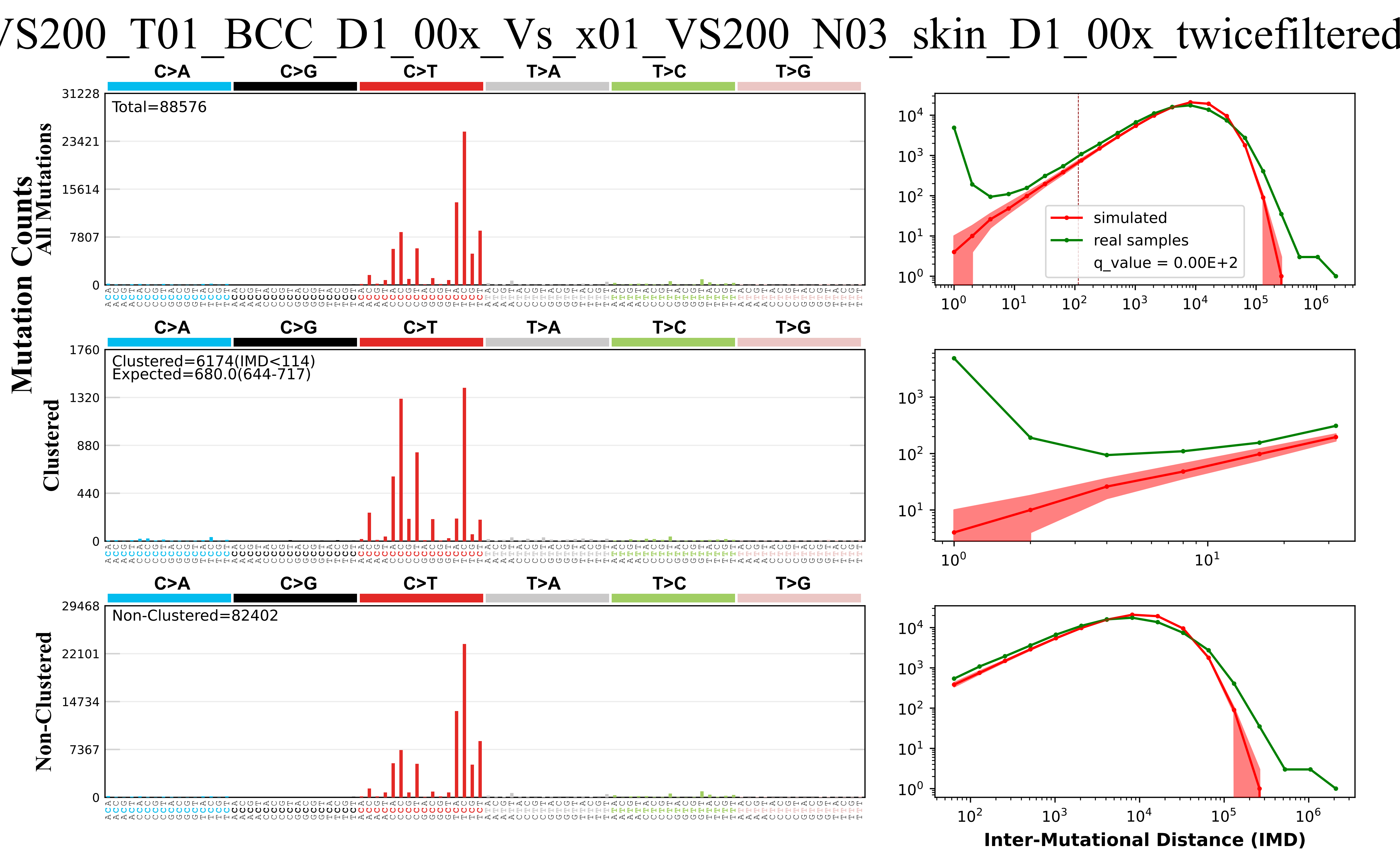
VS200_T01_BCC_D1_00x_Vs_x01_VS200_N03_skin_D1_00x_twicefiltered
Mutation Counts
All Mutations
Clustered
Non-Clustered
C>A
C>G
C>T
T>A
T>C
T>G
Total=88576
Clustered=6174(IMD<114)
Expected=680.0(644-717)
Non-Clustered=82402
31228
23421
15614
7807
0
1760
1320
880
440
29468
22101
14734
7367
simulated
real samples
q_value = 0.00E+2
Inter-Mutational Distance (IMD)

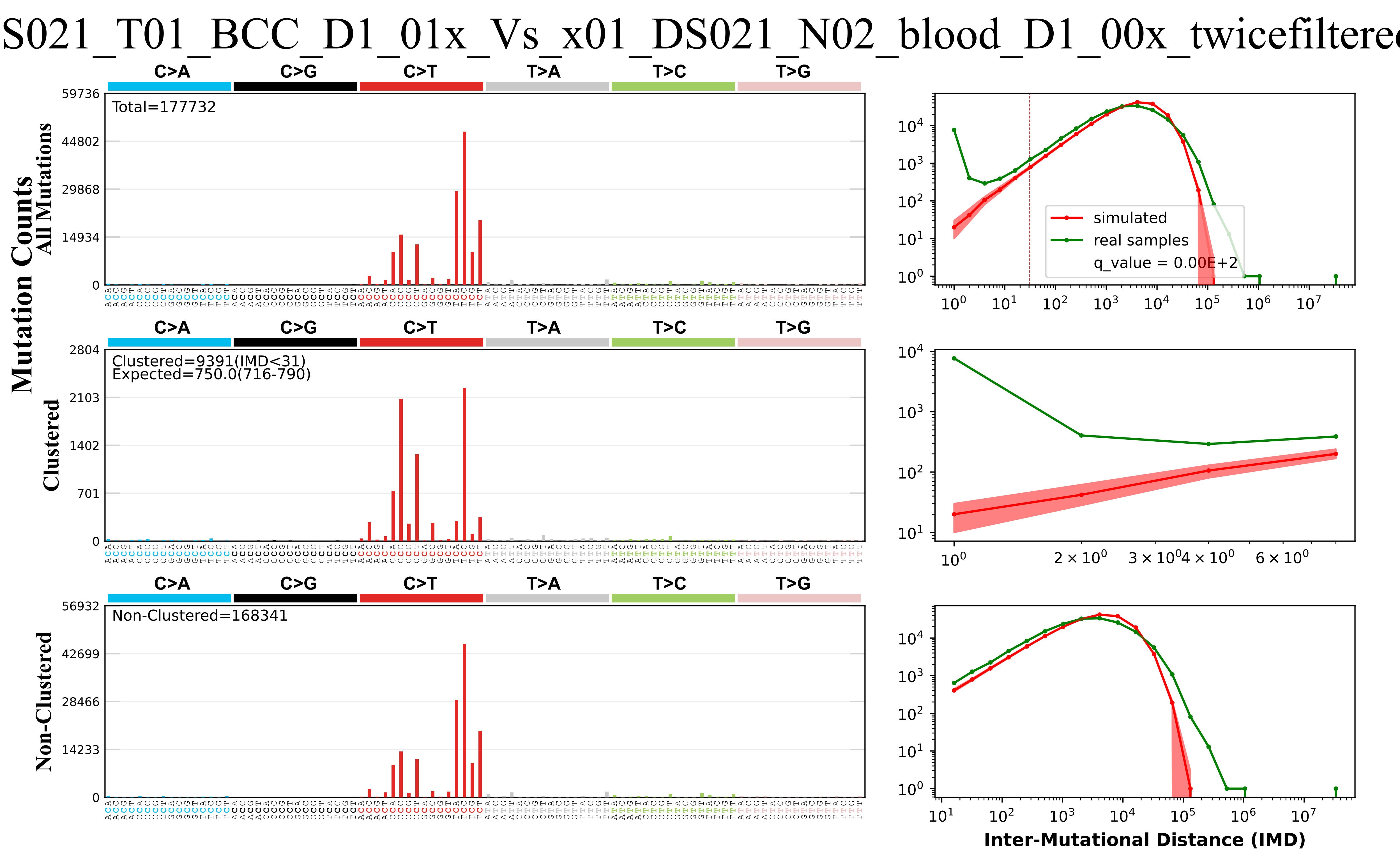
S021_T01_BCC_D1_01x_Vs_x01_DS021_N02_blood_D1_00x_twicefiltered
Mutation Counts
All Mutations
Clustered
Non-Clustered
C>A
C>G
C>T
T>A
T>C
T>G
Total=177732
Clustered=9391(IMD<31)
Expected=750.0(716-790)
Non-Clustered=168341
simulated
real samples
q_value = 0.00E+2
Inter-Mutational Distance (IMD)

SRR2194556_Vs_SRR2194565_twicefiltered_TvN

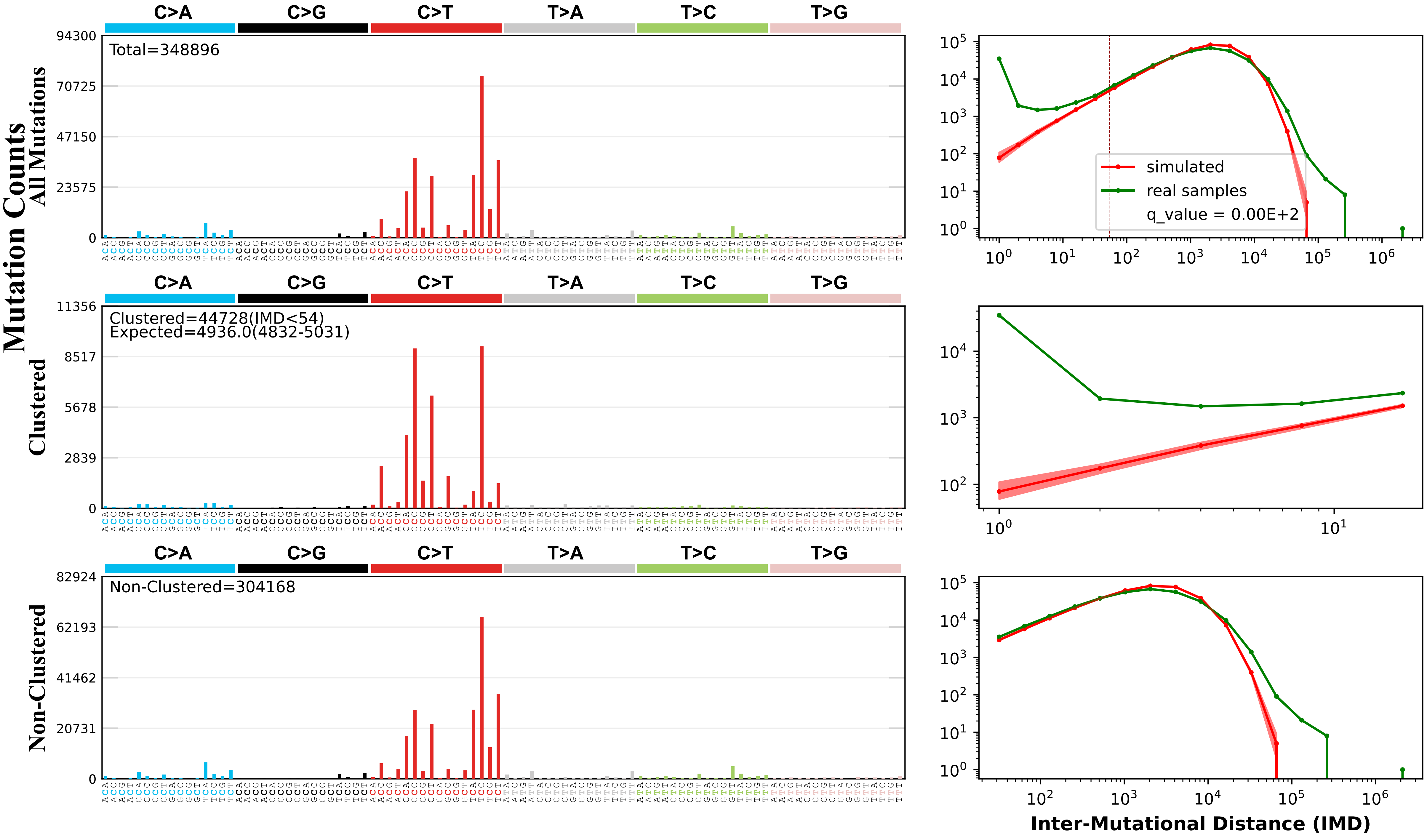

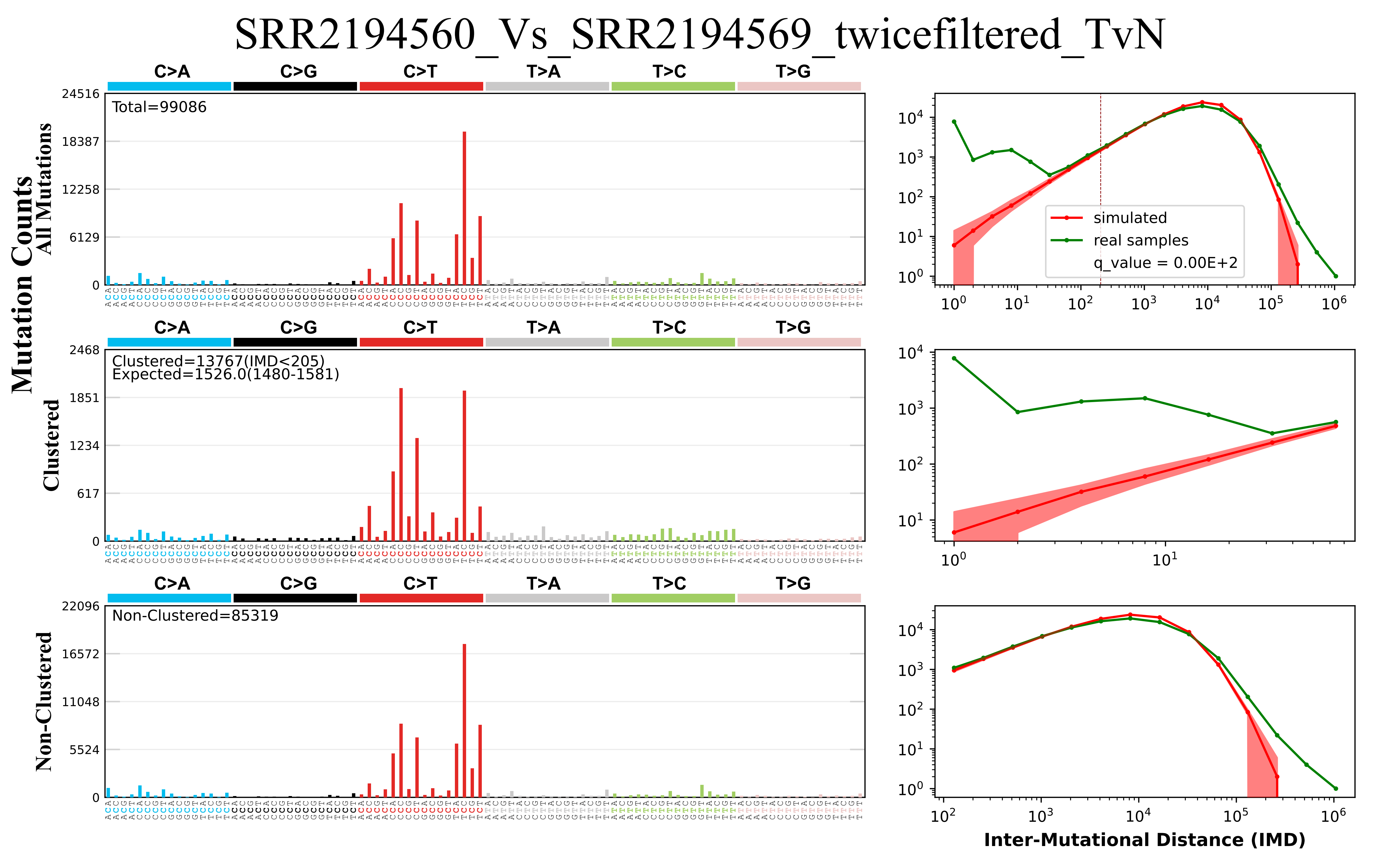
SRR2194560_Vs_SRR2194569_twicefiltered_TvN
C>A
C>G
C>T
T>A
T>C
T>G
Mutation Counts
All Mutations
Total=99086
24516
18387
12258
6129
0
Clustered
Clustered=13767(IMD<205)
Expected=1526.0(1480-1581)
2468
1851
1234
617
Non-Clustered
Non-Clustered=85319
22096
16572
11048
5524
simulated
real samples
q_value = 0.00E+2
Inter-Mutational Distance (IMD)

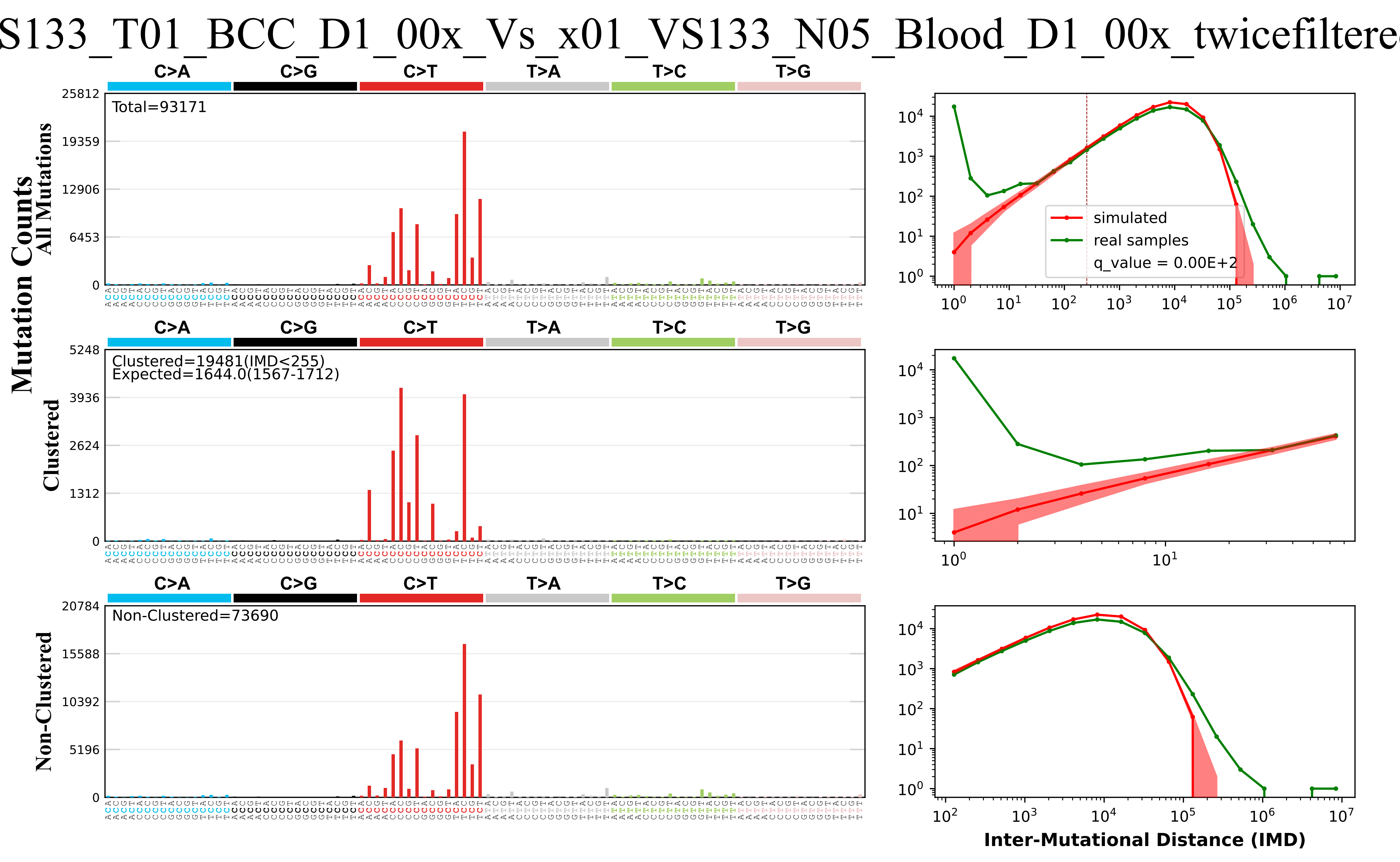
S133_T01_BCC_D1_00x_Vs_x01_VS133_N05_Blood_D1_00x_twicefiltere
Mutation Counts
All Mutations
Clustered
Non-Clustered
C>A
C>G
C>T
T>A
T>C
T>G
Total=93171
25812
19359
12906
6453
0
Clustered=19481(IMD<255)
Expected=1644.0(1567-1712)
5248
3936
2624
1312
0
Non-Clustered=73690
20784
15588
10392
5196
0
simulated
real samples
q_value = 0.00E+2
Inter-Mutational Distance (IMD)

CPCT02060041T_Vs_CPCT02060041R_twicefiltered_TvN

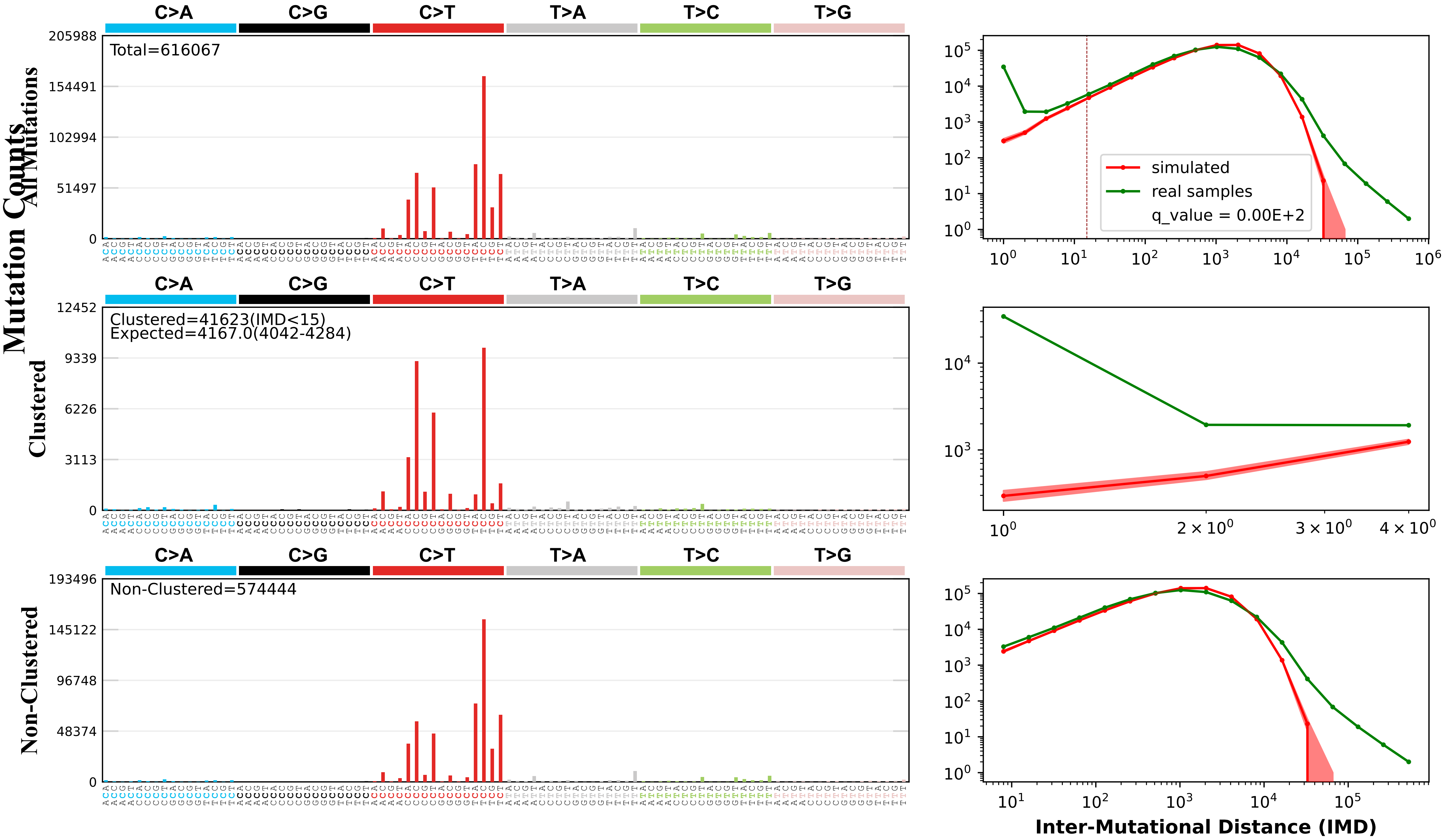

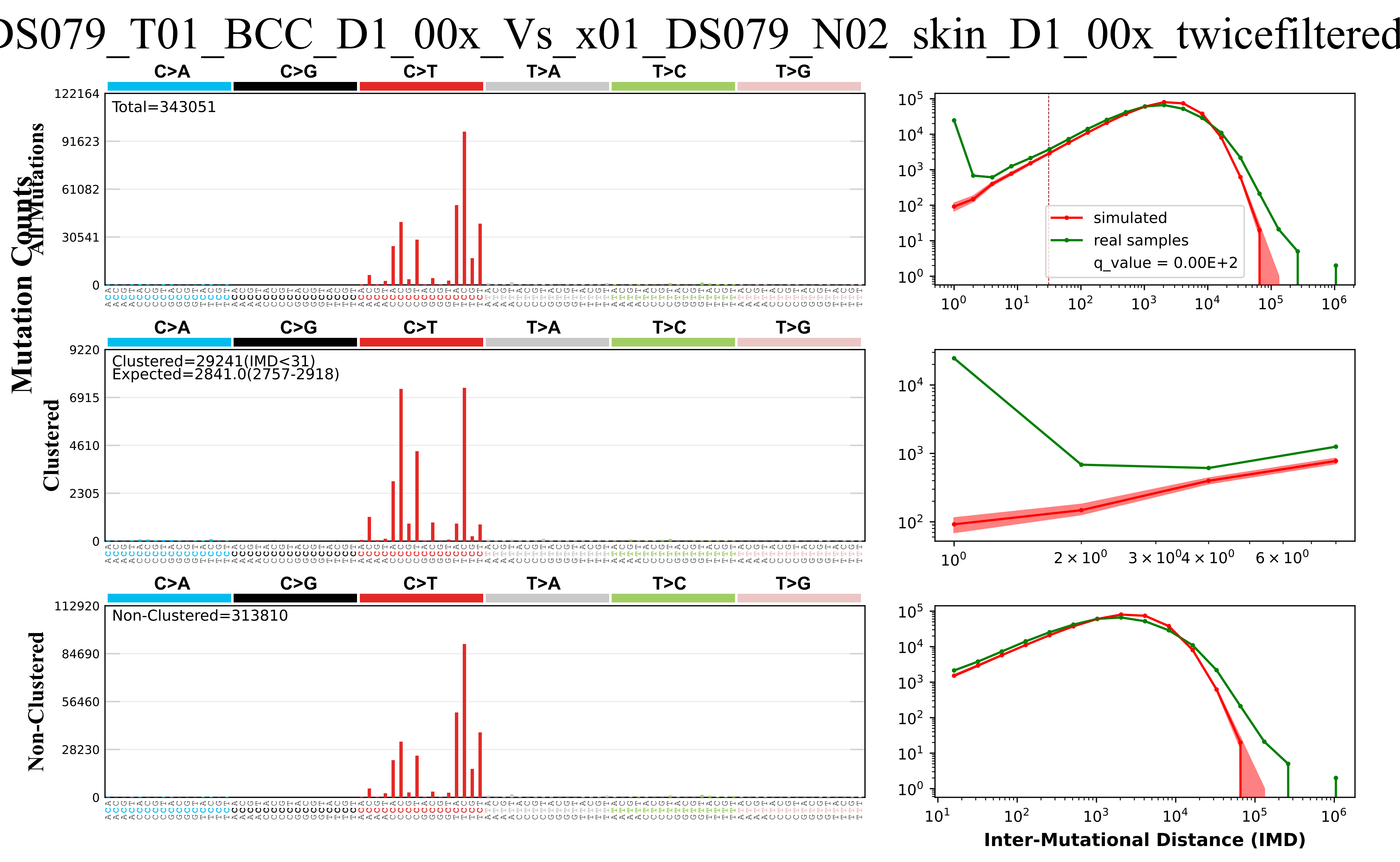
DS079_T01_BCC_D1_00x_Vs_x01_DS079_N02_skin_D1_00x_twicefiltered
Mutation Counts
All Mutations
Total=343051
122164
91623
61082
30541
0
C>A
C>G
C>T
T>A
T>C
T>G
simulated
real samples
q_value = 0.00E+2
Clustered
Clustered=29241(IMD<31)
Expected=2841.0(2757-2918)
9220
6915
4610
2305
Non-Clustered
Non-Clustered=313810
112920
84690
56460
28230
Inter-Mutational Distance (IMD)

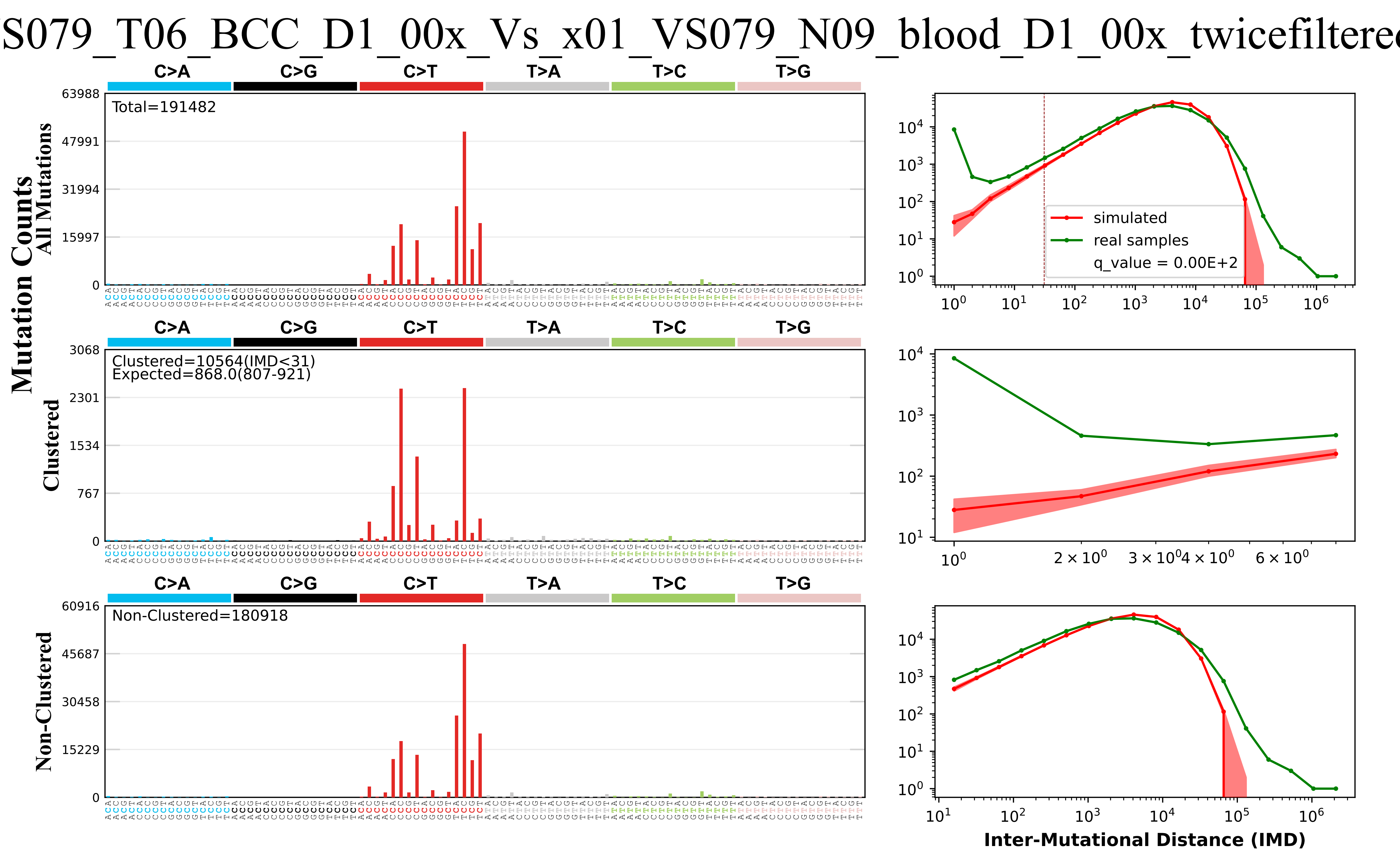
S079_T06_BCC_D1_00x_Vs_x01_VS079_N09_blood_D1_00x_twicefiltered
Mutation Counts
All Mutations
Clustered
Non-Clustered
C>A
C>G
C>T
T>A
T>C
T>G
Total=191482
Clustered=10564(IMD<31)
Expected=868.0(807-921)
Non-Clustered=180918
simulated
real samples
q_value = 0.00E+2
Inter-Mutational Distance (IMD)

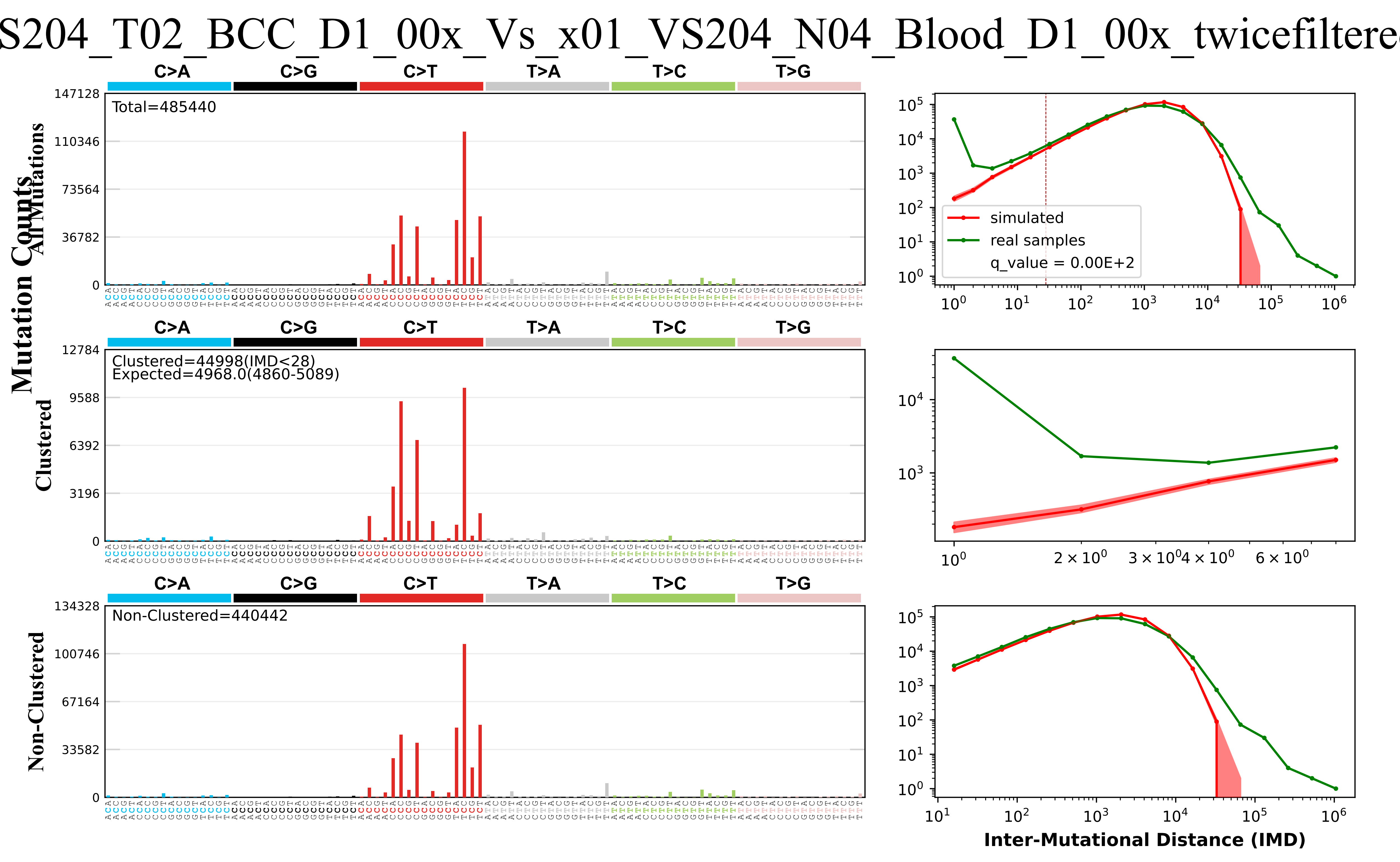
S204_T02_BCC_D1_00x_Vs_x01_VS204_N04_Blood_D1_00x_twicefiltere
Mutation Counts
All Mutations
C>A
C>G
C>T
T>A
T>C
T>G
Total=485440
147128
110346
73564
36782
0
Clustered
Clustered=44998(IMD<28)
Expected=4968.0(4860-5089)
12784
9588
6392
3196
0
Non-Clustered
Non-Clustered=440442
134328
100746
67164
33582
0
simulated
real samples
q_value = 0.00E+2
Inter-Mutational Distance (IMD)

S022_T03_BCC_D1_01x_Vs_x01_VS022_N05_blood_D1_00x_twicefiltered

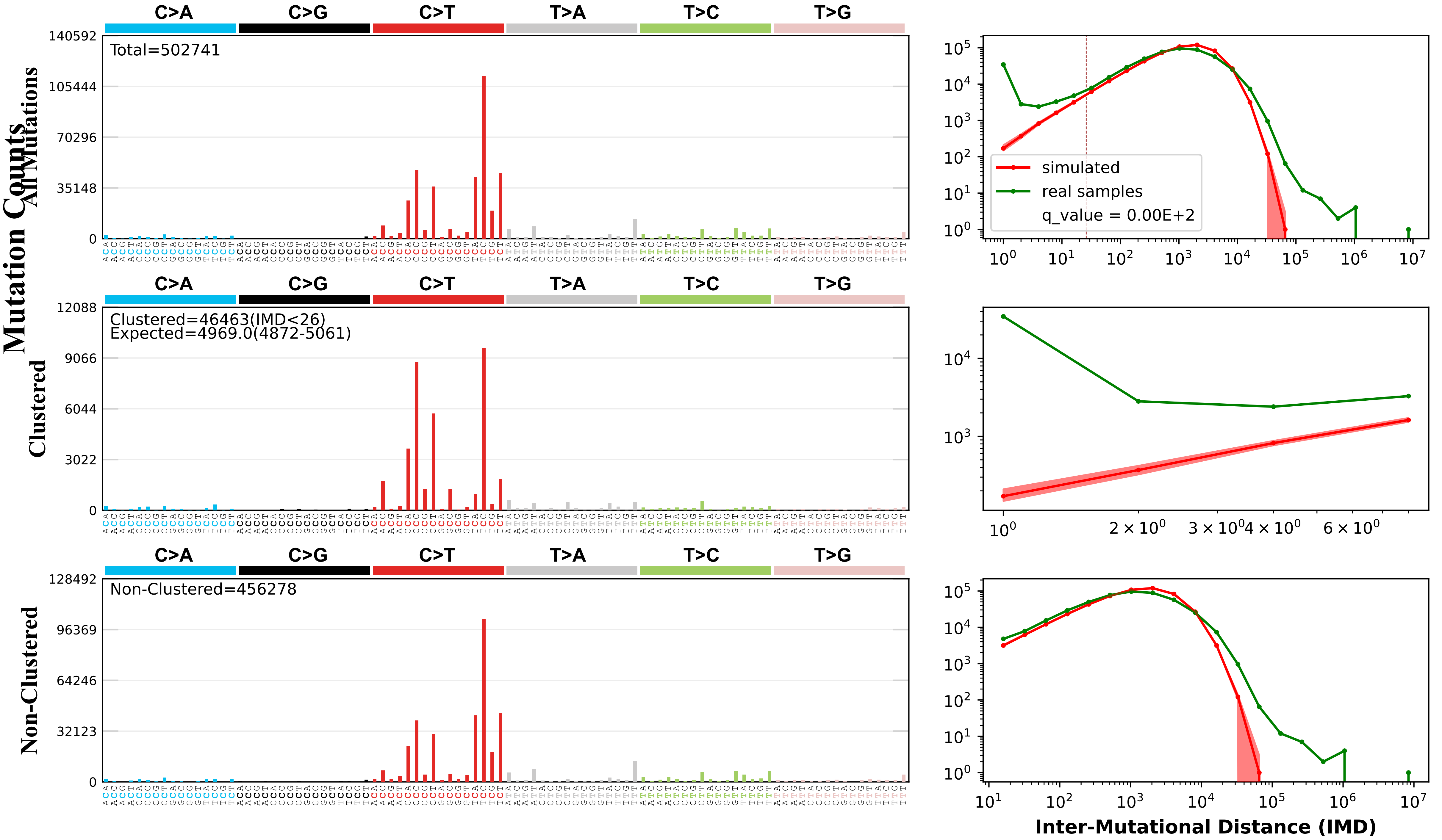

VS128_T05_BCC_D1_01x_Vs_x01_VS128_N12_skin_D1_00x_twicefiltered

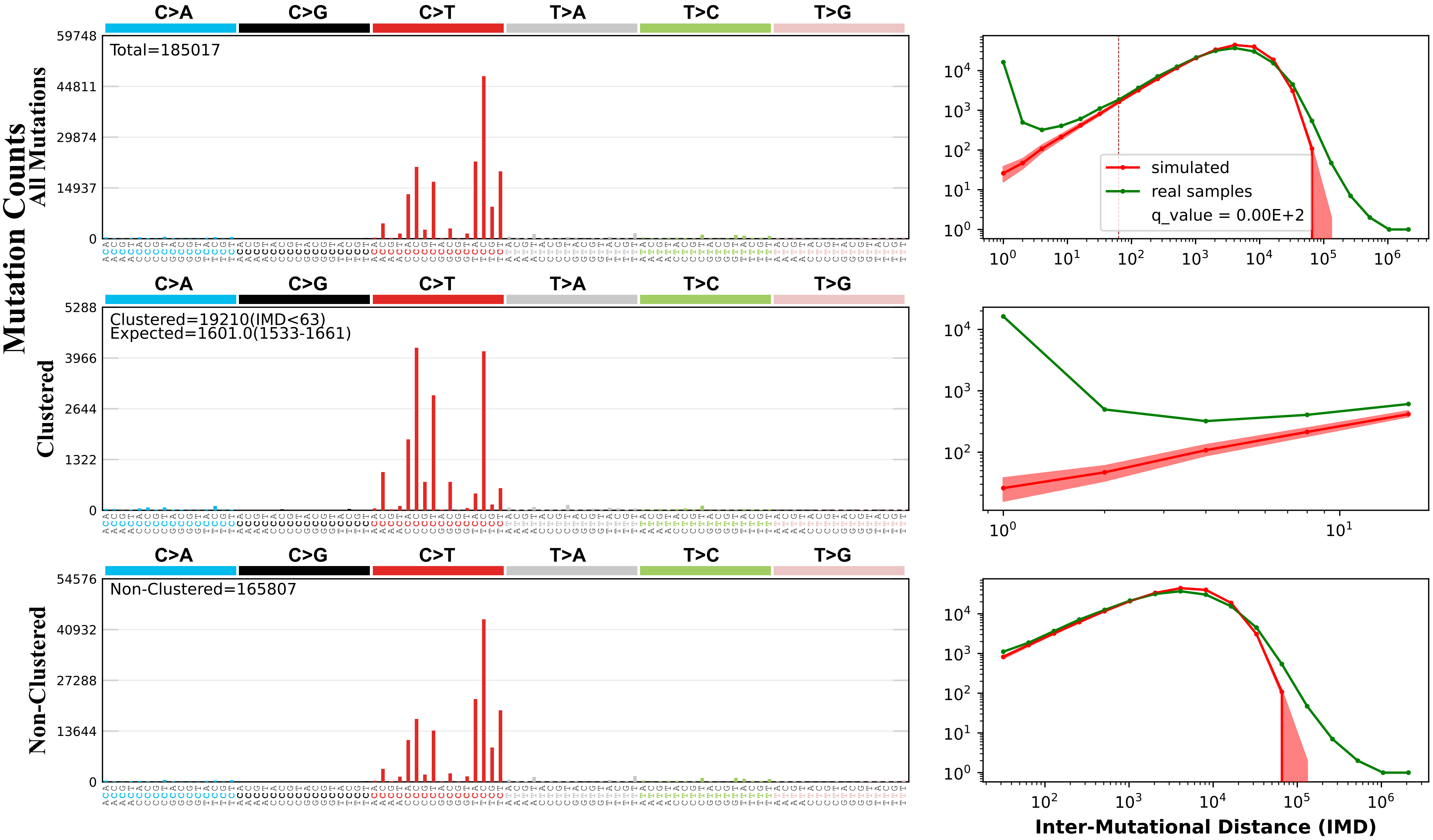

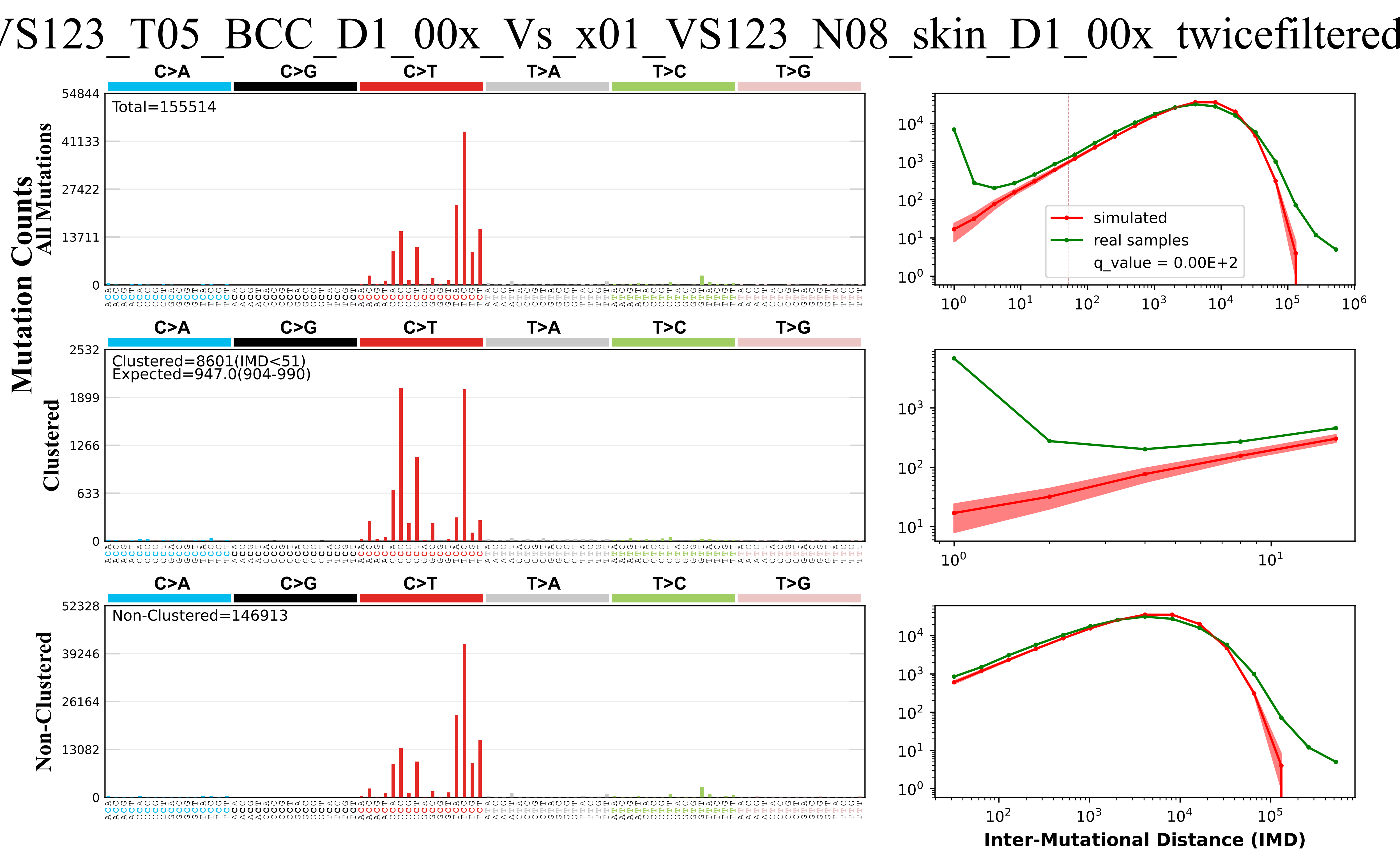
VS123_T05_BCC_D1_00x_Vs_x01_VS123_N08_skin_D1_00x_twicefiltered
Mutation Counts
All Mutations
Clustered
Non-Clustered
C>A
C>G
C>T
T>A
T>C
T>G
Total=155514
Clustered=8601(IMD<51)
Expected=947.0(904-990)
Non-Clustered=146913
simulated
real samples
q_value = 0.00E+2
Inter-Mutational Distance (IMD)

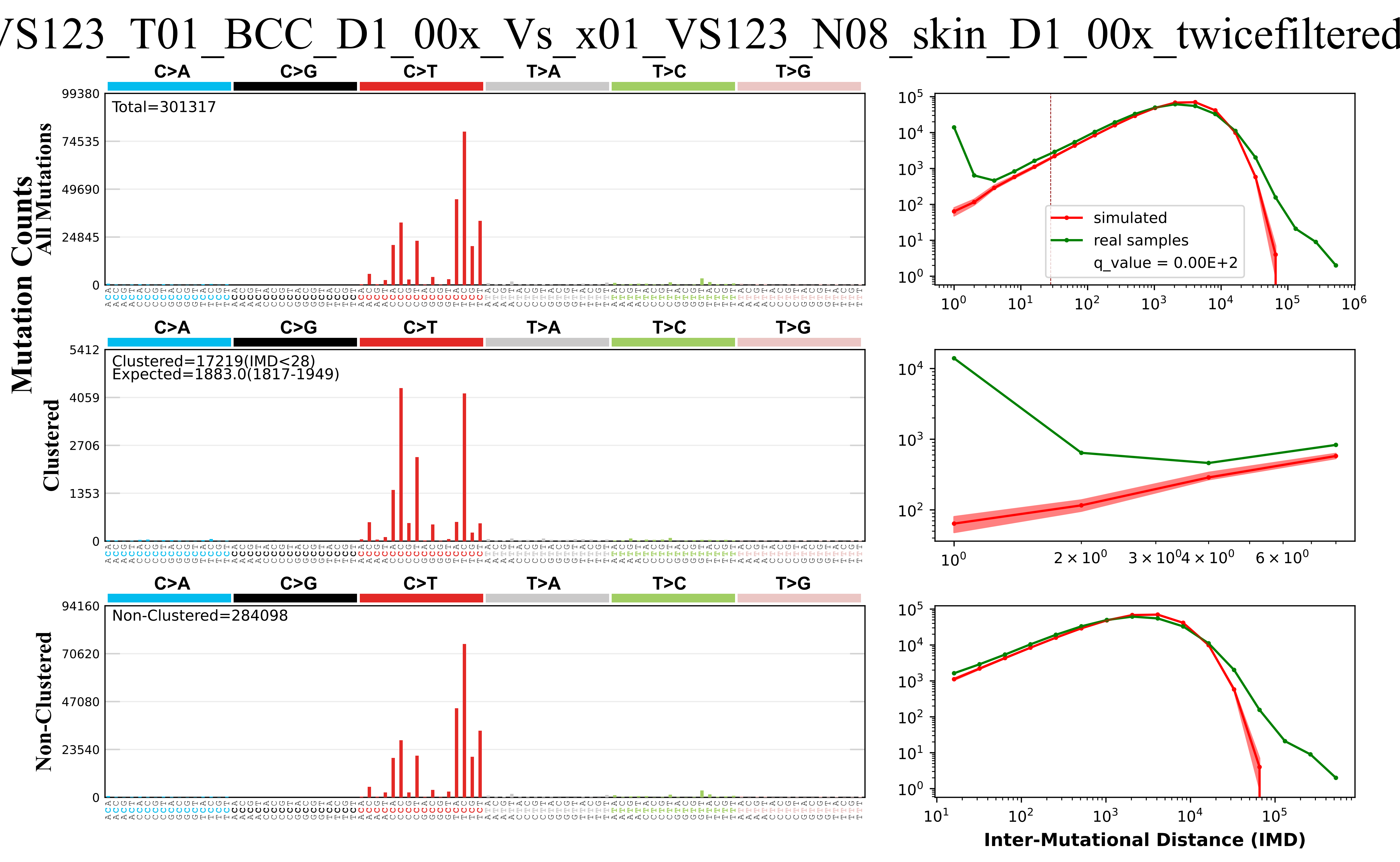

VS123_T01_BCC_D1_00x_Vs_x01_VS123_N08_skin_D1_00x_twicefiltered
Mutation Counts
All Mutations
Clustered
Non-Clustered
C>A
C>G
C>T
T>A
T>C
T>G
Total=301317
Clustered=17219(IMD<28)
Expected=1883.0(1817-1949)
Non-Clustered=284098
simulated
real samples
q_value = 0.00E+2
Inter-Mutational Distance (IMD)

DRUP01060021T_Vs_DRUP01060021R_twicefiltered_TvN

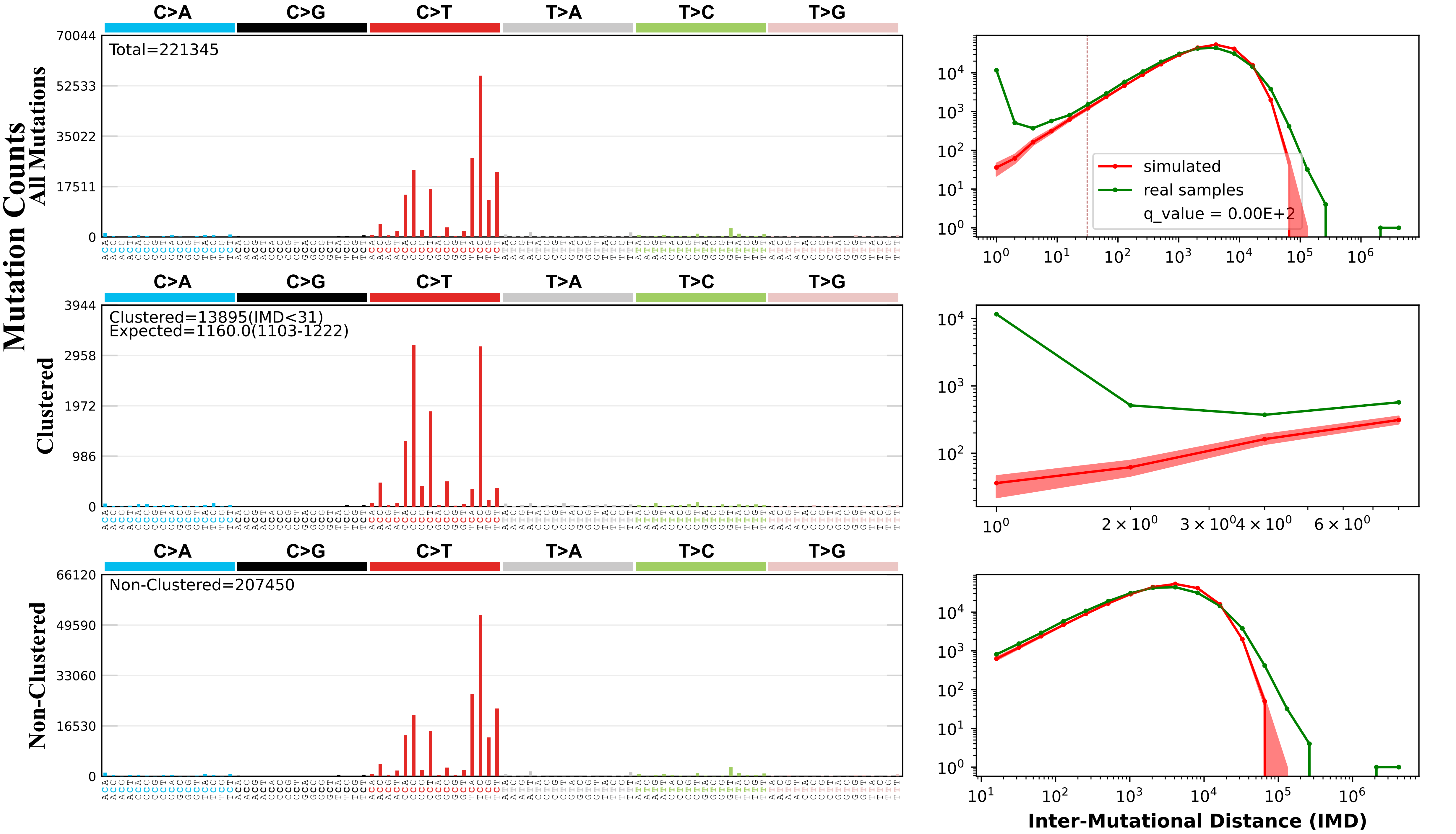

CPCT02060043T_Vs_CPCT02060043R_twicefiltered_TvN

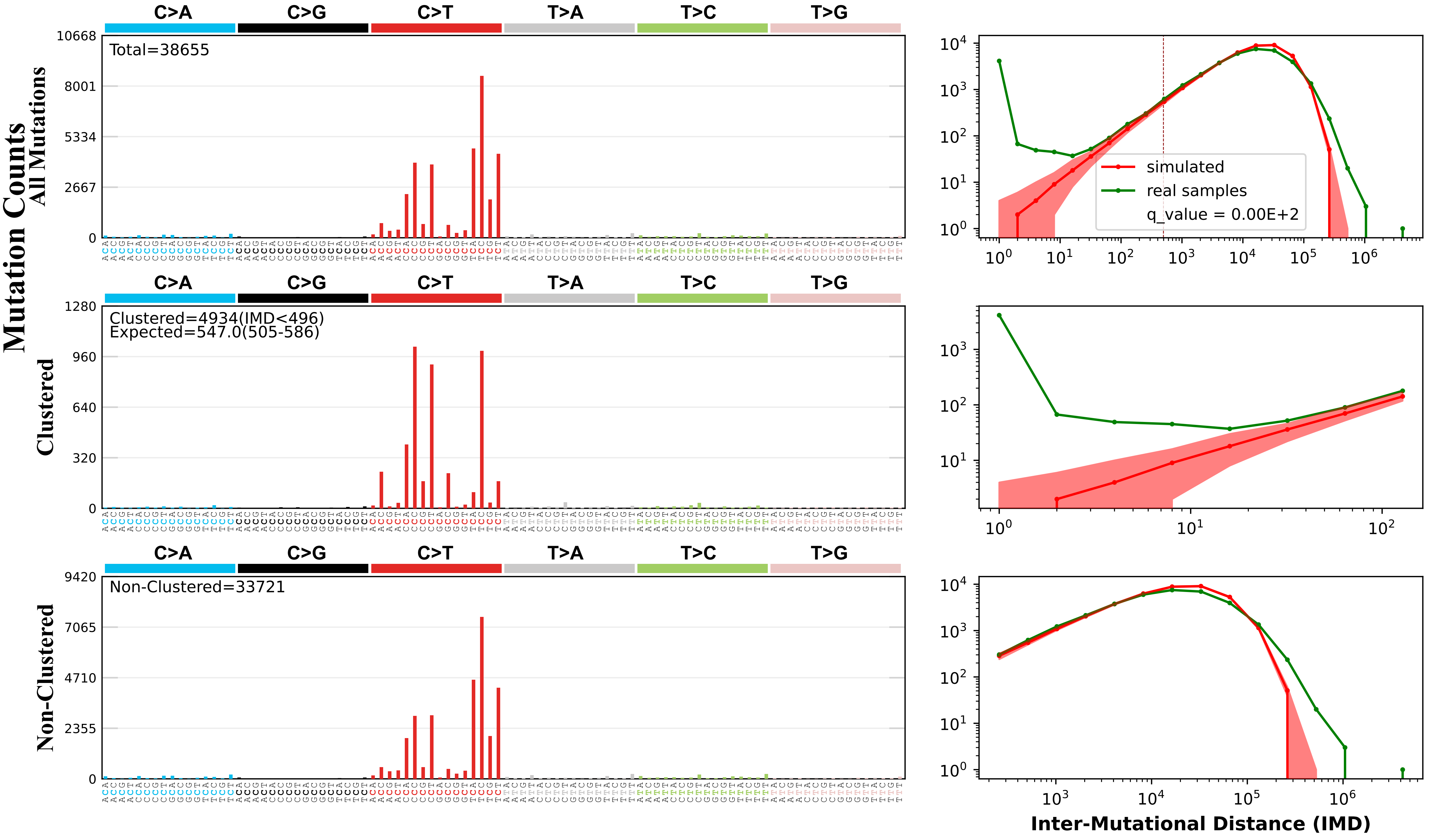

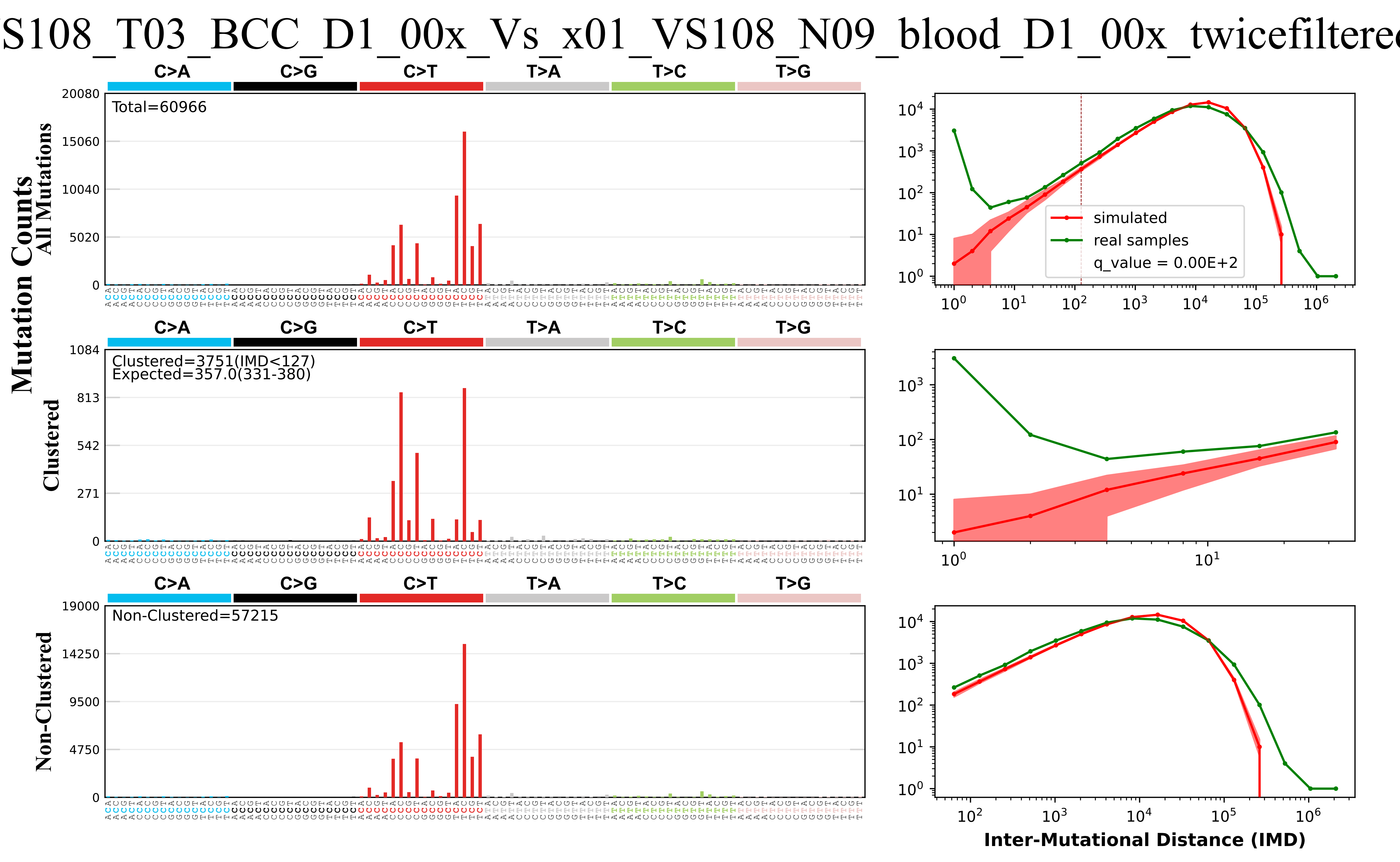
S108_T03_BCC_D1_00x_Vs_x01_VS108_N09_blood_D1_00x_twicefiltere
Mutation Counts
All Mutations
Clustered
Non-Clustered
C>A
C>G
C>T
T>A
T>C
T>G
Total=60966
Clustered=3751(IMD<127)
Expected=357.0(331-380)
Non-Clustered=57215
20080
15060
10040
5020
0
1084
813
542
271
19000
14250
9500
4750
simulated
real samples
q_value = 0.00E+2
Inter-Mutational Distance (IMD)

VS101_T05_SCC_D1_00x_Vs_x01_VS101_N04_skin_D1_00x_twicefiltered

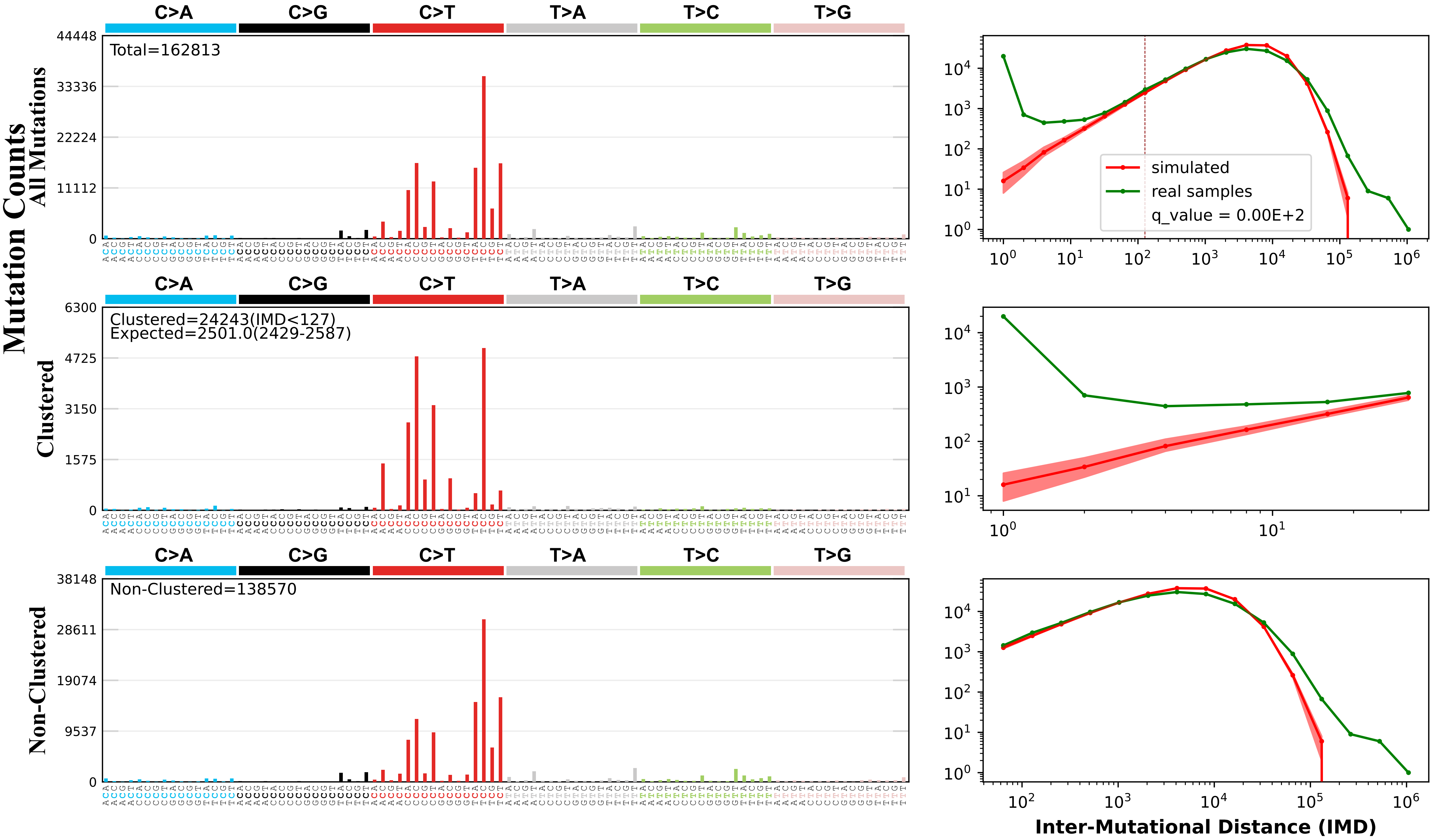

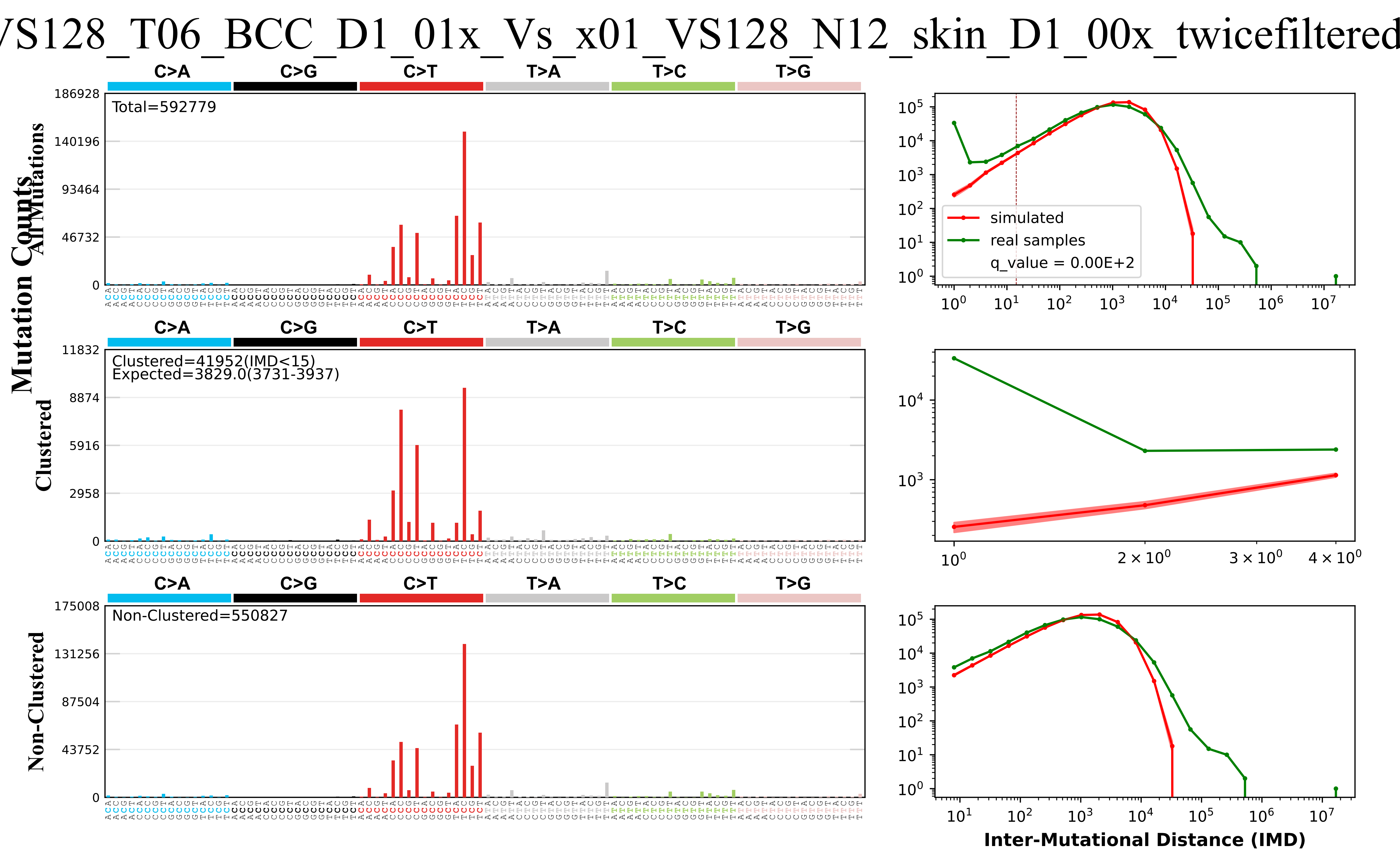
VS128_T06_BCC_D1_01x_Vs_x01_VS128_N12_skin_D1_00x_twicefiltered
Mutation Counts
All Mutations
Total=592779
Clustered
Clustered=41952(IMD<15)
Expected=3829.0(3731-3937)
Non-Clustered
Non-Clustered=550827
C>A
C>G
C>T
T>A
T>C
T>G
simulated
real samples
q_value = 0.00E+2
Inter-Mutational Distance (IMD)

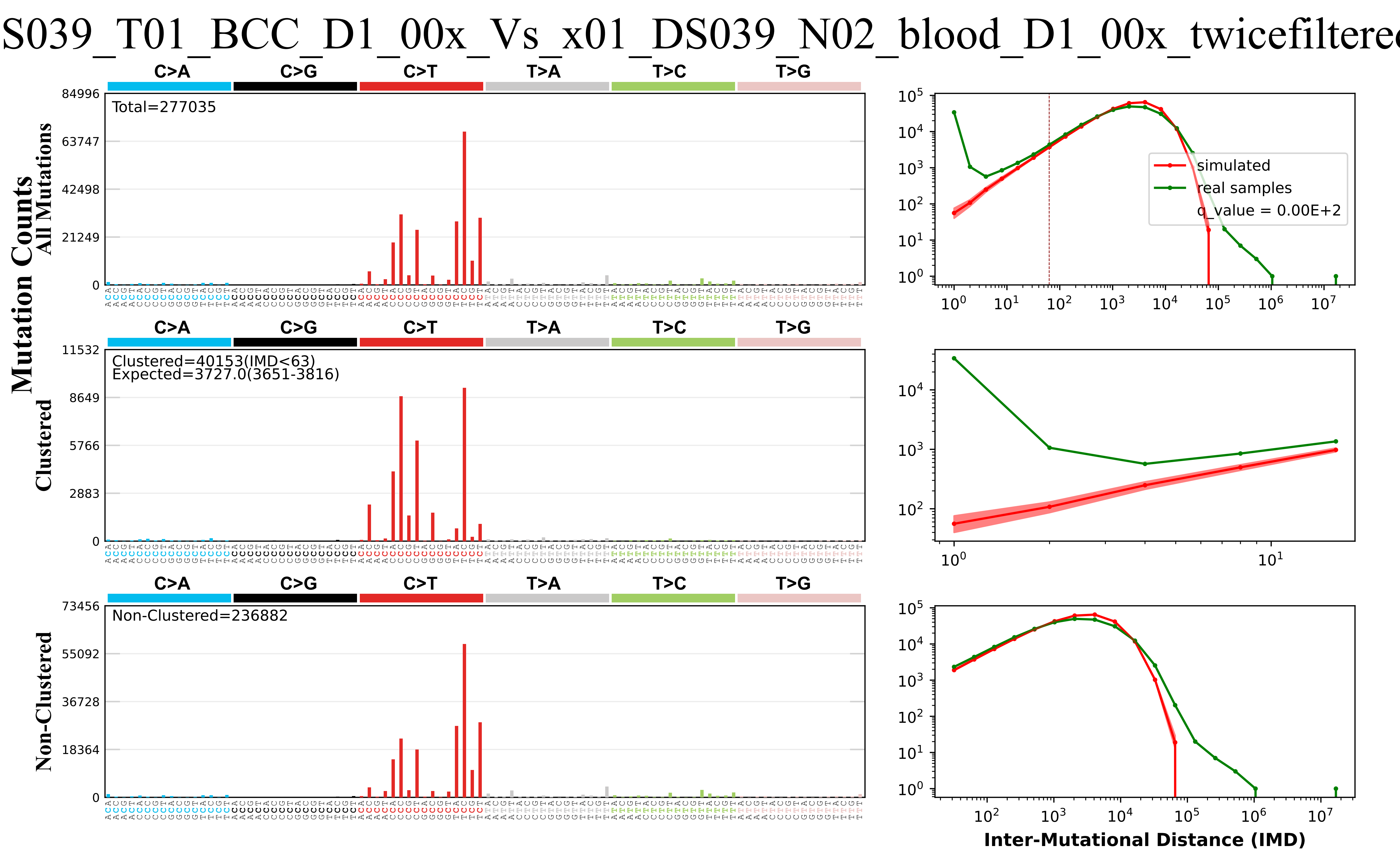
S039_T01_BCC_D1_00x_Vs_x01_DS039_N02_blood_D1_00x_twicefiltere
C>A
C>G
C>T
T>A
T>C
T>G
Mutation Counts
All Mutations
Total=277035
84996
63747
42498
21249
0
Clustered
Clustered=40153(IMD<63)
Expected=3727.0(3651-3816)
11532
8649
5766
2883
0
Non-Clustered
Non-Clustered=236882
73456
55092
36728
18364
0
simulated
real samples
q_value = 0.00E+2
Inter-Mutational Distance (IMD)

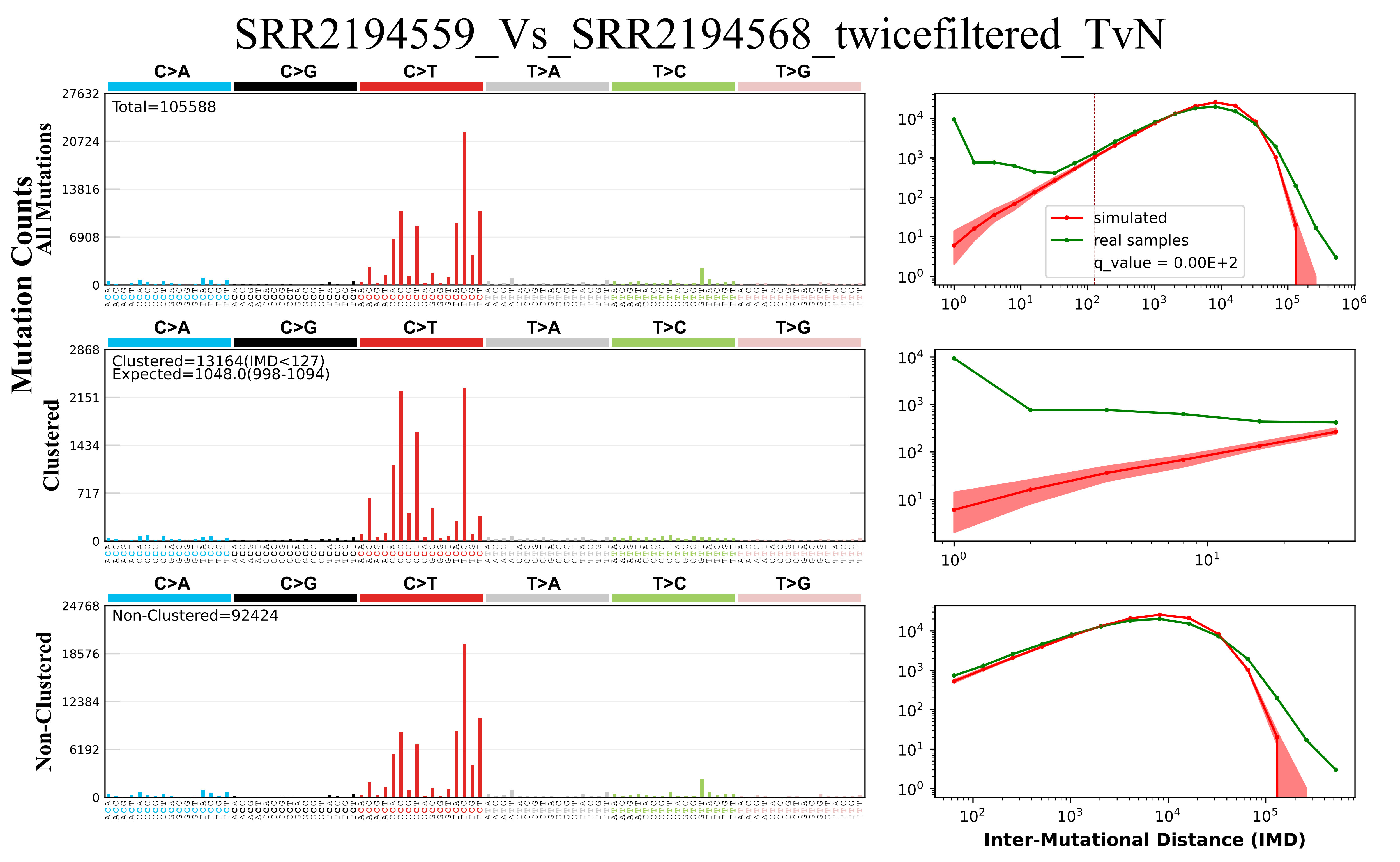

SRR2194559_Vs_SRR2194568_twicefiltered_TvN
Mutation Counts
All Mutations
Clustered
Non-Clustered
C>A
C>G
C>T
T>A
T>C
T>G
Total=105588
Clustered=13164(IMD<127)
Expected=1048.0(998-1094)
Non-Clustered=92424
27632
20724
13816
6908
0
2868
2151
1434
717
24768
18576
12384
6192
simulated
real samples
q_value = 0.00E+2
Inter-Mutational Distance (IMD)

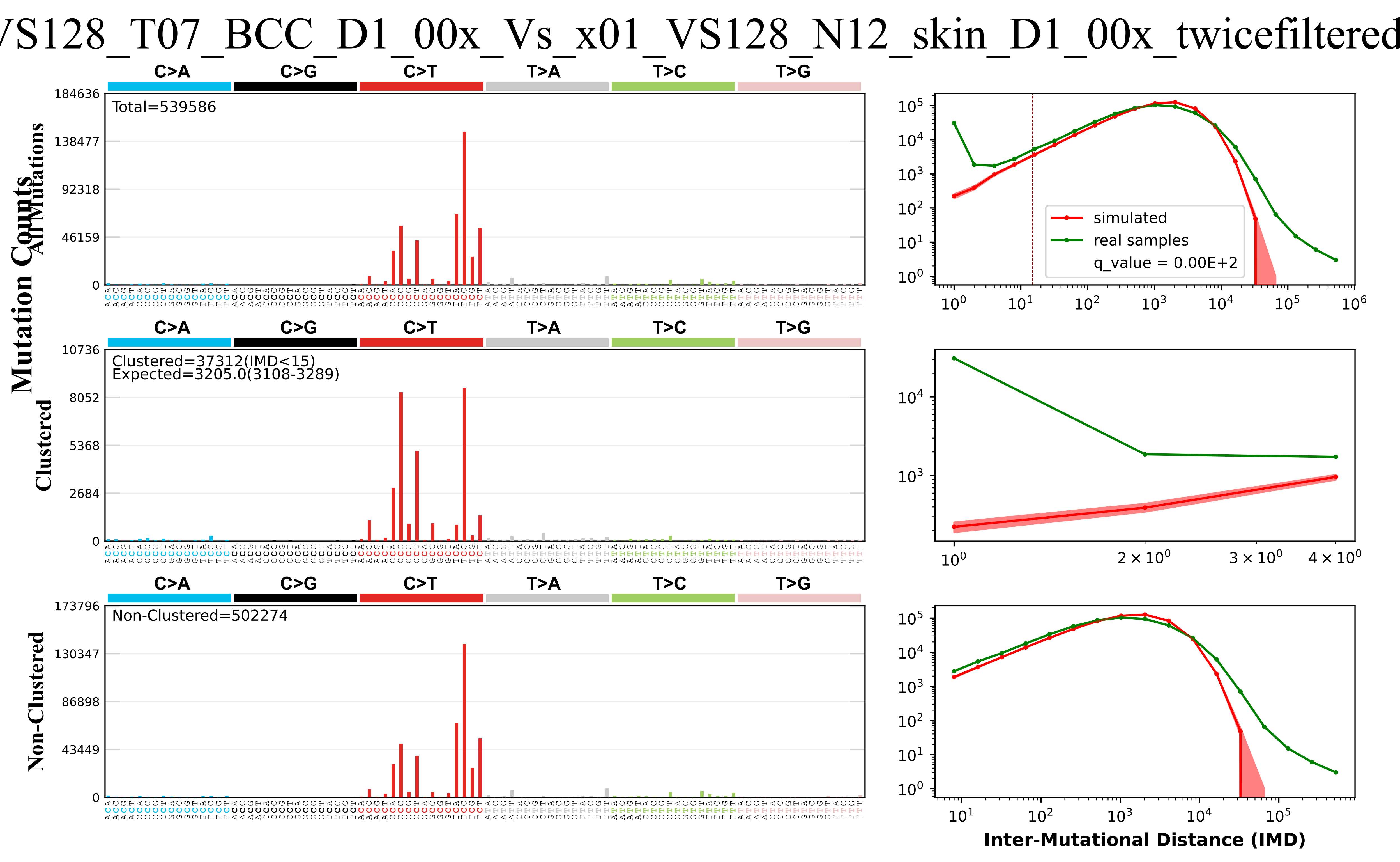

/S128_T07_BCC_D1_00x_Vs_x01_VS128_N12_skin_D1_00x_twicefiltered
Mutation Counts
All Mutations
C>A
C>G
C>T
T>A
T>C
T>G
Total=539586
184636
138477
92318
46159
0
simulated
real samples
q_value = 0.00E+2
Clustered
Clustered=37312(IMD<15)
Expected=3205.0(3108-3289)
10736
8052
5368
2684
Non-Clustered
Non-Clustered=502274
173796
130347
86898
43449
Inter-Mutational Distance (IMD)

VS128_T09_BCC_D1_00x_Vs_x01_VS128_N12_skin_D1_00x_twicefiltered

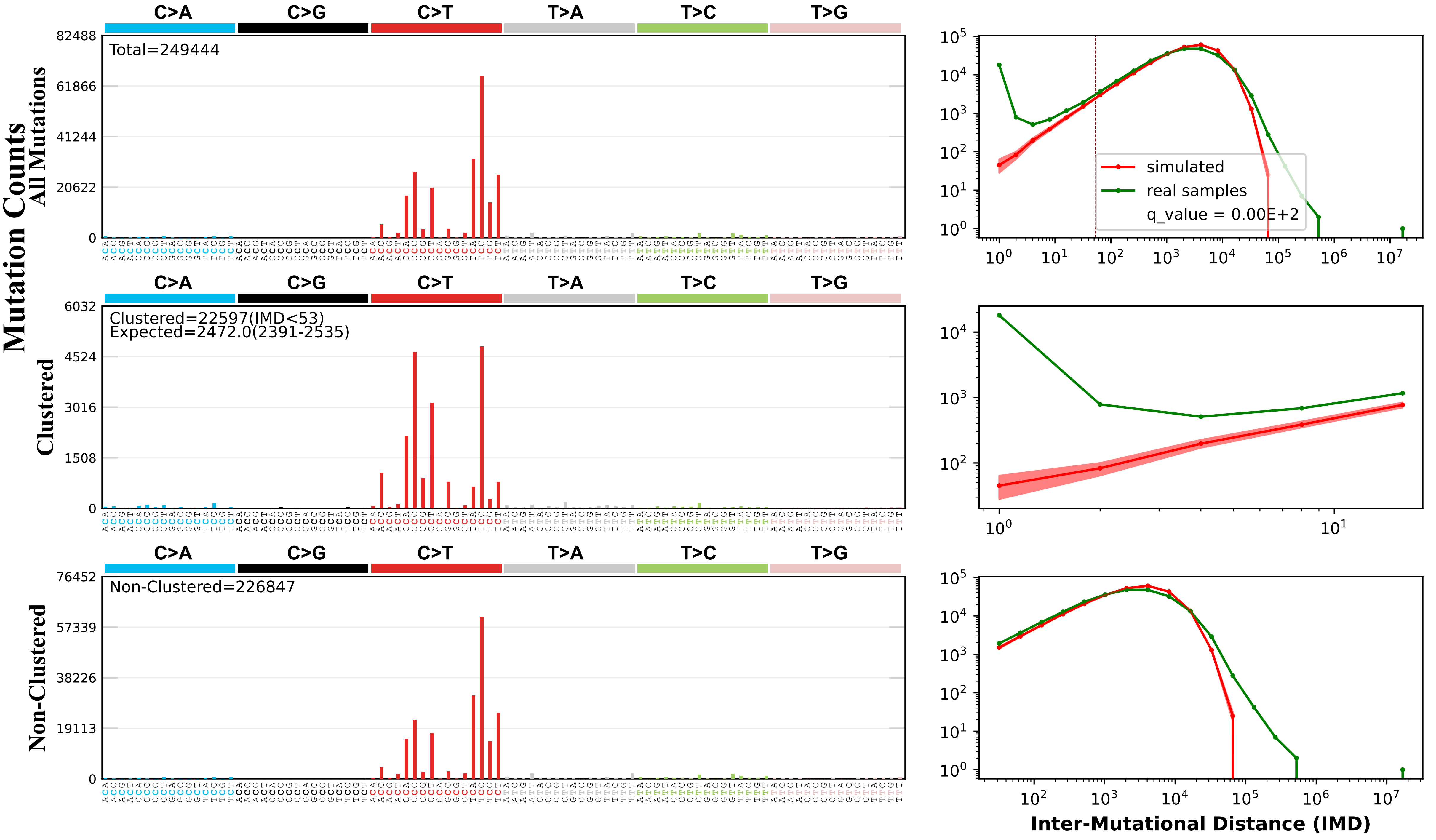

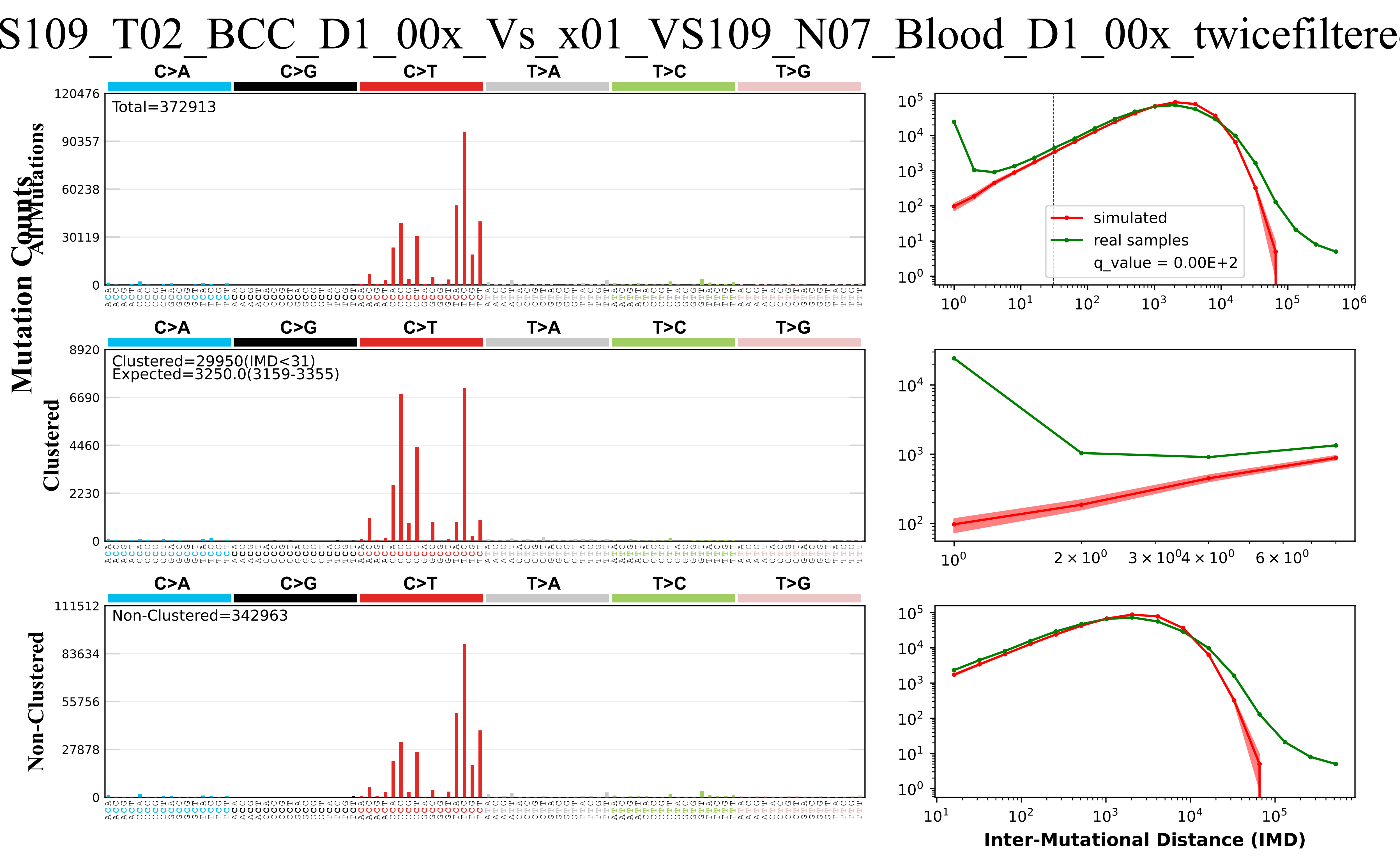

S109_T02_BCC_D1_00x_Vs_x01_VS109_N07_Blood_D1_00x_twicefiltere
Mutation Counts
All Mutations
C>A
C>G
C>T
T>A
T>C
T>G
Total=372913
Clustered
Clustered=29950(IMD<31)
Expected=3250.0(3159-3355)
Non-Clustered
Non-Clustered=342963
simulated
real samples
q_value = 0.00E+2
Inter-Mutational Distance (IMD)

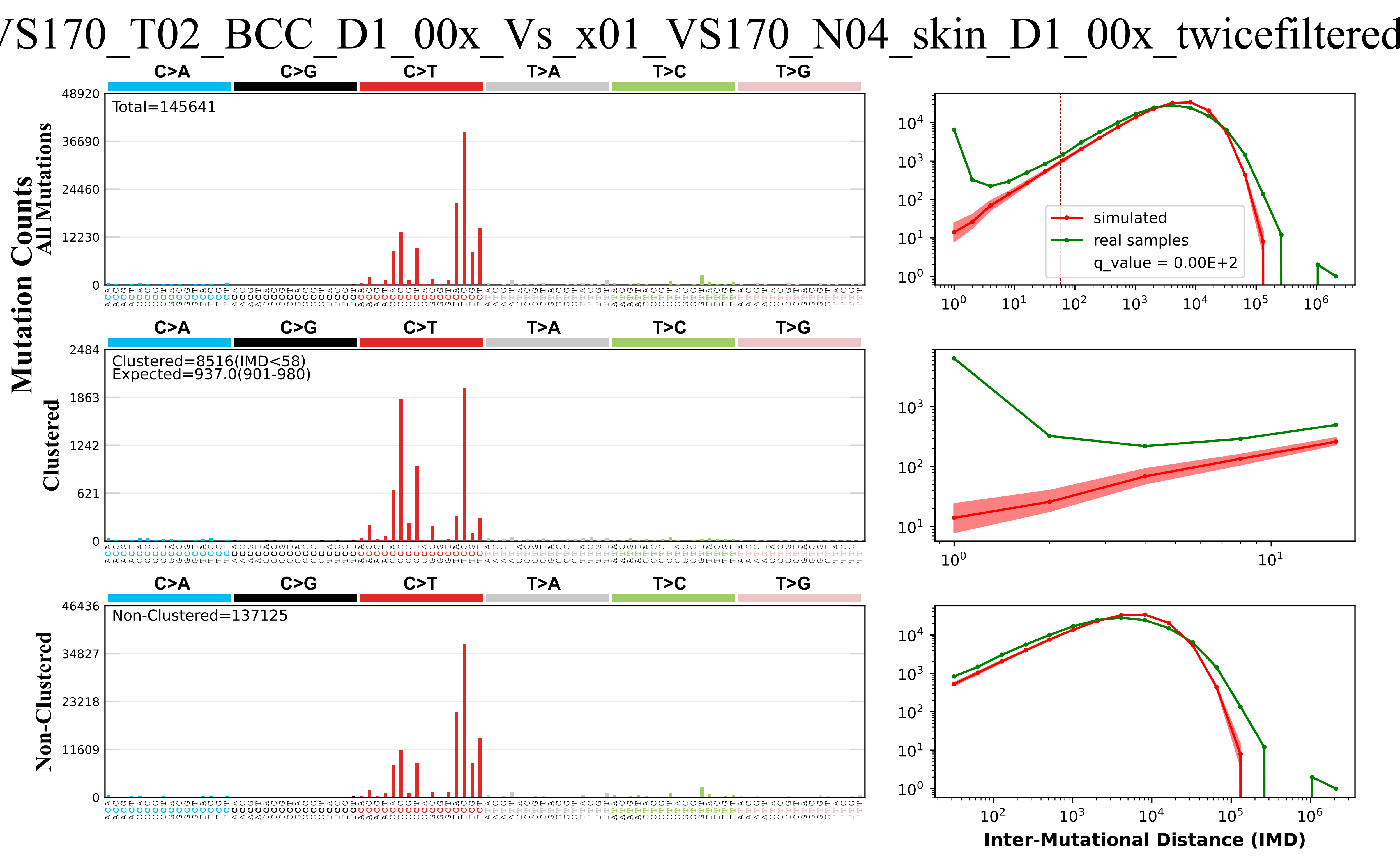

VS170_T02_BCC_D1_00x_Vs_x01_VS170_N04_skin_D1_00x_twicefiltered
Mutation Counts
All Mutations
Clustered
Non-Clustered
C>A
C>G
C>T
T>A
T>C
T>G
Total=145641
Clustered=8516(IMD<58)
Expected=937.0(901-980)
Non-Clustered=137125
simulated
real samples
q_value = 0.00E+2
Inter-Mutational Distance (IMD)

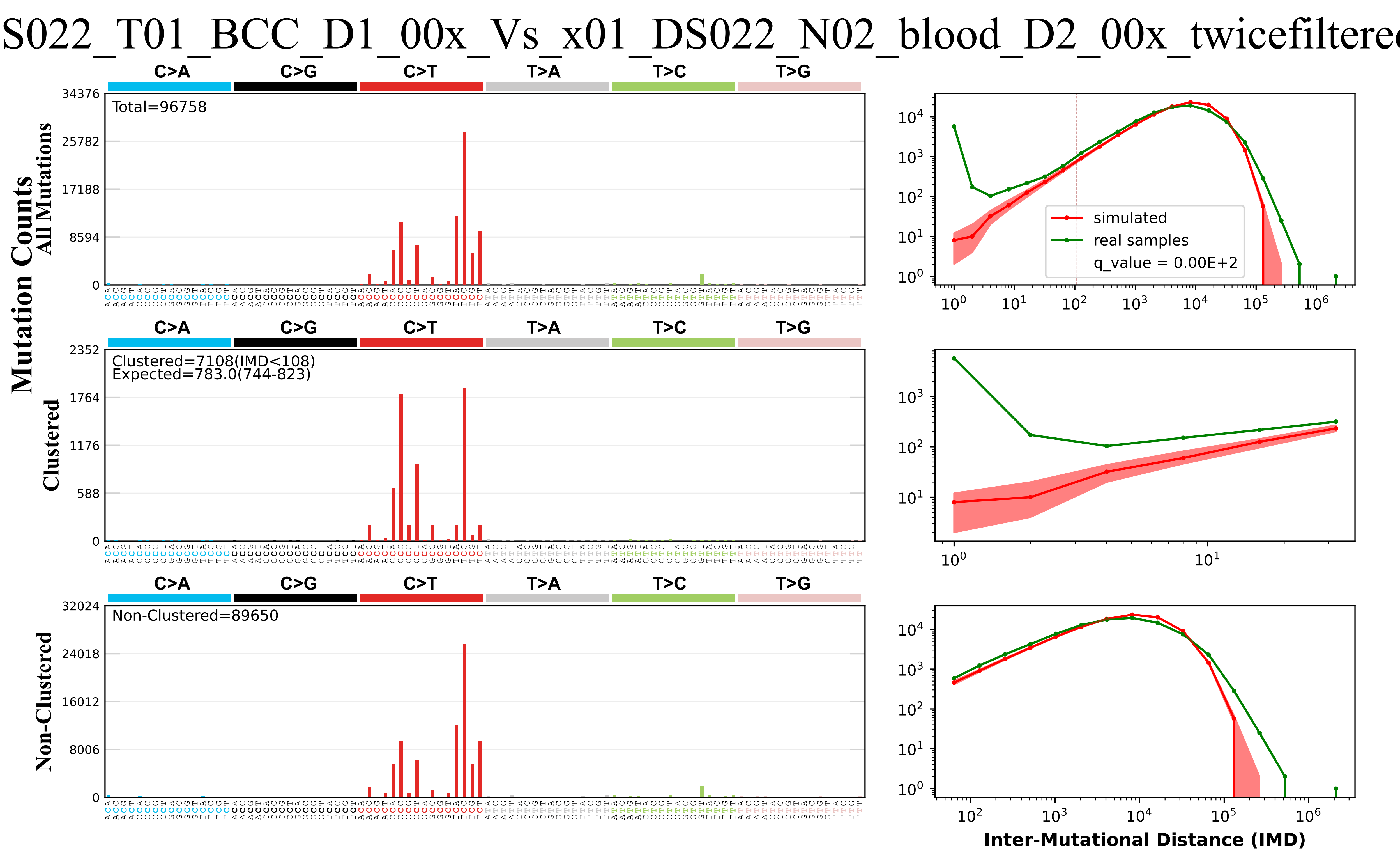
S022_T01_BCC_D1_00x_Vs_x01_DS022_N02_blood_D2_00x_twicefiltere
Mutation Counts
All Mutations
Clustered
Non-Clustered
C>A
C>G
C>T
T>A
T>C
T>G
Total=96758
Clustered=7108(IMD<108)
Expected=783.0(744-823)
Non-Clustered=89650
simulated
real samples
q_value = 0.00E+2
Inter-Mutational Distance (IMD)

VS173_T03_SCC_D1_01x_Vs_x01_VS173_N06_skin_D1_00x_twicefiltered

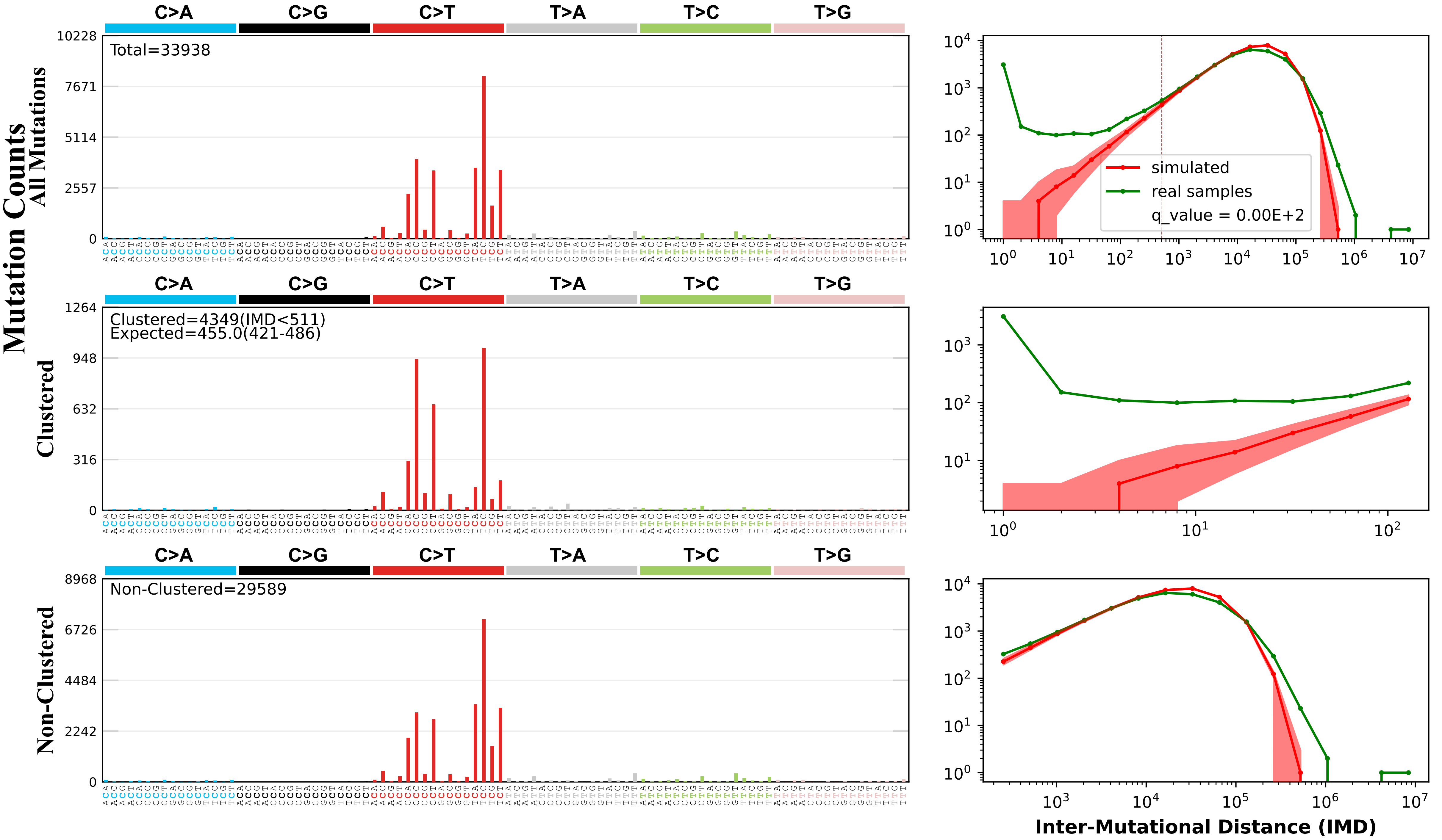

S079_T05_BCC_D1_01x_Vs_x01_VS079_N09_blood_D1_00x_twicefilterec

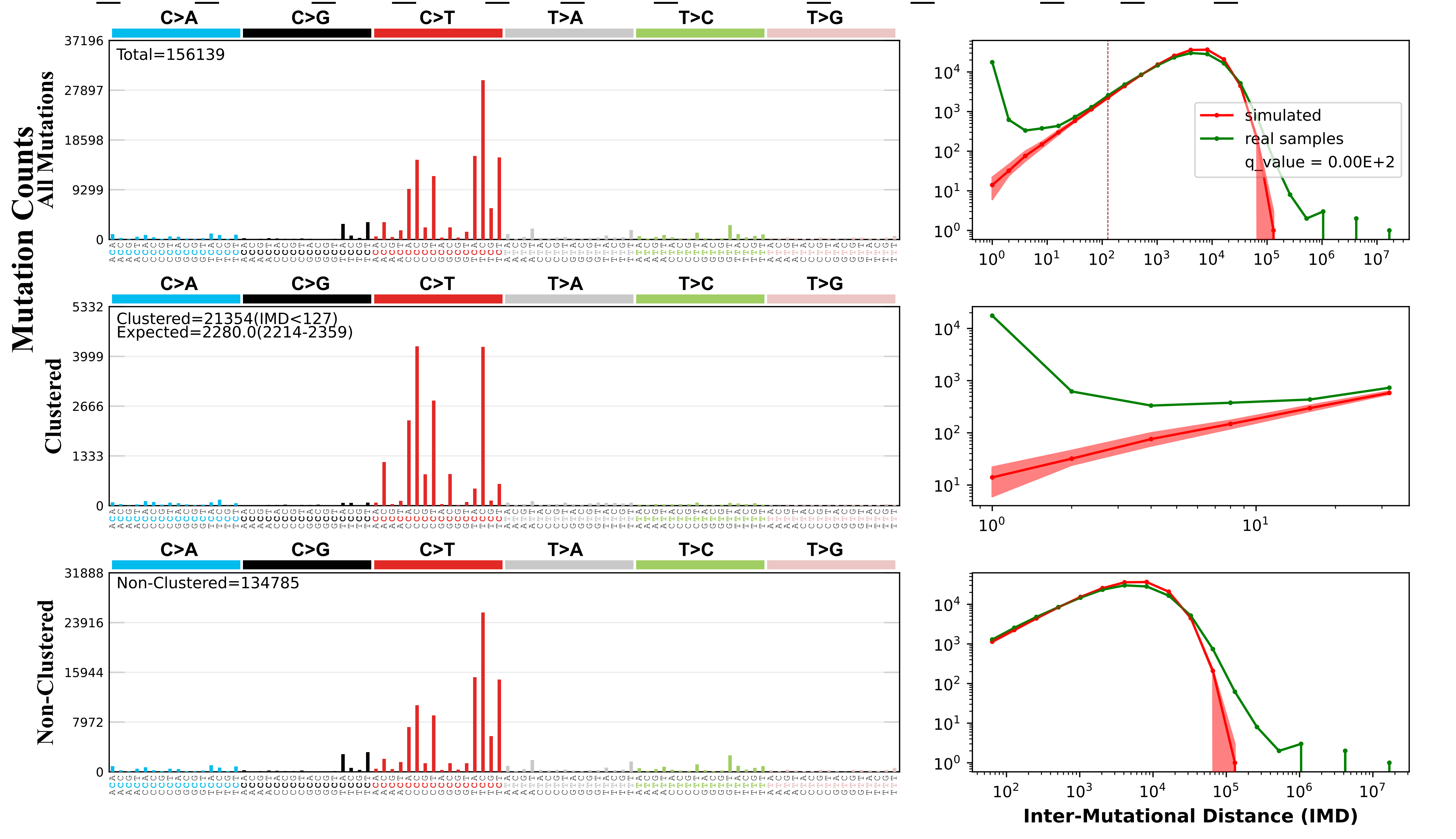

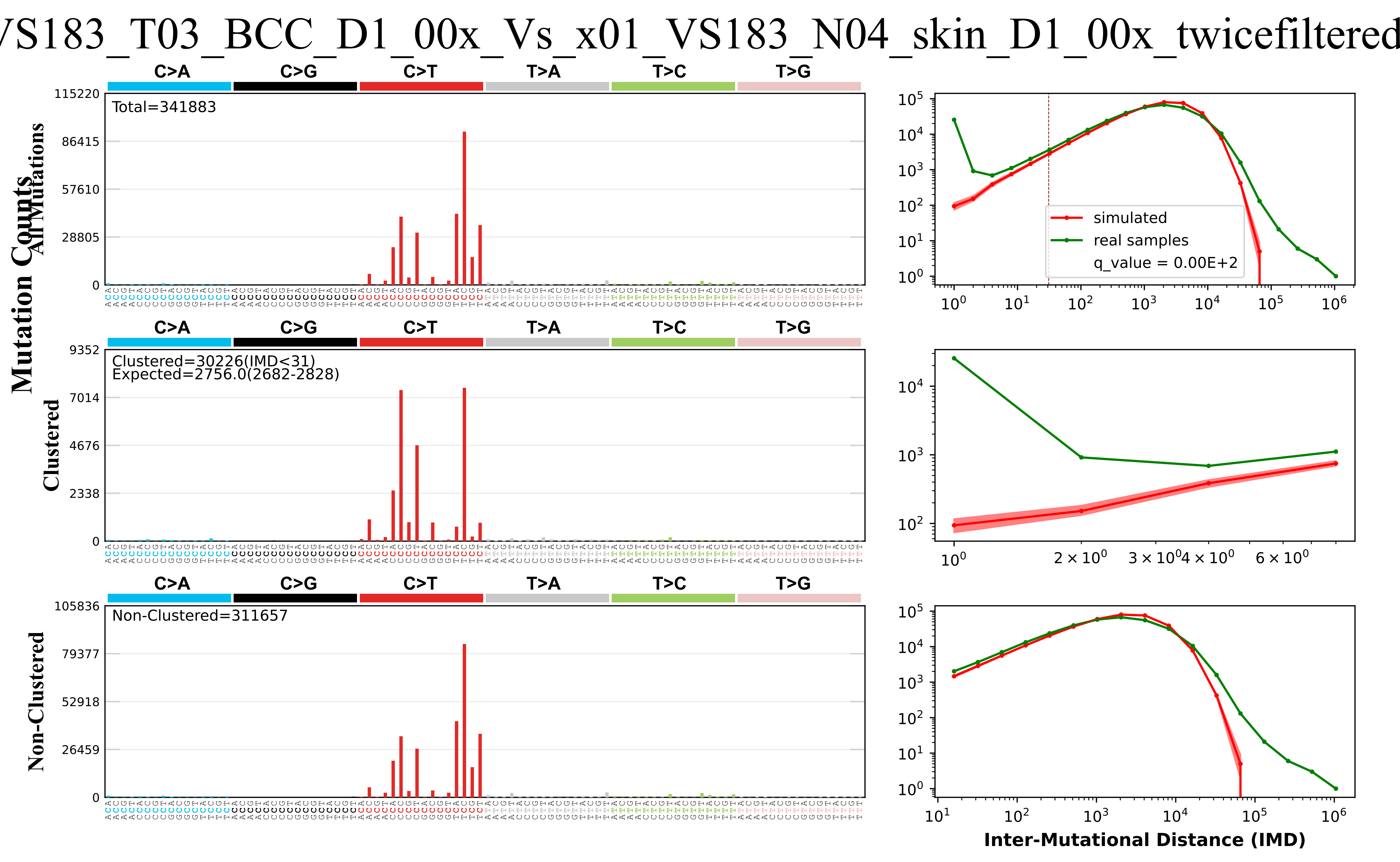

VS183_T03_BCC_D1_00x_Vs_x01_VS183_N04_skin_D1_00x_twicefiltered
Mutation Counts
All Mutations
Clustered
Non-Clustered
C>A
C>G
C>T
T>A
T>C
T>G
Total=341883
Clustered=30226(IMD<31)
Expected=2756.0(2682-2828)
Non-Clustered=311657
simulated
real samples
q_value = 0.00E+2
Inter-Mutational Distance (IMD)

S006_T02_BCC_D1_00x_Vs_x01_VS006_N16_blood_D1_00x_twicefiltere

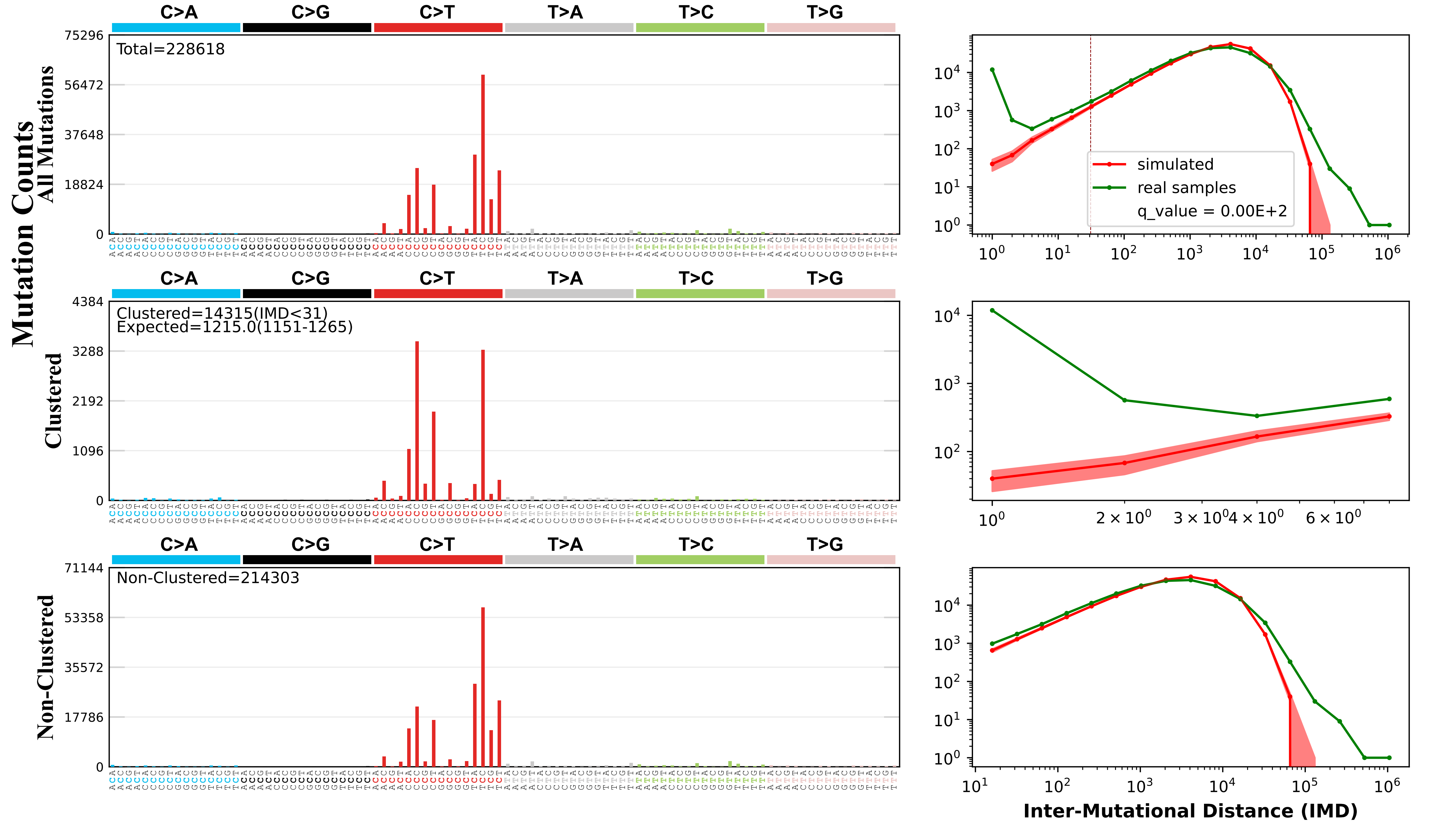

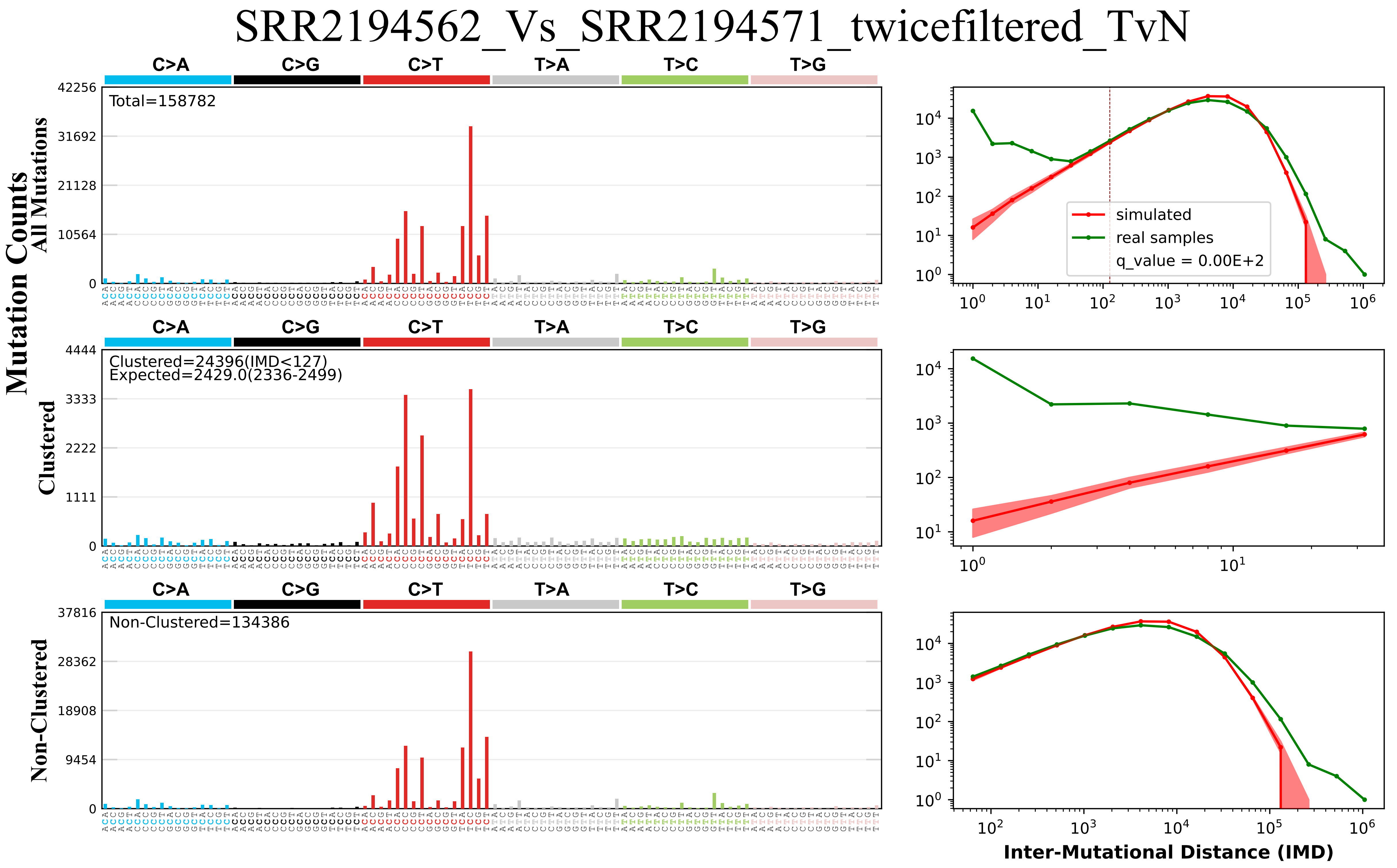
SRR2194562_Vs_SRR2194571_twicefiltered_TvN
C>A
C>G
C>T
T>A
T>C
T>G
Mutation Counts
All Mutations
Total=158782
Clustered
Clustered=24396(IMD<127)
Expected=2429.0(2336-2499)
Non-Clustered
Non-Clustered=134386
simulated
real samples
q_value = 0.00E+2
Inter-Mutational Distance (IMD)

VS194_T01_BCC_D1_00x_Vs_x01_VS194_N02_skin_D1_00x_twicefiltered

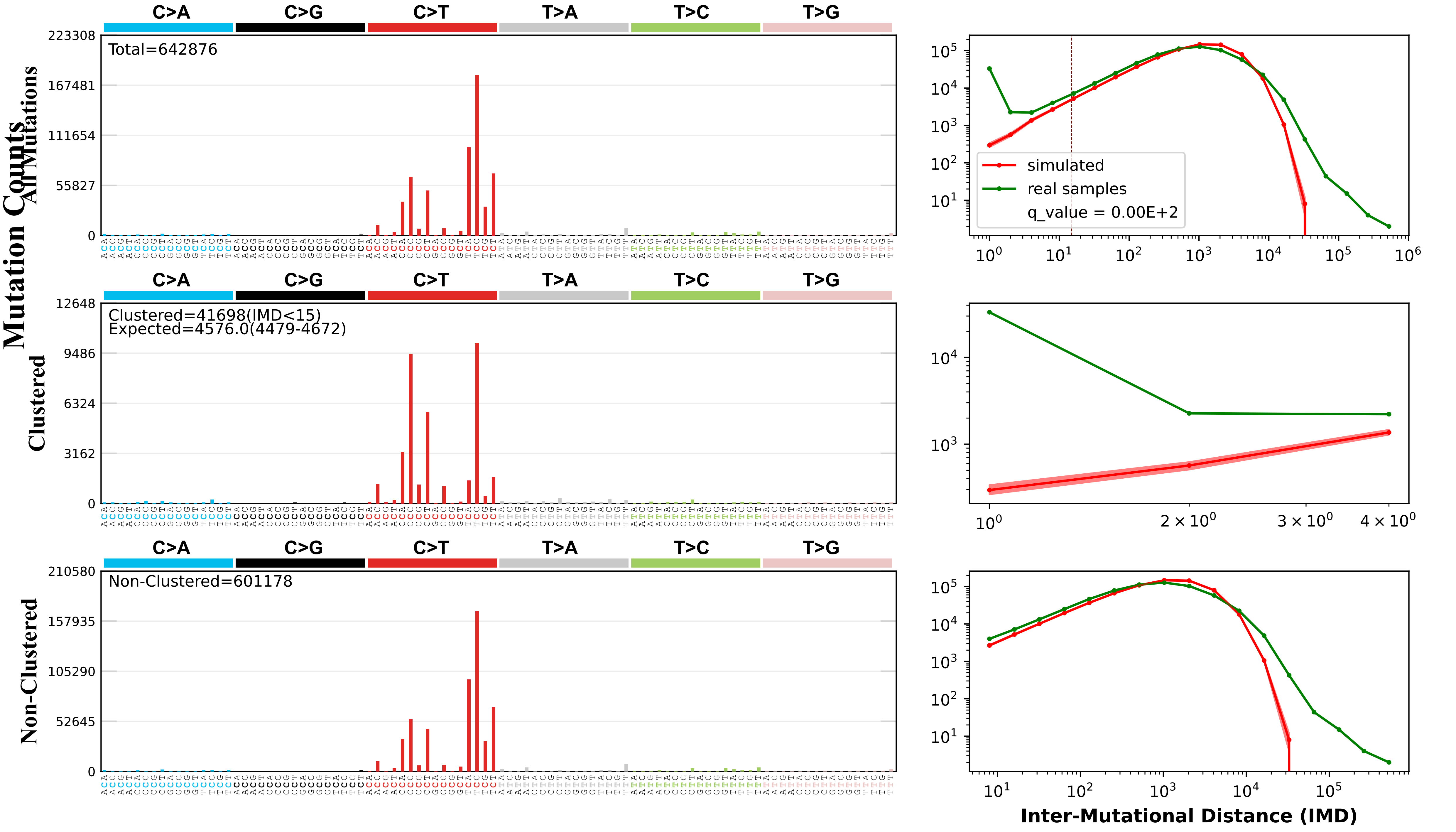

S102_T01_SCC_D1_00x_Vs_x01_DS102_N03_Blood_D1_00x_twicefiltere

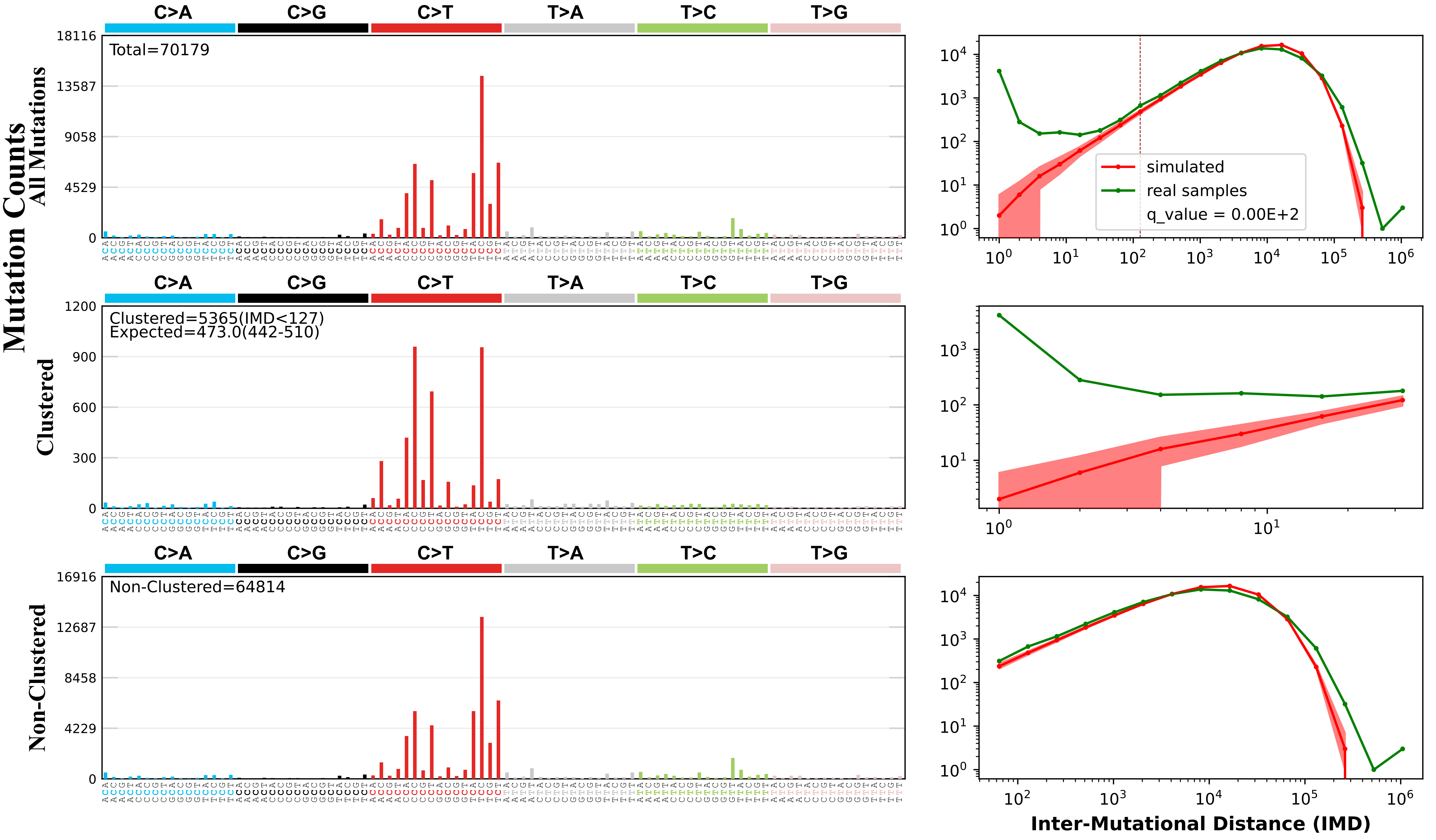

S113_T01_BCC_D1_00x_Vs_x01_DS113_N03_Blood_D1_00x_twicefiltere

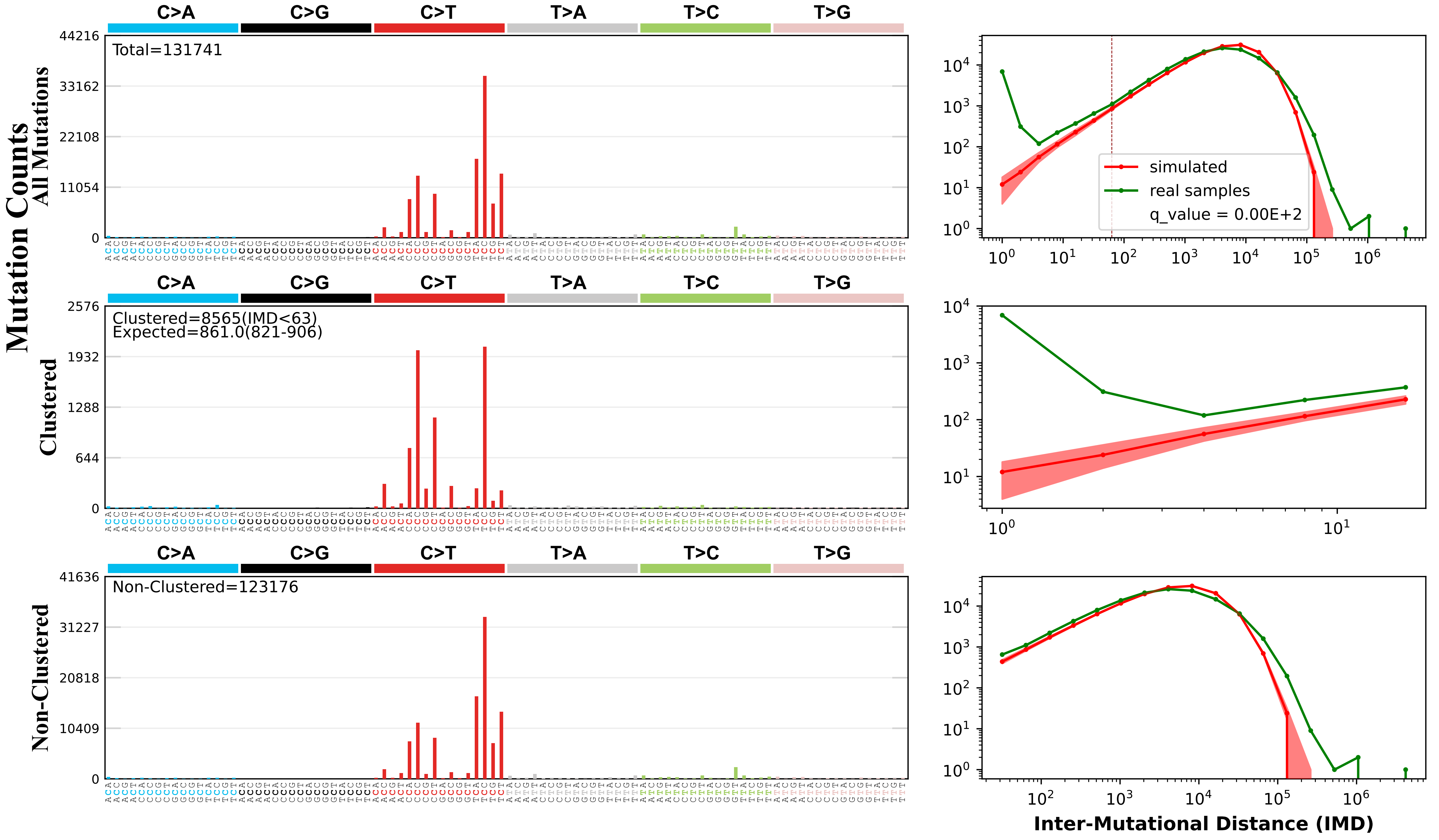

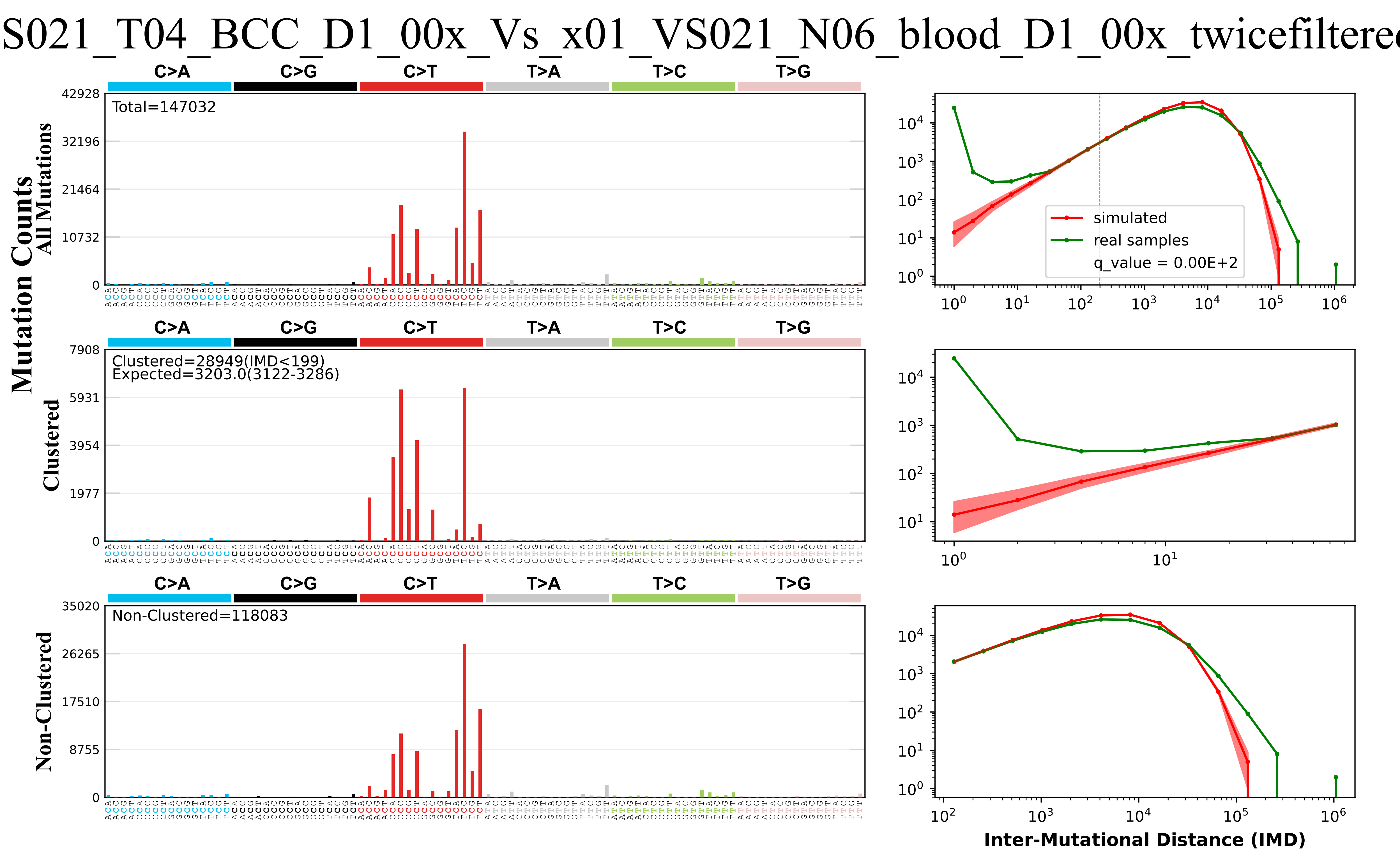
S021_T04_BCC_D1_00x_Vs_x01_VS021_N06_blood_D1_00x_twicefiltere
Mutation Counts
All Mutations
Clustered
Non-Clustered
C>A
C>G
C>T
T>A
T>C
T>G
Total=147032
Clustered=28949(IMD<199)
Expected=3203.0(3122-3286)
Non-Clustered=118083
simulated
real samples
q_value = 0.00E+2
Inter-Mutational Distance (IMD)

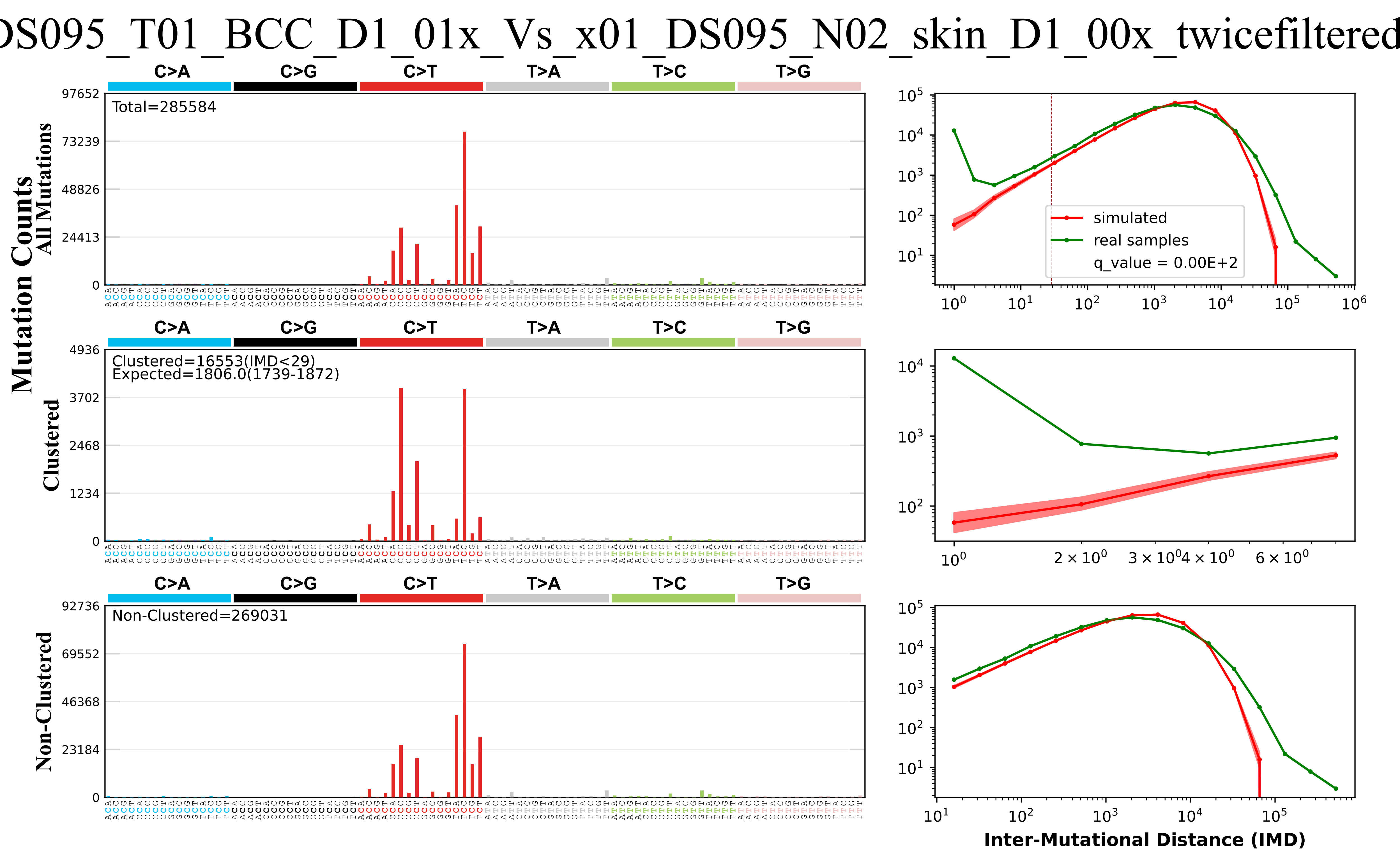

DS095_T01_BCC_D1_01x_Vs_x01_DS095_N02_skin_D1_00x_twicefiltered
Mutation Counts
All Mutations
Clustered
Non-Clustered
C>A
C>G
C>T
T>A
T>C
T>G
Total=285584
Clustered=16553(IMD<29)
Expected=1806.0(1739-1872)
Non-Clustered=269031
simulated
real samples
q_value = 0.00E+2
Inter-Mutational Distance (IMD)

S142_T01_BCC_D1_01x_Vs_x01_VS142_N07_Blood_D1_00x_twicefiltere

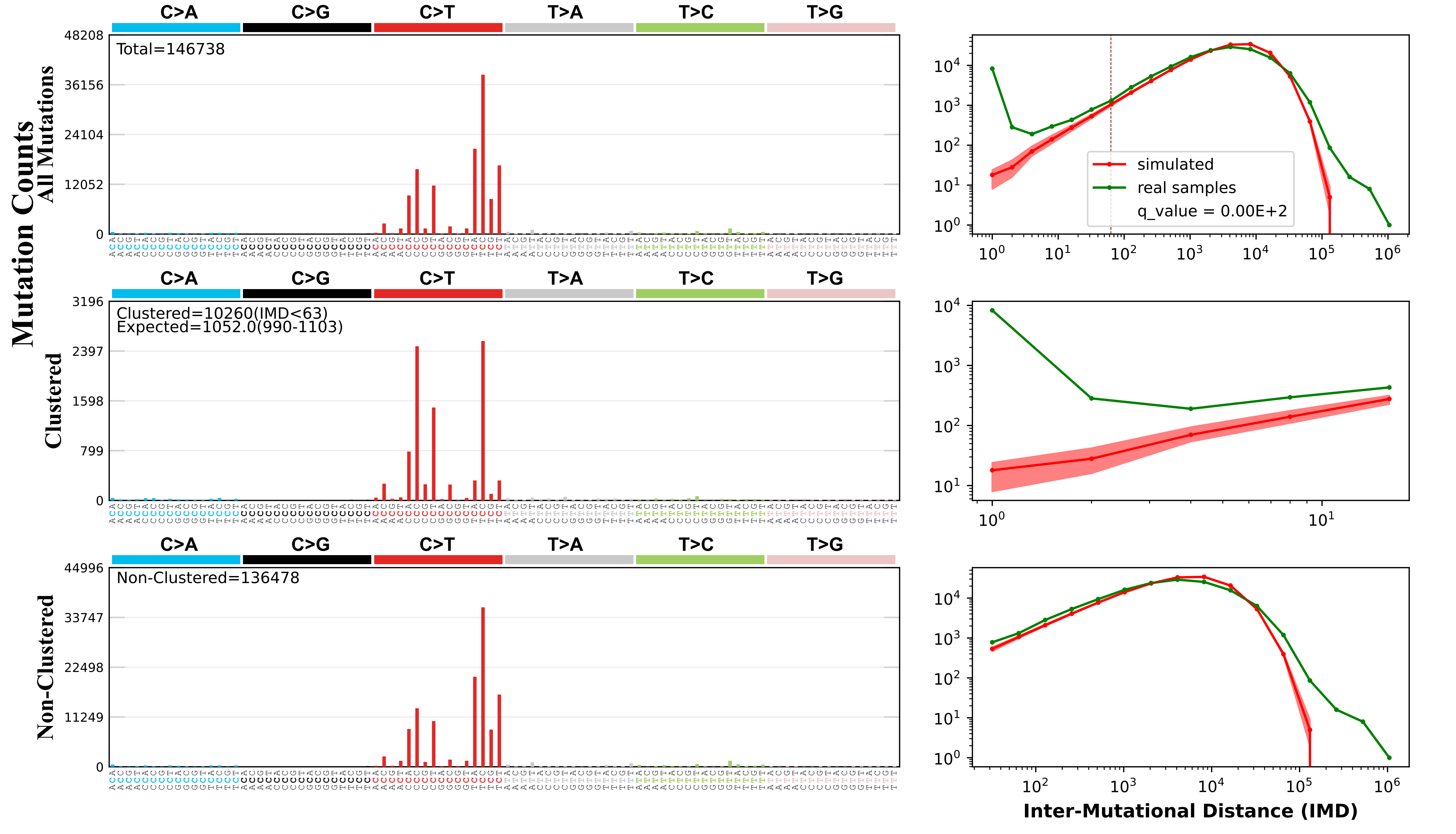

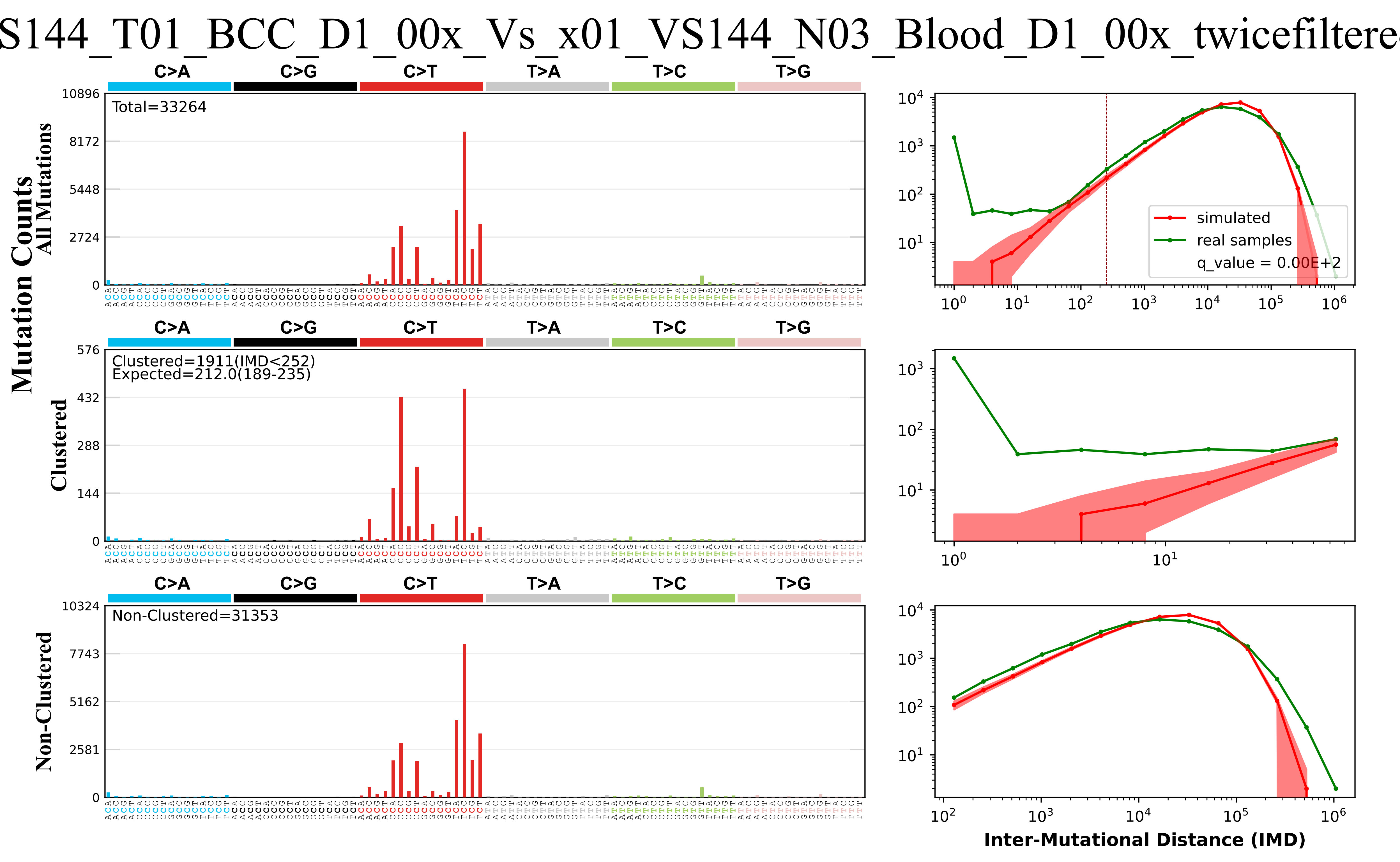
S144_T01_BCC_D1_00x_Vs_x01_VS144_N03_Blood_D1_00x_twicefiltere
Mutation Counts
All Mutations
Clustered
Non-Clustered
C>A
C>G
C>T
T>A
T>C
T>G
Total=33264
Clustered=1911(IMD<252)
Expected=212.0(189-235)
Non-Clustered=31353
simulated
real samples
q_value = 0.00E+2
Inter-Mutational Distance (IMD)

CPCT02050105T_Vs_CPCT02050105R_twicefiltered_TvN

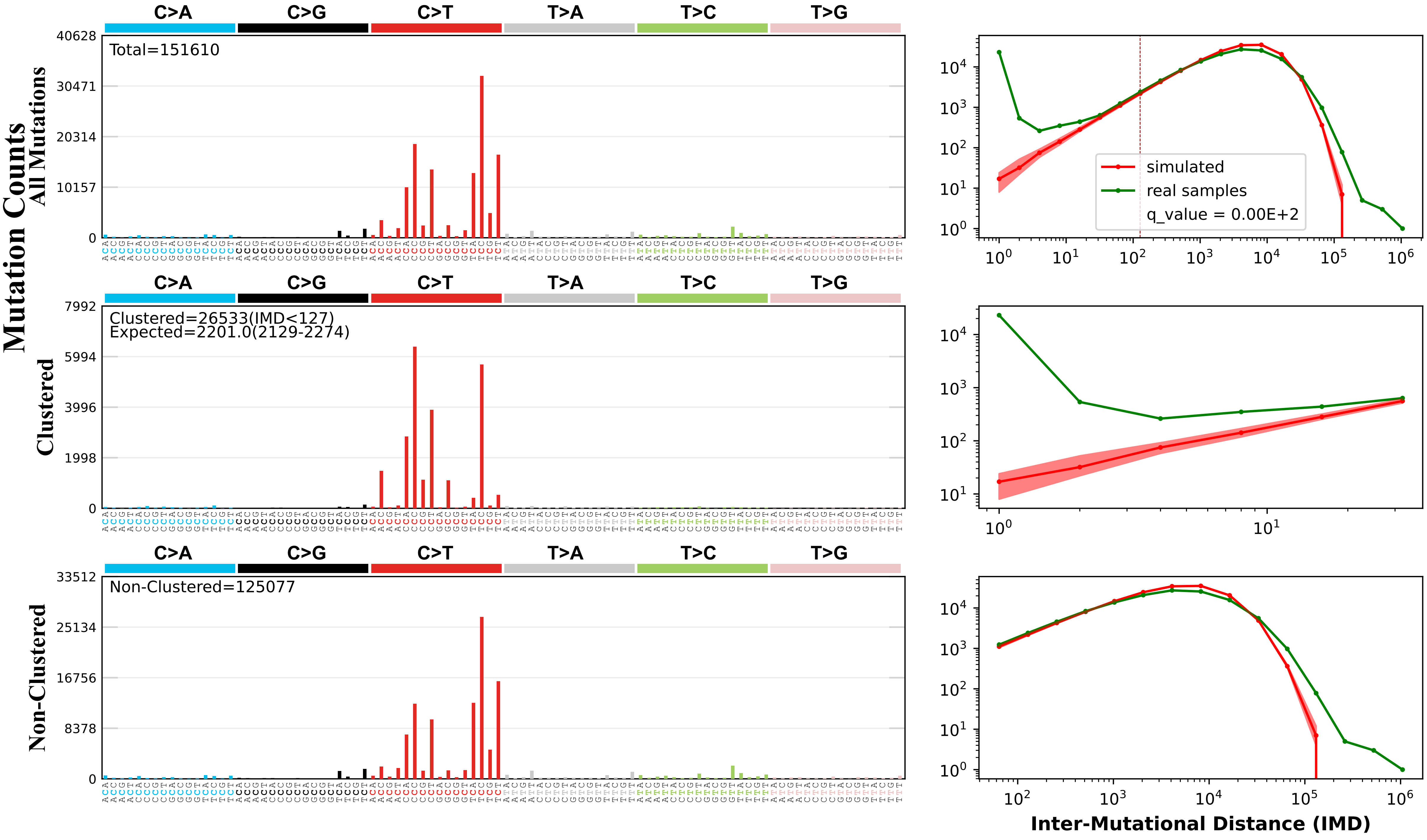

S135_T02_BCC_D1_00x_Vs_x01_VS135_N05_Blood_D1_00x_twicefiltere

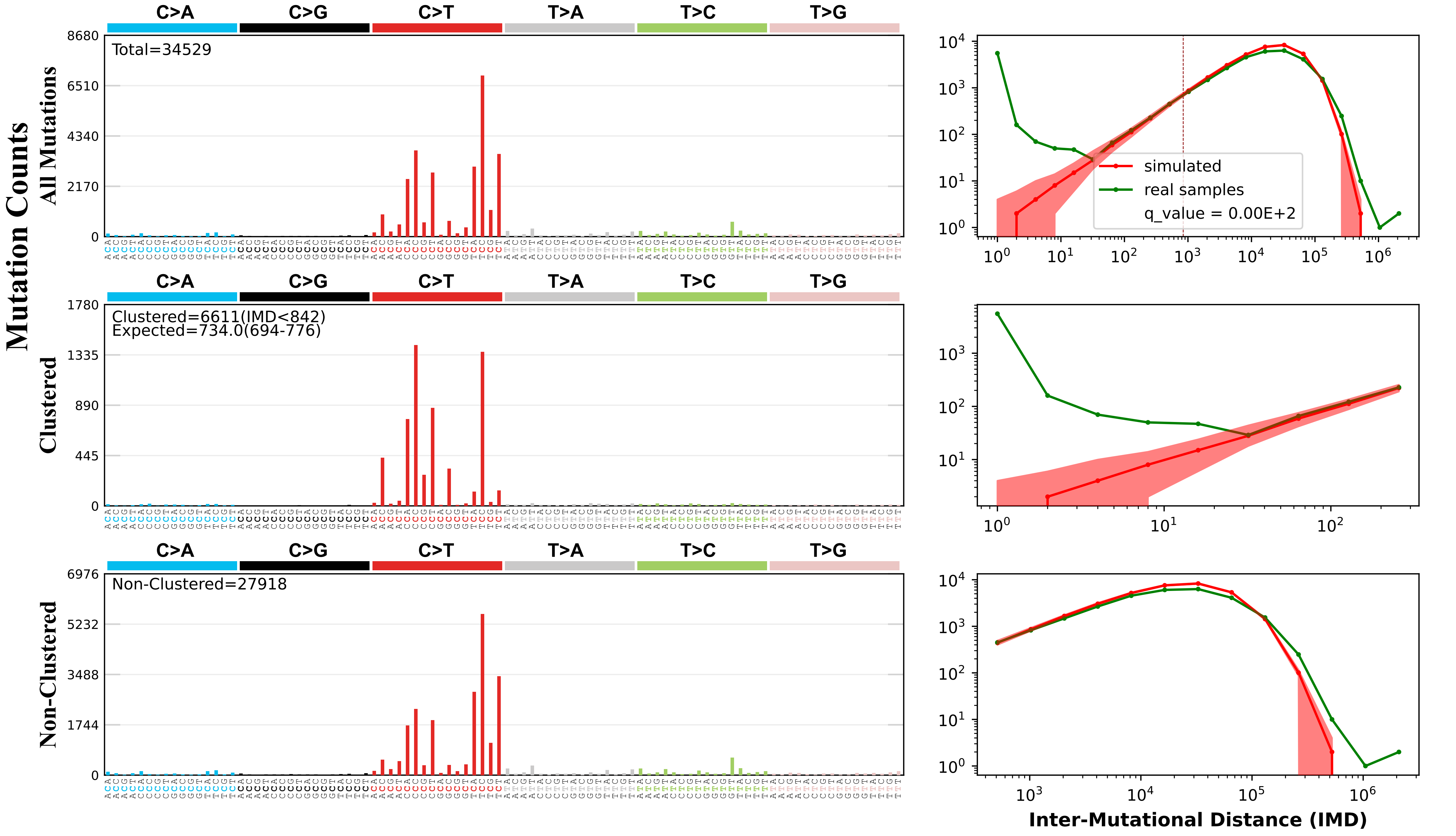

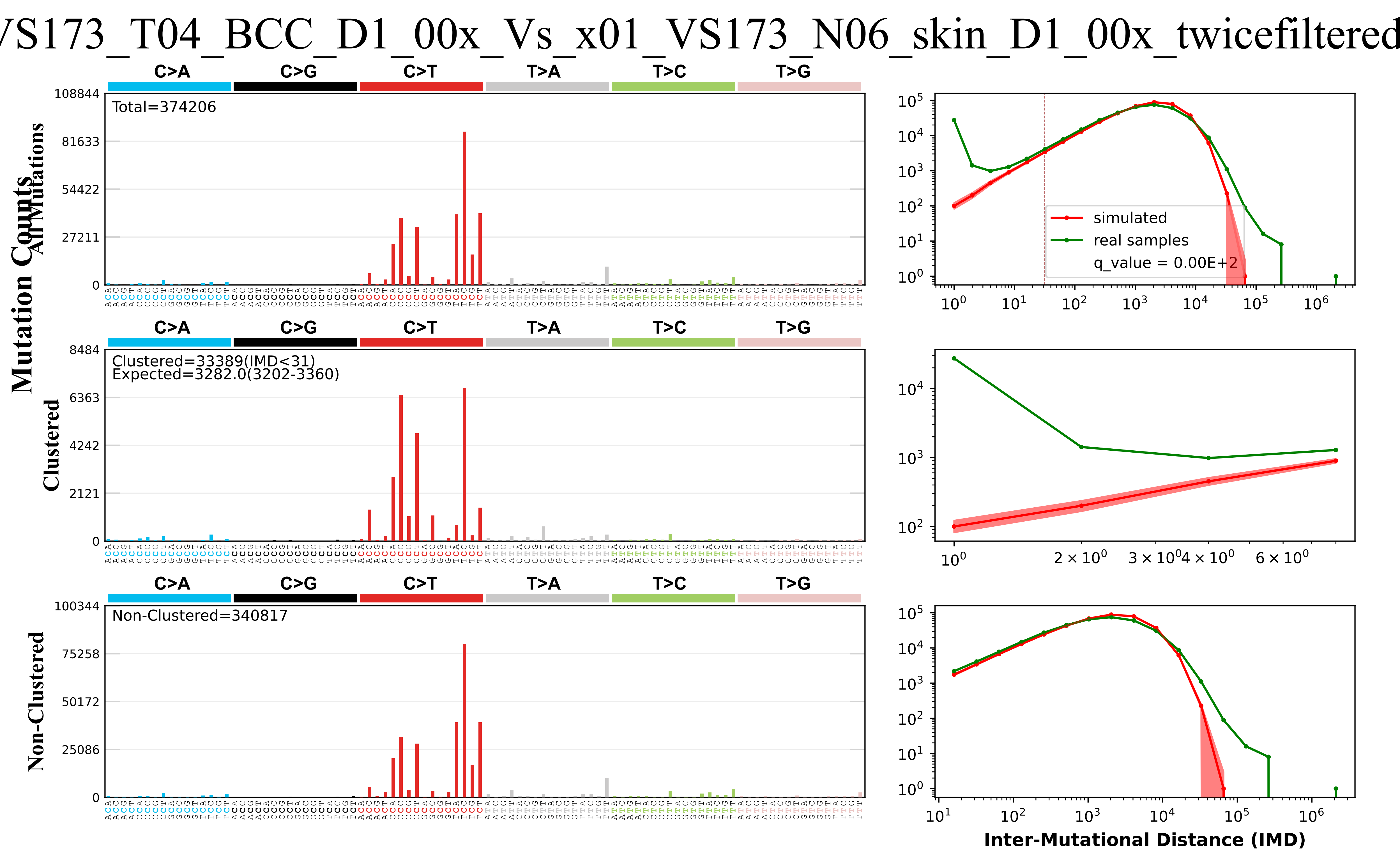

VS173_T04_BCC_D1_00x_Vs_x01_VS173_N06_skin_D1_00x_twicefiltered
Mutation Counts
All Mutations
C>A
C>G
C>T
T>A
T>C
T>G
108844
81633
54422
27211
0
Total=374206
simulated
real samples
q_value = 0.00E+2
Clustered
8484
6363
4242
2121
0
Clustered=33389(IMD<31)
Expected=3282.0(3202-3360)
Non-Clustered
100344
75258
50172
25086
0
Non-Clustered=340817
Inter-Mutational Distance (IMD)

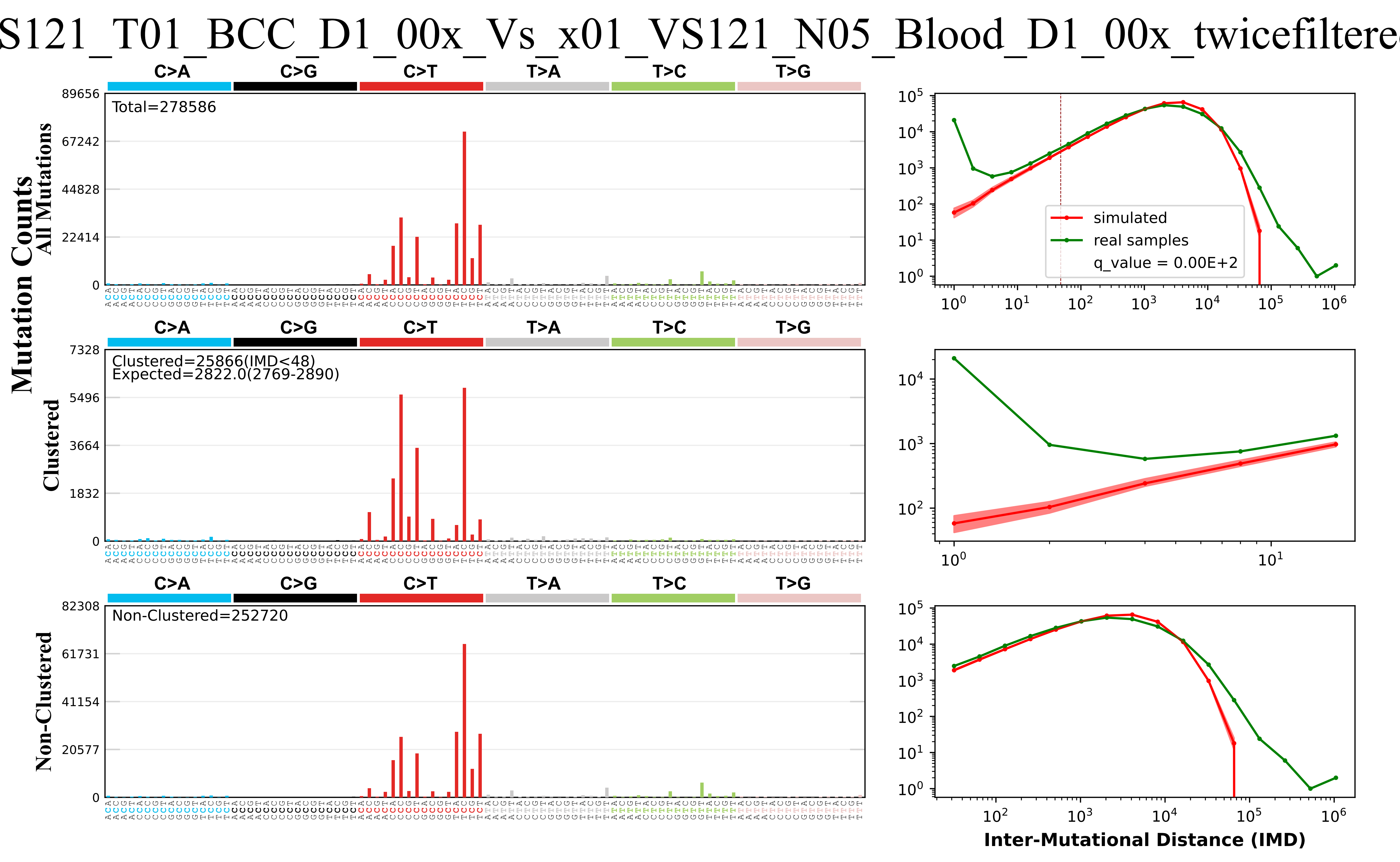
S121_T01_BCC_D1_00x_Vs_x01_VS121_N05_Blood_D1_00x_twicefilter
Mutation Counts
All Mutations
Clustered
Non-Clustered
C>A
C>G
C>T
T>A
T>C
T>G
Total=278586
89656
67242
44828
22414
0
Clustered=25866(IMD<48)
Expected=2822.0(2769-2890)
7328
5496
3664
1832
0
Non-Clustered=252720
82308
61731
41154
20577
0
simulated
real samples
q_value = 0.00E+2
Inter-Mutational Distance (IMD)

VS195_T01_BCC_D1_01x_Vs_x01_VS195_N02_skin_D1_00x_twicefiltered

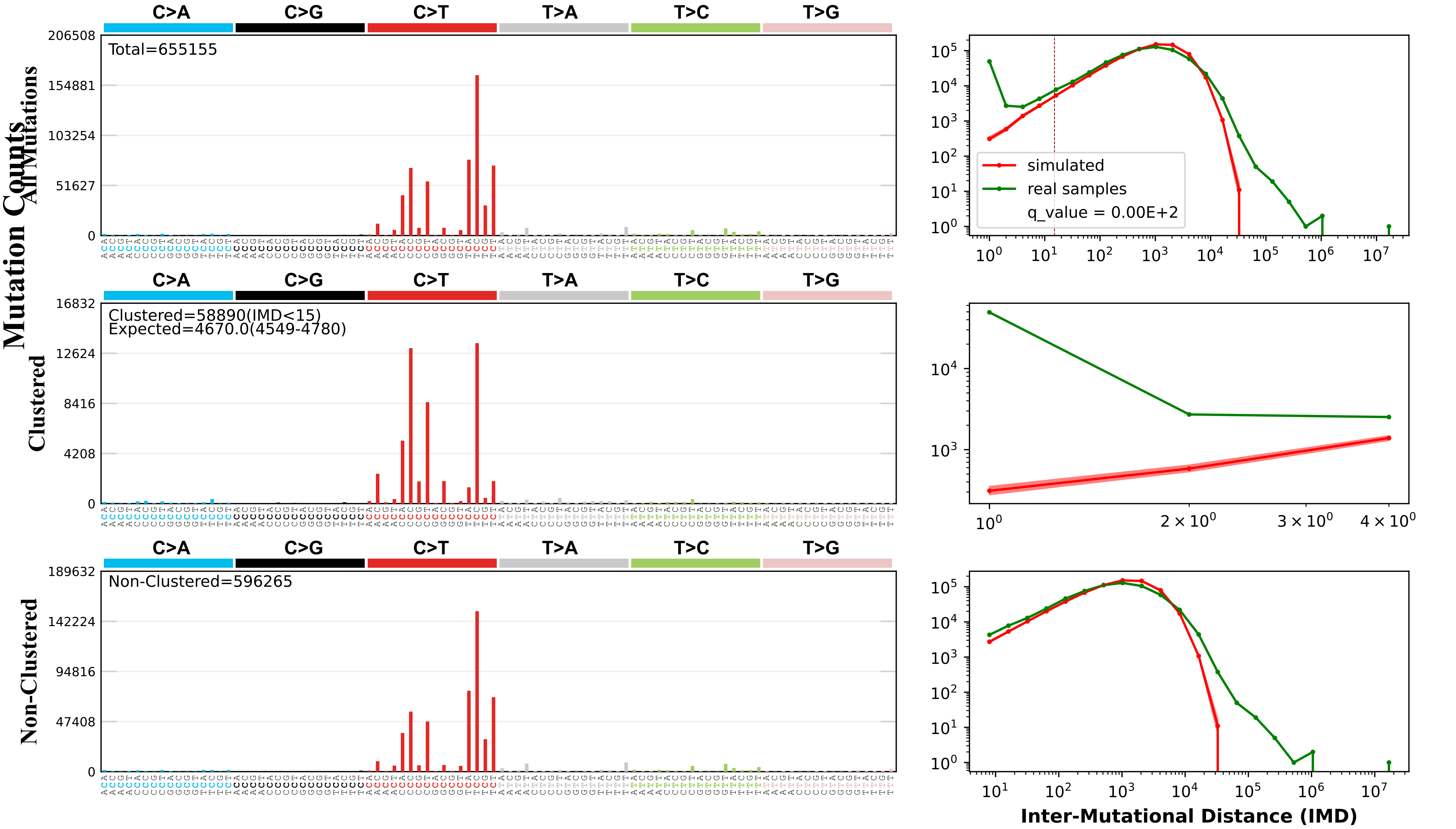

S140_T01_BCC_D1_00x_Vs_x01_VS140_N05_Blood_D1_00x_twicefiltere

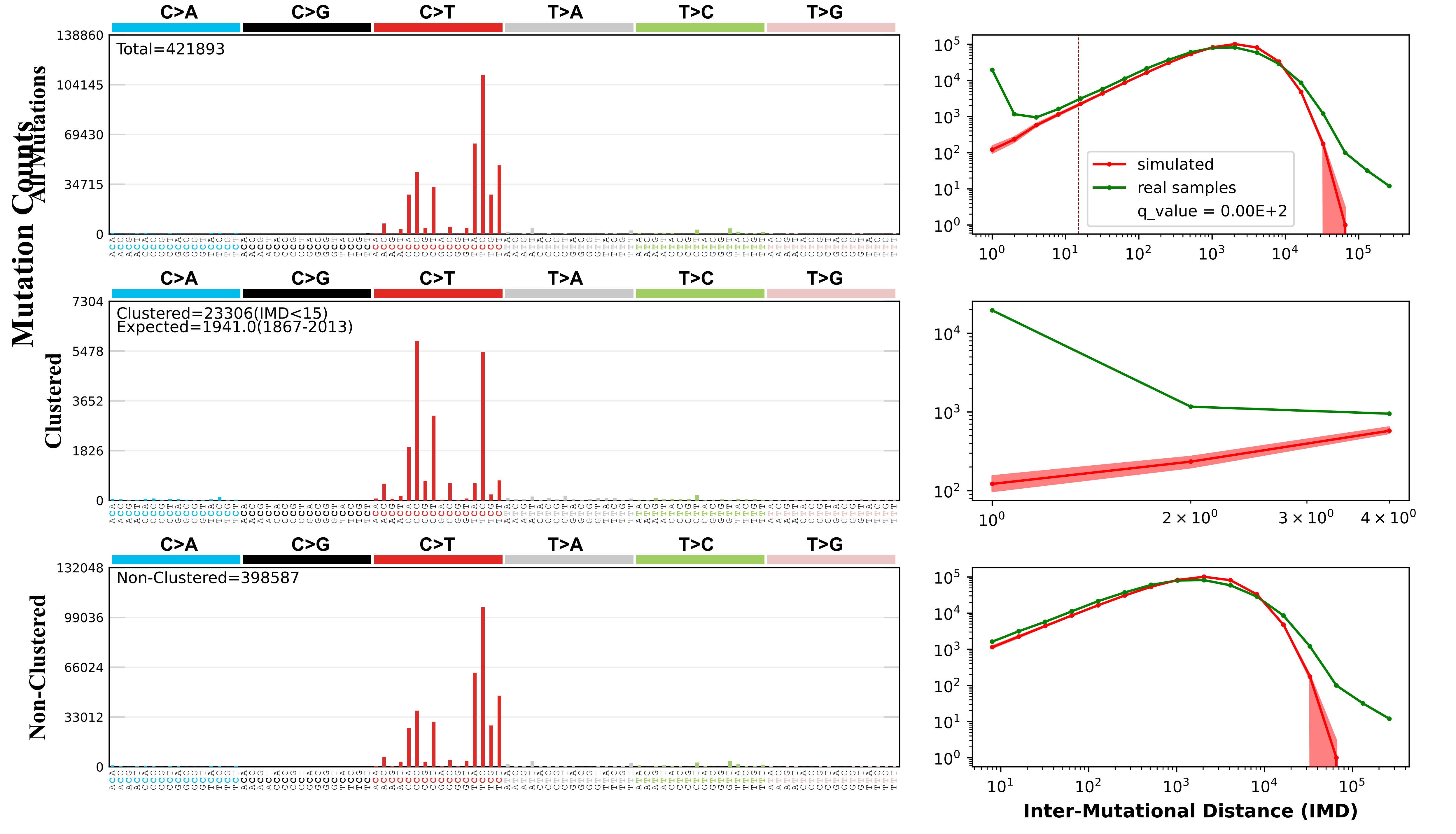

VS173_T05_BCC_D1_01x_Vs_x01_VS173_N06_skin_D1_00x_twicefiltered

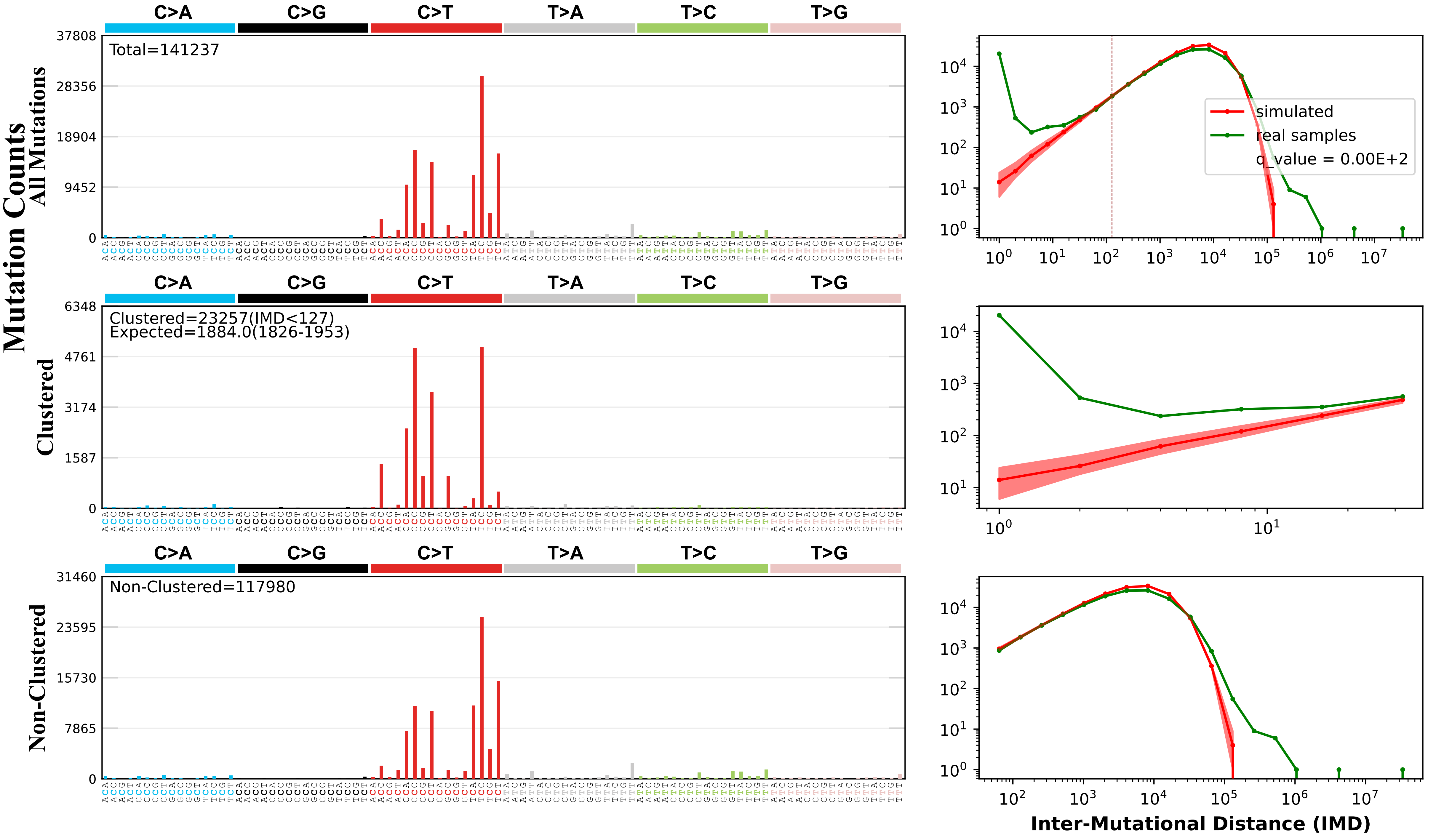

S009_T07_BCC_D1_00x_Vs_x01_DS009_N10_blood_D2_00x_twicefiltere

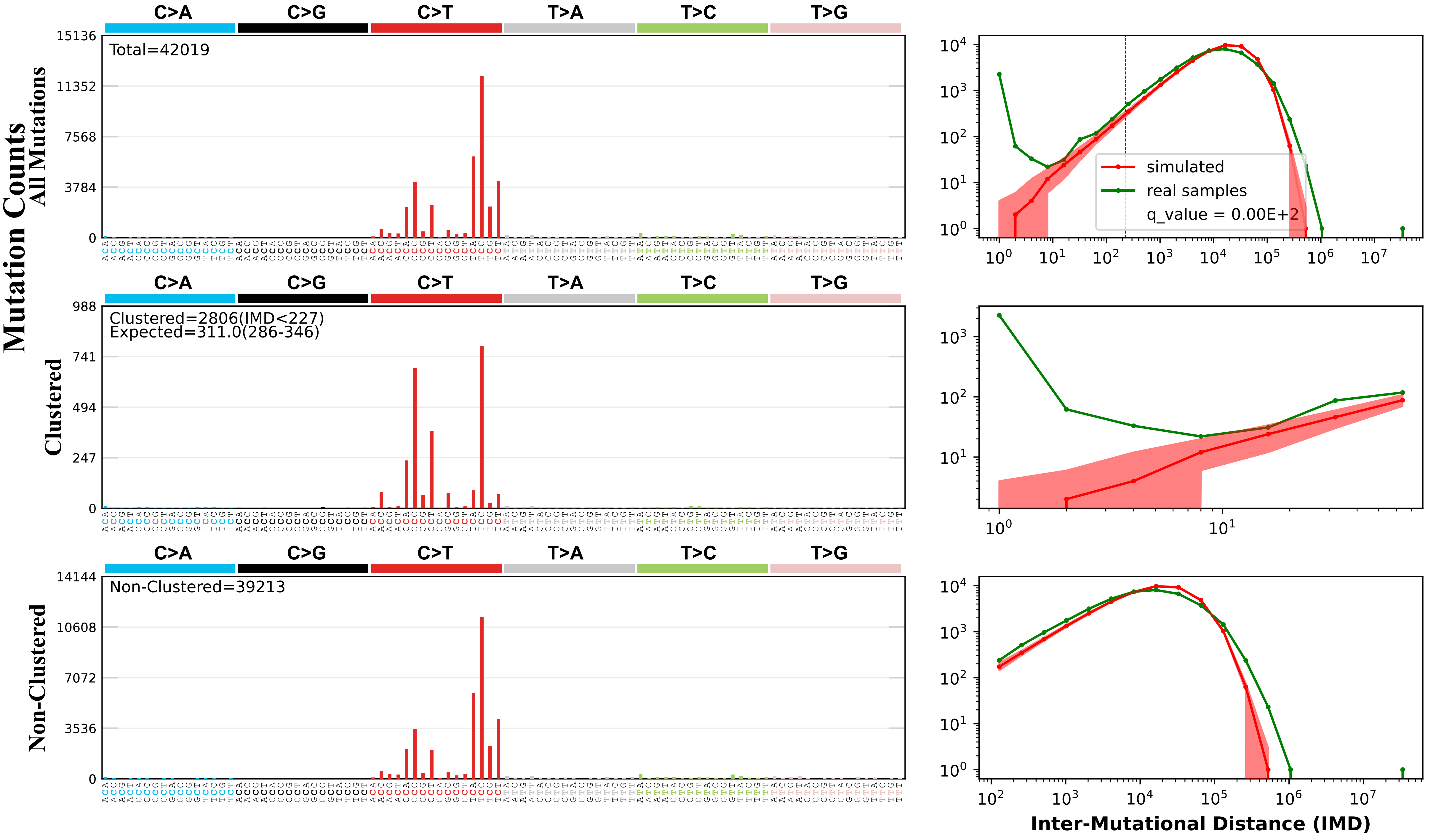

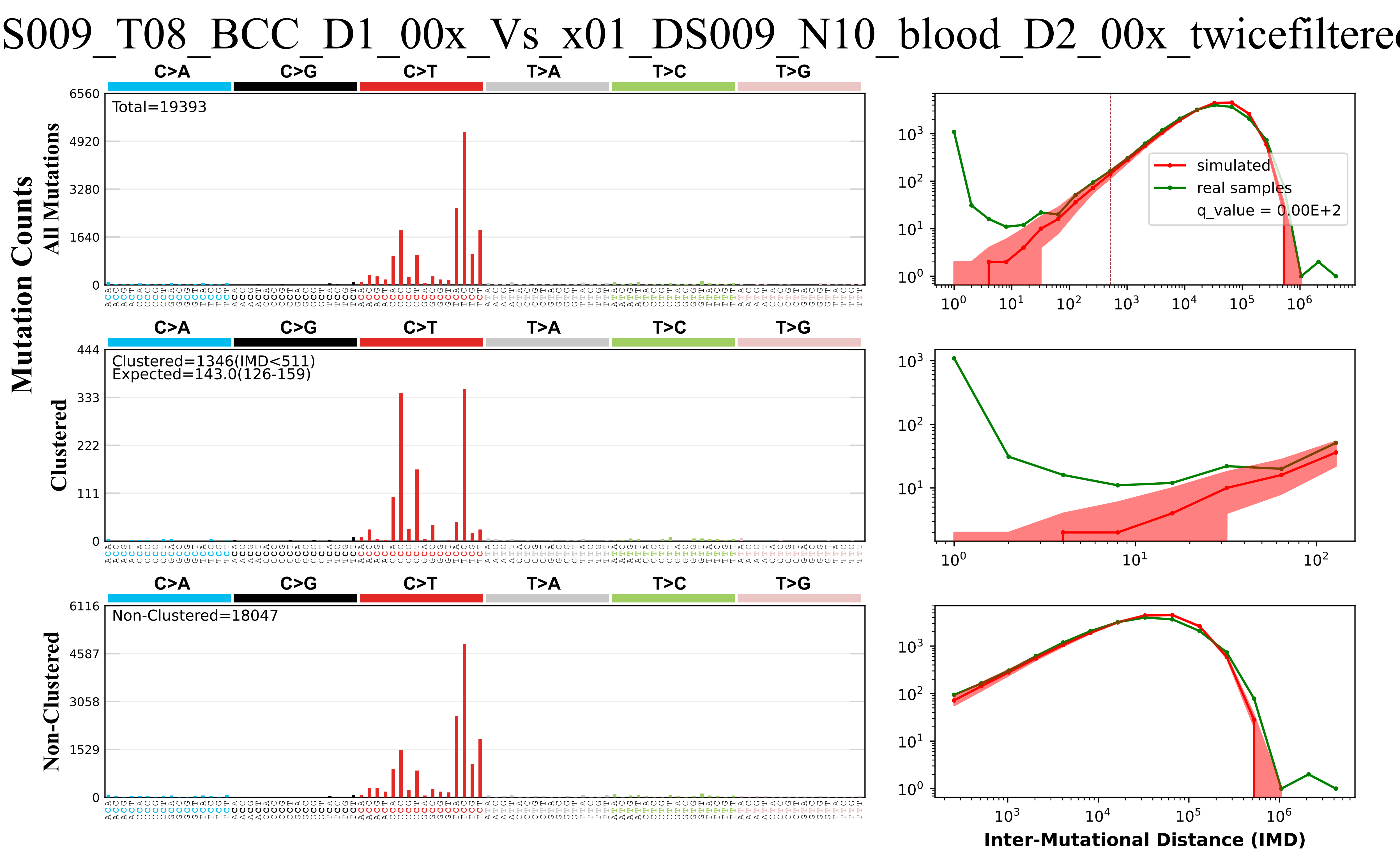

S009_T08_BCC_D1_00x_Vs_x01_DS009_N10_blood_D2_00x_twicefiltere
Mutation Counts
All Mutations
C>A
C>G
C>T
T>A
T>C
T>G
Total=19393
6560
4920
3280
1640
0
Clustered
Clustered=1346(IMD<511)
Expected=143.0(126-159)
444
333
222
111
0
Non-Clustered
Non-Clustered=18047
6116
4587
3058
1529
0
simulated
real samples
q_value = 0.00E+2
Inter-Mutational Distance (IMD)

VS190_T02_BCC_D1_00x_Vs_x01_VS190_N06_skin_D1_00x_twicefiltered

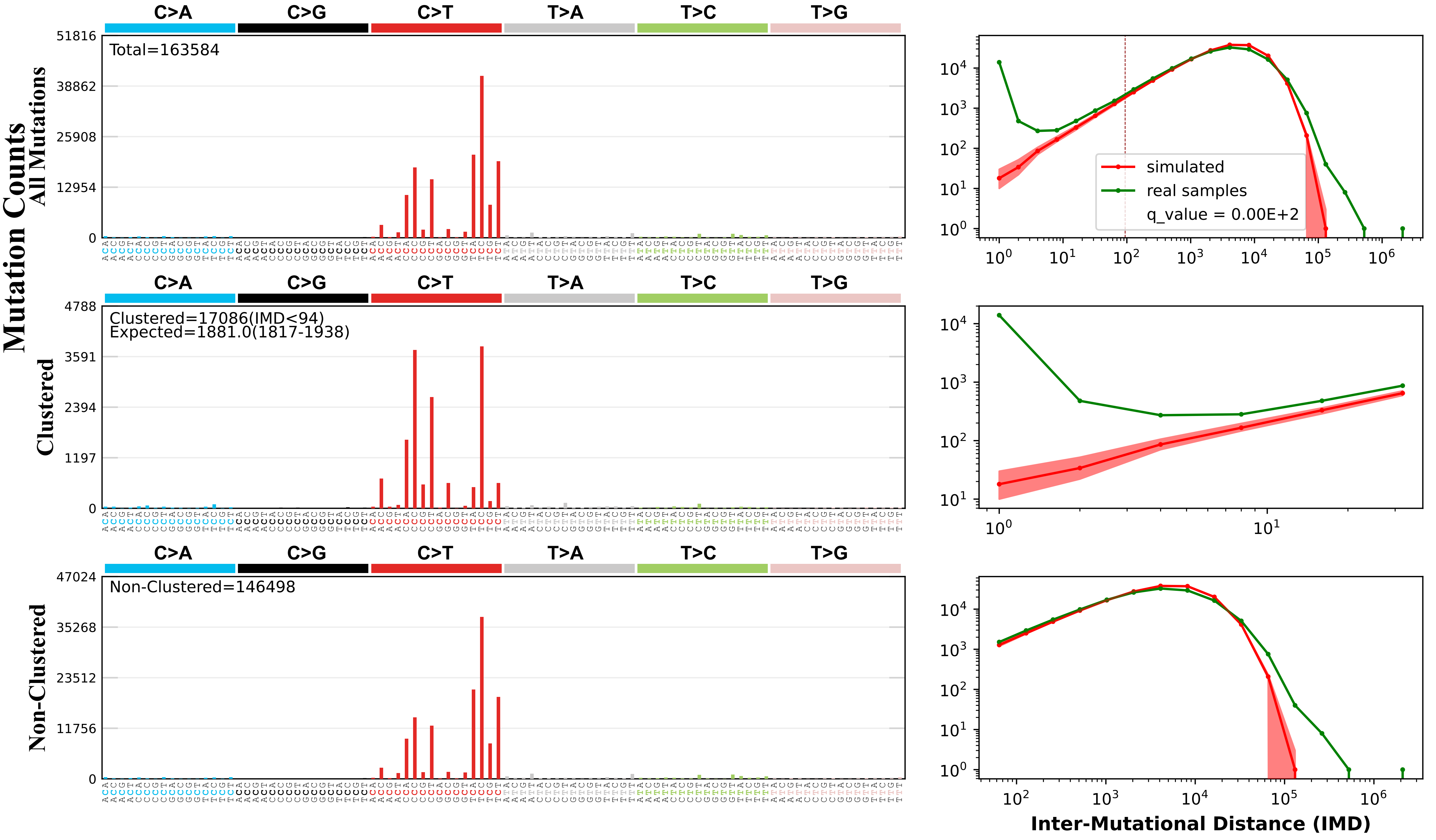

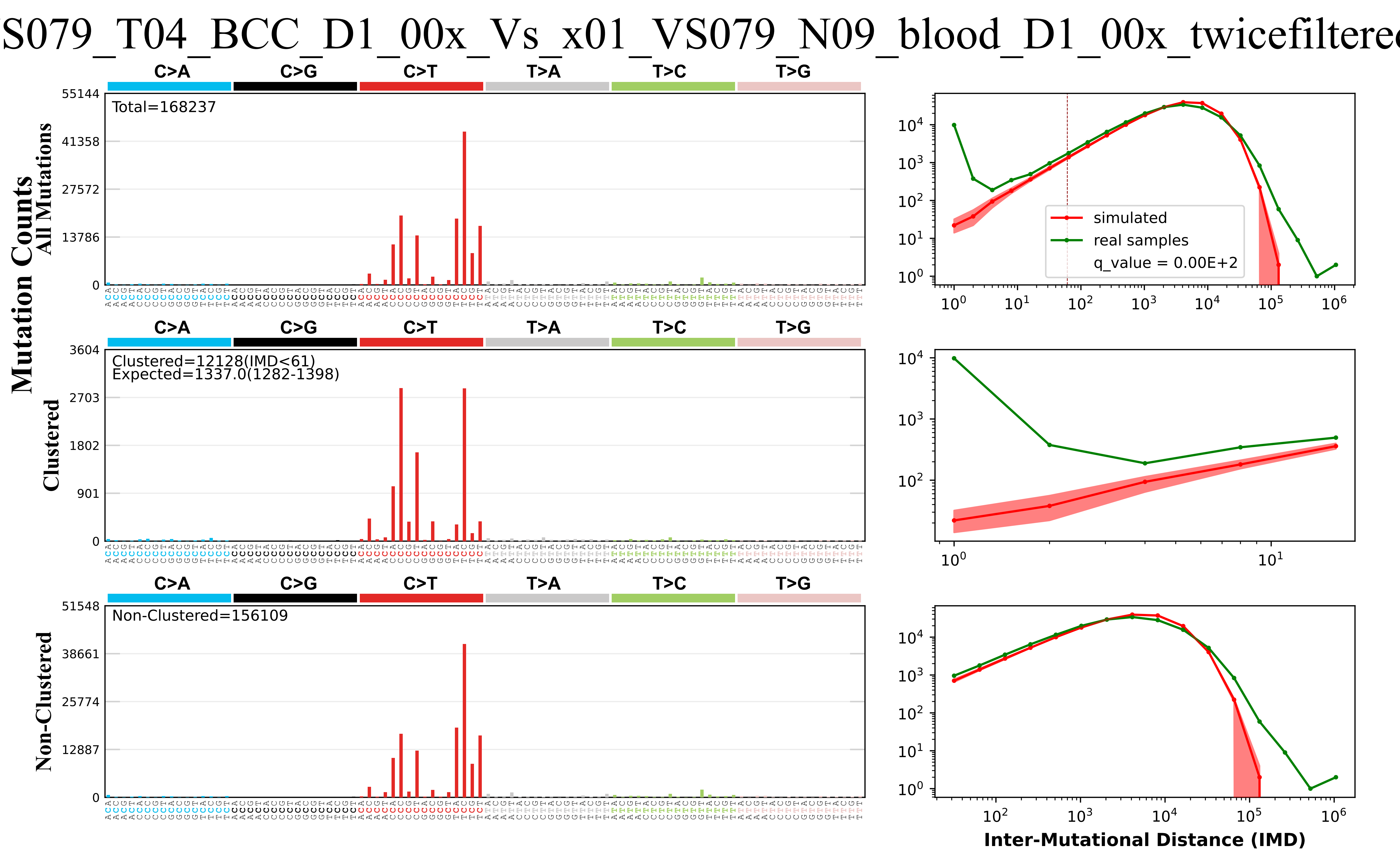

S079_T04_BCC_D1_00x_Vs_x01_VS079_N09_blood_D1_00x_twicefiltere
Mutation Counts
All Mutations
Total=168237
C>A
C>G
C>T
T>A
T>C
T>G
Clustered
Clustered=12128(IMD<61)
Expected=1337.0(1282-1398)
Non-Clustered
Non-Clustered=156109
simulated
real samples
q_value = 0.00E+2
Inter-Mutational Distance (IMD)

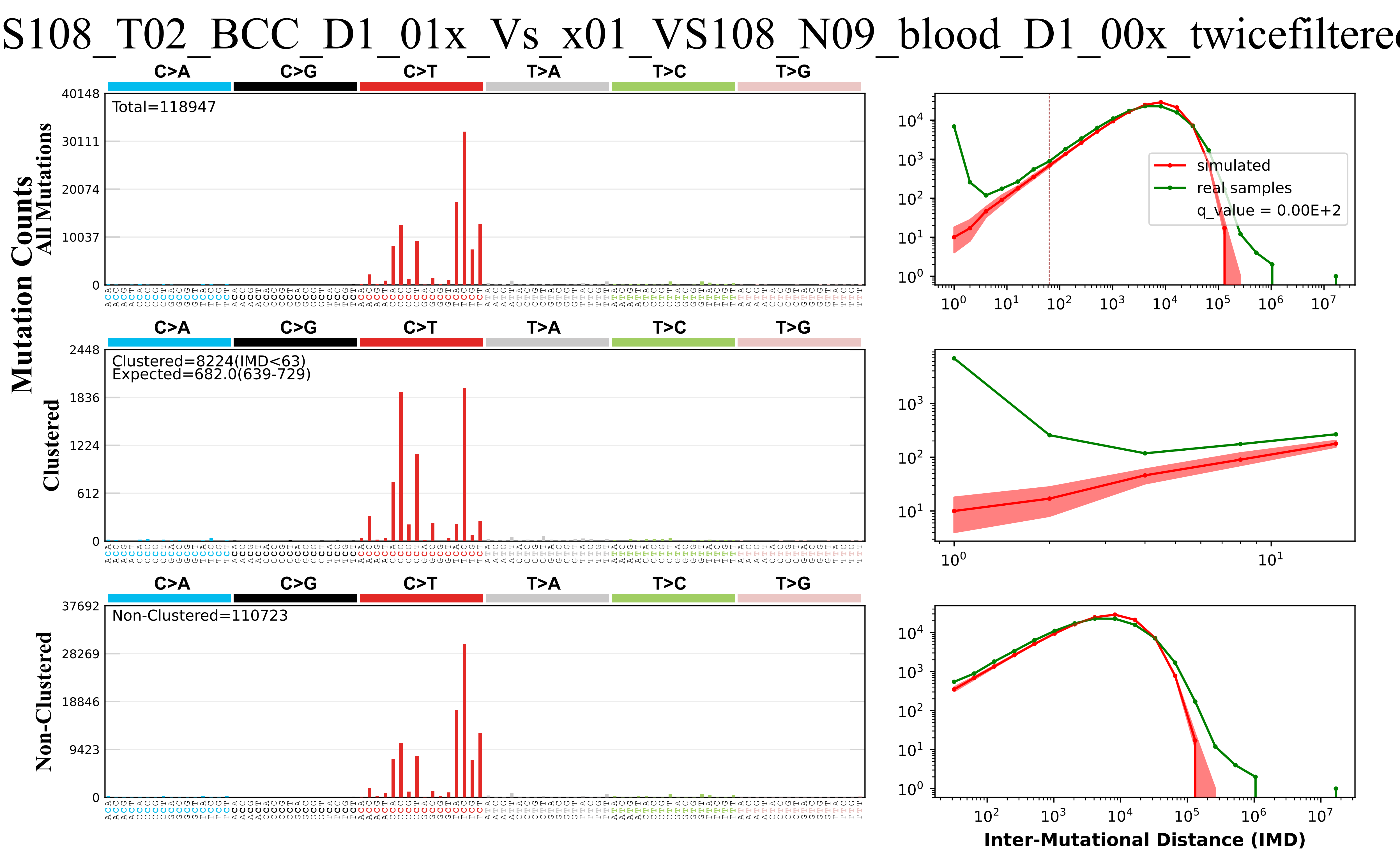

S108_T02_BCC_D1_01x_Vs_x01_VS108_N09_blood_D1_00x_twicefiltere
Mutation Counts
All Mutations
Clustered
Non-Clustered
C>A
C>G
C>T
T>A
T>C
T>G
Total=118947
Clustered=8224(IMD<63)
Expected=682.0(639-729)
Non-Clustered=110723
simulated
real samples
q_value = 0.00E+2
Inter-Mutational Distance (IMD)

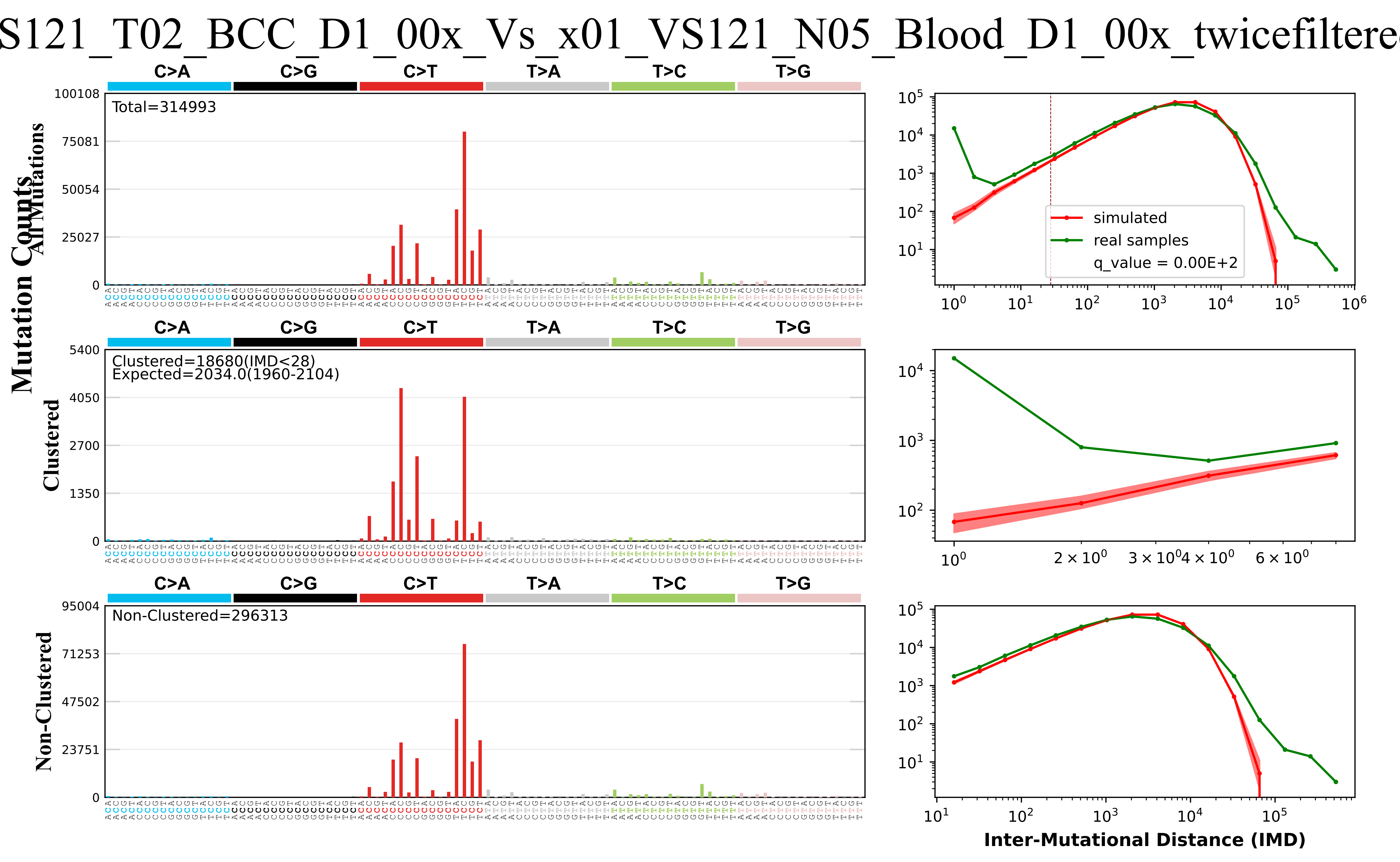
S121_T02_BCC_D1_00x_Vs_x01_VS121_N05_Blood_D1_00x_twicefilter
Mutation Counts
All Mutations
Clustered
Non-Clustered
C>A
C>G
C>T
T>A
T>C
T>G
Total=314993
100108
75081
50054
25027
0
Clustered=18680(IMD<28)
Expected=2034.0(1960-2104)
5400
4050
2700
1350
0
Non-Clustered=296313
95004
71253
47502
23751
0
simulated
real samples
q_value = 0.00E+2
Inter-Mutational Distance (IMD)

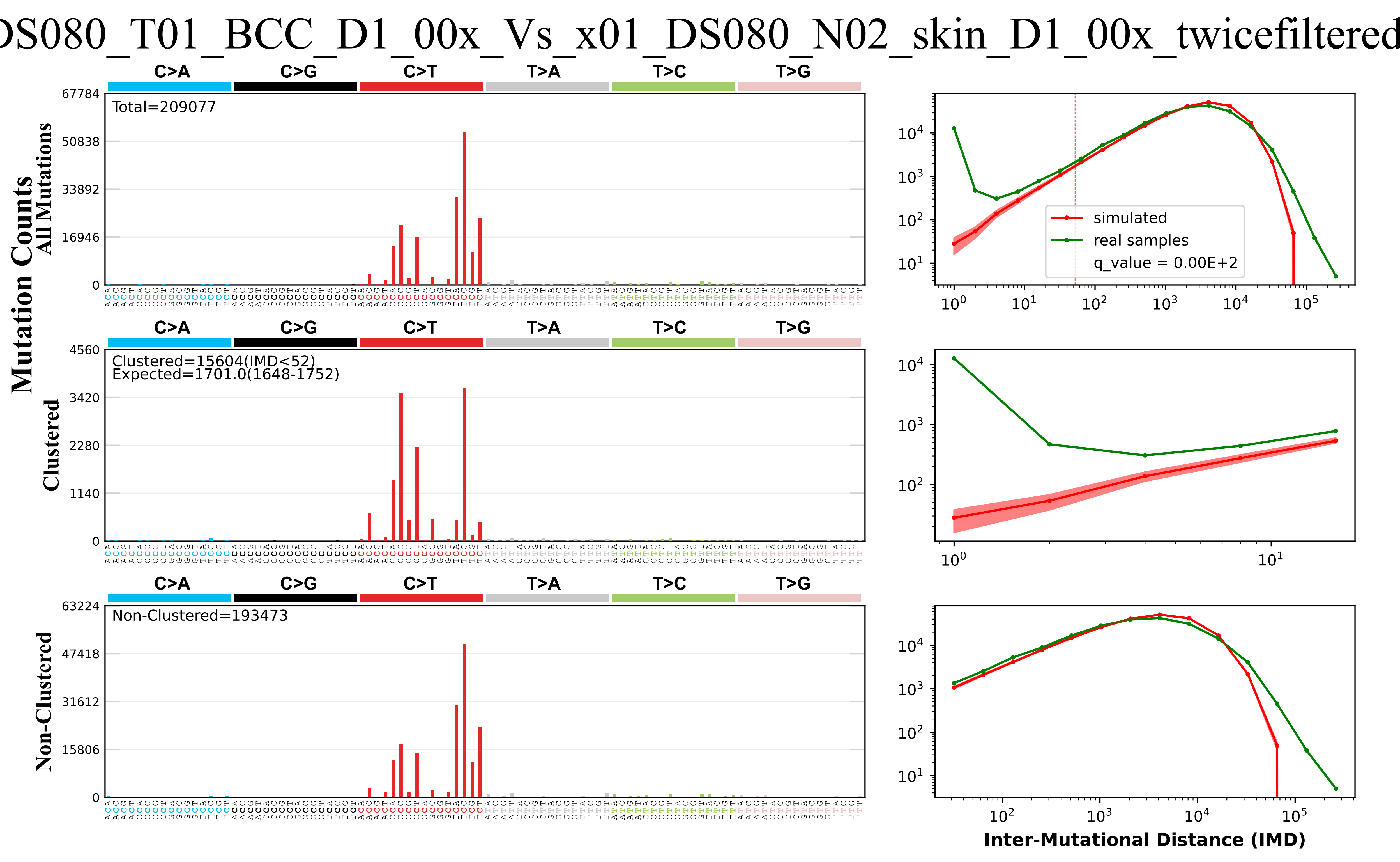

DS080_T01_BCC_D1_00x_Vs_x01_DS080_N02_skin_D1_00x_twicefiltered
Mutation Counts
All Mutations
Clustered
Non-Clustered
C>A
C>G
C>T
T>A
T>C
T>G
Total=209077
Clustered=15604(IMD<52)
Expected=1701.0(1648-1752)
Non-Clustered=193473
simulated
real samples
q_value = 0.00E+2
Inter-Mutational Distance (IMD)

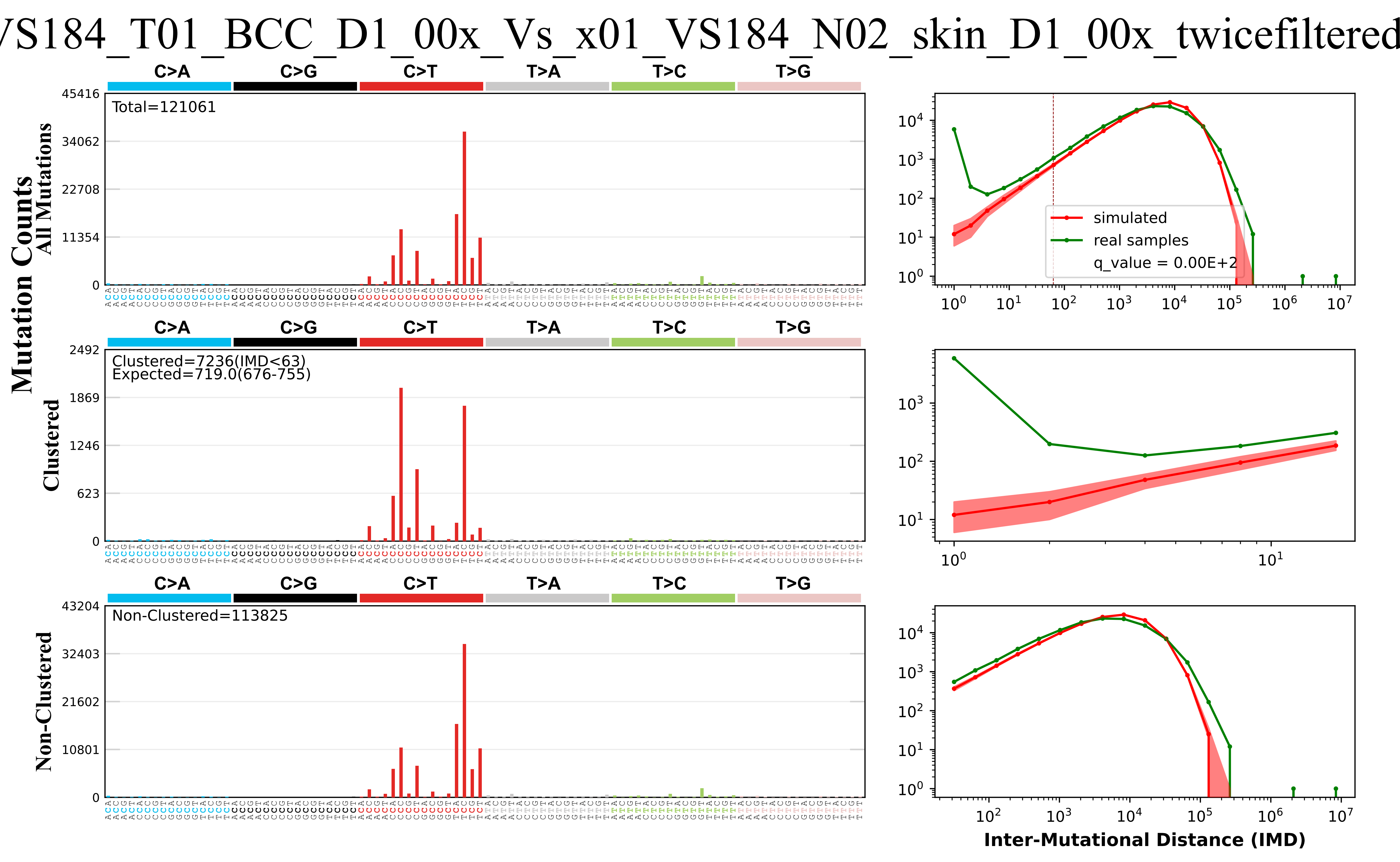
VS184_T01_BCC_D1_00x_Vs_x01_VS184_N02_skin_D1_00x_twicefiltered
Mutation Counts
All Mutations
Clustered
Non-Clustered
C>A
C>G
C>T
T>A
T>C
T>G
Total=121061
Clustered=7236(IMD<63)
Expected=719.0(676-755)
Non-Clustered=113825
simulated
real samples
q_value = 0.00E+2
Inter-Mutational Distance (IMD)

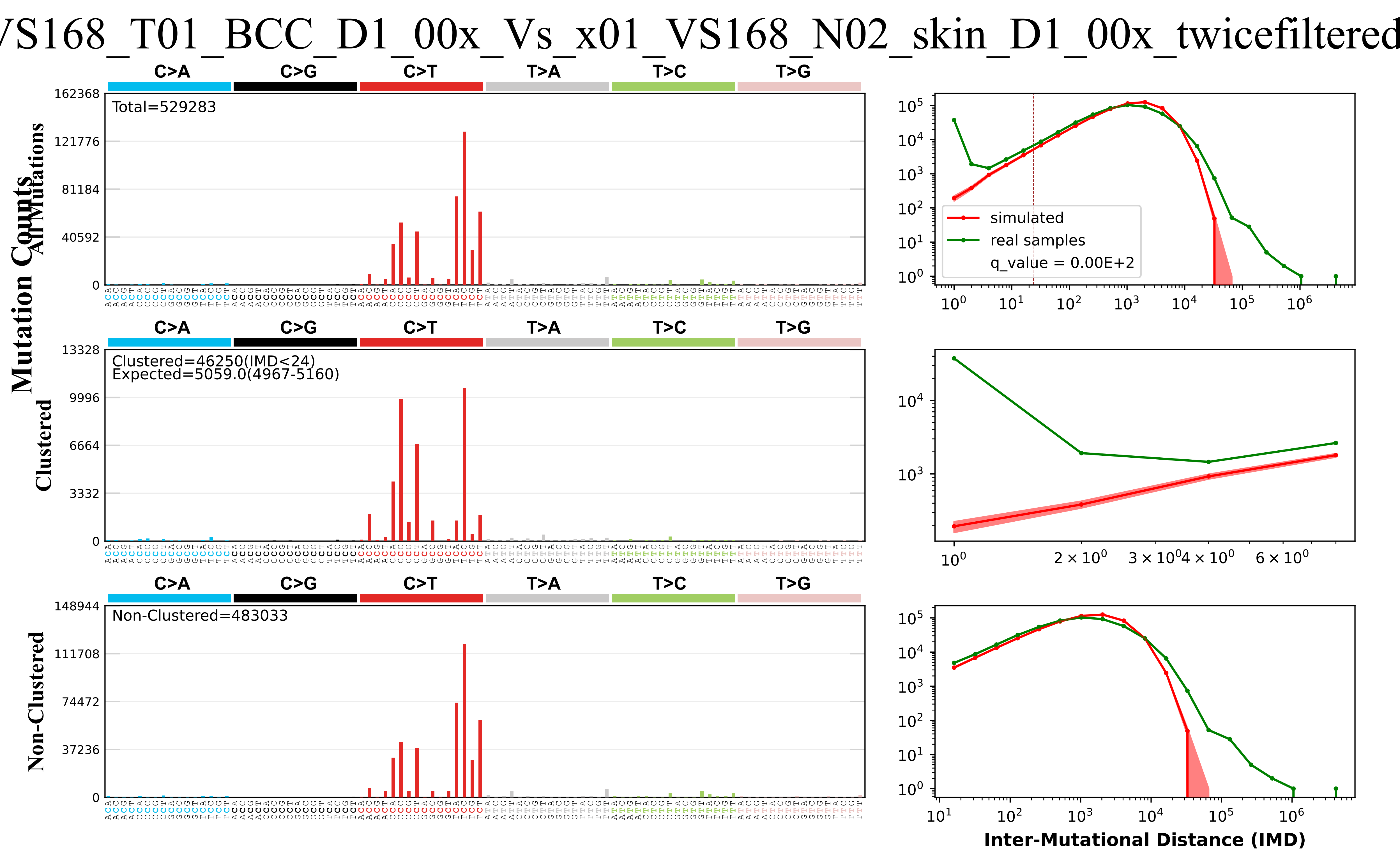

VS168_T01_BCC_D1_00x_Vs_x01_VS168_N02_skin_D1_00x_twicefiltered
Mutation Counts
All Mutations
Total=529283
Clustered
Clustered=46250(IMD<24)
Expected=5059.0(4967-5160)
Non-Clustered
Non-Clustered=483033
C>A
C>G
C>T
T>A
T>C
T>G
simulated
real samples
q_value = 0.00E+2
Inter-Mutational Distance (IMD)

DRUP01080021T_Vs_DRUP01080021R_twicefiltered_TvN

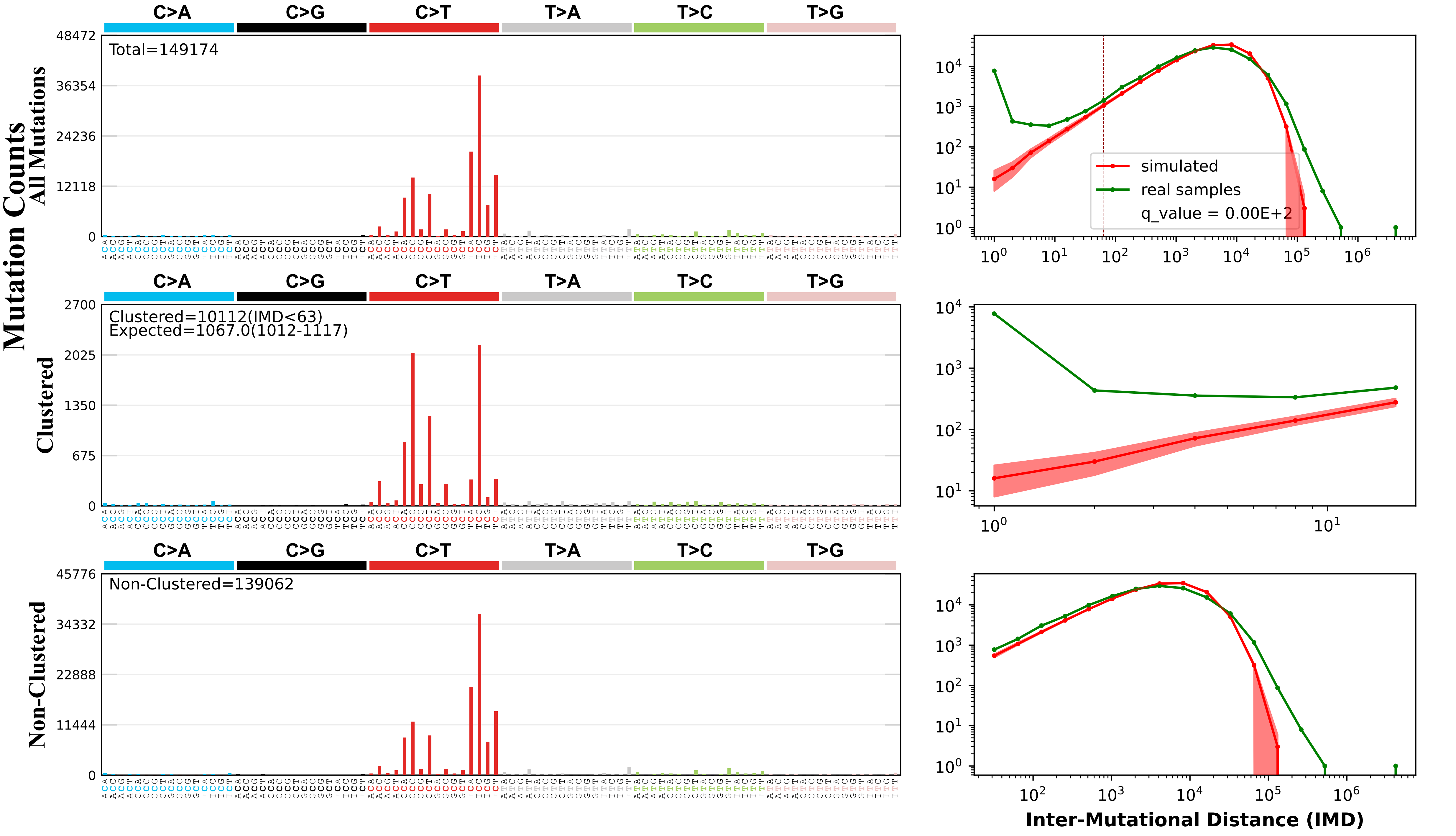

DRUP01080022T_Vs_DRUP01080022R_twicefiltered_TvN

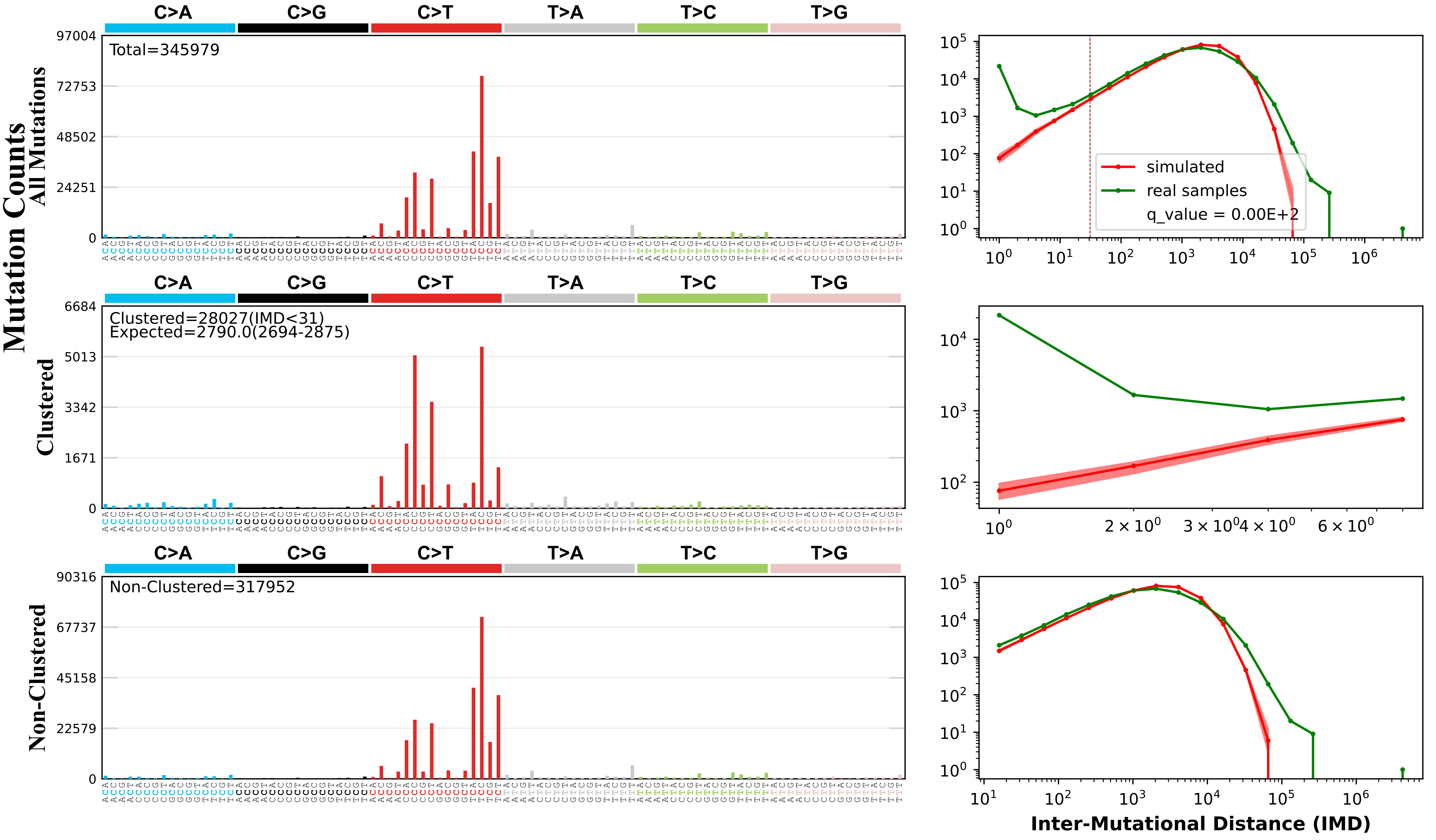

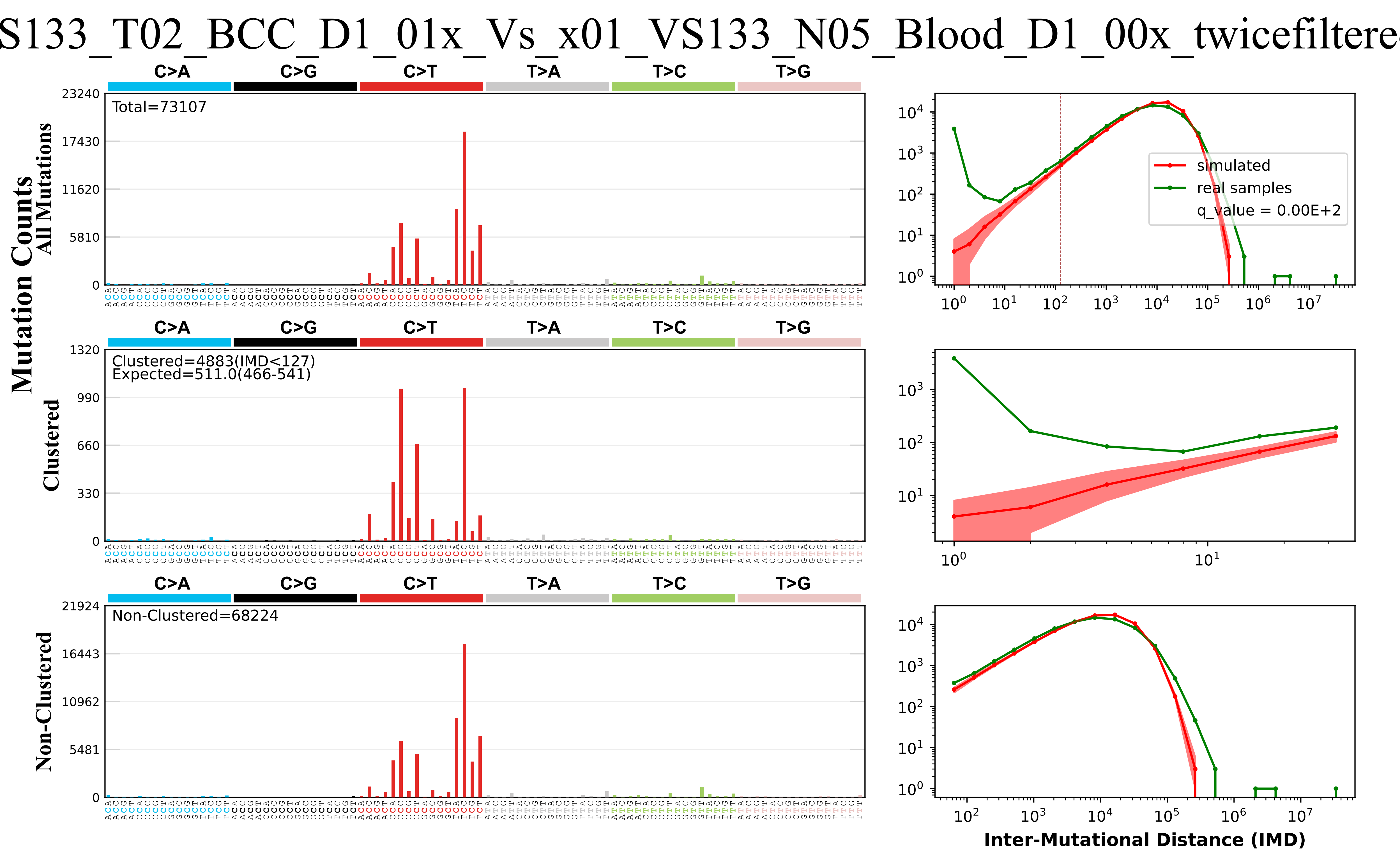

S133_T02_BCC_D1_01x_Vs_x01_VS133_N05_Blood_D1_00x_twicefiltere
Mutation Counts
All Mutations
Clustered
Non-Clustered
C>A
C>G
C>T
T>A
T>C
T>G
Total=73107
Clustered=4883(IMD<127)
Expected=511.0(466-541)
Non-Clustered=68224
simulated
real samples
q_value = 0.00E+2
Inter-Mutational Distance (IMD)

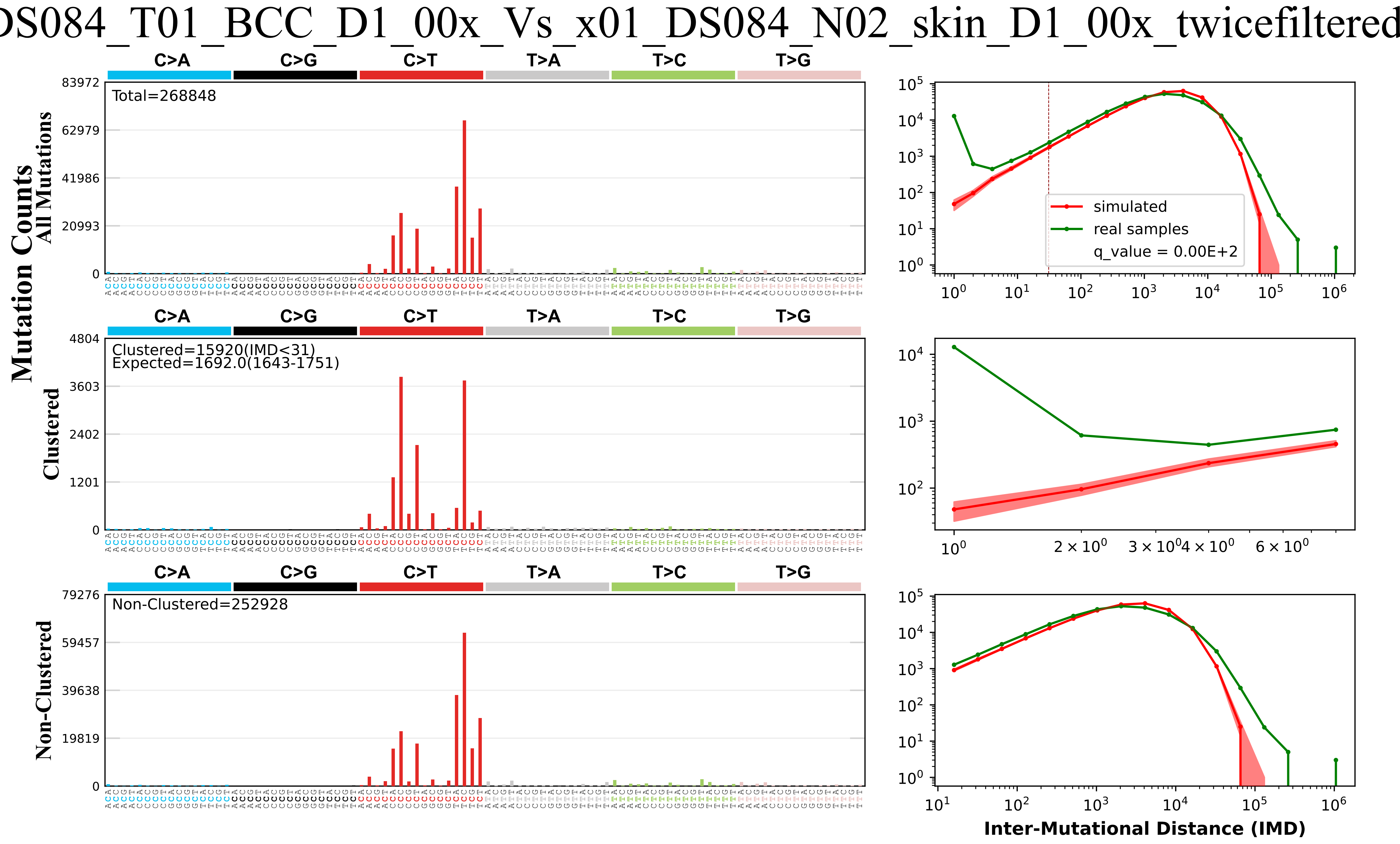
DS084_T01_BCC_D1_00x_Vs_x01_DS084_N02_skin_D1_00x_twicefiltered
Mutation Counts
All Mutations
Clustered
Non-Clustered
C>A
C>G
C>T
T>A
T>C
T>G
Total=268848
Clustered=15920(IMD<31)
Expected=1692.0(1643-1751)
Non-Clustered=252928
simulated
real samples
q_value = 0.00E+2
Inter-Mutational Distance (IMD)

CPCT02030459TII_Vs_CPCT02030459R_twicefiltered_TvN

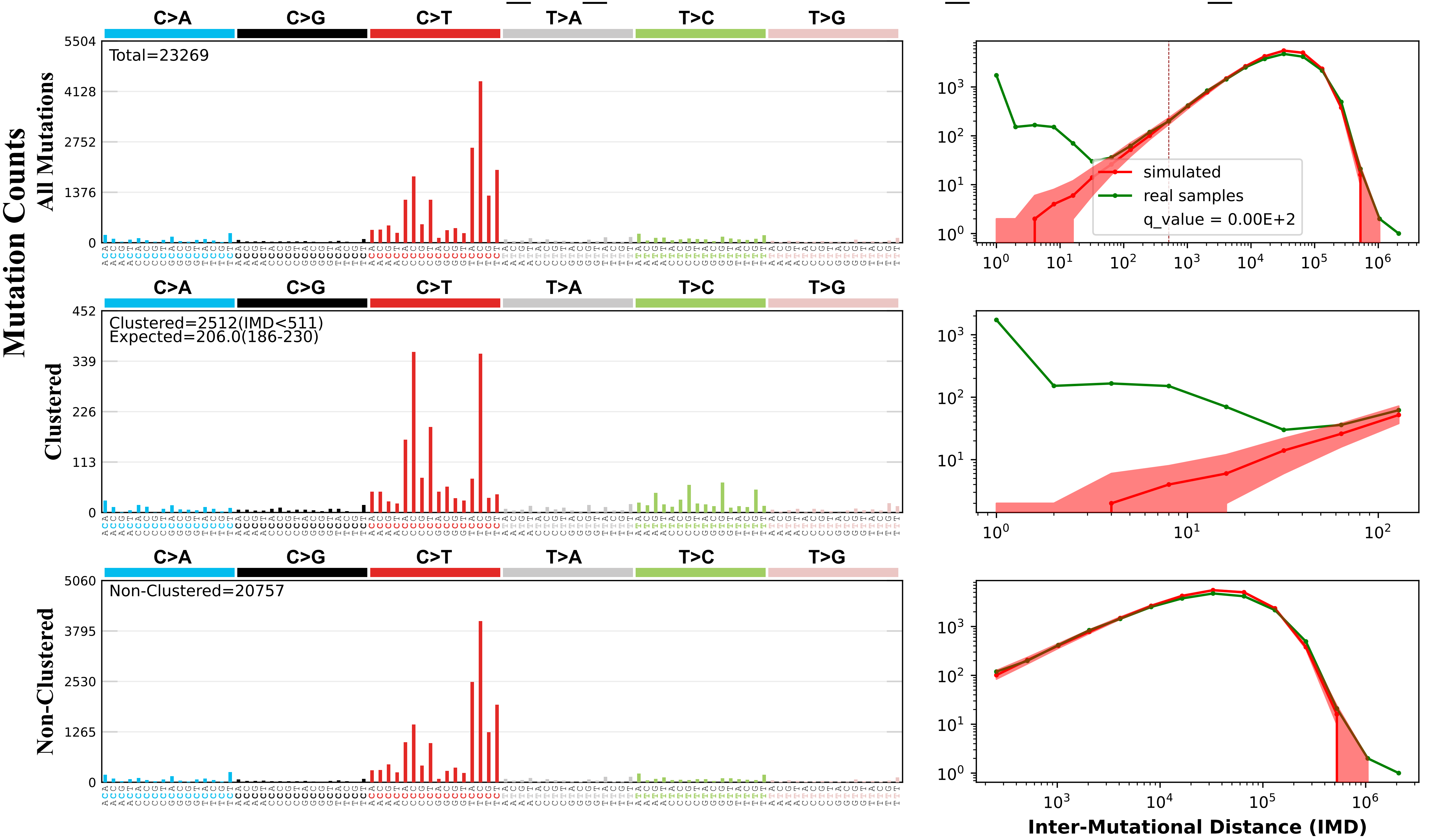

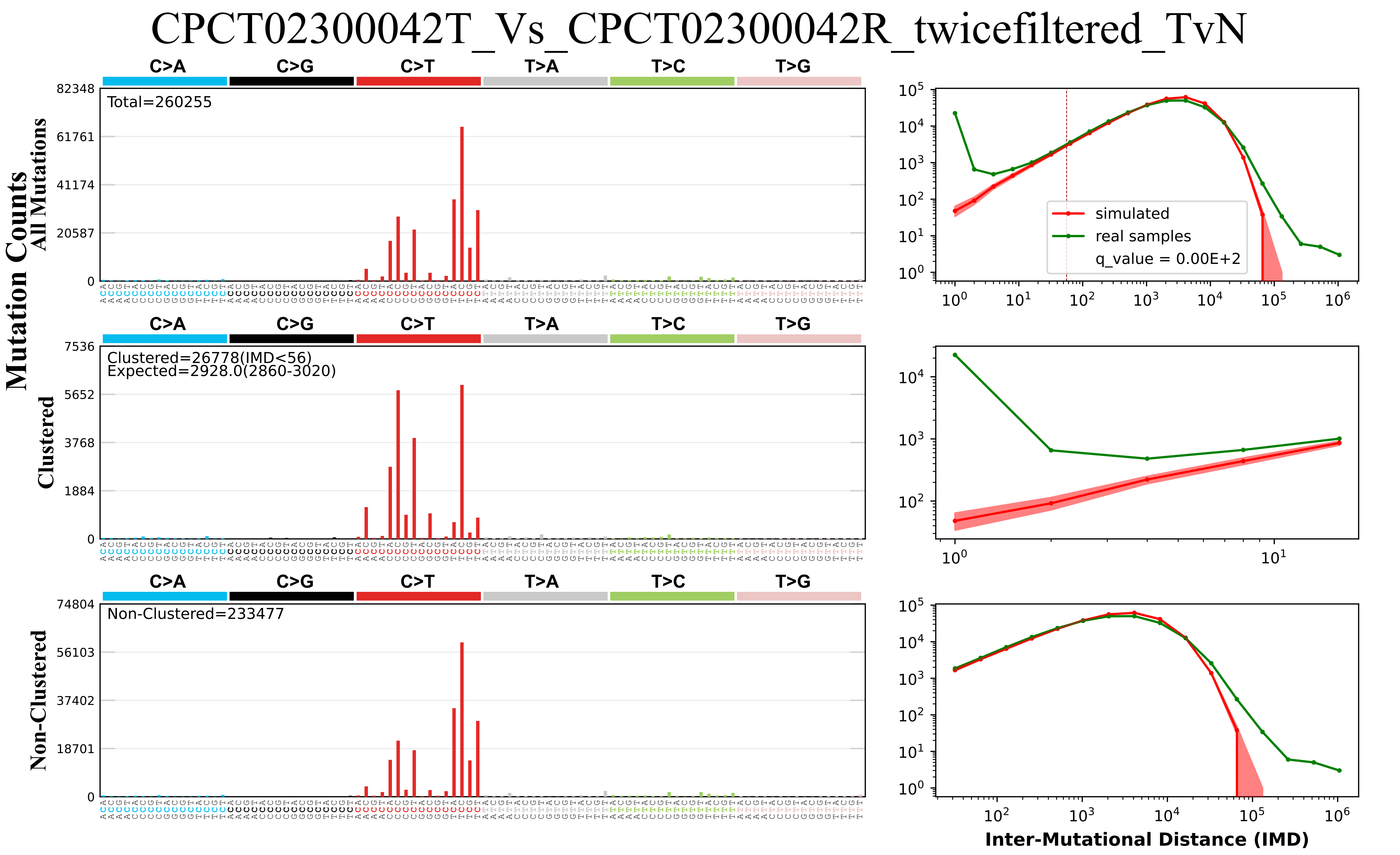
CPCT02300042T_Vs_CPCT02300042R_twicefiltered_TvN
Mutation Counts
All Mutations
Clustered
Non-Clustered
C>A
C>G
C>T
T>A
T>C
T>G
Total=260255
Clustered=26778(IMD<56)
Expected=2928.0(2860-3020)
Non-Clustered=233477
simulated
real samples
q_value = 0.00E+2
Inter-Mutational Distance (IMD)

DS103_T01_BCC_D1_00x_Vs_x01_DS103_N02_skin_D1_00x_twicefiltered

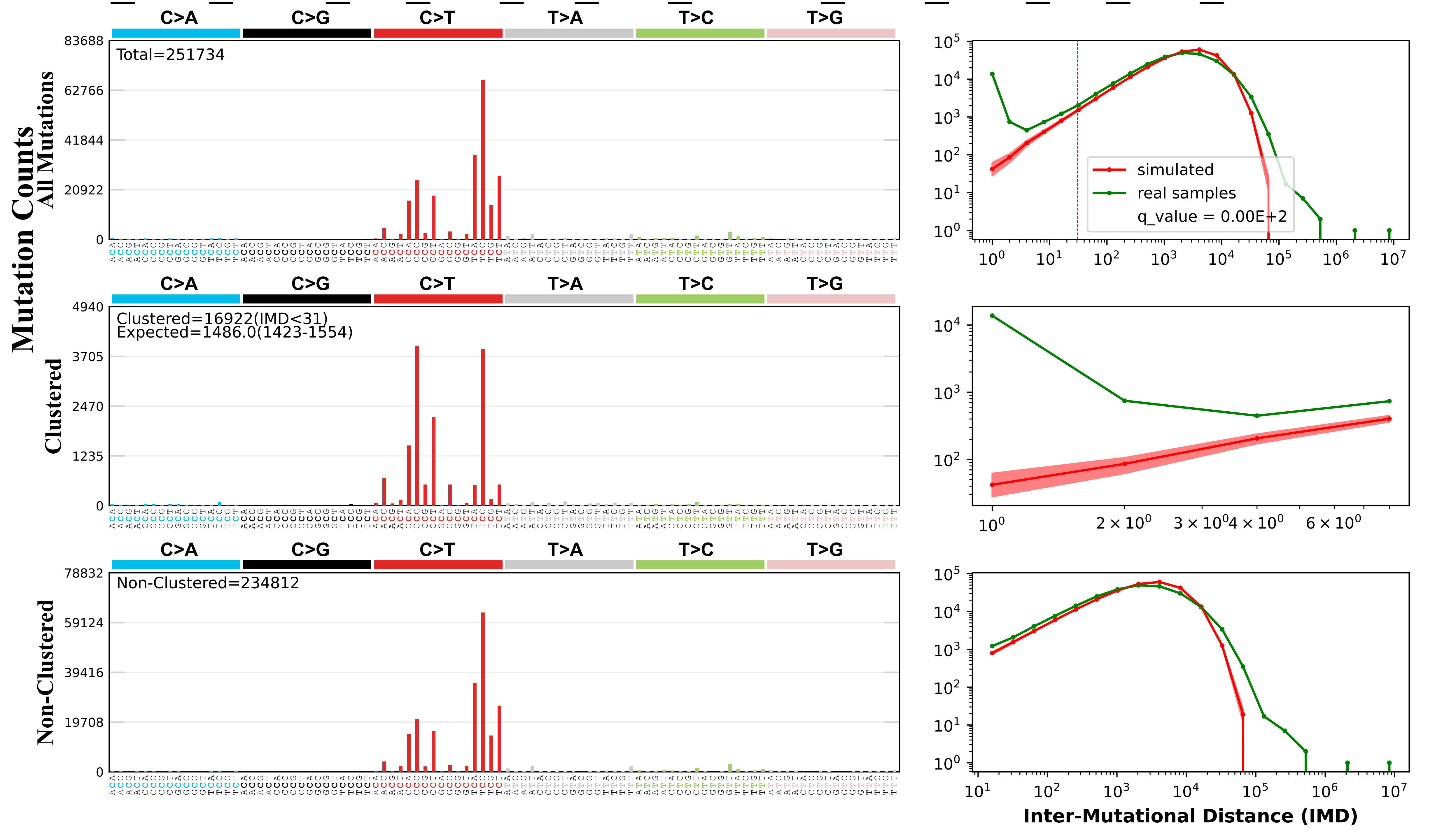

VS112_T01_BCC_D1_00x_Vs_x01_VS112_N02_skin_D1_00x_twicefiltered

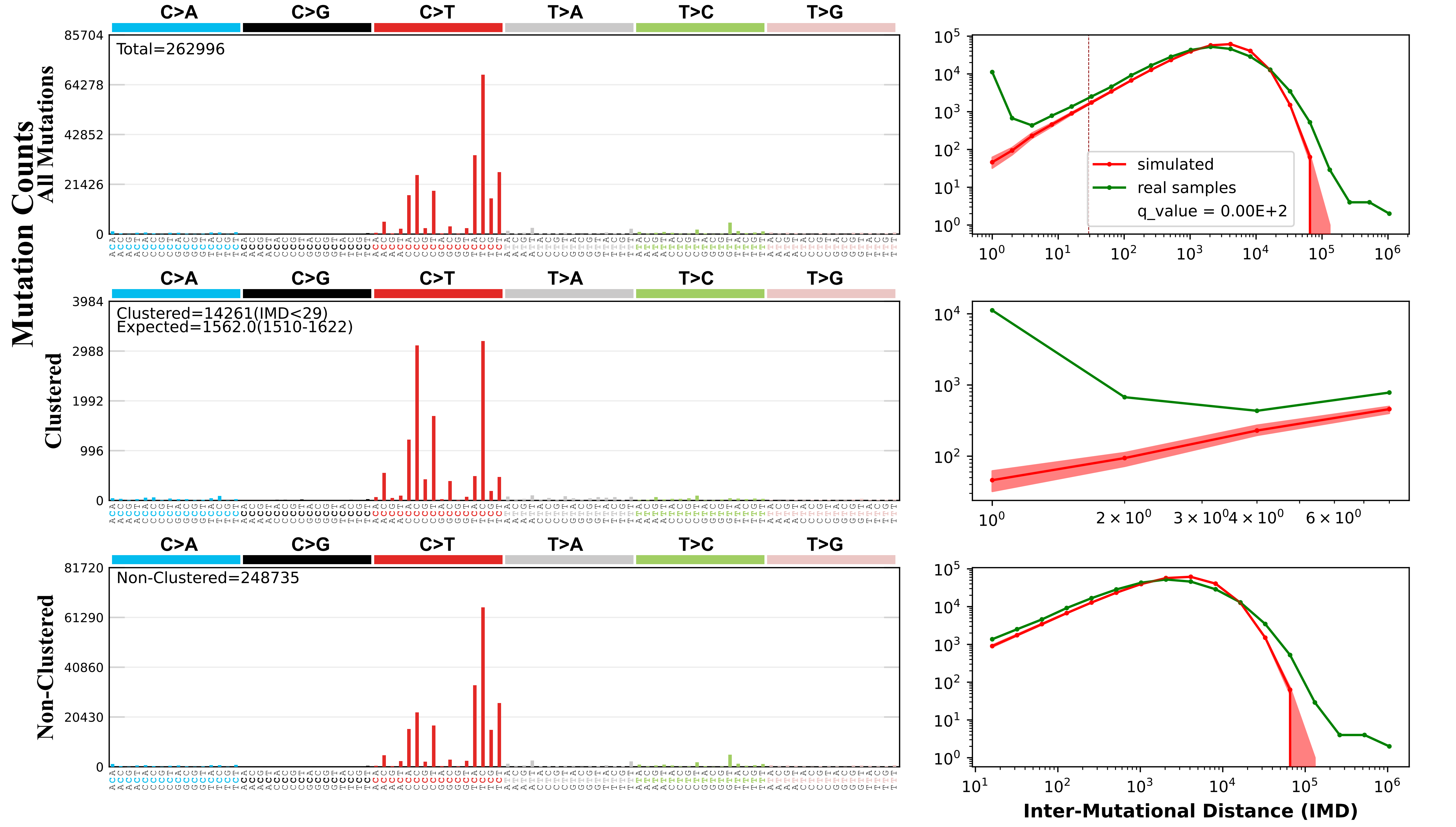

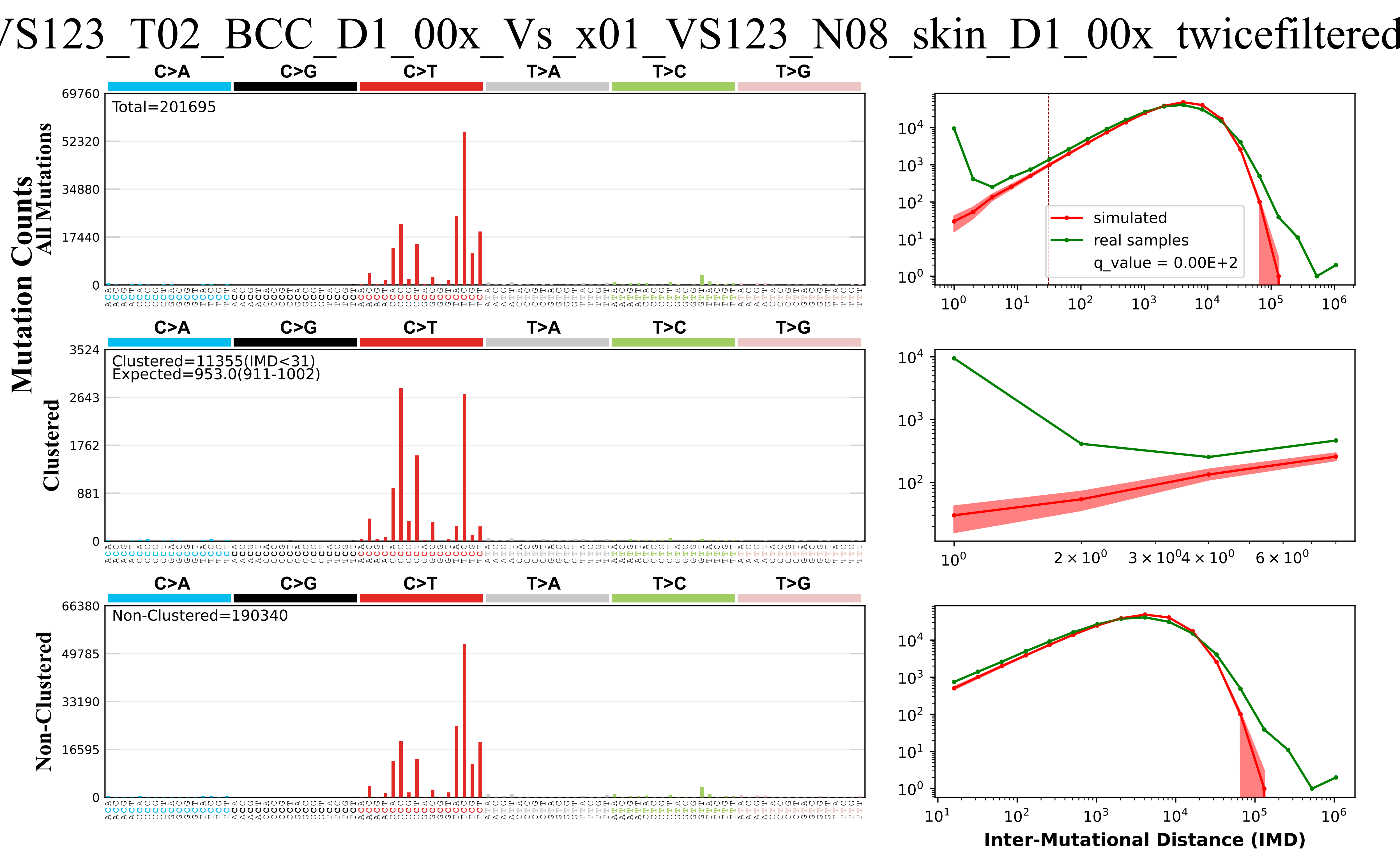
VS123_T02_BCC_D1_00x_Vs_x01_VS123_N08_skin_D1_00x_twicefiltered
Mutation Counts
All Mutations
Total=201695
C>A
C>G
C>T
T>A
T>C
T>G
Clustered
Clustered=11355(IMD<31)
Expected=953.0(911-1002)
Non-Clustered
Non-Clustered=190340
simulated
real samples
q_value = 0.00E+2
Inter-Mutational Distance (IMD)

SRR2194561_Vs_SRR2194570_twicefiltered_TvN

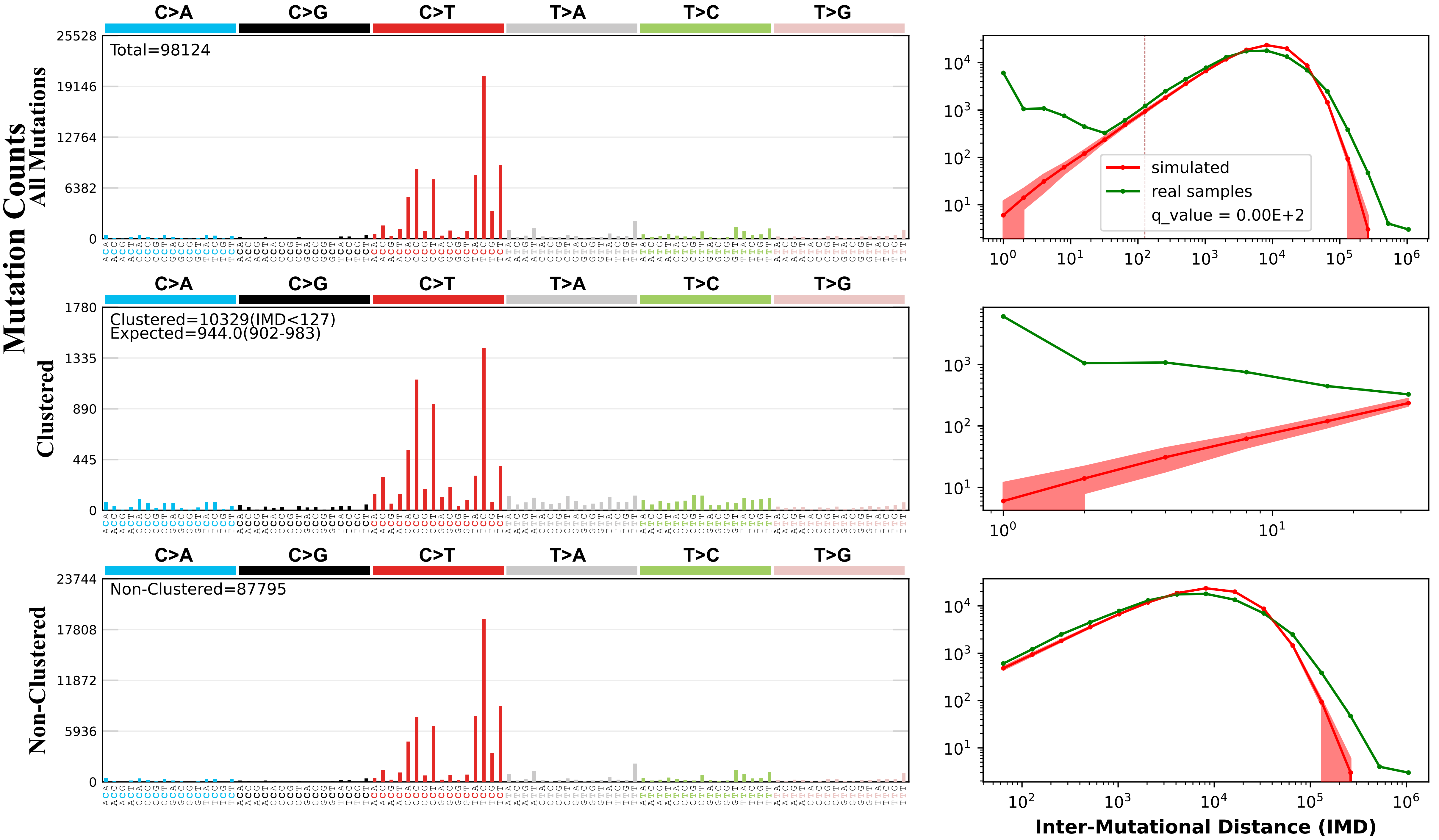

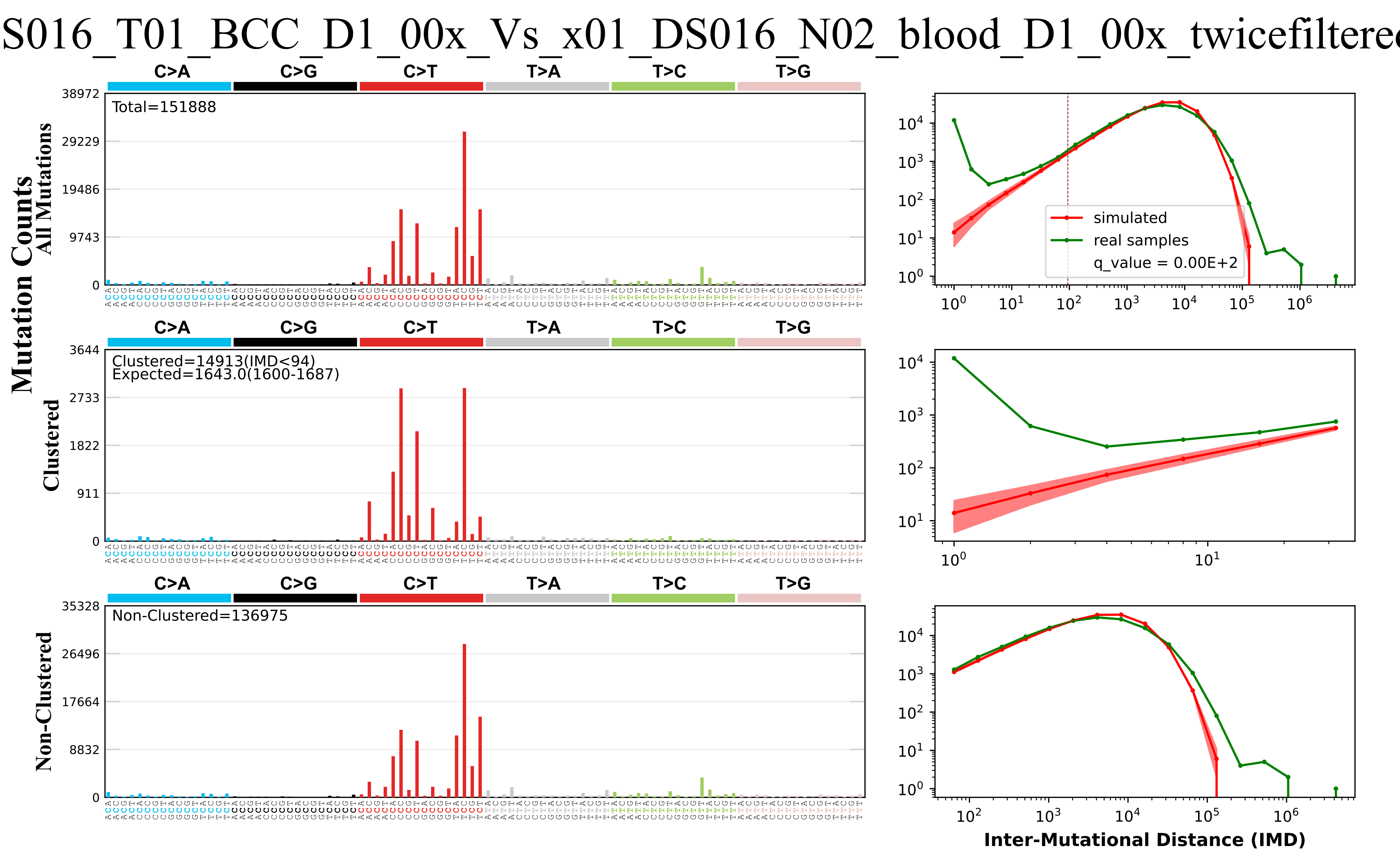
S016_T01_BCC_D1_00x_Vs_x01_DS016_N02_blood_D1_00x_twicefiltere
Mutation Counts
All Mutations
C>A
C>G
C>T
T>A
T>C
T>G
Total=151888
38972
29229
19486
9743
0
simulated
real samples
q_value = 0.00E+2
Clustered
Clustered=14913(IMD<94)
Expected=1643.0(1600-1687)
3644
2733
1822
911
0
Non-Clustered
Non-Clustered=136975
35328
26496
17664
8832
0
Inter-Mutational Distance (IMD)

CPCT02080205T_Vs_CPCT02080205R_twicefiltered_TvN

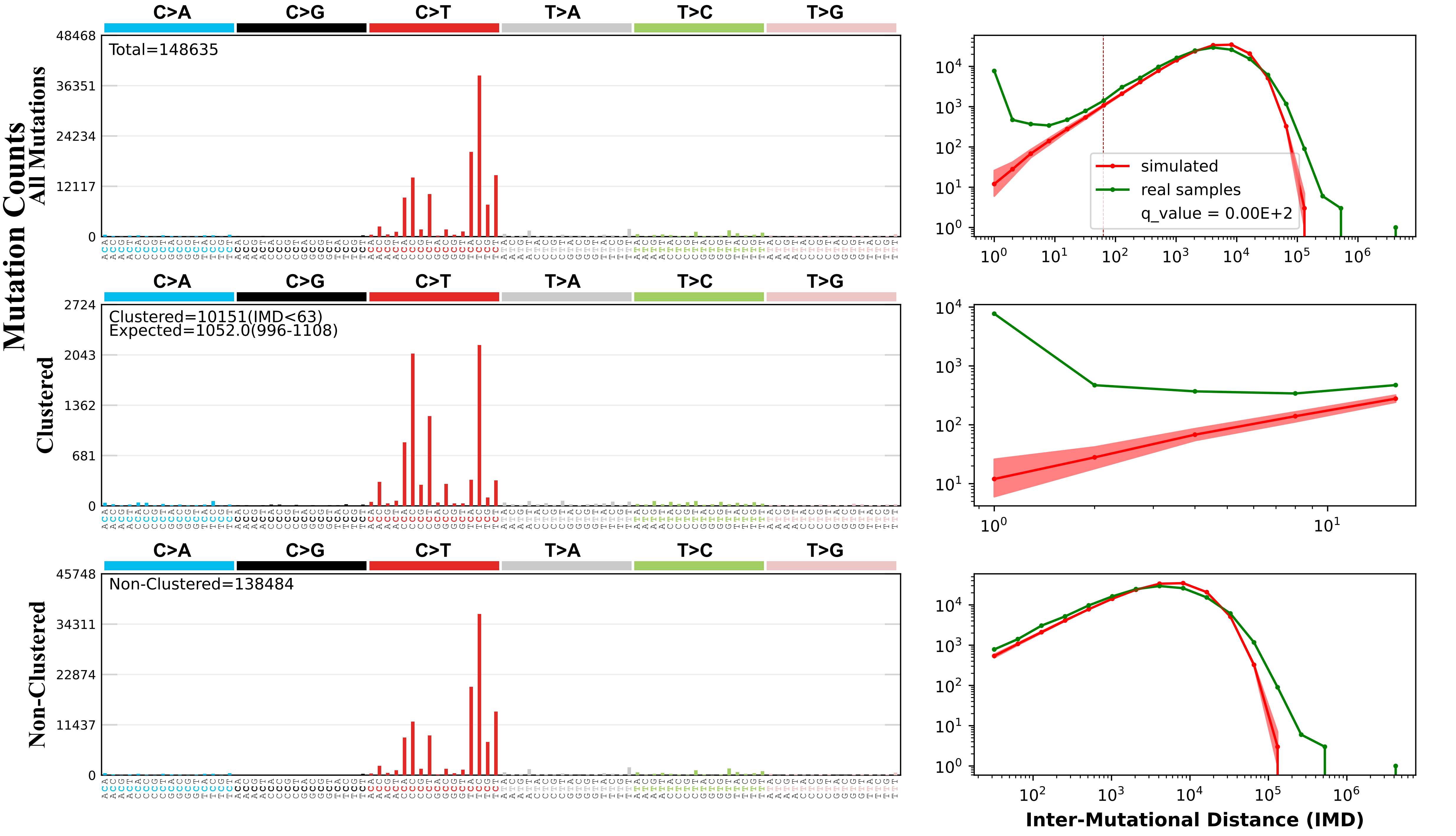

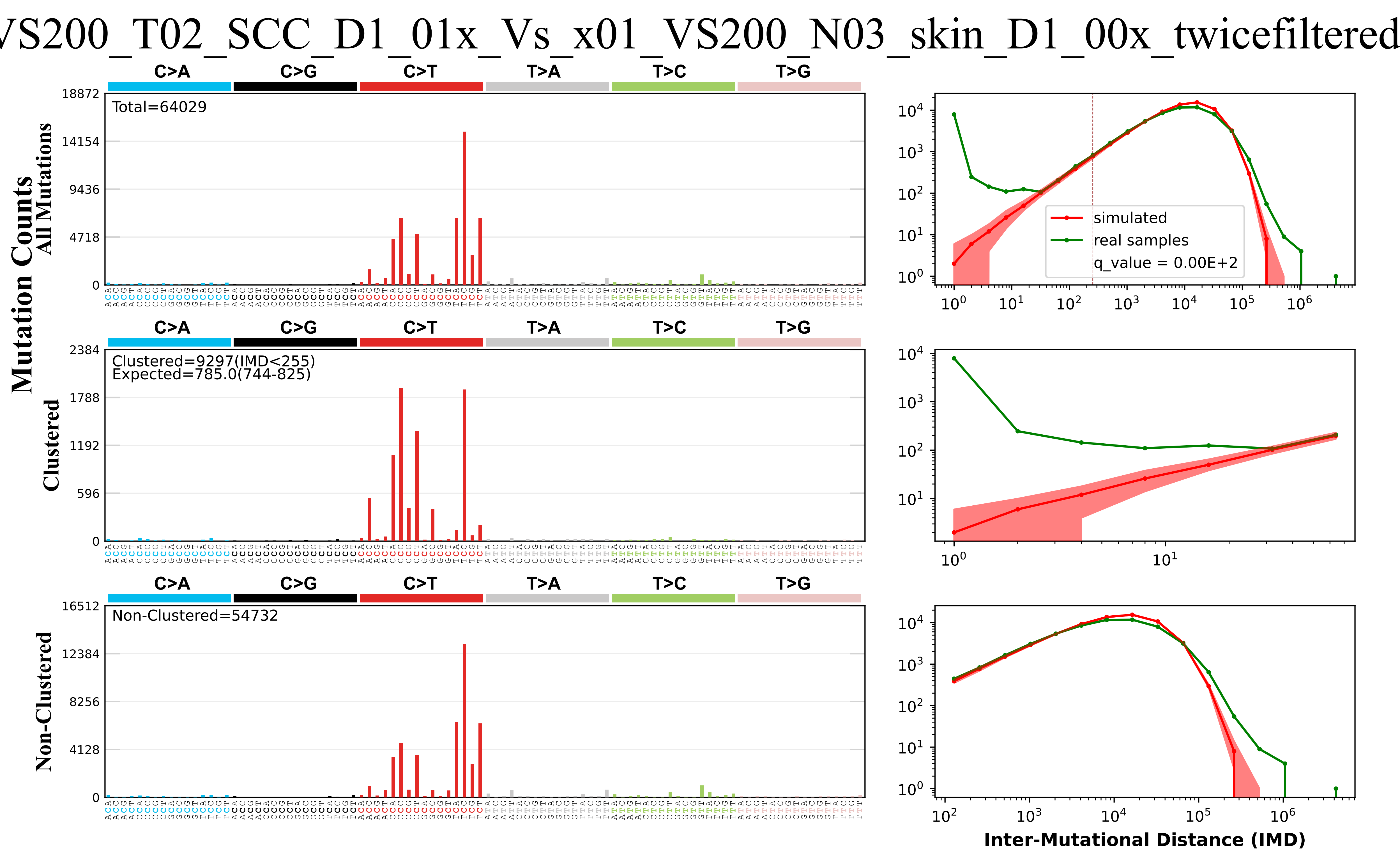

VS200_T02_SCC_D1_01x_Vs_x01_VS200_N03_skin_D1_00x_twicefiltered
Mutation Counts
All Mutations
Clustered
Non-Clustered
C>A
C>G
C>T
T>A
T>C
T>G
Total=64029
Clustered=9297(IMD<255)
Expected=785.0(744-825)
Non-Clustered=54732
simulated
real samples
q_value = 0.00E+2
Inter-Mutational Distance (IMD)

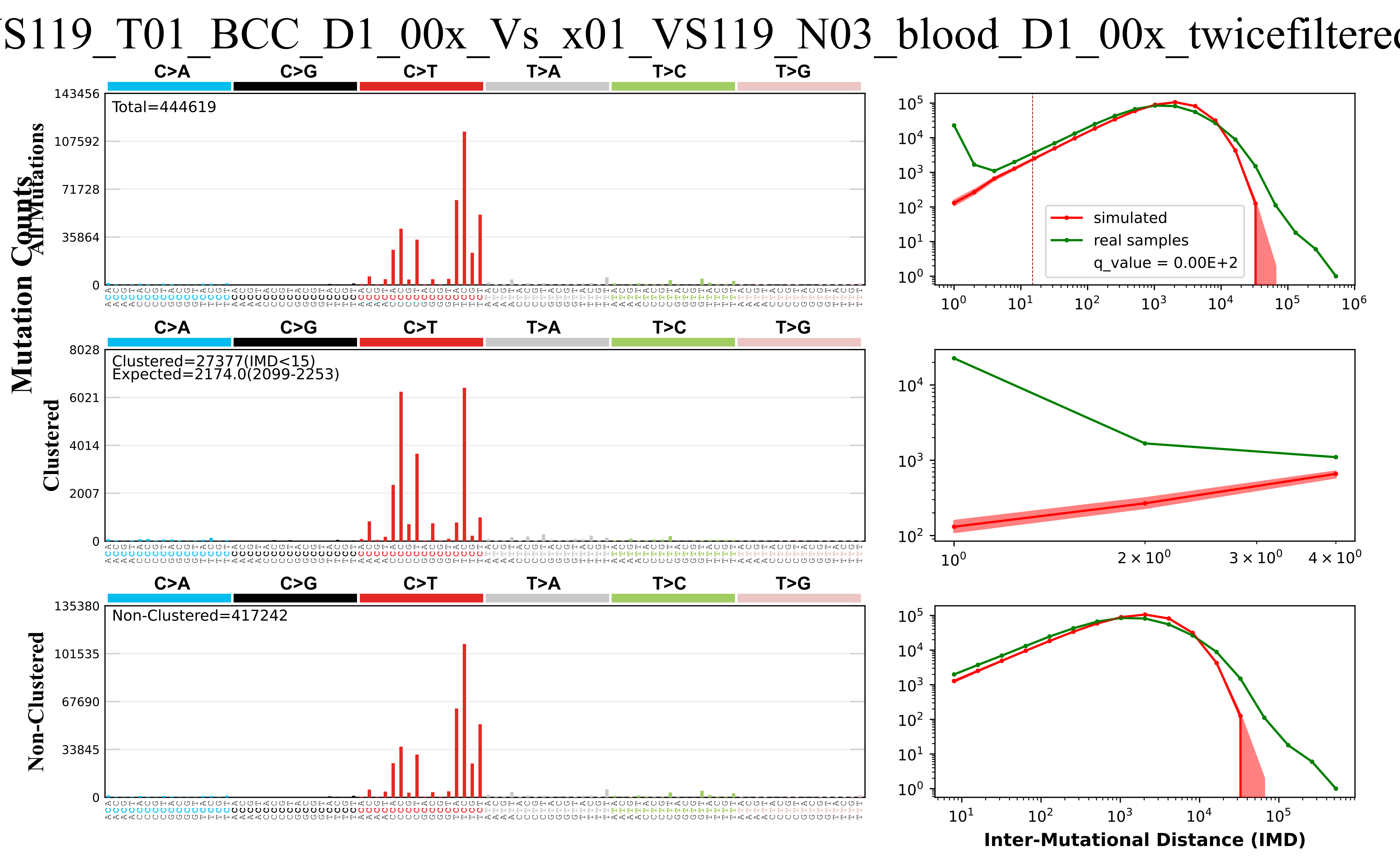

S119_T01_BCC_D1_00x_Vs_x01_VS119_N03_blood_D1_00x_twicefiltere
Mutation Counts
All Mutations
Total=444619
Clustered
Clustered=27377(IMD<15)
Expected=2174.0(2099-2253)
Non-Clustered
Non-Clustered=417242
C>A
C>G
C>T
T>A
T>C
T>G
simulated
real samples
q_value = 0.00E+2
Inter-Mutational Distance (IMD)

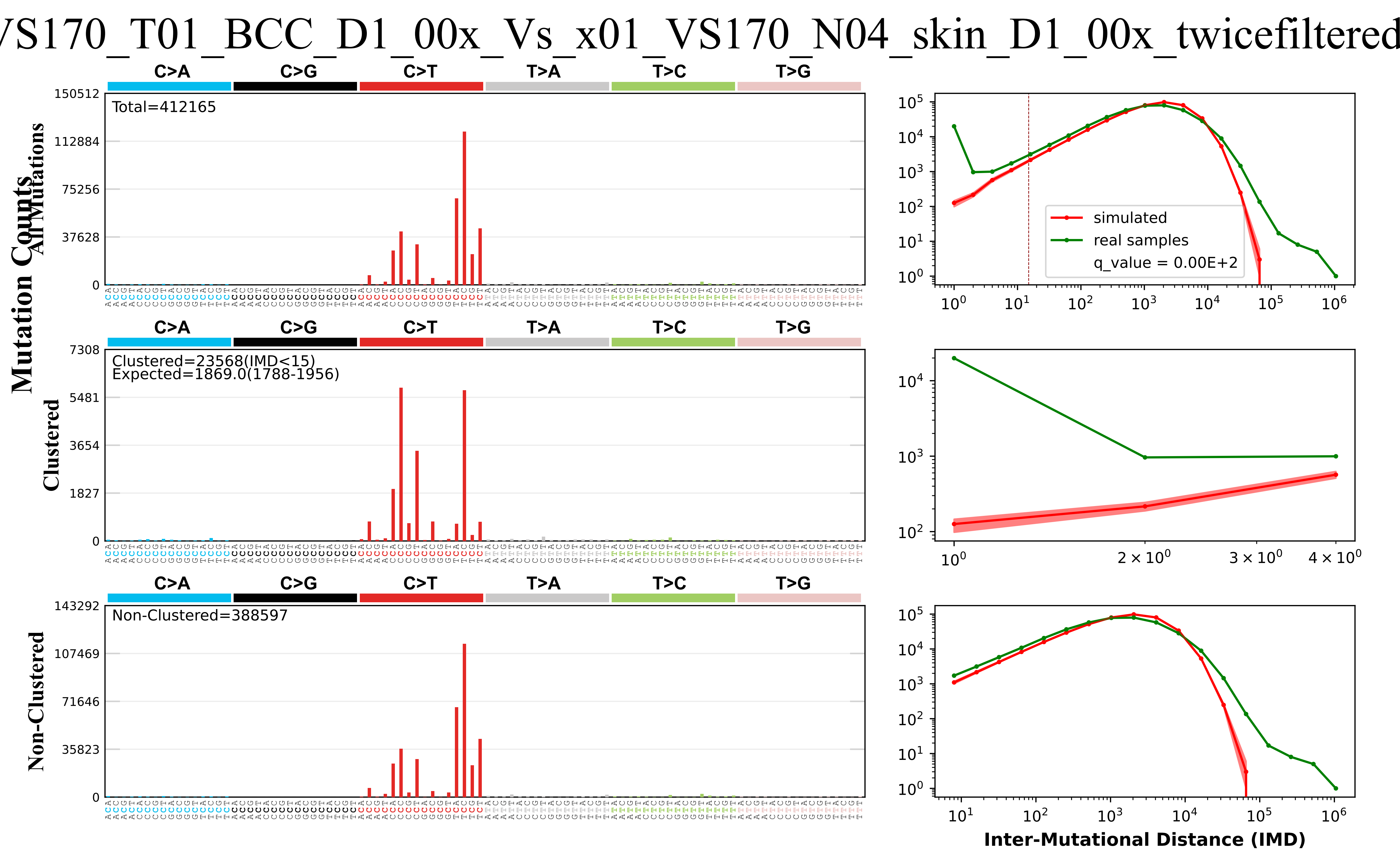
VS170_T01_BCC_D1_00x_Vs_x01_VS170_N04_skin_D1_00x_twicefiltered
Mutation Counts
All Mutations
C>A
C>G
C>T
T>A
T>C
T>G
Total=412165
150512
112884
75256
37628
0
Clustered
Clustered=23568(IMD<15)
Expected=1869.0(1788-1956)
7308
5481
3654
1827
0
Non-Clustered
Non-Clustered=388597
143292
107469
71646
35823
0
simulated
real samples
q_value = 0.00E+2
Inter-Mutational Distance (IMD)

S132_T01_BCC_D1_00x_Vs_x01_VS132_N03_Blood_D1_00x_twicefiltere

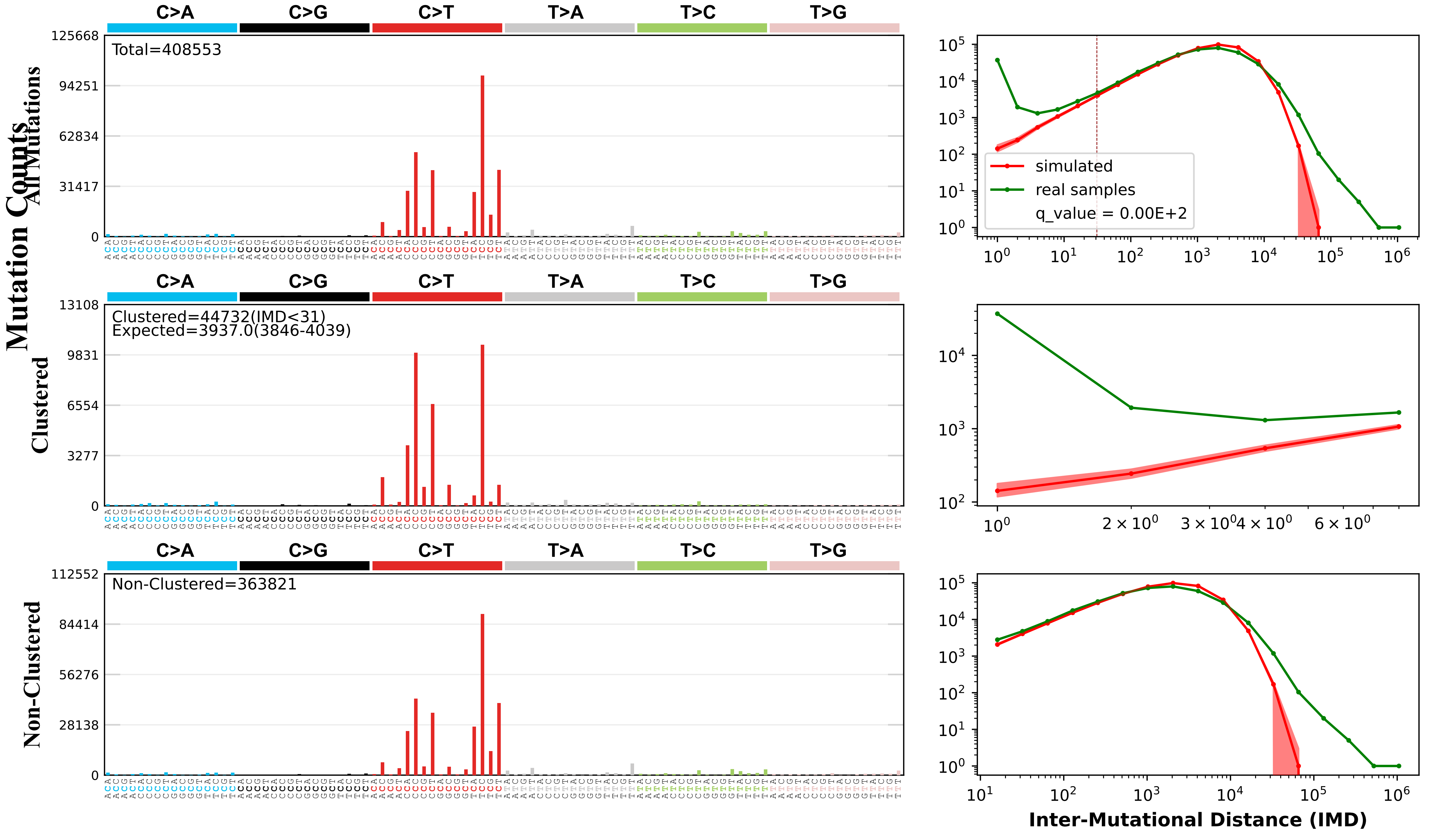

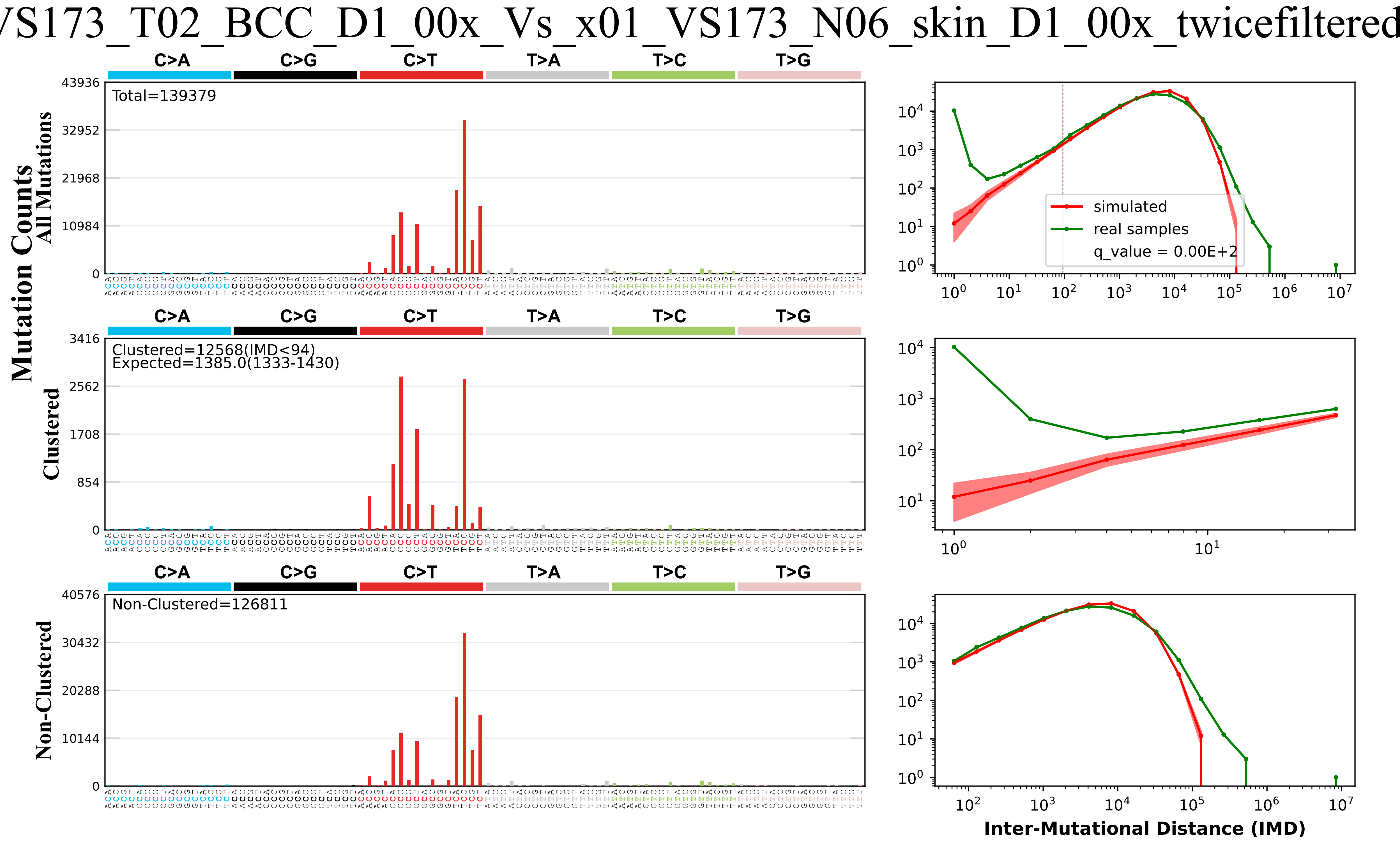
VS173_T02_BCC_D1_00x_Vs_x01_VS173_N06_skin_D1_00x_twicefiltered
Mutation Counts
All Mutations
Clustered
Non-Clustered
C>A
C>G
C>T
T>A
T>C
T>G
Total=139379
Clustered=12568(IMD<94)
Expected=1385.0(1333-1430)
Non-Clustered=126811
simulated
real samples
q_value = 0.00E+2
Inter-Mutational Distance (IMD)

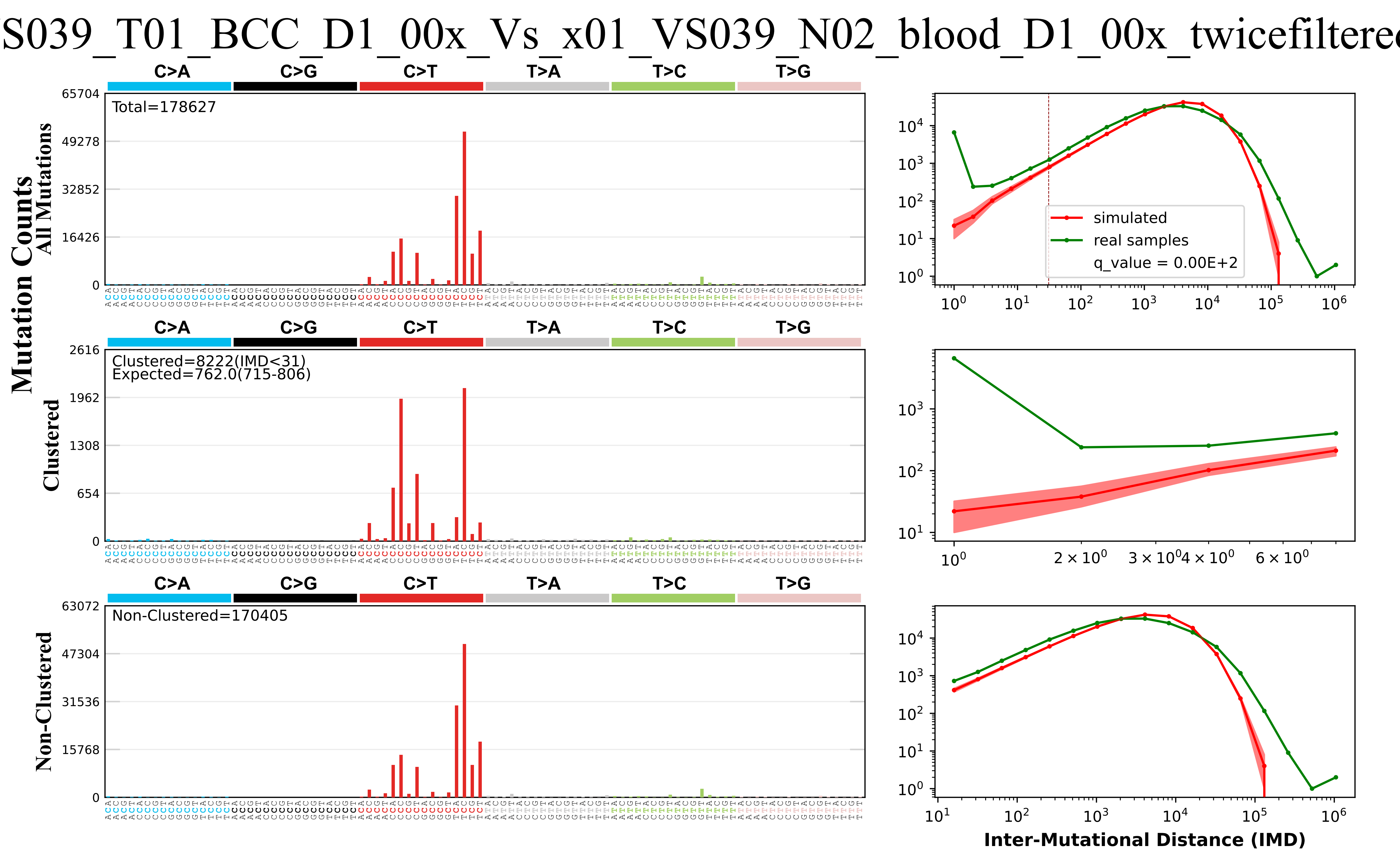
S039_T01_BCC_D1_00x_Vs_x01_VS039_N02_blood_D1_00x_twicefiltere
Mutation Counts
All Mutations
Total=178627
Clustered
Clustered=8222(IMD<31)
Expected=762.0(715-806)
Non-Clustered
Non-Clustered=170405
C>A
C>G
C>T
T>A
T>C
T>G
simulated
real samples
q_value = 0.00E+2
Inter-Mutational Distance (IMD)

S021_T03_BCC_D1_00x_Vs_x01_VS021_N06_blood_D1_00x_twicefiltere

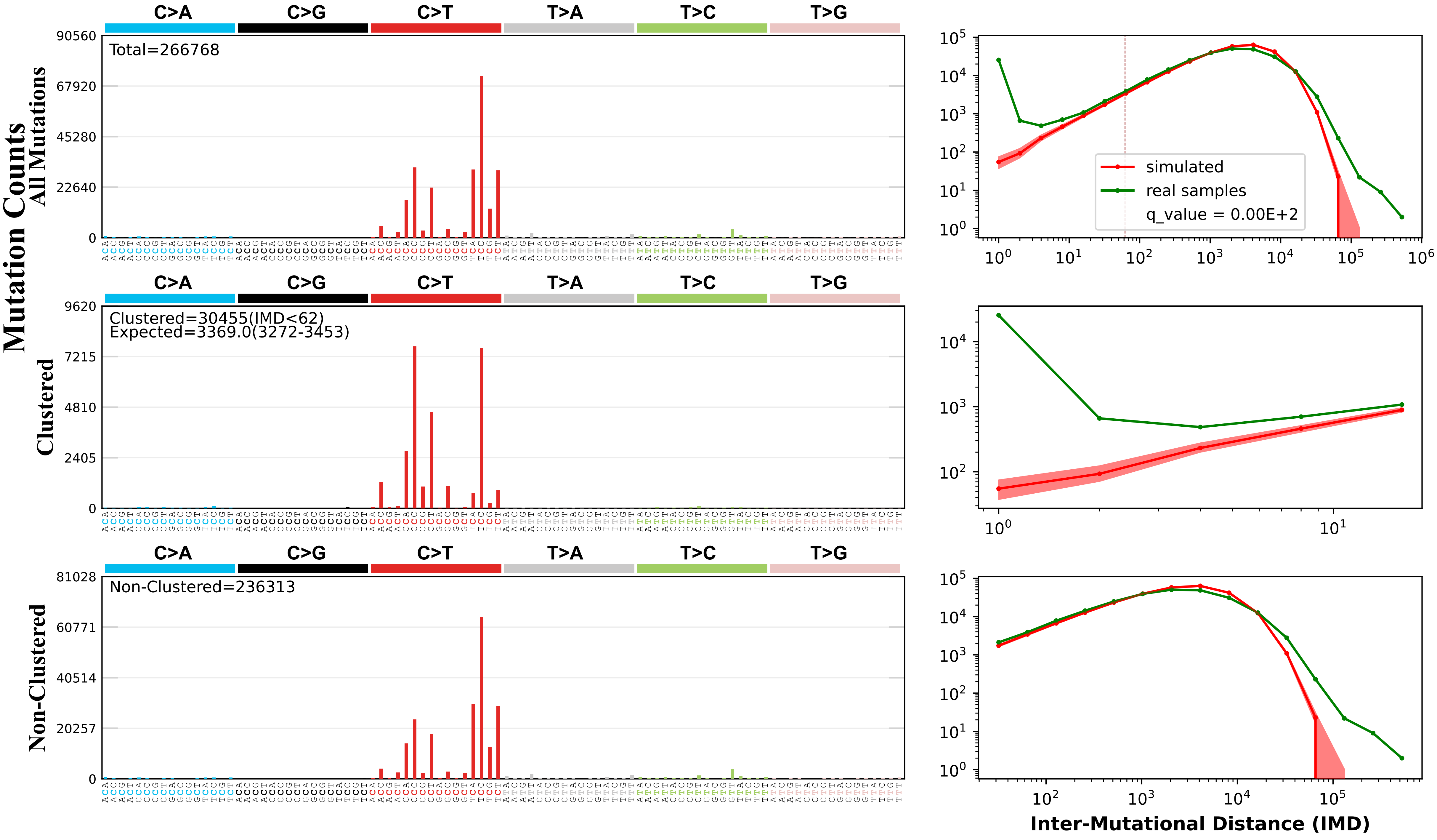

S037_T13_BCC_D1_00x_Vs_x01_VS037_N15_blood_D1_00x_twicefiltere

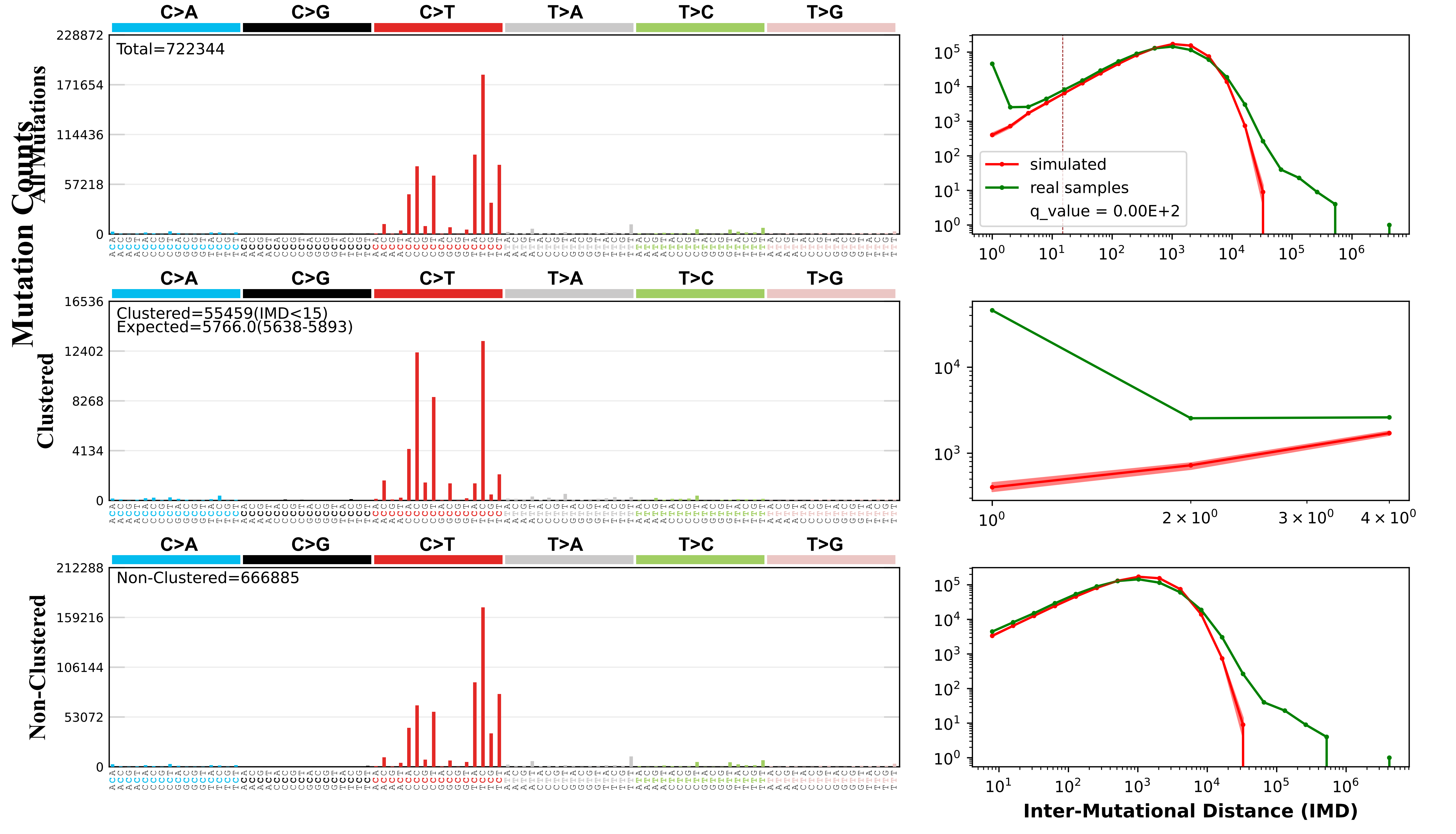

S109_T01_BCC_D1_00x_Vs_x01_VS109_N07_Blood_D1_00x_twicefiltere

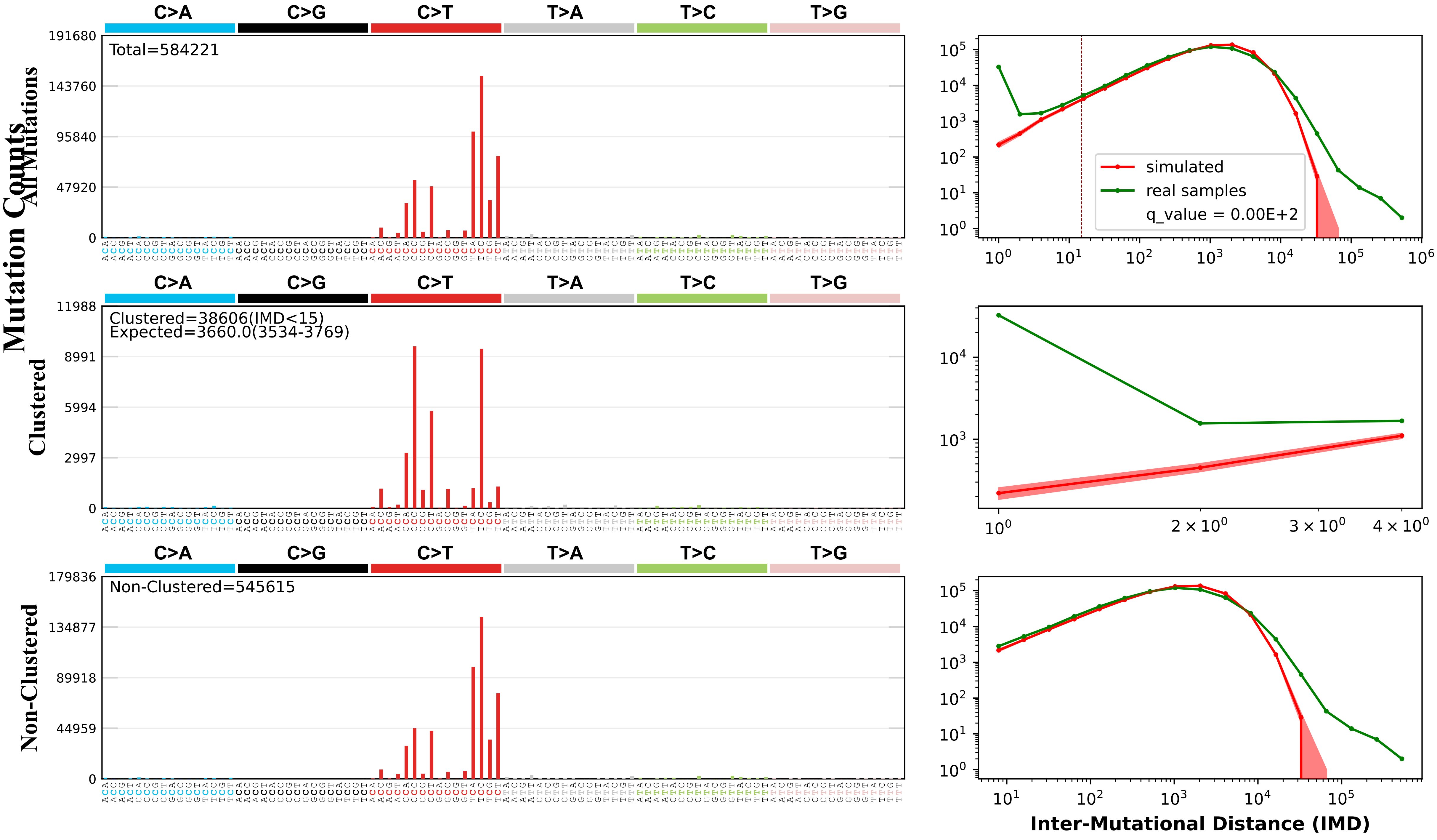

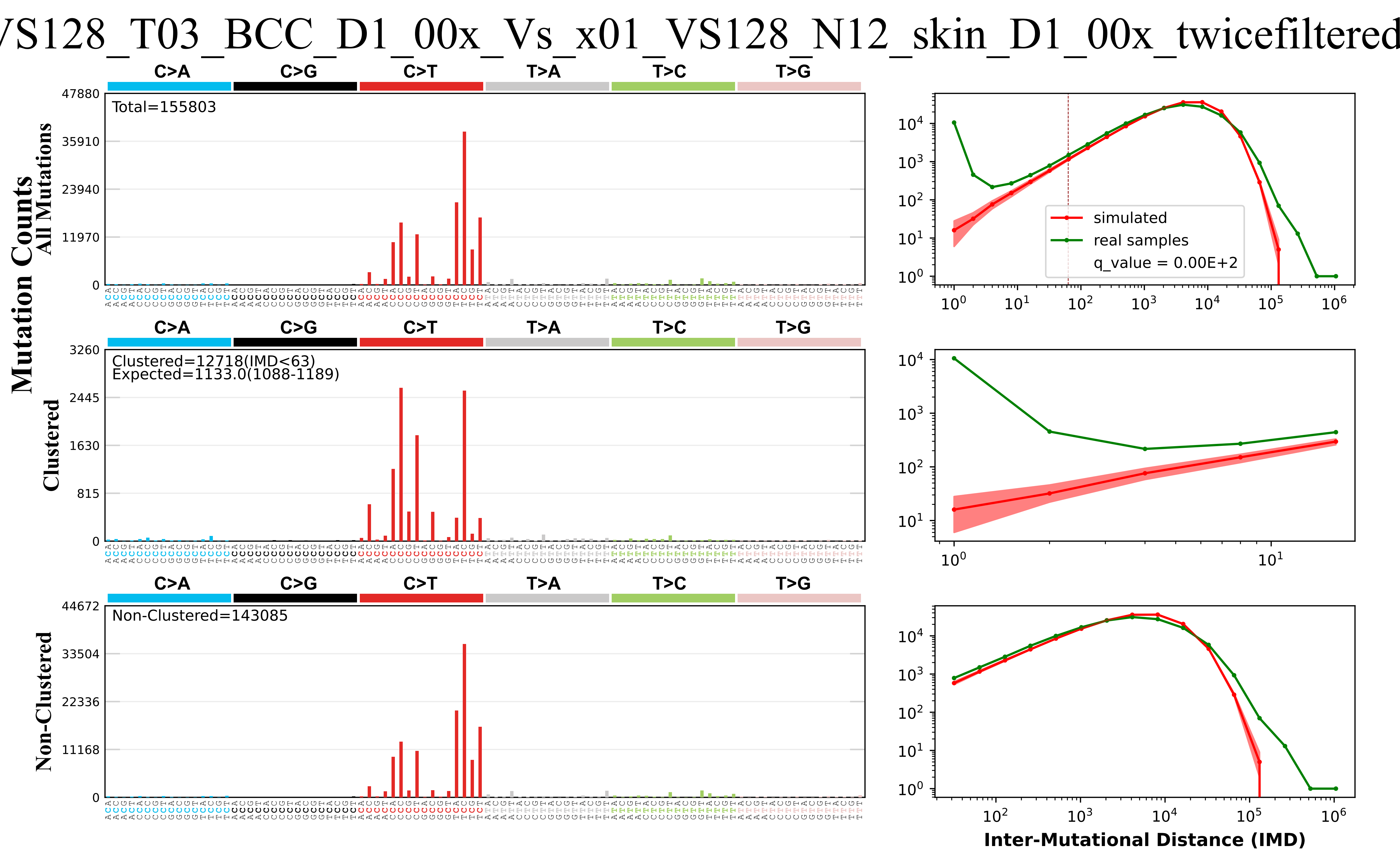

/S128_T03_BCC_D1_00x_Vs_x01_VS128_N12_skin_D1_00x_twicefiltered
Mutation Counts
All Mutations
Clustered
Non-Clustered
C>A
C>G
C>T
T>A
T>C
T>G
Total=155803
Clustered=12718(IMD<63)
Expected=1133.0(1088-1189)
Non-Clustered=143085
simulated
real samples
q_value = 0.00E+2
Inter-Mutational Distance (IMD)

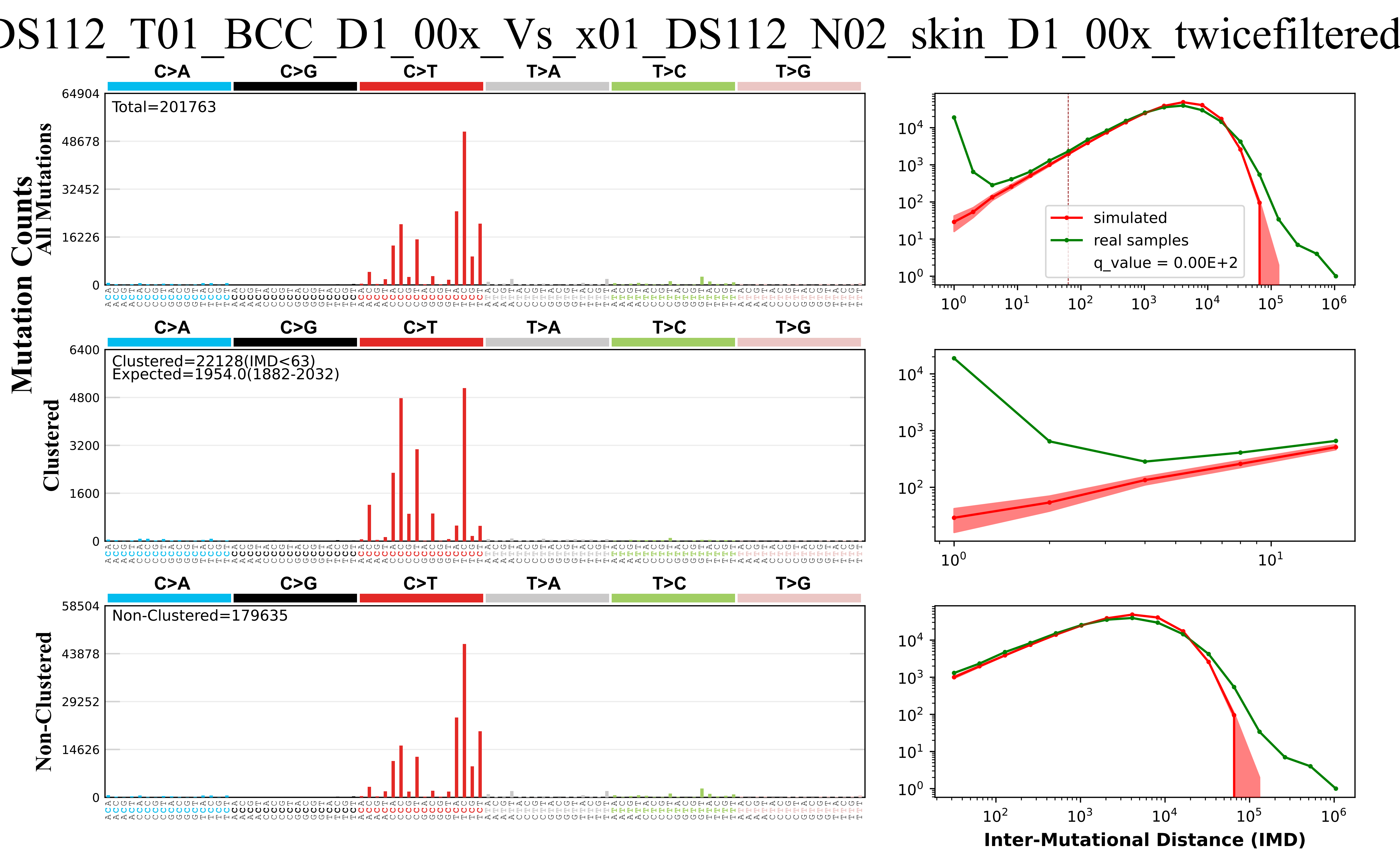
DS112_T01_BCC_D1_00x_Vs_x01_DS112_N02_skin_D1_00x_twicefiltered
Mutation Counts
All Mutations
Total=201763
Clustered
Clustered=22128(IMD<63)
Expected=1954.0(1882-2032)
Non-Clustered
Non-Clustered=179635
C>A
C>G
C>T
T>A
T>C
T>G
simulated
real samples
q_value = 0.00E+2
Inter-Mutational Distance (IMD)

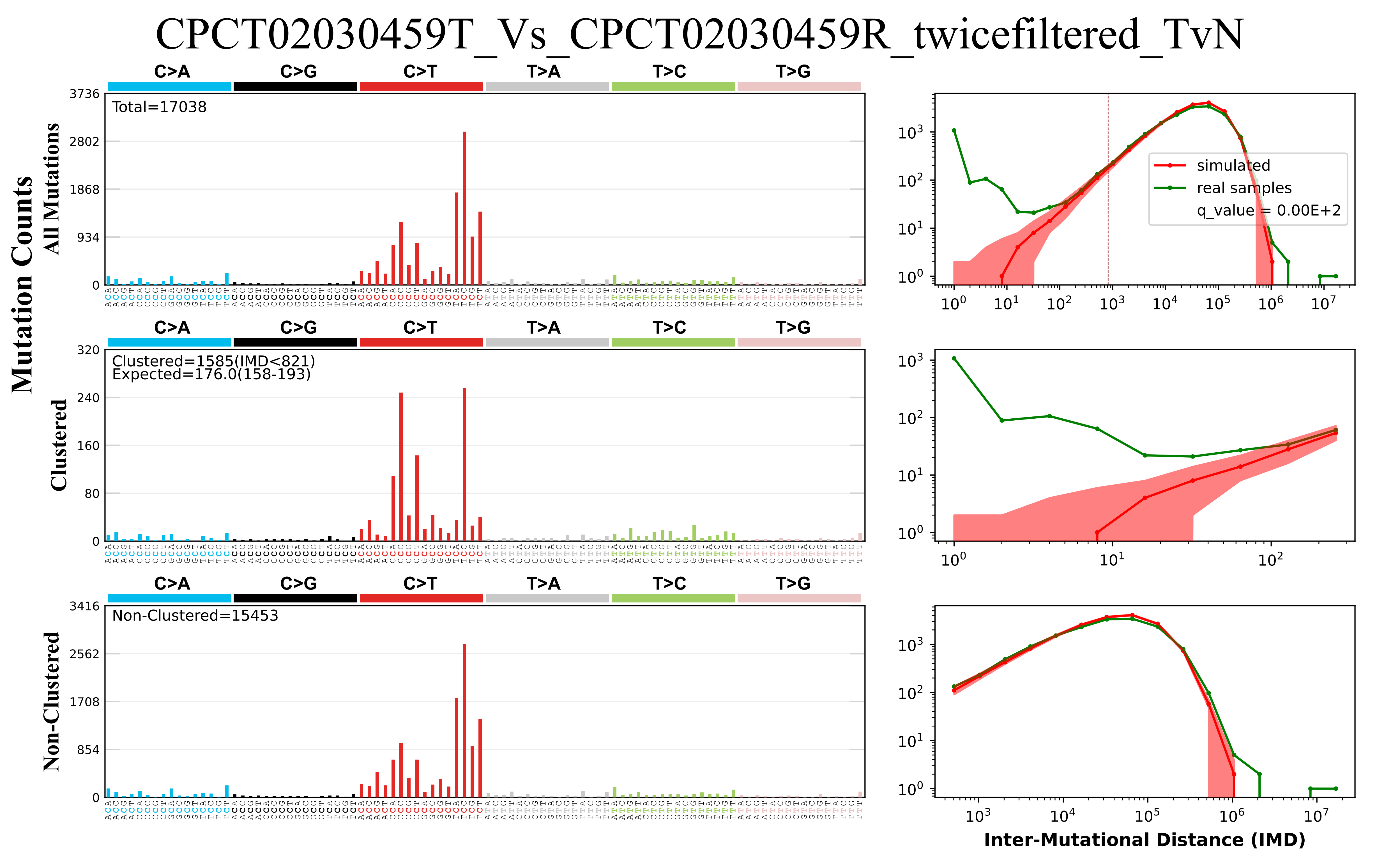

CPCT02030459T_Vs_CPCT02030459R_twicefiltered_TvN
Mutation Counts
All Mutations
Clustered
Non-Clustered
C>A
C>G
C>T
T>A
T>C
T>G
Total=17038
Clustered=1585(IMD<821)
Expected=176.0(158-193)
Non-Clustered=15453
simulated
real samples
q_value = 0.00E+2
Inter-Mutational Distance (IMD)
