# Supplementary material for: Two Faces of UV Mutagenesis: Independent and Collateral Mutagenesis in Skin Cancers
Source: Life (Basel). 2026 Aug 7;16(8):1300. doi: 10.3390/life16081300 (PMC13514280; doi:10.3390/life16081300)
Supplement: Supplementary file 1 [file life-16-01300-s001.zip › Supplementary_file_S1.zip!/MEL_SigProfilerClusters_intradistance_plots_6144_corrected.pdf]

SP129287

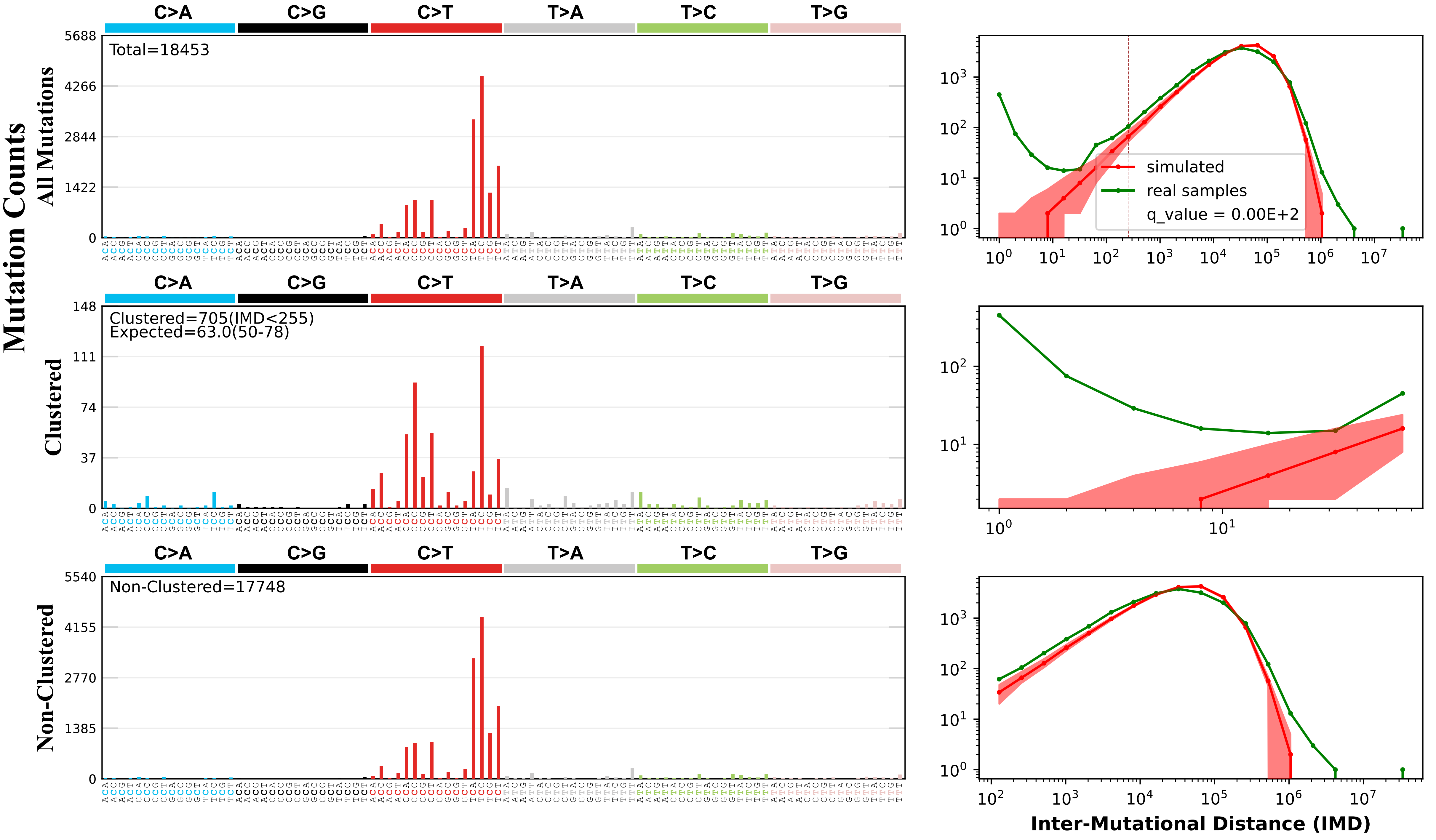

Mutation Counts
All Mutations
Clustered
Non-Clustered
C>A
C>G
C>T
T>A
T>C
T>G
Total=18453
Clustered=705(IMD<255)
Expected=63.0(50-78)
Non-Clustered=17748
simulated
real samples
q_value = 0.00E+2
Inter-Mutational Distance (IMD)

d432e99a-67fb-4609-b90f-99438eee7cae

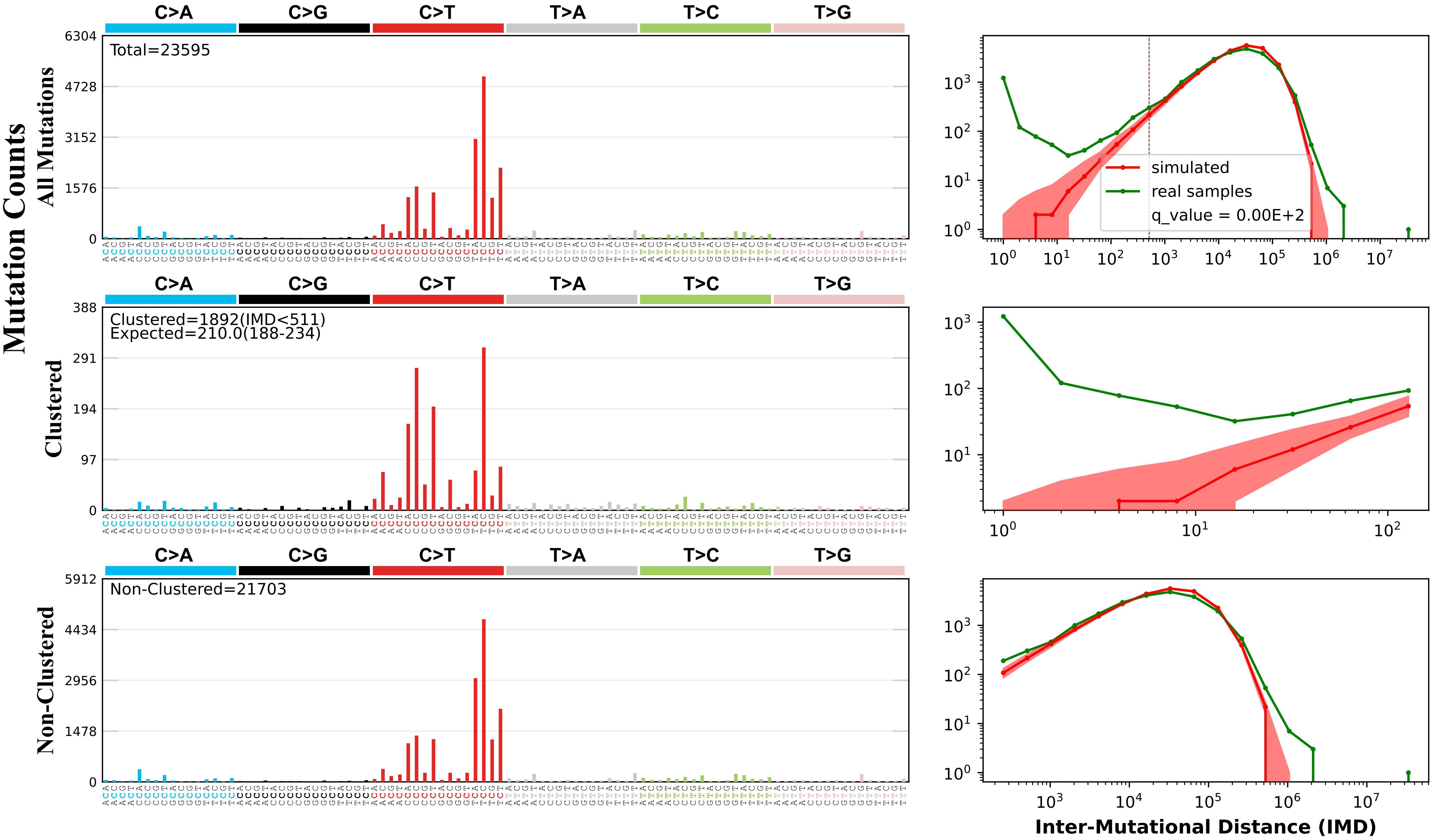

14630c19-9ee0-4ab3-9d8b-8c3309d1e7d0

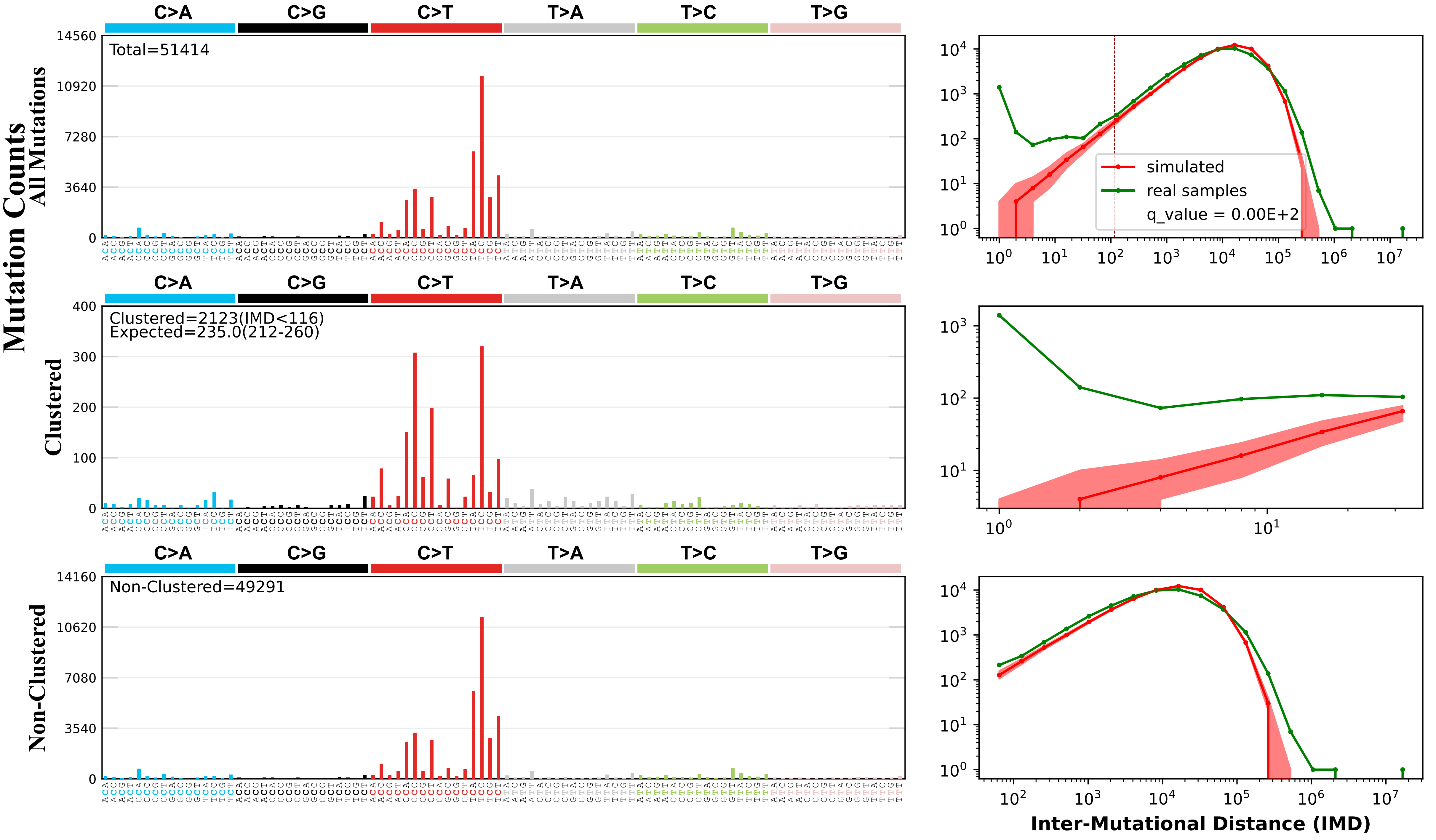

SP128789

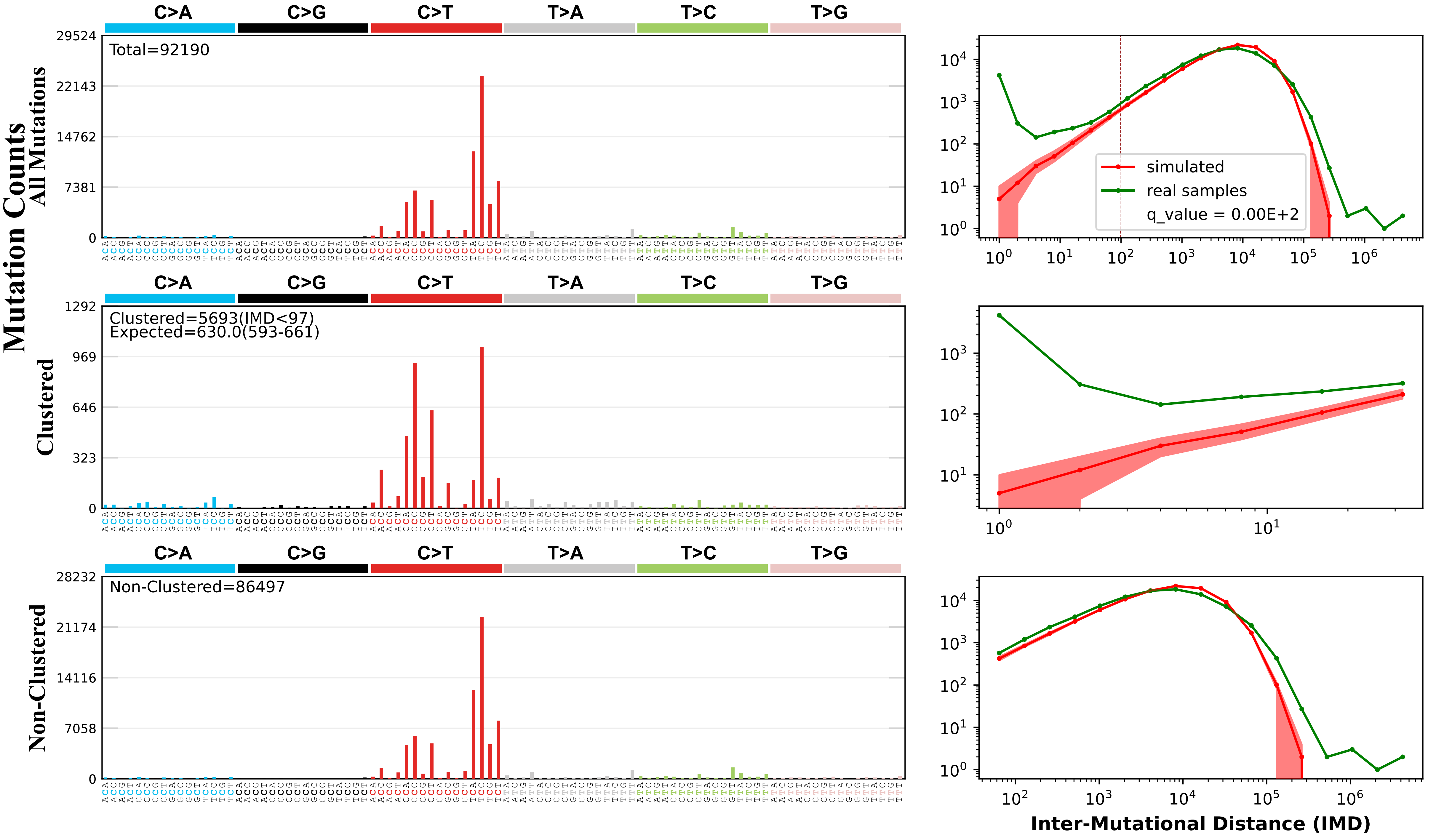

C>A
C>G
C>T
T>A
T>C
T>G
Mutation Counts
All Mutations
Total=92190
29524
22143
14762
7381
0
Clustered
Clustered=5693(IMD<97)
Expected=630.0(593-661)
1292
969
646
323
0
Non-Clustered
Non-Clustered=86497
28232
21174
14116
7058
0
simulated
real samples
q_value = 0.00E+2
Inter-Mutational Distance (IMD)

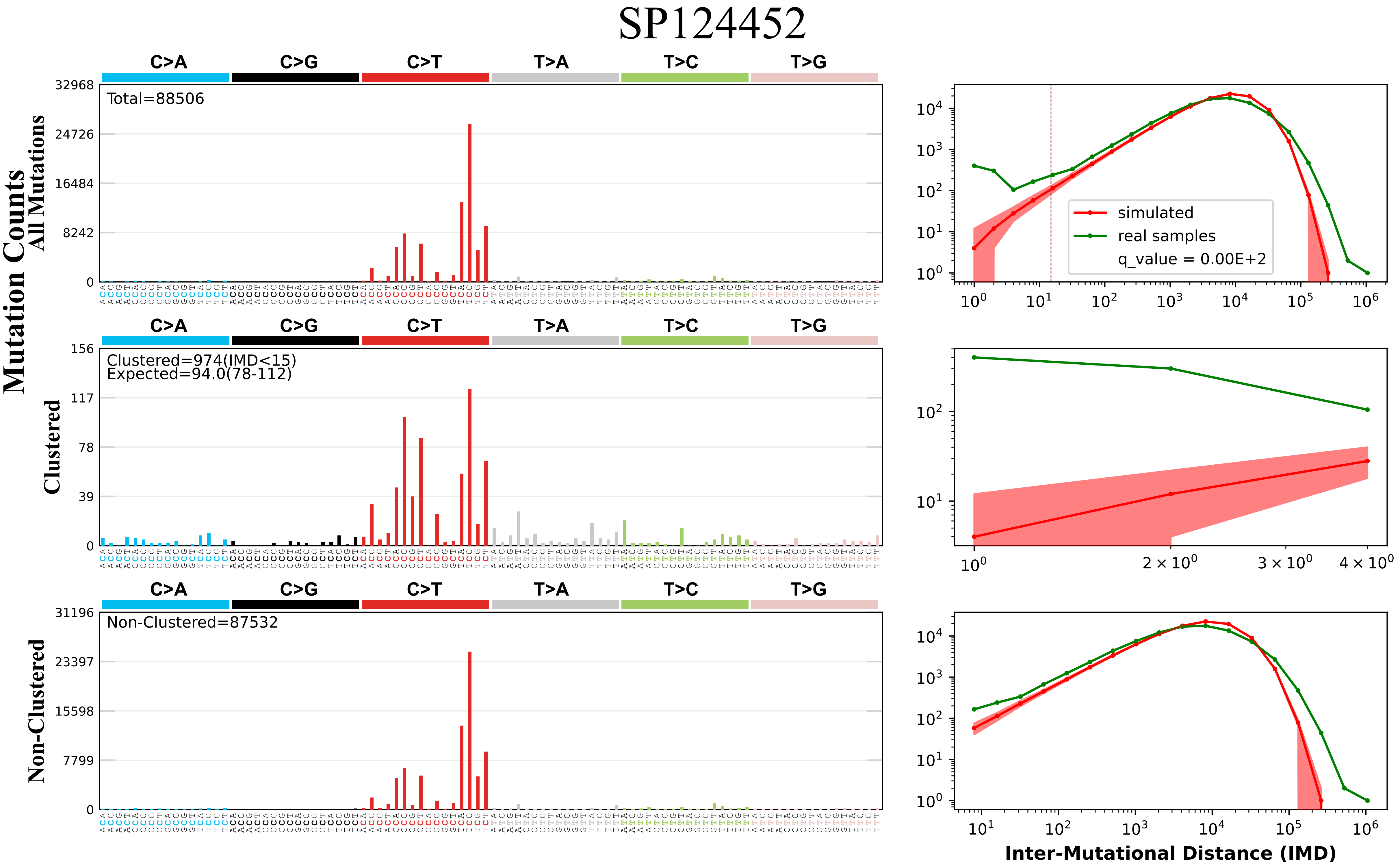
SP124452
C>A
C>G
C>T
T>A
T>C
T>G
Mutation Counts
All Mutations
Total=88506
32968
24726
16484
8242
0
simulated
real samples
q_value = 0.00E+2
Clustered
Clustered=974(IMD<15)
Expected=94.0(78-112)
156
117
78
39
Non-Clustered
Non-Clustered=87532
31196
23397
15598
7799
Inter-Mutational Distance (IMD)

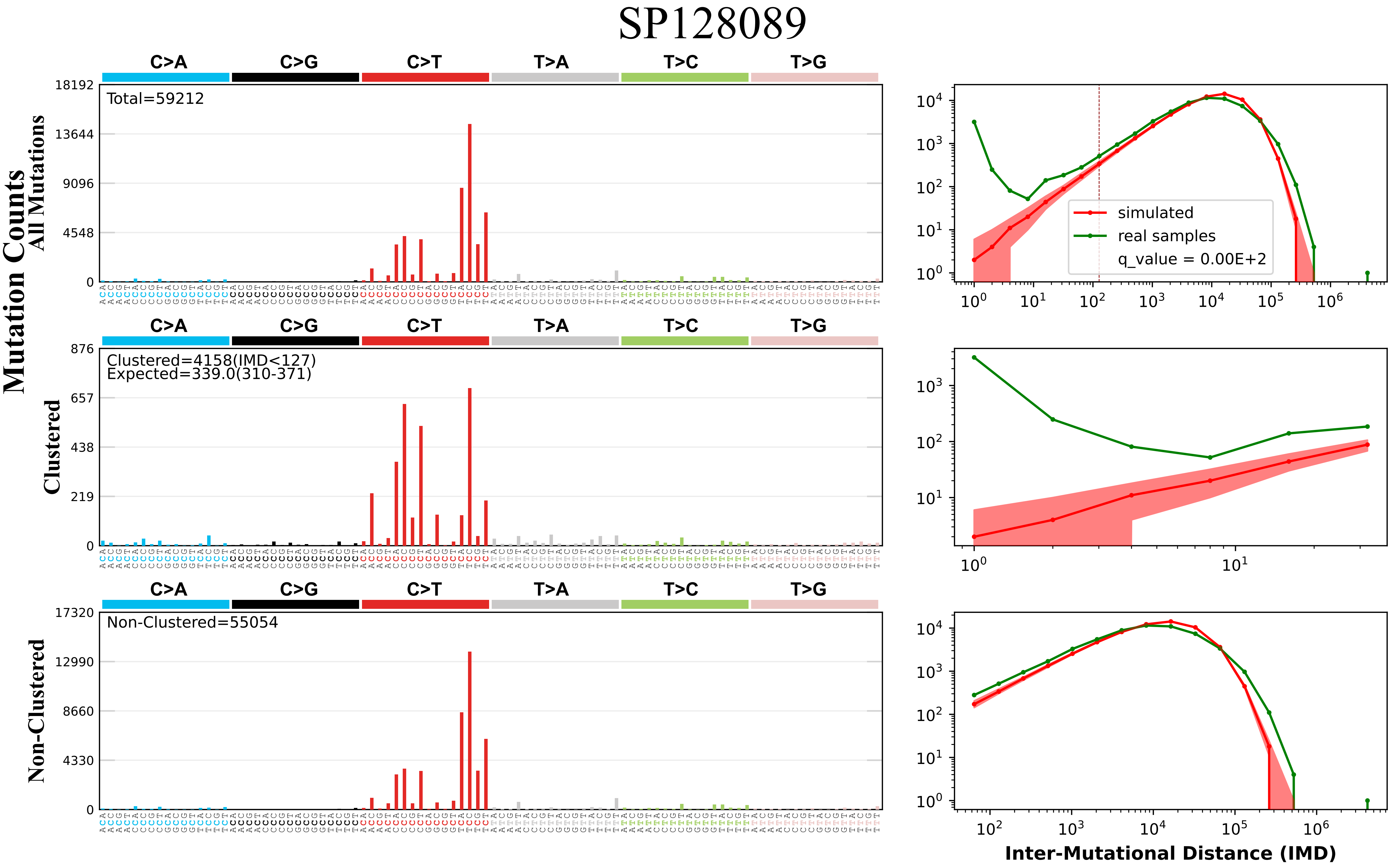
SP128089
C>A
C>G
C>T
T>A
T>C
T>G
Mutation Counts
All Mutations
Total=59212
Clustered
Clustered=4158(IMD<127)
Expected=339.0(310-371)
Non-Clustered
Non-Clustered=55054
simulated
real samples
q_value = 0.00E+2
Inter-Mutational Distance (IMD)

fc249113-83d4-4abe-8c80-a4f7305dcd91

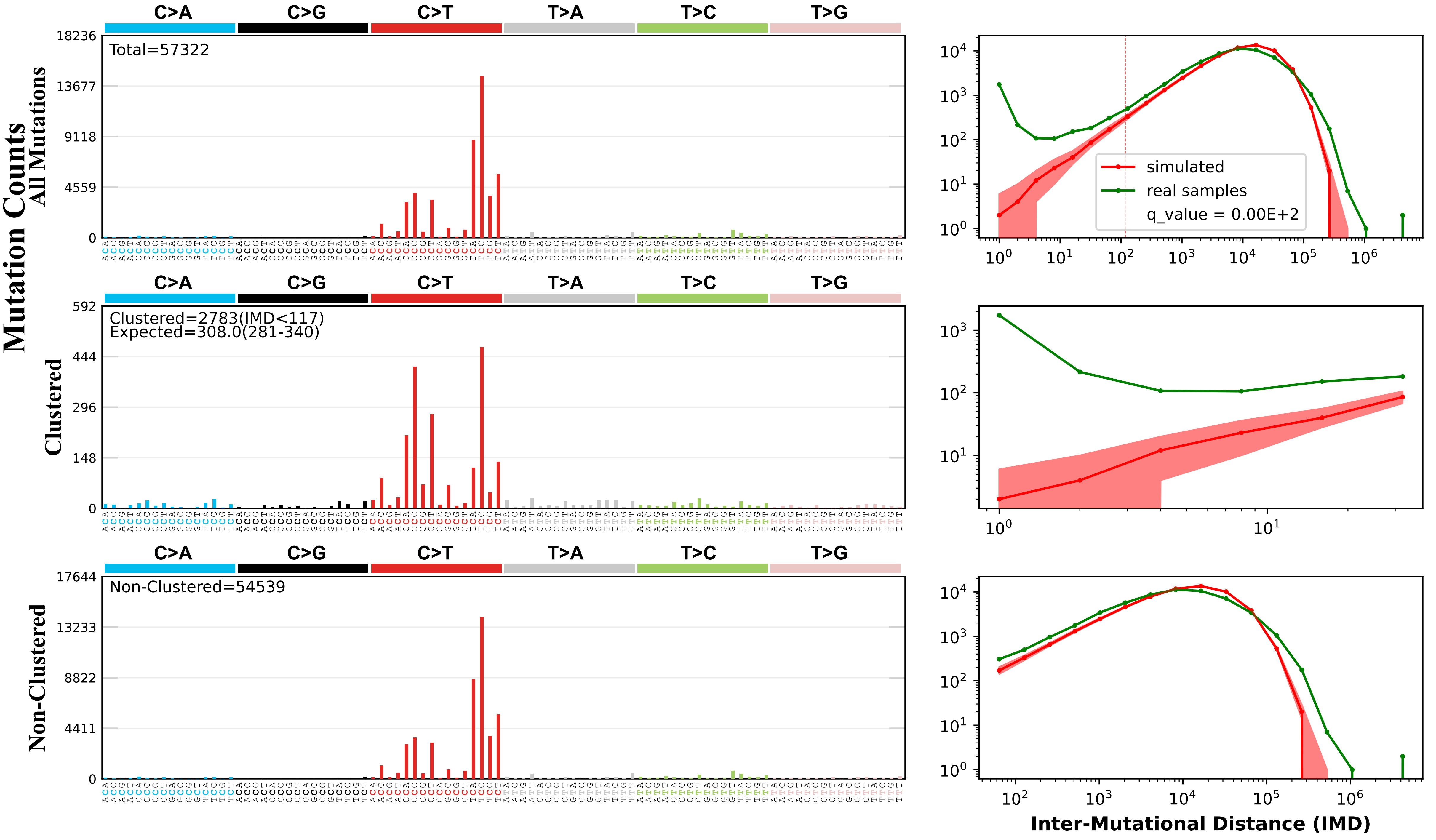

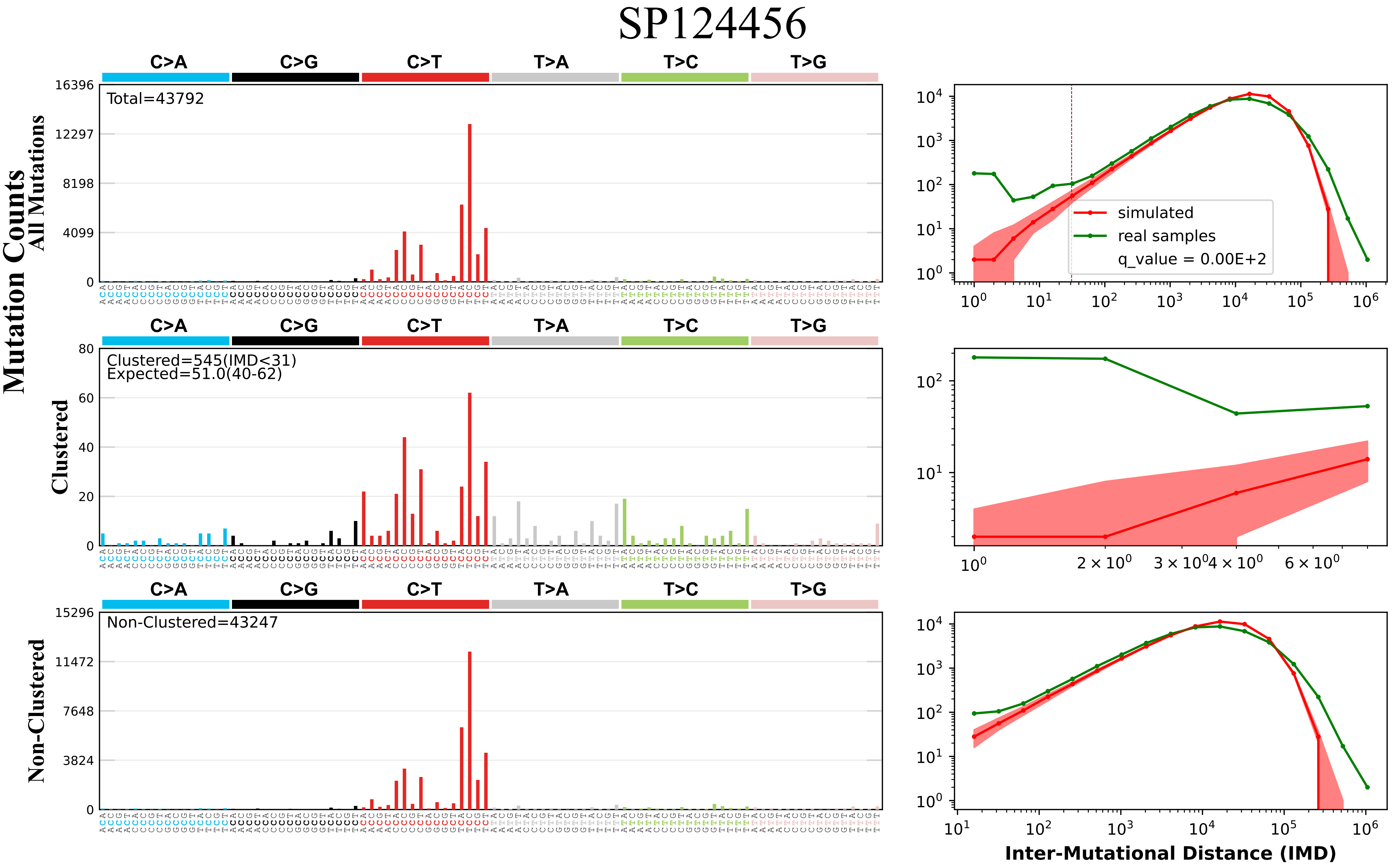

SP124456
C>A
C>G
C>T
T>A
T>C
T>G
Mutation Counts
All Mutations
Total=43792
16396
12297
8198
4099
0
Clustered
Clustered=545(IMD<31)
Expected=51.0(40-62)
80
60
40
20
0
Non-Clustered
Non-Clustered=43247
15296
11472
7648
3824
0
simulated
real samples
q_value = 0.00E+2
Inter-Mutational Distance (IMD)

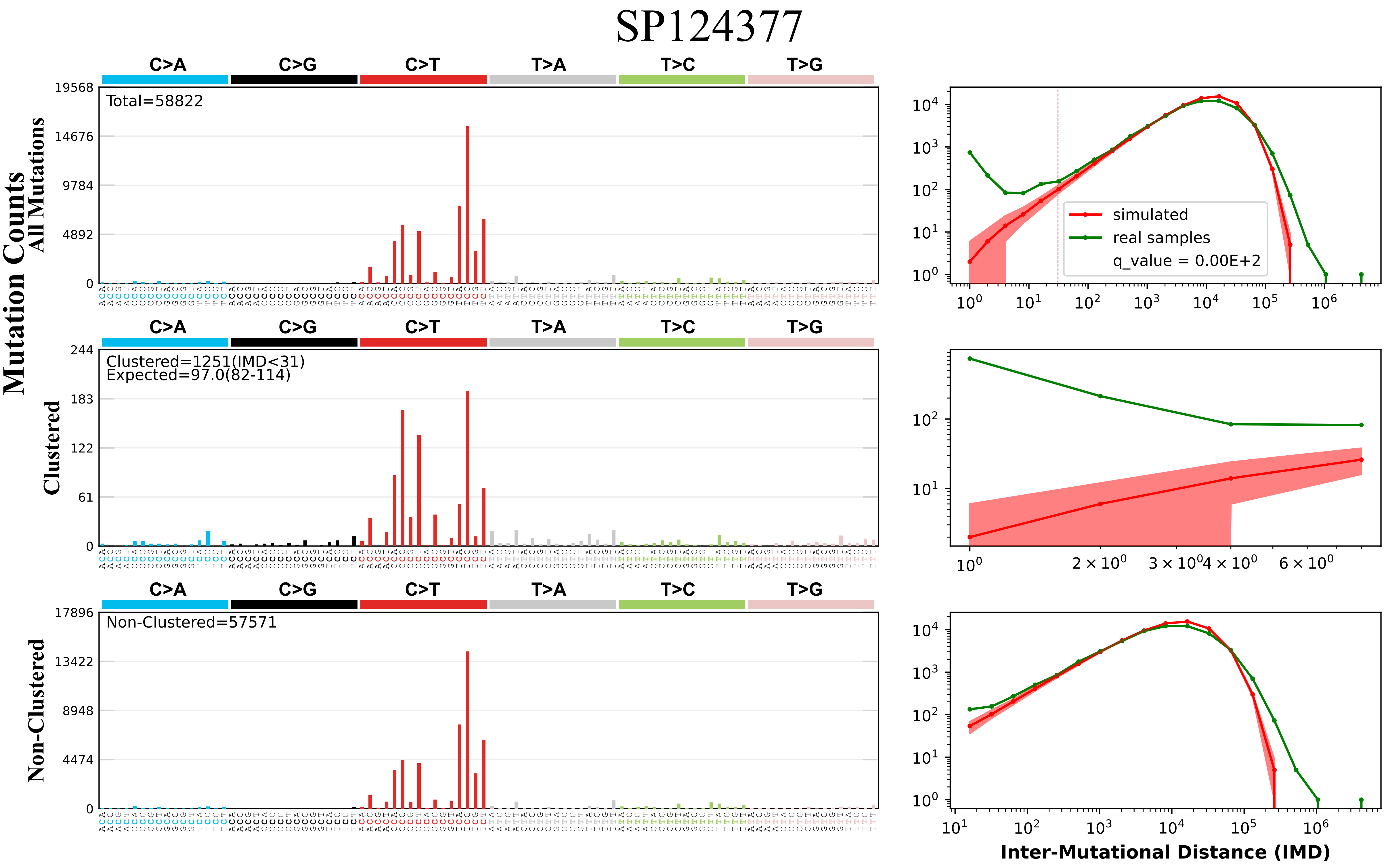
SP124377
C>A
C>G
C>T
T>A
T>C
T>G
Mutation Counts
All Mutations
Total=58822
Clustered
Clustered=1251(IMD<31)
Expected=97.0(82-114)
Non-Clustered
Non-Clustered=57571
simulated
real samples
q_value = 0.00E+2
Inter-Mutational Distance (IMD)

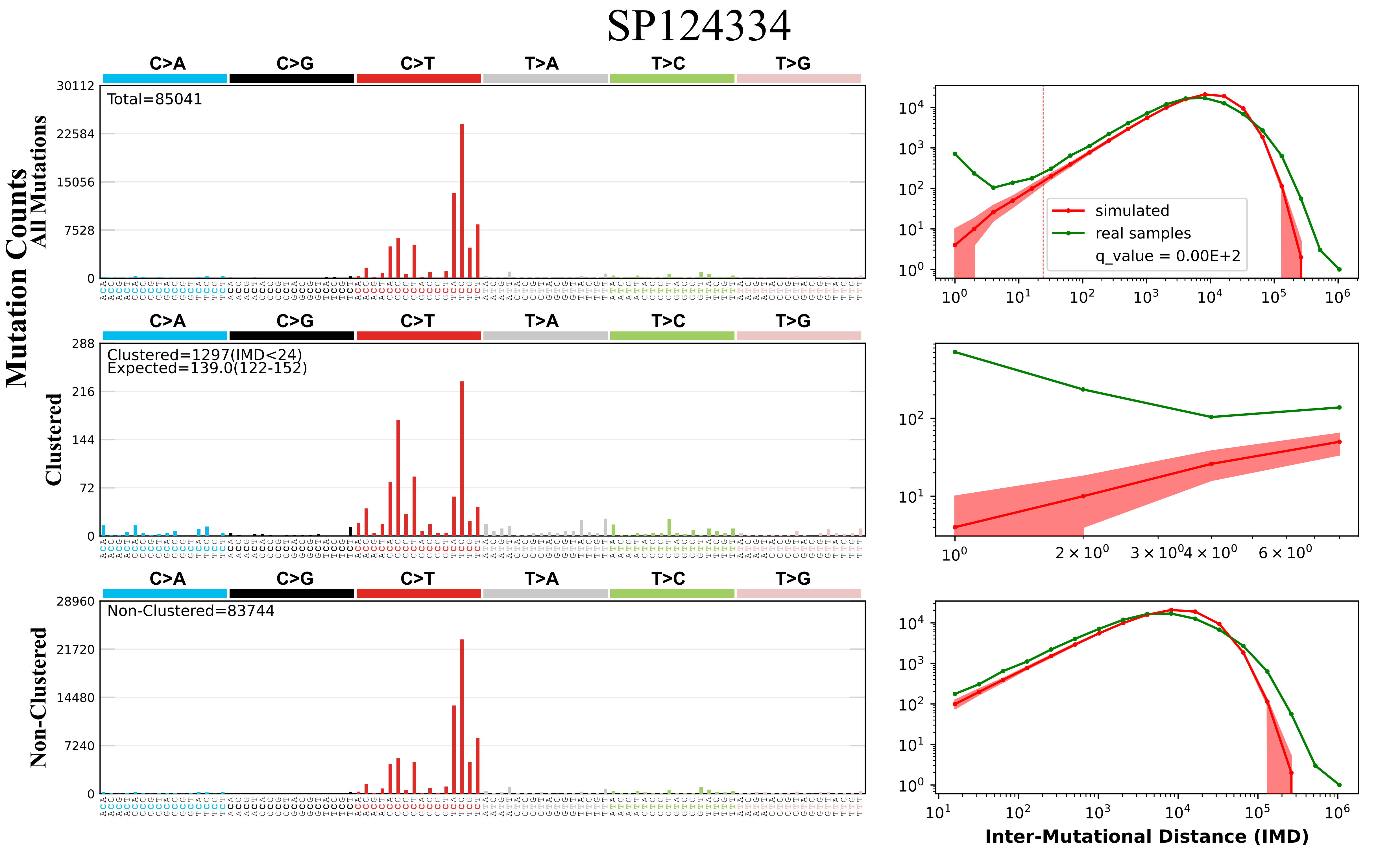

SP124334
Mutation Counts
All Mutations
Clustered
Non-Clustered
C>A
C>G
C>T
T>A
T>C
T>G
Total=85041
Clustered=1297(IMD<24)
Expected=139.0(122-152)
Non-Clustered=83744
simulated
real samples
q_value = 0.00E+2
Inter-Mutational Distance (IMD)

22edd867-dcd6-42b5-92bb-025ceb84b106

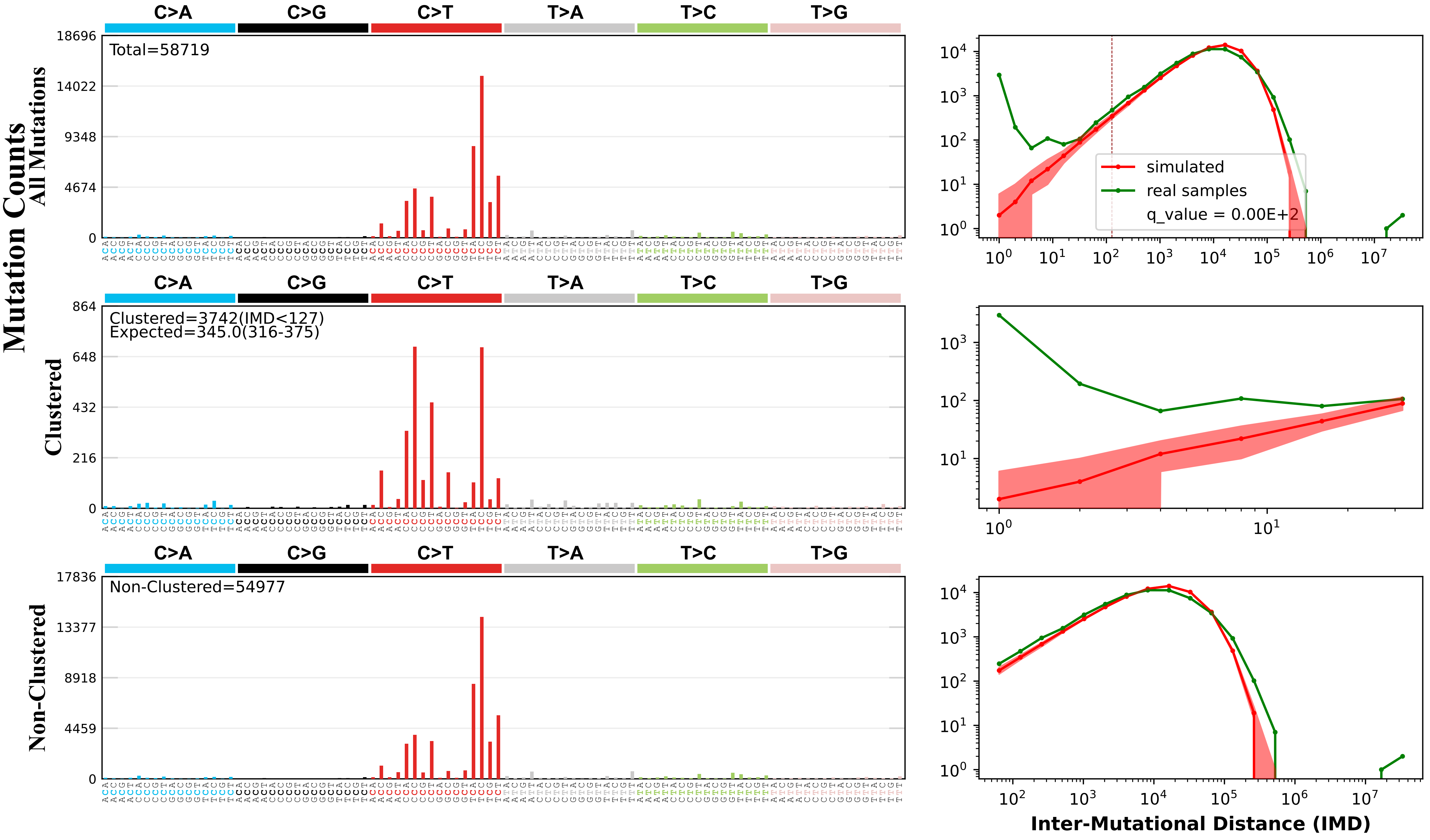

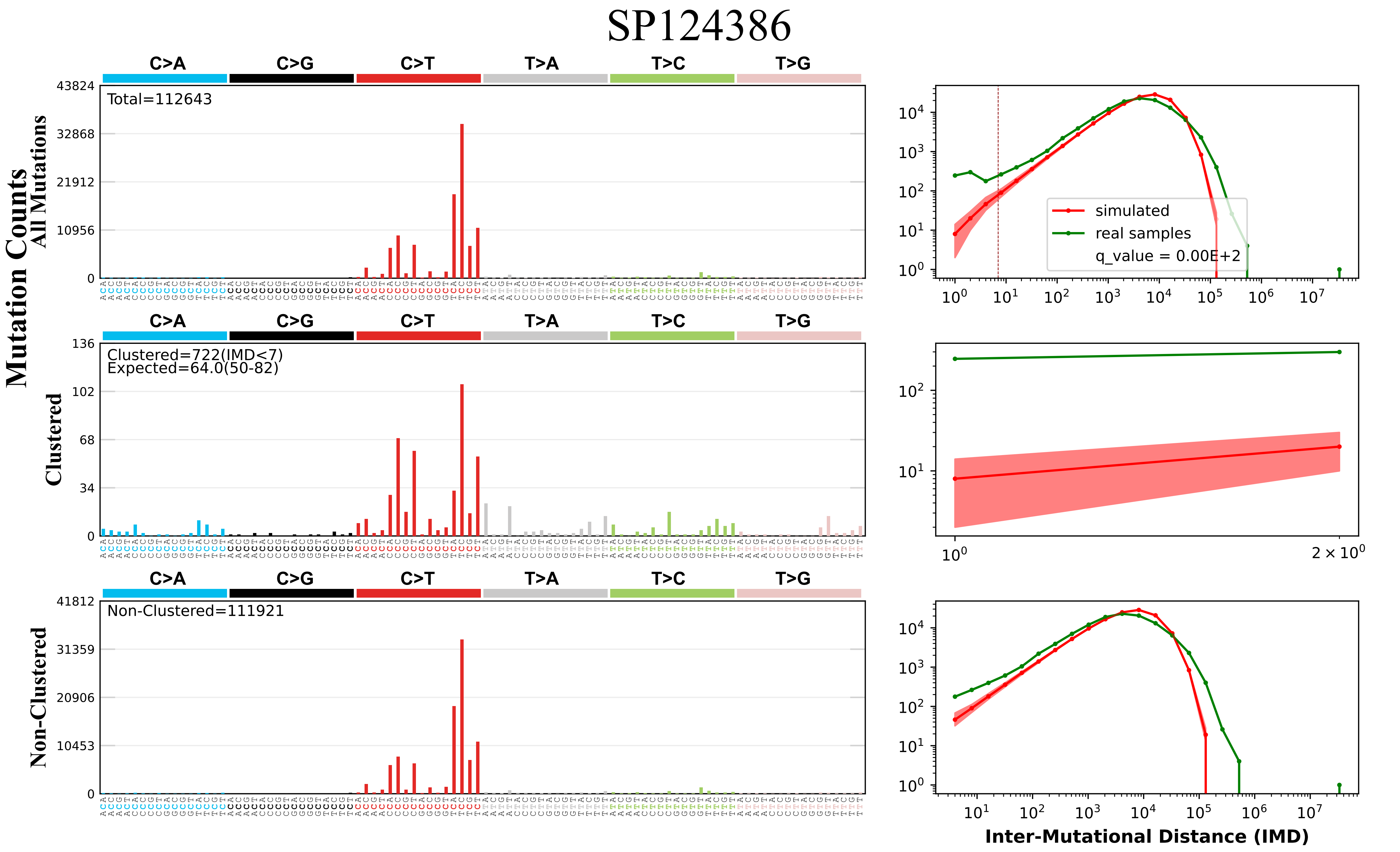
SP124386
C>A
C>G
C>T
T>A
T>C
T>G
Mutation Counts
All Mutations
Total=112643
43824
32868
21912
10956
0
Clustered
Clustered=722(IMD<7)
Expected=64.0(50-82)
136
102
68
34
0
Non-Clustered
Non-Clustered=111921
41812
31359
20906
10453
0
simulated
real samples
q_value = 0.00E+2
10^0
10^1
10^2
10^3
10^4
10^5
10^6
10^7
2×10^0
Inter-Mutational Distance (IMD)

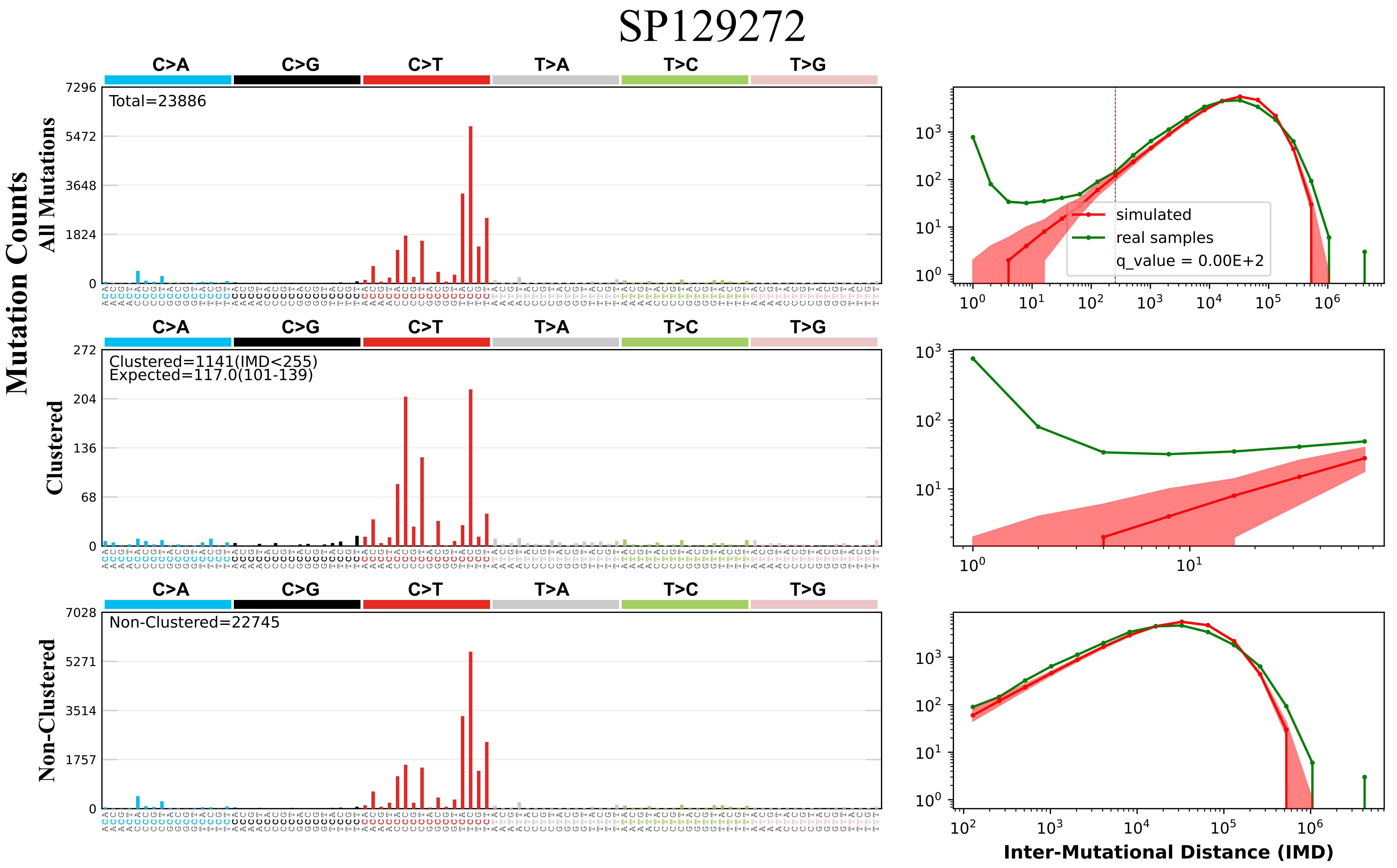

SP129272
C>A
C>G
C>T
T>A
T>C
T>G
Mutation Counts
All Mutations
Total=23886
Clustered
Clustered=1141(IMD<255)
Expected=117.0(101-139)
Non-Clustered
Non-Clustered=22745
simulated
real samples
q_value = 0.00E+2
Inter-Mutational Distance (IMD)

SP124389

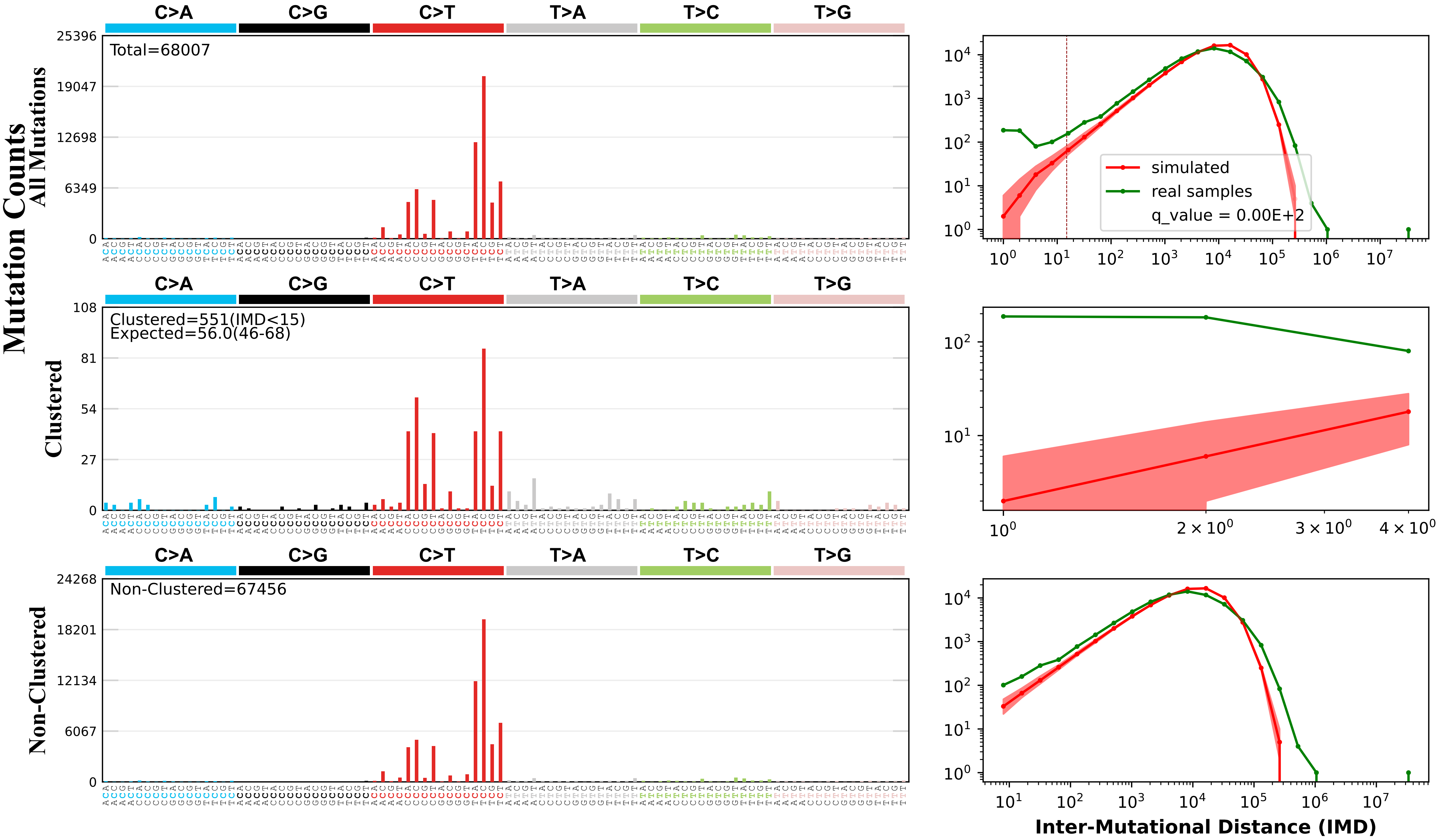

Mutation Counts
All Mutations
Clustered
Non-Clustered
C>A
C>G
C>T
T>A
T>C
T>G
Total=68007
25396
19047
12698
6349
0
Clustered=551(IMD<15)
Expected=56.0(46-68)
108
81
54
27
Non-Clustered=67456
24268
18201
12134
6067
simulated
real samples
q_value = 0.00E+2
Inter-Mutational Distance (IMD)

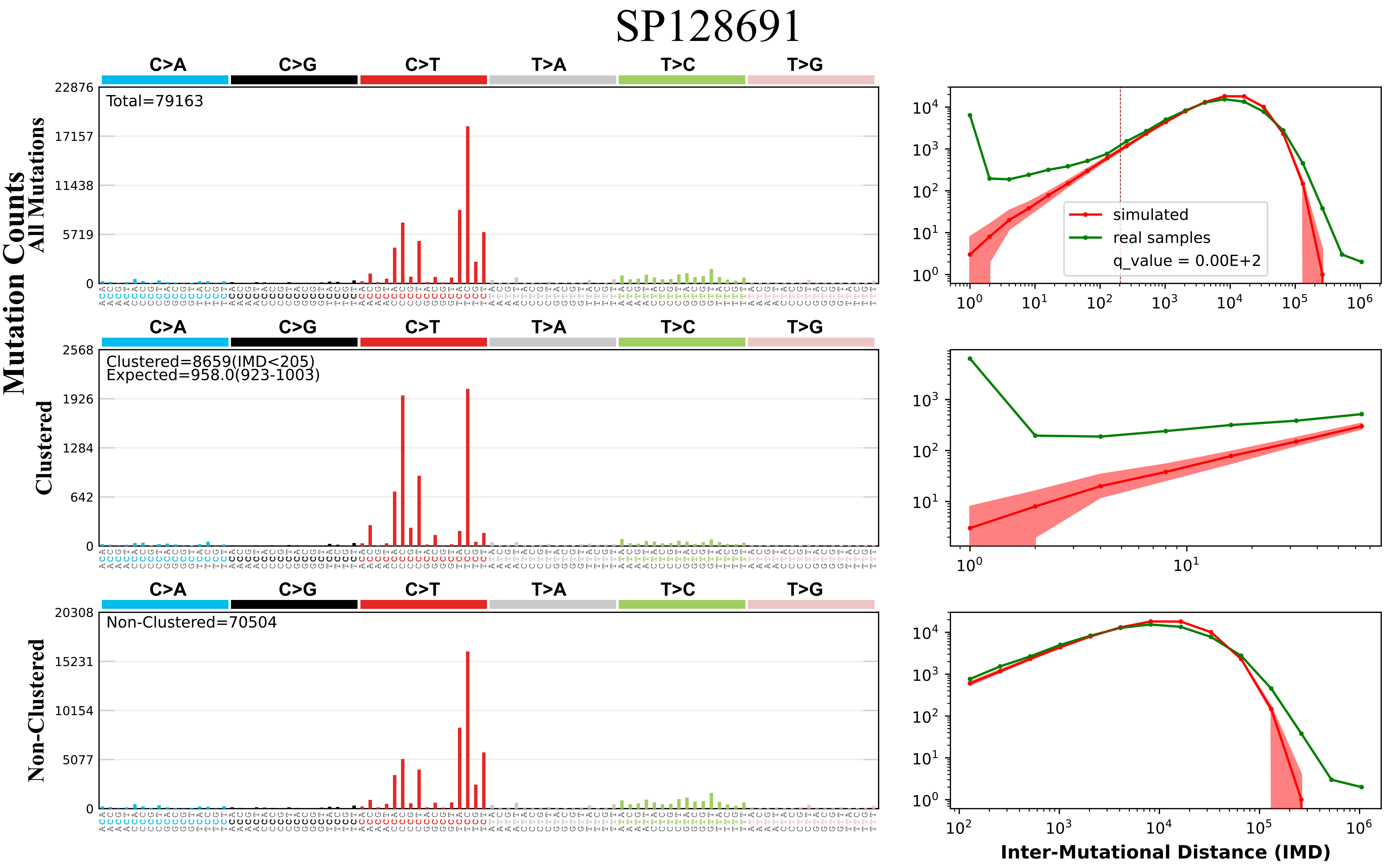

SP128691
Mutation Counts
All Mutations
Clustered
Non-Clustered
C>A
C>G
C>T
T>A
T>C
T>G
Total=79163
Clustered=8659(IMD<205)
Expected=958.0(923-1003)
Non-Clustered=70504
simulated
real samples
q_value = 0.00E+2
Inter-Mutational Distance (IMD)

SP128166

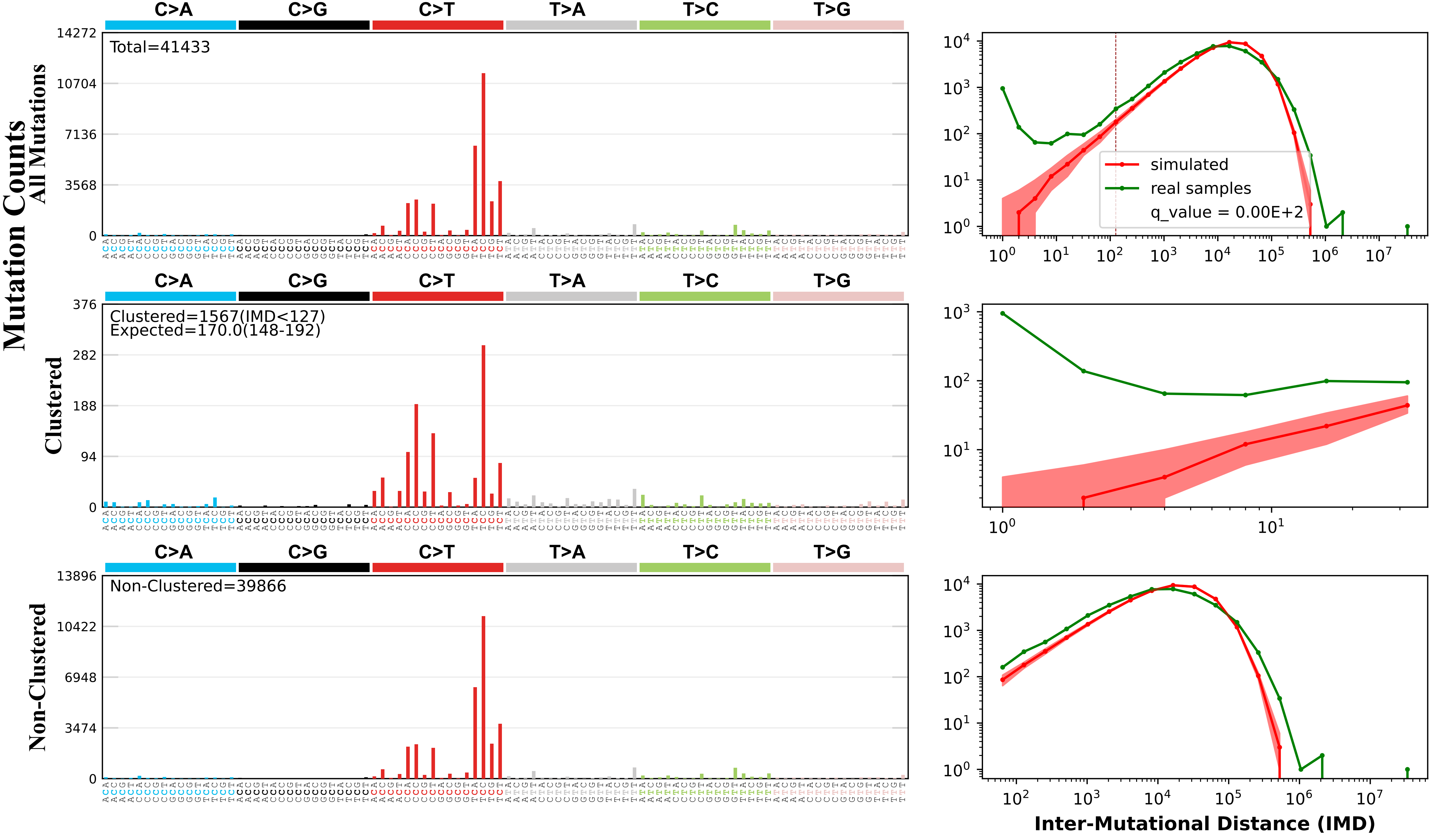

Mutation Counts
All Mutations
Total=41433
Clustered
Clustered=1567(IMD<127)
Expected=170.0(148-192)
Non-Clustered
Non-Clustered=39866
C>A
C>G
C>T
T>A
T>C
T>G
simulated
real samples
q_value = 0.00E+2
Inter-Mutational Distance (IMD)

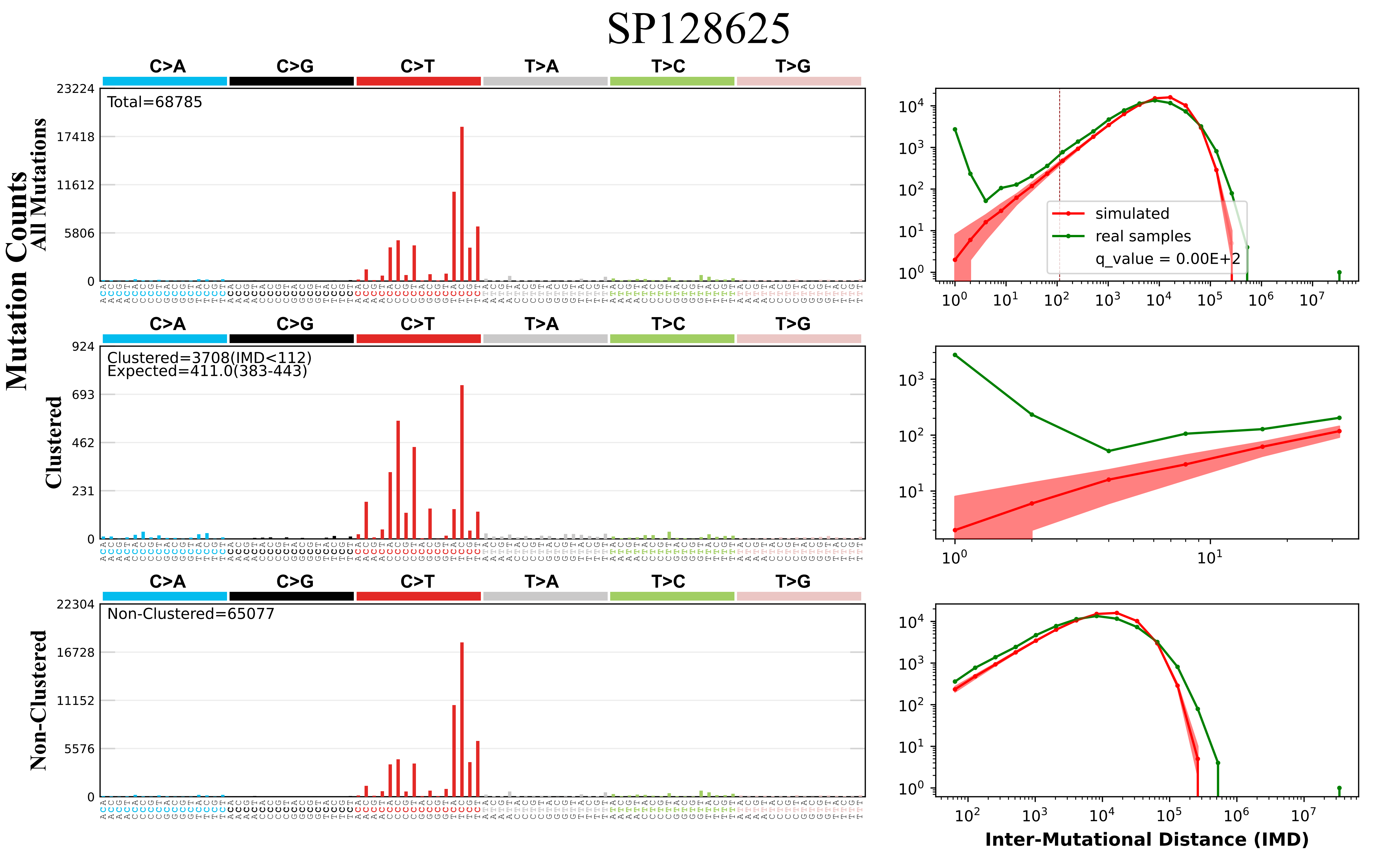
SP128625
Mutation Counts
All Mutations
Total=68785
C>A
C>G
C>T
T>A
T>C
T>G
23224
17418
11612
5806
0
simulated
real samples
q_value = 0.00E+2
Clustered
Clustered=3708(IMD<112)
Expected=411.0(383-443)
924
693
462
231
0
Non-Clustered
Non-Clustered=65077
22304
16728
11152
5576
0
Inter-Mutational Distance (IMD)

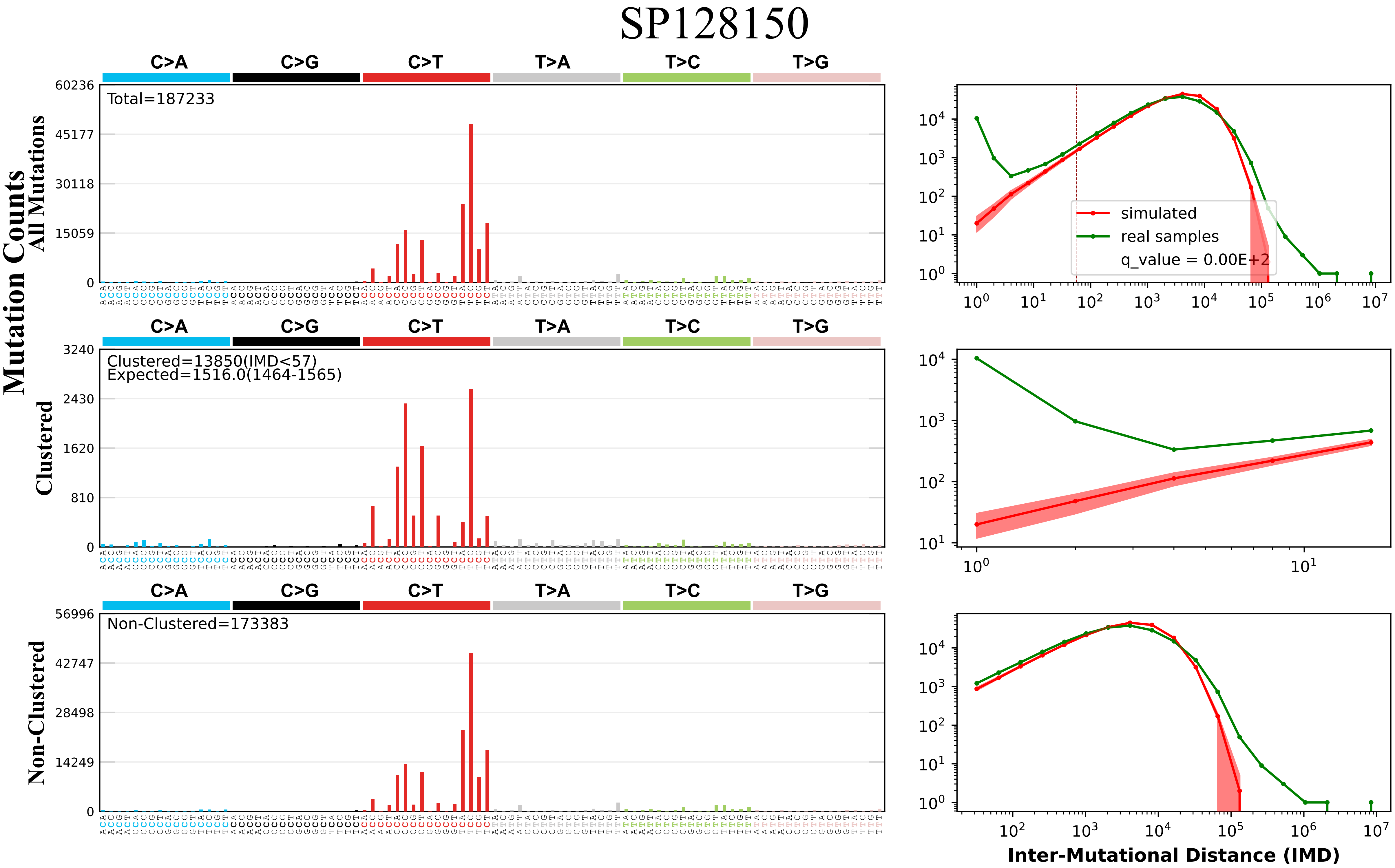
SP128150
C>A
C>G
C>T
T>A
T>C
T>G
Mutation Counts
All Mutations
Total=187233
60236
45177
30118
15059
0
Clustered
Clustered=13850(IMD<57)
Expected=1516.0(1464-1565)
3240
2430
1620
810
0
Non-Clustered
Non-Clustered=173383
56996
42747
28498
14249
0
simulated
real samples
q_value = 0.00E+2
Inter-Mutational Distance (IMD)

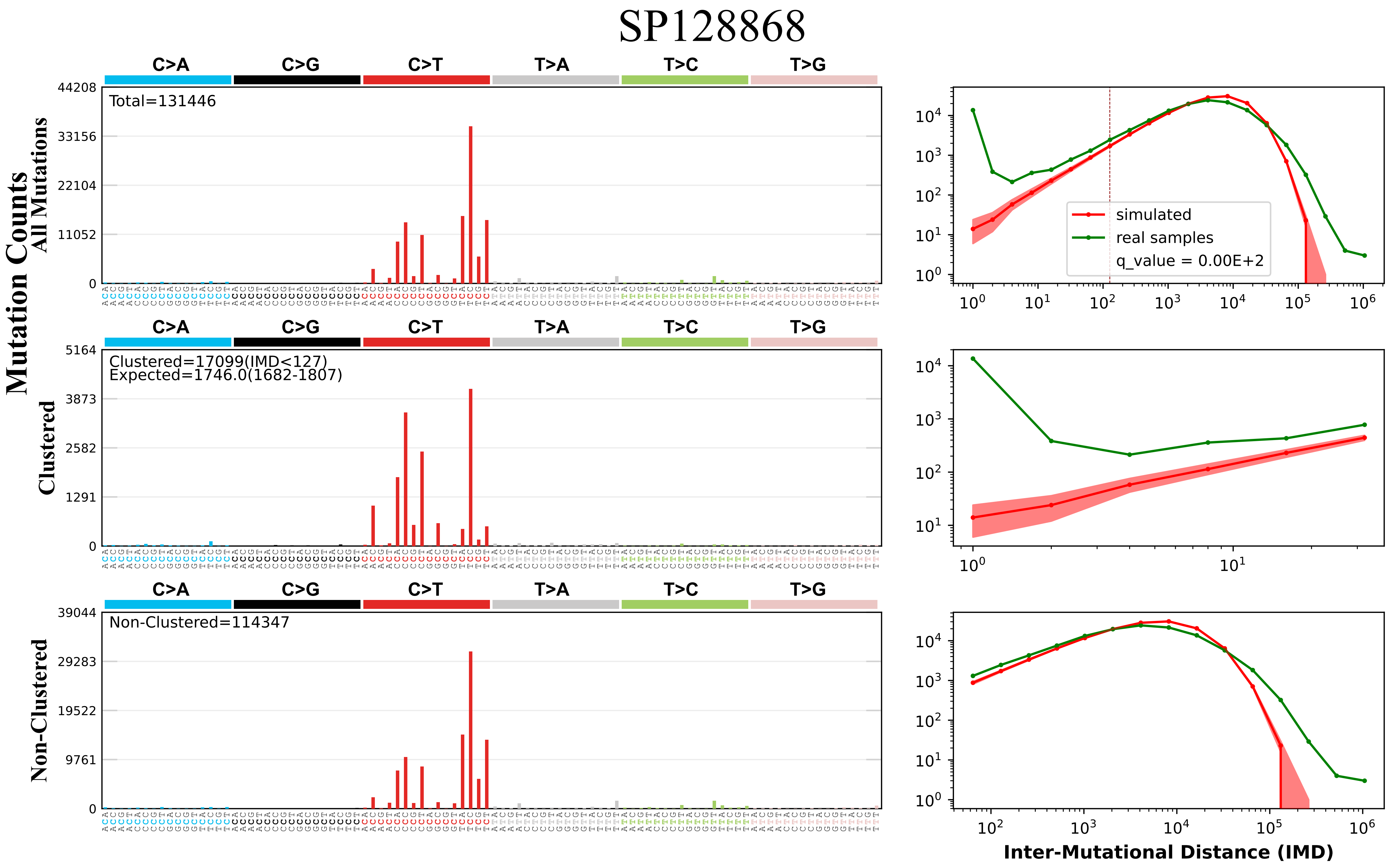
SP128868
C>A
C>G
C>T
T>A
T>C
T>G
Mutation Counts
All Mutations
Total=131446
44208
33156
22104
11052
0
Clustered
Clustered=17099(IMD<127)
Expected=1746.0(1682-1807)
5164
3873
2582
1291
0
Non-Clustered
Non-Clustered=114347
39044
29283
19522
9761
0
simulated
real samples
q_value = 0.00E+2
Inter-Mutational Distance (IMD)

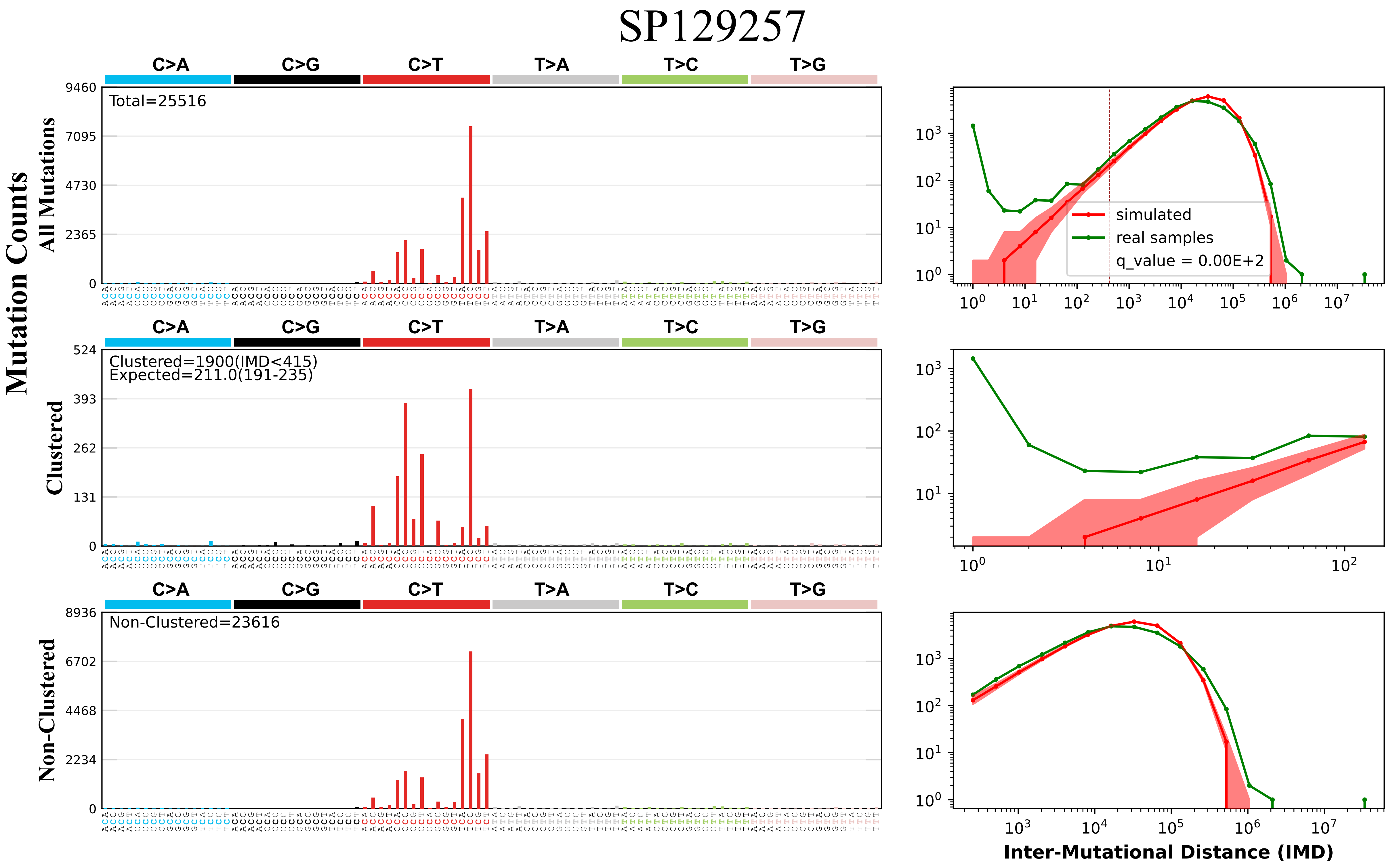
SP129257
Mutation Counts
All Mutations
Total=25516
Clustered
Clustered=1900(IMD<415)
Expected=211.0(191-235)
Non-Clustered
Non-Clustered=23616
C>A
C>G
C>T
T>A
T>C
T>G
simulated
real samples
q_value = 0.00E+2
Inter-Mutational Distance (IMD)

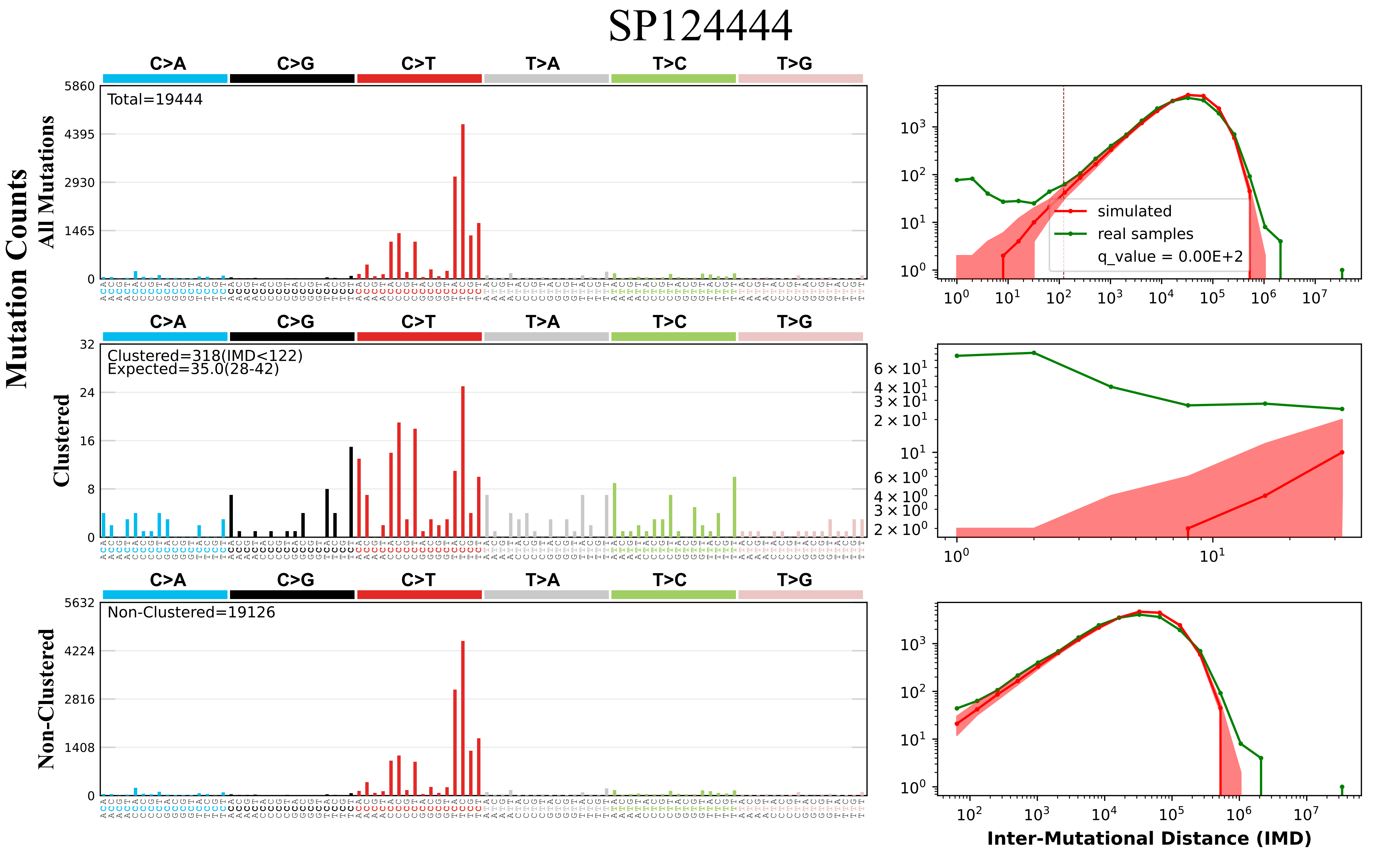
SP124444
Mutation Counts
All Mutations
Total=19444
Clustered
Clustered=318(IMD<122)
Expected=35.0(28-42)
Non-Clustered
Non-Clustered=19126
C>A
C>G
C>T
T>A
T>C
T>G
simulated
real samples
q_value = 0.00E+2
Inter-Mutational Distance (IMD)

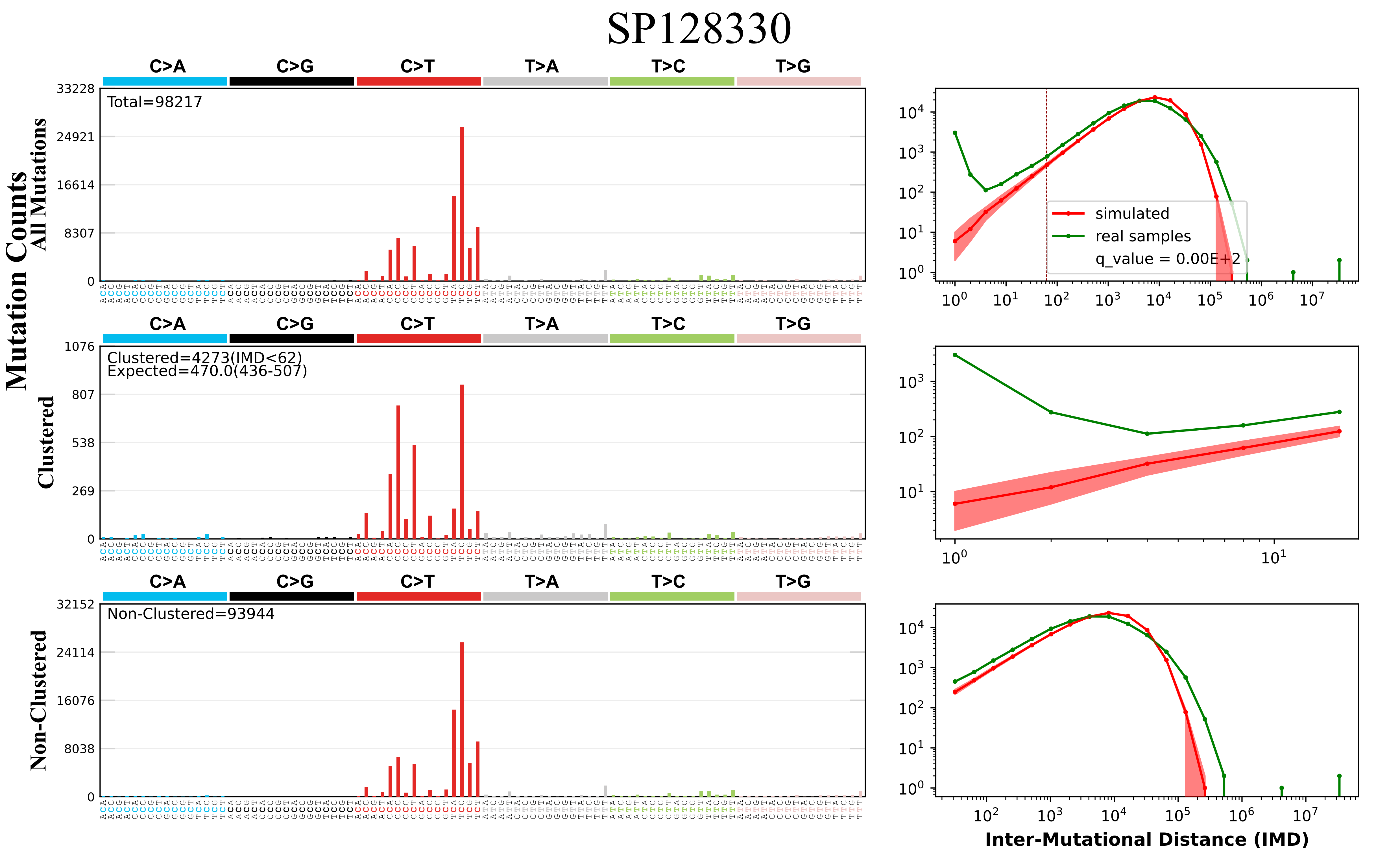
SP128330
Mutation Counts
All Mutations
Clustered
Non-Clustered
C>A
C>G
C>T
T>A
T>C
T>G
Total=98217
Clustered=4273(IMD<62)
Expected=470.0(436-507)
Non-Clustered=93944
simulated
real samples
q_value = 0.00E+2
Inter-Mutational Distance (IMD)

SP128133

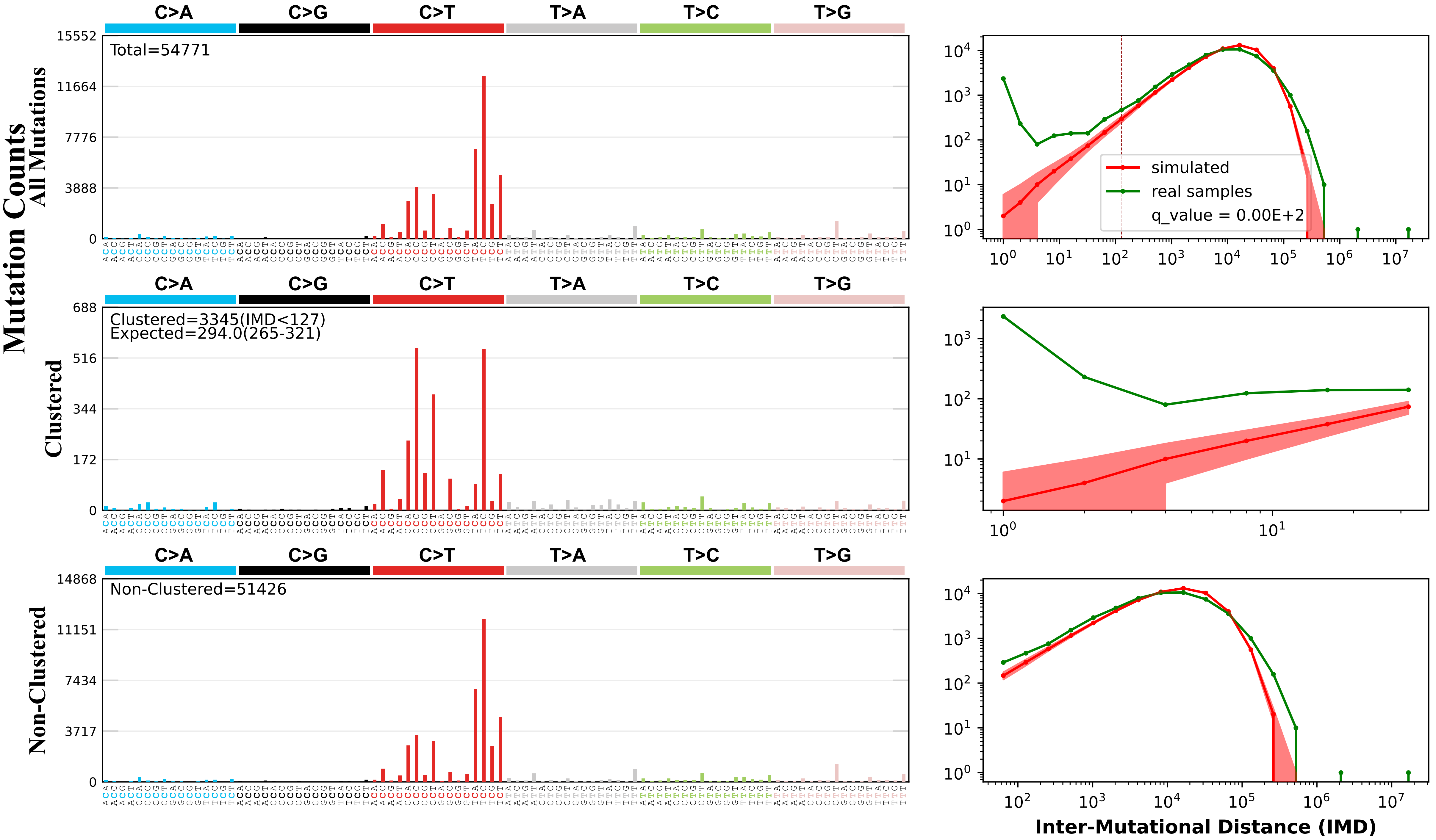

Mutation Counts
All Mutations
Clustered
Non-Clustered
C>A
C>G
C>T
T>A
T>C
T>G
Total=54771
Clustered=3345(IMD<127)
Expected=294.0(265-321)
Non-Clustered=51426
simulated
real samples
q_value = 0.00E+2
Inter-Mutational Distance (IMD)

6c884037-49f9-41c3-b0e5-9cbcd545aeb7

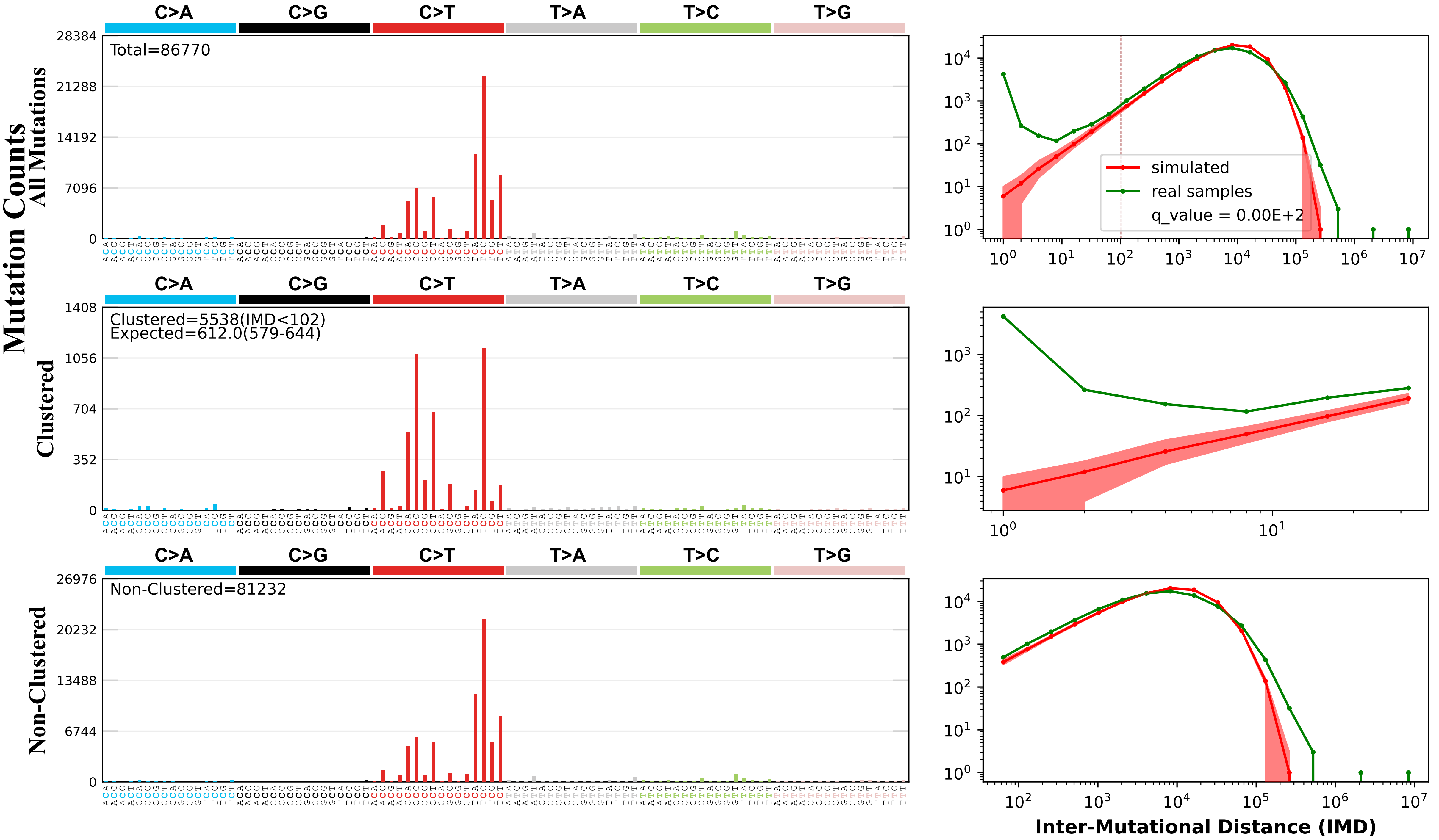

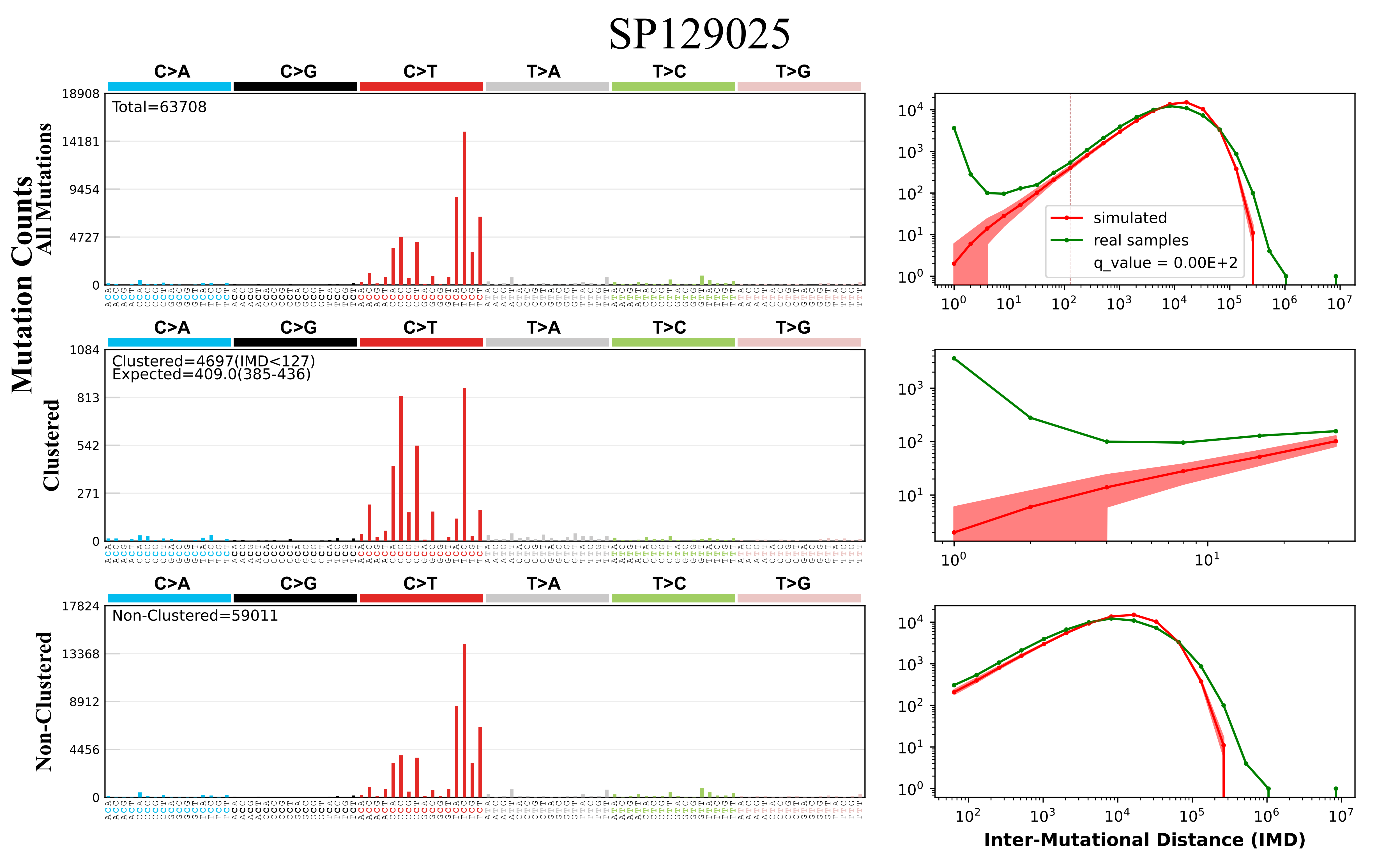
SP129025
Mutation Counts
All Mutations
C>A
C>G
C>T
T>A
T>C
T>G
Total=63708
18908
14181
9454
4727
0
simulated
real samples
q_value = 0.00E+2
Clustered
Clustered=4697(IMD<127)
Expected=409.0(385-436)
1084
813
542
271
Non-Clustered
Non-Clustered=59011
17824
13368
8912
4456
Inter-Mutational Distance (IMD)

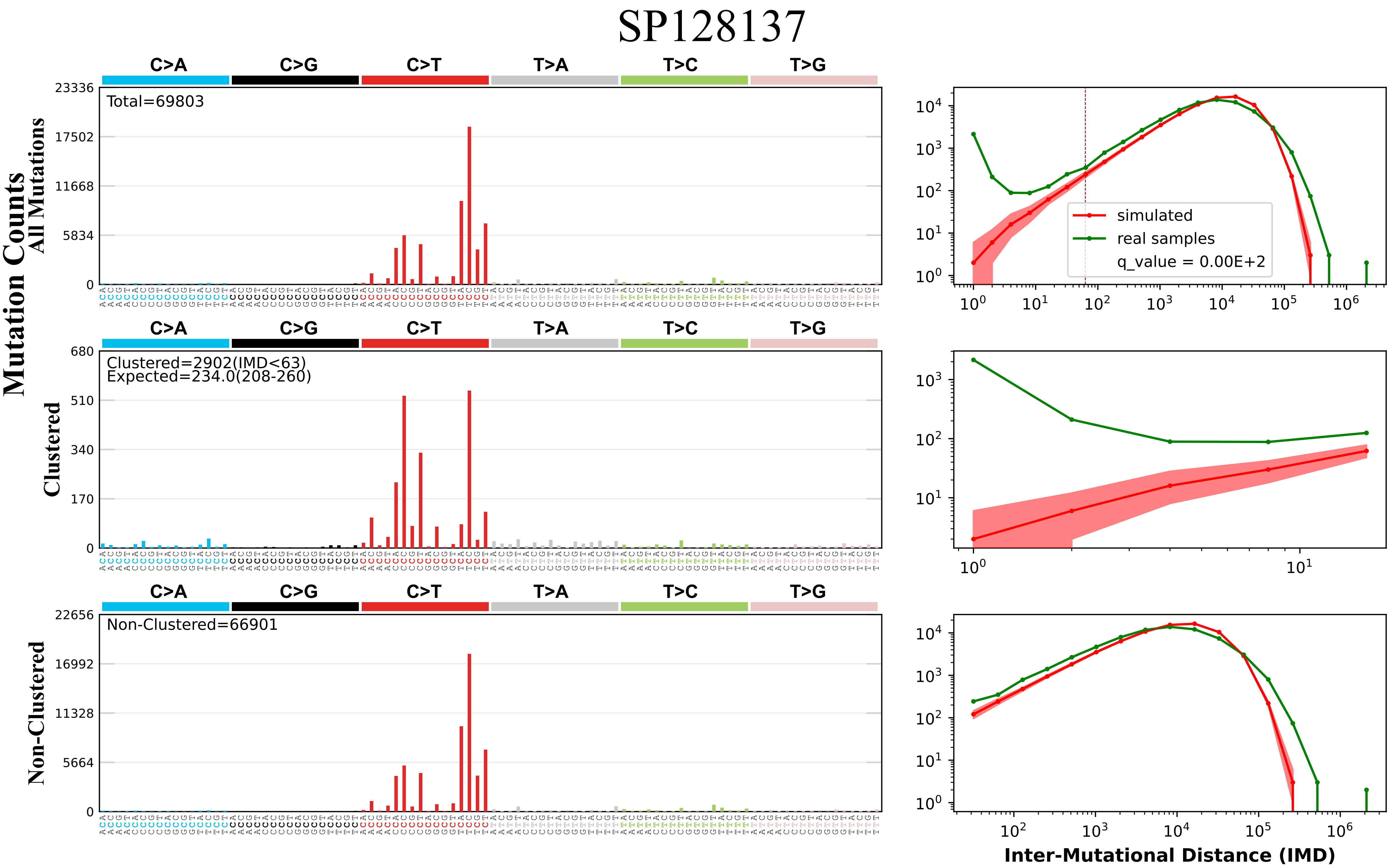
SP128137
C>A
C>G
C>T
T>A
T>C
T>G
Mutation Counts
All Mutations
Total=69803
23336
17502
11668
5834
0
Clustered
Clustered=2902(IMD<63)
Expected=234.0(208-260)
680
510
340
170
0
Non-Clustered
Non-Clustered=66901
22656
16992
11328
5664
0
simulated
real samples
q_value = 0.00E+2
Inter-Mutational Distance (IMD)

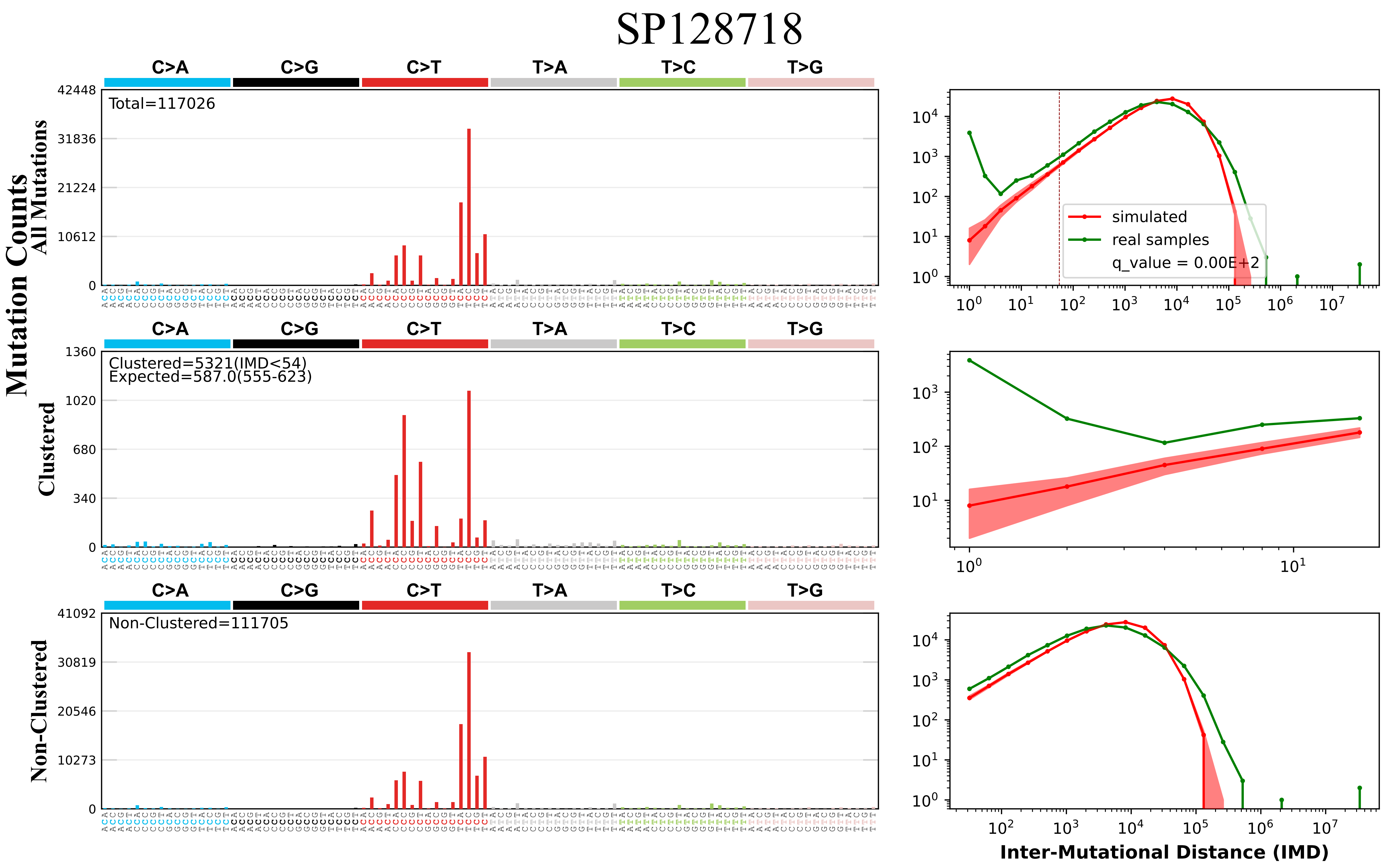

SP128718
Mutation Counts
All Mutations
Total=117026
Clustered
Clustered=5321(IMD<54)
Expected=587.0(555-623)
Non-Clustered
Non-Clustered=111705
C>A
C>G
C>T
T>A
T>C
T>G
simulated
real samples
q_value = 0.00E+2
Inter-Mutational Distance (IMD)

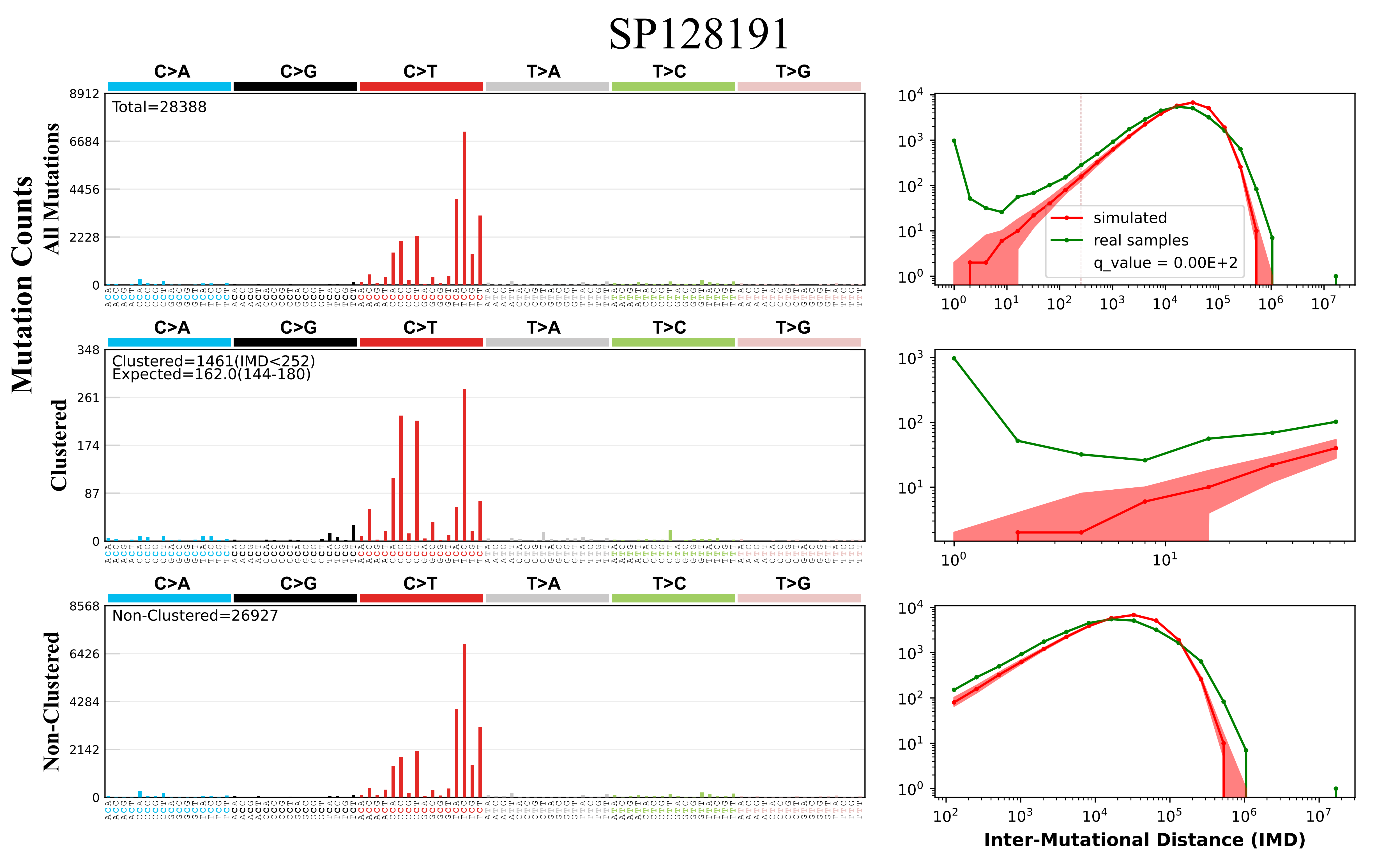
SP128191
Mutation Counts
All Mutations
Clustered
Non-Clustered
C>A
C>G
C>T
T>A
T>C
T>G
Total=28388
Clustered=1461(IMD<252)
Expected=162.0(144-180)
Non-Clustered=26927
simulated
real samples
q_value = 0.00E+2
Inter-Mutational Distance (IMD)

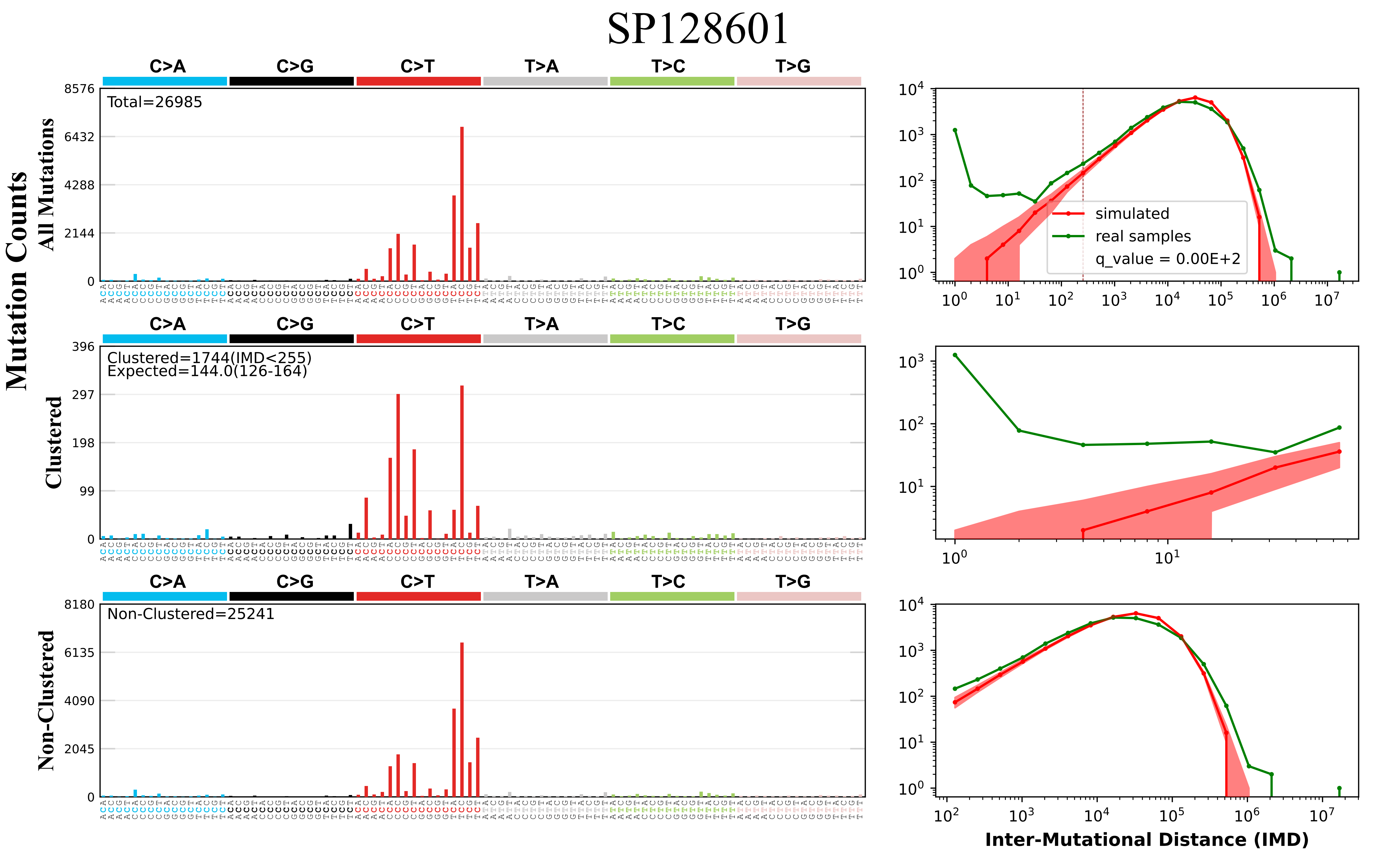
SP128601
Mutation Counts
All Mutations
Clustered
Non-Clustered
C>A
C>G
C>T
T>A
T>C
T>G
Total=26985
Clustered=1744(IMD<255)
Expected=144.0(126-164)
Non-Clustered=25241
simulated
real samples
q_value = 0.00E+2
Inter-Mutational Distance (IMD)

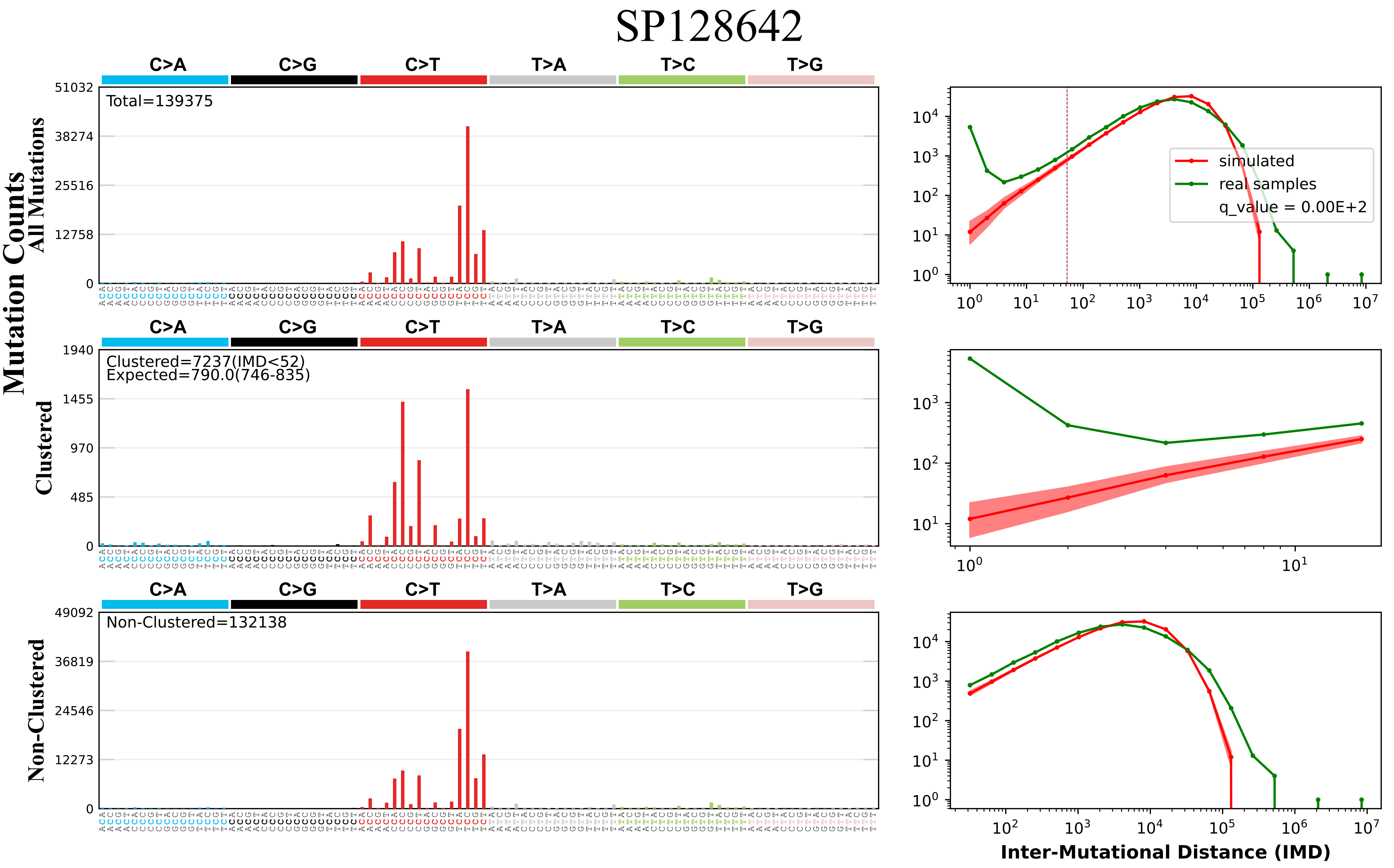
SP128642
Mutation Counts
All Mutations
Clustered
Non-Clustered
C>A
C>G
C>T
T>A
T>C
T>G
Total=139375
51032
38274
25516
12758
0
Clustered=7237(IMD<52)
Expected=790.0(746-835)
1940
1455
970
485
0
Non-Clustered=132138
49092
36819
24546
12273
0
simulated
real samples
q_value = 0.00E+2
Inter-Mutational Distance (IMD)

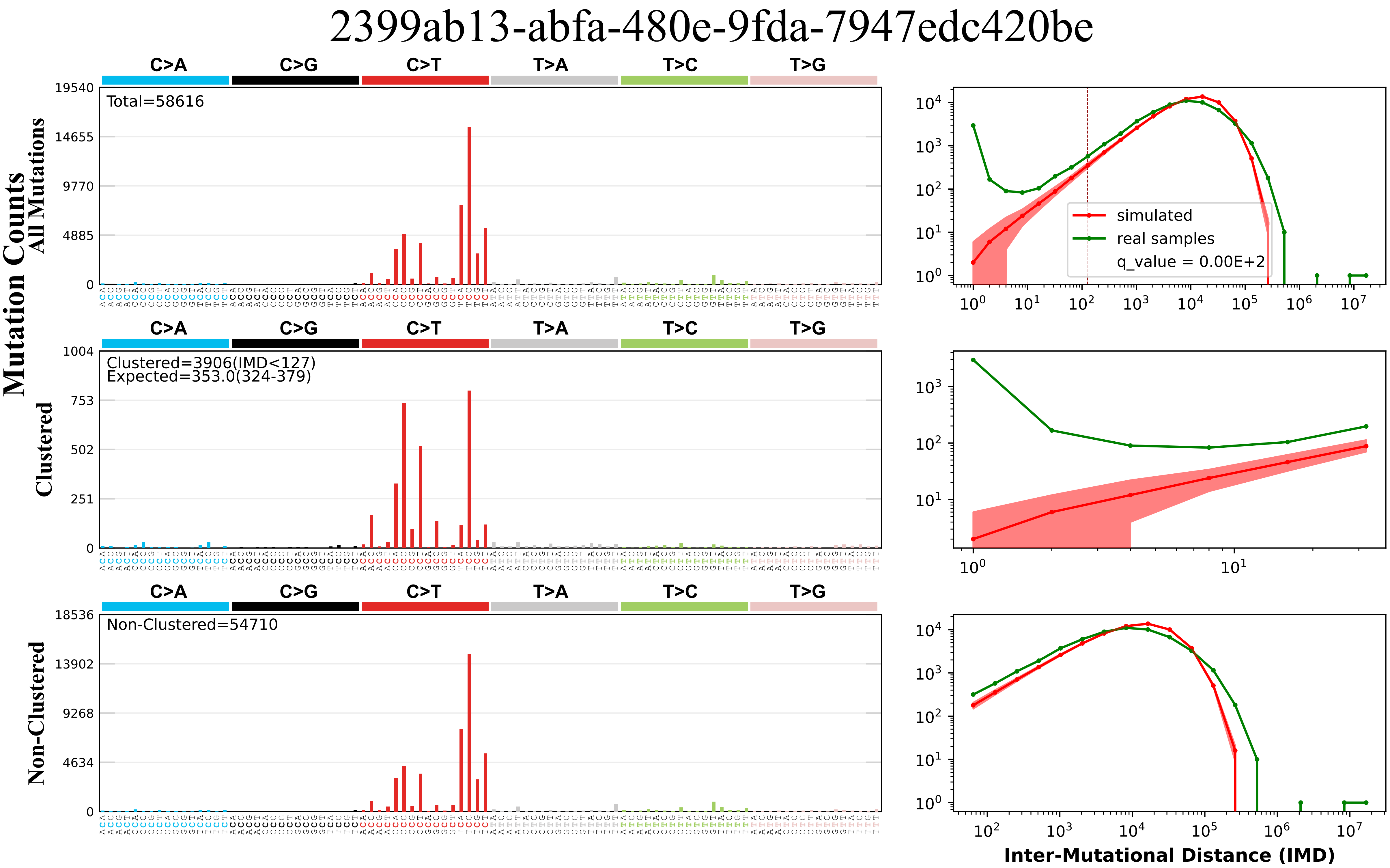
2399ab13-abfa-480e-9fda-7947edc420be
Mutation Counts
All Mutations
Clustered
Non-Clustered
C>A
C>G
C>T
T>A
T>C
T>G
Total=58616
19540
14655
9770
4885
0
Clustered=3906(IMD<127)
Expected=353.0(324-379)
1004
753
502
251
0
Non-Clustered=54710
18536
13902
9268
4634
0
simulated
real samples
q_value = 0.00E+2
Inter-Mutational Distance (IMD)

d053f88f-09e6-472e-a691-bb293c461399

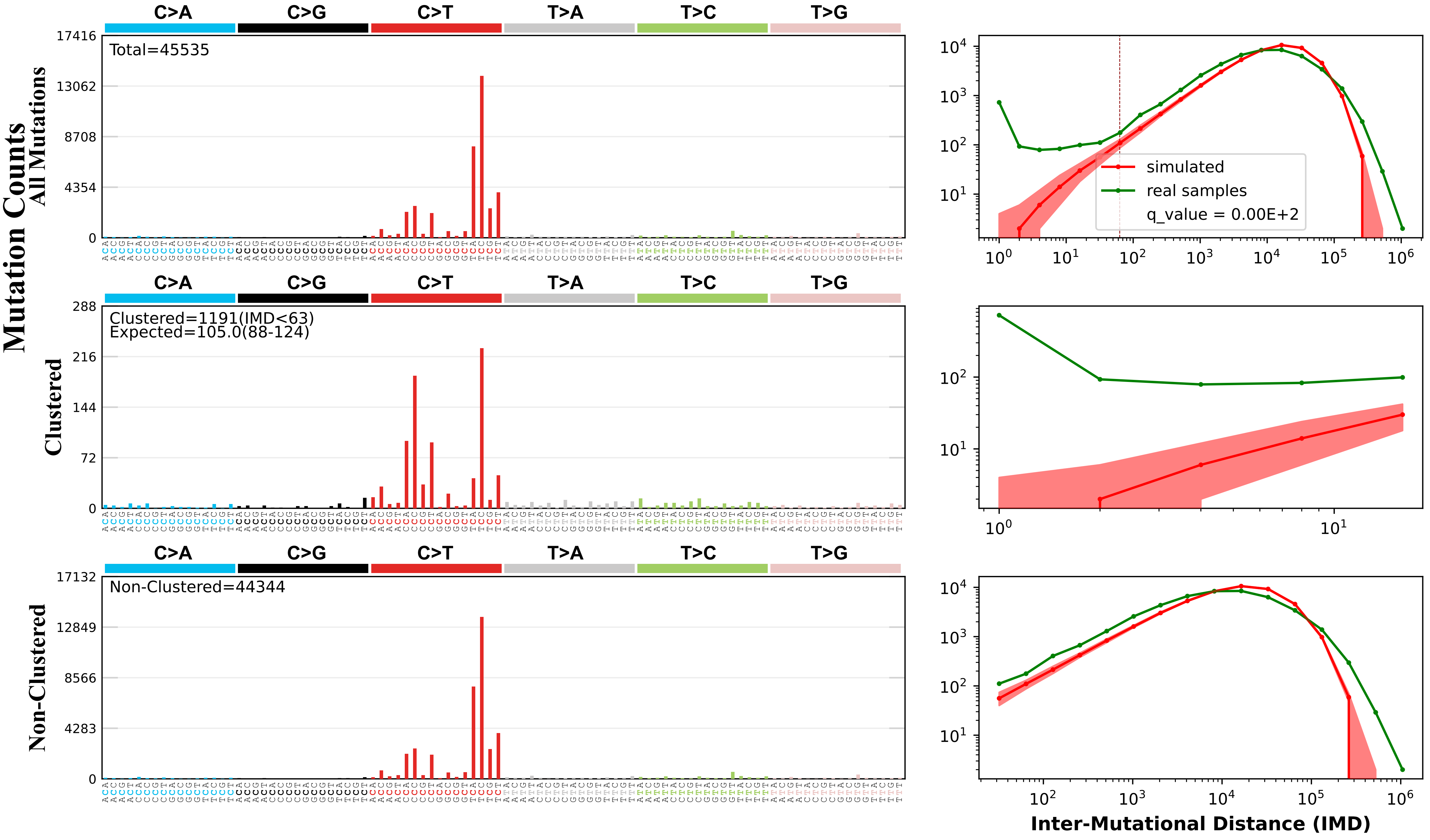

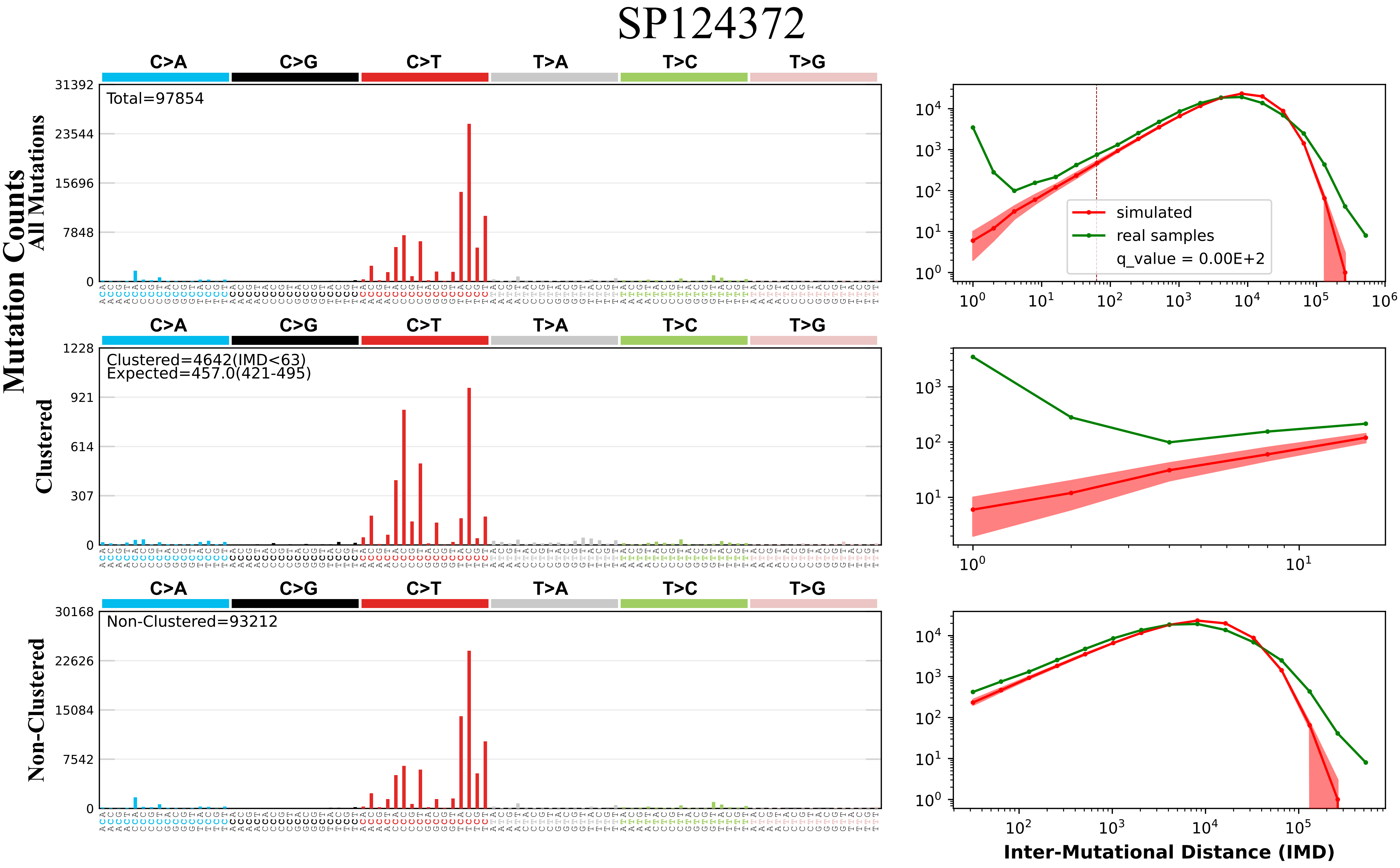
SP124372
C>A
C>G
C>T
T>A
T>C
T>G
Mutation Counts
All Mutations
Total=97854
31392
23544
15696
7848
0
Clustered
Clustered=4642(IMD<63)
Expected=457.0(421-495)
1228
921
614
307
0
Non-Clustered
Non-Clustered=93212
30168
22626
15084
7542
0
simulated
real samples
q_value = 0.00E+2
Inter-Mutational Distance (IMD)

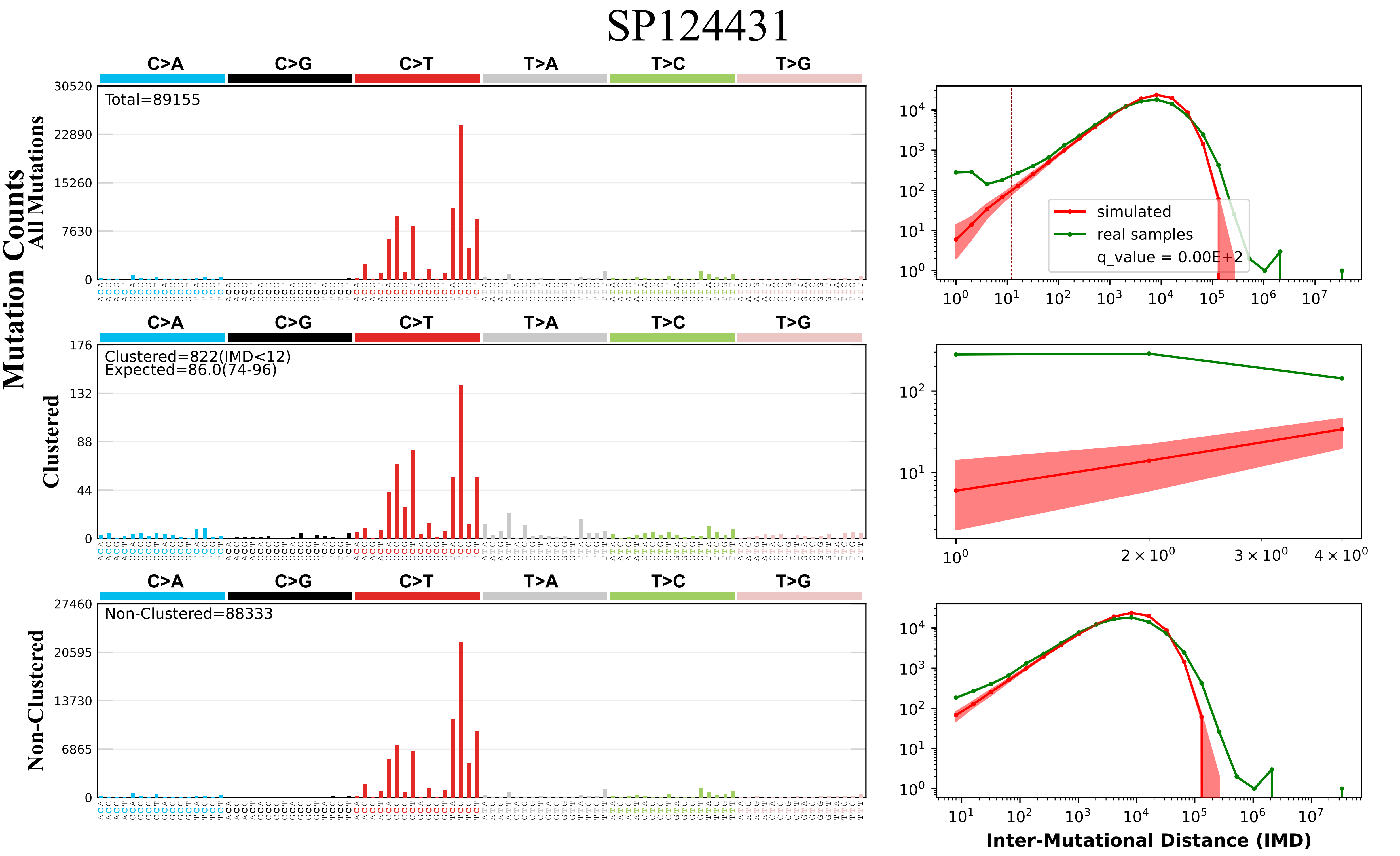
SP124431
C>A
C>G
C>T
T>A
T>C
T>G
Mutation Counts
All Mutations
Total=89155
Clustered
Clustered=822(IMD<12)
Expected=86.0(74-96)
Non-Clustered
Non-Clustered=88333
simulated
real samples
q_value = 0.00E+2
Inter-Mutational Distance (IMD)

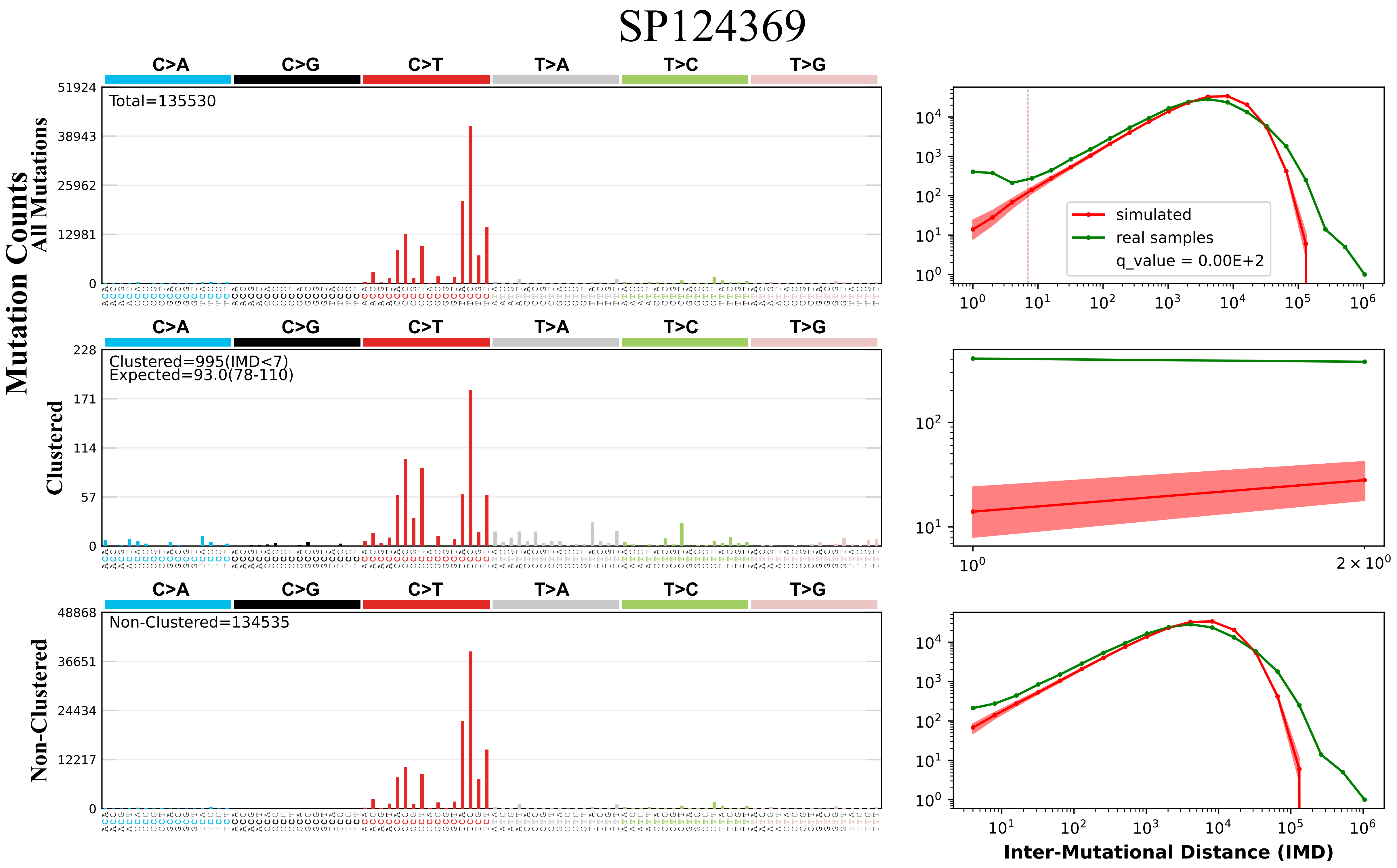
SP124369
Mutation Counts
All Mutations
Clustered
Non-Clustered
C>A
C>G
C>T
T>A
T>C
T>G
Total=135530
Clustered=995(IMD<7)
Expected=93.0(78-110)
Non-Clustered=134535
simulated
real samples
q_value = 0.00E+2
Inter-Mutational Distance (IMD)

0dd0718d-5ddf-4c59-8c47-0f51303daeb5

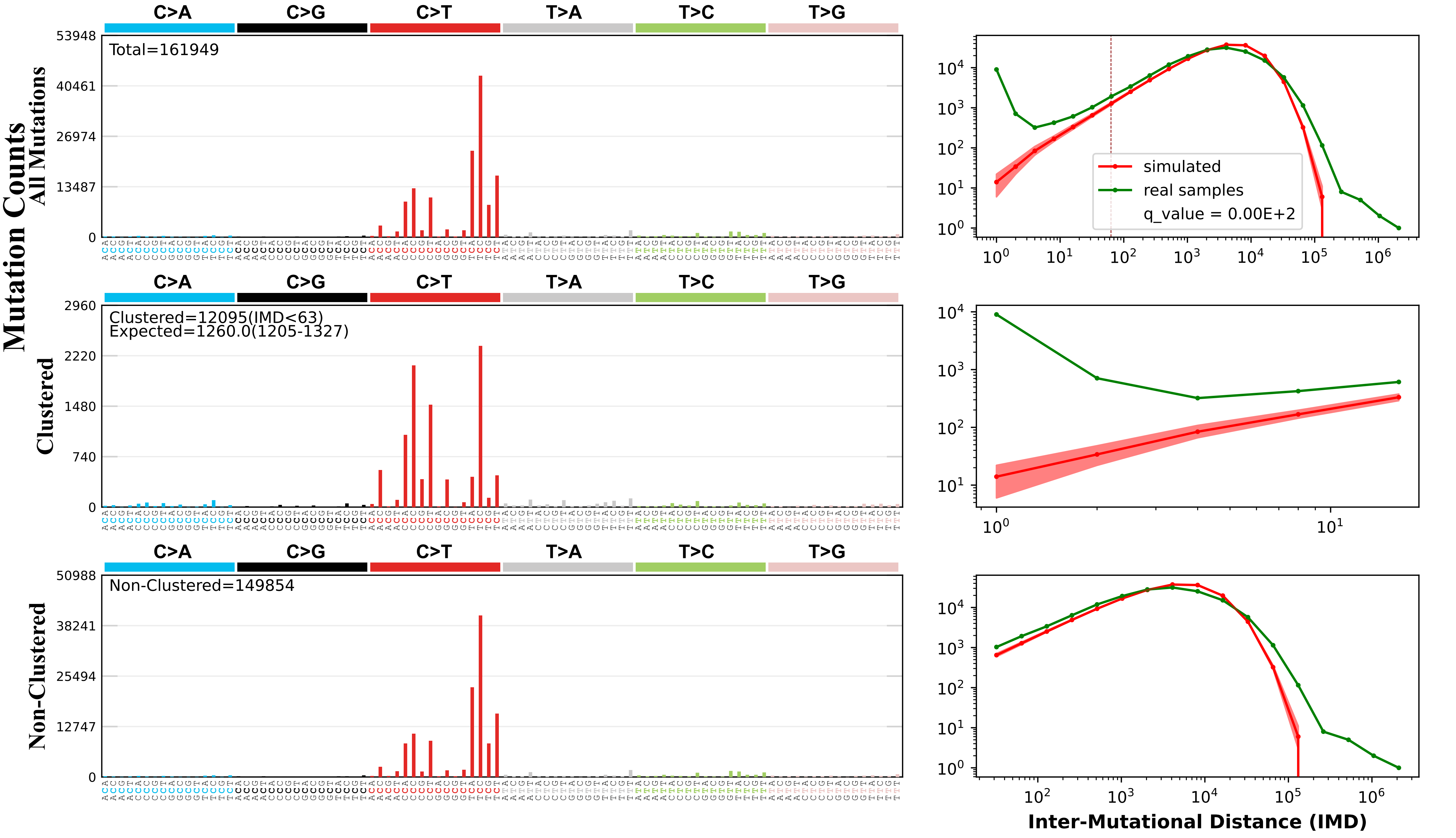

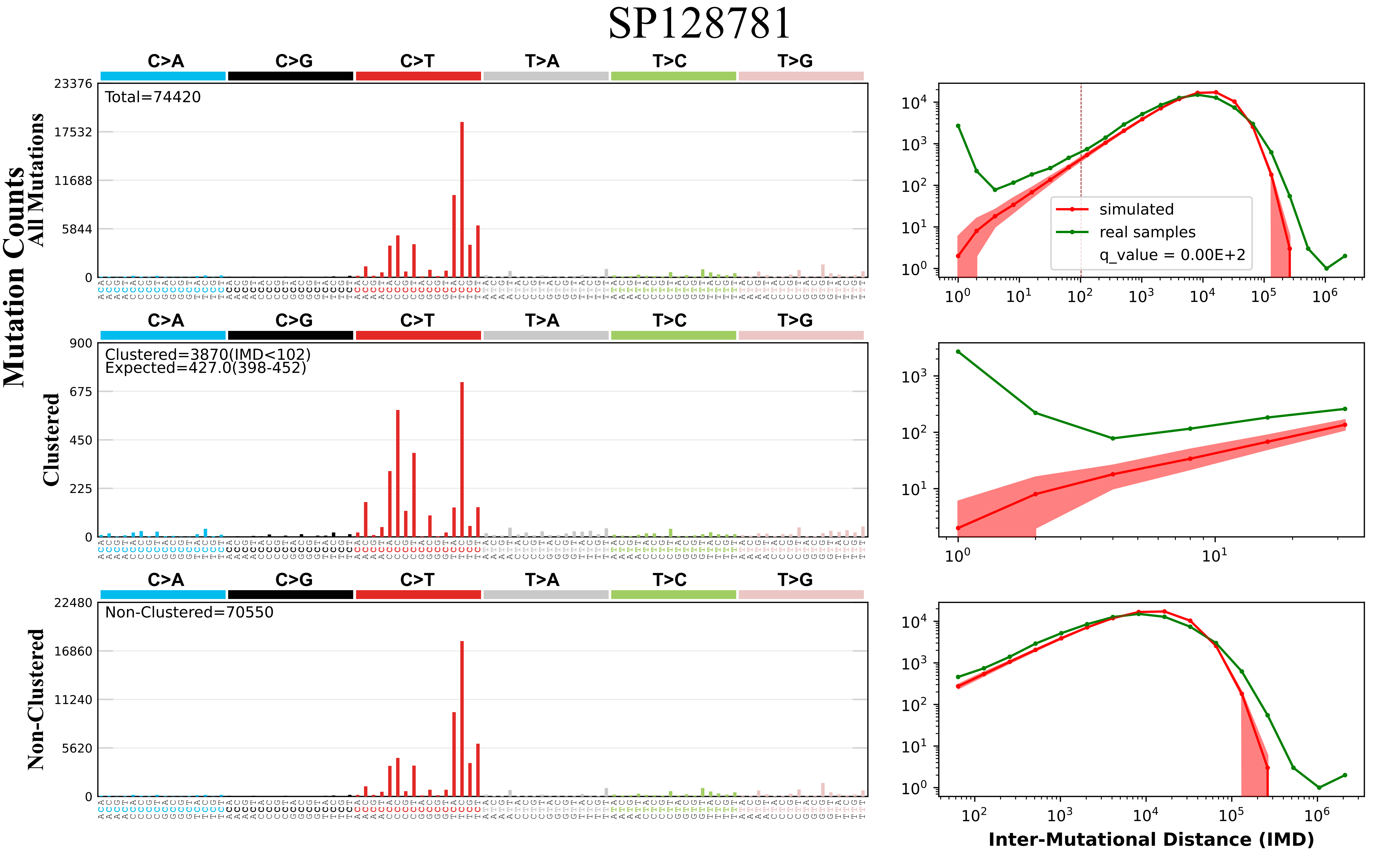
SP128781
Mutation Counts
All Mutations
Clustered
Non-Clustered
C>A
C>G
C>T
T>A
T>C
T>G
Total=74420
Clustered=3870(IMD<102)
Expected=427.0(398-452)
Non-Clustered=70550
simulated
real samples
q_value = 0.00E+2
Inter-Mutational Distance (IMD)

3f2ecbcd-5c9e-4d57-8aa5-962e2e86a83c

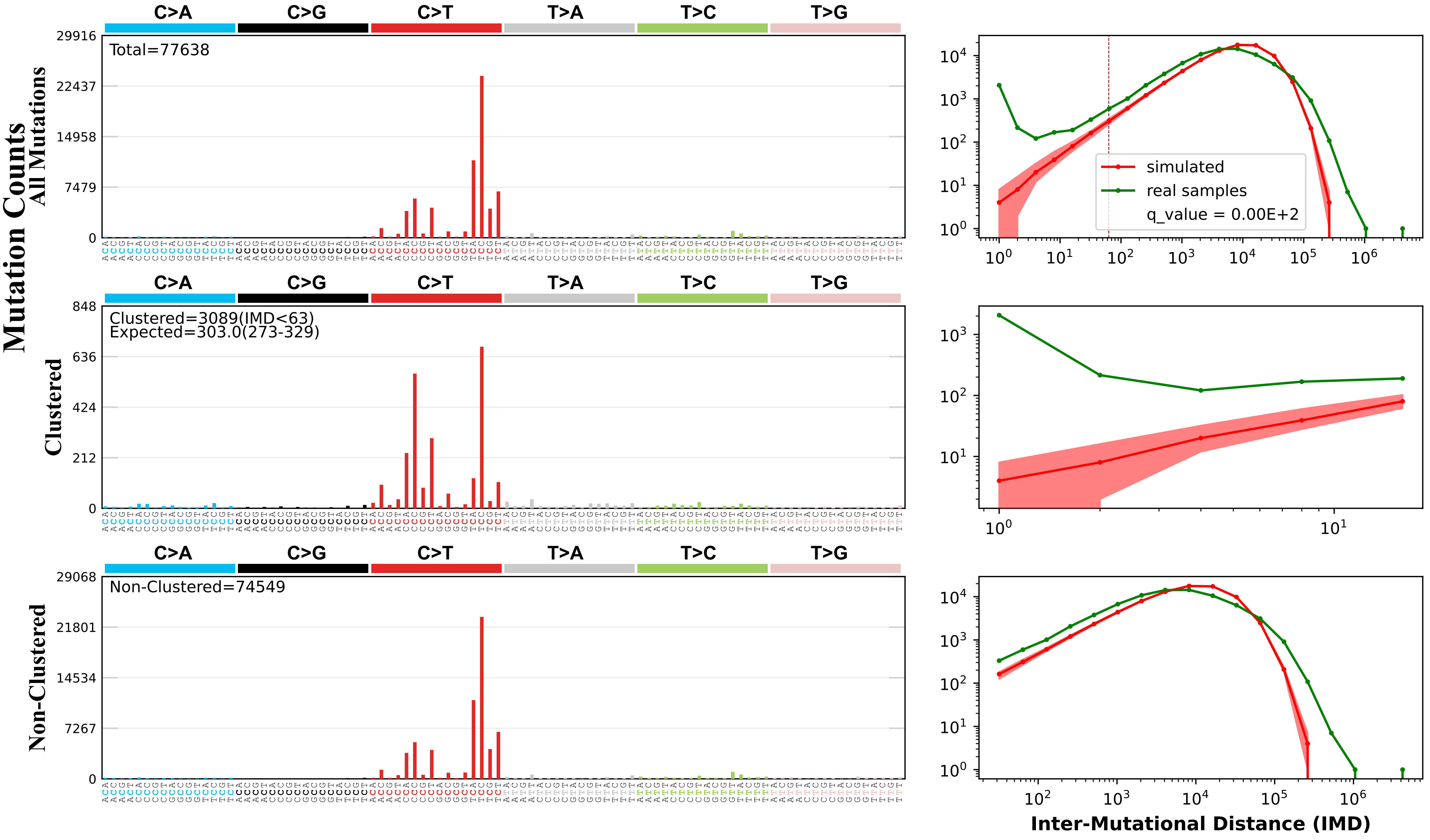

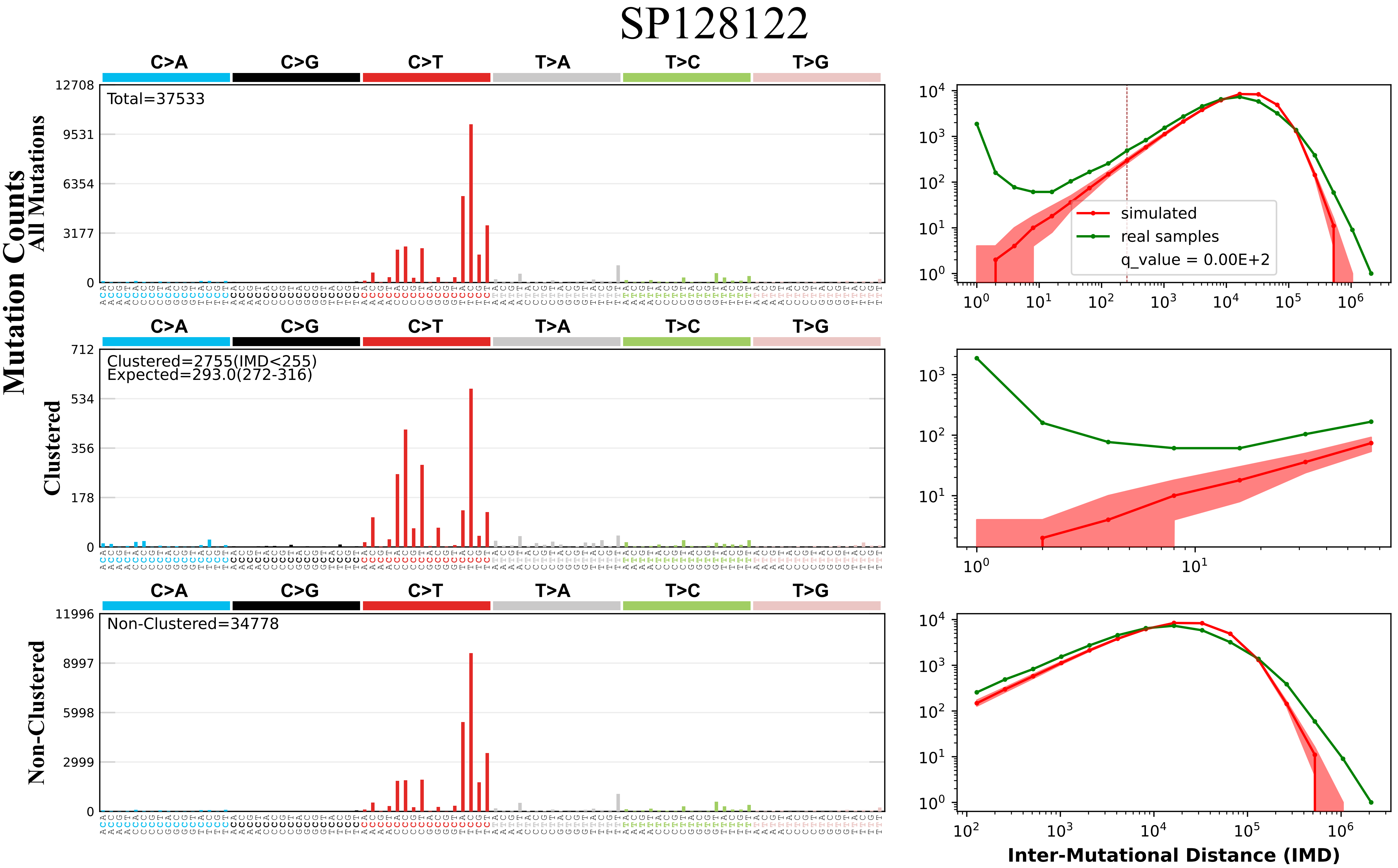
SP128122
Mutation Counts
All Mutations
Total=37533
Clustered
Clustered=2755(IMD<255)
Expected=293.0(272-316)
Non-Clustered
Non-Clustered=34778
C>A
C>G
C>T
T>A
T>C
T>G
simulated
real samples
q_value = 0.00E+2
Inter-Mutational Distance (IMD)

5310f698-d31a-4628-84b4-dc9ac29722a5

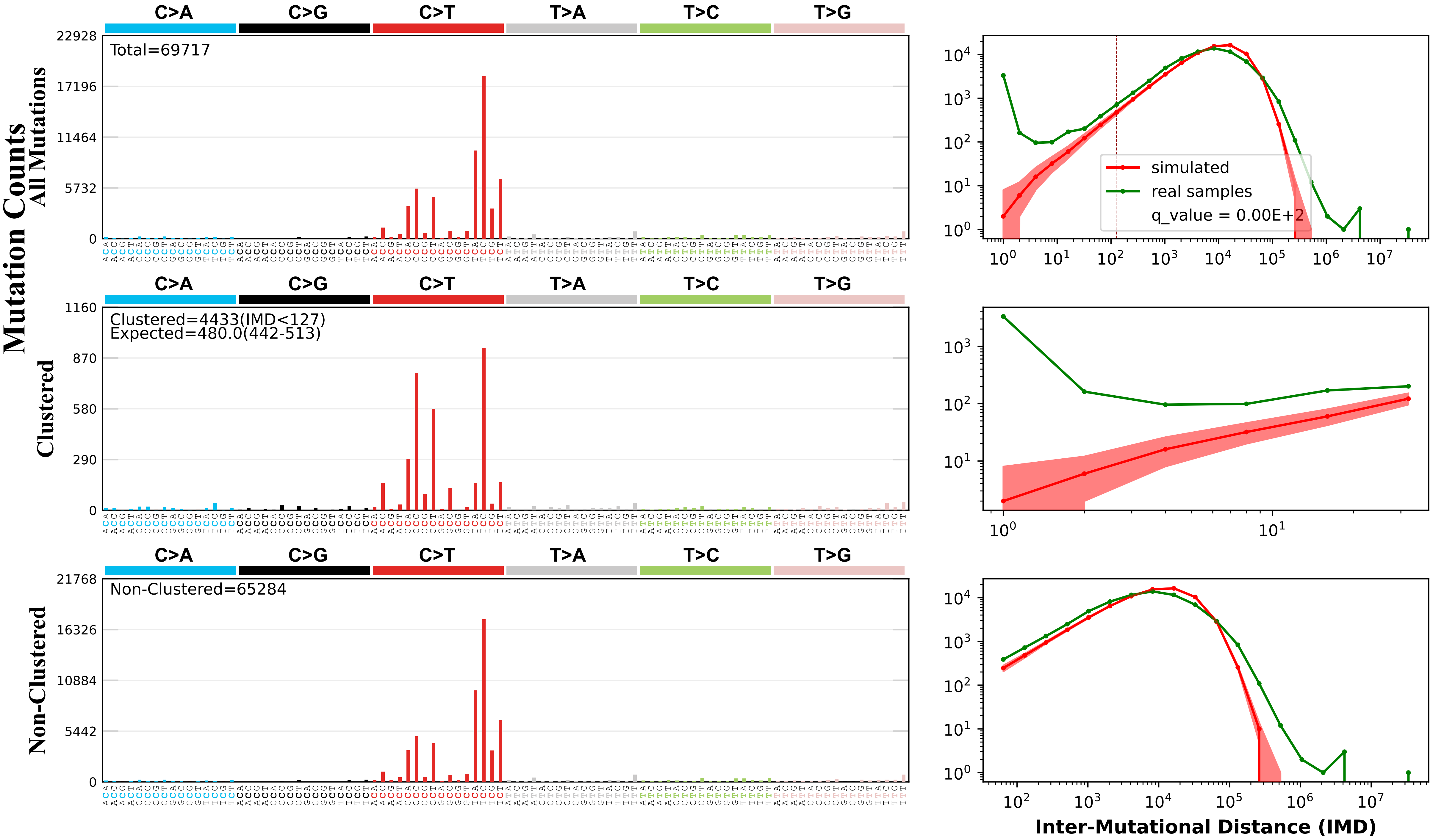

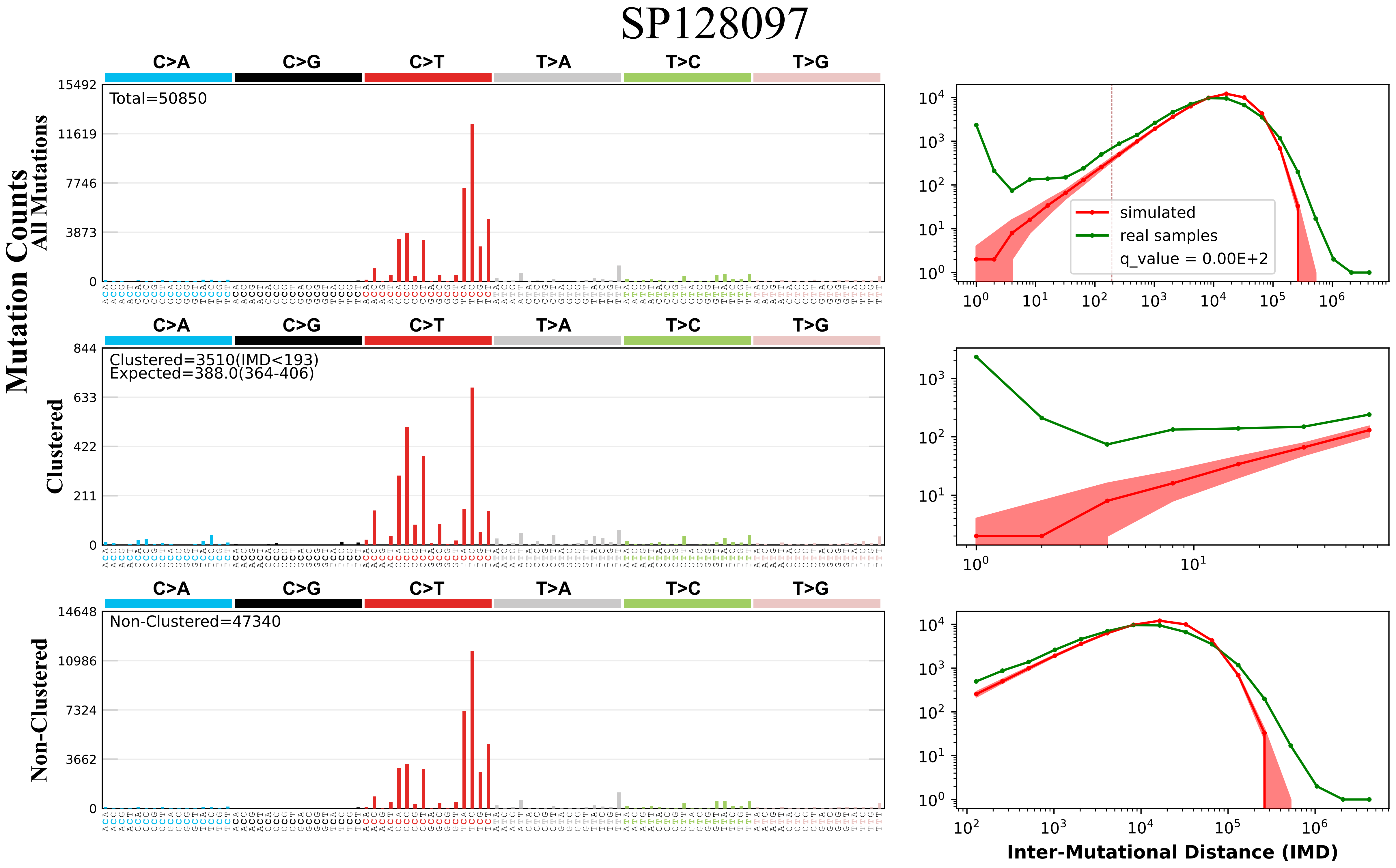
SP128097
Mutation Counts
All Mutations
Total=50850
Clustered
Clustered=3510(IMD<193)
Expected=388.0(364-406)
Non-Clustered
Non-Clustered=47340
C>A
C>G
C>T
T>A
T>C
T>G
simulated
real samples
q_value = 0.00E+2
Inter-Mutational Distance (IMD)

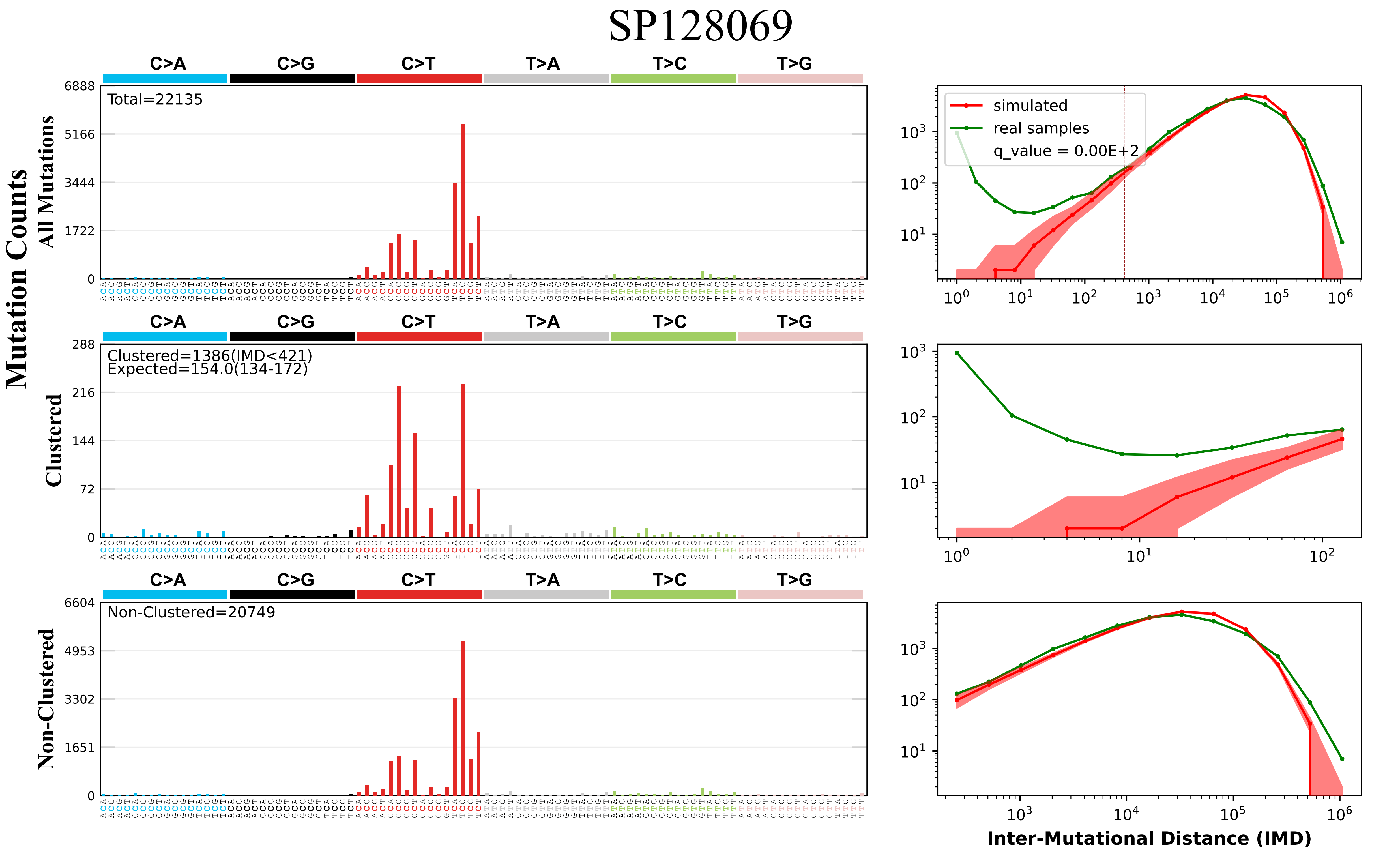
SP128069
Mutation Counts
All Mutations
Total=22135
Clustered
Clustered=1386(IMD<421)
Expected=154.0(134-172)
Non-Clustered
Non-Clustered=20749
C>A
C>G
C>T
T>A
T>C
T>G
simulated
real samples
q_value = 0.00E+2
Inter-Mutational Distance (IMD)

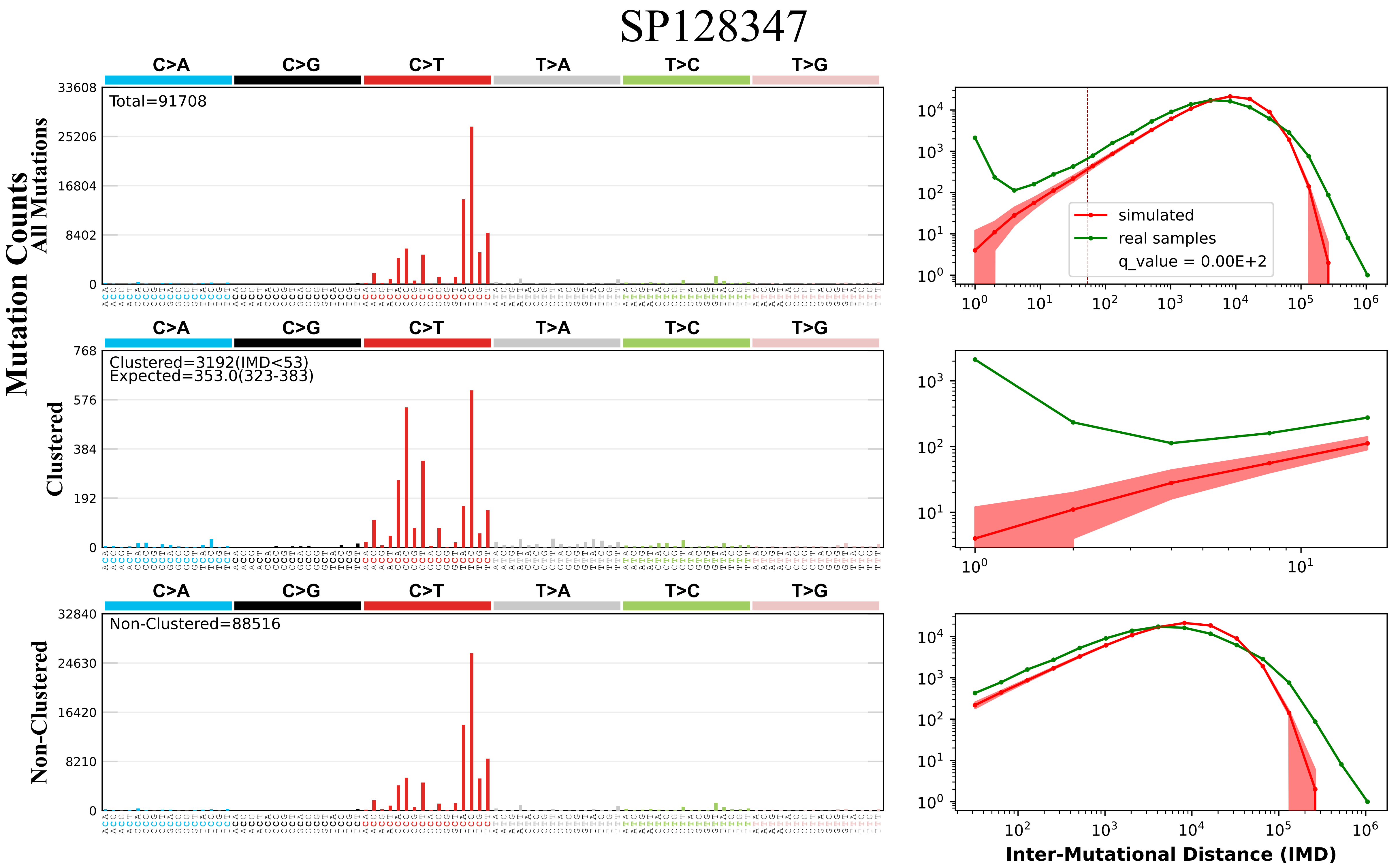
SP128347
C>A
C>G
C>T
T>A
T>C
T>G
Mutation Counts
All Mutations
Total=91708
33608
25206
16804
8402
0
simulated
real samples
q_value = 0.00E+2
Clustered
Clustered=3192(IMD<53)
Expected=353.0(323-383)
768
576
384
192
Non-Clustered
Non-Clustered=88516
32840
24630
16420
8210
Inter-Mutational Distance (IMD)

SP124326

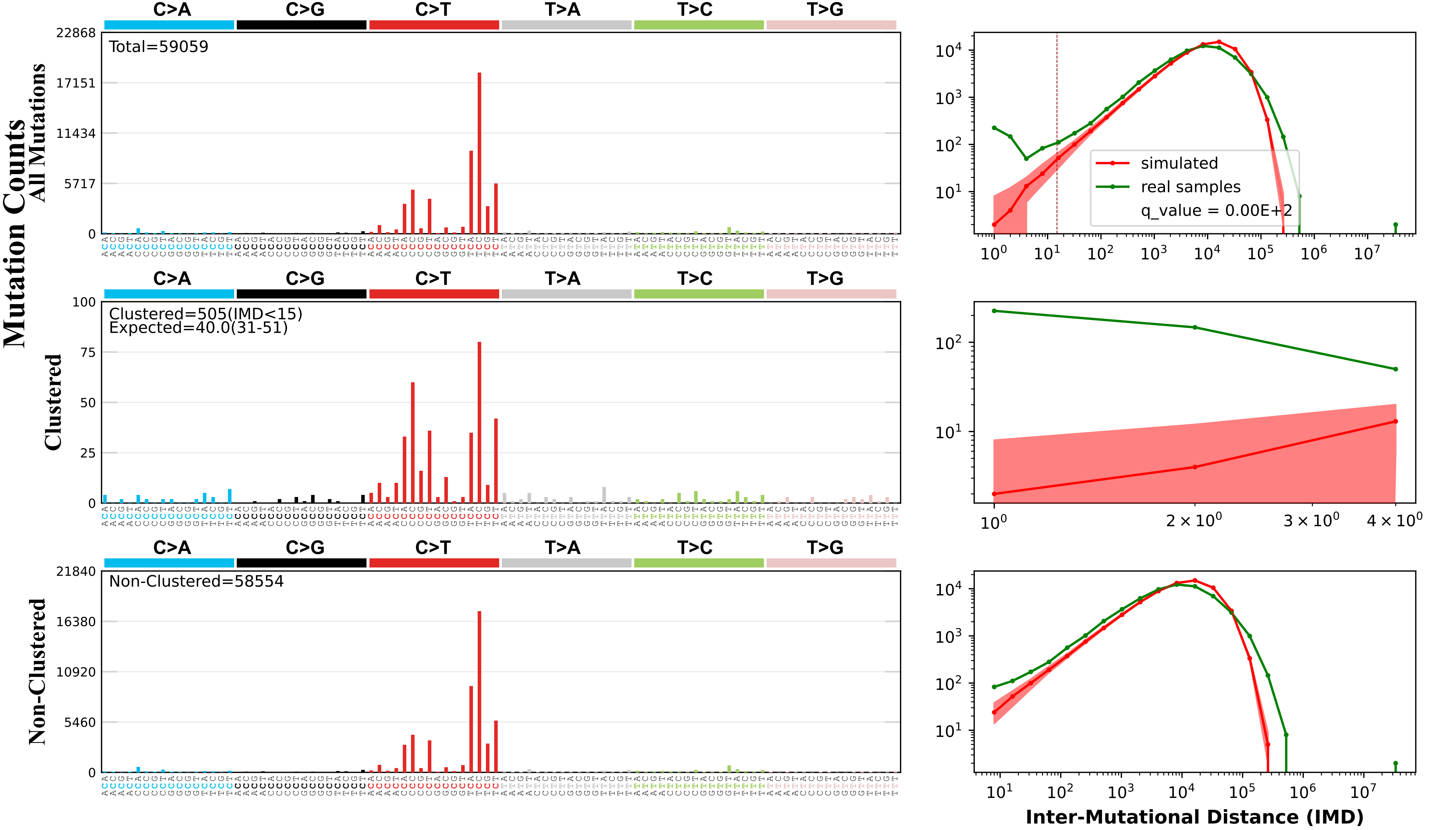

Mutation Counts
All Mutations
Total=59059
Clustered
Clustered=505(IMD<15)
Expected=40.0(31-51)
Non-Clustered
Non-Clustered=58554
C>A
C>G
C>T
T>A
T>C
T>G
simulated
real samples
q_value = 0.00E+2
Inter-Mutational Distance (IMD)

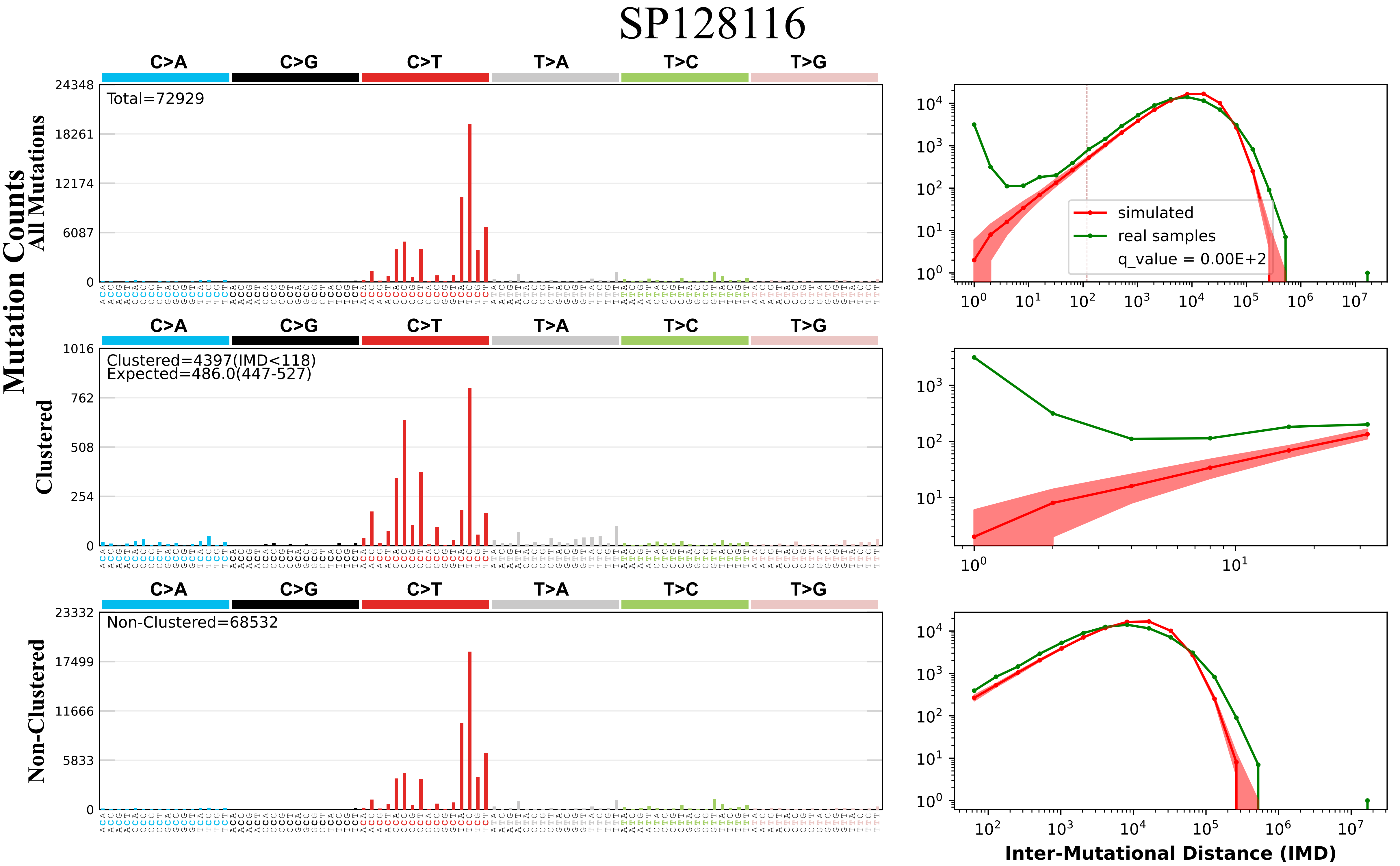

SP128116
Mutation Counts
All Mutations
Clustered
Non-Clustered
C>A
C>G
C>T
T>A
T>C
T>G
Total=72929
Clustered=4397(IMD<118)
Expected=486.0(447-527)
Non-Clustered=68532
simulated
real samples
q_value = 0.00E+2
Inter-Mutational Distance (IMD)

SP124420

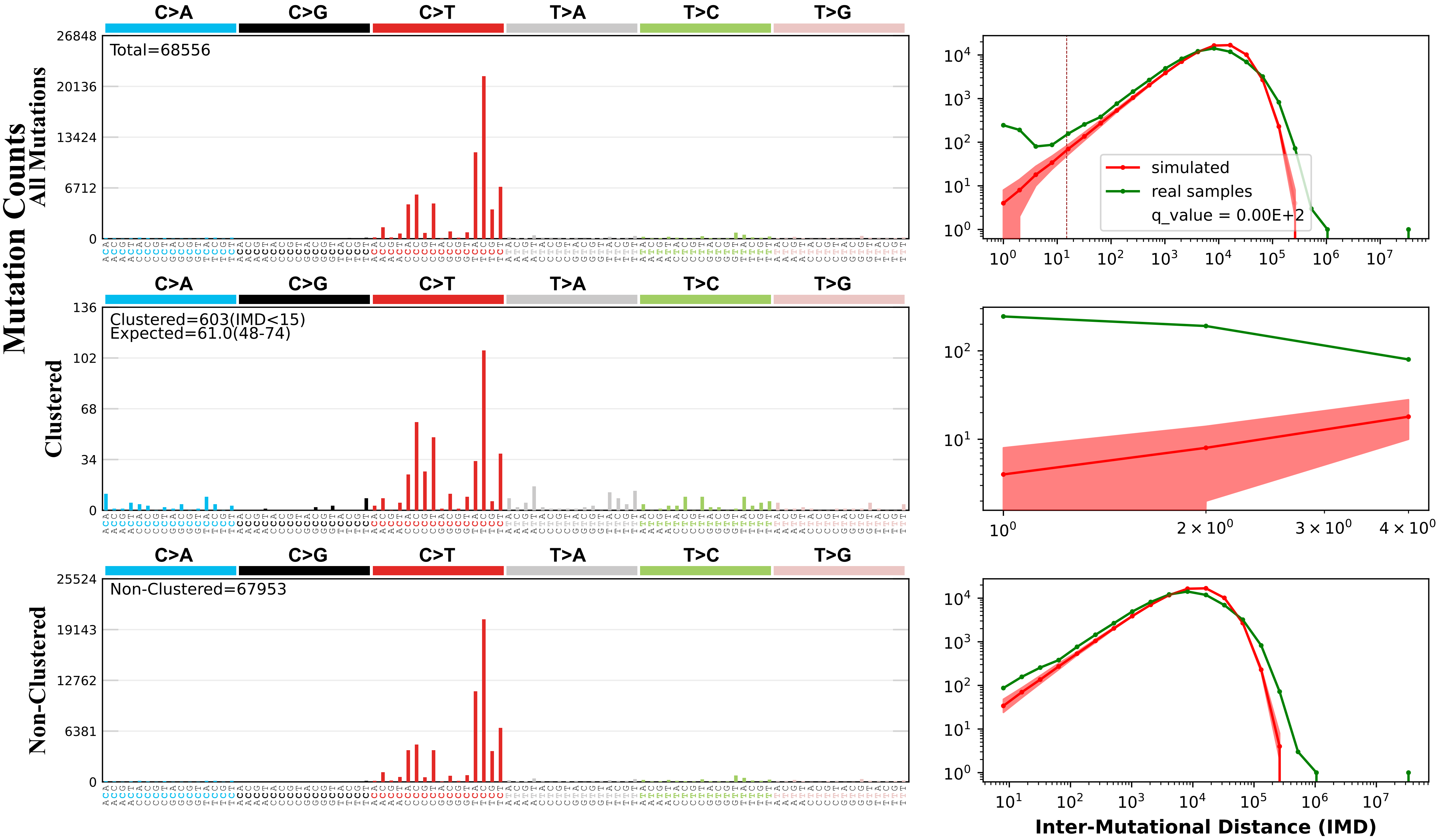

Mutation Counts
All Mutations
Clustered
Non-Clustered
C>A
C>G
C>T
T>A
T>C
T>G
Total=68556
Clustered=603(IMD<15)
Expected=61.0(48-74)
Non-Clustered=67953
26848
20136
13424
6712
0
136
102
68
34
25524
19143
12762
6381
simulated
real samples
q_value = 0.00E+2
Inter-Mutational Distance (IMD)

SP128172

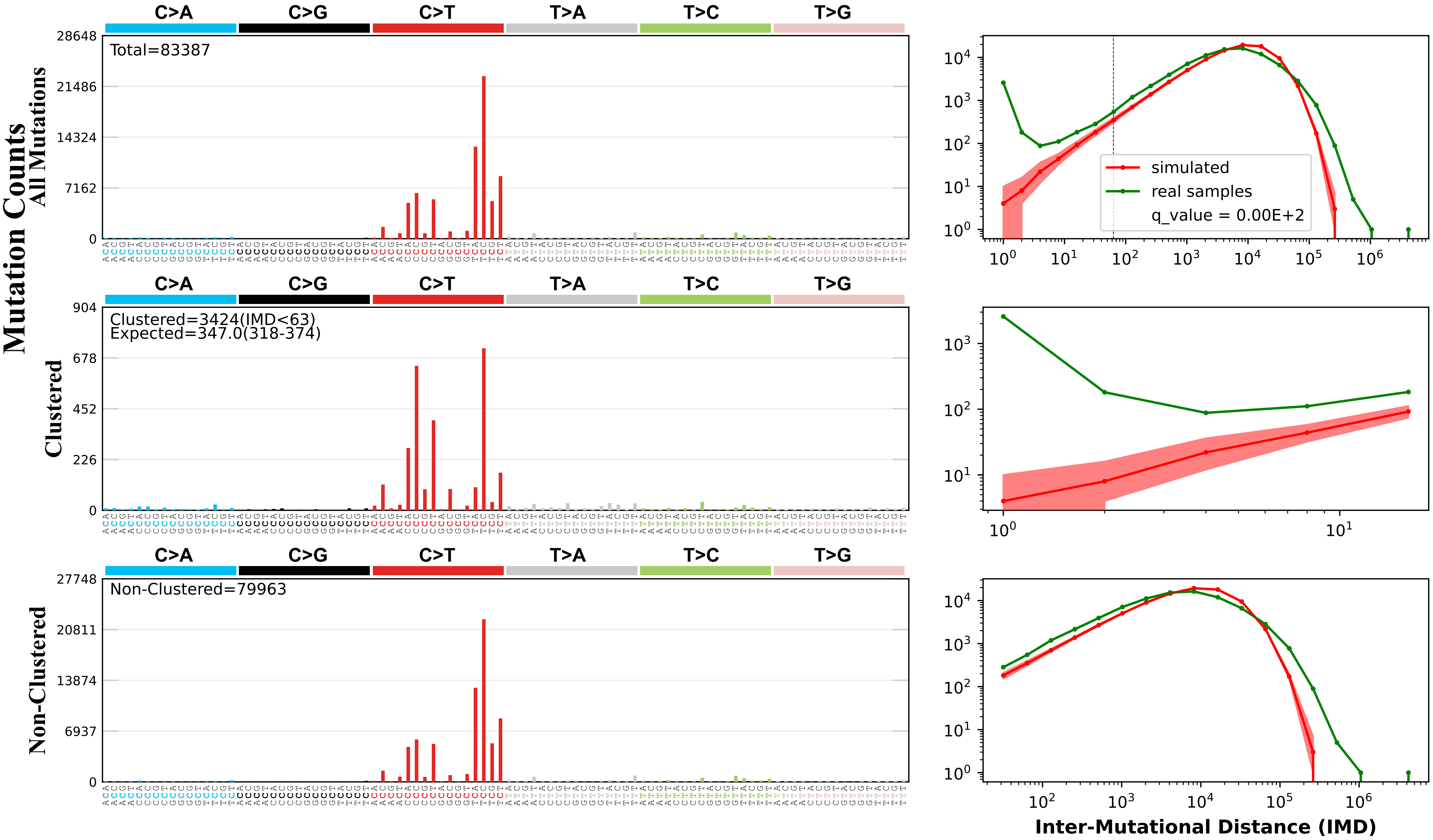

Mutation Counts
All Mutations
Total=83387
Clustered
Clustered=3424(IMD<63)
Expected=347.0(318-374)
Non-Clustered
Non-Clustered=79963
C>A
C>G
C>T
T>A
T>C
T>G
simulated
real samples
q_value = 0.00E+2
Inter-Mutational Distance (IMD)

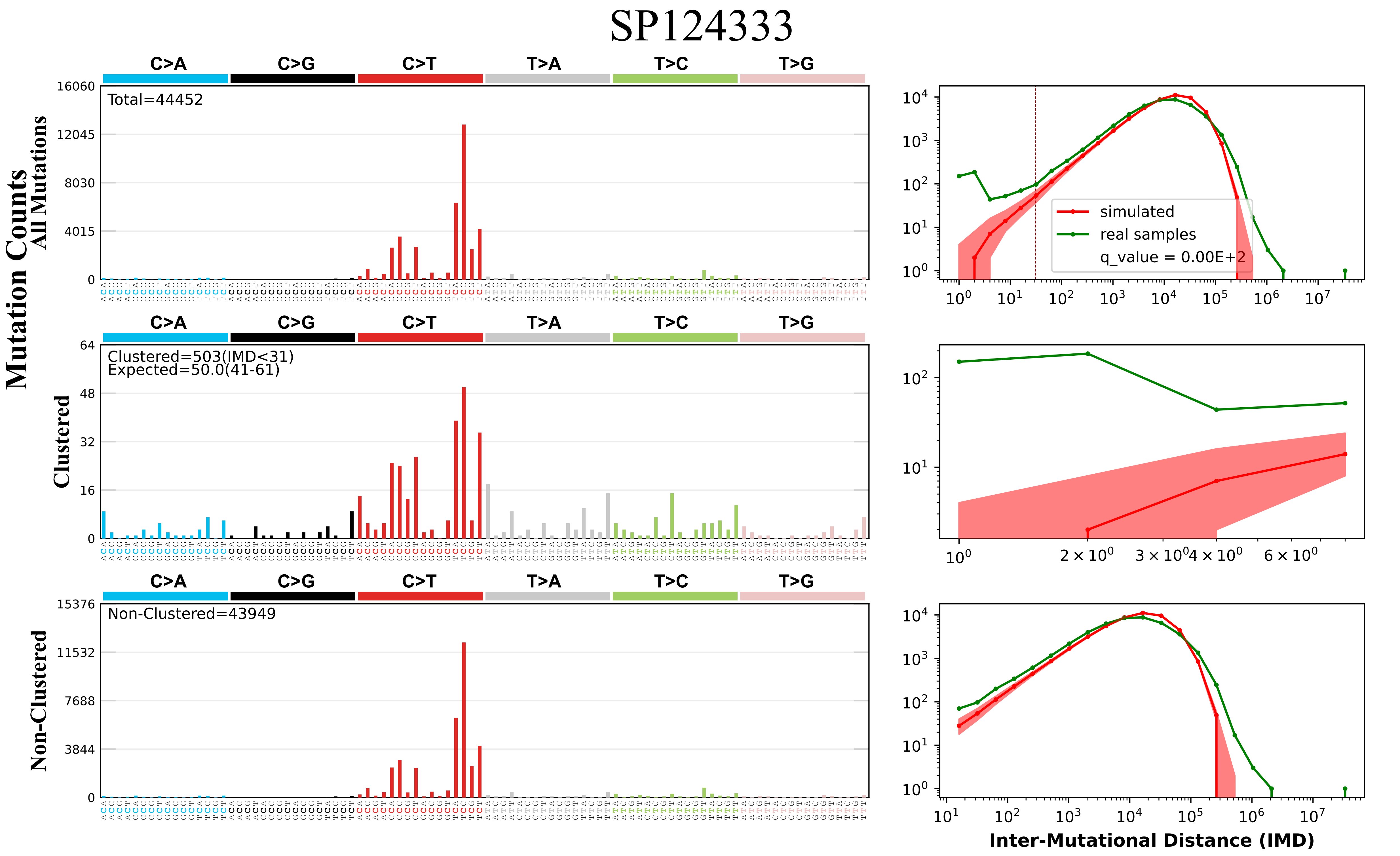

SP124333
Mutation Counts
All Mutations
Total=44452
Clustered
Clustered=503(IMD<31)
Expected=50.0(41-61)
Non-Clustered
Non-Clustered=43949
C>A
C>G
C>T
T>A
T>C
T>G
simulated
real samples
q_value = 0.00E+2
Inter-Mutational Distance (IMD)

SP128672

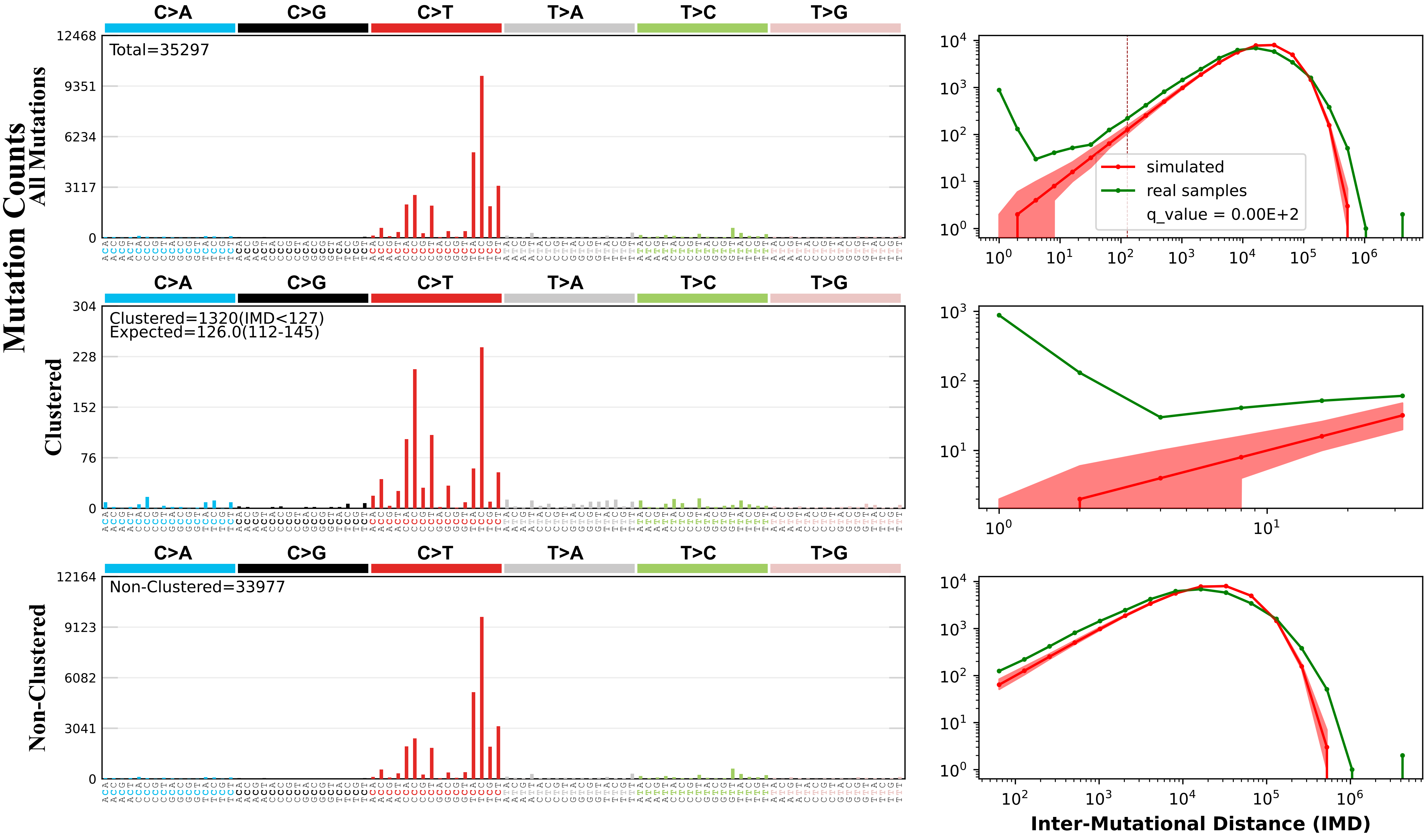

Mutation Counts
All Mutations
Total=35297
Clustered
Clustered=1320(IMD<127)
Expected=126.0(112-145)
Non-Clustered
Non-Clustered=33977
C>A
C>G
C>T
T>A
T>C
T>G
simulated
real samples
q_value = 0.00E+2
Inter-Mutational Distance (IMD)

SP124425

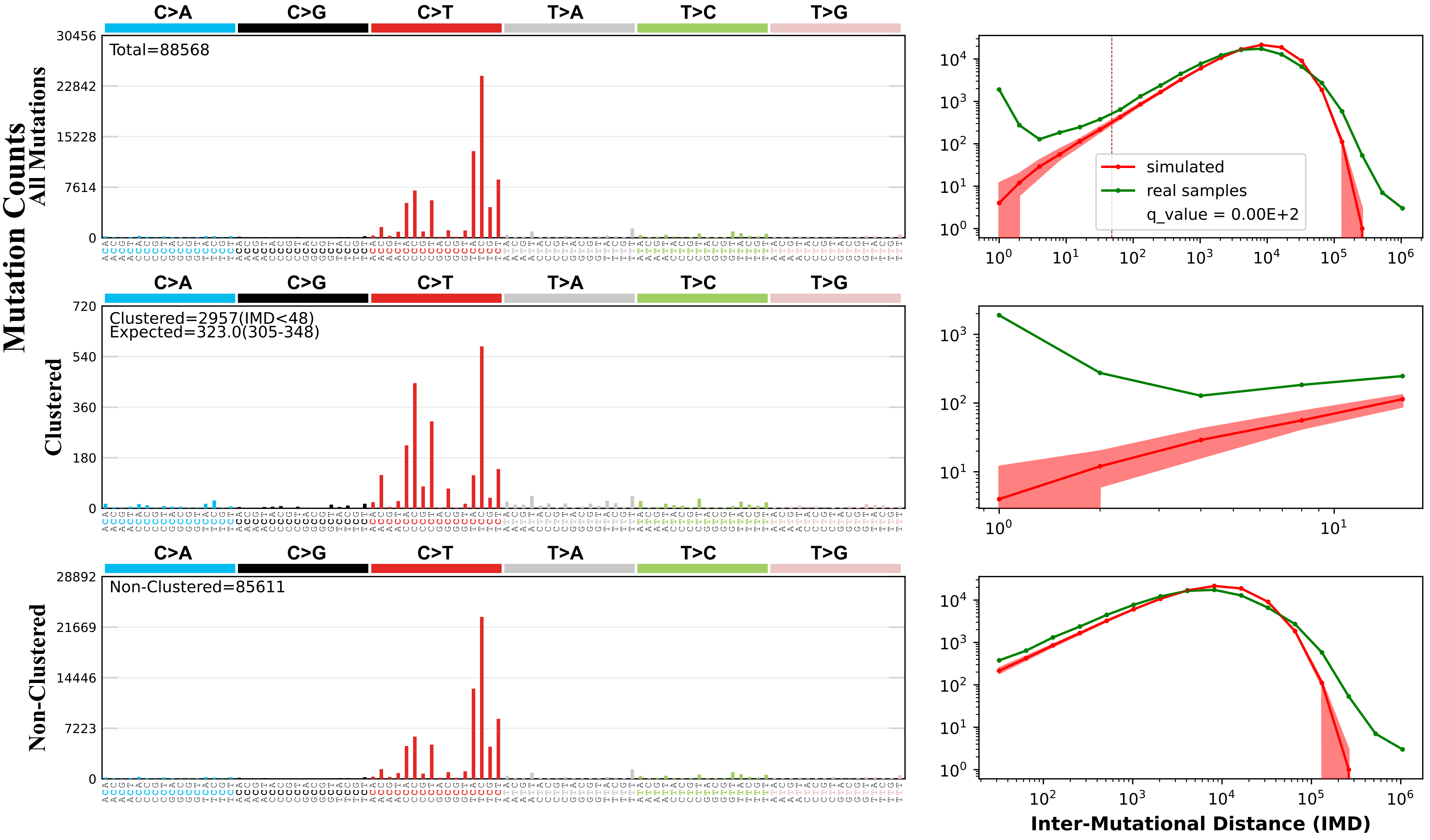
C>A
C>G
C>T
T>A
T>C
T>G
Mutation Counts
All Mutations
Total=88568
30456
22842
15228
7614
0
Clustered
Clustered=2957(IMD<48)
Expected=323.0(305-348)
720
540
360
180
0
Non-Clustered
Non-Clustered=85611
28892
21669
14446
7223
0
simulated
real samples
q_value = 0.00E+2
Inter-Mutational Distance (IMD)

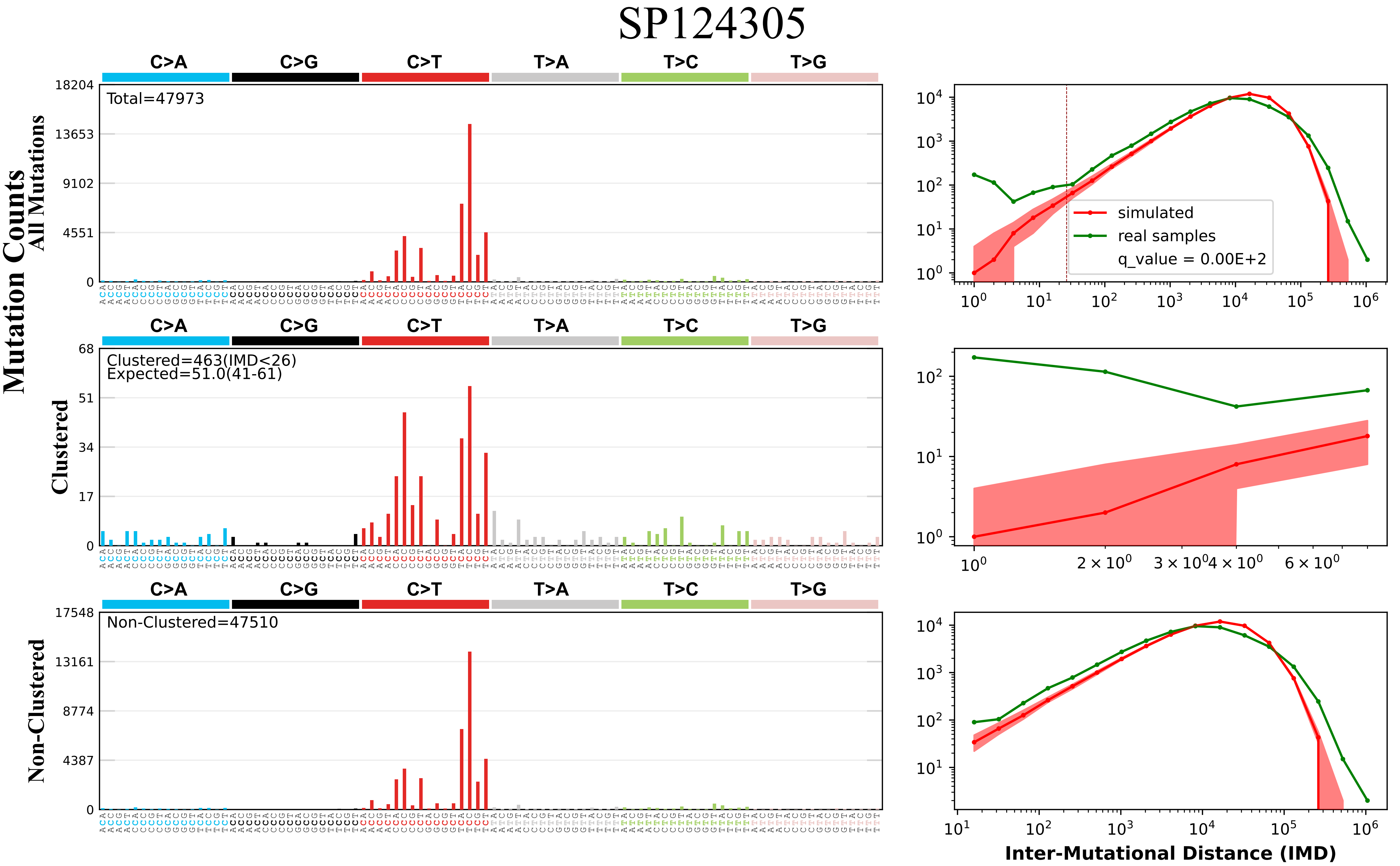

SP124305
Mutation Counts
All Mutations
Total=47973
Clustered
Clustered=463(IMD<26)
Expected=51.0(41-61)
Non-Clustered
Non-Clustered=47510
C>A
C>G
C>T
T>A
T>C
T>G
simulated
real samples
q_value = 0.00E+2
Inter-Mutational Distance (IMD)

SP124412

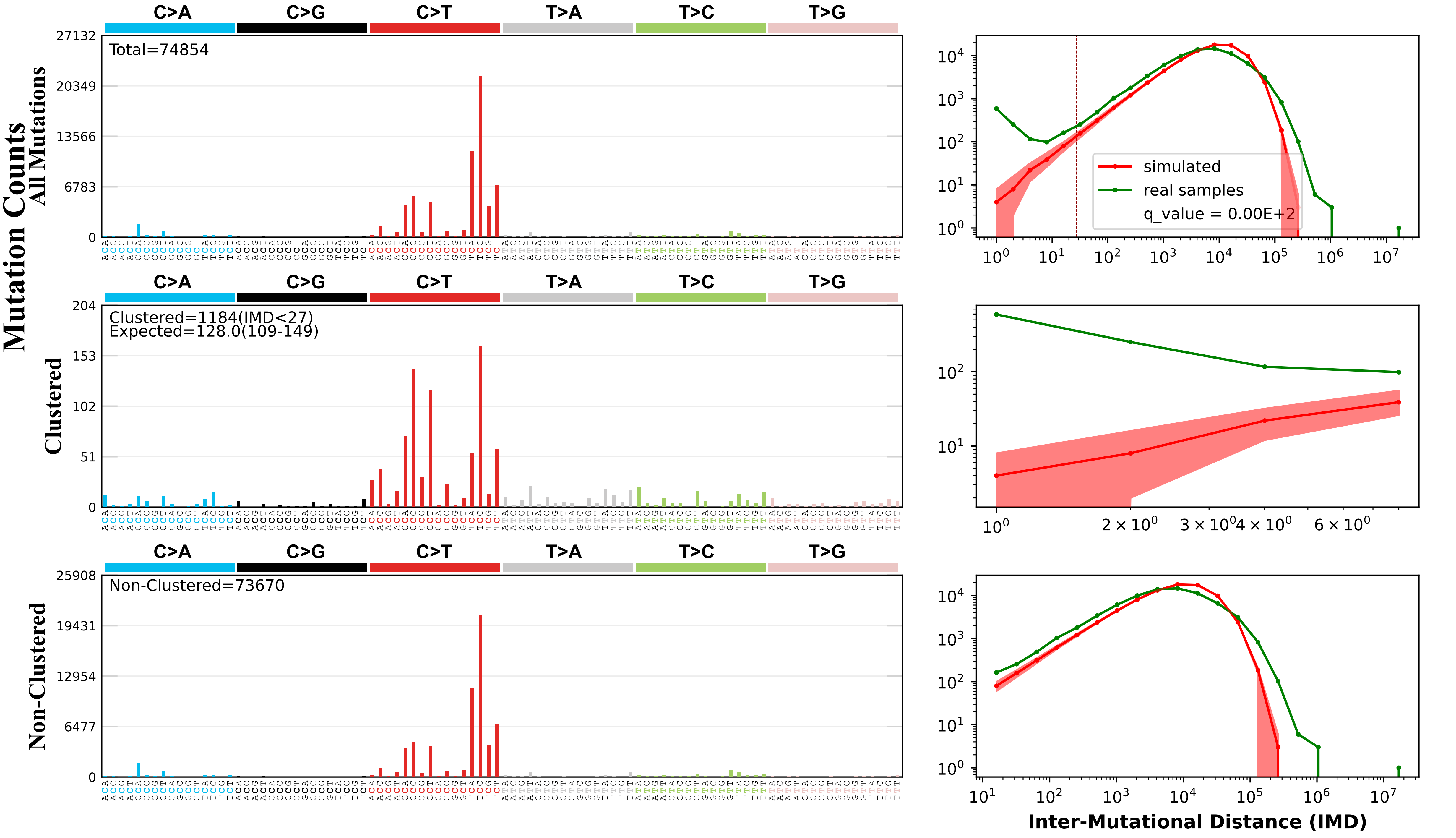

Mutation Counts
All Mutations
Total=74854
Clustered
Clustered=1184(IMD<27)
Expected=128.0(109-149)
Non-Clustered
Non-Clustered=73670
C>A
C>G
C>T
T>A
T>C
T>G
simulated
real samples
q_value = 0.00E+2
Inter-Mutational Distance (IMD)

SP124367

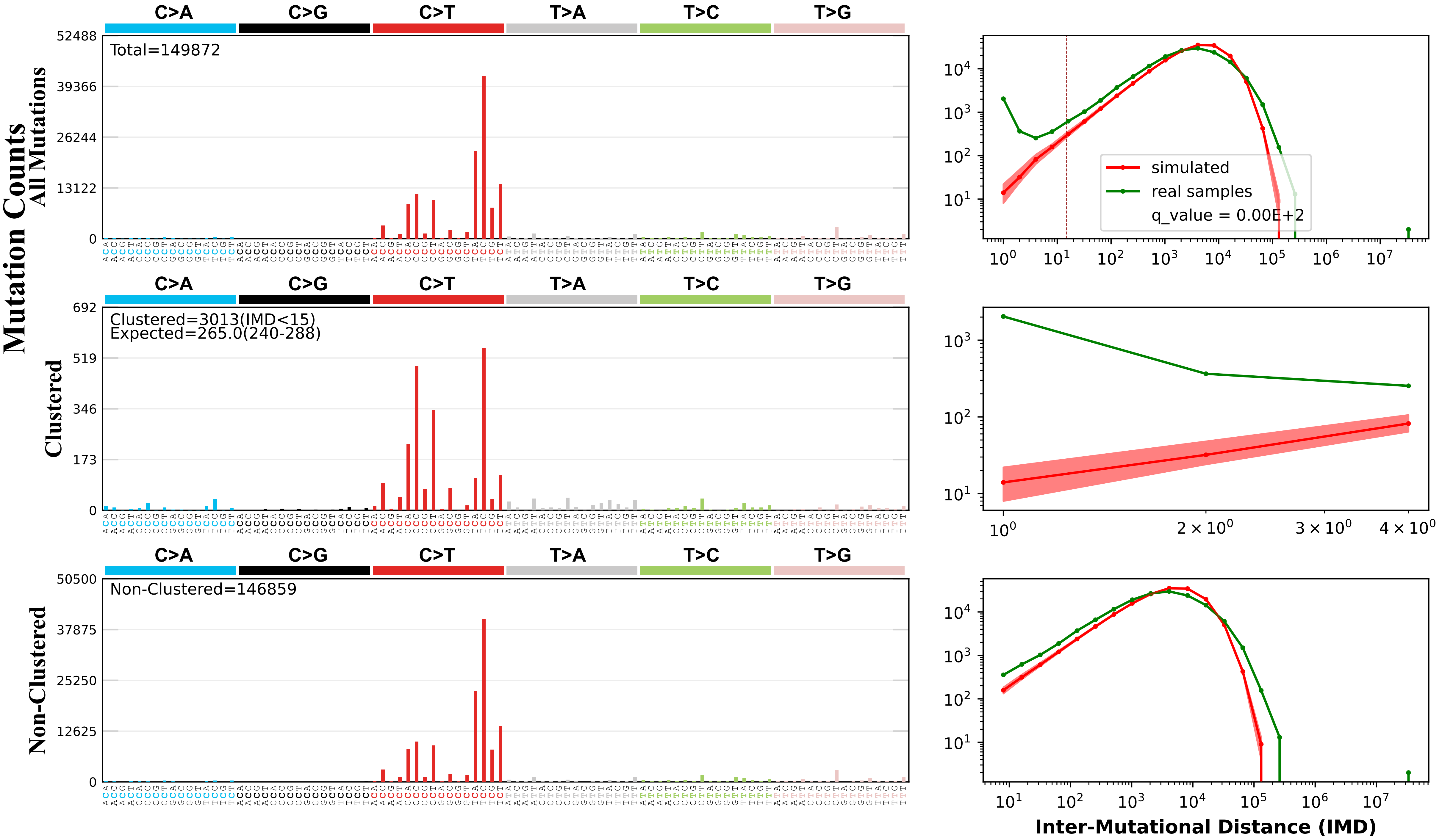

Mutation Counts
All Mutations
Total=149872
Clustered
Clustered=3013(IMD<15)
Expected=265.0(240-288)
Non-Clustered
Non-Clustered=146859
C>A
C>G
C>T
T>A
T>C
T>G
simulated
real samples
q_value = 0.00E+2
Inter-Mutational Distance (IMD)

d0e033bc-8e97-4322-b3b1-8626f1e2449b

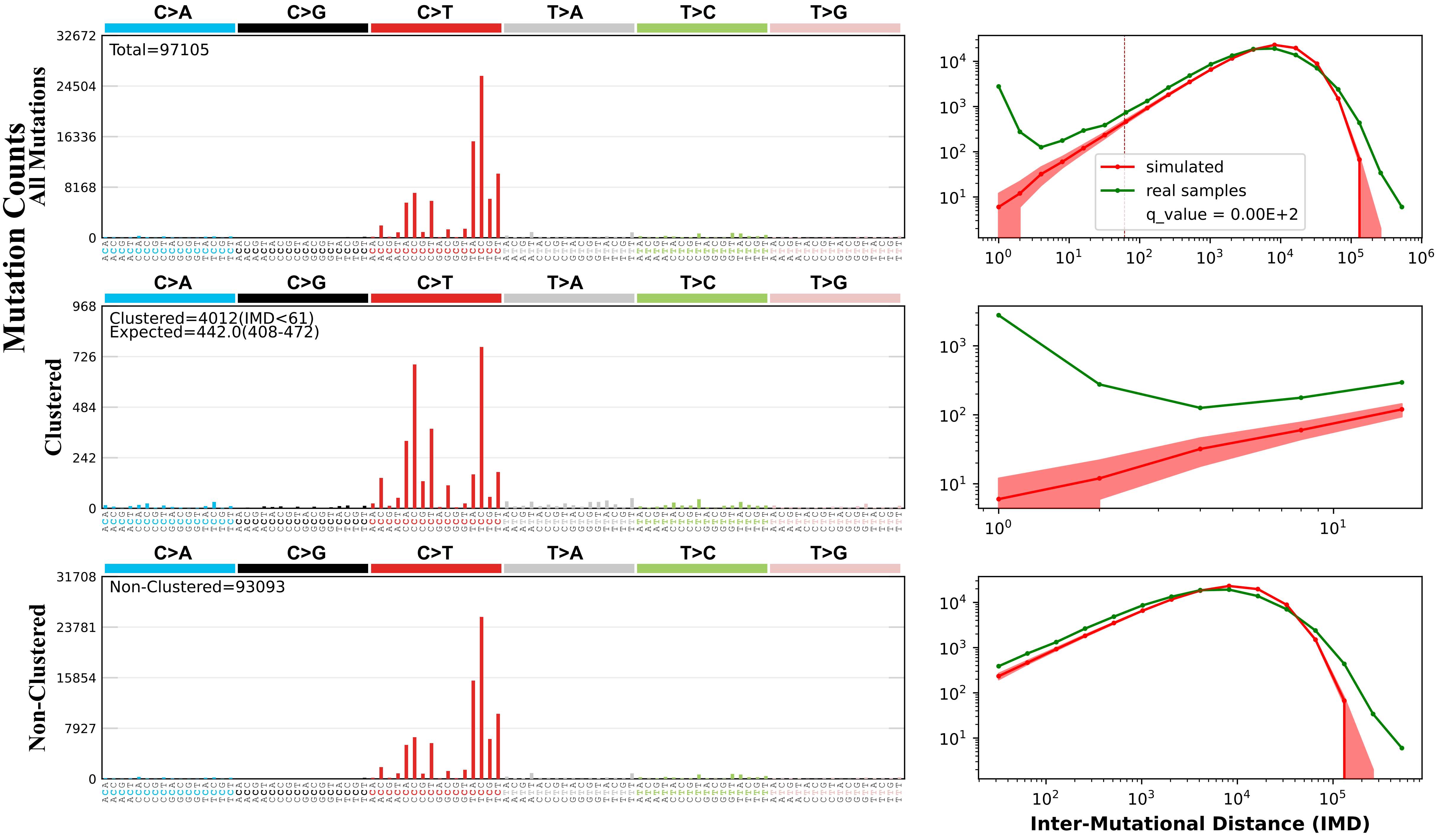

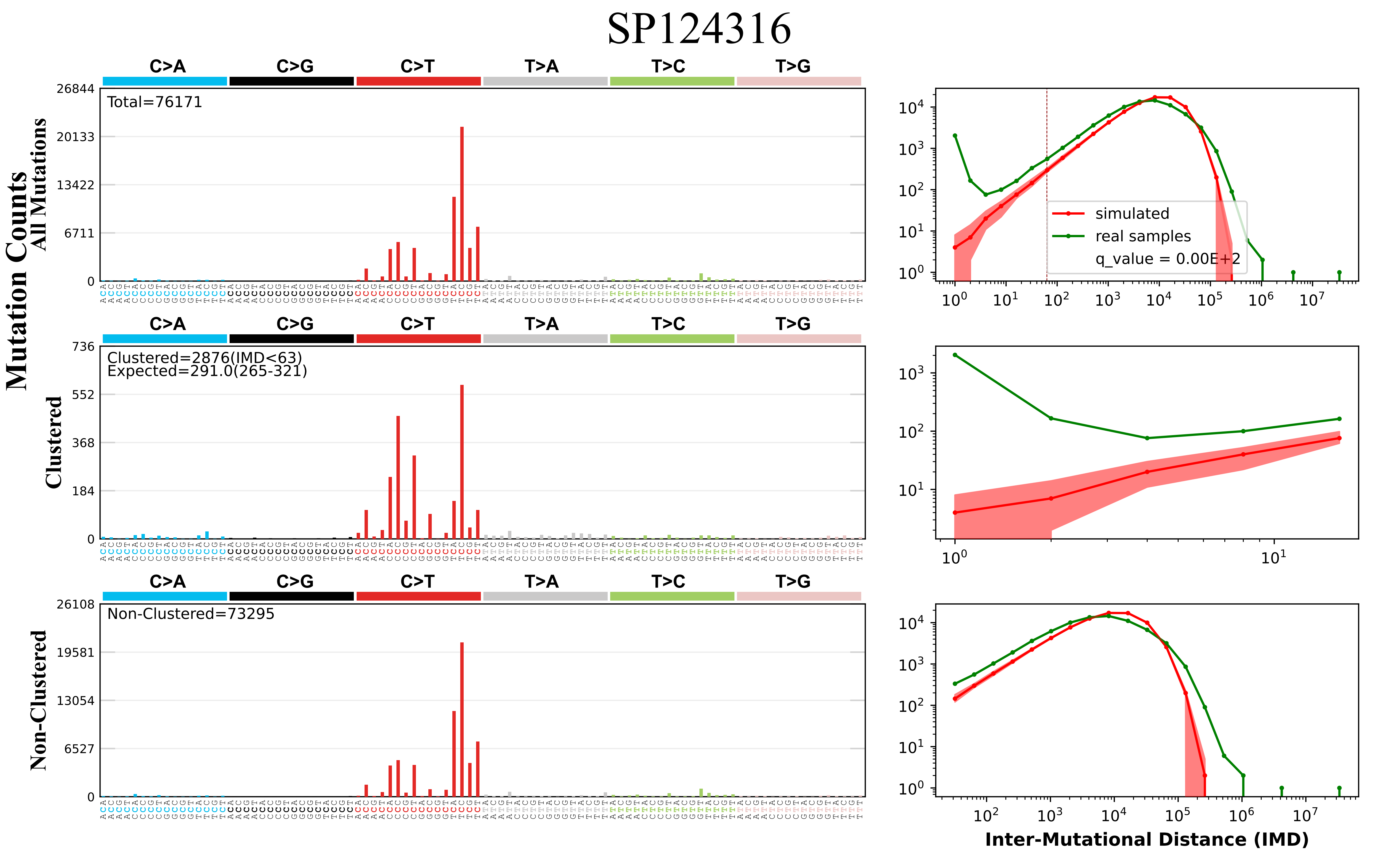
SP124316
Mutation Counts
All Mutations
Total=76171
Clustered
Clustered=2876(IMD<63)
Expected=291.0(265-321)
Non-Clustered
Non-Clustered=73295
C>A
C>G
C>T
T>A
T>C
T>G
simulated
real samples
q_value = 0.00E+2
Inter-Mutational Distance (IMD)

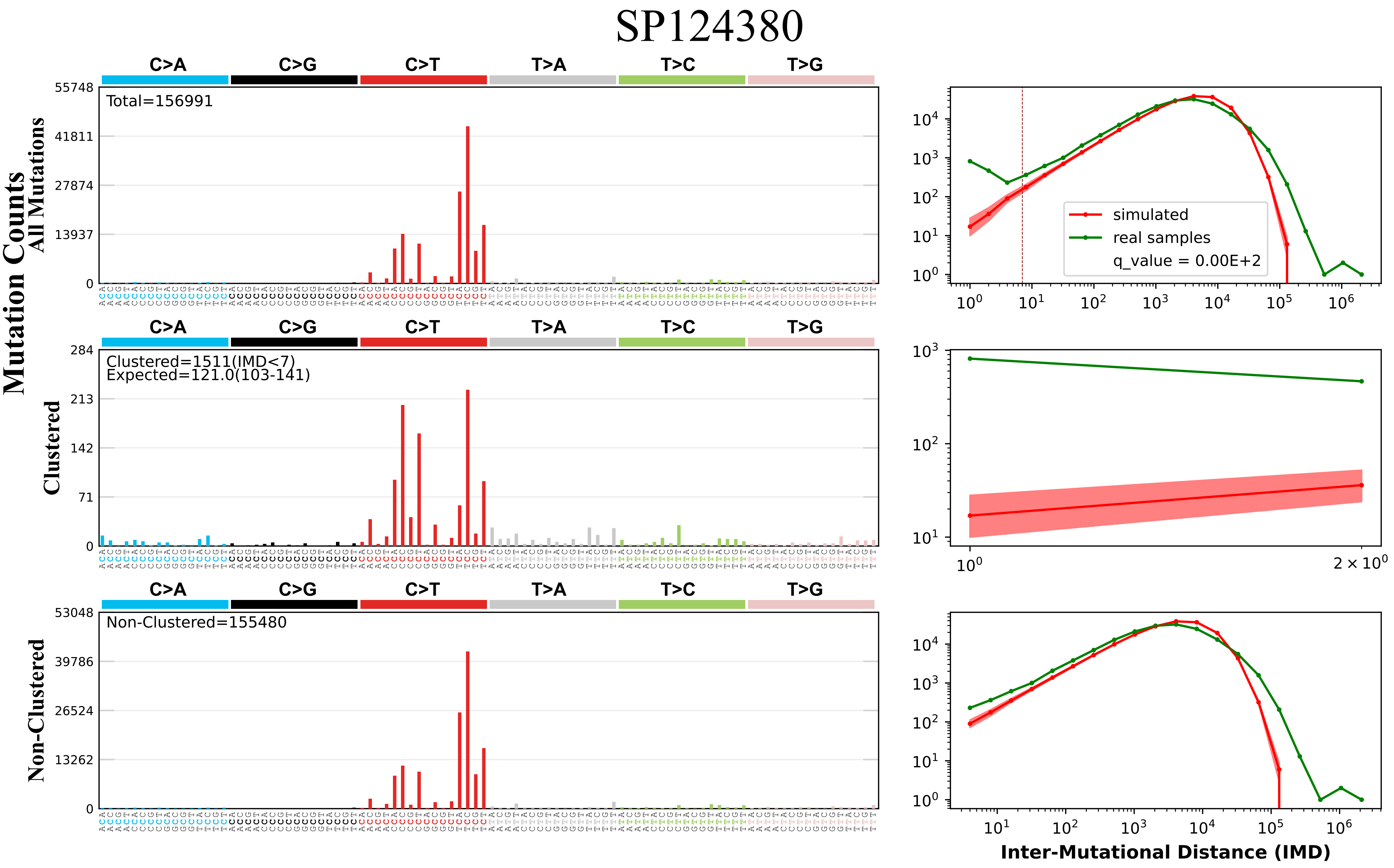

SP124380
Mutation Counts
All Mutations
Total=156991
Clustered
Clustered=1511(IMD<7)
Expected=121.0(103-141)
Non-Clustered
Non-Clustered=155480
C>A
C>G
C>T
T>A
T>C
T>G
simulated
real samples
q_value = 0.00E+2
Inter-Mutational Distance (IMD)

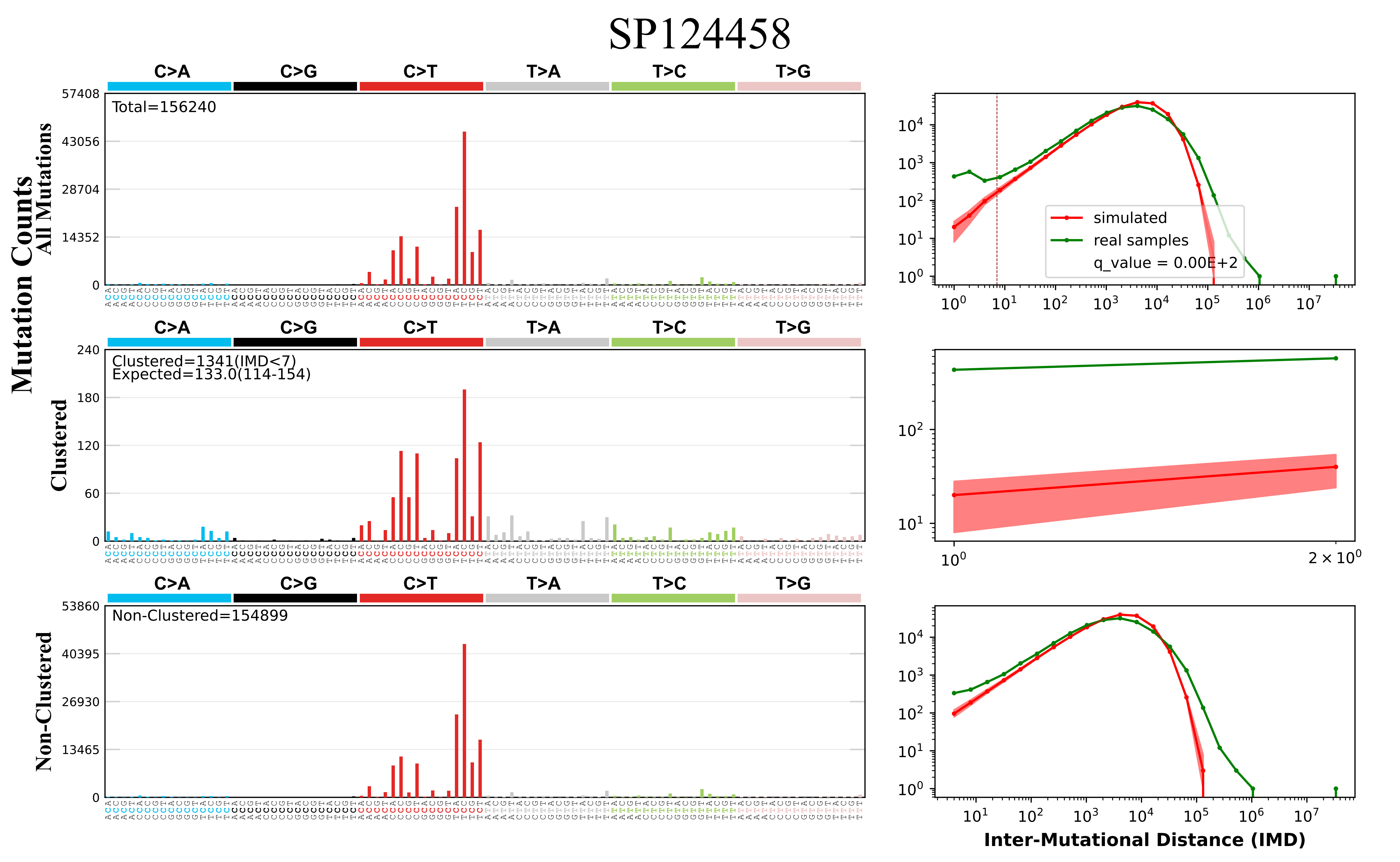
SP124458
C>A
C>G
C>T
T>A
T>C
T>G
Mutation Counts
All Mutations
Total=156240
57408
43056
28704
14352
0
Clustered
Clustered=1341(IMD<7)
Expected=133.0(114-154)
240
180
120
60
0
Non-Clustered
Non-Clustered=154899
53860
40395
26930
13465
0
simulated
real samples
q_value = 0.00E+2
Inter-Mutational Distance (IMD)

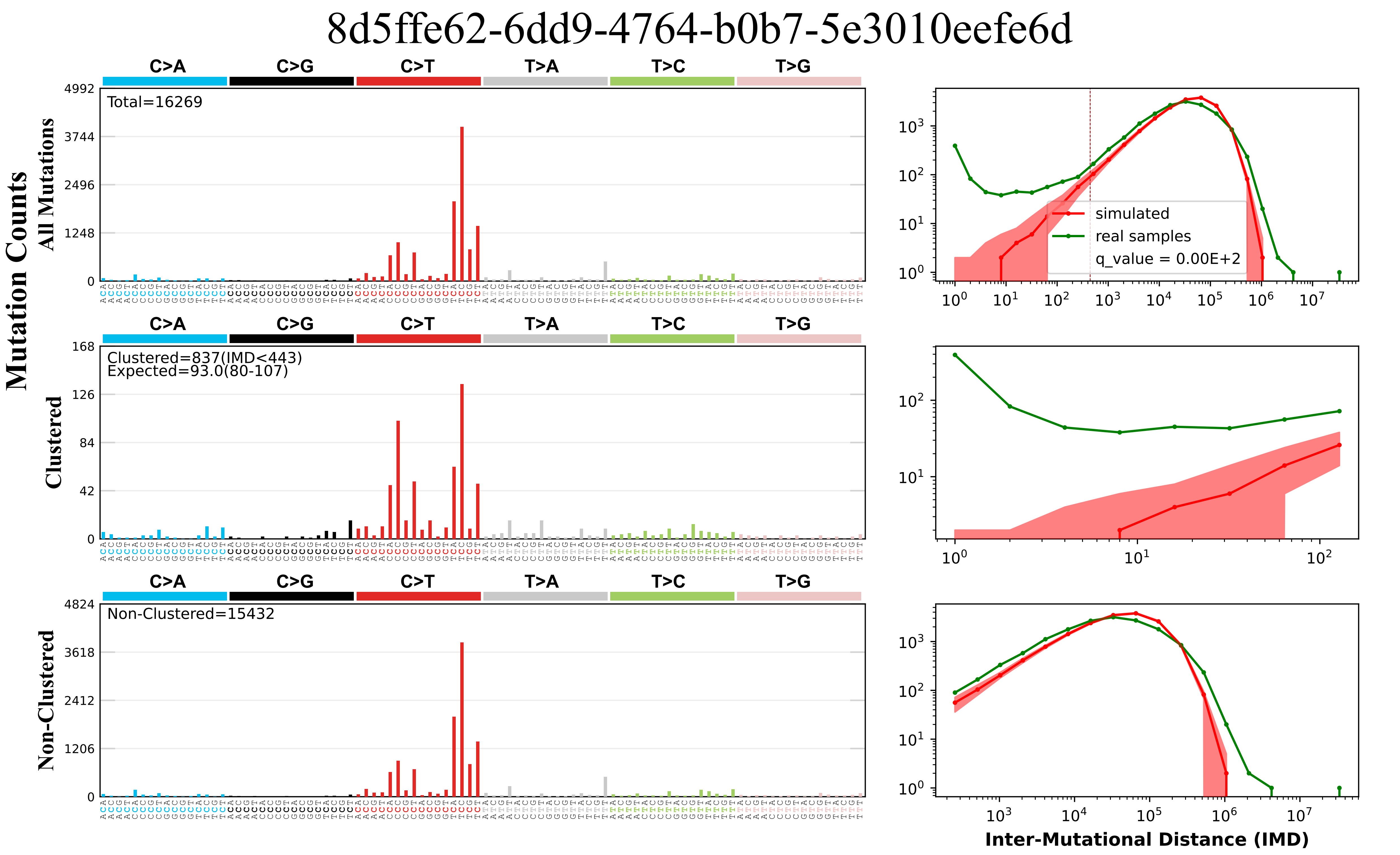
8d5ffe62-6dd9-4764-b0b7-5e3010eefe6d
Mutation Counts
All Mutations
Total=16269
Clustered
Clustered=837(IMD<443)
Expected=93.0(80-107)
Non-Clustered
Non-Clustered=15432
C>A
C>G
C>T
T>A
T>C
T>G
simulated
real samples
q_value = 0.00E+2
Inter-Mutational Distance (IMD)

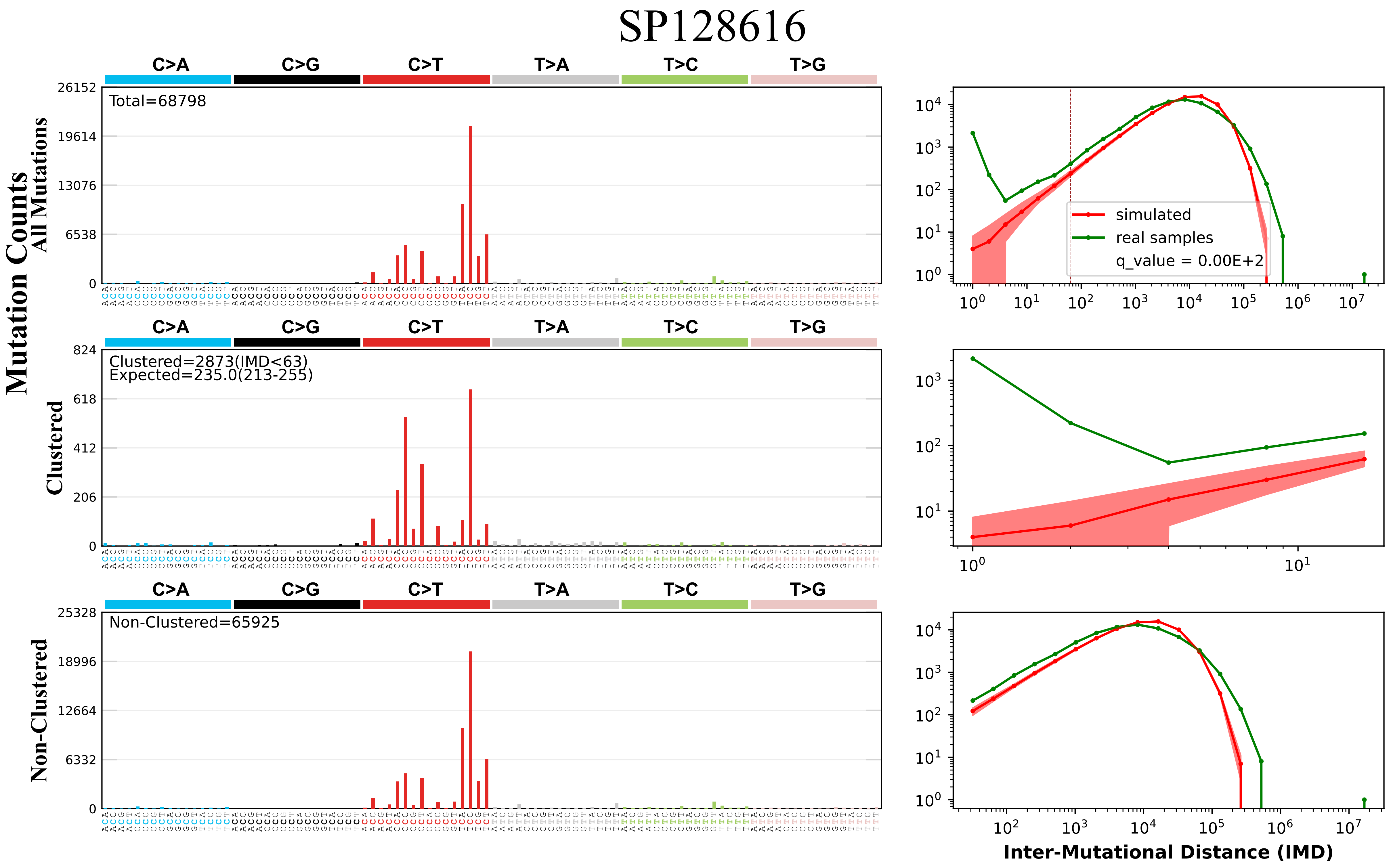
SP128616
Mutation Counts
All Mutations
Clustered
Non-Clustered
C>A
C>G
C>T
T>A
T>C
T>G
Total=68798
Clustered=2873(IMD<63)
Expected=235.0(213-255)
Non-Clustered=65925
26152
19614
13076
6538
824
618
412
206
25328
18996
12664
6332
0
simulated
real samples
q_value = 0.00E+2
Inter-Mutational Distance (IMD)

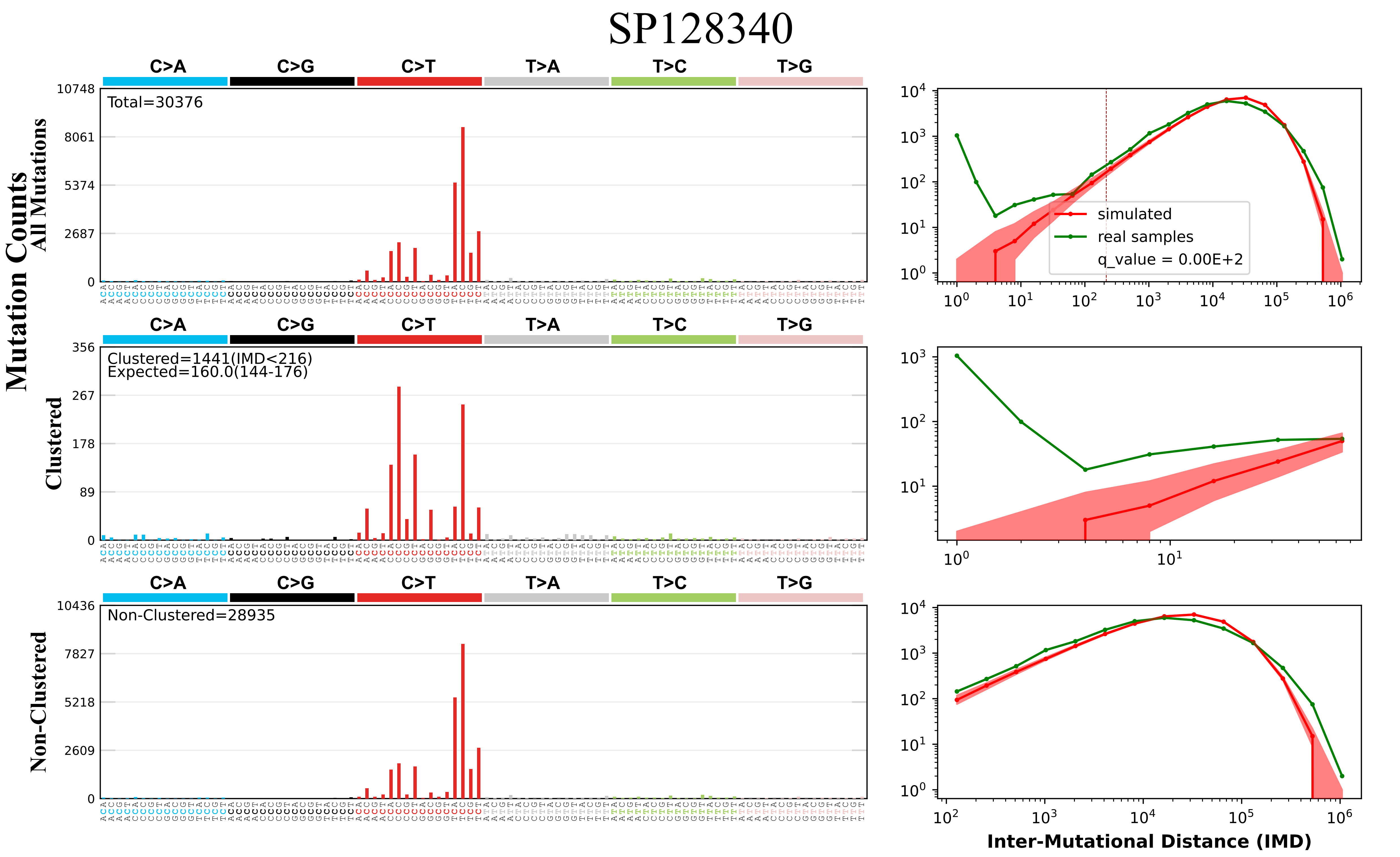

SP128340
Mutation Counts
All Mutations
Clustered
Non-Clustered
C>A
C>G
C>T
T>A
T>C
T>G
Total=30376
Clustered=1441(IMD<216)
Expected=160.0(144-176)
Non-Clustered=28935
simulated
real samples
q_value = 0.00E+2
Inter-Mutational Distance (IMD)

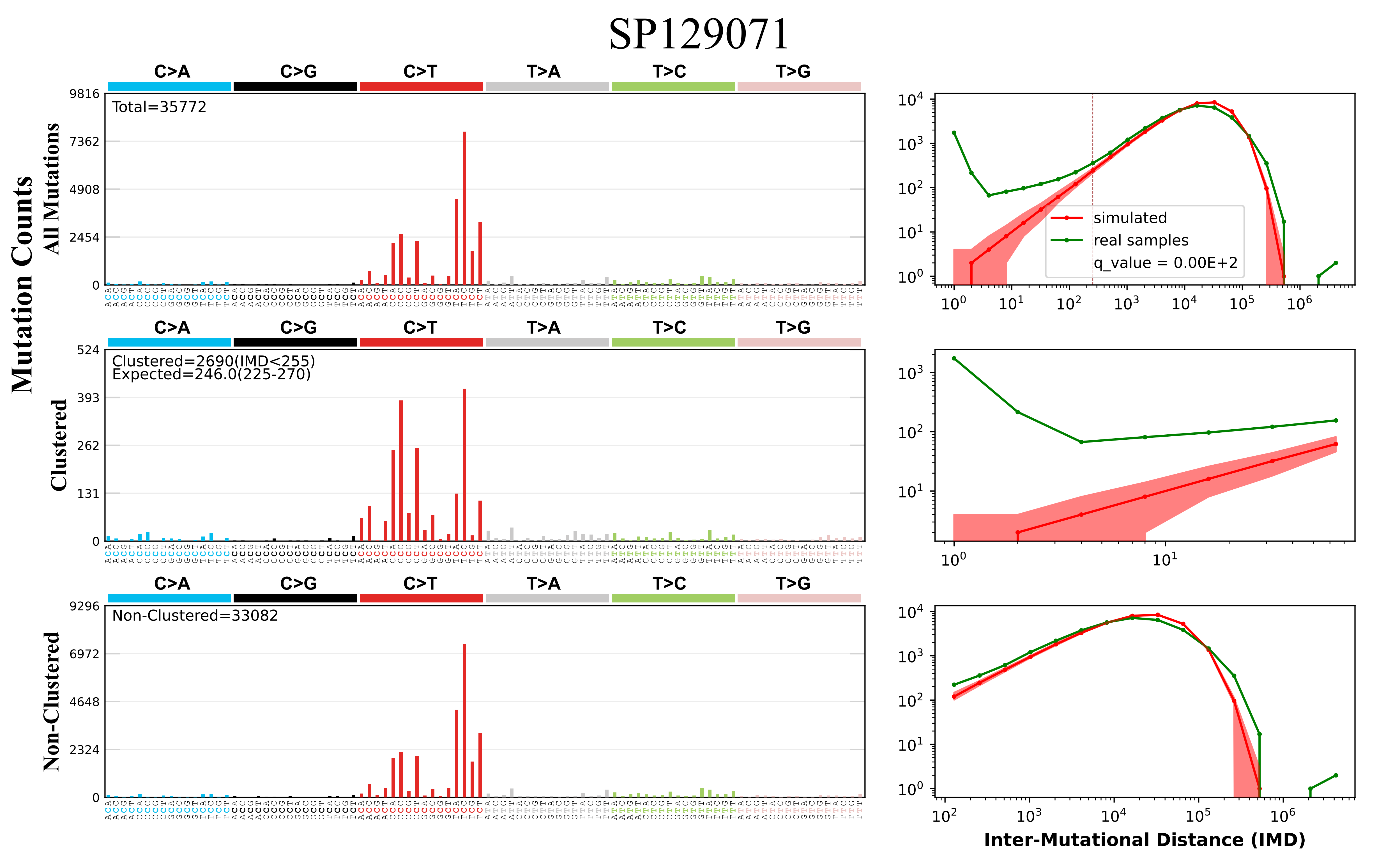

SP129071
Mutation Counts
All Mutations
Clustered
Non-Clustered
C>A
C>G
C>T
T>A
T>C
T>G
Total=35772
Clustered=2690(IMD<255)
Expected=246.0(225-270)
Non-Clustered=33082
simulated
real samples
q_value = 0.00E+2
Inter-Mutational Distance (IMD)

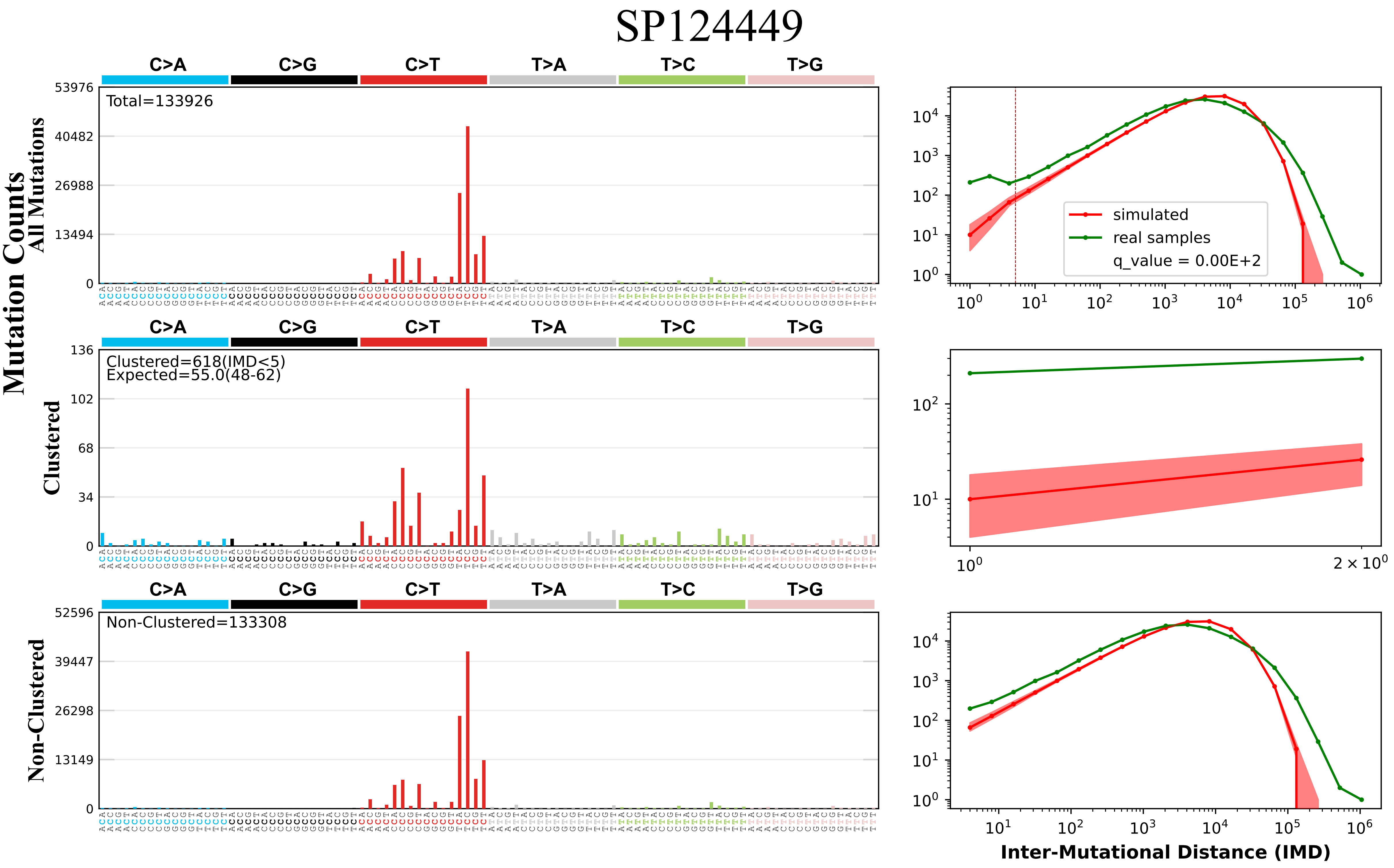
SP124449
C>A
C>G
C>T
T>A
T>C
T>G
Mutation Counts
All Mutations
Total=133926
53976
40482
26988
13494
0
Clustered
Clustered=618(IMD<5)
Expected=55.0(48-62)
136
102
68
34
0
Non-Clustered
Non-Clustered=133308
52596
39447
26298
13149
0
simulated
real samples
q_value = 0.00E+2
Inter-Mutational Distance (IMD)

15daadb0-6b24-4248-9225-470dc08f1552

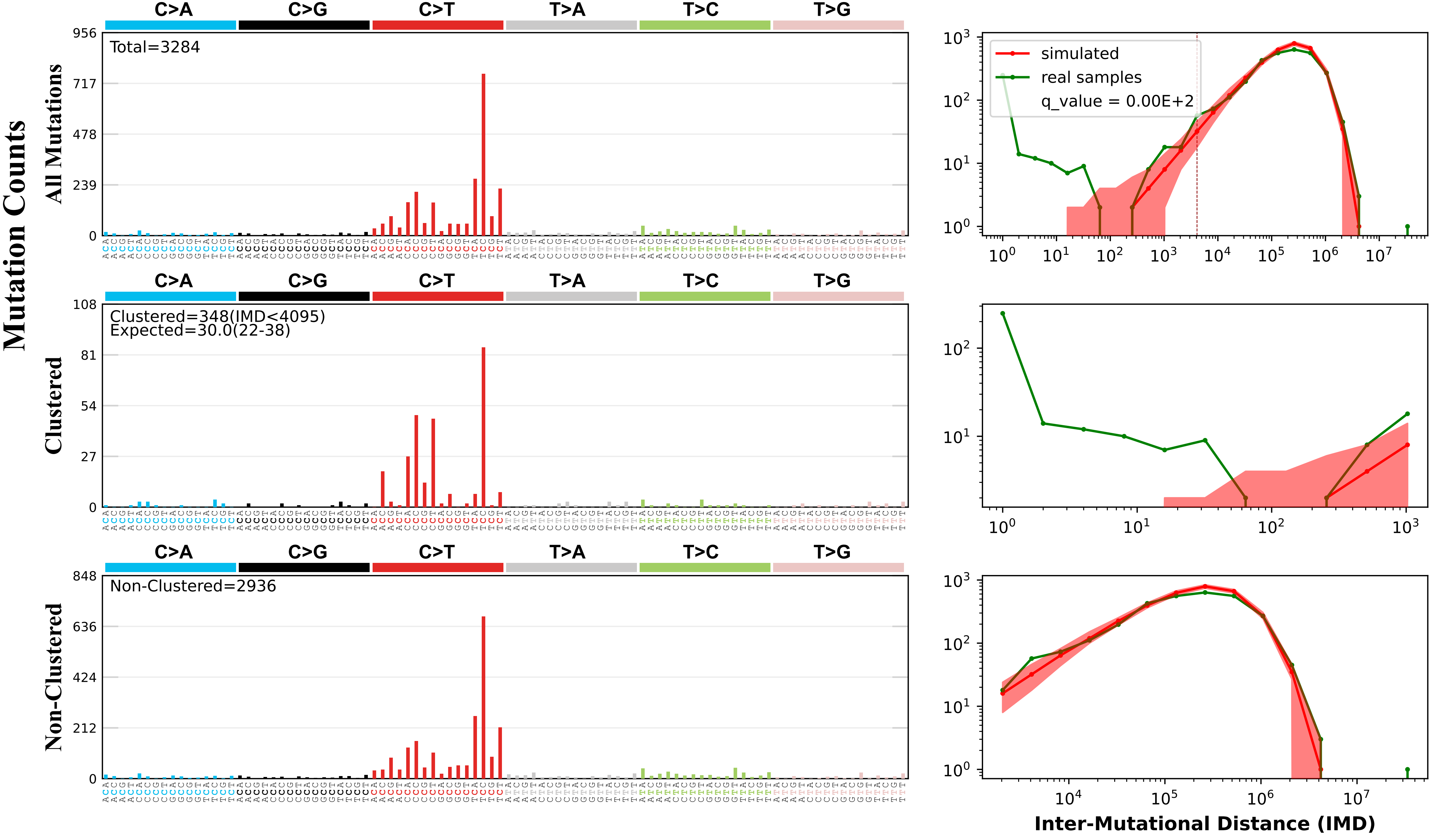

SP128705

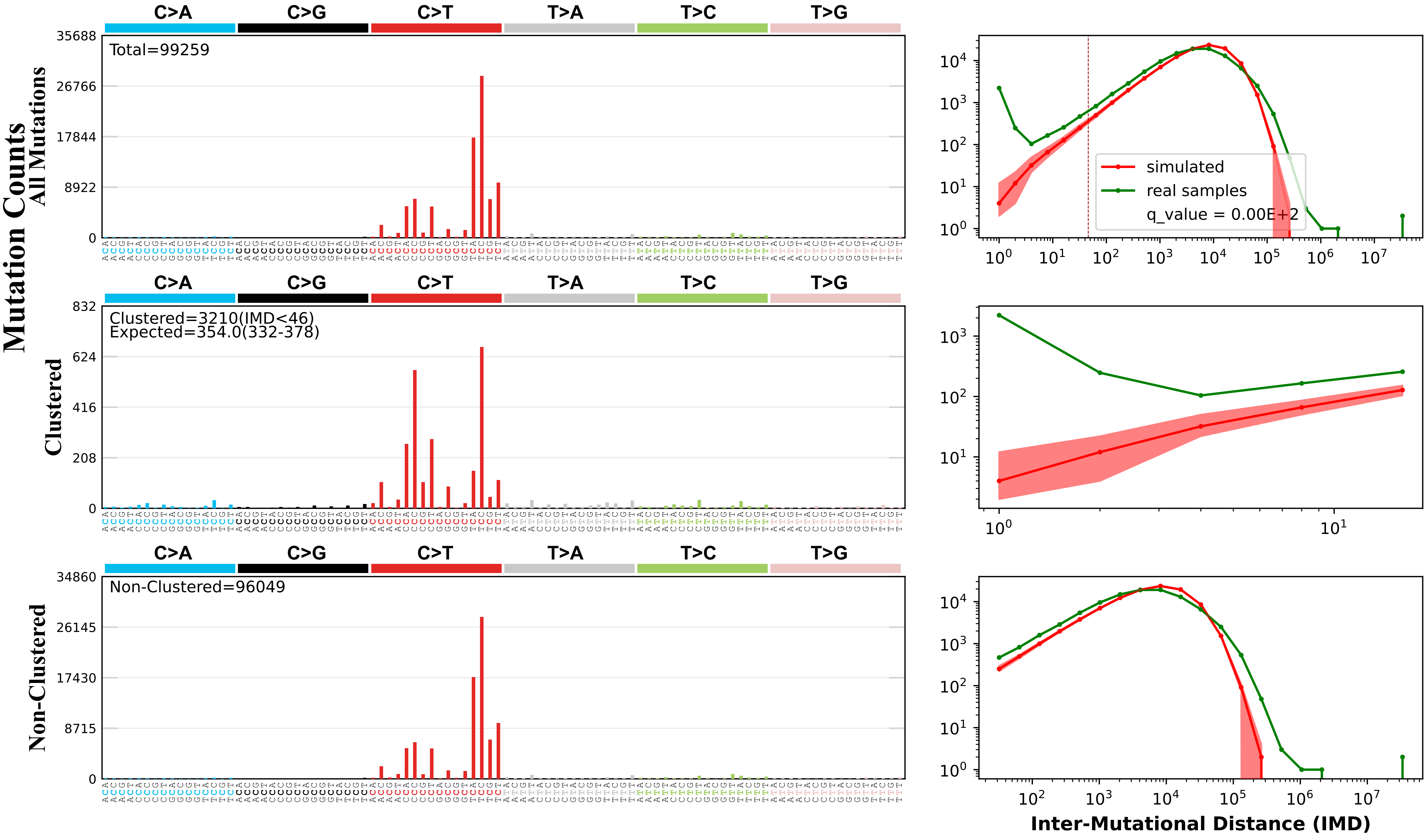
C>A
C>G
C>T
T>A
T>C
T>G
Mutation Counts
All Mutations
Total=99259
35688
26766
17844
8922
0
simulated
real samples
q_value = 0.00E+2
Clustered
Clustered=3210(IMD<46)
Expected=354.0(332-378)
832
624
416
208
Non-Clustered
Non-Clustered=96049
34860
26145
17430
8715
Inter-Mutational Distance (IMD)

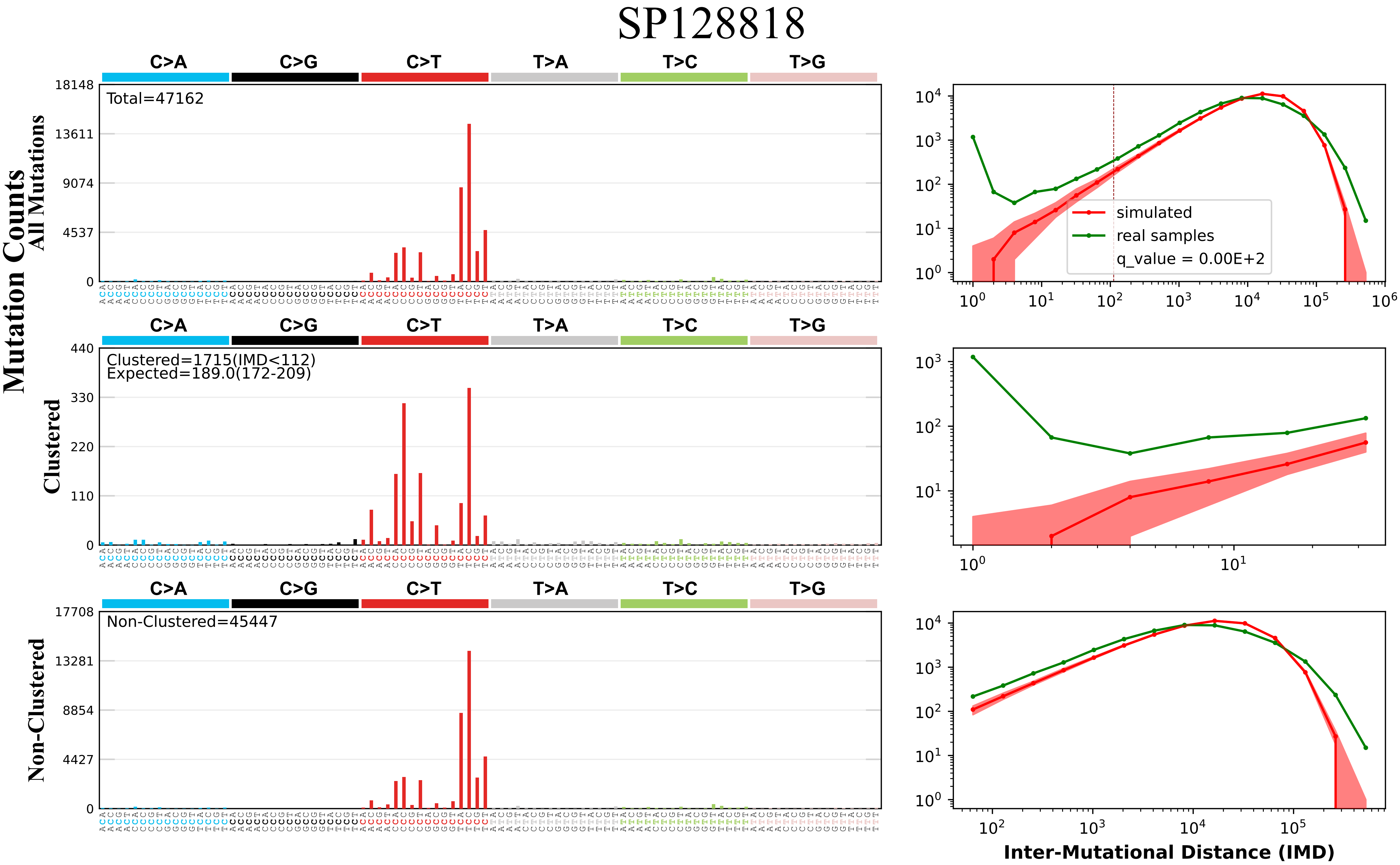

SP128818
C>A
C>G
C>T
T>A
T>C
T>G
Mutation Counts
All Mutations
Total=47162
18148
13611
9074
4537
0
Clustered
Clustered=1715(IMD<112)
Expected=189.0(172-209)
440
330
220
110
0
Non-Clustered
Non-Clustered=45447
17708
13281
8854
4427
0
simulated
real samples
q_value = 0.00E+2
Inter-Mutational Distance (IMD)

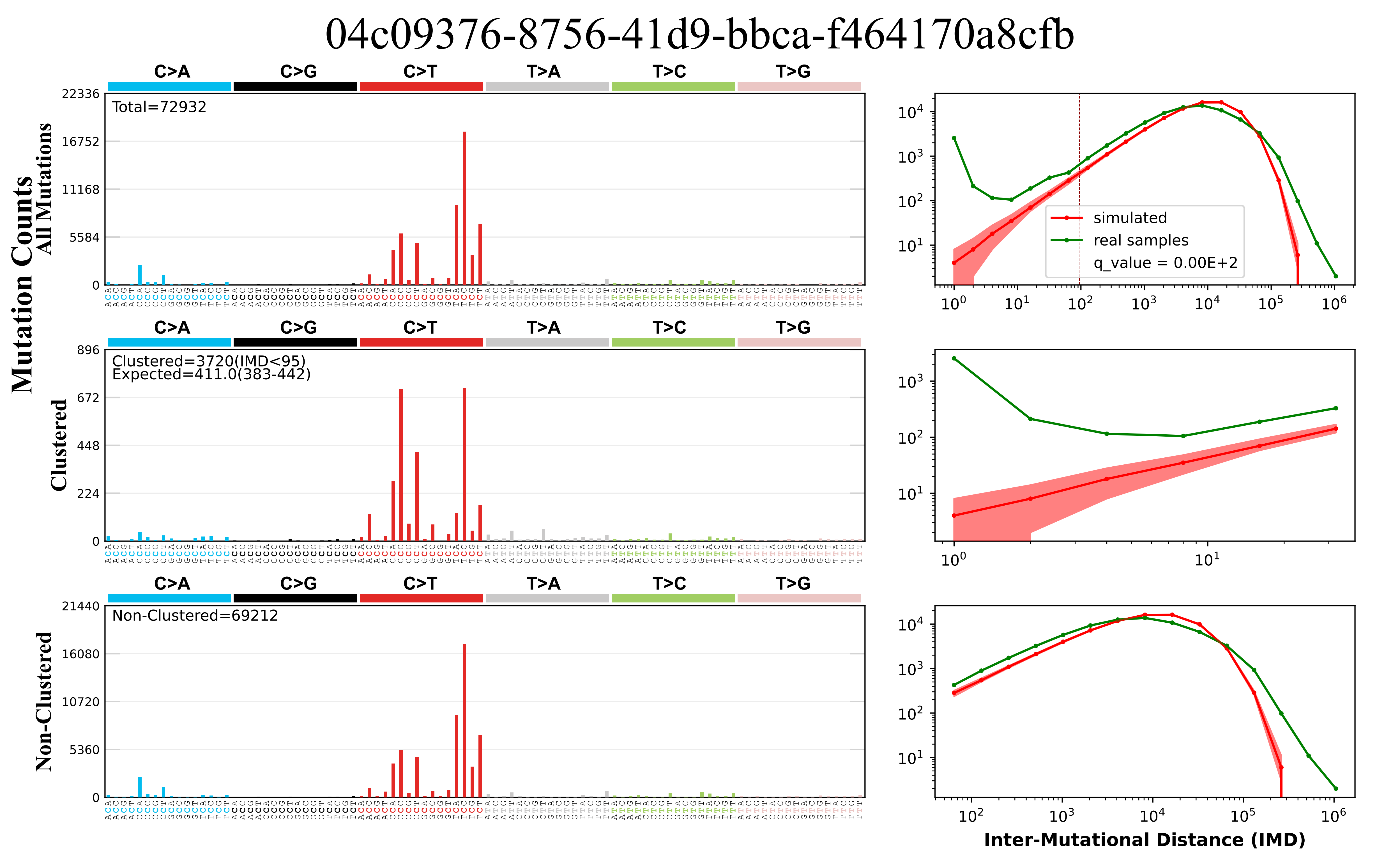
04c09376-8756-41d9-bbca-f464170a8cfb
Mutation Counts
All Mutations
Total=72932
Clustered
Clustered=3720(IMD<95)
Expected=411.0(383-442)
Non-Clustered
Non-Clustered=69212
C>A
C>G
C>T
T>A
T>C
T>G
simulated
real samples
q_value = 0.00E+2
Inter-Mutational Distance (IMD)

507620ef-f0fc-4ac3-a7c0-ea2e36c40f77

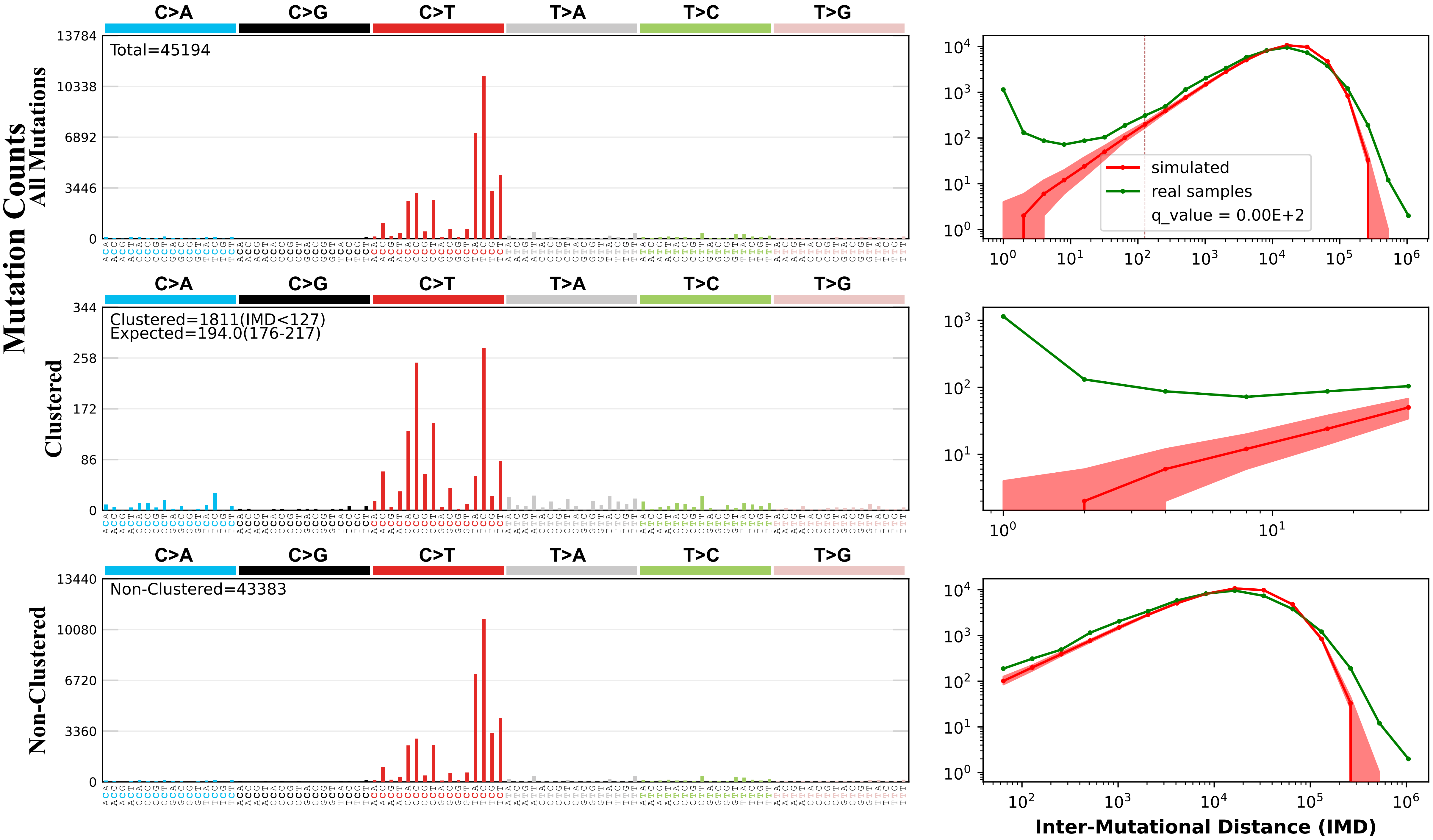

SP128094

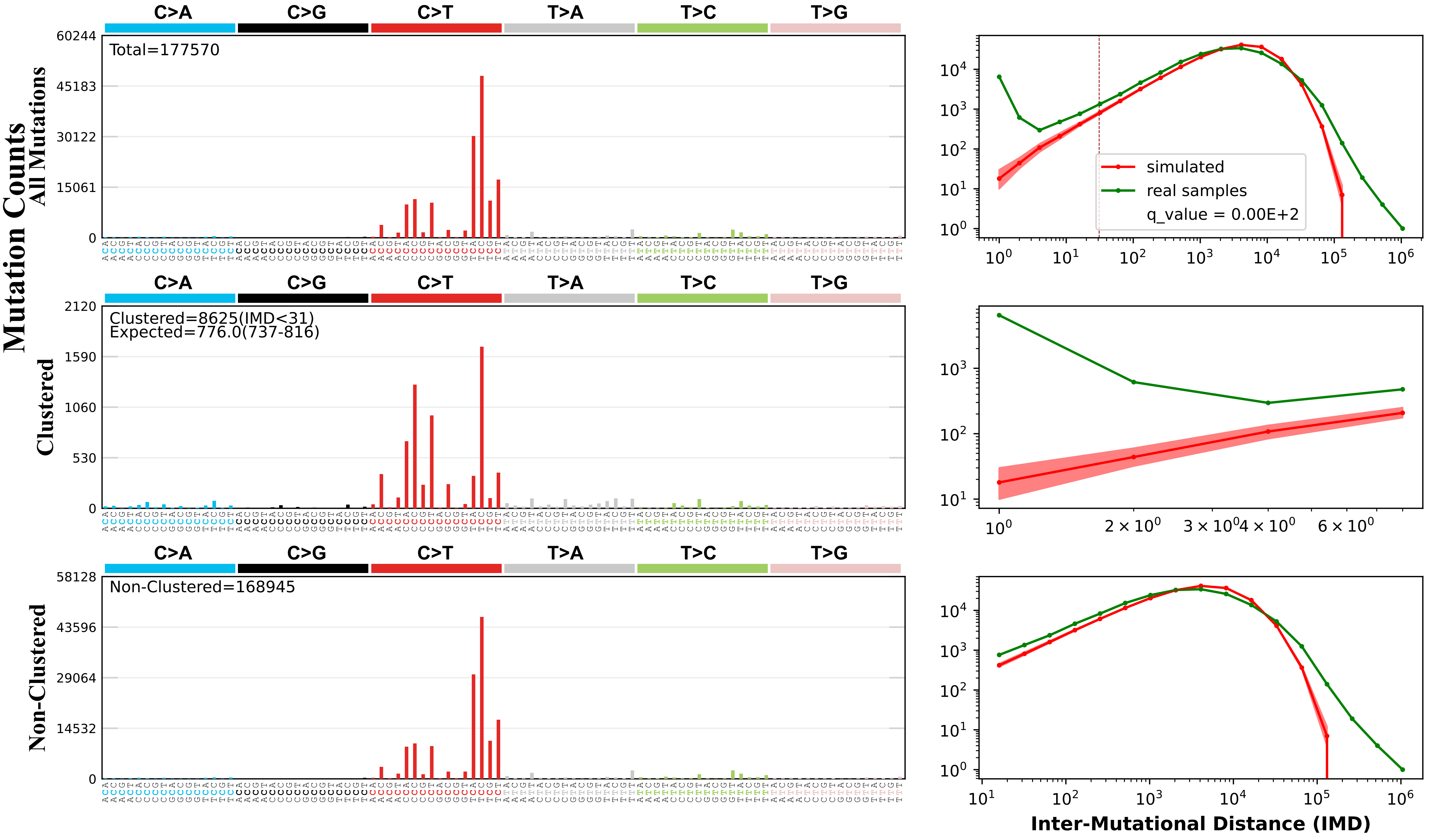

Mutation Counts
All Mutations
Clustered
Non-Clustered
C>A
C>G
C>T
T>A
T>C
T>G
Total=177570
Clustered=8625(IMD<31)
Expected=776.0(737-816)
Non-Clustered=168945
60244
45183
30122
15061
0
2120
1590
1060
530
0
58128
43596
29064
14532
0
simulated
real samples
q_value = 0.00E+2
Inter-Mutational Distance (IMD)

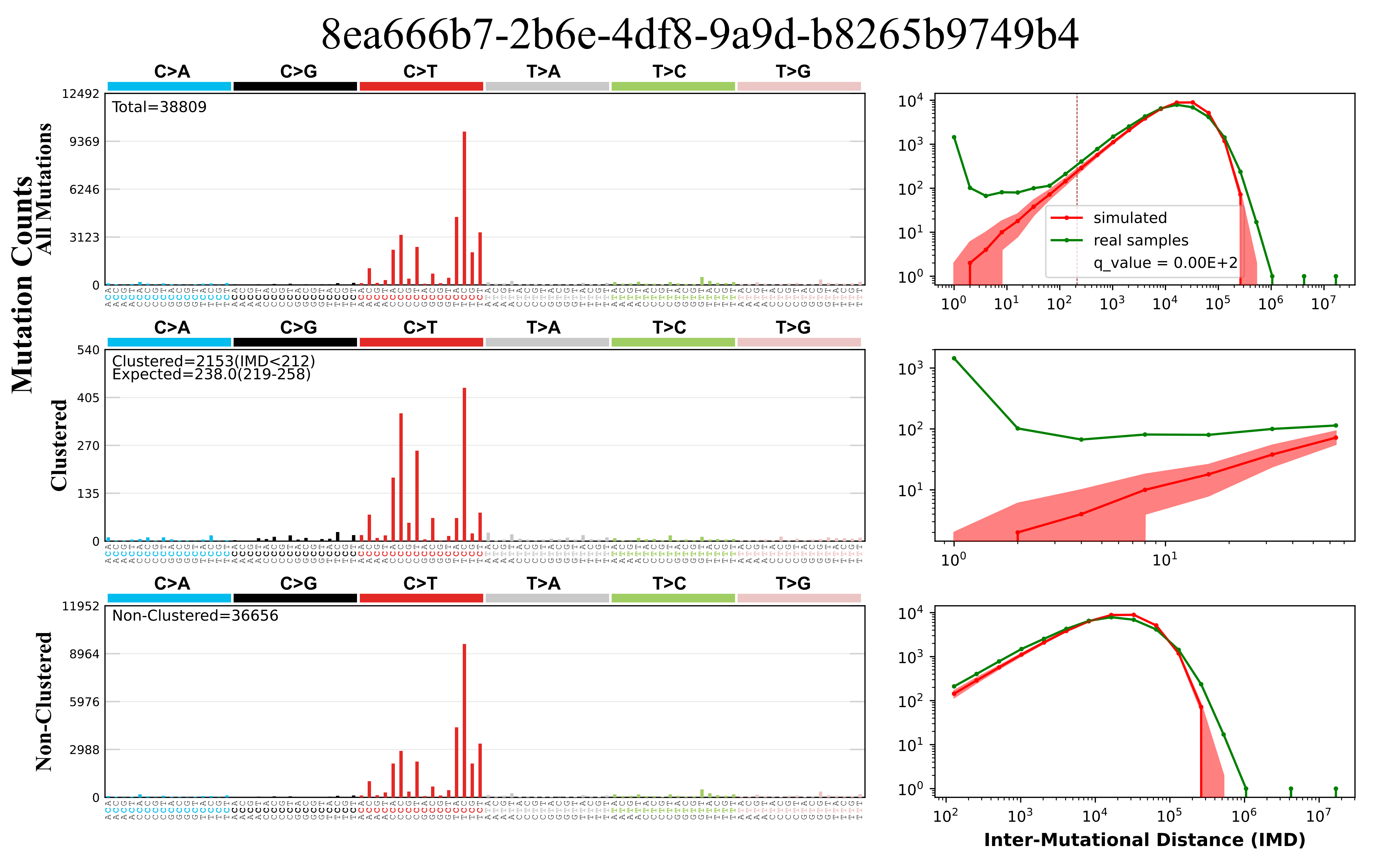
8ea666b7-2b6e-4df8-9a9d-b8265b9749b4
Mutation Counts
All Mutations
Total=38809
Clustered
Clustered=2153(IMD<212)
Expected=238.0(219-258)
Non-Clustered
Non-Clustered=36656
C>A
C>G
C>T
T>A
T>C
T>G
simulated
real samples
q_value = 0.00E+2
Inter-Mutational Distance (IMD)

c21a5d55-9790-429f-a4e6-420293c07b53

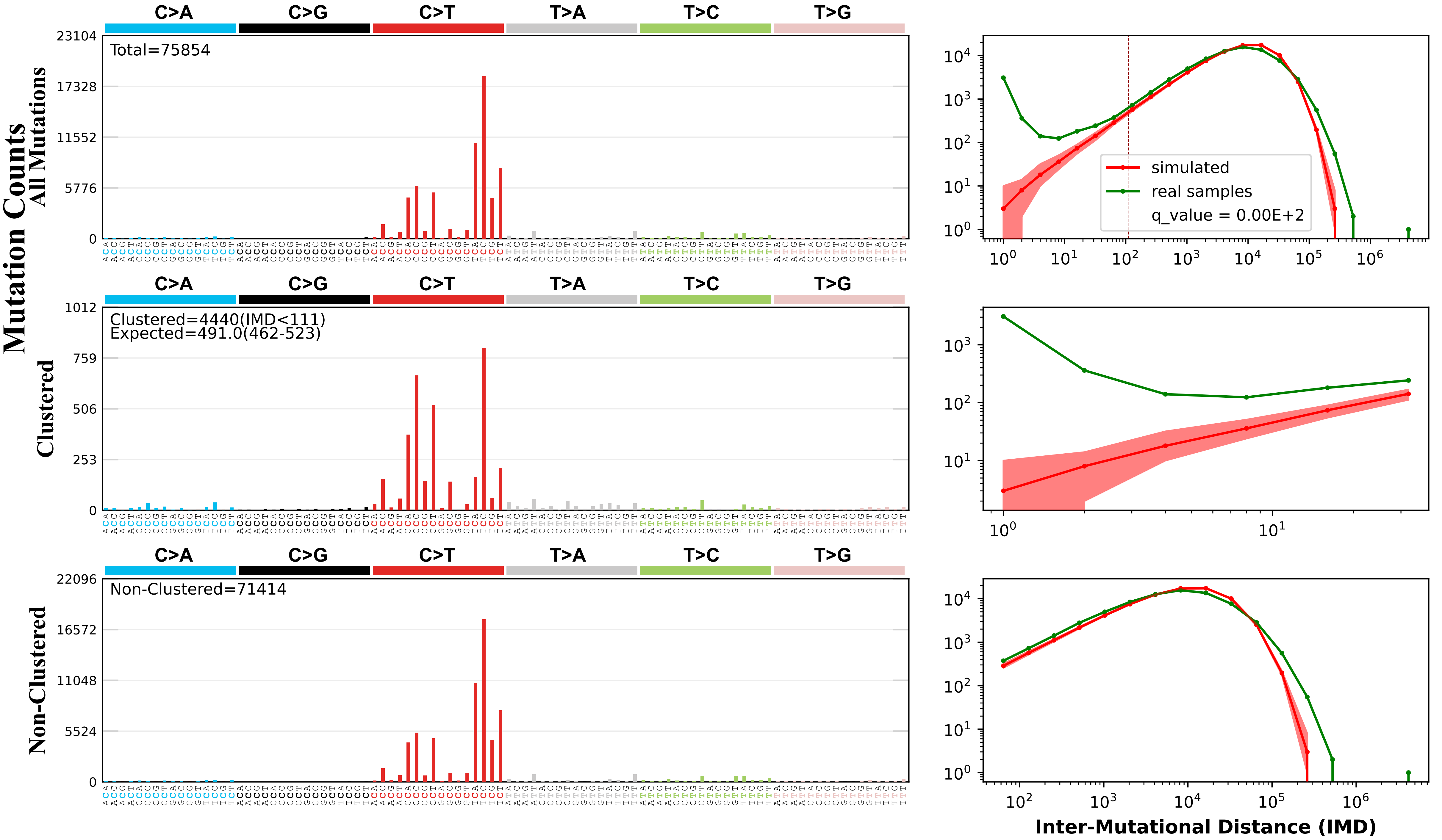

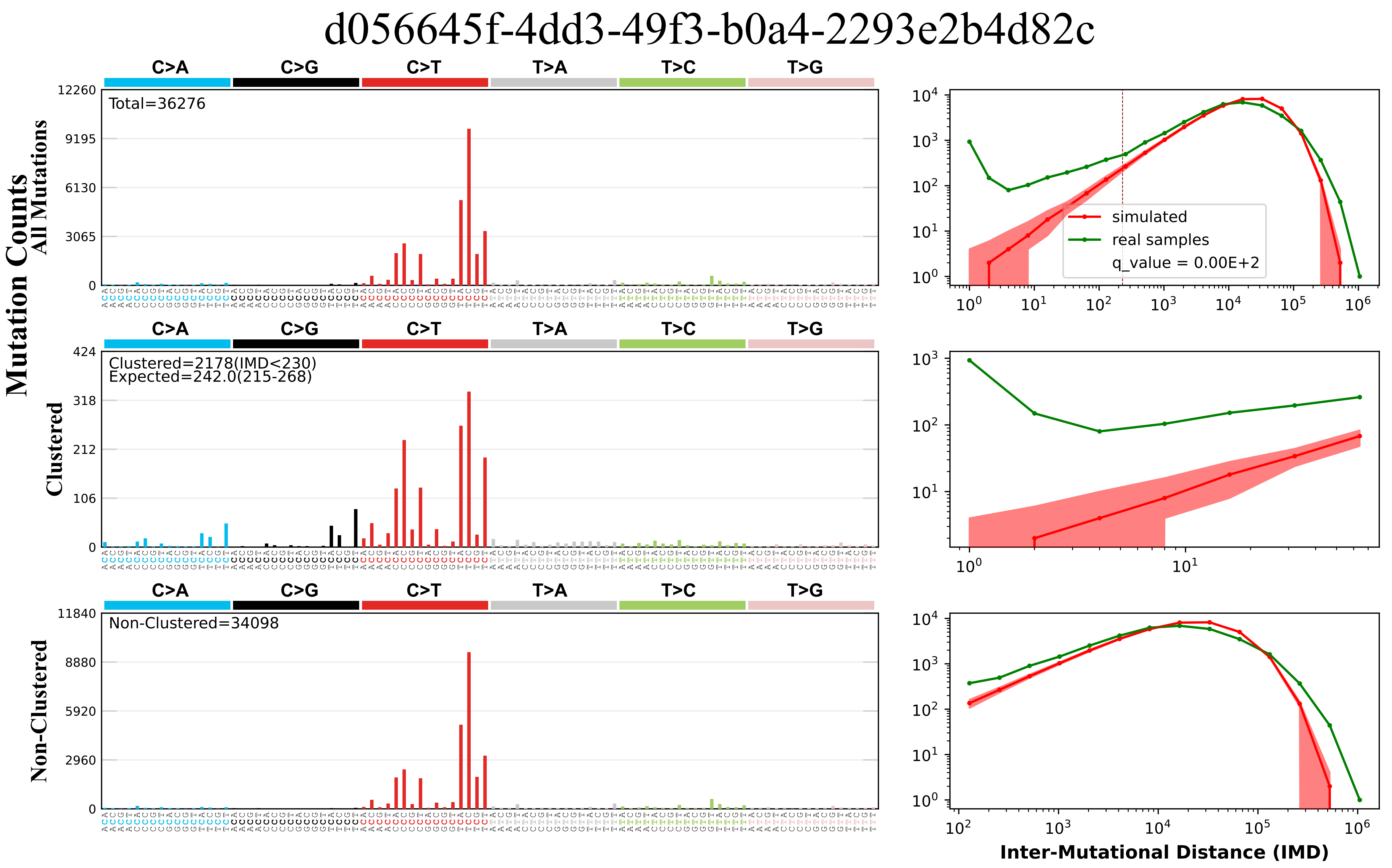

d056645f-4dd3-49f3-b0a4-2293e2b4d82c
Mutation Counts
All Mutations
Total=36276
Clustered
Clustered=2178(IMD<230)
Expected=242.0(215-268)
Non-Clustered
Non-Clustered=34098
C>A
C>G
C>T
T>A
T>C
T>G
simulated
real samples
q_value = 0.00E+2
Inter-Mutational Distance (IMD)

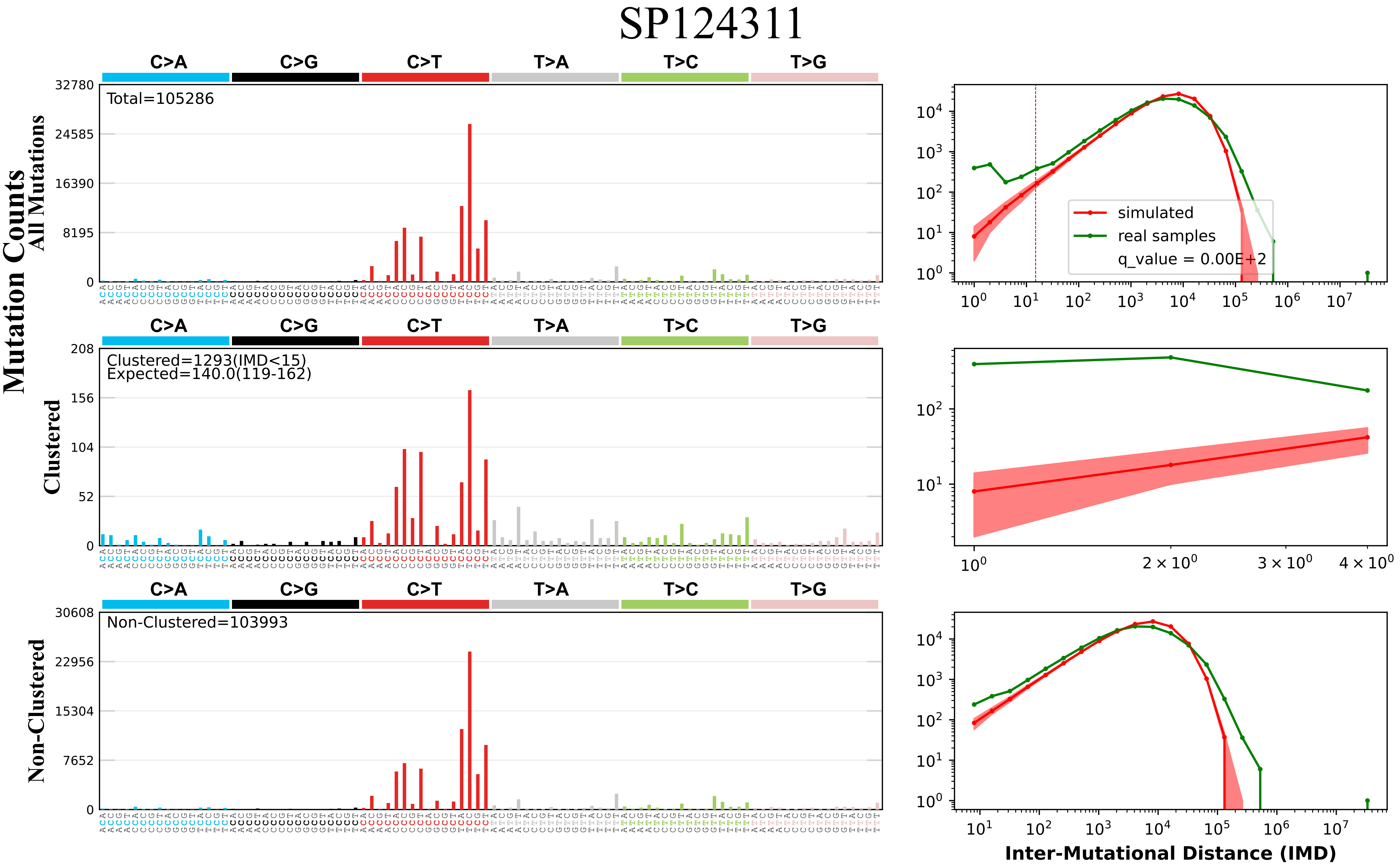

SP124311
C>A
C>G
C>T
T>A
T>C
T>G
Mutation Counts
All Mutations
Total=105286
Clustered
Clustered=1293(IMD<15)
Expected=140.0(119-162)
Non-Clustered
Non-Clustered=103993
simulated
real samples
q_value = 0.00E+2
Inter-Mutational Distance (IMD)

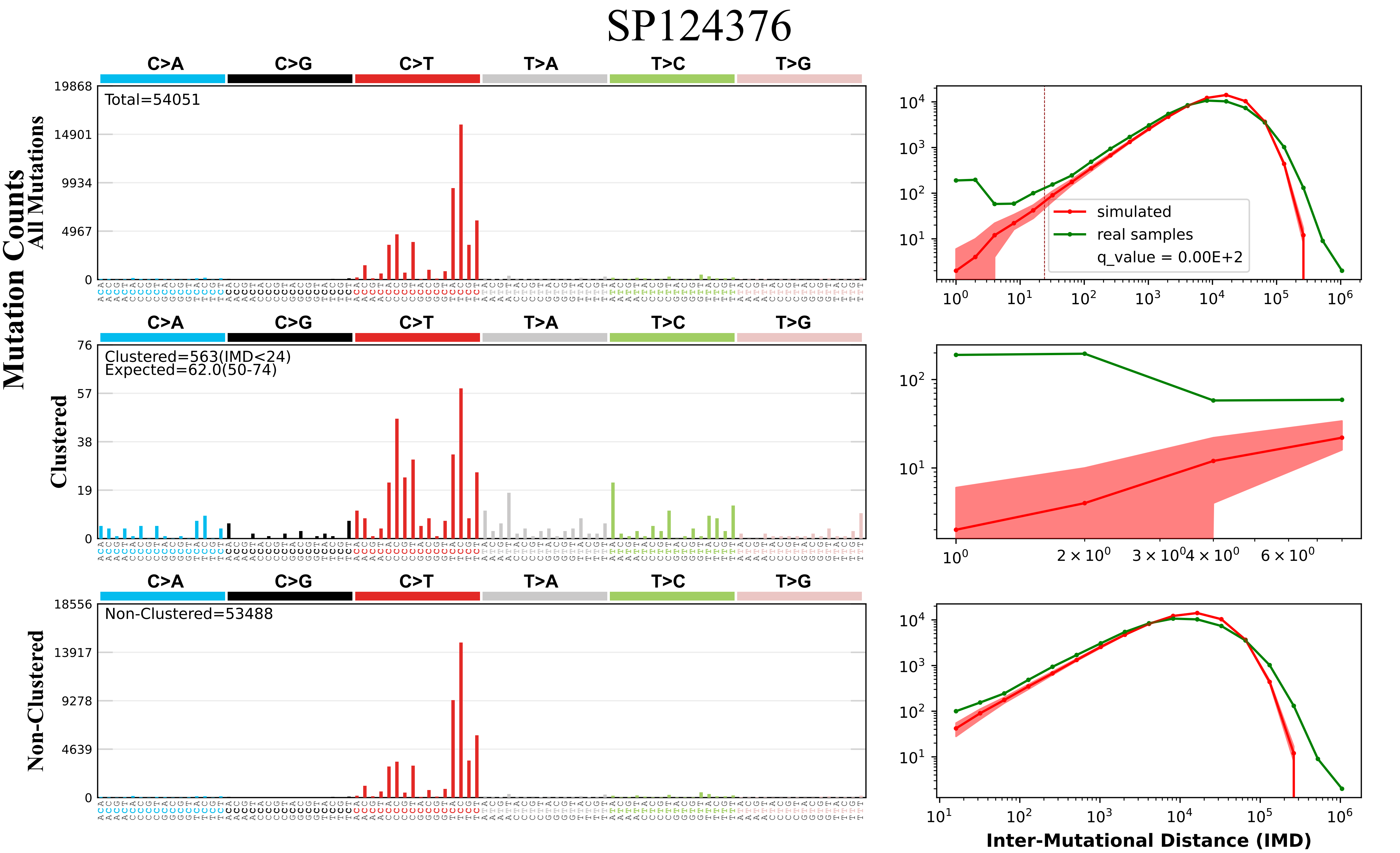

SP124376
Mutation Counts
All Mutations
Clustered
Non-Clustered
C>A
C>G
C>T
T>A
T>C
T>G
Total=54051
Clustered=563(IMD<24)
Expected=62.0(50-74)
Non-Clustered=53488
simulated
real samples
q_value = 0.00E+2
Inter-Mutational Distance (IMD)

54a77010-2f16-4da9-8ca0-746a51801a00

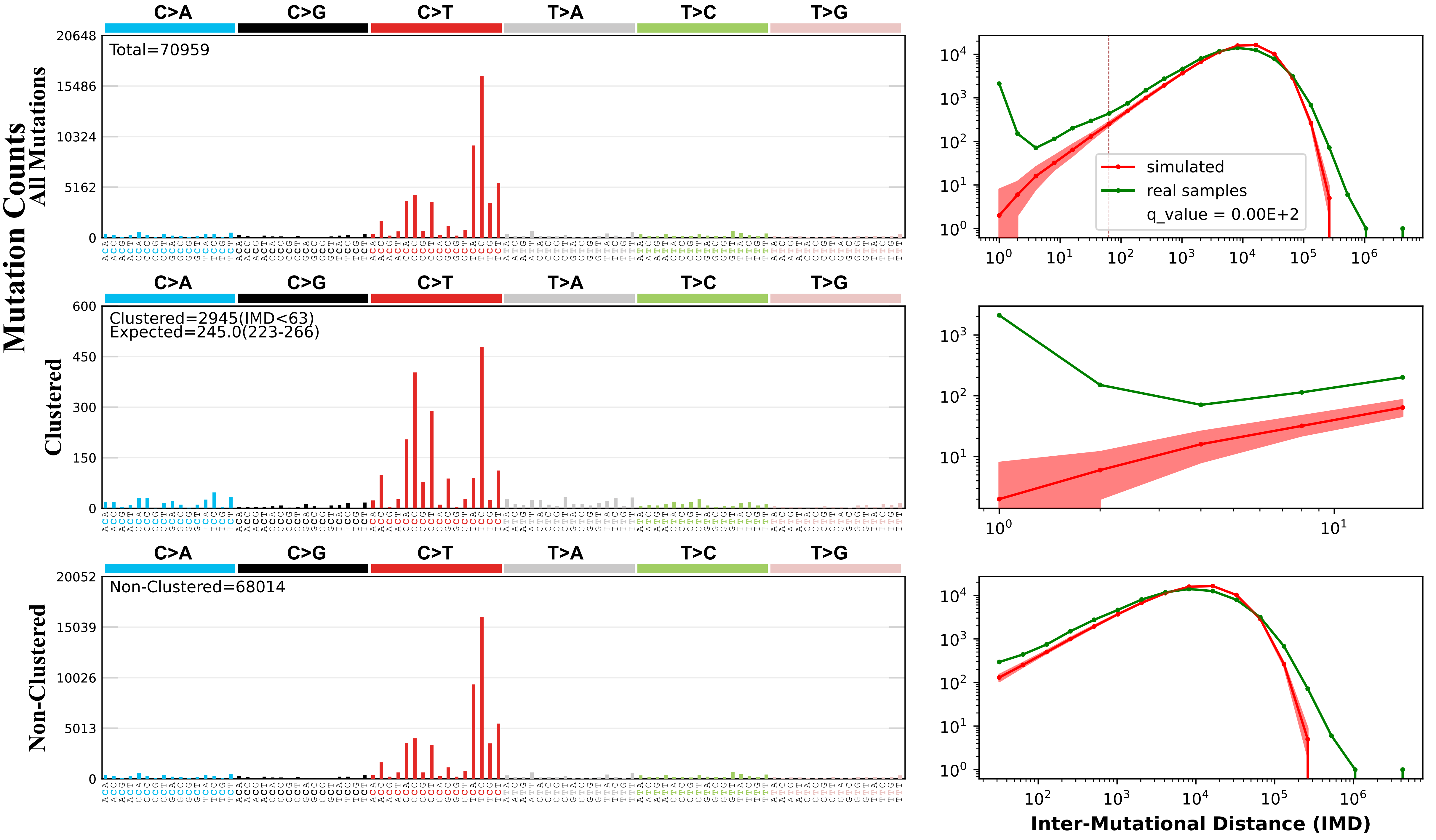

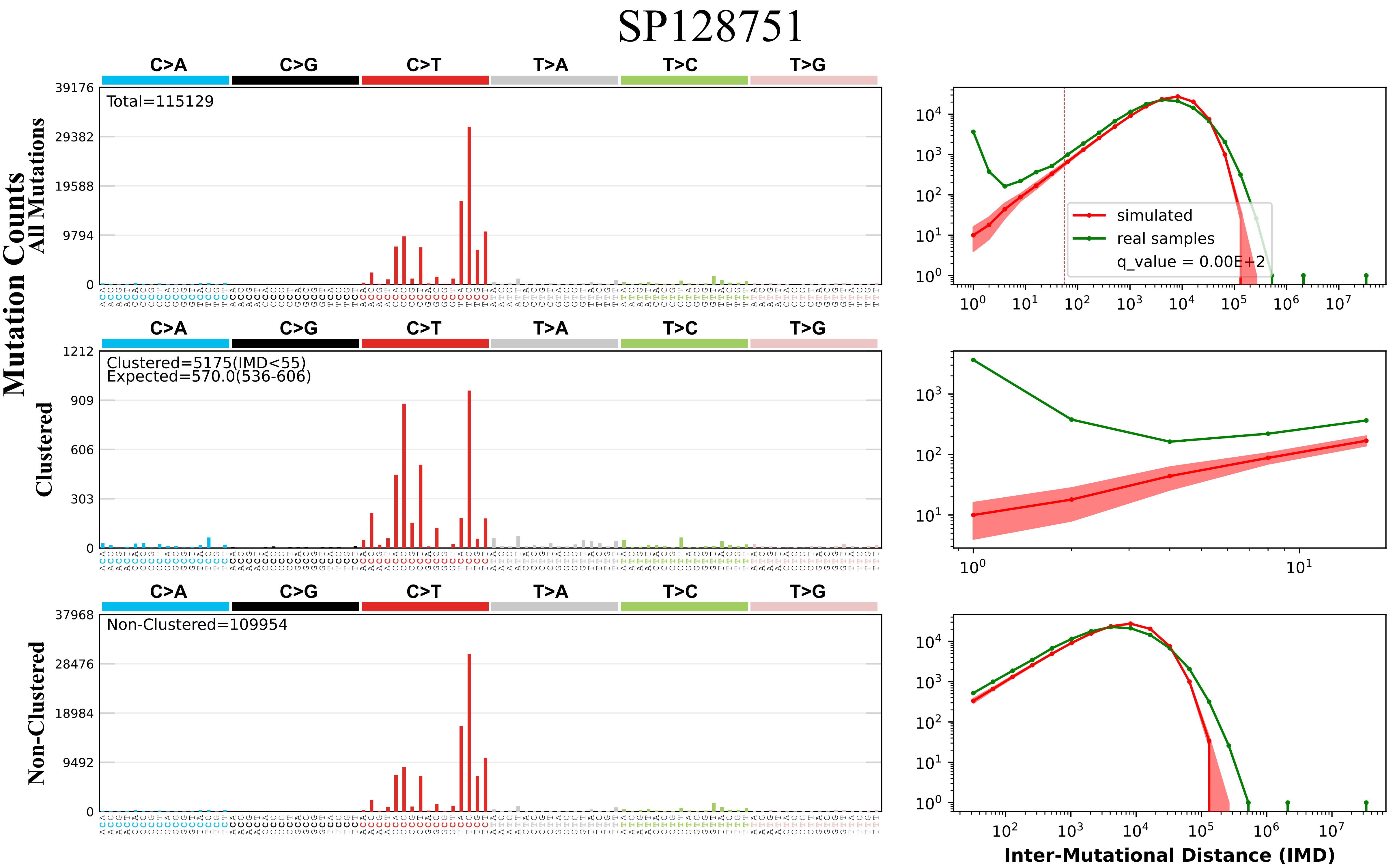
SP128751
Mutation Counts
All Mutations
Total=115129
C>A
C>G
C>T
T>A
T>C
T>G
39176
29382
19588
9794
0
Clustered
Clustered=5175(IMD<55)
Expected=570.0(536-606)
1212
909
606
303
0
Non-Clustered
Non-Clustered=109954
37968
28476
18984
9492
0
simulated
real samples
q_value = 0.00E+2
Inter-Mutational Distance (IMD)

SP124454

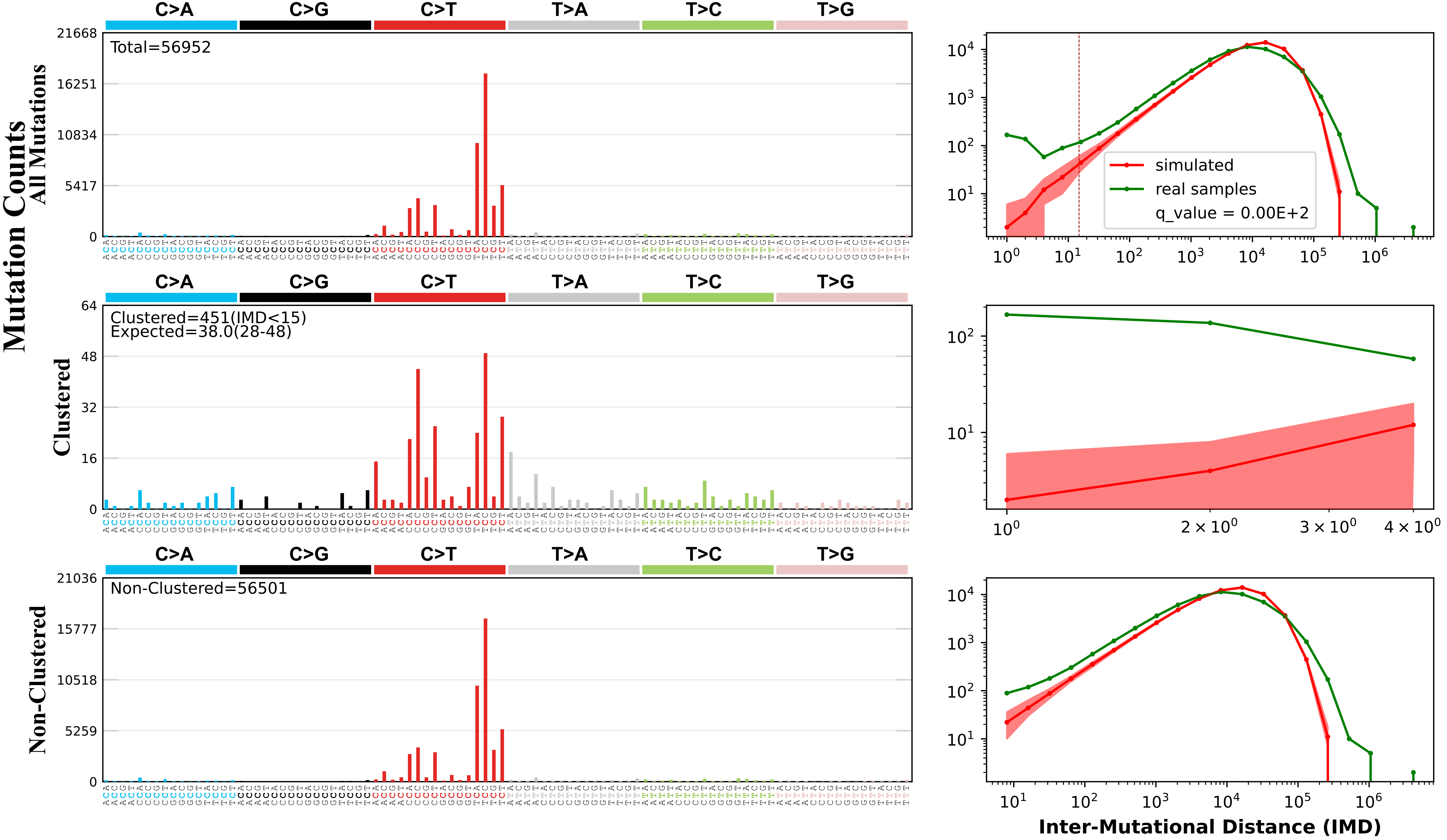

Mutation Counts
All Mutations
Total=56952
Clustered
Clustered=451(IMD<15)
Expected=38.0(28-48)
Non-Clustered
Non-Clustered=56501
C>A
C>G
C>T
T>A
T>C
T>G
simulated
real samples
q_value = 0.00E+2
Inter-Mutational Distance (IMD)

SP124307

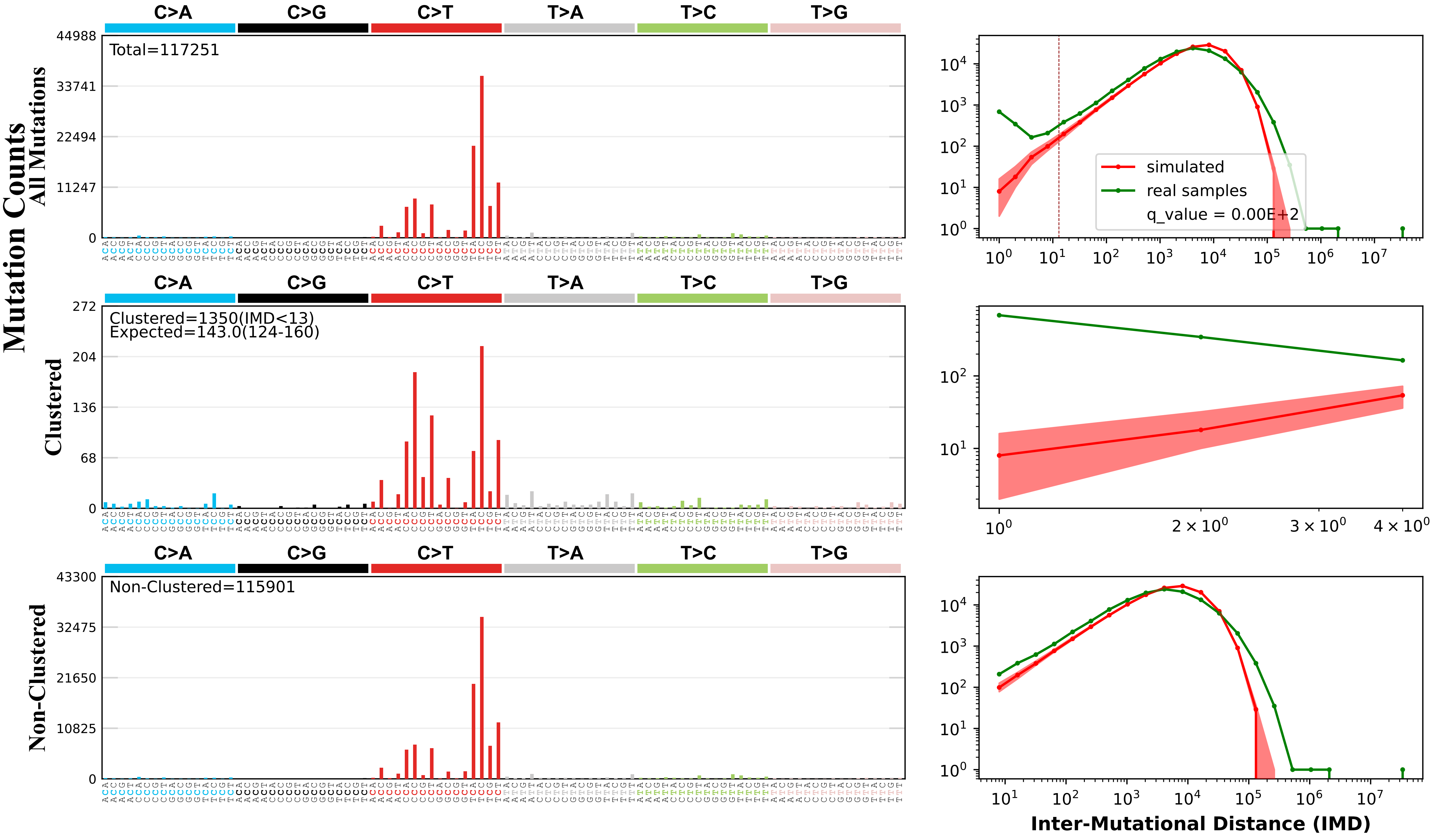
Mutation Counts
All Mutations
Clustered
Non-Clustered
C>A
C>G
C>T
T>A
T>C
T>G
Total=117251
Clustered=1350(IMD<13)
Expected=143.0(124-160)
Non-Clustered=115901
simulated
real samples
q_value = 0.00E+2
Inter-Mutational Distance (IMD)

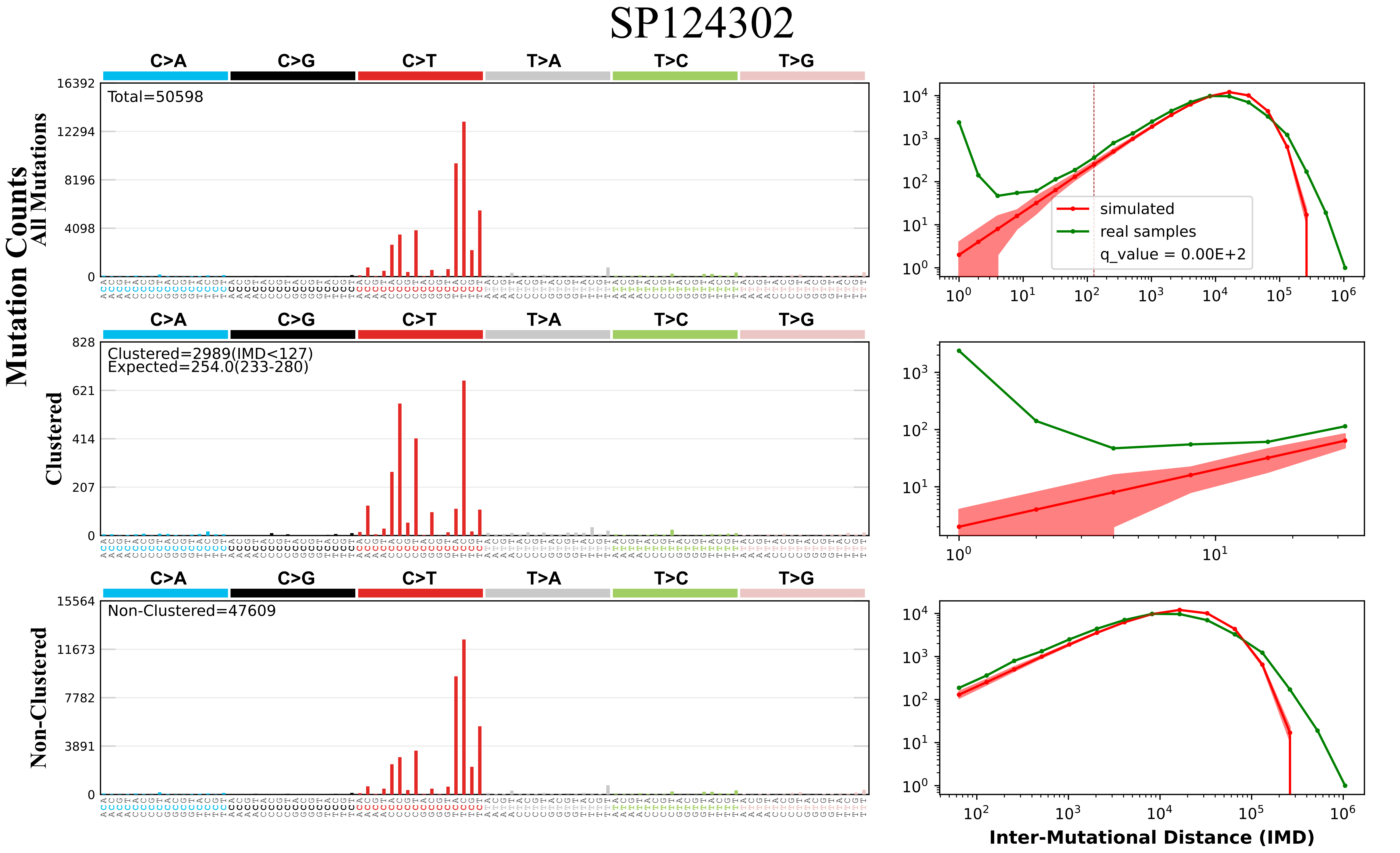

SP124302
Mutation Counts
All Mutations
Total=50598
Clustered
Clustered=2989(IMD<127)
Expected=254.0(233-280)
Non-Clustered
Non-Clustered=47609
C>A
C>G
C>T
T>A
T>C
T>G
simulated
real samples
q_value = 0.00E+2
Inter-Mutational Distance (IMD)

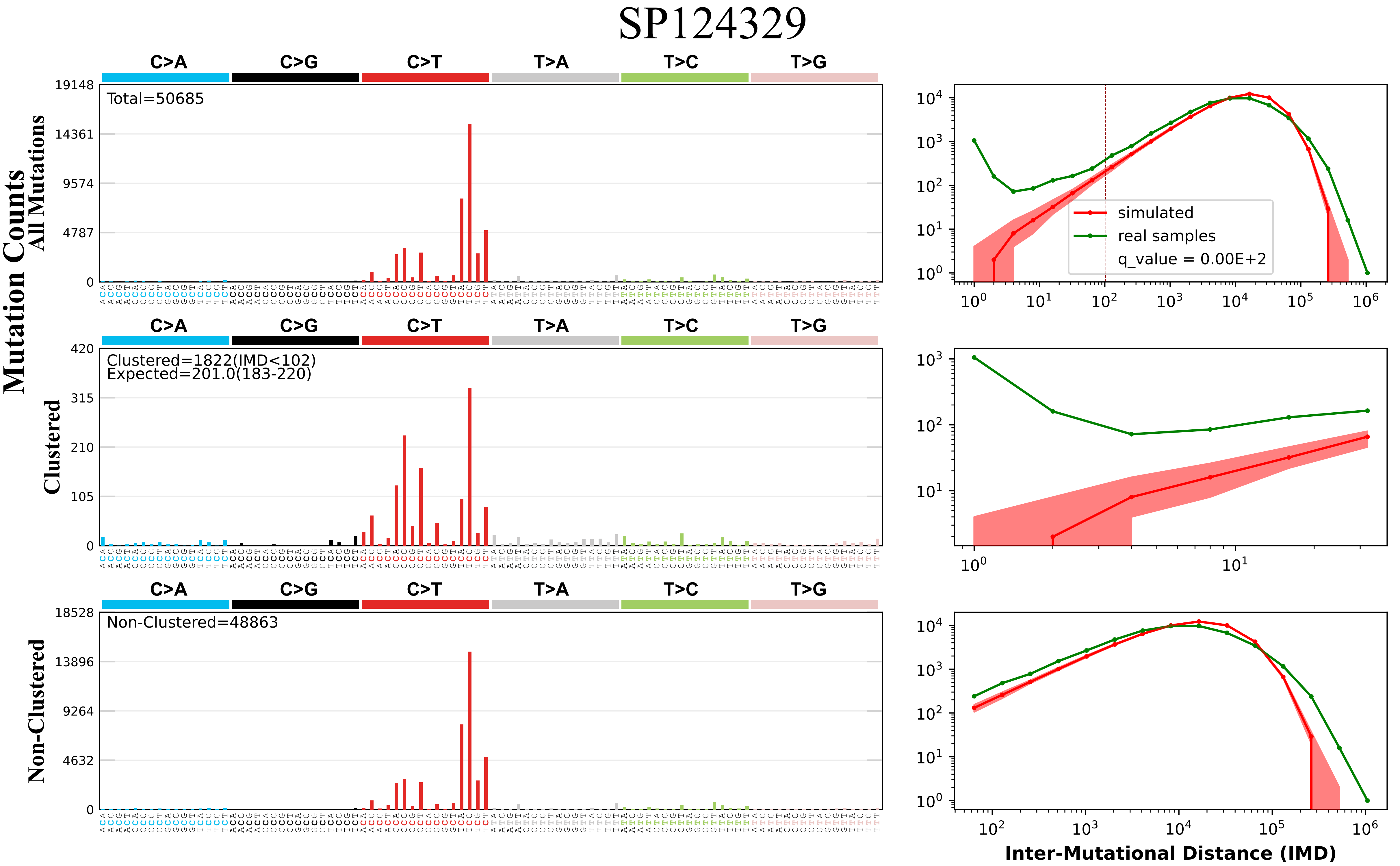
SP124329
C>A
C>G
C>T
T>A
T>C
T>G
Mutation Counts
All Mutations
Total=50685
19148
14361
9574
4787
0
Clustered
Clustered=1822(IMD<102)
Expected=201.0(183-220)
420
315
210
105
Non-Clustered
Non-Clustered=48863
18528
13896
9264
4632
simulated
real samples
q_value = 0.00E+2
Inter-Mutational Distance (IMD)

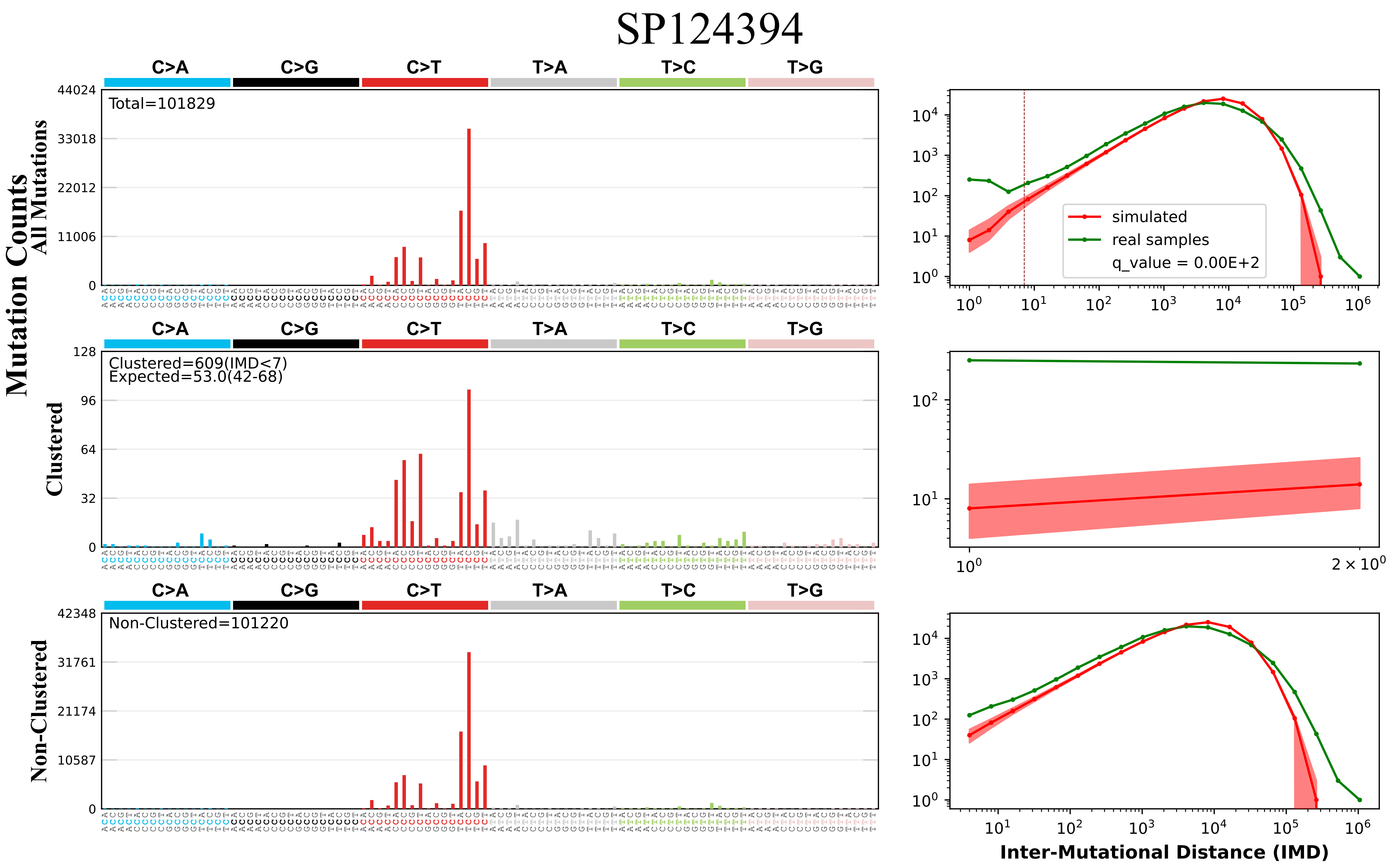

SP124394
C>A
C>G
C>T
T>A
T>C
T>G
Mutation Counts
All Mutations
Total=101829
Clustered
Clustered=609(IMD<7)
Expected=53.0(42-68)
Non-Clustered
Non-Clustered=101220
simulated
real samples
q_value = 0.00E+2
Inter-Mutational Distance (IMD)

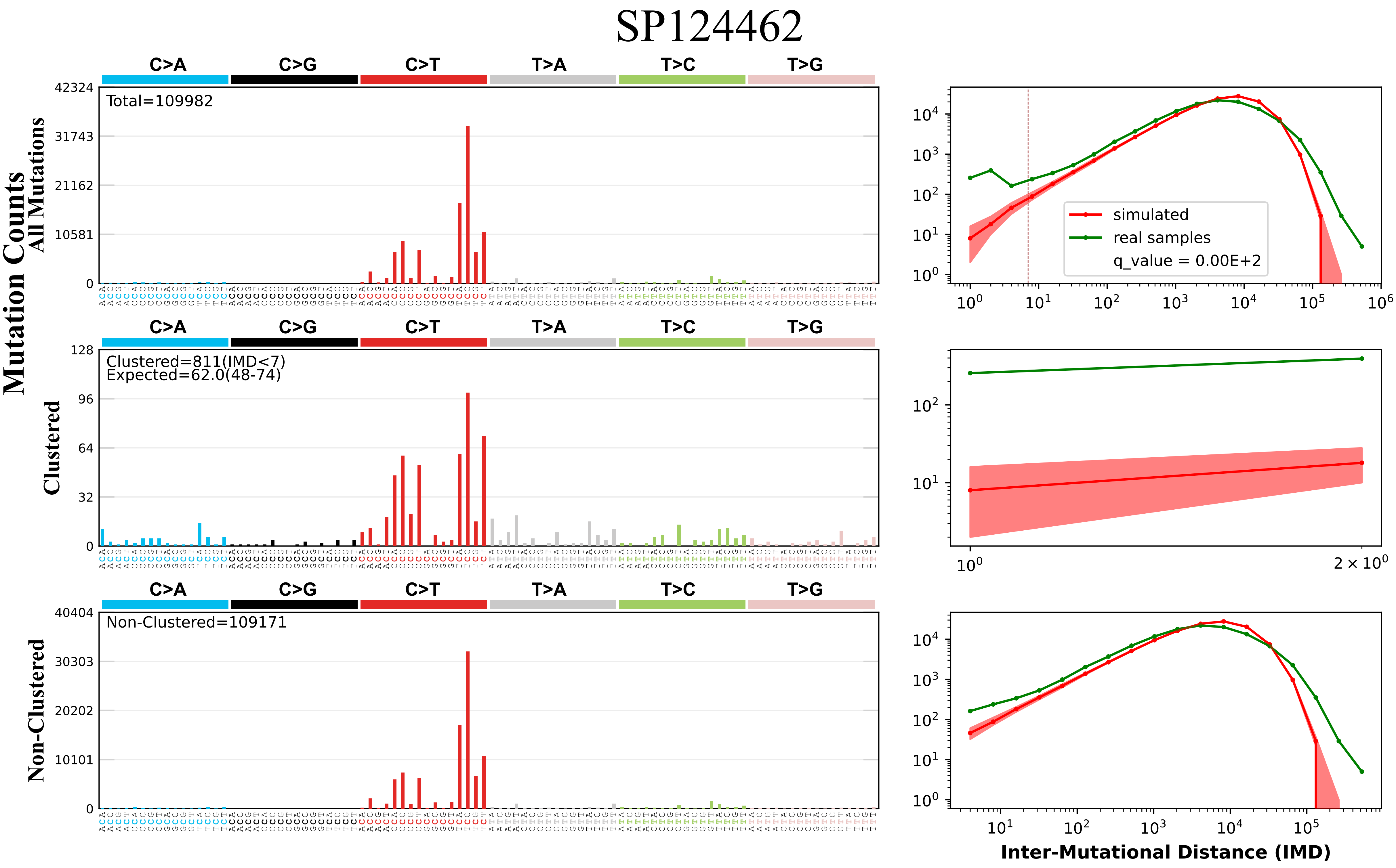
SP124462
Mutation Counts
All Mutations
Total=109982
Clustered
Clustered=811(IMD<7)
Expected=62.0(48-74)
Non-Clustered
Non-Clustered=109171
C>A
C>G
C>T
T>A
T>C
T>G
simulated
real samples
q_value = 0.00E+2
Inter-Mutational Distance (IMD)

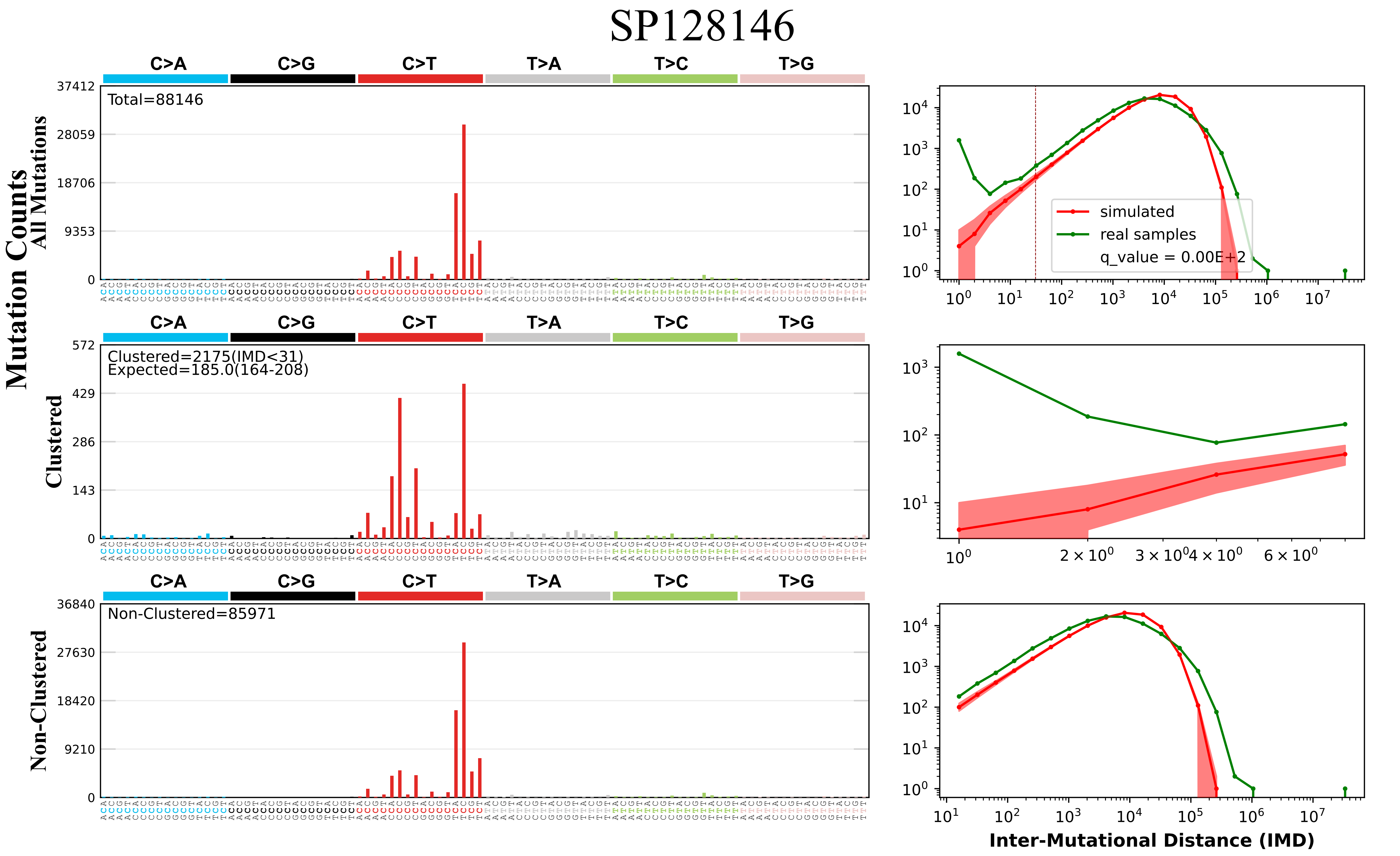
SP128146
Mutation Counts
All Mutations
Total=88146
C>A
C>G
C>T
T>A
T>C
T>G
37412
28059
18706
9353
0
simulated
real samples
q_value = 0.00E+2
Clustered
Clustered=2175(IMD<31)
Expected=185.0(164-208)
572
429
286
143
Non-Clustered
Non-Clustered=85971
36840
27630
18420
9210
Inter-Mutational Distance (IMD)

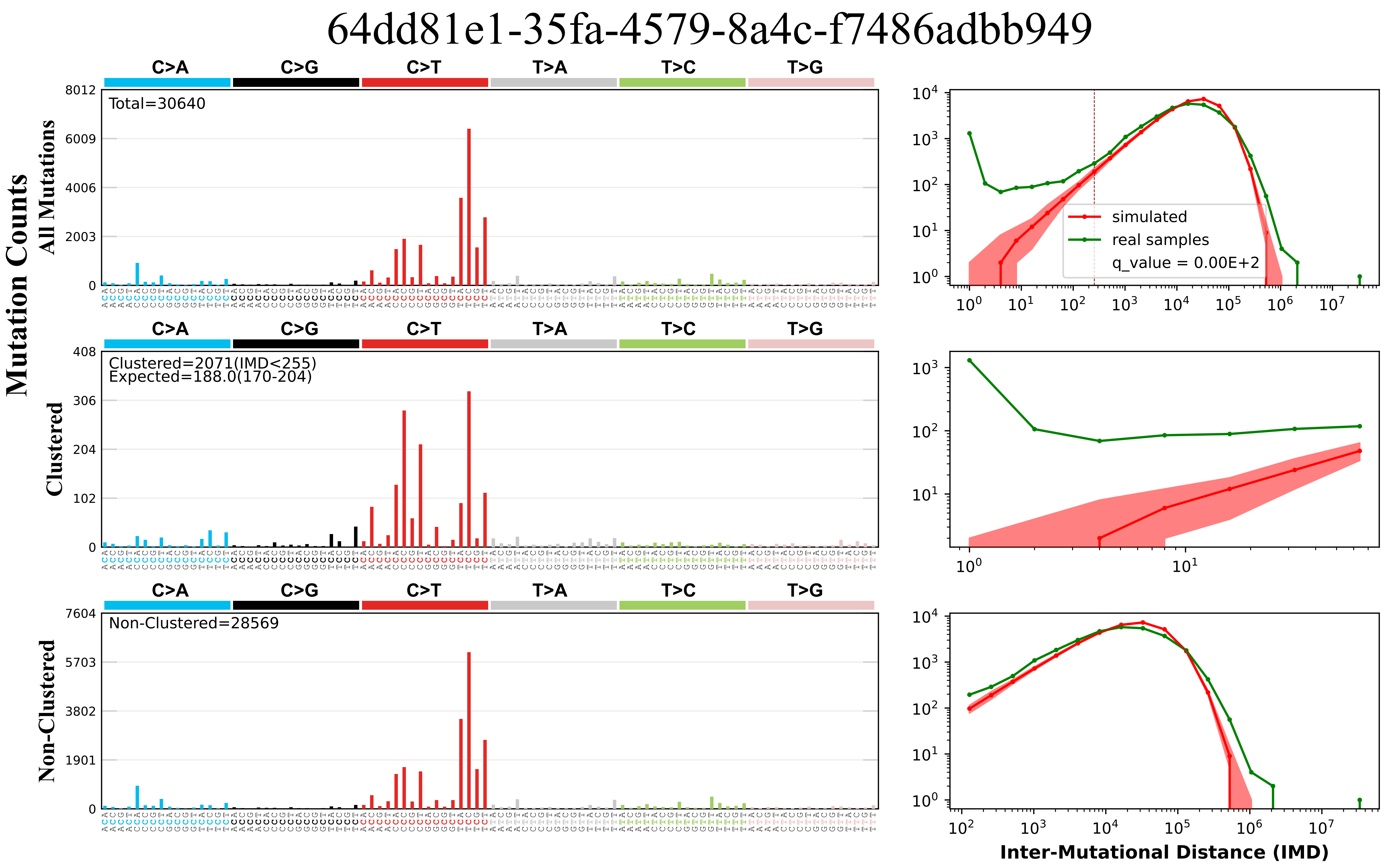
64dd81e1-35fa-4579-8a4c-f7486adbb949
Mutation Counts
All Mutations
Total=30640
Clustered
Clustered=2071(IMD<255)
Expected=188.0(170-204)
Non-Clustered
Non-Clustered=28569
C>A
C>G
C>T
T>A
T>C
T>G
simulated
real samples
q_value = 0.00E+2
Inter-Mutational Distance (IMD)

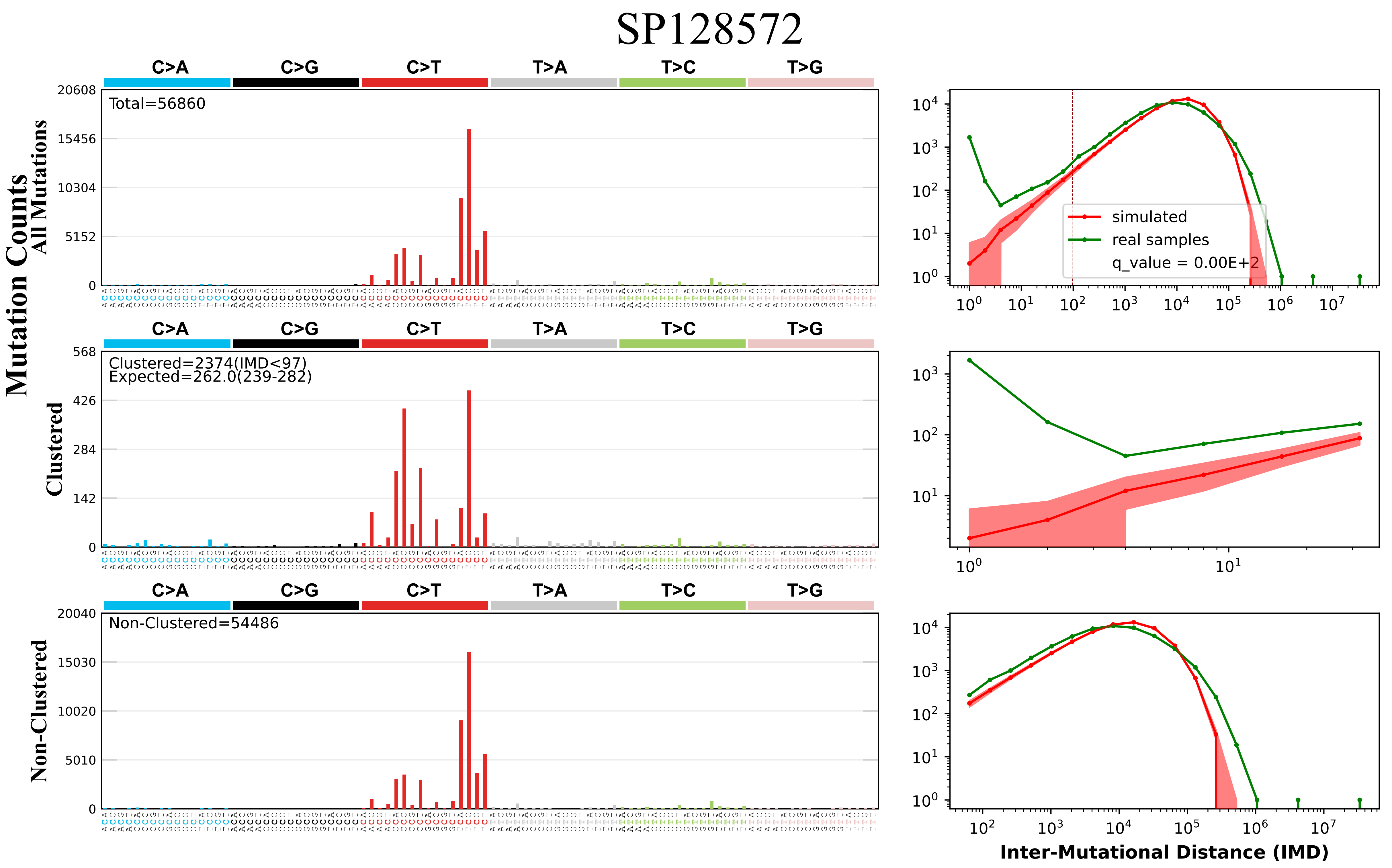
SP128572
Mutation Counts
All Mutations
C>A
C>G
C>T
T>A
T>C
T>G
Total=56860
Clustered
Clustered=2374(IMD<97)
Expected=262.0(239-282)
Non-Clustered
Non-Clustered=54486
simulated
real samples
q_value = 0.00E+2
Inter-Mutational Distance (IMD)

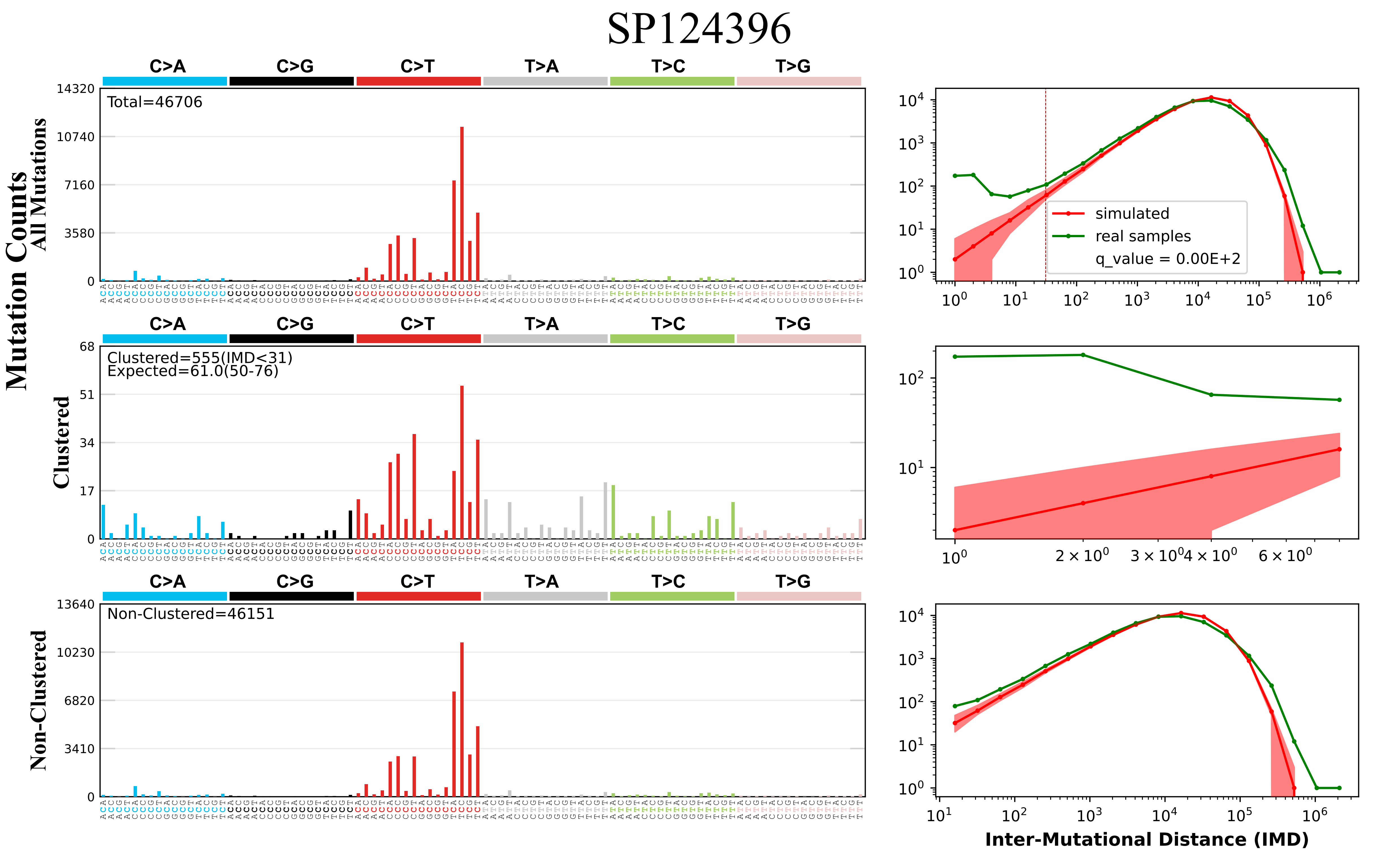
SP124396
Mutation Counts
All Mutations
Clustered
Non-Clustered
C>A
C>G
C>T
T>A
T>C
T>G
Total=46706
Clustered=555(IMD<31)
Expected=61.0(50-76)
Non-Clustered=46151
simulated
real samples
q_value = 0.00E+2
Inter-Mutational Distance (IMD)

66d312fc-809d-428b-98e3-a29d211db35c

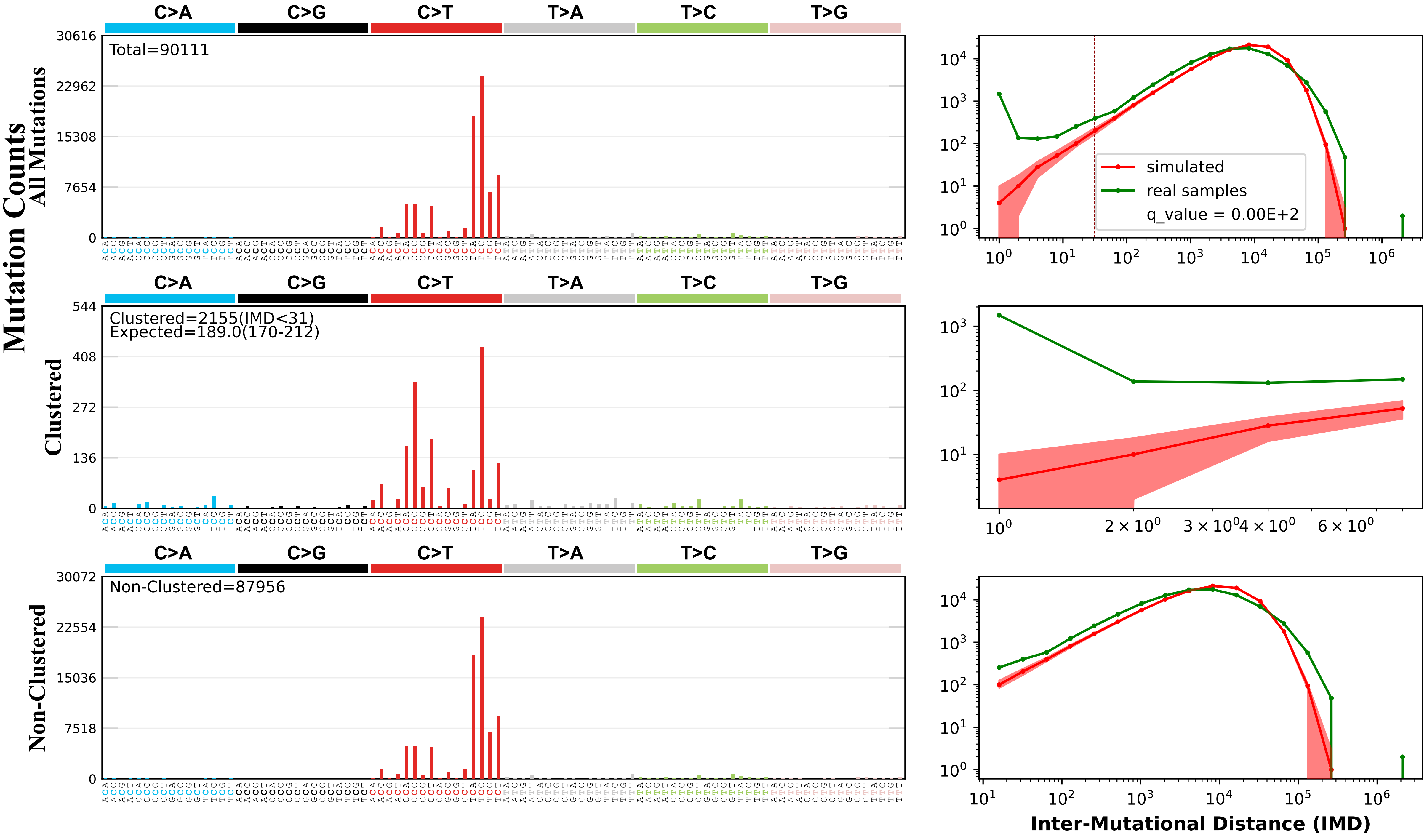

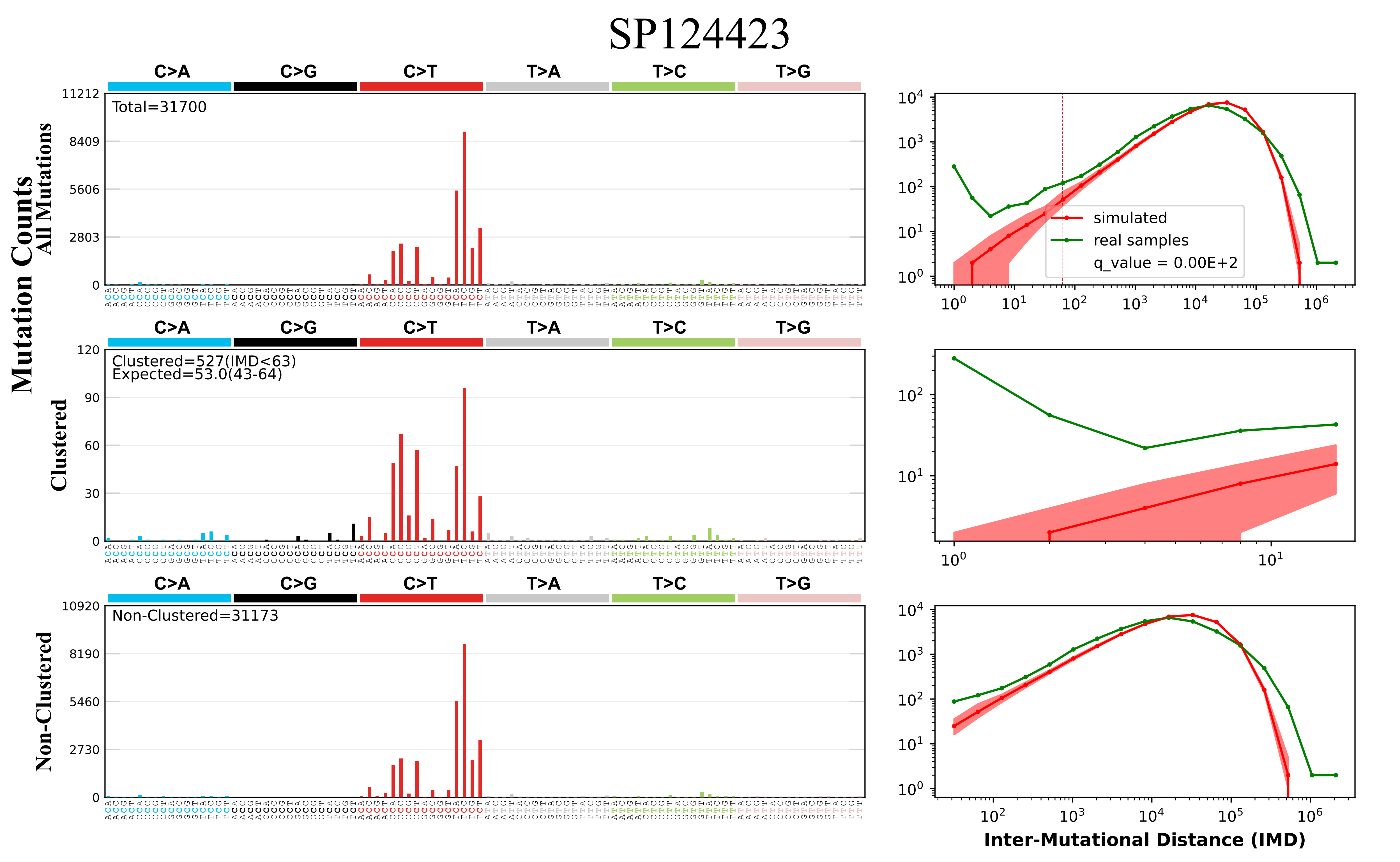

SP124423
Mutation Counts
All Mutations
Total=31700
Clustered
Clustered=527(IMD<63)
Expected=53.0(43-64)
Non-Clustered
Non-Clustered=31173
C>A
C>G
C>T
T>A
T>C
T>G
simulated
real samples
q_value = 0.00E+2
Inter-Mutational Distance (IMD)

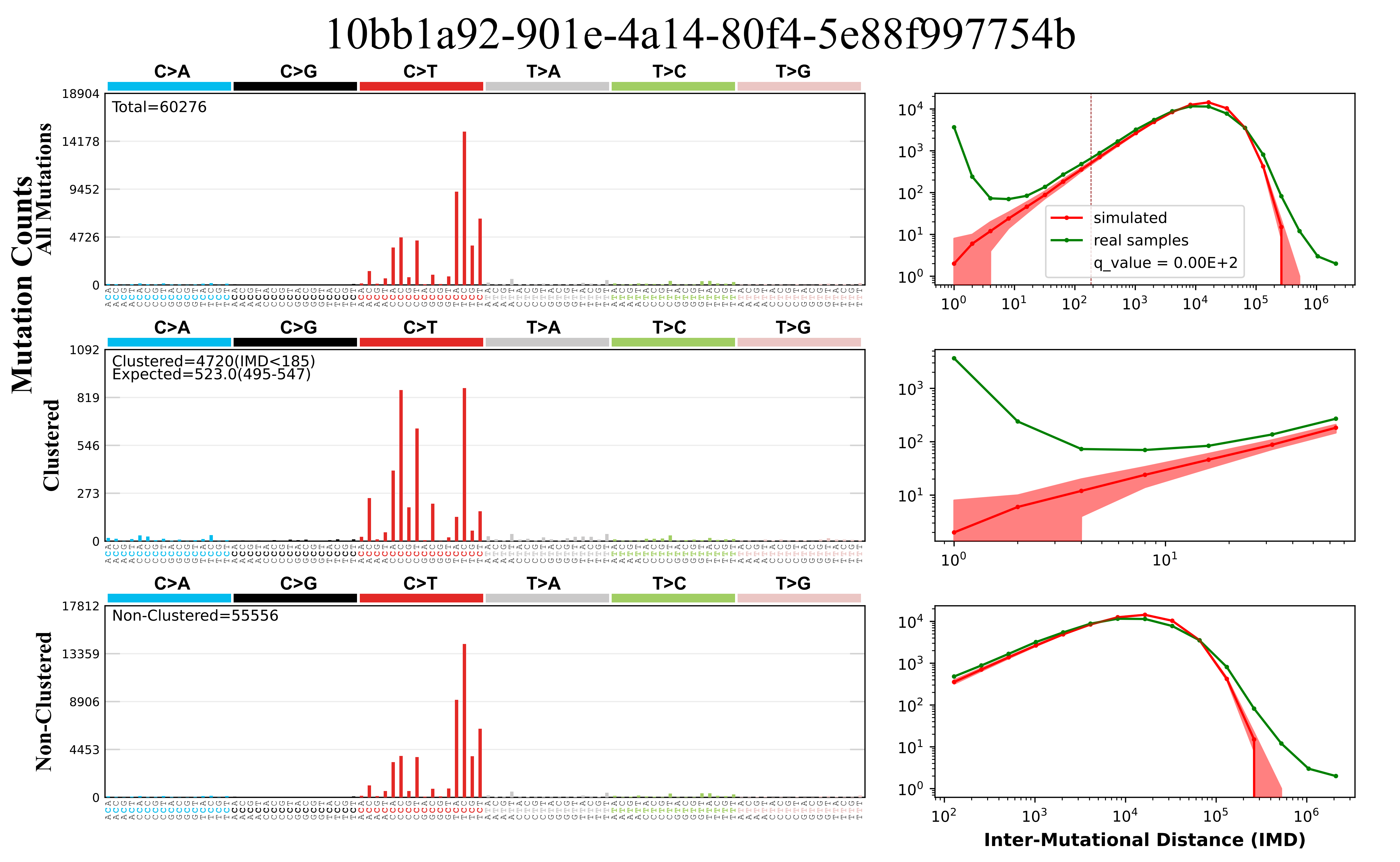
10bb1a92-901e-4a14-80f4-5e88f997754b
Mutation Counts
All Mutations
Total=60276
Clustered
Clustered=4720(IMD<185)
Expected=523.0(495-547)
Non-Clustered
Non-Clustered=55556
C>A
C>G
C>T
T>A
T>C
T>G
simulated
real samples
q_value = 0.00E+2
Inter-Mutational Distance (IMD)

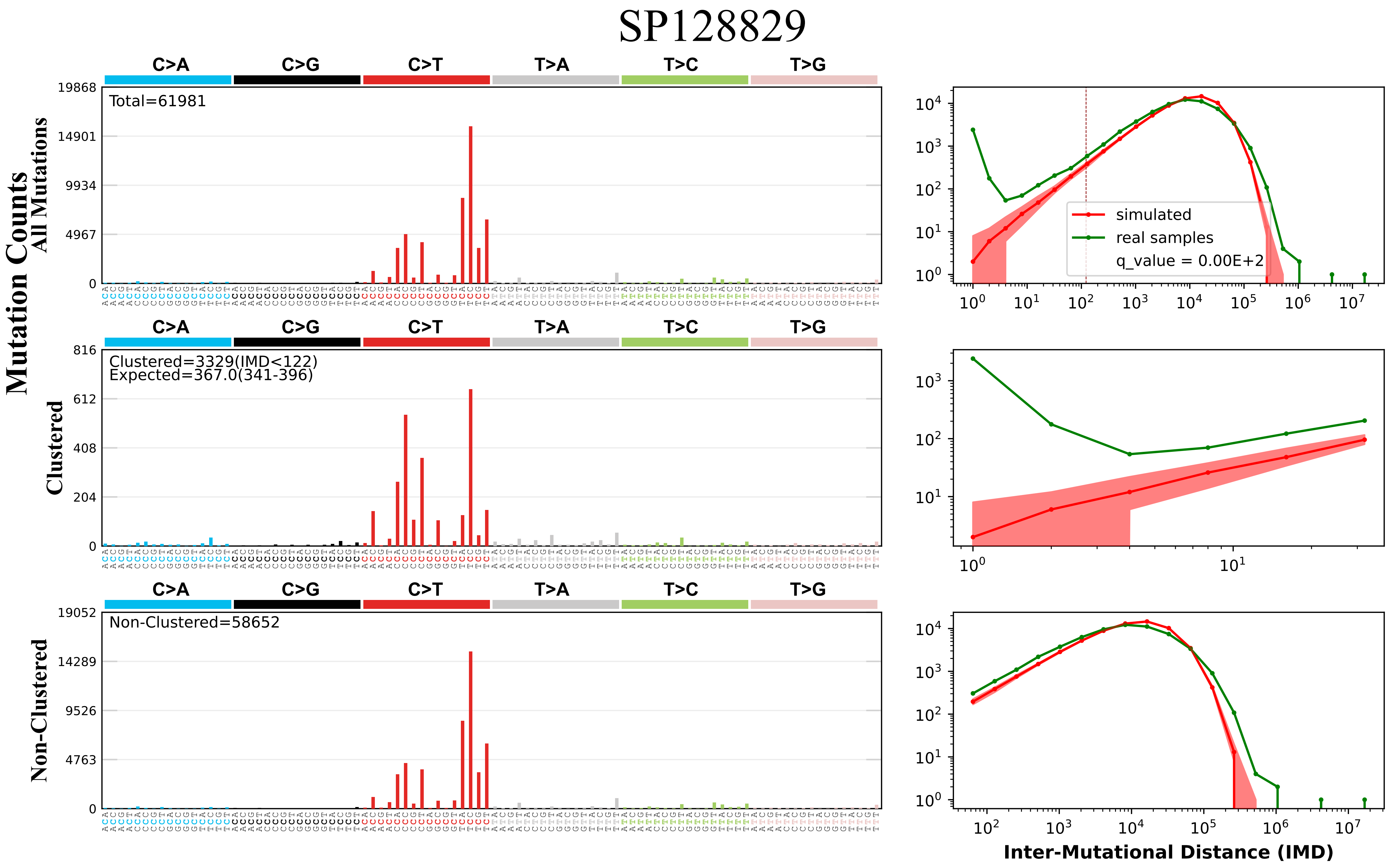
SP128829
Mutation Counts
All Mutations
Clustered
Non-Clustered
C>A
C>G
C>T
T>A
T>C
T>G
Total=61981
Clustered=3329(IMD<122)
Expected=367.0(341-396)
Non-Clustered=58652
simulated
real samples
q_value = 0.00E+2
Inter-Mutational Distance (IMD)

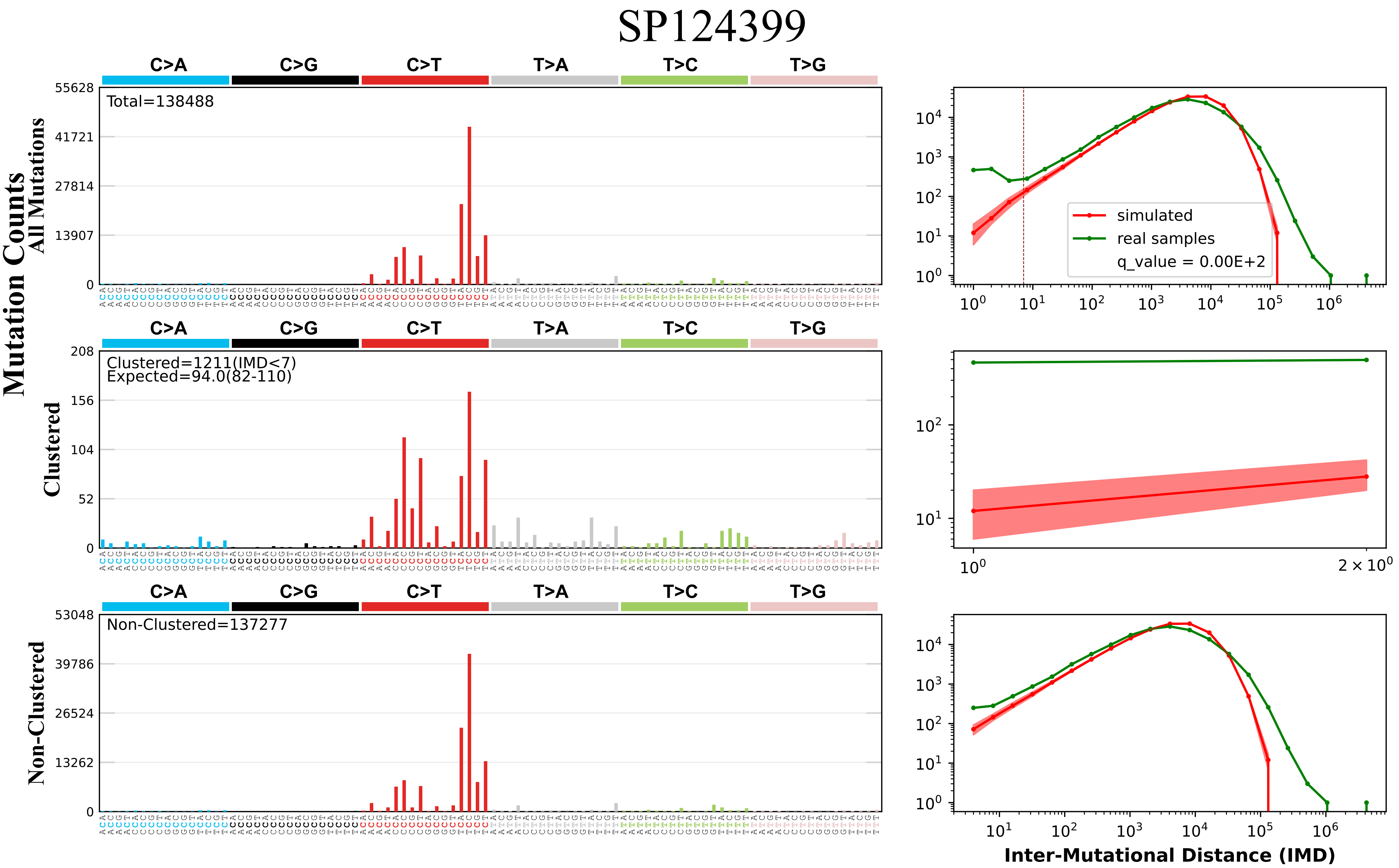

SP124399
Mutation Counts
All Mutations
Clustered
Non-Clustered
C>A
C>G
C>T
T>A
T>C
T>G
Total=138488
Clustered=1211(IMD<7)
Expected=94.0(82-110)
Non-Clustered=137277
simulated
real samples
q_value = 0.00E+2
Inter-Mutational Distance (IMD)

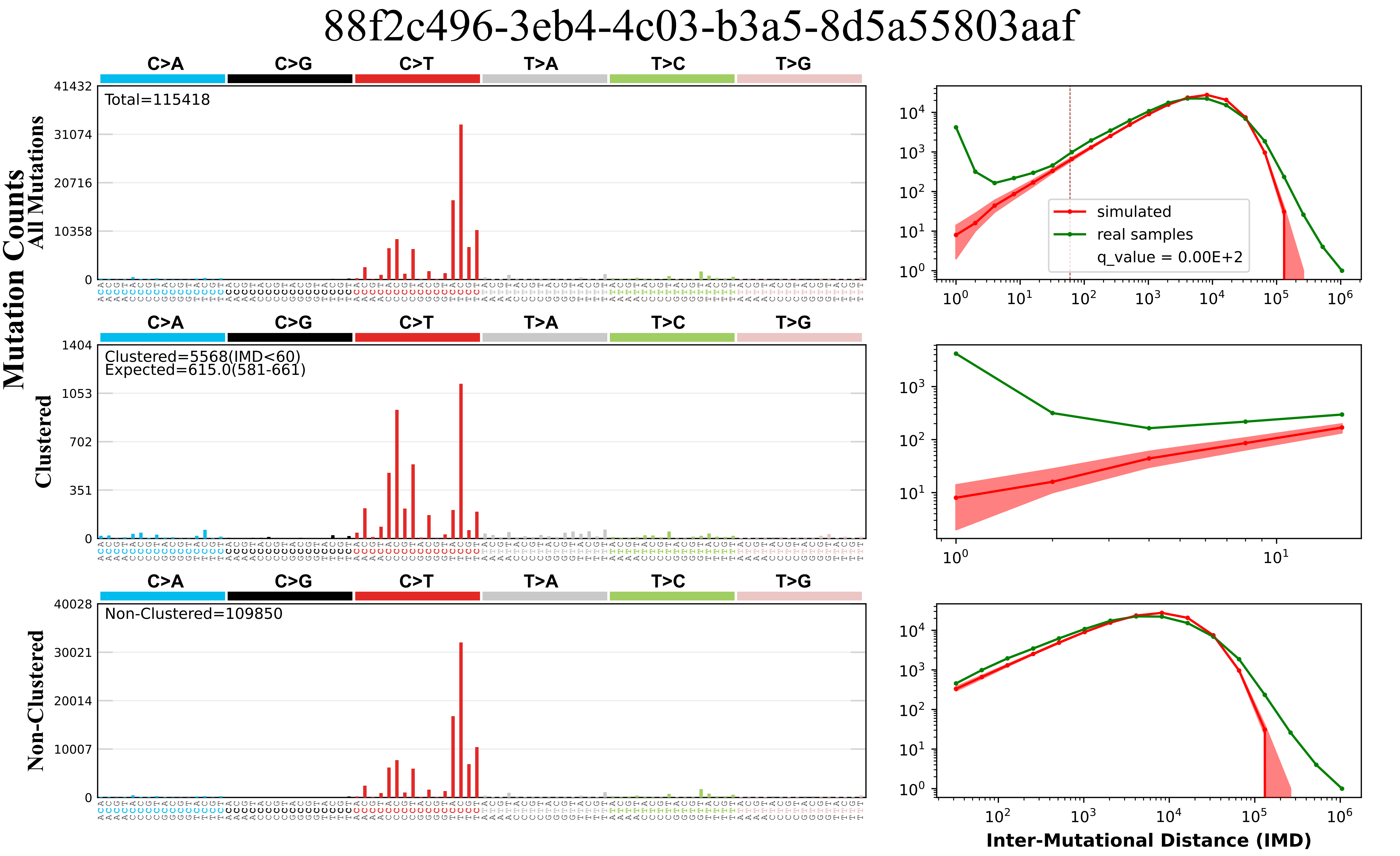
88f2c496-3eb4-4c03-b3a5-8d5a55803aaf
Mutation Counts
All Mutations
C>A
C>G
C>T
T>A
T>C
T>G
Total=115418
41432
31074
20716
10358
0
simulated
real samples
q_value = 0.00E+2
Clustered
Clustered=5568(IMD<60)
Expected=615.0(581-661)
1404
1053
702
351
Non-Clustered
Non-Clustered=109850
40028
30021
20014
10007
Inter-Mutational Distance (IMD)

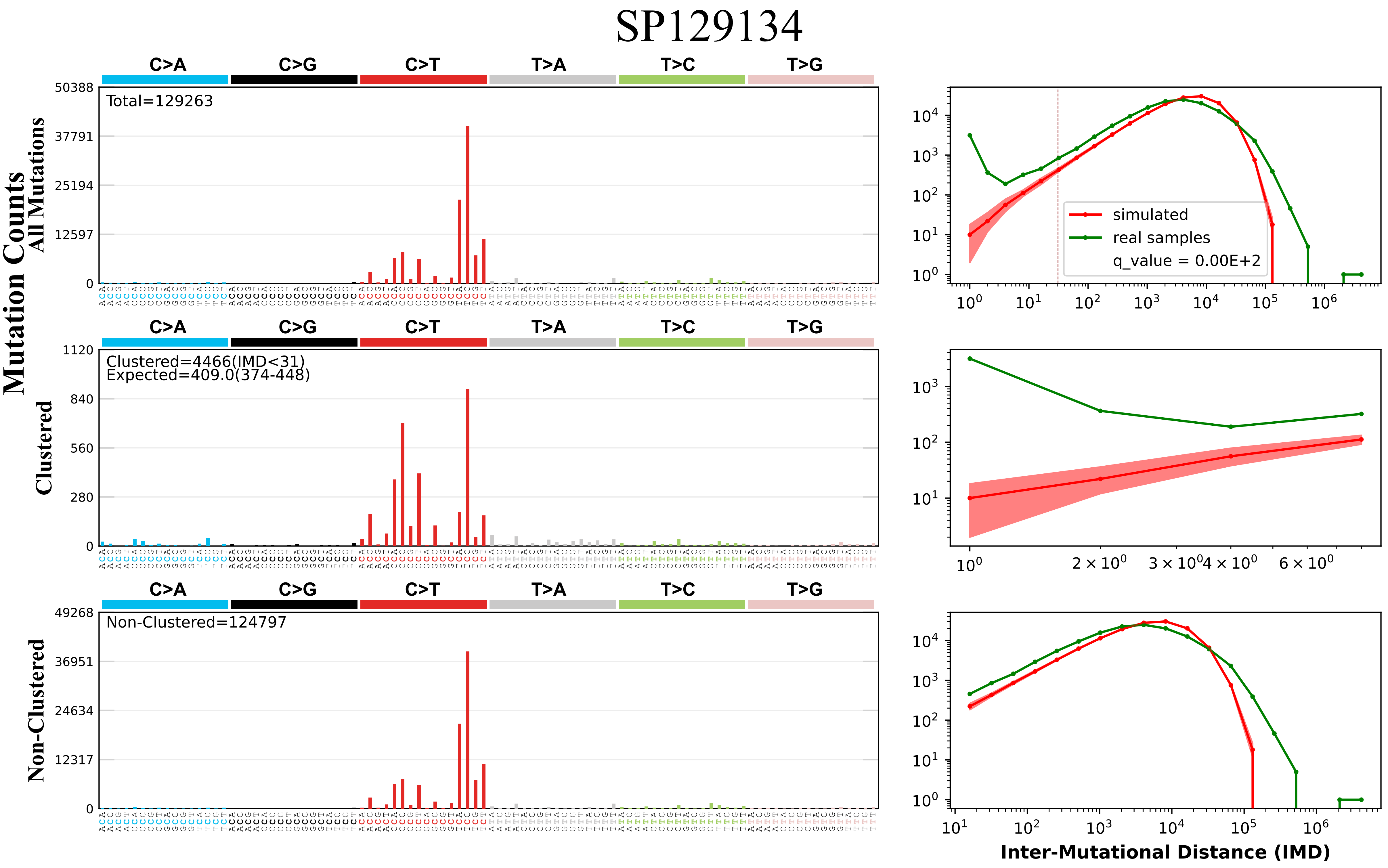
SP129134
C>A
C>G
C>T
T>A
T>C
T>G
Mutation Counts
All Mutations
Total=129263
50388
37791
25194
12597
0
Clustered
Clustered=4466(IMD<31)
Expected=409.0(374-448)
1120
840
560
280
0
Non-Clustered
Non-Clustered=124797
49268
36951
24634
12317
0
simulated
real samples
q_value = 0.00E+2
Inter-Mutational Distance (IMD)

SP124434

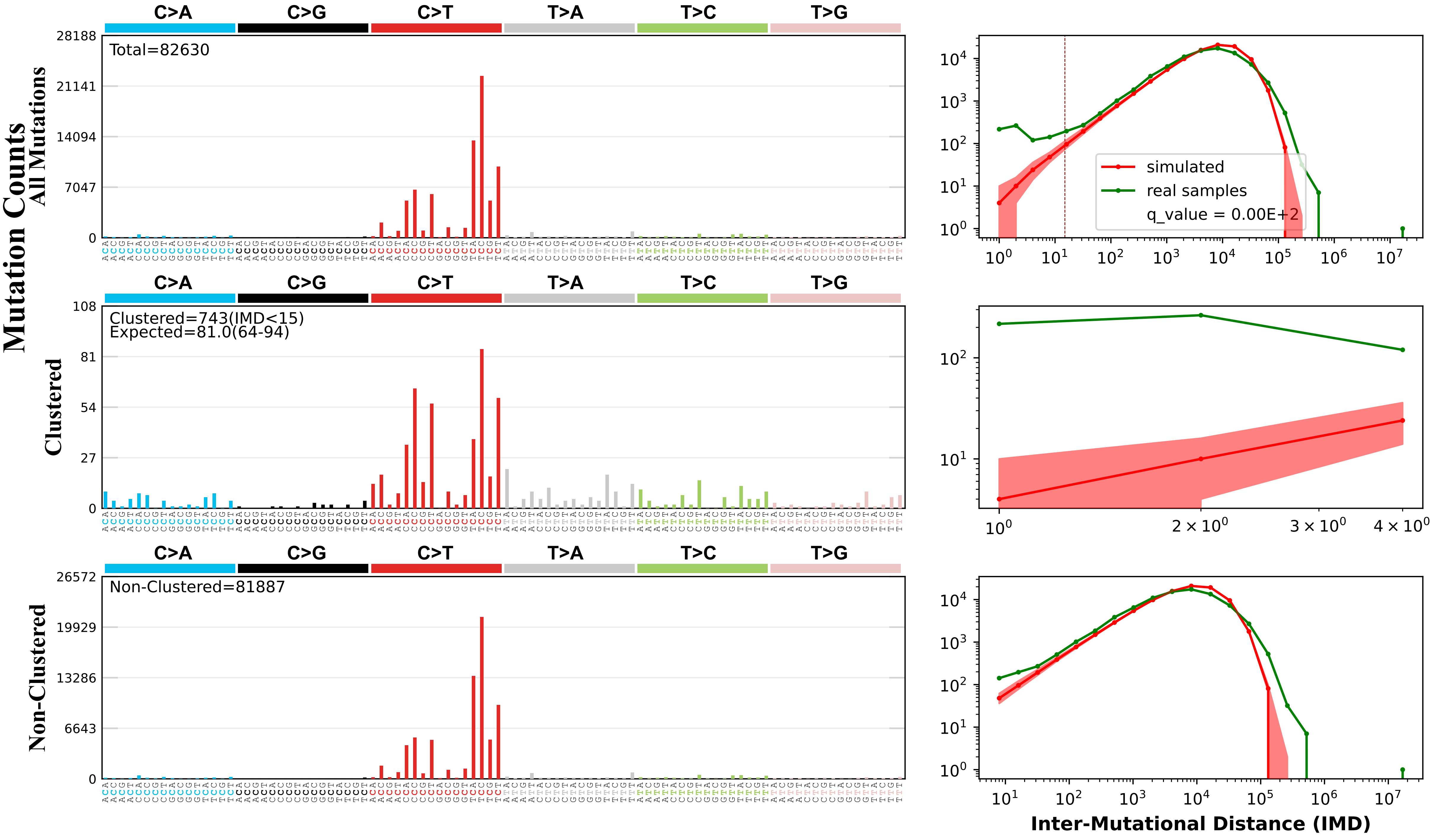

Mutation Counts
All Mutations
Clustered
Non-Clustered
C>A
C>G
C>T
T>A
T>C
T>G
Total=82630
Clustered=743(IMD<15)
Expected=81.0(64-94)
Non-Clustered=81887
simulated
real samples
q_value = 0.00E+2
Inter-Mutational Distance (IMD)

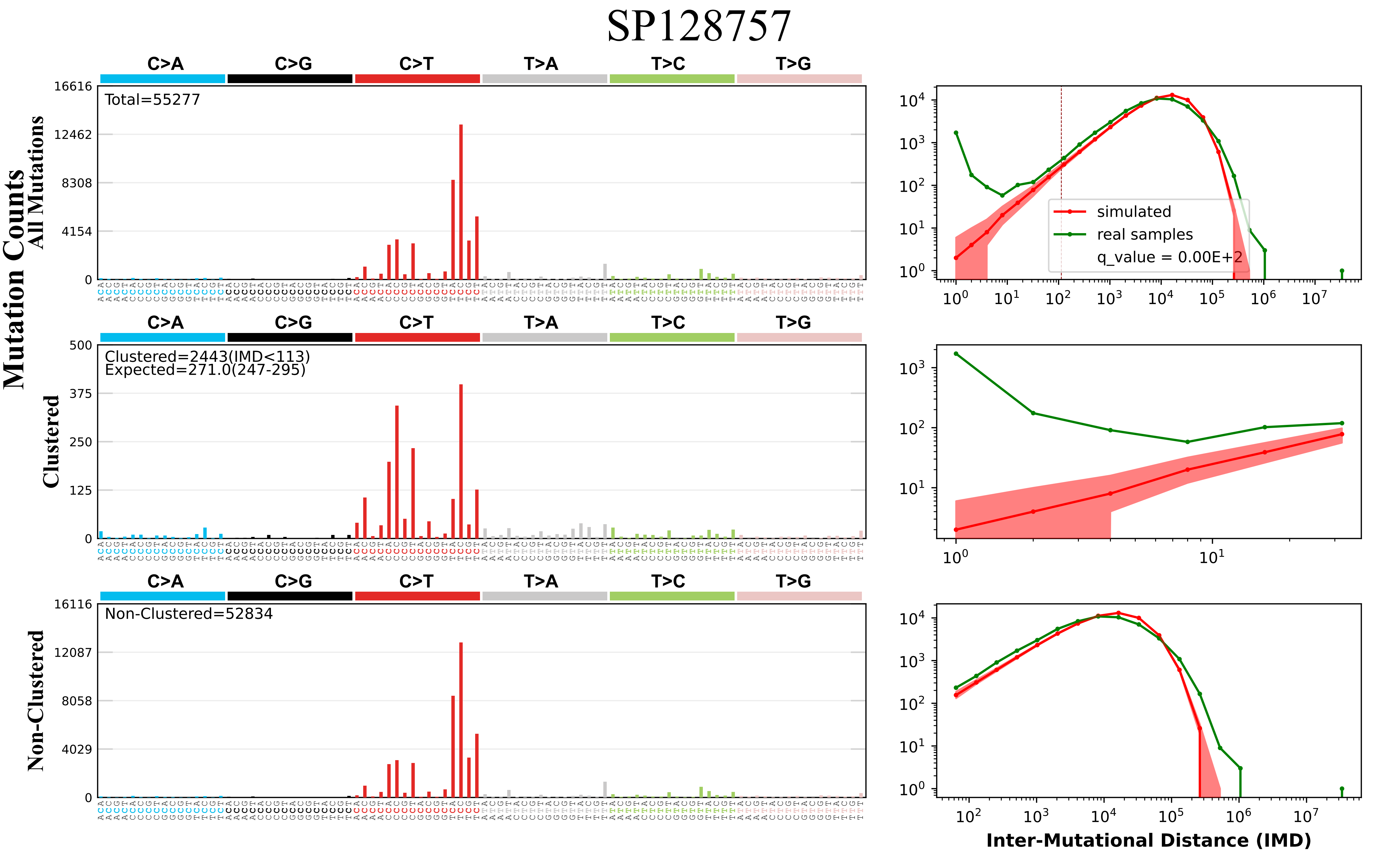
SP128757
Mutation Counts
All Mutations
Total=55277
Clustered
Clustered=2443(IMD<113)
Expected=271.0(247-295)
Non-Clustered
Non-Clustered=52834
C>A
C>G
C>T
T>A
T>C
T>G
simulated
real samples
q_value = 0.00E+2
Inter-Mutational Distance (IMD)

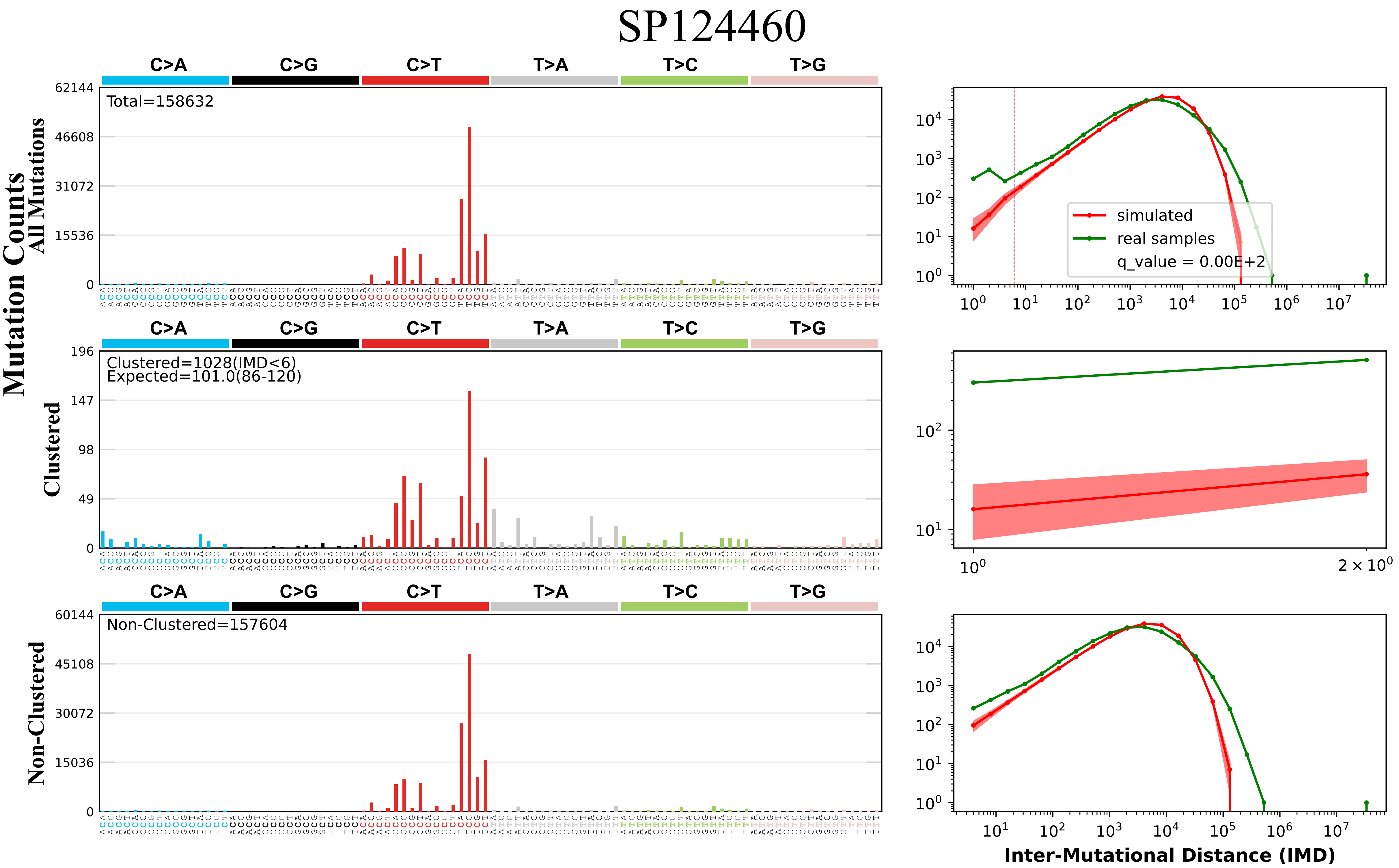
SP124460
Mutation Counts
All Mutations
Total=158632
Clustered
Clustered=1028(IMD<6)
Expected=101.0(86-120)
Non-Clustered
Non-Clustered=157604
C>A
C>G
C>T
T>A
T>C
T>G
simulated
real samples
q_value = 0.00E+2
Inter-Mutational Distance (IMD)

SP124365

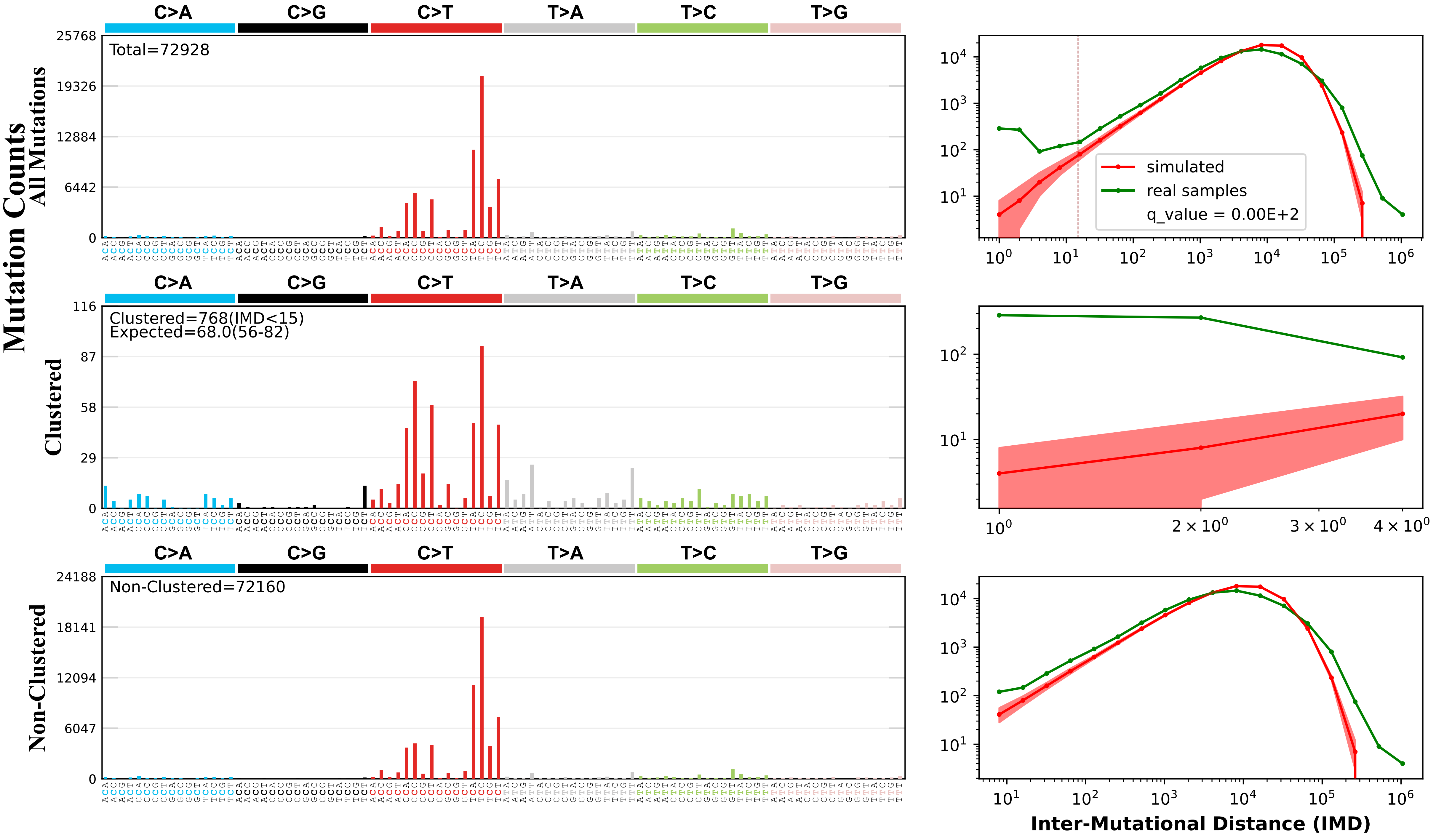

Mutation Counts
All Mutations
Clustered
Non-Clustered
C>A
C>G
C>T
T>A
T>C
T>G
Total=72928
Clustered=768(IMD<15)
Expected=68.0(56-82)
Non-Clustered=72160
simulated
real samples
q_value = 0.00E+2
Inter-Mutational Distance (IMD)

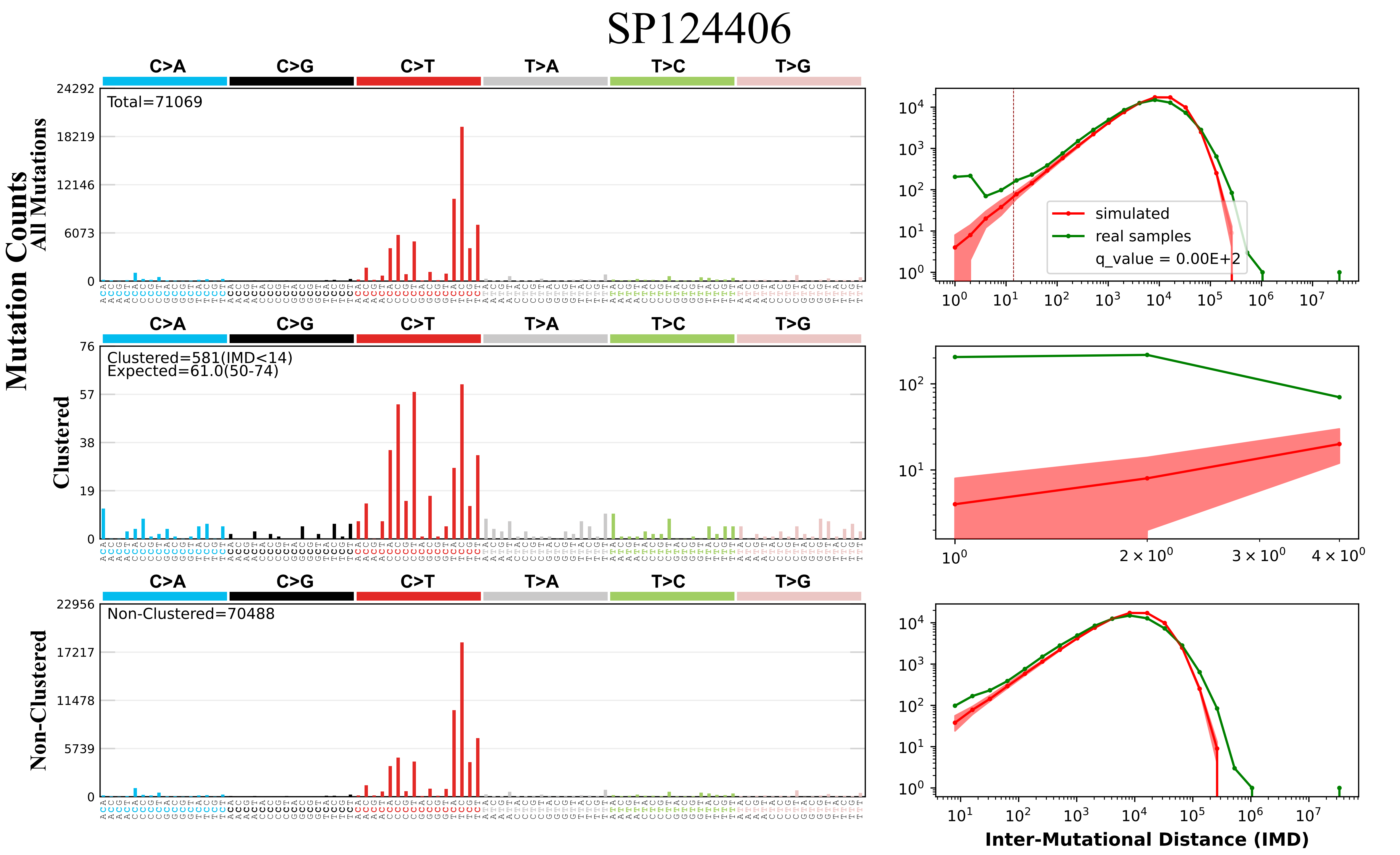
SP124406
Mutation Counts
All Mutations
Total=71069
Clustered
Clustered=581(IMD<14)
Expected=61.0(50-74)
Non-Clustered
Non-Clustered=70488
C>A
C>G
C>T
T>A
T>C
T>G
simulated
real samples
q_value = 0.00E+2
Inter-Mutational Distance (IMD)

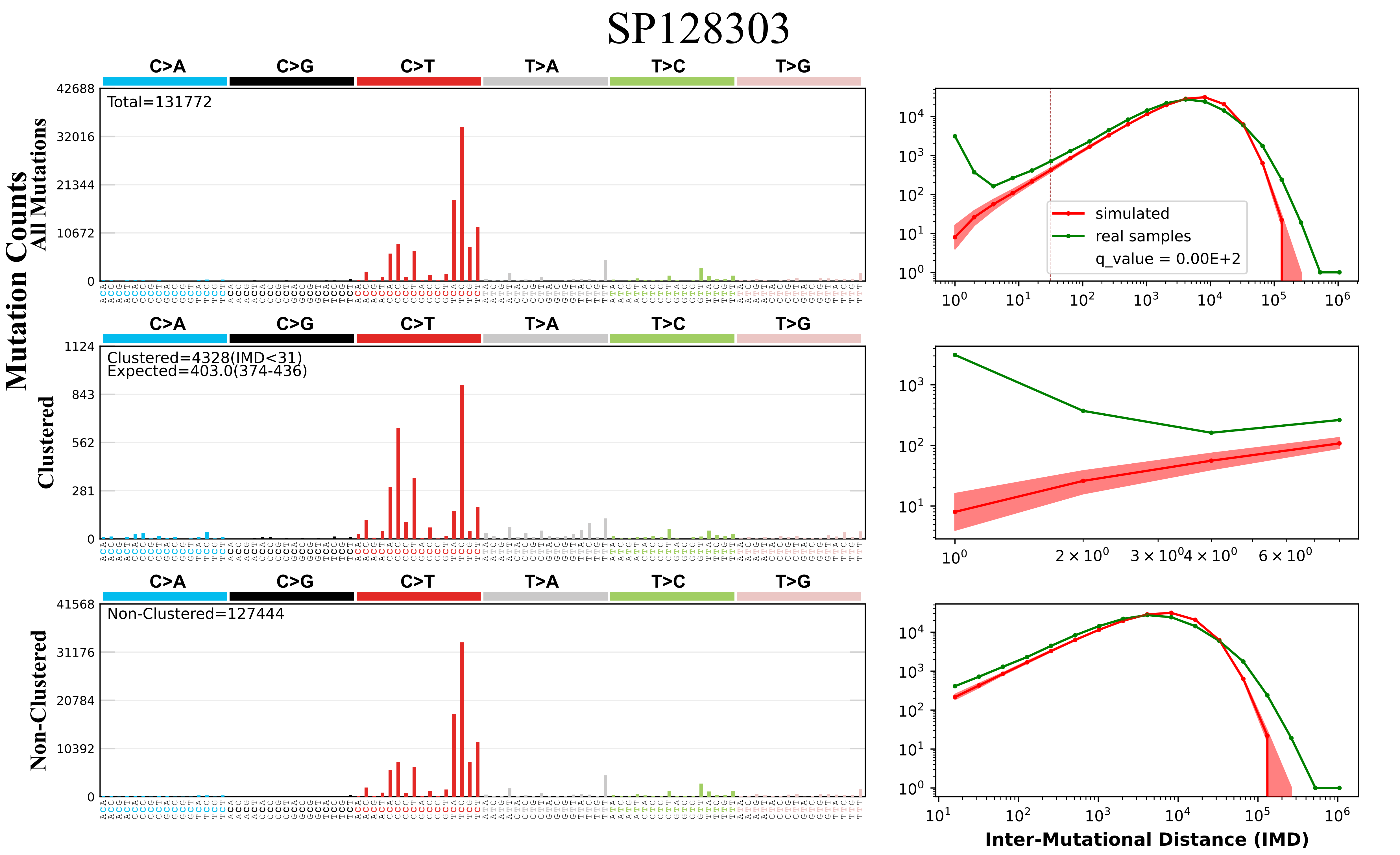

SP128303
Mutation Counts
All Mutations
Total=131772
Clustered
Clustered=4328(IMD<31)
Expected=403.0(374-436)
Non-Clustered
Non-Clustered=127444
C>A
C>G
C>T
T>A
T>C
T>G
simulated
real samples
q_value = 0.00E+2
Inter-Mutational Distance (IMD)

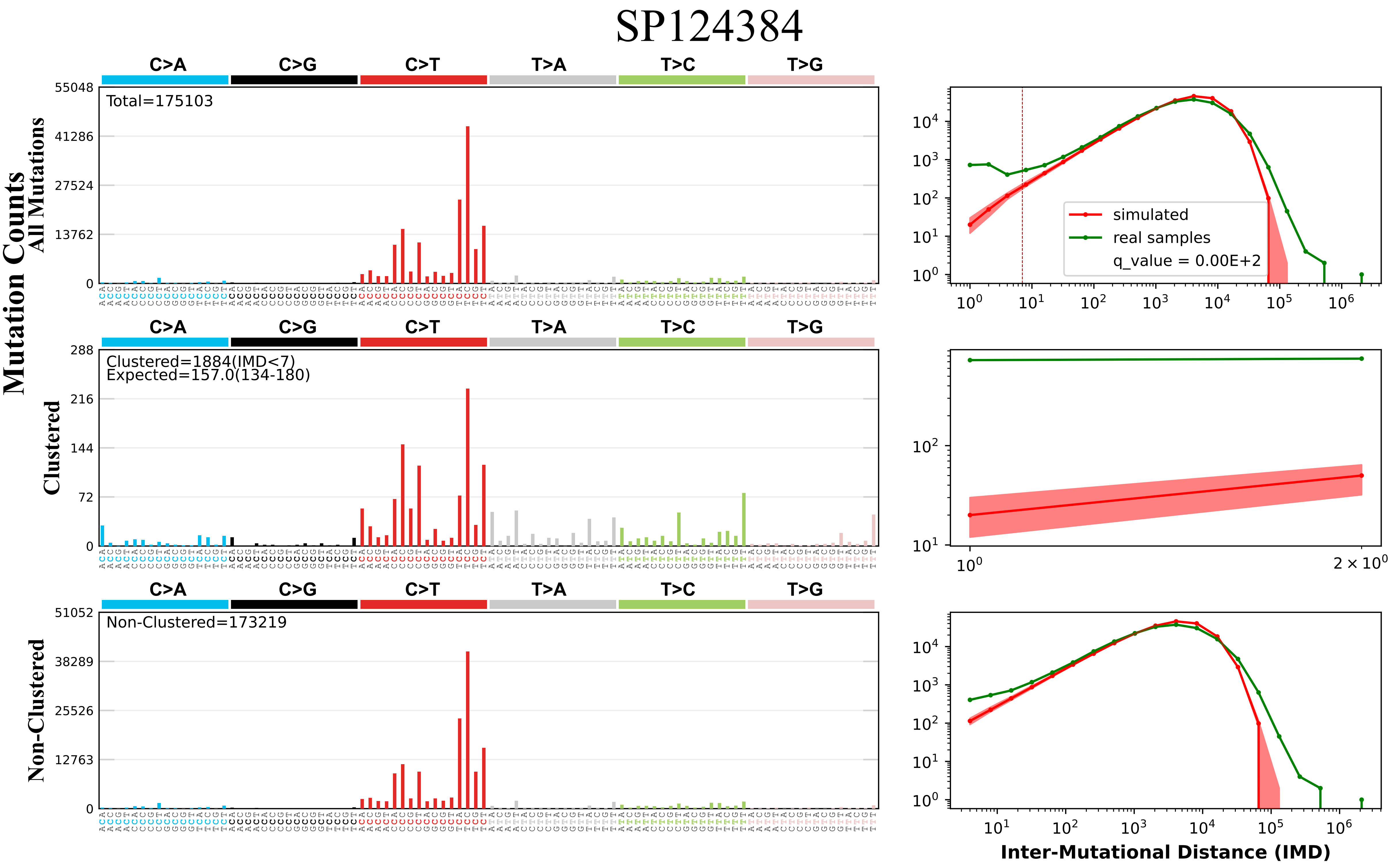
SP124384
Mutation Counts
All Mutations
Clustered
Non-Clustered
C>A
C>G
C>T
T>A
T>C
T>G
Total=175103
Clustered=1884(IMD<7)
Expected=157.0(134-180)
Non-Clustered=173219
simulated
real samples
q_value = 0.00E+2
Inter-Mutational Distance (IMD)

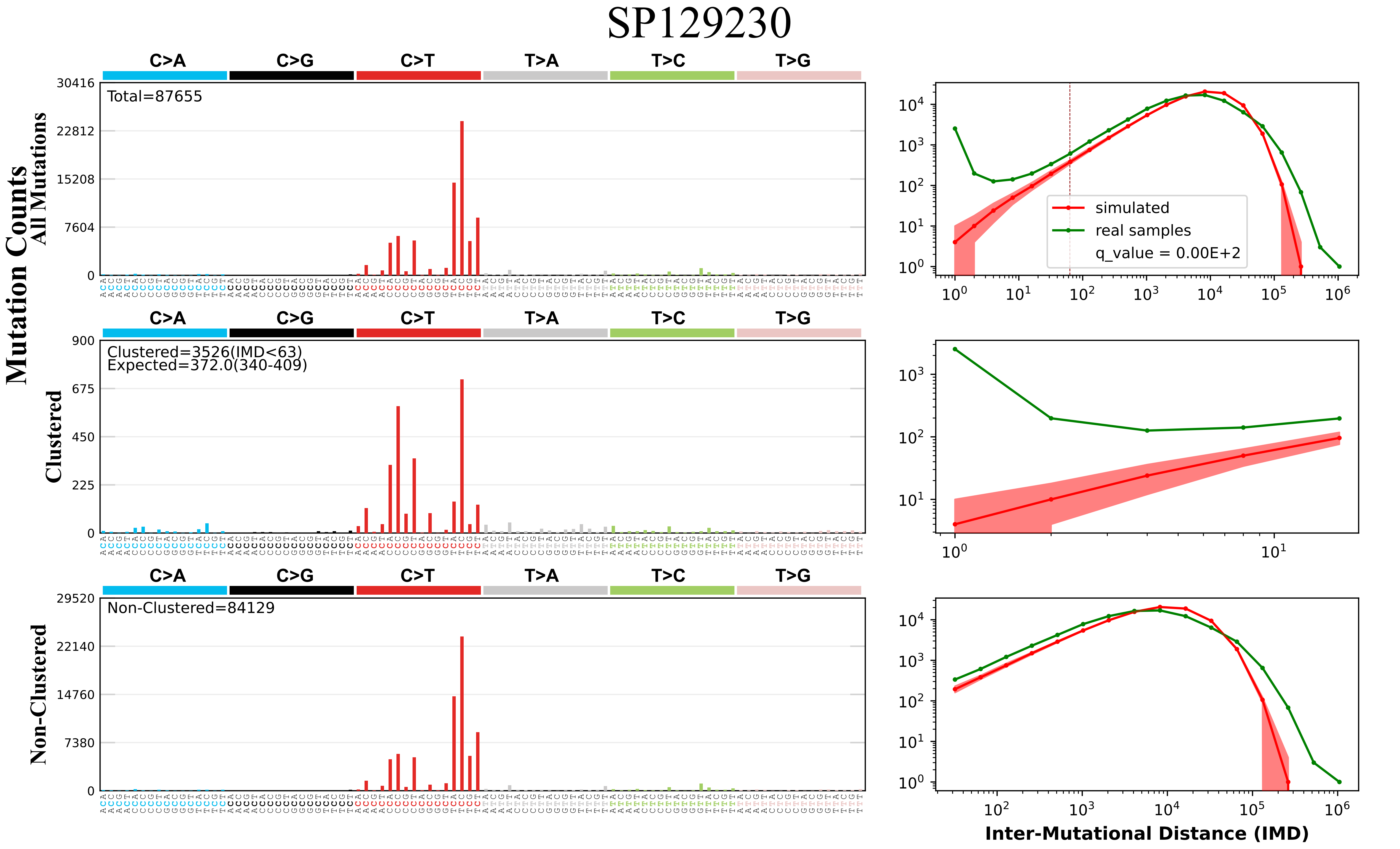
SP129230
Mutation Counts
All Mutations
Total=87655
Clustered
Clustered=3526(IMD<63)
Expected=372.0(340-409)
Non-Clustered
Non-Clustered=84129
C>A
C>G
C>T
T>A
T>C
T>G
simulated
real samples
q_value = 0.00E+2
Inter-Mutational Distance (IMD)

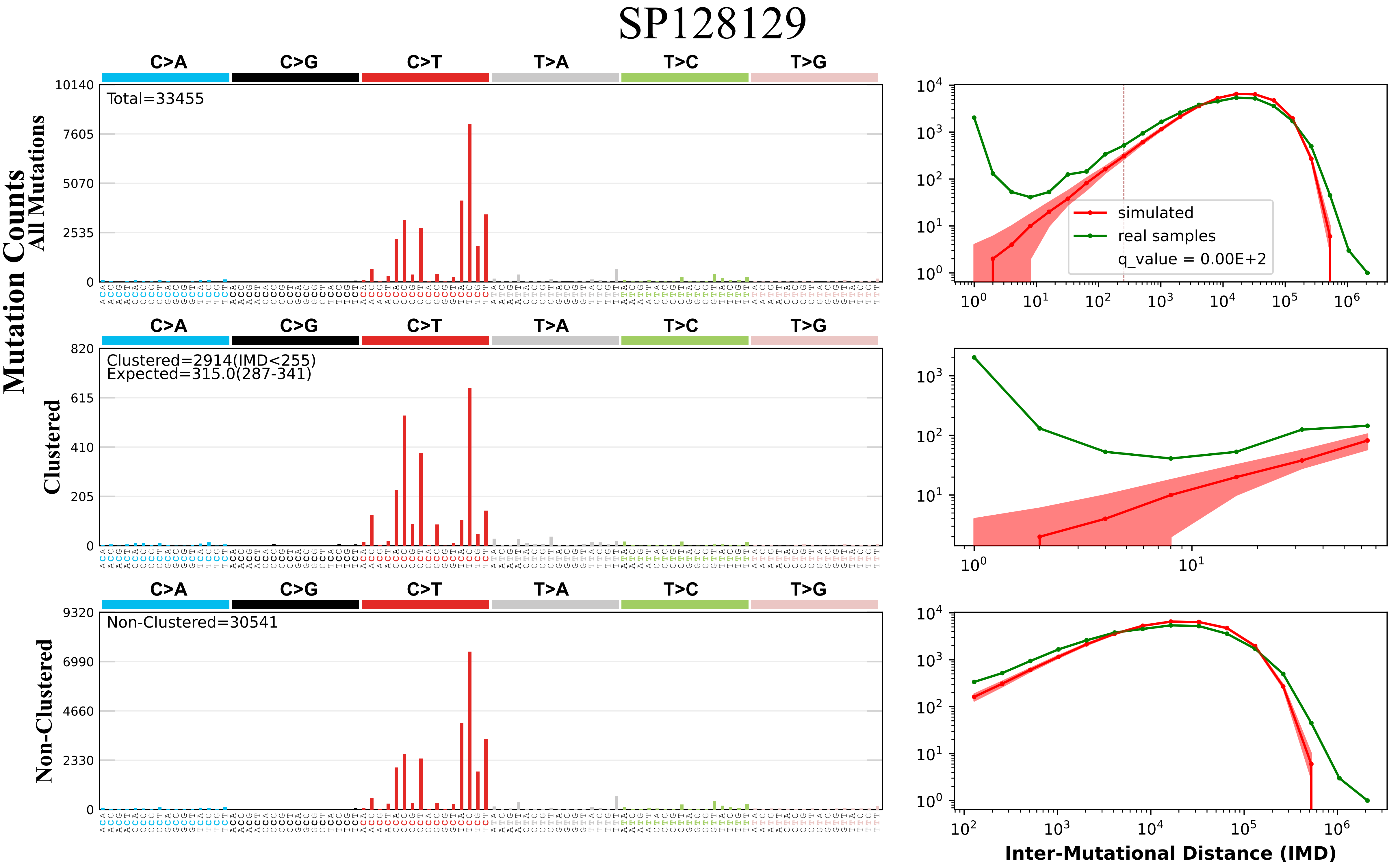
SP128129
Mutation Counts
All Mutations
Clustered
Non-Clustered
C>A
C>G
C>T
T>A
T>C
T>G
Total=33455
Clustered=2914(IMD<255)
Expected=315.0(287-341)
Non-Clustered=30541
simulated
real samples
q_value = 0.00E+2
Inter-Mutational Distance (IMD)

fa4d35e3-fc38-4e26-a9e0-521a648acec1

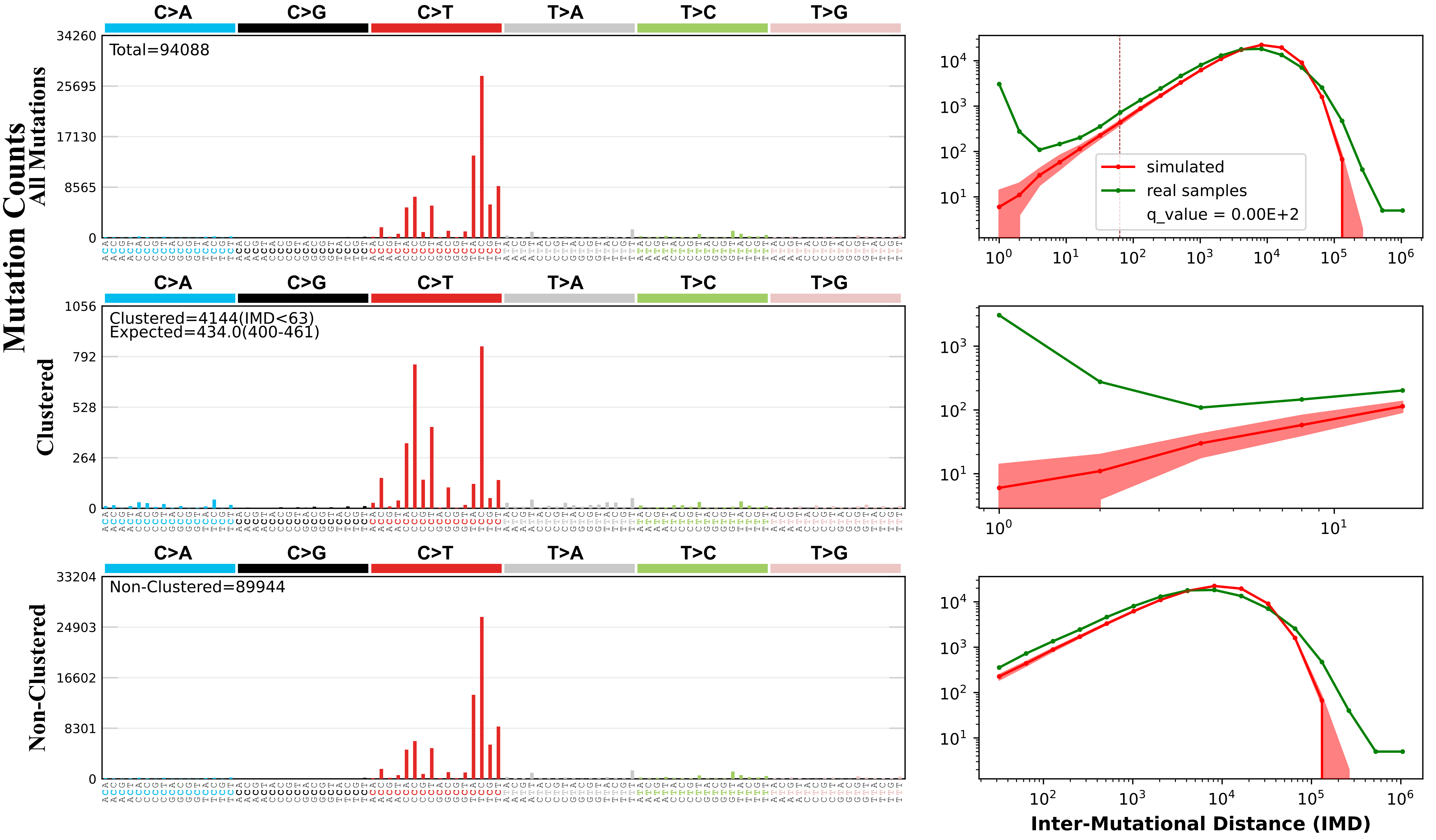

SP128316

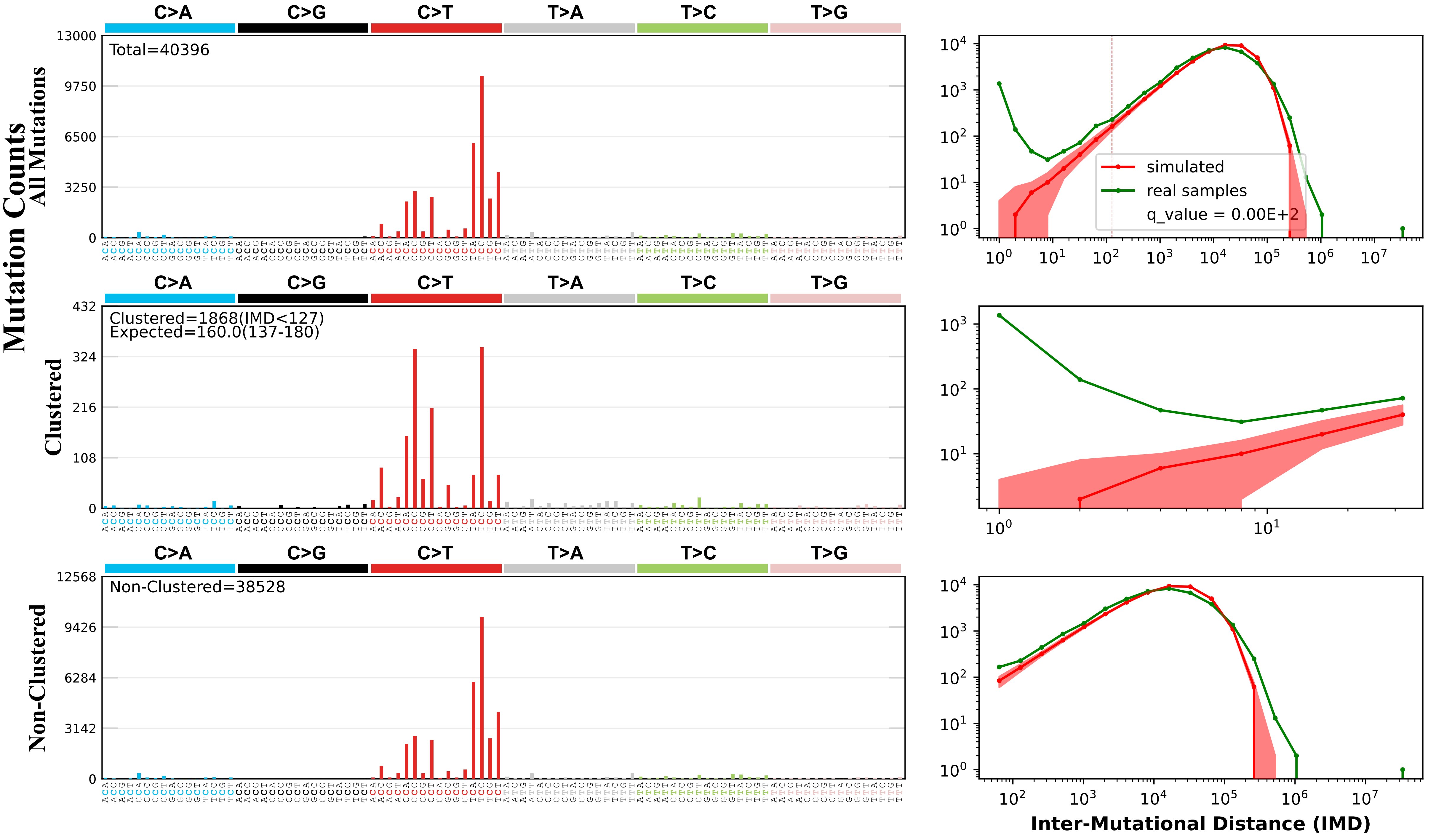
Mutation Counts
All Mutations
C>A
C>G
C>T
T>A
T>C
T>G
Total=40396
13000
9750
6500
3250
0
Clustered
Clustered=1868(IMD<127)
Expected=160.0(137-180)
432
324
216
108
0
Non-Clustered
Non-Clustered=38528
12568
9426
6284
3142
0
simulated
real samples
q_value = 0.00E+2
Inter-Mutational Distance (IMD)

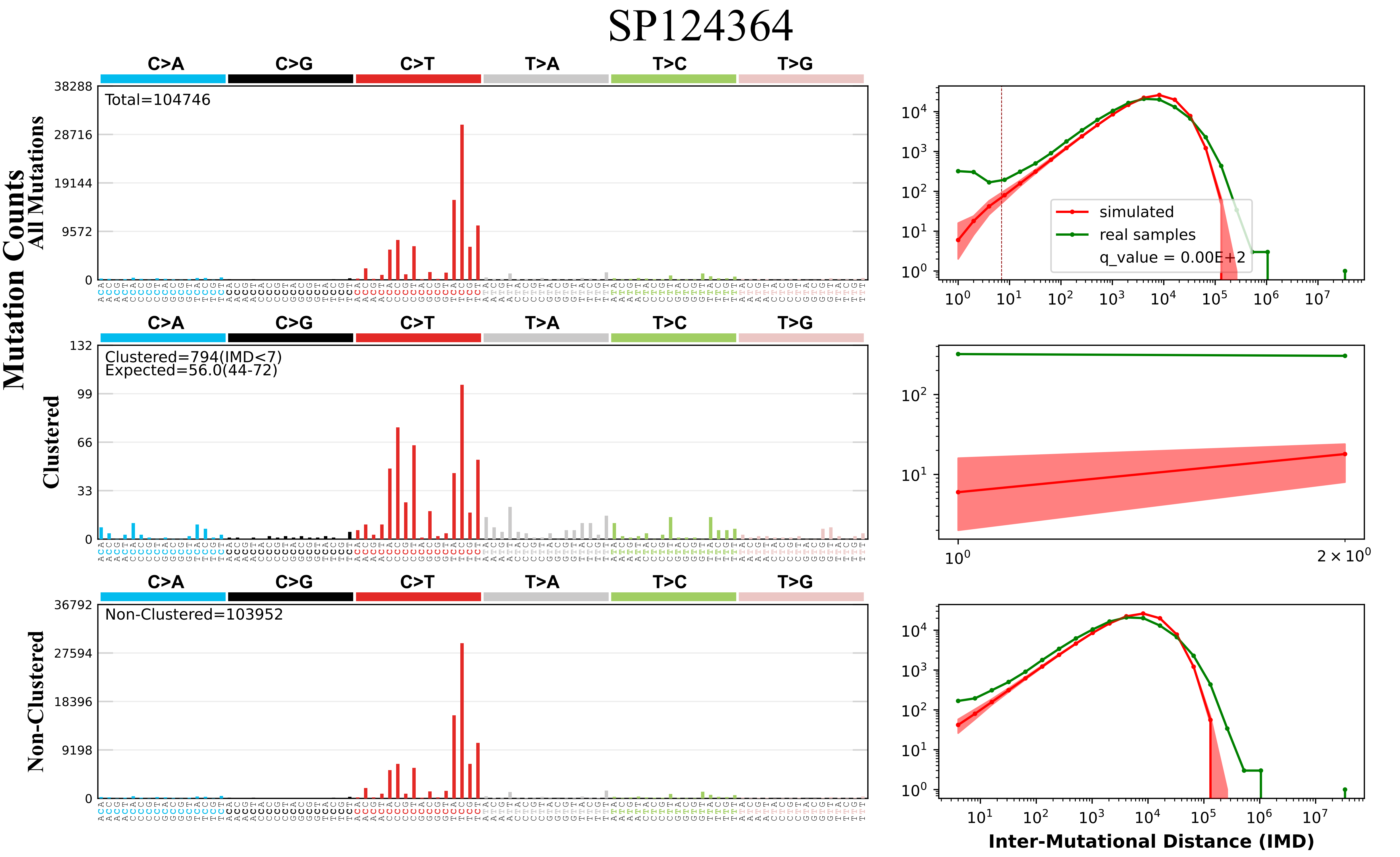

SP124364
C>A
C>G
C>T
T>A
T>C
T>G
Total=104746
Clustered=794(IMD<7)
Expected=56.0(44-72)
Non-Clustered=103952
Mutation Counts
All Mutations
Clustered
Non-Clustered
simulated
real samples
q_value = 0.00E+2
Inter-Mutational Distance (IMD)

SP124418

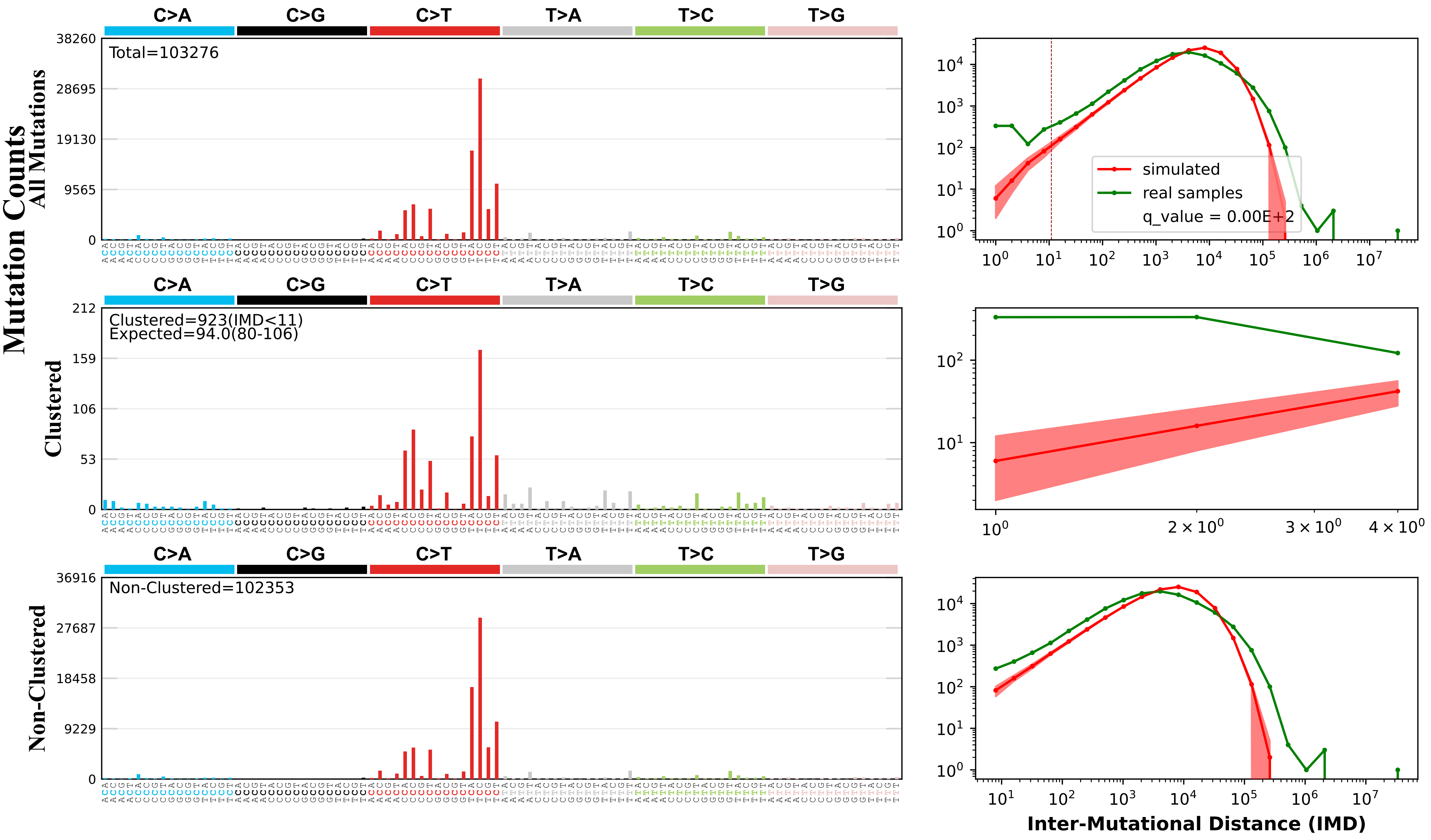

Mutation Counts
All Mutations
C>A
C>G
C>T
T>A
T>C
T>G
38260
28695
19130
9565
0
Total=103276
Clustered
212
159
106
53
0
Clustered=923(IMD<11)
Expected=94.0(80-106)
Non-Clustered
36916
27687
18458
9229
0
Non-Clustered=102353
simulated
real samples
q_value = 0.00E+2
Inter-Mutational Distance (IMD)

affa4ef4-02ec-40f1-b6a2-739f8bf2afa9

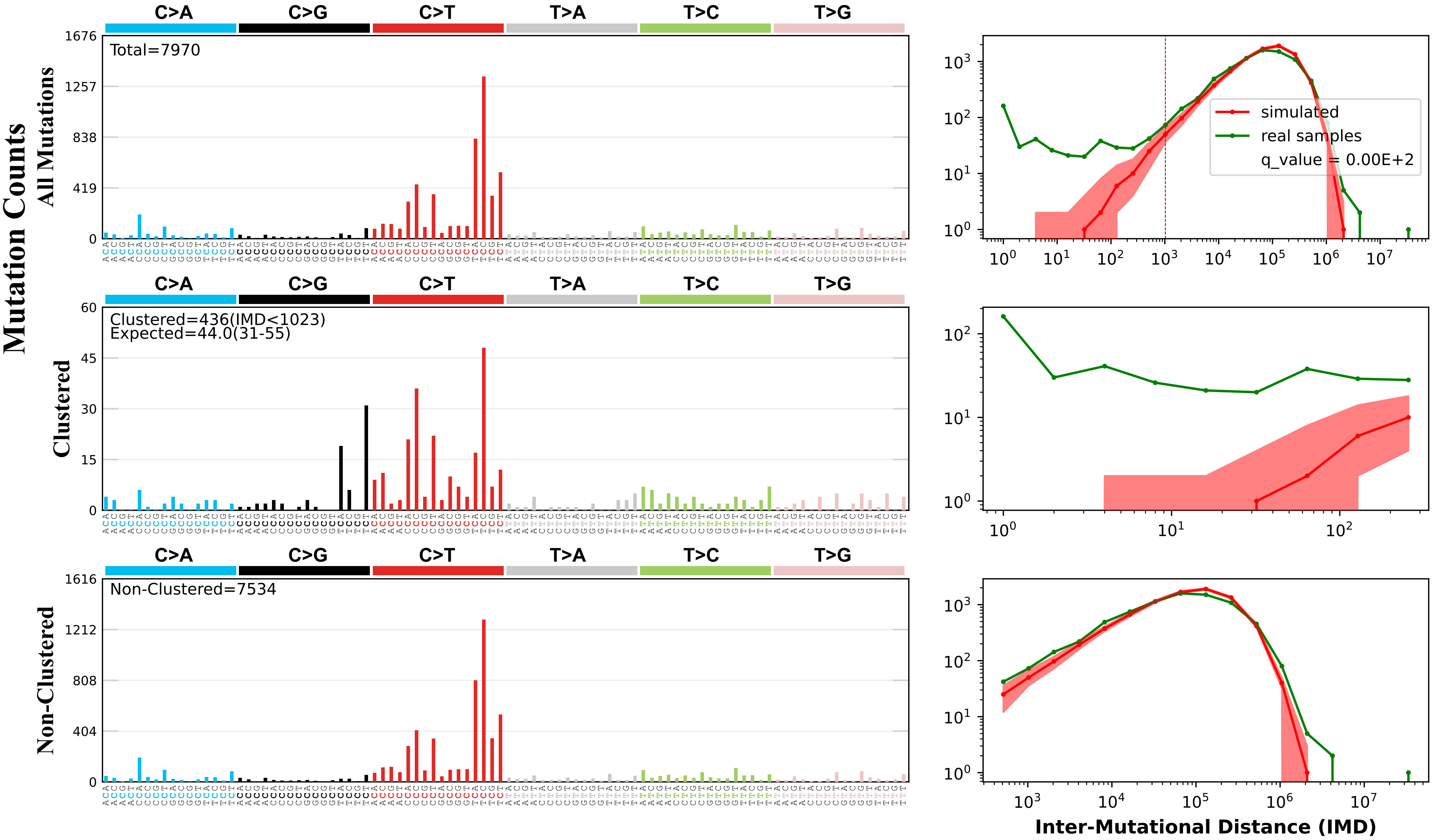

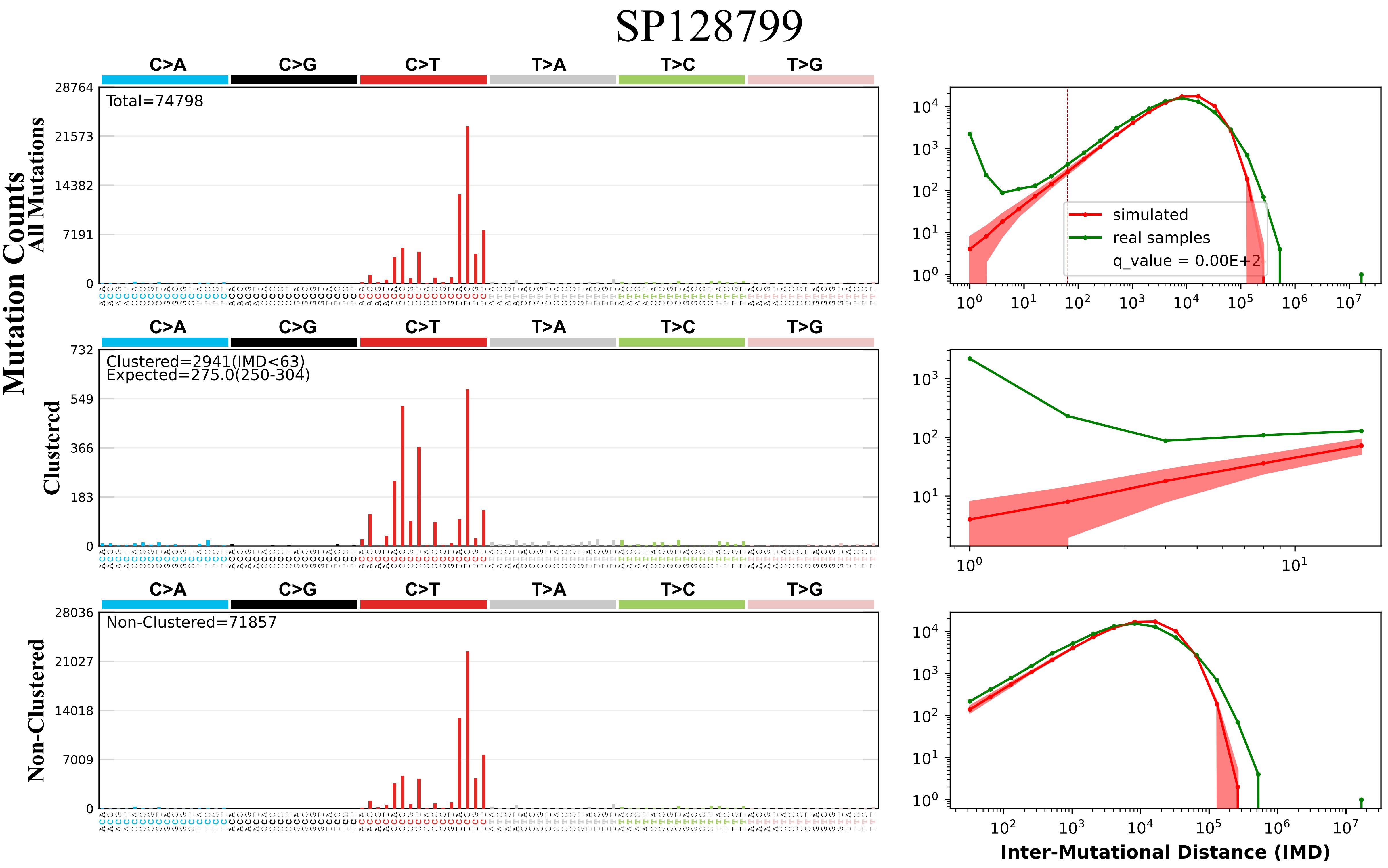
SP128799
Mutation Counts
All Mutations
Clustered
Non-Clustered
C>A
C>G
C>T
T>A
T>C
T>G
Total=74798
28764
21573
14382
7191
0
Clustered=2941(IMD<63)
Expected=275.0(250-304)
732
549
366
183
0
Non-Clustered=71857
28036
21027
14018
7009
0
simulated
real samples
q_value = 0.00E+2
Inter-Mutational Distance (IMD)
